# Lung development genes, adult lung function and cardiovascular comorbidities

**DOI:** 10.1136/thorax-2024-222474

**Published:** 2025-05-30

**Authors:** Laura Portas, Mohammad Talaei, Charlotte Dean, Nay Aung, Matthew David Hind, Alfred Pozarickij, Robin G Walters, China Kadoorie Biobank Collaborative Group, Junshi Chen, Peter GJ Burney, Steffen Petersen, Cosetta Minelli, Seif O Shaheen

**Affiliations:** 1 National Heart and Lung Institute, Imperial College London, London, UK; 2 Nuffield Department of Population Health, University of Oxford, Oxford, UK; 3 Li Ka Shing Centre for Health Information and Discovery, University of Oxford, Oxford, UK; 4 Wolfson Institute of Population Health, Barts and The London School of Medicine and Dentistry, Queen Mary University of London, London, UK; 5 National Heart and Lung Institute, Division of Respiratory Science, London, UK; 6 Barts and The London School of Medicine and Dentistry William Harvey Research Institute, London, UK; 7 Saint Bartholomew’s Hospital Barts Heart Centre, London, UK; 8 Respiratory Medicine, Royal Brompton Hosital, London, UK; 9 Centre for Advanced Cardiovascular Imaging, Barts and The London School of Medicine an Dentistry, London, UK

**Keywords:** respiratory measurement, lung physiology

## Abstract

**ABSTRACT:**

**Background:**

The association between lower adult lung function and increased cardiovascular comorbidity has not been adequately explained. We investigated whether shared developmental signalling pathways, critical to lung development and repair, could partly explain it.

**Methods:**

In UK Biobank (UKB), we performed pairwise colocalisation analysis of variants in 55 lung development genes associated with adult forced vital capacity (FVC) or forced expiratory volume in 1 s (FEV_1_)/FVC, to see if these are also associated with coronary heart disease (CHD), blood pressure (systolic, diastolic, hypertension), pulse pressure, Arterial Stiffness index and carotid intima-media thickness. For CHD, we meta-analysed data from UKB and the CARDIoGRAM consortium.

**Results:**

We found that 12 of the 55 genes shared the same variant between one (or more) lung function trait and one (or more) cardiovascular trait (H4colocalisation). The direction of effects was always in keeping with our hypothesis (lower lung function–higher cardiovascular risk) for FVC, but not always for FEV_1_/FVC. The seven signals for hypertension and CHD all replicated nominally in the FinnGen study, while replication was poor in the China Kadoorie Biobank (CKB) study. In addition, we found a further 10 genes where genetic associations with lung function and cardiovascular traits were within the same gene but involved different variants (H3 colocalisation). Interestingly, six of all 22 genes (H4 and H3 colocalisation) were novel for cardiovascular traits; four replicated in FinnGen, three in CKB.

**Conclusion:**

Lung function and cardiovascular traits have shared developmental pathways that may partly explain why lower lung function, especially FVC, is associated with increased cardiovascular risk.

**WHAT IS ALREADY KNOWN ON THIS TOPIC**

- Lower adult lung function, particularly a restrictive pattern, has been strongly linked to higher cardiovascular mortality and morbidity even after adjusting for shared risk factors, but this association has not been fully explained.

**WHAT THIS STUDY ADDS**

- We show that lung development pathways influencing adult lung function may also influence adult cardiovascular outcomes, thus partly explaining the link between lower adult lung function and higher cardiovascular risk.
- The same variant in 12 genes was associated with a lung function trait and one or more cardiovascular traits; importantly, variants in nine of these genes showed direction of effects supporting our hypothesis.

**HOW THIS STUDY MIGHT AFFECT RESEARCH, PRACTICE OR POLICY**

- Our findings could inform future research on druggable targets aimed at optimising lung and cardiovascular development and repair.

## Introduction

 Lower adult lung function, especially a restrictive spirometry pattern characterised by a lower forced vital capacity (FVC), is strongly associated with higher cardiovascular (CV) mortality.^1 2^ This association is not explained by smoking; in UK Biobank (UKB), among never-smokers, those in the lowest quartile of FVC had more than double the risk of death from a circulatory disease compared with those in the highest quartile.^3^ Even in young adults, lower lung function has been associated with premature CV mortality.^4^ Lower lung function is also associated with incident coronary heart disease (CHD),^5^ hypertension (HTN),^6^ increased carotid intima-media thickness (CIMT)^7^ and central arterial stiffness.^8^ Compared with other lifetime spirometric trajectories, a restrictive-only pattern is associated with the highest prevalence of CV comorbidities.^9^ These associations persist after adjustment for other adult risk factors, including inflammatory markers,^10^ and have not been adequately explained.

Lower lung function in late adult life is a measure of poorer lung health and may arise through suboptimal lung development and a failure to attain maximal capacity as a young adult.^11^,^13^ This developmental paradigm for lung ageing complements the alternative model of accelerated lung function decline in later life. It has been mooted that the environmental and genetic factors that influence lung development might also affect the development of other organ systems.^14^ For example, low birth weight, a marker of impaired fetal growth, has been implicated in the aetiology of both restrictive lung function impairment and increased CV risk and mortality.^15 16^ A limitation of observational studies implicating the early environment is the possibility of spurious findings caused by environmental and lifestyle confounding, a problem which does not affect genetic studies. Investigating genetic factors influencing organ development can therefore strengthen causal inference regarding pathogenetic mechanisms related to early life, as well as provide insight into the role of specific developmental signalling pathways.

In a previous study, we provided further evidence that lung development plays a crucial role in adult lung health.^17^ We identified (and externally replicated) 55 lung development genes associated with adult lung function in UKB, influencing both restrictive and obstructive patterns, 36 of which had not been previously identified in genome-wide association studies (GWAS). The fundamental function of the majority of the 55 genes is to regulate organ size and cell integrity. We therefore propose that signalling pathways, which are critical to lung development and repair, are also important for the development and repair of the CV system and that perturbation of these shared developmental pathways may partly explain the association between lower adult lung function, especially FVC, and CV comorbidities.

We investigate this hypothesis by looking for shared genetic signals between adult lung function and CV traits in the 55 genes, using colocalisation analysis^18^ applied to UKB data, complemented by CHD data from the international CARDIoGRAM consortium.^19^ We sought replication of our main CV findings in the Finnish FinnGen study^20^ and in the China Kadoorie Biobank (CKB).^21^

## Methods

### Data

#### UK Biobank

UKB is a study of about 500 000 volunteers aged 40–69 years from the UK, with information on risk factors for chronic disease and related traits, and genetic data.^22^ Here, we included participants of self-reported white ethnicity with good quality lung function data. Details on UKB data and variables used are reported in online supplemental methods and table S1.

We analysed:

- FVC and forced expiratory volume in 1 s (FEV_1_) to FVC ratio (FEV_1_/FVC; n=3 06 476).
- Blood pressure (n=369 905): systolic and diastolic blood pressure (SBP, DBP); HTN (n=380 689; 217 771 cases, SBP ≥140 or DBP ≥90 mm Hg or antihypertensive treatment)
- Pulse pressure (PP) (n=369 905), calculated as the difference between SBP and DBP
- Arterial Stiffness Index (ASI) (n=55 041), measured using finger photo-plethysmography.
- CIMT (n=38 469), a measure of subclinical atherosclerosis, measured using ultrasound.
- CHD (n=405 570; 19 294 cases), defined as self-reported myocardial infarction or angina; coronary artery bypass or coronary angioplasty.

#### CARDIoGRAM

To increase statistical power for CHD analyses, we combined UKB data with data from CARDIoGRAMplusC4D (www.cardiogramplusc4d.org/data-downloads), resulting in a total of 80 095 CHD cases and 509 780 controls. CARDIoGRAMplusC4D is a GWAS meta-analysis including 60 801 CHD cases and 123 504 controls, mostly of European ancestry, with CHD defined as myocardial infarction, acute coronary syndrome, chronic stable angina or coronary stenosis >50%.^19^

### Statistical analysis

**Figure 1:**
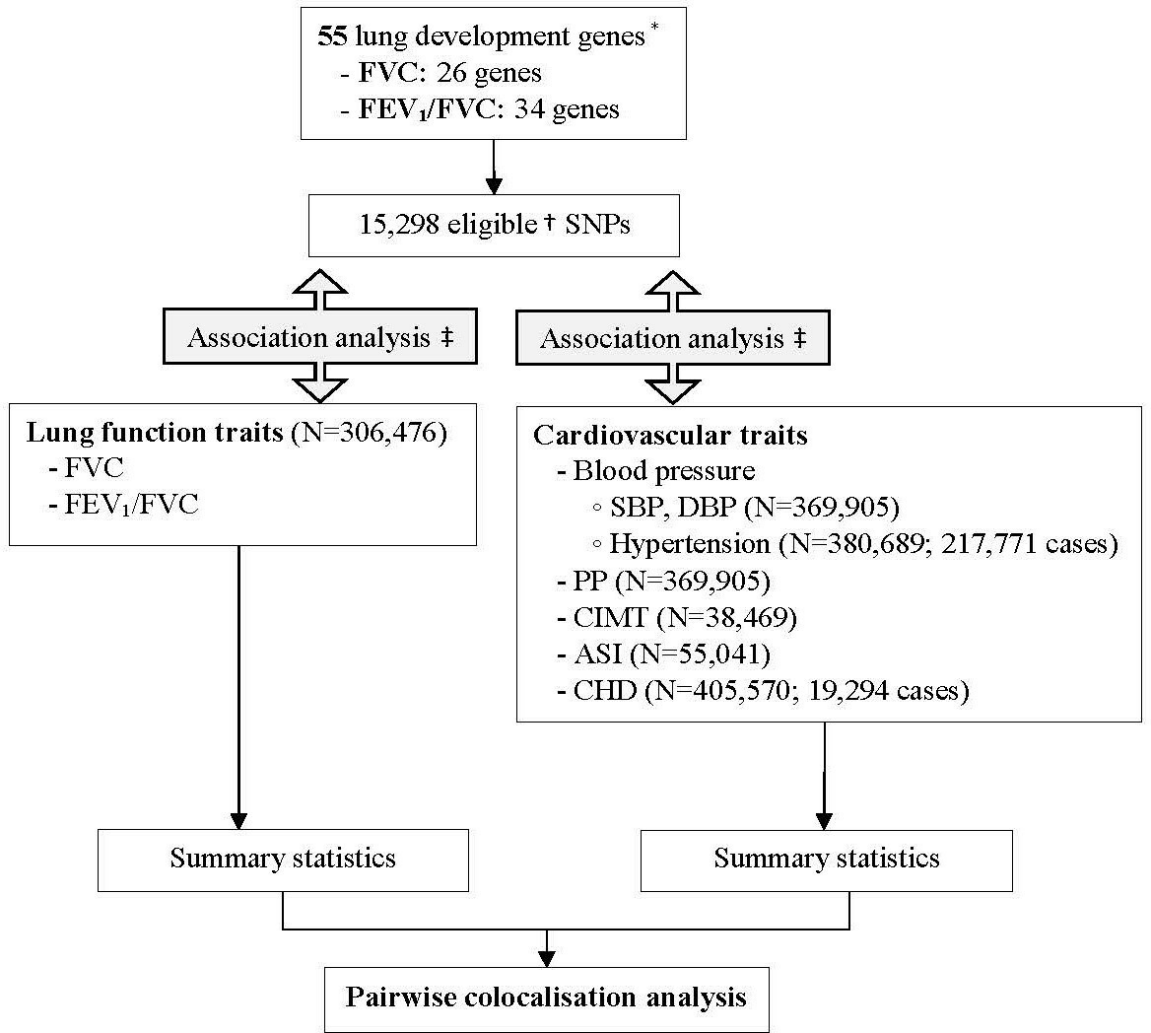
Analysis flow chart. *Previously identified in: Portas *et al*.^17^ †Minor allele frequency ≥1% and imputation quality ≥0.5 (online supplemental table S6). ‡Linear/Logistic regression analysis, adjusted for age, sex, genotyping array, centre and 10 ancestry principal components. Analysis of FVC additionally adjusted for height. ASI, Arterial Stiffness Index; CHD, coronary heart disease; CIMT, carotid intima-media thickness; DBP, diastolic blood pressure; FEV_1_, forced expiratory volume in 1 s; FVC, forced vital capacity; PP, pulse pressure; SBP, systolic blood pressure; SNP, single nucleotide polymorphism.

The analyses are summarised in figure 1. We investigated whether any of the 55 lung development genes we previously identified as associated with adult lung function^17^ share an effect on CV traits, through a colocalisation analysis of variants (single nucleotide polymorphisms (SNPs)) in the 55 genes, based on summary data. In our previous work, the 55 genes were identified from a list of 391 genes related to lung development prepared by two experts (CD and MDH). Briefly, they independently compiled initial lists based on knowledge from human and experimental data, then merged them into a single list, and extended it to include further genes identified from KEGG (Kyoto encyclopedia of genes and genomes) pathways and HuGE Navigator literature.^17^

We obtained summary statistics (beta and SE) for the association of all SNPs within the 55 lung development genes with lung function measures and with CV traits using Regenie,^23^ assuming an additive genetic model and adjusting for age, sex, genotyping array, centre and first 10 ancestry principal components. Analysis of FVC was additionally adjusted for height to ensure effects were independent of body size, and analysis of ASI for the device used. For CHD, a fixed-effect meta-analysis was used to combine UKB and CARDIoGRAM summary statistics.

We applied colocalisation analysis to all SNPs within the boundaries of the 55 genes with minor allele frequency ≥1% and imputation quality ≥0.5 (online supplemental table S2). Pairwise colocalisation analysis (each lung function measure vs each CV trait) was conducted using *coloc.abf* in the R package *coloc*.^18^ Colocalisation analysis in *coloc* is performed using a Bayesian framework; while there is no need to correct for multiple testing, prior beliefs about SNP-specific associations under each scenario must be specified and the choice of priors justified (see online supplemental methods). The Bayesian approach provides an estimate of the posterior probability that a genetic variant is shared between the traits studied, based on probabilistic models that incorporate the prior beliefs and the observed data.^18^ In particular, results are expressed as posterior probability of association under each of five possible scenarios: H0=no association with either trait; H1=association with trait 1 (lung function trait), but not trait 2 (CV trait); H2=association with trait 2, but not trait 1; H3=association with both traits, but separate variants; H4=association with both traits, same variant.

The term ‘colocalisation’ has been used, strictly speaking, to refer to a shared association with the same variant (H4), but colocalisation analysis also allows the identification of associations with different variants within the same gene (H3). Although our main interest is in H4, we broadly refer to colocalisation to indicate either scenario. This is because the focus of our study is on genes rather than individual variants, and H3 complements H4 in supporting our hypothesis of shared genetic effects of lung development genes on adult lung function and CV comorbidities. Colocalisation was defined as a posterior probability ≥0.70 of either one common associated variant (H4) or two distinct associated variants (H3, ‘*regional*’ posterior probability).

For H4 findings, we investigated the direction of the two genetic effects by looking at the summary results for the lung function and CV traits used in the colocalisation analysis. We included in the H4 findings the case where colocalisation was at two variants in high linkage disequilibrium (LD), which we refer to as ‘H4 equivalent’. ‘H4-equivalent’ cases were identified using fine mapping analysis (*finemap.abf* in *coloc*) to pinpoint the two variants with the highest posterior probability for each trait, and checking whether they were in high LD (r²>0.8).

### Sensitivity analysis

Only prebronchodilator lung function data are available in UKB, so we could not rule out that a lower FEV_1_/FVC might be due to reversible airflow obstruction, caused by asthma. As asthma and HTN have previously shown genetic overlap,^24^ we repeated the FEV_1_/FVC analyses after excluding participants with self-reported doctor-diagnosed asthma (≅12% of the sample).

### Replication

We sought replication of the CV associations for the shared variants identified by our H4 findings using the publicly available FinnGen dataset (n=377 277),^20^ where GWAS results are available for two of our CV traits, HTN (n=111 581 cases) and CHD (n=43 518 cases). We also sought replication in CKB (sample sizes ranging from 22 543 for CIMT to 100 453 for blood pressure),^21^ where all our CV traits are available. We defined replication as an effect size in the same direction as in UKB, and for this reason, we used a one-sided p value. We applied Bonferroni correction to account for multiple testing, but we also report replication at only nominal level (one-sided p<0.05 but not surviving Bonferroni correction). The definition of CV traits in UKB, CKB and FinnGen, as well as the baseline characteristics of UKB and CKB, are reported in online supplemental tables S3 and S4, respectively.

## Results

### All colocalisation findings

Of the 55 genes, we found colocalisation (H4 or H3) with at least one CV trait for 22 genes, of which eight genes with FVC, 11 with FEV_1_/FVC, and three with both. The highest posterior probability for all other genes supported H1—association with the lung function trait only—reflecting the selection of the 55 genes.

Table 1 reports all identified colocalisation pairs, with colocalisation posterior probabilities. Table 2 summarises the function of the 22 genes and the biological category they belong to, defined as in our previous work^17^; 17 of the 22 are either transcriptional modifiers (six genes) or genes affecting cell:cell integrity, that is, cell-to-cell adhesion, cytoskeleton or extracellular matrix (ECM) genes (11 genes). Colocalisation was found predominantly with blood pressure traits for both lung function measures. For FVC, we also found colocalisation with CHD (*CSNK2B, ITGB5*, *MMP24* and *TNS1*) and CIMT (*MMP24*), while for FEV_1_/FVC we only found colocalisation with CHD in one gene (*TNS1*). We found no colocalisation with ASI for either lung function measure.

**Table 1:** Colocalisation results for lung function (FVC and FEV_1_/FVC) and CV traits

Gene	Lung function trait	CV traits	Posterior probability*	Previously reported association of the gene with any CV trait (PubMed ID)†
*ACTN4*	FVC	PP, DBP	1, 1	PP (34594039), HR (23583979)
*AGER*	FVC	DBP, PP	1, 1	SBP (27618447), CHD (29212778)
	FEV_1_/FVC	DBP, PP	1, 1	
***CLDN20***	FVC	**PP**	0.8	*None*
***CSNK2B* ‡**	FVC	**CHD**, **HTN**, **SBP**, DBP	1, 1, 1, 1	*None* §
	FEV_1_/FVC	HTN, SBP, **DBP**, **PP**	1, 1, 1, 1	
*CTNND1*	FEV_1_/FVC	HTN, SBP, PP	0.8, 1, 1	SBP (30595370), PP (34594039, 30578418)
*DSP*	FEV_1_/FVC	PP	0.8	SBP and DBP (34989438)
*ELN*	FEV1/FVC	PP	0.7	PP (33230300, 34594039, 30224653), SBP and DBP (34989438)
***FARP2***	FEV_1_/FVC	**HTN**, **PP**, **SBP**	0.9, 0.9, 0.9	SBP (30224653)
***GFI1* ¶**	FEV_1_/FVC	**HTN**	0.9	*None*
***IGF1***	FVC	**HTN**	0.8	SBP (30224653)
***ITGB5***	FVC	**CHD**	*	PP (27841878), SBP (27841878, 30578418, 30595370, 34594039), DBP (27841878, 34594039), MAP (34594039), CHD (28714975)
***KAT8***	FVC	**HTN**	0.7	*None*
*MAPRE1*	FEV_1_/FVC	**PP**	0.9	*None*
***MMP24***	FVC	**PP**, **CIMT**, CHD	1, 0.9, 1	CIMT (34852643)
***PPARD***	FEV_1_/FVC	HTN, **DBP**, SBP	0.7, 0.9, 0.8	SBP (30595370, 34594039), MAP (34594039)
***RUNX3***	FEV_1_/FVC	**DBP**	0.8	MAP (34594039)
***SERPING1***	FEV_1_/FVC	**PP**, **SBP**	0.7, 0.7	*None*
*TCF7L1*	FEV_1_/FVC	PP, SBP	1, 0.9	PP (27841878, 28135244, 27841878, 30578418, 30487518, 34594039)
*TGFB2*	FEV_1_/FVC	HTN, DBP	1, 1	SBP (30578418), DBP (30224653)
***TNS1***	FVC	**HTN**, **SBP**, **DBP**, **CHD**	1, 1, 1, 0.9	SBP (27841878, 30578418, 30595370, 34594039), DBP (27841878, 28135244, 34594039), MAP (34594039), CHD (29212778, 33020668), MI (33532862)
	FEV_1_/FVC	**SBP**, **DBP**, **CHD**	1, 1, 1	
*WNT2B*	FVC	HTN, PP, DBP, SBP	1, 1, 1, 1	HTN (30487518), SBP (27618452, 30578418, 29403010, 30487518, 30595370, 34594039, 29455858), DBP (27618452, 29403010, 30487518, 34594039, 29455858), MAP (29403010, 30487518, 34594039), PP (30487518, 34594039), CHD (30595370)
*WNT9A*	FVC	HTN, PP, DBP, SBP	1, 1, 1, 1	SBP (27841878, 34594039), DBP (27841878)

H4 results are highlighted in bold—colocalisation pairs for 12 genes where the variants for the two traits were either the same (H4), or, for the *GFI1* gene, distinct but in high LD (H4 equivalent).

* Highest posterior probability for H4 (in bold) or H3.

† Previous GWAS associations (p<5×10−8) with any CV trait in GWAS catalogue (www.ebi.ac.uk/gwas), accessed on 8 May 2024.

‡ Two different SNPs for FVC and FEV_1_/FVC (table 3).

§ SBP (27618447, 21909115; p=2.14×10−6 and p=2.2×10−6), PP (27618447; p=2.3×10−6) and CHD (29212778; p=1.2×10−6).

¶ Two different SNPs for HTN and FEV_1_/FVC, but in high LD, r2=0.85 (H4 equivalent).

CHD, coronary heart disease; CIMT, carotid intima-media thickness; CV, cardiovascular; FEV_1_/FVC, forced expiratory volume in 1 s to forced vital capacity ratio; GWAS, genome-wide association studies; HR, heart rate; HTN, hypertension; LD, linkage disequilibrium; MAP, mean arterial pressure; MI, myocardial infarction; PP, pulse pressure; SBP/DBP, systolic/diastolic blood pressure; SNP, single nucleotide polymorphism.

**Table 2:** Gene function and evidence of gene expression for the genes identified in the colocalisation analysis

Gene	Full name and function (biological category)	Evidence on tissue expression*		
Lung	Heart	Vasculature		
*ACTN4*	Actinin α 4: actin-binding protein, part of the cytoskeleton (*cell-to-cell adhesion and cytoskeleton*)	++	++	++
*AGER*	Advanced glycosylation end-product (AGE) specific receptor: multiligand receptor, also called RAGE; role in chronic vascular injury (*oxidative stress and endothelial dysfunction*)	+++	+	─
*CLDN20*	Claudin 20: encodes a tight junction protein; important for cell polarity and regulating movement of molecules via the paracellular route (*cell-to-cell adhesion and cytoskeleton*)	+	+	─
*CSNK2B*	Casein kinase 2β: ubiquitous protein kinase that regulates metabolic pathways, signal transduction, transcription, translation and replication (*transcriptional regulators*)	++	++	++
*CTNND1*	Catenin delta 1: armadillo protein family, which function in adhesion between cells and signal transduction (*cell-to-cell adhesion and cytoskeleton*)	+++	+++	++
*DSP*	Desmoplakin: encodes a protein component of functional desmosomes (*cell-to-cell adhesion and cytoskeleton*)	+++	+++	─
*ELN*	Elastin: encodes a protein that is one of the two components of elastic fibres (*extracellular matrix*)	+++	+++	─
*FARP2*	FERM, ARH/RhoGEF and pleckstrin domain protein 2: ρ guanidine exchange factor (*cell-to-cell adhesion and cytoskeleton*)	+++	+++	+
*GFI1*	Growth factor independent 1 transcriptional repressor: encodes a nuclear zinc-finger protein that functions as a transcriptional repressor (*transcriptional regulators*)	++	++	─
*IGF1*	Insulin-like growth factor 1: encodes an insulin-like protein involved in mediating growth and development (*growth factors*)	++	++	─
*ITGB5*	Integrin subunit β 5: encodes the integrin β subunit 5 protein (*extracellular matrix*)	+++	+++	+
*KAT8*	Lysine acetyltransferase 8: encodes a member of the MYST histone acetylase protein family; the encoded protein regulates gene transcription by influencing chromatin conformation (*transcriptional regulators*)	+++	+++	++
*MAPRE1*	Microtubule-associated protein RP/EB family member 1: encodes a protein that localises to microtubules, a dynamic network of filaments that form part of the cytoskeleton (*cell-to-cell adhesion and cytoskeleton*)	+++	+++	─
*MMP24*	Matrix metallopeptidase 24: encodes a member of the peptidase M10 family of MMPs (*extracellular matrix*)	++	+	─
*PPARD*	Peroxisome proliferator-activated receptor delta: encodes a member of the PPAR family that is believed to function as an integrator of transcriptional repression and nuclear receptor signalling (*transcriptional regulators*)	+++	++	+
*RUNX3*	Runt-related transcription factor 3: encodes for a member of the runt family of transcription factors that regulate haematopoiesis and skeletal development (*transcriptional regulators*)	+++	+++	─
*SERPING1*	Serpin family G member 1: encodes a highly glycosylated plasma protein involved in the regulation of the complement cascade. Mutations in *SERPING1* cause hereditary angioedema (*extracellular matrix*)	+++	+++	+
*TCF7L1*	Transcription factor 7-like 1: encodes a member of the T-cell factor/lymphoid enhancer factor family of transcription factors (*transcriptional regulators*)	++	++	+
*TGFB2*	Transforming growth factor β2: encodes a secreted ligand of the TGF-β superfamily of proteins (*growth factors*)	+++	+++	─
*TNS1*	Tensin 1: encodes for a protein that localises to focal adhesions and crosslinks actin filaments (*cell-to-cell adhesion and cytoskeleton*; *extracellular matrix*)	+++	+++	+
*WNT2B*	Wnt family member 2: member of the WNT gene family (*growth factors*)	+++	+++	+
*WNT9A*	Wnt family member 9A: member of the WNT gene family (*growth factors*)	+++	+++	─

* Evidence of gene expression (high: +++, medium: ++, low: +) from Expression Atlas (www.ebi.ac.uk/gxa/home) for lung and for cardiovascular tissues, in particular heart (heart left/right ventricle, left/right cardiac atrium, heart muscle) and vasculature (artery).

**Table 3:** Direction of the genetic effect on both lung function and CV trait for the 12 genes with H4 colocalisation (same signal)

FVC								
Gene	SNP (EA)*	FVC (mL)	PP (mm Hg)	SBP (mm Hg)	DBP (mm Hg)	HTN	CIMT (mm)	CHD
		Beta (*p value*)	Beta (*p value*)	Beta (*p value*)	Beta (*p value*)	OR (*p value*)	Beta (*p value*)	OR (*p value*)
*CLDN20*	rs1969863 (T)	−9.09 (*2.2×10^−9^*)	**0.17 (*6.6×10^−9^***)	−	−	−	−	−
*CSNK2B*	rs3117578 (G)	−17.75 (*8.2×10^−18^*)	−	**0.32 (*5.5×10^−9^*)**	−	**1.05 (*1.0×10^−15^*)**	−	**1.04 (*3.1×10^−5^*)**
*IGF1*	rs10745941 (T)	−9.58 (*4.2×10^−8^)*	−	−	−	**1.03 (*3.7×10^−6^*)**	−	−
*ITGB5*	rs17282078 (A)	−9.32 (*2.1×10^−5^*)	−	−	−	−	−	**1.06 (*6.2×10^−10^*)**
*KAT8*	rs1978487 (T)	−9.30 (*1.4×10^−9^*)	−	−	−	**1.02 (*5.4×10*^−5^)**	−	−
*MMP24*	rs6120880 (G)	−11.35 (*4.8×10^−14^*)	**0.16 (*1.3×10^−8^*)**	−	−	−	**0.01 (*5.0×10^−8^*)**	−
*TNS1†*	rs2571445 (A)	−11.46 (*3.3×10^−14^*)	−	**0.21 (*1.6×10^−7^*)**	**0.13 (*5.4×10^−9^*)**	**1.02 (*1.5×10^−5^*)**	−	**1.04 (*2.9×10*^−7^)**

FEV_1_/FVC							
Gene	SNP (EA)*	FEV_1_/FVC (%)	PP (mm Hg)	SBP (mm Hg)	DBP (mm Hg)	HTN	CHD
		Beta (*p value*)	Beta (*p value*)	Beta (*p value*)	Beta (*p value*)	OR (*p value*)	OR (*p value*)
*CSNK2B*	rs9267531 (G)	−0.30 (*5.6×10^−38^*)	**0.23 (*2.1×10^−8^*)**	−	−0.28 (*1.5×10^−17^*)	−	−
*FARP2*	rs139354822 (T)	−0.18 (*2.7×10^−4^*)	**0.47 (*1.0×10^−7^*)**	**0.68 (*2.3×10^−8^*)**	−	**1.10 (*2.8×10^−9^*)**	−
*GFI1*	rs6676846‡ (A)	−0.13 (*8.1×10^−12^*)	−	−	−	0.97 (2.7*×*10^−6^)	−
*PPARD*	rs2395623 (T)	−0.12 (*1.9×10^−10^*)	−	−	**0.14 (*1.0×10^−7^*)**		−
*RUNX3*	rs111283598 (G)	−0.21 (*6.6×10^−8^*)	−	−	−0.26 (*1.3×10^−6^*)	−	−
*SERPING1*	rs10896631 (C)	−0.13 (*1.4×10^−13^*)	−0.22 (2.4*×*10^−12^)	−0.25 (9.3×10^−09^)	−	−	−
*TNS1†*	rs2571445 (A)	−0.11 (*2.8×10^−12^*)	−	**0.21 (*1.6×10^−7^*)**	**0.13 (*5.4×10^−9^*)**	**1.02 (*1.5×10^−5^*)**	**1.04 (*2.9×10^−7^*)**

Results from linear/logistic regression analysis adjusted for age, sex, genotyping array, centre and 10 principal components, in addition to height for FVC. As the EA considered is the allele detrimental for lung function, the results for CV traits consistent with our hypothesis of lower lung function associated with higher CV risk are those with OR >1 (HTN and CHD) or beta >0 (continuous traits); these results are in bold.

* To simplify interpretation, results are presented by considering the EA as the allele, which is detrimental for lung function.

† Please note that for the TNS1 gene, the SNP is the same for FVC and FEV_1_/FVC, which explains why the results for SBP, DBP, HTN and CHD are identical in the final rows of the two tables.

‡ The best variants for HTN and FEV_1_/FVC are not the same but are in high LD, r2=0.85 (H4 equivalent): FEV_1_/FVC_rs6676141 and HTN_rs6676846; betas and p values refer to the HTN SNP, rs6676846, identified with fine mapping in coloc.

CHD, coronary heart disease; CIMT, carotid intima-media thickness; CV, cardiovascular; EA, effect allele; FEV_1_/FVC, forced expiratory volume in 1 s to forced vital capacity ratio; HR, heart rate; HTN, hypertension; LD, linkage disequilibrium; PP, pulse pressure; SBP/DBP, systolic/diastolic blood pressure; SNP, single nucleotide polymorphism.

For the 22 genes with colocalisation, we investigated gene expression in both human lung and CV tissues, in particular, heart (heart left/right ventricle, left/right cardiac atrium, heart muscle) and vasculature (artery), using the ‘Expression Atlas’ database (www.ebi.ac.uk/gxa/home). All 22 genes are expressed in both human lung and heart, with 11 being also expressed in the vasculature (table 2).

### H4 colocalisation findings

Of main interest for our study are the 13 signals in 12 genes, where the same SNP is shared between the lung function and CV traits, for which we could investigate the direction of the two genetic effects (regression analysis), and for which we sought replication. Of these, 11 shared the same variant (H4) and one colocalised at two variants in high LD (H4 equivalent; see table 1). For FVC, all colocalisation pairs (seven genes) showed directions consistent with the hypothesis that a variant which reduces lung function also has a detrimental effect on the CV trait (higher risk of disease or higher continuous measure) (table 3). For FEV_1_/FVC, among the colocalisation pairs (seven genes), the direction of the effect was consistent with our hypothesis in four genes (table 3).

The sensitivity analysis for FEV_1_/FVC excluding participants with asthma confirmed the findings of the main analysis (data not shown).

In FinnGen, all seven colocalisation signals for FVC or FEV_1_/FVC with either HTN or CHD (available phenotypes) showed replication of the CV association at nominal level (p<0.05), with five of them surviving Bonferroni correction for multiple testing; three SNPs also showed associations with myocardial infarction (table 4a).

**Table 4:** Replication of H4 colocalisation signals for CV traits

(a) FinnGen (only data on HTN and CHD were available)								
Gene	SNP	LF trait	CV trait	EA	EAF UKB/FinnGen	UKB	FinnGen	Association with other CV traits
						OR (*p value*)	OR (*p value; one-sided p value*)*	
***CSNK2B***	rs3117578	FVC	**HTN**	G	0.85/0.86	1.05 (*1.0×10^−15^*)	1.04 *(9.7×10^−8^;* ***4.8×10^−8^***)*	**MI** (1.05; *p=6.6×10*^*−4*^)
			**CHD**			1.04 (*3.1×10^−5^*)	1.06 (2.9×10^−6^; **1.4×10^−6^**)*	
***FARP2***	rs139354822	FEV_1_/FVC	**HTN**	T	0.97/0.95	1.10 (*2.8×10^−9^*)	1.06 (*5.3×10^−5^;* ***2.6×10^−5^***)*	
***GFI1***	rs6676846	FEV_1_/FVC	**HTN**	A	0.79/0.78	0.97 (2.7*×10*^−6^)	0.98 (*1.7×10^−3^;* ***8.3×10^−4^***)*	
***IGF1* †**	rs10745941	FVC	**HTN**	T	0.76/0.77	1.03 (*3.7×10^−6^*)	1.02 *(0.02;* ***9.3×10^−3^****)*	
***ITGB5***	rs17282078	FVC	**CHD**	A	0.13/0.10	1.06 (*6.2×10^−10^*)	1.03 *(0.04;* ***0.02****)*	**MI** (1.04; *p=0.03*)
***KAT8***	rs1978487	FVC	**HTN**	T	0.64/0.62	1.02 (5.4*×10*^−5^)	1.01 (*0.06;* ***0.03***)	
***TNS1***	rs2571445	FVC, FEV_1_/FVC	**HTN**	A	0.39/0.44	1.02 (*1.5×10^−5^*)	1.01 *(0.02;* ***8.4×10^−3^****)*	**MI** (1.04; *p=1.3×10*^*−4*^)
			**CHD**			1.04 (2.9*×10*^−7^)	1.04 *(3.7×10^−7^;* ***1.9×10^−7^***)*	

(b) CKB (data on all phenotypes were available)								
Gene	SNP	LF trait	CV trait	EA	EAF UKB/CKB	UKB	CKB	Association with other CV traits
						Beta/OR (*p value*)	Beta/OR (*p value; one-sided p value*)*	
***CLDN20***	rs1969863	FVC	**PP**	T	0.62/0.98	0.17 (*6.6×10^−9^*)	0.37 (*0.11; **0.05***)	
*CSNK2B*	rs3117578	FVC	SBP	G	0.85/0.98	0.32 (*5.5×10^−9^*)	−0.47 (*p*=*0.17*)	
			HTN			1.05 (*1.0×10^−15^*)	1.01 (*0.78; 0.39*)	
			CHD			1.04 (*3.1×10^−5^*)	1.01 (*0.87; 0.44*)	
*GFI1*	rs6676846	FEV_1_/FVC	HTN	A	0.79/0.96	0.97 (2.7*×10*^−6^)	0.99 (*0.70; 0.35*)	
*IGF1*	rs10745941	FVC	HTN	T	0.76/0.84	1.03 (*3.7×10^−6^*)	1.01 (*0.75; 0.37*)	
*ITGB5*	rs17282078	FVC	CHD	A	0.13/0.08	1.06 (*6.2×10^−10^*)	1.01 (*0.72; 0.36*)	
*KAT8*	rs1978487	FVC	HTN	T	0.64/0.09	1.02 (5.4*×10*^−5^)	1.02 (*0.25; 0.13*)	**SBP** (0.50; *p=3.3*^*−3*^) **DBP** (0.32, *p=7.9*^*−4*^)
*MMP24*	rs6120880	FVC	PP	G	0.42/0.35	0.16 (*1.3×10^−8^*)	0.10 (*0.14; 0.07*)	
			CIMT			0.01 (*5.0×10^−8^*)	0.01 (*0.19; 0.09*)	
*PPARD*	rs2395623	FEV_1_/FVC	DBP	T	0.23/0.29	0.14 (*1.0×10^−7^*)	−0.12 (*p*=*0.04)*	
*RUNX3*	rs111283598	FEV_1_/FVC	DBP	G	0.96/0.91	−0.26 (*1.3×10^−6^*)	−0.09 (*0.38; 0.19*)	
***SERPING1***	rs10896631	FEV_1_/FVC	**PP**	C	0.27/0.14	−0.22 (*2.4×10^−12^*)	−0.16 (*0.08; **0.04***)	
			SBP			−0.25 (*9.3×10^−9^*)	−0.16 (*0.24; 0.12*)	*0.24* (*0.12*)
***TNS1***	rs2571445	FVC, FEV_1_/FVC	**SBP**	A	0.39/0.40	0.21	0.21 (*0.03; **0.02***)	*0.03* (***0.02***)
			**DBP**			0.13	0.12 (*0.03; **0.01***)	*0.03* (***0.01***)
			HTN			1.02	0.99 (*p*=*0.32)*	*0.32*
			CHD			1.04	1.02 (*0.25; 0.12*)	*0.25* (*0.12*)

In bold are results replicated at least at nominal level (same effect direction and one-sided p<0.05), with an asterisk indicating replication at Bonferroni correction (FinnGen: p<5.6×10⁻3; 0.05/9 tests; CKB: p<2.8×10⁻3; 0.05/18 tests). Statistically significant association with other CV traits are also reported with OR (binary variables) or beta (continuous variables) and p values (two-sided).

EA: allele associated with decreased lung function, as shown in table 3.

* One-sided p values calculated only for effects in same direction as in UKB.

† A proxy was used, rs5742694 (r2=0.91), as rs10745941 was not available in FinnGen.

CHD, coronary heart disease; CKB, China Kadoorie Biobank; CV, cardiovascular; EA, effect allele; EAF, effect allele frequency; FEV_1_, forced expiratory volume in 1 s; FVC, forced vital capacity; HTN, hypertension; LF, lung function; SBP/DBP, systolic/diastolic blood pressure; UKB, UK Biobank.

In CKB, data were available for 11 of the 13 SNPs. Only three replicated at nominal significance, none surviving Bonferroni correction; for another signal, replication was observed for SBP and DBP, although not for the original HTN. Replication power was limited in this Chinese population by low minor allele frequency of six of the 11 SNPs (table 4b).

### Novelty of our colocalisation findings for CV traits

To assess whether any of the 22 genes identified by our overall colocalisation analysis were novel for CV traits, we searched for previous GWAS findings using the GWAS catalogue (www.ebi.ac.uk/gwas) (table 1). Six of the 22 genes (*CLDN20*, *CSNK2B*, *GFI1*, *KAT8*, *MAPRE1* and *SERPING1*) have not been associated with any CV trait in previous GWAS, although for *CSNK2B* there was some evidence of association with CHD and blood pressure failing to reach genome-wide significance. For all six novel genes, we found evidence supporting a genuine association from animal studies, cell/in vitro studies or previous candidate-gene studies (online supplemental table S5).

In FinnGen, we observed associations with CV traits for four of the six genes: *CSNK2B* (HTN, CHD, myocardial infarction), *KAT8* (HTN) and *GFI1* (HTN) (table 4a); and *CLDN20* (HTN) (online supplemental table S6). In CKB, there was some evidence of association only for *CLDN20* (PP), *SERPING1* (PP) and *KAT8* (SBP, DBP) (table 4b).

## Discussion

The findings of this study support the hypothesis that signalling pathways critical to lung development and repair, and hence lung ageing, are also important for the development and/or repair of the CV system. We found colocalisation predominantly with blood pressure traits, and we speculate that this may reflect pathways required to drive vascular development in the lungs, heart and blood vessels. In the lungs, reciprocal interactions between the airways and blood vessels are essential to drive the development of both the airways and the alveoli.^25 26^ Our findings for blood pressure traits may reflect impaired vascular development, resulting in either fewer vessels/capillaries or reduced integrity of them, and impaired capacity for vascular growth may underlie essential HTN.^27^ Particularly for FVC, we also found colocalisation with CHD (*CSNK2B, ITGB5*, *MMP24* and *TNS1*) and CIMT (*MMP24*). A lack of colocalisation with ASI may reflect limited statistical power, as this phenotype was measured in a smaller subsample of UKB participants. In fact, while we were able to detect a signal for CIMT in an even smaller subsample, the power of the ASI analysis is likely to be further reduced by the measurement error and variability associated with finger photo-plethysmography measurements, compared with measurements of aortic stiffness from tonometry/Doppler techniques (pulse wave velocity) or CV MRIs.^28^

Importantly, when focusing on colocalisation defined in the strictest form, that is, sharing of the same signal within a gene (H4), all results for FVC showed directions of the genetic effects consistent with our hypothesis that a variant that decreases lung function will have a detrimental effect on the CV trait. In contrast, this was not always the case for FEV_1_/FVC. However, this is perhaps not surprising, given that the epidemiological evidence for a link between lower lung function and increased CV risk is stronger for a restrictive defect (as indicated by a lower FVC) than for an obstructive defect (as measured by lower FEV_1_/FVC).^1 2 6 9^ Associations with CV traits identified by our H4 colocalisation findings were replicated in FinnGen, although not all survived correction for multiple testing. On the contrary, replication was poor in CKB. Limited statistical power is a possible explanation for this: the sample size in CKB was smaller than in FinnGen and much smaller than in UKB, and the minor allele frequency for many SNPs was lower in this Chinese population. Reduced replication ability in CKB might also be due to ethnic differences in LD patterns; a marker SNP identified in Europeans as in LD with the underlying causal variant may not be in LD with the causal variant in a Chinese population.^29^

Our findings show that the magnitude of the effects of the individual genetic variants is small, as is typically the case in genetic association studies, and therefore these shared genetic signals are likely to explain only a modest part of the observational relationship between low lung function and CV disease. The importance of our findings rather rests on the demonstration of shared developmental pathways, with the identification of specific genes and variants that are worth further investigation.

### Biological interpretation

One of our strongest colocalisation signals was in *CSNK2B*. This gene encodes for the beta subunit of casein kinase II, a protein known to mediate diverse cellular pathways (online supplemental table S5); one of these is Wnt signalling, which plays a key role in lung and CV development (online supplemental table S5). Notably, three other genes with colocalisation are also involved in Wnt signalling: *WNT2B*, *WNT9A* and *TCF7L1*. The proteins encoded by *MMP24* and *TNS1*, two of our strongest colocalisation findings, affect the ECM, and ECM remodelling is a cause of both vascular and respiratory diseases.^30^ Regulation of ECM is tightly controlled by balancing maintenance of its components, such as collagen and elastin (coded by *ELN*, also showing colocalisation), with their degradation by matrix metalloproteases (MMPs). MMPs influence vascular ECM remodelling, and *MMP24* (with other MMP genes) has been recently linked to intracranial aneurysms (online supplemental table S5). Our colocalisation finding for the *ELN* gene (FEV_1_/FVC and PP) is of interest because elastin is essential for elastic recoil, both in the lung and in the walls of the aorta and large arteries. As elastin is only produced early in life and is difficult to repair later in life,^31^ optimal deposition of elastin in these tissues during development is crucial. Impaired elastin synthesis in early life could increase the risk of an emphysematous form of airflow obstruction,^32^ and also reduced compliance (ie, increased stiffness) of the aorta and large arteries, with widening of PP.^33^

Another gene showing colocalisation was *CLDN20*, which encodes the tight junction protein CLDN20 (claudin 20). Tight junctions regulate paracellular permeability, and their dysfunction disrupts the tissue barrier leading to oedema. While other claudins are known to influence the regulation of blood pressure (online supplemental table S5), the specific role of CLDN20 has barely been investigated despite its expression in the heart, lungs and vasculature; however, CLDN20 has been recently shown to be significantly decreased after ischaemia in a multi-omics mouse study of ischaemic stroke (online supplemental table S5). Among the other genes identified, *DSP* encodes desmoplakin, which is involved in cell:cell interactions and, like claudins, is important for tissue integrity. In a zebrafish model, the knockout of *DSP* has been shown to result in loss of cardiac Wnt signalling.^34^ We also found colocalisation for *MAPRE1*, which encodes a microtubule-associated protein. Microtubules are part of the cytoskeletal network that maintains cell tension and integrity. In particular, *MAPRE1* has been shown to play a role in cardiomyocyte integrity and adaptation to pressure overload, and in cardiac conduction and arrhythmias (online supplemental table S5).

Finally, we looked for evidence of the association of the proteins coded by the identified genes with both lung function and CV traits. We did so by interrogating the publicly available Proteome PheWAS Browser (www.epigraphdb.org/pqtl/), which leverages protein quantitative trait loci data from published proteomic studies and Mendelian Randomisation analysis to investigate the causal effects of multiple circulating proteins on multiple phenotypes.

For the IGF1 gene, the colocalisation for FVC with HTN is supported by the association of the IGF1 protein with DBP and SBP, in addition to association with lung function and disease traits. Similarly for SERPING1, one of the six genes with no previous GWAS association with CV traits, our colocalisation findings for FEV1/FVC with SBP and PP are supported by association of the SERPING1 protein with SBP, in addition to association with lung function. With the continuously accumulating genome-wide proteomic studies, similar evidence for further genes identified in our colocalisation analysis is likely to become available in the near future.

### Strengths and limitations

Our study has a number of strengths. First, the large sample size provided by UKB, complemented by publicly available data from CARDIoGRAM, meant we had considerable statistical power to analyse blood pressure and CHD phenotypes. Second, our findings provide stronger evidence for the importance of the shared developmental origins of adult lung function and CV disease than could be achieved in observational studies, which are prone to confounding. Third, six of the 22 genes found in our colocalisation analysis (*CLDN20*, *CSNK2B*, *GFI1*, *KAT8*, *MAPRE1*, *SERPING1*) have not been associated with any CV trait in previous GWASs, and yet, for all of them, we found evidence from laboratory studies and candidate-gene studies supporting a genuine association. This gives added value to our study and illustrates the potential of hypothesis-driven investigations to complement a GWAS approach for the identification of novel candidate genes.

Our study also has some limitations. Some of the genes that we investigated have a role in lung development, and later in life, in adult lung homeostasis and repair through alveolar maintenance and regeneration after injury. In fact, we expect the majority of the 22 colocalised genes identified in this study will play a role in repair in addition to their developmental role. There is already published evidence of such a role for some of the genes, including *RUNX3*,^35^ *IGF1*^36^ and *SERPING1*.^37^ For others, such as *FARP2* and *CLDN20*, there is not yet published evidence for a role in lung repair, but this is probably just because these specific studies have not been done yet. *FARP2* regulates the actin cytoskeleton, and this fundamental cellular process is critical for repair. Likewise, *CLDN20* is a tight junction protein that helps cells stick together, so it will also be important for repair. Our results highlight the importance of investigating the role of these genes in repair in the near future.

Yet, given the age at which lung function and CV phenotypes were measured cross-sectionally in UKB participants, we cannot say whether our findings predominantly reflect effects on development or repair. While distinguishing between the two aspects is important, data from longitudinal cohorts covering the life course are needed to address this. It would be interesting to investigate the effects of lung development genes on lung function and CV phenotypes in younger cohorts, before lung function decline has occurred; a stronger genetic signal in children and young adults would suggest that the effect seen in older adults in UKB predominantly reflects an effect on lung and CV development, rather than repair in later life. On the contrary, a stronger effect in older adults following lung function decline would point to an important role in maintenance and regeneration. Indeed, indirect evidence that later repair might be relevant to our FVC-HTN findings comes from the CARDIA (coronary artery risk development in young adults) study, in which faster decline in FVC in young adults was associated with incident HTN, after controlling for smoking and other confounders.^38^ Another limitation is that UKB only measured prebronchodilator lung function, thus a low prebronchodilator FEV_1_/FVC cannot exclude reversible obstruction, the hallmark of asthma; however, a sensitivity analysis for FEV_1_/FVC excluding individuals with a diagnosis of asthma showed similar findings.

Finally, the UKB study population is not fully representative of the general UK population. Participants tend to be healthier, of higher socioeconomic status and predominantly of white European ancestry compared with the UK population as a whole.^39^ Due to the voluntary recruitment and low response rate in UKB, there is also a risk of healthy volunteer bias, which can lead to systematic differences between the study population and the overall UK population.^39^ However, while this has been shown to distort genetic associations with behaviour, lifestyles and social outcomes, a limited impact has been observed for genetic associations with physical traits.^40^ A more general limitation of genetic association studies related to the characteristics of a given study population is the potential bias and limited generalisability of findings due to gene-environment interactions, if the interacting environmental factors vary across populations and population subgroups. Future studies in different populations and ethnic groups will be important to establish the generalisability of our findings.

## Conclusion

In conclusion, our results provide evidence that adult lung function and CV comorbidities have shared developmental signalling pathways. This may partly explain why lower lung function, especially FVC, is associated with increased CV risk. Our findings may inform future research on druggable targets aimed at optimising lung and CV development and/or repair, and preventing premature morbidity and mortality. Of note, our work has also identified potentially novel CV genes.

## Supplementary material

10.1136/thorax-2024-222474 online supplemental file 1

10.1136/thorax-2024-222474 online supplemental file 2

10.1136/thorax-2024-222474 visual abstract

## Data Availability

Data are available on reasonable request.
