# Supplementary material for: Lung development genes, adult lung function and cardiovascular comorbidities
Source: Thorax. 2025 May 30;80(10):e222474. doi: 10.1136/thorax-2024-222474 (PMC12505039; doi:10.1136/thorax-2024-222474)
Supplement: online supplemental file 1 [file thorax-80-10-s001.pdf]

Online Data Supplement

Lung development genes, adult lung function and cardiovascular comorbidities

Laura Portas PhD, Mohammad Talaei PhD, Charlotte Dean PhD, Nay Aung PhD, Matthew Hind MD, Alfred Pozarickij PhD, Robin Walters PhD, China Kadoorie Biobank Collaborative Group, Peter GJ Burney MD, Steffen E. Petersen DPhil, Cosetta Minelli PhD, Seif O. Shaheen PhD

Members of the *China Kadoorie Biobank* collaborative group:

International Steering Committee: Junshi Chen, Zhengming Chen (PI), Robert Clarke, Rory Collins, Liming Li (PI), Jun Lv, Richard Peto, Robin Walters.

International Co-ordinating Centre, Oxford: Daniel Avery, Maxim Barnard, Derrick Bennett, Ruth Boxall, Ka Hung Chan, Yiping Chen, Zhengming Chen, Charlotte Clarke, Johnathan Clarke; Robert Clarke, Huaidong Du, Geoffrey Ma, Ahmed Edris Mohamed, Hannah Fry, Simon Gilbert, Pek Kei Im, Andri Iona, Maria Kakkoura, Christiana Kartsonaki, Kshitij Kolhe, Hubert Lam, Kuang Lin, James Liu, Mohsen Mazidi, Iona Millwood, Sam Morris, Qunhua Nie, Alfred Pozarickij, Maryam Rahmati, Paul Ryder, Dan Schmidt, Becky Stevens, Iain Turnbull, Robin Walters, Baihan Wang, Lin Wang, Neil Wright, Ling Yang, Xiaoming Yang, Pang Yao.

National Co-ordinating Centre, Beijing: Xiao Han, Can Hou, Qingmei Xia, Chao Liu, Jun Lv, Pei Pei, Dianjianyi Sun, Canqing Yu, Lang Pan.

Regional Co-ordinating Centres:

Qingdao CDC: Zengchang Pang, Ruqin Gao, Shanpeng Li, Haiping Duan, Shaojie Wang, Yongmei Liu, Ranran Du, Yajing Zang, Liang Cheng, Xiaocao Tian, Hua Zhang, Yaoming Zhai, Feng Ning, Xiaohui Sun, Feifei Li. Licang CDC: Silu Lv, Junzheng Wang, Wei Hou. Heilongjiang Provincial CDC: Wei Sun, Shichun Yan, Xiaoming Cui. Nangang CDC: Chi Wang, Zhenyuan Wu,Yanjie Li, Quan Kang. Hainan Provincial CDC: Huiming Luo, Tingting Ou. Meilan CDC: Xiangyang Zheng, Zhendong Guo, Shukuan Wu, Yilei Li, Huimei Li. Jiangsu Provincial CDC: Ming Wu, Yonglin Zhou, Jinyi Zhou, Ran Tao, Jie Yang, Jian Su. Suzhou CDC: Fang Liu, Jun Zhang, Yihe Hu, Yan Lu, Liangcai Ma, Aiyu Tang, Shuo Zhang, Jianrong Jin, Jingchao Liu. Guangxi Provincial CDC: Mei Lin, Zhenzhen Lu. Liuzhou CDC: Lifang Zhou, Changping Xie, Jian Lan,Tingping Zhu,Yun Liu, Liuping Wei, Liyuan Zhou, Ningyu Chen, Yulu Qin, Sisi Wang. Sichuan Provincial CDC: Xianping Wu, Ningmei Zhang, Xiaofang Chen, Xiaoyu Chang. Pengzhou CDC: Mingqiang Yuan, Xia Wu, Xiaofang Chen, Wei Jiang, Jiaqiu Liu, Qiang Sun. Gansu Provincial CDC: Faqing Chen, Xiaolan Ren, Caixia Dong. Maiji CDC: Hui Zhang, Enke Mao, Xiaoping Wang, Tao Wang, Xi zhang. Henan Provincial CDC: Kai Kang, Shixian Feng, Huizi Tian, Lei Fan. Huixian CDC: XiaoLin Li, Huarong Sun, Pan He, Xukui Zhang. Zhejiang Provincial CDC: Min Yu, Ruying Hu, Hao Wang. Tongxiang CDC: Xiaoyi Zhang, Yuan Cao, Kaixu Xie, Lingli Chen, Dun Shen. Hunan Provincial CDC: Xiaojun Li, Donghui Jin, Li Yin, Huilin Liu, Zhongxi Fu. Liuyang CDC: Xin Xu, Hao Zhang, Jianwei Chen,Yuan Peng, Libo Zhang, Chan Qu.

FinnGen study

The FinnGen study is a large-scale genomics initiative that has analyzed over 500,000 Finnish biobank samples and correlated genetic variation with health data to understand disease mechanisms and predispositions. The project is a collaboration between research organisations and biobanks within Finland and international industry partners.

Data

UKB data

UKB is a study of 502,543 volunteer participants aged 40-69, recruited from 22 study centres across the United Kingdom, which collected data on a large number of genetic and non-genetic risk factors for chronic disease and related disease traits. We included in our study UKB participants of self-reported white ethnicity and with good quality lung function data ("best measure"). Supplementary Table 1 provides UKB data field numbers and web links for full descriptions of all variables used in the analyses.

The traits analysed were:

- Lung function, including FVC and Forced Expiratory Volume in one second (FEV_1)/FVC (N=306,476). Spirometry was performed without bronchodilator administration, so only "pre-bronchodilator" lung function data are available.
- Blood pressure traits (N=369,905). These included:
    - Systolic blood pressure (SBP) and diastolic blood pressure (DBP), calculated from two measurements. In individuals on antihypertensive treatment, 15 mmHg and 10 mmHg were added to SBP and DBP, respectively, as previously suggested[1].
    - Hypertension (HTN), defined by a SBP ≥140 mmHg or a DBP ≥90 mmHg or taking blood pressure lowering medication.
- Pulse pressure (PP), calculated as the difference between SBP and DBP.
- Pulse wave arterial stiffness index (ASI), a non-invasive measure of arterial stiffness, was measured using finger photo-plethysmography (N=55,041). The raw ASI variable was cleaned by excluding individuals with an absent notch position in the pulse waveform and outlier ASI values which were considered implausible, i.e. those which were three inter-quartile ranges below the first quartile or above the third quartile.

- CIMT, a measure of subclinical atherosclerosis was measured using ultrasound (N=38,469). The mean of four maximum values (in mm) was used, two from the left carotid artery, and two from the right. Measurements were log-transformed.
- CHD was defined as a self-reported heart attack (myocardial infarction) or angina; coronary artery bypass graft surgery, triple heart bypass or coronary angioplasty (with or without a stent) (N=405,570, including N=19,294 cases).

Colocalization analysis

Pairwise colocalization analysis, as implemented in the R package coloc[2], allows investigation of whether two traits share the same genetic signals in specific genomic regions, which in our study correspond to specific genes. We used coloc with summary data to investigate whether any of the 55 lung development genes we previously identified as associated with adult lung function[3, 4] also have an effect on cardiovascular (CV) traits.

Colocalization analysis in coloc is performed using a Bayesian framework; while there is no need to correct for multiple testing, prior beliefs about SNP-specific associations under each scenario must be specified[2, 5]. The choice of priors is described below. The Bayesian approach provides an estimate of the posterior probability that a genetic variant is shared between the traits studied, based on probabilistic models that incorporate the prior beliefs and the observed data[2].

Choice of priors

In this Bayesian framework for the colocalization analysis, results for each genomic region (gene) are expressed in terms of a posterior probability of association under each of 5 scenarios: H0, H1, H2, H3 and H4. These scenarios are defined as follows:

- **H0** = No association with either trait in region
- **H1** = Association with trait 1 in region, but not trait 2
- **H2** = Association with trait 2 in region, but not trait 1
- **H3** = Association with both traits in region, but separate causal variants
- **H4** = Association with both traits in region, same causal variants

where ***trait 1*** is the **lung function trait**, and ***trait 2*** is the **CV trait**.

As with any Bayesian method, prior beliefs about the 5 scenarios need to be specified before running the analysis. This can be done by specifying three prior probabilities for each SNP in a given gene:

- **p12** = Prior probability a random SNP in the region is jointly causal for both traits
- **p1** = Prior probability a random SNP in the region is causally associated with trait 1 and not trait 2
- **p2** = Prior probability a random SNP in the region is causally associated with trait 2 and not trait 1

Implemented in coloc are default prior distributions for these probabilities that reflect the use of colocalization analysis in a hypothesis-free genome-wide approach. For our hypothesis-driven colocalization analysis focused on 55 lung development genes, we therefore modified these default priors to reflect our *a priori* knowledge of association of these genes with adult lung function (FVC and/or FEV_1/FVC). While this prior knowledge is directly derived from our previous study based on the same UKB data used here, thus violating the basic requirement for prior information having to come from external sources, association with lung function had been previously found in other datasets for 19 of the 55 genes, and we could replicate most of the other 36 novel genes in external datasets, CHARGE[4] and SpiroMeta[6].

For each gene, we specified SNP-specific prior probabilities as follows, with further details in the Stata code reported at the end of this section:

- **p1** (referring to trait 1 = LF trait) of 0.85/n_SPNs, where n_SNPs is the number of SNPs within the gene. This means an overall (cumulative) probability that the gene is associated with the LF trait, but not with the CV trait, of 0.85;

- **p2** and **p1|2** were derived as described below (see *STATA code for deriving p2 and p12*) in order to obtain (approximately) the following prior probabilities about the 5 scenarios:

o **H0**: 0
o **H1**: 0.85
o **H2**: 0.05 (slightly more - 0.053)
o **H3**: 0.05 (slightly less - 0.045)
o **H4**: 0.05 (slightly more - 0.053)

This setting of prior probabilities can be interpreted as an *a priori* hypothesis that 85% of our 55 genes are only associated with the LF trait (H1), while we would expect 15% of them (8 genes) to be associated with either only the CV trait (H2), both traits at different variants (H3), or both

traits at the same variant (H4) – each with a probability of 5%. The probability of no association with either trait (H0) is 0.

Using the *Prior explorer for coloc* tool developed by the same group who developed the coloc method, Chris Wallace et al ((https://chr1swallace.shinyapps.io/coloc-priors/), we show below a graphical illustration of our prior beliefs reflected by this choice of priors for 3 examples of genes with different numbers of SNPs: *ACTN4* (232 SNPs), *ACTN3* (24 SNPs), and *GJE1* (2 SNPs). It's only in the extreme example with 2 SNPs that the H0 to H4 probabilities are not exactly what we aimed at – with H0 being slightly higher than 0 (2.2%). In our 55 genes, the number of SNPs varies from 2 to 2,762.

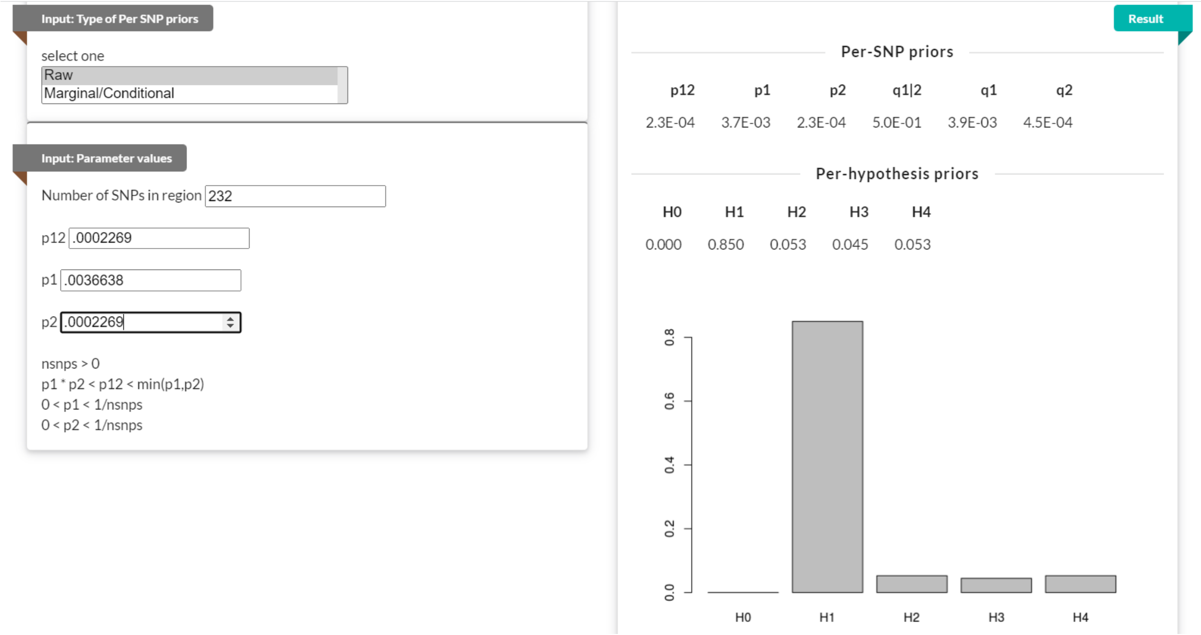
Input: Type of Per SNP priors
select one
Raw
Marginal/Conditional
Input: Parameter values
Number of SNPs in region 232
p12 .0002269
p1 .0036638
p2 .0002269
nsnps > 0
p1 * p2 < p12 < min(p1,p2)
0 < p1 < 1/nsnps
0 < p2 < 1/nsnps
Result
Per-SNP priors
p12 p1 p2 q1|2 q1 q2
2.3E-04 3.7E-03 2.3E-04 5.0E-01 3.9E-03 4.5E-04
Per-hypothesis priors
H0 H1 H2 H3 H4
0.000 0.850 0.053 0.045 0.053
0.0 0.2 0.4 0.6 0.8
H0 H1 H2 H3 H4

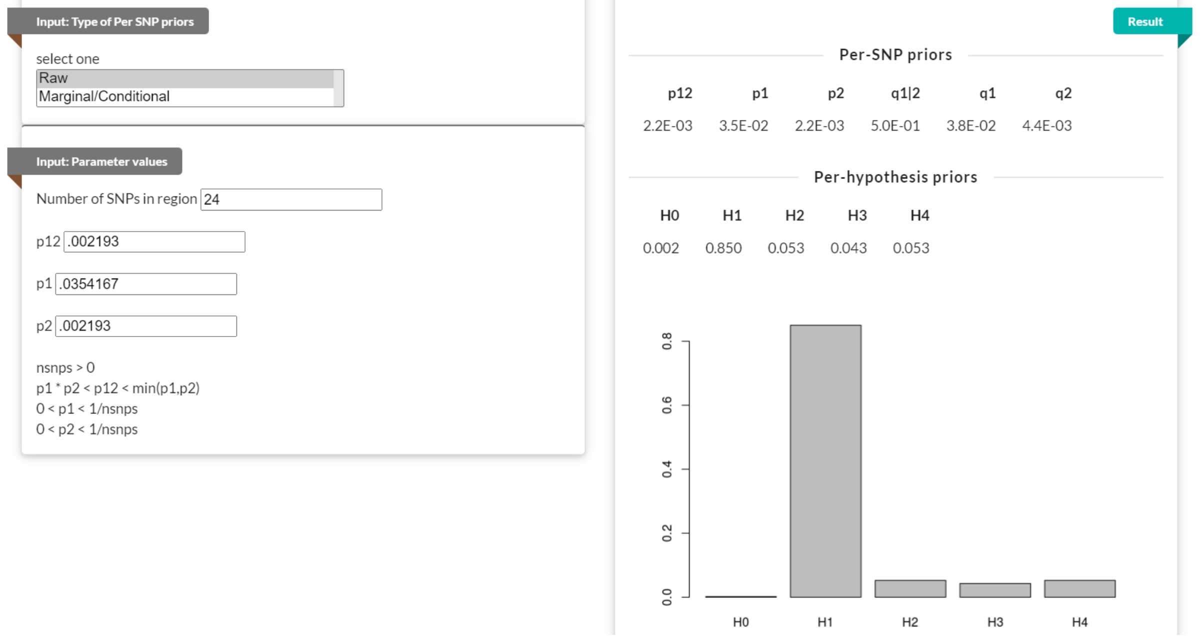
Input: Type of Per SNP priors
select one
Raw
Marginal/Conditional
Input: Parameter values
Number of SNPs in region 24
p12 .002193
p1 .0354167
p2 .002193
nsnps > 0
p1 * p2 < p12 < min(p1,p2)
0 < p1 < 1/nsnps
0 < p2 < 1/nsnps
Result
Per-SNP priors
p12 p1 p2 q1|2 q1 q2
2.2E-03 3.5E-02 2.2E-03 5.0E-01 3.8E-02 4.4E-03
Per-hypothesis priors
H0 H1 H2 H3 H4
0.002 0.850 0.053 0.043 0.053
0.0 0.2 0.4 0.6 0.8
H0 H1 H2 H3 H4

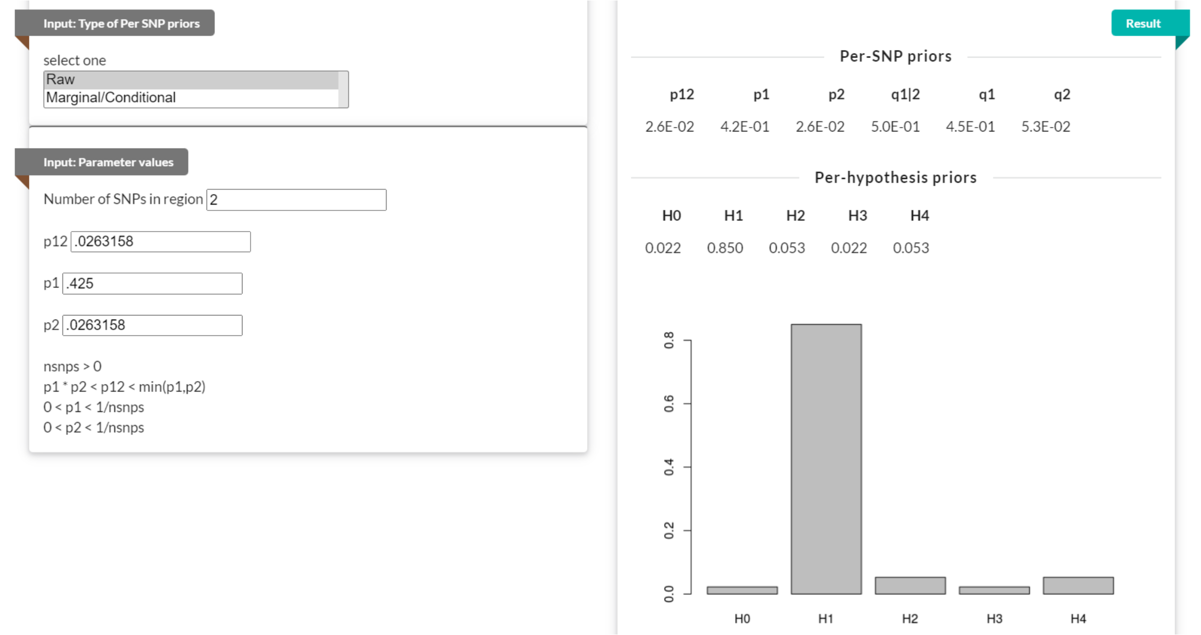
Input: Type of Per SNP priors
select one
Raw
Marginal/Conditional
Input: Parameter values
Number of SNPs in region 2
p12 .0263158
p1 .425
p2 .0263158
nsnps > 0
p1 * p2 < p12 < min(p1,p2)
0 < p1 < 1/nsnps
0 < p2 < 1/nsnps
Result
Per-SNP priors
p12 p1 p2 q1|2 q1 q2
2.6E-02 4.2E-01 2.6E-02 5.0E-01 4.5E-01 5.3E-02
Per-hypothesis priors
H0 H1 H2 H3 H4
0.022 0.850 0.053 0.022 0.053
0.0 0.2 0.4 0.6 0.8
H0 H1 H2 H3 H4

STATA code for deriving p2 and p12

Input required: number of SNPs (snps) per gene. Value for p1 (cumulatively for the whole gene) fixed at 0.85.

```
** Overall (gene-level) prior probabilities
gen p1_g = 0.85
gen p2_g = (1-p1_g)/(2+p1_g)
gen p12_g = p2_g
gen sum_p_g = (p1_g + 2*p2_g + p12_g)
list n_snps p1_g p2_g p12_g sum_p_g

** SNP-level prior probabilities
gen p1 = p1_g/n_snps
	gen p2 = p2_g/n_snps
	gen p12 = p12_g/n_snps
gen sum_p = (p1 + 2*p2 + p12)*n_snps
list n_snps p1 p2 p12 sum_p
```

Table S1: UK Biobank Data fields. For each variable used in the analyses, the UK Biobank Data field is presented with the related web link for a full description of the variable.

Variable	UKB Data field; Related web link
FVC (*best measure*) *	20151; https://biobank.ctsu.ox.ac.uk/crystal/field.cgi?id=20151
FEV_1 (*best measure*) *	20150; https://biobank.ctsu.ox.ac.uk/crystal/field.cgi?id=20150
Age	21022; https://biobank.ctsu.ox.ac.uk/crystal/field.cgi?id=21022
Sex	31; https://biobank.ctsu.ox.ac.uk/crystal/field.cgi?id=31
Height	50; https://biobank.ctsu.ox.ac.uk/crystal/field.cgi?id=50
Genotyping array	22000; https://biobank.ctsu.ox.ac.uk/crystal/field.cgi?id=22000
Assessment centre	54; https://biobank.ctsu.ox.ac.uk/crystal/field.cgi?id=54
Self-reported ethnicity	21000; https://biobank.ctsu.ox.ac.uk/crystal/field.cgi?id=21000
Blood pressure traits	2966; https://biobank.ndph.ox.ac.uk/showcase/field.cgi?id=2966
	6150 (*high blood pressure* code: 4); https://biobank.ndph.ox.ac.uk/showcase/field.cgi?id=6150
	20002 (*hypertension* code: 1065; *essential hypertension* code: 1072); https://biobank.ndph.ox.ac.uk/showcase/field.cgi?id=20002
	6177 (*blood pressure medication* code: 2); https://biobank.ndph.ox.ac.uk/showcase/field.cgi?id=6177
	6153 (*blood pressure medication* code: 2); https://biobank.ndph.ox.ac.uk/showcase/field.cgi?id=6153
	4080; https://biobank.ndph.ox.ac.uk/showcase/field.cgi?id=4080
	4079; https://biobank.ndph.ox.ac.uk/showcase/field.cgi?id=4079
ASI	21021; https://biobank.ndph.ox.ac.uk/showcase/field.cgi?id=21021
	4204; https://biobank.ndph.ox.ac.uk/showcase/field.cgi?id=4204
	4206; https://biobank.ndph.ox.ac.uk/showcase/field.cgi?id=4206
CIMT	22672; https://biobank.ndph.ox.ac.uk/showcase/field.cgi?id=22672
	22675; https://biobank.ndph.ox.ac.uk/showcase/field.cgi?id=22675
	22678; https://biobank.ndph.ox.ac.uk/showcase/field.cgi?id=22678
	22681; https://biobank.ndph.ox.ac.uk/showcase/field.cgi?id=22681
CHD	6150 (*heart attack* code: 1; *angina* code: 2); https://biobank.ndph.ox.ac.uk/showcase/field.cgi?id=6150
	20002 (*angina* code: 1074, *heart attack/myocardial infarction* code: 1075); https://biobank.ndph.ox.ac.uk/showcase/field.cgi?id=20002
	20004 (*coronary angioplasty (ptca) +/- stent* code: 1070; *coronary artery bypass grafts (cabg)* code: 1095; *triple heart bypass* code: 1523); https://biobank.ndph.ox.ac.uk/showcase/field.cgi?id=20004
	3894; https://biobank.ndph.ox.ac.uk/showcase/field.cgi?id=3894

* For lung function data, we used FVC and FEV_1 "best measure," as proposed in the UK BiLEVE (Biobank Lung Exome Variant Evaluation) study[7].

Table S3: Definition of cardiovascular traits in the UK Biobank, China Kadoorie Biobank, and FinnGen datasets.

**Variable**	**UKB**	**CKB**	**FinnGen**	**Notes**
ASI	Measured in m/s	–	–	
CHD	- Self-reported heart attack (myocardial infarction) or angina<br>*or*<br>- Coronary artery bypass graft surgery, triple heart bypass, or coronary angioplasty (with or without a stent)	First event ICD-10 codes in *cause of death*, *disease reporting* and *inpatient health insurance*: I20-I25	Main entry ICD-10 codes:<br>- *hospital discharge records*: I21; I22; I200<br>- *cause of death*: I21-I25; I46; R96; R98	I20: angina pectoris<br>I21: acute myocardial infarction (heart attack)<br>I22: subsequent myocardial infarction (MI)<br>I23: complications following acute MI<br>I24: other acute ischemic heart diseases<br>I25: chronic ischemic heart disease<br>I46: cardiac arrest<br>I200: unstable angina<br>R96: other sudden death, cause unknown<br>R98: unattended death
CIMT	Measured in mm	Measured in mm	–	
HTN	SBP ≥140 mmHg<br>*or* DBP ≥90 mmHg<br>*or* antihypertensive treatment	SBP > 140 mmHg<br>*and* DBP > 90 mmHg	Main entry ICD-10 codes for *hospital discharge records* and *cause of death*: I10-I13, I15, I674)	I10: essential (primary) hypertension<br>I11: hypertensive heart disease<br>I12: hypertensive chronic kidney disease<br>I13: hypertensive heart & chronic kidney disease<br>I15: secondary hypertension<br>I674: hypertensive encephalopathy
SBP	Measured in mmHg	Measured in mmHg	–	In our UKB analyses, BP values adjusted for antihypertensive treatment: + mmHg for SBP; + mmHg for DBP.<br>No adjustment performed in CKB analyses
DBP	Measured in mmHg	Measured in mmHg	–	
PP	Difference between SBP and DBP (mmHg)	Difference between SBP and DBP (mmHg)	–	In our UKB analyses, PP values calculated from adjusted BP values (see above)

ASI: Arterial Stiffness Index; CHD: Coronary heart disease; CIMT: Carotid intima-media thickness; HTN: Hypertension; PP: Pulse pressure; SBP/DBP: Systolic/diastolic blood pressure.

Table S4: Baseline characteristics of the UK Biobank and China Kadoorie Biobank (baseline characteristics not available for the publicly available FinnGen dataset).

Variable	UKB	CKB	Notes
Age (years)[1]	55.9 (8.1)	53.4 (10.9)	
Female[2]	163,618 (53.4)	47,316 (56.5)	
Ever smoking[2]	185,792 (60.6)	34,686 (41.4)	
Height (m)[1]	1.69 (0.09)	1.58 (0.08)	
Weight (kg)[1]	79.9 (16.6)	59.2 (10.7)	
BMI (kg/m2)[1]	27.5 (4.8)	23.5 (3.4)	
FVC (ml)[1]	3,899 (1009)	2,648 (776)	
FEV1/FVC (%)[1]	75.4 (6.6)	83.1 (8.9)	
FEV1 (ml)[1]	2,854 (779.2)	2,208 (689)	
ASI (m/s)[1]	8.7 (1.2)	–	Not measured in CKB
CHD[2]	19,294 (4.8)	15,538 (18.7)	The CKB sample is enriched with CHD cases and is therefore not representative of the general population
CIMT (mm)[1]	0.80 (0.15)	0.66 (0.15)	
HTN[2]	217,771 (57.0)	29,110 (35.0)	Unlike in our UKB analyses, antihypertensive treatment not included in the definition in CKB (See Table S3)
SBP (mmHg)[1]	138.2 (18.6)	135.7 (24.3)	BP values adjusted for antihypertensive treatment in our UKB analyses but not in CKB analyses (See Table S3)
DBP (mmHg)[1]	82.2 (10.1)	80.05 (12.8)	
PP (mmHg)[1]	57.0 (14.2)	55,63 (16.6)	PP values calculated from adjusted BP values in UKB analyses but not in CKB analyses (See Table S3)

[1] Mean (SD); [2] Number (%).

Abbreviations: BMI: Body Mass Index; FVC: Forced Vital Capacity; FEV1/FVC: Forced Expiratory Volume in one second to Forced Vital Capacity ratio; FEV1: Forced Expiratory Volume in one second; ASI: Arterial Stiffness Index; CHD: Coronary Heart Disease; CIMT: Carotid Intima-Media Thickness; HTN: Hypertension; PP: Pulse Pressure; SBP/DBP: Systolic/Diastolic Blood Pressure.

Table S5: Supporting evidence for the 7 genes not previously identified as associated with cardiovascular traits by published GWASs. See text and Table 1.

Gene	Results of association analysis with CV trait (SNP and p-value *)	Supporting evidence (including literature references)
CLDN20	PP (rs1969863; p=6.6^{-09})	- CLDN20 was significantly decreased after ischemia in a multi-omics study of responses to ischemic stroke[8]<br>Other claudins play a role in the regulation of blood pressure - e.g. CLDN3 in pregnancy-induced hypertension[9]; CLDN4 in regulation of blood pressure through kidney chloride reabsorption[10]
CSNK2B	HTN (rs3117578; p=1.0^{-15})<br>SBP (rs3117578; p=5.5^{-09})<br>DBP (rs9267531; p=1.5^{-17})	- The activity of the protein kinase CK2 has been associated with a number of CV diseases, such as cardiac ischemia–reperfusion injury, atherosclerosis, and cardiac hypertrophy[11, 12]. In particular, the *CSNK2B* gene regulates Wnt signalling pathways, which plays a key role in lung and cardiovascular development[13, 14]
GFI1	HTN (rs6676846; p=2.7^{-06})	- DNA methylation at *GFI1* linked to atherosclerosis at multiple vascular sites[15]
KAT8<br>(also called *MOF* or *MYST1*)	DBP (rs60996860; p=6.5^{-07})	- MOF linked to *cardiac hypertrophy* in mouse model[16]<br>- IMPC mouse mutant for KAT 8 shows altered CV phenotypes[17]<br>- MYST1 associated with *severity of atherosclerosis* in tissue of carotid plaques[18]<br>- KAT8 expressed in tissue of *abdominal aortic aneurysm*[19]
MAPRE1	PP (rs402951; p=6.7^{-07})	- *MAPRE1* plays a role in cardiomyocyte integrity and adaptation to pressure overload through stress-responsive signalling[20], and in cardiac conduction and arrhythmias[21, 22]
MMP24	PP (rs6120880; p=1.3^{-08})<br>CIMT (rs7280; p=4.0^{-09})<br>CHD (rs2275274; p=1.1^{-07})	Many members of the MMPs family have been *linked* to CV outcomes[23, 24]. For *MMP24* in particular:<br>- *MMP24* can activate *MMP2*, which has a strong link with cardiovascular outcomes[25]<br>- *MMP24* associated with intracranial aneurysms[26]
SERPING1	PP (rs10896631; p=2.4^{-12})<br>SBP (rs10896631, p=9.3^{-09})	- C1-Inhibitor (coded by *SERPING1*) expressed in the heart after *acute myocardial infarction*[27]<br>- SerpinG1 levels associated with *heart failure risk and severity*[28]

* p-value obtained from summary statistics using linear/logistic regression analysis, adjusted for age, sex, genotyping array, centre, and 10 ancestry principal components.

Abbreviations: CV: Cardiovascular; HTN: Hypertension; SBP: Systolic Blood Pressure; DBP: Diastolic Blood Pressure; MAP: Mean Arterial Pressure; MI: Myocardial Infarction; PP: Pulse Pressure; CHD: Coronary Heart Disease; CIMT: Carotid Intima-Media Thickness.

Table S6: FinnGen and CKB. The effect allele (EA) is the allele associated with decreased lung function (FVC and FEV_1/FVC), as in Table 3. **In bold**: results replicated in FinnGen and/or CBK at nominal level (same effect direction and $p<0.05$); an asterisk indicates replication at Bonferroni correction, $p<2.2*10^{-3}$ (0.05/23 tests). Replication 1-side p-values are calculated only for effect estimates in the same direction as in UKB. Associations with additional CV traits are reported in Notes.

Gene	SNP	EA	EAF	LF trait	CV trait	UKB		FinnGen			CKB		
						Beta	*p*	Beta	*p (1-side p)*	Notes	Beta	*p (1-side p)*	Notes
CLDN20	rs1969863	T	0.62	FVC	**PP**	0.17	6.6^{-09}	/	/		0.37	0.11 (**0.05**)	Very low MAF (0.02) in CKB
***CSNK2B* ***	rs3117578	G	0.85	FVC	SBP	0.32	5.5^{-09}	/	/	Also associated with **MI** (1.05; $p=6.6^{-04}$)	-0.47	0.17	Very low MAF (0.02) in CKB
					**HTN**	1.05	1.0^{-15}	1.04	9.7^{-08} (**4.8^{-08}**) *****		1.01	0.78 (0.39)	
					**CHD**	1.04	3.1^{-05}	1.06	2.9^{-06} (**1.4^{-06}**) *****		1.01	0.87 (0.44)	
CSNK2B	rs9267531	G	0.13	FEV_1/FVC	PP	0.23	2.1^{-08}	/	/	Associated with **MI** (1.05; $p=7.5^{-03}$)	/	/	CKB data not available
					DBP	-0.28	1.5^{-17}	/	/		/	/	
***FARP2* ***	rs139354822	T	0.97	FEV_1/FVC	PP	0.47	1.0^{-07}	/	/		/	/	CKB data not available
					SBP	0.68	2.3^{-08}	/	/				
					**HTN**	1.10	2.8^{-09}	1.06	5.3^{-05} (**2.6^{-05}**) *****				
***GFI1* ***	rs6676846	A	0.79	FEV_1/FVC	**HTN**	0.97	2.7^{-06}	0.98	1.7^{-03} (**8.3^{-04}**) *****		0.99	0.70 (0.35)	Low MAF (0.04) in CKB
IGF1[1]	rs10745941	T	0.76	FVC	**HTN**	1.03	3.7^{-06}	1.02	0.02 (**9.3^{-03}**)		1.01	0.75 (0.37)	
ITGB5	rs17282078	A	0.13	FVC	**CHD**	1.06	6.2^{-10}	1.03	0.04 (**0.02**)	Also associated with **MI** (1.04; $p=0.03$)	1.01	0.72 (0.36)	
KAT8	rs1978487	T	0.64	FVC	**HTN**	1.02	5.4^{-05}	1.01	0.06 (**0.03**)		1.02	0.25 (0.13)	Associated with: **SBP** (0.50; $p=3.3^{-03}$); **DBP** (0.32, $p=7.9^{-04}$)
MMP24	rs6120880	G	0.42	FVC	PP	0.16	1.3^{-08}	/	/	Associated with **CHD** (1.05; $p=8.9^{-03}$)	0.10	0.14 (0.07)	
					CIMT	0.01	5.0^{-08}	/	/		0.01	0.19 (0.09)	
PPARD	rs2395623	T	0.23	FEV_1/FVC	DBP	0.14	1.0^{-07}	/	/		-0.12	0.04	
RUNX3	rs111283598	G	0.96	FEV_1/FVC	DBP	-0.26	1.3^{-06}	/	/	Associated with **HTN** (0.95; $p=3.0^{-08}$)	-0.09	0.38 (0.19)	
SERPING1	rs10896631	C	0.27	FEV_1/FVC	**PP**	-0.22	2.4^{-12}	/	/		-0.16	0.08 (**0.04**)	
					SBP	-0.25	9.3^{-09}	/	/		-0.16	0.24 (0.12)	
***TNS1* ***	rs2571445	A	0.39	FVC, FEV_1/FVC	**SBP**	0.21	1.6^{-07}	/	/	Also associated with **MI** (1.04; $p=1.3^{-04}$)	0.21	0.03 (**0.02**)	
					**DBP**	0.13	5.4^{-09}	/	/		0.12	0.03 (**0.01**)	
					**HTN**	1.02	1.5^{-05}	1.01	0.02 (**8.4^{-03}**)		0.99	0.32	
					**CHD**	1.04	2.9^{-07}	1.04	3.7^{-07} (**1.9^{-07}**) *****		1.02	0.25 (0.12)	

1 A proxy was used for FinnGen, rs5742694 (r^2=0.91) as rs10745941 was not available

References

1. Tobin MD, Sheehan NA, Scurrah KJ, Burton PR. Adjusting for treatment effects in studies of quantitative traits: antihypertensive therapy and systolic blood pressure. Stat Med. 2005;24(19):2911-35.
2. Giambartolomei C, Vukcevic D, Schadt EE, Franke L, Hingorani AD, Wallace C, et al. Bayesian test for colocalisation between pairs of genetic association studies using summary statistics. PLoS Genet. 2014;10(5):e1004383.
3. Portas L, Pereira M, Shaheen SO, Wyss AB, London SJ, Burney PGJ, et al. Lung Development Genes and Adult Lung Function. Am J Respir Crit Care Med. 2020;202(6):853-65.
4. Wyss AB, Sofer T, Lee MK, Terzikhan N, Nguyen JN, Lahousse L, et al. Multiethnic meta-analysis identifies ancestry-specific and cross-ancestry loci for pulmonary function. Nat Commun. 2018;9(1):2976.
5. Gelman A. Why We (Usually) Don't Have to Worry About Multiple Comparisons. Journal of Research on Educational Effectiveness. 2012;5(2):189-211.
6. Shrine N, Guyatt AL, Erzurumluoglu AM, Jackson VE, Hobbs BD, Melbourne CA, et al. New genetic signals for lung function highlight pathways and chronic obstructive pulmonary disease associations across multiple ancestries. Nat Genet. 2019;51(3):481-93.
7. Wain LV, Shrine N, Miller S, Jackson VE, Ntalla I, Soler Artigas M, et al. Novel insights into the genetics of smoking behaviour, lung function, and chronic obstructive pulmonary disease (UK BiLEVE): a genetic association study in UK Biobank. Lancet Respir Med. 2015;3(10):769-81.
8. Simats A, Ramiro L, Garcia-Berrocoso T, Brianso F, Gonzalo R, Martin L, et al. A Mouse Brain-based Multi-omics Integrative Approach Reveals Potential Blood Biomarkers for Ischemic Stroke. Mol Cell Proteomics. 2020;19(12):1921-36.
9. Zhao A, Qi Y, Liu K. CLDN3 expression and function in pregnancy-induced hypertension. Exp Ther Med. 2020;20(4):3798-806.
10. Gong Y, Yu M, Yang J, Gonzales E, Perez R, Hou M, et al. The Cap1-claudin-4 regulatory pathway is important for renal chloride reabsorption and blood pressure regulation. Proc Natl Acad Sci U S A. 2014;111(36):E3766-74.
11. Borgo C, D'Amore C, Sarno S, Salvi M, Ruzzene M. Protein kinase CK2: a potential therapeutic target for diverse human diseases. Signal Transduct Target Ther. 2021;6(1):183.
12. Asif M, Kaygusuz E, Shinawi M, Nickelsen A, Hsieh TC, Wagle P, et al. De novo variants of CSNK2B cause a new intellectual disability-craniodigital syndrome by disrupting the canonical Wnt signaling pathway. HGG Adv. 2022;3(3):100111.
13. De Langhe SP, Reynolds SD. Wnt signaling in lung organogenesis. Organogenesis. 2008;4(2):100-8.
14. Tian Y, Cohen ED, Morrisey EE. The importance of Wnt signaling in cardiovascular development. Pediatr Cardiol. 2010;31(3):342-8.
15. Ammous F, Zhao W, Lin L, Ratliff SM, Mosley TH, Bielak LF, et al. Epigenetics of single-site and multi-site atherosclerosis in African Americans from the Genetic Epidemiology Network of Arteriopathy (GENOA). Clin Epigenetics. 2022;14(1):10.
16. Qiao W, Zhang W, Gai Y, Zhao L, Fan J. The histone acetyltransferase MOF overexpression blunts cardiac hypertrophy by targeting ROS in mice. Biochem Biophys Res Commun. 2014;448(4):379-84.
17. Mouse Genome Informatics Web Site [Available from: http://www.informatics.jax.org/allele/genoview/MGI:6461361?counter=2#cardiovascular_system_id.
18. Greissel A, Culmes M, Burgkart R, Zimmermann A, Eckstein HH, Zernecke A, et al. Histone acetylation and methylation significantly change with severity of atherosclerosis in human carotid plaques. Cardiovasc Pathol. 2016;25(2):79-86.
19. Han Y, Tanios F, Reeps C, Zhang J, Schwamborn K, Eckstein HH, et al. Histone acetylation and histone acetyltransferases show significant alterations in human abdominal aortic aneurysm. Clin Epigenetics. 2016;8:3.

20. Trembley MA, Quijada P, Agullo-Pascual E, Tylock KM, Colpan M, Dirkx RA, Jr., et al. Mechanosensitive Gene Regulation by Myocardin-Related Transcription Factors Is Required for Cardiomyocyte Integrity in Load-Induced Ventricular Hypertrophy. Circulation. 2018;138(17):1864-78.
21. Marchal GA, Jouni M, Chiang DY, Perez-Hernandez M, Podliesna S, Yu N, et al. Targeting the Microtubule EB1-CLASP2 Complex Modulates Na(V)1.5 at Intercalated Discs. Circ Res. 2021;129(3):349-65.
22. Marchal GA, Galjart N, Portero V, Remme CA. Microtubule plus-end tracking proteins: novel modulators of cardiac sodium channels and arrhythmogenesis. Cardiovasc Res. 2023;119(7):1461-79.
23. Belo VA, Guimaraes DA, Castro MM. Matrix Metalloproteinase 2 as a Potential Mediator of Vascular Smooth Muscle Cell Migration and Chronic Vascular Remodeling in Hypertension. J Vasc Res. 2015;52(4):221-31.
24. Azevedo A, Prado AF, Antonio RC, Issa JP, Gerlach RF. Matrix metalloproteinases are involved in cardiovascular diseases. Basic Clin Pharmacol Toxicol. 2014;115(4):301-14.
25. In: Zivkovic M, Travascio, F., editor. The Role of Matrix Metalloproteinase in Human Body Pathologies.
26. Kim BJ, Hong EP, Youn DH, Jeon JP, First Korean Stroke Genetics Association R. Genome-Wide Association Study of the Relationship Between Matrix Metalloproteinases and Intracranial Aneurysms. J Clin Neurol. 2022;18(2):163-70.
27. Emmens RW, Baylan U, Juffermans LJ, Karia RV, Ylstra B, Wouters D, et al. Endogenous C1-inhibitor production and expression in the heart after acute myocardial infarction. Cardiovasc Pathol. 2016;25(1):33-9.
28. Zhang YN, Vernooij F, Ibrahim I, Ooi S, Gijsberts CM, Schoneveld AH, et al. Extracellular Vesicle Proteins Associated with Systemic Vascular Events Correlate with Heart Failure: An Observational Study in a Dyspnoea Cohort. PLoS One. 2016;11(1):e0148073.
