# Supplementary material for: Lung development genes, adult lung function and cardiovascular comorbidities
Source: Thorax. 2025 May 30;80(10):e222474. doi: 10.1136/thorax-2024-222474 (PMC12505039; doi:10.1136/thorax-2024-222474)
Supplement: online supplemental file 2 [file thorax-80-10-s002.pdf]

Table S2. 15,298 SNPs in the 55 genes included in the colocalization analysis.

SNP	Chromosome	Position	Effect Allele	Other Allele	Effect Allele Frequency	Imputation quality	Gene
rs116010861	1	25,226,986	G	A	0.986	0.938	RUNX3
rs112094569	1	25,227,230	G	A	0.98	0.937	RUNX3
rs3766283	1	25,227,535	T	G	0.989	0.787	RUNX3
rs2003679	1	25,227,538	T	G	0.948	0.967	RUNX3
rs13157	1	25,227,677	G	A	0.956	0.965	RUNX3
rs111283598	1	25,229,307	G	A	0.956	0.994	RUNX3
rs543620002	1	25,229,313	T	C	0.985	0.623	RUNX3
rs34296216	1	25,229,390	G	GC	0.873	0.899	RUNX3
rs115992982	1	25,229,792	G	A	0.984	0.934	RUNX3
rs7536526	1	25,230,131	C	T	0.96	0.968	RUNX3
rs11249200	1	25,231,355	C	G	0.96	0.967	RUNX3
rs9438875	1	25,231,562	G	T	0.66	0.987	RUNX3
rs112128596	1	25,231,674	G	A	0.988	0.807	RUNX3
rs116379175	1	25,231,807	A	G	0.954	0.975	RUNX3
rs114043123	1	25,233,660	G	A	0.967	0.976	RUNX3
rs34878696	1	25,234,062	T	G	0.589	0.994	RUNX3
rs74060469	1	25,234,156	C	T	0.959	0.966	RUNX3
rs181862027	1	25,234,207	G	A	0.949	0.908	RUNX3
rs145866205	1	25,234,307	G	A	0.981	0.961	RUNX3
rs113470029	1	25,234,489	T	C	0.588	0.994	RUNX3
rs72874394	1	25,234,515	G	A	0.589	0.993	RUNX3
rs112694611	1	25,234,615	A	G	0.6	0.963	RUNX3
rs111733290	1	25,234,668	G	C	0.559	0.949	RUNX3
rs12082398	1	25,234,781	T	C	0.585	0.994	RUNX3
rs141903105	1	25,234,810	G	C	0.981	0.961	RUNX3
rs962544	1	25,235,108	T	C	0.589	0.994	RUNX3
rs150972362	1	25,237,503	G	T	0.988	0.953	RUNX3
rs1984520	1	25,237,986	C	T	0.632	0.992	RUNX3
rs116292813	1	25,239,017	T	A	0.987	0.949	RUNX3
rs16830141	1	25,239,289	T	C	0.96	0.965	RUNX3
rs113585396	1	25,239,362	A	G	0.984	0.893	RUNX3
rs2236850	1	25,240,341	T	C	0.581	0.992	RUNX3
rs2282718	1	25,241,056	G	A	0.629	0.977	RUNX3
rs2282719	1	25,241,090	A	C	0.44	0.96	RUNX3
rs9438876	1	25,241,116	A	G	0.487	0.958	RUNX3
rs5773115	1	25,241,669	C	CT	0.985	0.98	RUNX3
rs1003699	1	25,242,576	C	T	0.958	0.959	RUNX3
rs12142264	1	25,242,730	T	C	0.983	0.99	RUNX3
rs12137677	1	25,242,926	G	A	0.983	0.989	RUNX3
rs57931142	1	25,243,194	A	G	0.983	0.989	RUNX3
rs7535613	1	25,243,474	G	A	0.983	0.991	RUNX3
rs2236851	1	25,243,554	C	T	0.819	0.934	RUNX3
rs7537912	1	25,243,598	G	T	0.983	0.988	RUNX3
rs12408466	1	25,243,675	G	T	0.584	0.958	RUNX3
rs2236852	1	25,243,767	A	G	0.566	0.958	RUNX3
rs6697340	1	25,244,706	C	T	0.958	0.959	RUNX3
1:25244717_TAGGCCTCCTGCCCC_T	1	25,244,717	TAGGCCTCCTGCCCC	T	0.987	0.984	RUNX3
1:25245056_TTTCA_T	1	25,245,056	TTTCA	T	0.986	0.948	RUNX3
rs9438861	1	25,245,081	G	T	0.193	0.954	RUNX3
rs138838625	1	25,245,085	G	T	0.956	0.946	RUNX3
rs577677713	1	25,246,374	G	GT	0.956	0.902	RUNX3
rs138960076	1	25,248,273	G	A	0.988	0.886	RUNX3
rs7518125	1	25,249,252	C	A	0.423	0.996	RUNX3
1:25250533_CA_C	1	25,250,533	CA	C	0.423	0.995	RUNX3
rs10794666	1	25,250,830	C	T	0.426	0.999	RUNX3
rs34349689	1	25,250,919	G	GT	0.43	0.992	RUNX3
rs742230	1	25,251,424	G	A	0.423	0.999	RUNX3
rs742231	1	25,251,456	G	A	0.423	0.999	RUNX3
rs742232	1	25,251,706	T	C	0.423	0.999	RUNX3
rs760805	1	25,251,923	A	T	0.426	0.999	RUNX3
rs111802397	1	25,252,652	C	T	0.958	0.972	RUNX3
rs7517302	1	25,254,317	C	T	0.423	0.997	RUNX3
rs537002152	1	25,254,852	A	C	0.988	0.893	RUNX3
rs79784290	1	25,255,850	C	G	0.954	0.96	RUNX3
rs75012901	1	25,255,982	C	T	0.965	0.987	RUNX3
rs71514255	1	25,256,444	G	A	0.424	0.994	RUNX3
rs7520923	1	25,257,915	G	C	0.954	0.96	RUNX3
rs4649037	1	25,258,301	G	T	0.422	0.996	RUNX3
rs11249203	1	25,258,585	G	C	0.422	0.996	RUNX3
rs61774728	1	25,258,867	G	C	0.422	0.992	RUNX3
rs3845302	1	25,259,884	C	G	0.419	0.995	RUNX3
rs4648883	1	25,261,203	T	C	0.421	0.992	RUNX3
rs4648884	1	25,261,386	T	C	0.421	0.992	RUNX3
rs10903115	1	25,261,696	A	G	0.42	0.99	RUNX3
rs11249204	1	25,261,740	A	G	0.42	0.989	RUNX3
rs1005733	1	25,261,960	T	A	0.423	0.987	RUNX3
rs1005734	1	25,262,022	C	A	0.424	0.987	RUNX3
1:25262401_GA_G	1	25,262,401	GA	G	0.954	0.934	RUNX3
rs77435405	1	25,263,043	C	T	0.976	0.91	RUNX3
rs116347778	1	25,263,151	C	T	0.987	0.901	RUNX3
rs141390120	1	25,263,306	C	T	0.985	0.896	RUNX3
rs1293202	1	25,263,997	G	A	0.872	0.948	RUNX3
rs72876127	1	25,264,853	C	T	0.987	0.974	RUNX3
rs148525958	1	25,265,681	G	A	0.99	0.946	RUNX3
rs79175418	1	25,266,109	G	A	0.934	0.95	RUNX3
rs74759232	1	25,266,446	C	T	0.989	0.951	RUNX3
rs12725096	1	25,268,289	A	T	0.948	0.899	RUNX3
rs759156031	1	25,269,282	GA	G	0.984	0.84	RUNX3
rs61774731	1	25,269,355	G	A	0.907	0.944	RUNX3
rs72876135	1	25,269,437	C	G	0.964	0.946	RUNX3
rs115839673	1	25,270,009	G	A	0.983	0.862	RUNX3
rs61774732	1	25,270,251	C	A	0.911	0.948	RUNX3
rs61774733	1	25,270,344	G	A	0.845	0.945	RUNX3
rs1395621	1	25,270,572	T	C	0.518	0.951	RUNX3
rs77105859	1	25,270,583	C	T	0.934	0.948	RUNX3
rs56218248	1	25,271,352	A	G	0.933	0.949	RUNX3
rs12117581	1	25,272,809	G	A	0.963	0.948	RUNX3
rs142059784	1	25,273,017	G	A	0.983	0.923	RUNX3
rs79598985	1	25,273,023	G	A	0.934	0.949	RUNX3
rs7551188	1	25,273,200	T	C	0.513	0.966	RUNX3
rs876109	1	25,273,288	T	C	0.386	0.971	RUNX3
rs181922971	1	25,274,154	T	C	0.988	0.908	RUNX3
rs6688058	1	25,274,998	G	A	0.875	0.955	RUNX3
rs1293203	1	25,275,859	C	T	0.649	0.969	RUNX3
rs12744797	1	25,276,521	T	A	0.951	0.997	RUNX3
rs61774734	1	25,276,537	G	A	0.9	0.97	RUNX3
rs779477785	1	25,277,695	CA	C	0.922	0.836	RUNX3
rs11249206	1	25,277,982	C	T	0.486	0.993	RUNX3
rs1507099	1	25,278,451	A	C	0.368	0.987	RUNX3
rs534122042	1	25,278,452	C	T	0.987	0.911	RUNX3
rs10127543	1	25,279,116	G	C	0.492	0.995	RUNX3
rs2135756	1	25,279,180	C	T	0.491	0.996	RUNX3
rs758892170	1	25,279,443	A	AT	0.96	0.895	RUNX3
rs61774735	1	25,279,639	C	T	0.89	0.982	RUNX3
rs71577738	1	25,279,854	G	GA	0.639	0.873	RUNX3
rs12124894	1	25,280,908	A	T	0.89	0.984	RUNX3
rs4478762	1	25,281,015	G	A	0.89	0.984	RUNX3
rs1848186	1	25,282,856	G	T	0.381	0.992	RUNX3
rs1848185	1	25,282,961	T	C	0.378	0.993	RUNX3
rs4288539	1	25,282,993	G	A	0.379	0.993	RUNX3
rs11249207	1	25,283,069	C	T	0.379	0.993	RUNX3
rs11249208	1	25,283,126	G	A	0.379	0.993	RUNX3
rs10903116	1	25,283,162	C	T	0.379	0.993	RUNX3
rs12031692	1	25,283,274	A	C	0.379	0.993	RUNX3
rs11580845	1	25,283,356	C	G	0.379	0.993	RUNX3
rs10903117	1	25,283,592	G	A	0.379	0.993	RUNX3
rs7527098	1	25,284,666	T	C	0.379	0.993	RUNX3
rs6600245	1	25,284,678	T	C	0.378	0.992	RUNX3
rs115130234	1	25,285,828	C	T	0.977	0.87	RUNX3
rs12126847	1	25,288,313	A	G	0.87	0.987	RUNX3
rs60097574	1	25,288,832	A	C	0.87	0.989	RUNX3
rs66880507	1	25,289,275	T	C	0.87	0.989	RUNX3
rs11249209	1	25,289,427	G	A	0.87	0.99	RUNX3
rs11249210	1	25,289,612	T	C	0.87	0.99	RUNX3
rs72657048	1	25,289,734	C	G	0.503	0.994	RUNX3
rs11802714	1	25,289,794	G	C	0.87	0.99	RUNX3
rs12128982	1	25,290,446	A	G	0.87	0.991	RUNX3
rs141682513	1	25,290,698	C	T	0.981	0.905	RUNX3
rs6676141	1	92,939,217	T	C	0.794	0.994	GFI1
rs58394161	1	92,939,959	T	C	0.847	0.992	GFI1
rs11582379	1	92,939,978	T	C	0.868	0.988	GFI1
rs41313373	1	92,940,411	C	T	0.874	0.947	GFI1
rs1325432	1	92,940,850	G	T	0.834	0.972	GFI1
rs4970714	1	92,941,357	A	G	0.355	0.99	GFI1
rs6676846	1	92,942,352	G	A	0.206	0.992	GFI1
rs942753	1	92,942,466	G	A	0.356	0.993	GFI1
rs80337126	1	92,944,188	G	A	0.977	0.965	GFI1
rs4970702	1	92,944,994	A	G	0.356	0.993	GFI1
rs528617828	1	92,945,129	T	G	0.962	0.638	GFI1
rs11164601	1	92,945,507	T	C	0.355	0.993	GFI1
rs11164605	1	92,946,479	G	A	0.206	0.993	GFI1
rs10874656	1	92,947,024	T	C	0.206	0.993	GFI1
rs11164607	1	92,947,138	A	C	0.356	0.993	GFI1

SNP	Chromosome	Position	Effect Allele	Other Allele	Effect Allele Frequency	Imputation quality	Gene
rs11164608	1	92,947,427	C	T	0.356	0.993	GFI1
rs200635146	1	92,947,509	T	TGCGCGCGC	0.065	0.62	GFI1
rs115971380	1	92,947,689	C	T	0.98	0.987	GFI1
rs11448559	1	92,947,981	G	GC	0.355	0.986	GFI1
rs185633223	1	92,950,315	G	A	0.986	0.909	GFI1
rs61779244	1	92,950,404	C	G	0.969	0.971	GFI1
rs79160953	1	92,950,515	T	A	0.894	0.989	GFI1
rs4565725	1	92,950,937	A	G	0.355	0.993	GFI1
rs6689470	1	92,951,210	G	A	0.851	0.993	GFI1
rs6603975	1	92,951,239	A	G	0.206	0.993	GFI1
rs150037086	1	92,952,080	G	GC	0.313	0.964	GFI1
rs114423517	1	113,009,315	C	T	0.983	0.938	WNT2B
rs2488786	1	113,010,808	A	G	0.384	0.98	WNT2B
rs145437926	1	113,011,064	C	T	0.971	0.969	WNT2B
rs7416024	1	113,011,244	C	T	0.762	0.979	WNT2B
rs2798243	1	113,011,245	A	G	0.144	0.986	WNT2B
1:113011679_AG_A	1	113,011,679	AG	A	0.987	0.864	WNT2B
rs2483338	1	113,013,053	A	G	0.383	0.98	WNT2B
rs5777123	1	113,013,506	C	CT	0.761	0.967	WNT2B
rs371894864	1	113,015,682	G	GGAAAGGAAGAAAAA	0.771	0.951	WNT2B
rs796077467	1	113,015,761	G	GAAAAAGGAAGGAAGGA	0.149	0.962	WNT2B
rs185446963	1	113,016,544	G	T	0.95	0.935	WNT2B
rs72699029	1	113,016,701	A	G	0.34	0.959	WNT2B
1:113016934_AT_A	1	113,016,934	AT	A	0.337	0.969	WNT2B
rs1537413	1	113,017,515	A	C	0.146	0.982	WNT2B
rs1175649	1	113,019,277	T	G	0.1	0.973	WNT2B
rs1175650	1	113,019,574	T	C	0.147	0.98	WNT2B
1:113020096_TA_T	1	113,020,096	TA	T	0.148	0.968	WNT2B
rs200355435	1	113,020,912	T	TA	0.952	0.947	WNT2B
rs4644492	1	113,021,218	G	A	0.354	0.967	WNT2B
rs113839020	1	113,021,443	G	A	0.937	0.952	WNT2B
rs148440148	1	113,021,758	G	A	0.977	0.924	WNT2B
rs116006884	1	113,021,884	T	C	0.967	0.906	WNT2B
rs150550334	1	113,021,967	G	A	0.939	0.966	WNT2B
rs186674985	1	113,022,025	G	A	0.989	0.844	WNT2B
rs1175651	1	113,022,154	C	T	0.788	0.995	WNT2B
rs115486014	1	113,022,364	C	A	0.984	0.846	WNT2B
rs146763821	1	113,022,395	G	A	0.976	0.972	WNT2B
rs139488818	1	113,022,419	G	T	0.937	0.966	WNT2B
rs10857956	1	113,022,434	A	G	0.777	0.994	WNT2B
rs145241532	1	113,022,525	G	A	0.939	0.966	WNT2B
rs149176916	1	113,022,557	A	G	0.939	0.966	WNT2B
rs7554976	1	113,022,576	T	C	0.778	0.995	WNT2B
rs115264132	1	113,022,698	A	G	0.937	0.967	WNT2B
1:113022987_CA_C	1	113,022,987	CA	C	0.05	0.885	WNT2B
rs1759693	1	113,023,292	C	T	0.836	0.995	WNT2B
rs1777618	1	113,023,312	G	C	0.194	0.974	WNT2B
rs6657499	1	113,023,548	T	C	0.943	0.988	WNT2B
1:113023772_CAA_C	1	113,023,772	CAA	C	0.944	0.974	WNT2B
1:113023791_CAA_C	1	113,023,791	CAA	C	0.788	0.97	WNT2B
rs1777612	1	113,023,941	T	C	0.836	0.995	WNT2B
rs1620668	1	113,023,980	A	G	0.836	0.994	WNT2B
rs149473819	1	113,024,359	G	A	0.988	0.949	WNT2B
rs56768833	1	113,024,566	T	C	0.779	0.998	WNT2B
rs141779711	1	113,024,589	T	A	0.928	0.97	WNT2B
rs114036493	1	113,024,614	A	G	0.988	0.931	WNT2B
rs145551351	1	113,024,866	G	A	0.955	0.979	WNT2B
rs61820468	1	113,025,109	C	T	0.55	0.98	WNT2B
rs2780266	1	113,025,285	G	C	0.779	0.998	WNT2B
1:113025803_TACACAC_T	1	113,025,803	TACACAC	T	0.238	0.963	WNT2B
1:113026194_AT_A	1	113,026,194	AT	A	0.779	0.995	WNT2B
rs971790	1	113,026,282	A	G	0.461	0.984	WNT2B
1:113026401_CA_C	1	113,026,401	CA	C	0.453	0.963	WNT2B
1:113026485_AT_A	1	113,026,485	AT	A	0.985	0.825	WNT2B
rs971791	1	113,026,534	G	A	0.24	0.979	WNT2B
rs971792	1	113,026,557	T	C	0.461	0.984	WNT2B
rs7412537	1	113,026,693	A	G	0.779	0.998	WNT2B
rs77619873	1	113,026,708	C	A	0.943	0.987	WNT2B
1:113026764_TATA_T	1	113,026,764	TATA	T	0.461	0.975	WNT2B
rs10857957	1	113,026,801	T	A	0.779	0.997	WNT2B
rs11102481	1	113,026,852	T	C	0.837	0.976	WNT2B
rs145030807	1	113,026,894	C	CATAT	0.432	0.944	WNT2B
rs111969006	1	113,026,928	T	G	0.984	0.954	WNT2B
rs36078713	1	113,026,930	G	T	0.479	0.957	WNT2B
rs12729170	1	113,027,048	G	A	0.534	0.985	WNT2B
rs116179474	1	113,027,215	T	C	0.927	0.97	WNT2B
rs543169420	1	113,027,601	A	C	0.989	0.966	WNT2B
rs1759694	1	113,027,789	T	C	0.836	0.994	WNT2B
rs1759695	1	113,027,802	T	A	0.179	0.981	WNT2B
rs1777605	1	113,027,815	G	A	0.779	0.994	WNT2B
rs72699035	1	113,027,956	G	A	0.943	0.987	WNT2B
1:113028082_CA_C	1	113,028,082	CA	C	0.346	0.924	WNT2B
rs1777606	1	113,028,105	G	A	0.779	0.997	WNT2B
rs974442	1	113,028,271	T	C	0.461	0.984	WNT2B
rs1777607	1	113,028,740	G	C	0.781	0.992	WNT2B
rs1759696	1	113,028,932	T	G	0.24	0.98	WNT2B
rs1613341	1	113,029,062	C	A	0.779	0.996	WNT2B
rs1759697	1	113,029,169	T	C	0.836	0.993	WNT2B
rs72699043	1	113,029,189	C	A	0.916	0.544	WNT2B
1:113029281_GA_G	1	113,029,281	GA	G	0.779	0.993	WNT2B
rs143050529	1	113,030,284	G	A	0.939	0.968	WNT2B
rs61820471	1	113,030,791	C	A	0.943	0.987	WNT2B
rs72699044	1	113,030,985	C	G	0.944	0.986	WNT2B
1:113031059_CCTT_C	1	113,031,059	CCTT	C	0.22	0.993	WNT2B
rs79759984	1	113,031,771	A	T	0.938	0.971	WNT2B
rs57706772	1	113,031,851	G	C	0.944	0.986	WNT2B
rs35427726	1	113,032,404	A	AT	0.418	0.95	WNT2B
1:113032749_GAAAC_G	1	113,032,749	GAAAC	G	0.522	0.979	WNT2B
rs140243716	1	113,033,079	G	A	0.975	0.923	WNT2B
rs2780261	1	113,033,261	A	G	0.229	0.969	WNT2B
rs530730001	1	113,033,262	T	C	0.986	0.774	WNT2B
rs368153910	1	113,033,266	C	T	0.984	0.791	WNT2B
rs143157067	1	113,033,268	C	T	0.939	0.967	WNT2B
rs12074529	1	113,033,269	A	G	0.985	0.756	WNT2B
rs148247208	1	113,033,285	G	A	0.944	0.985	WNT2B
rs61820473	1	113,033,302	A	G	0.97	0.958	WNT2B
rs61820474	1	113,033,459	T	C	0.944	0.986	WNT2B
rs2483336	1	113,034,287	T	C	0.24	0.981	WNT2B
1:113035707_TATC_T	1	113,035,707	TATC	T	0.944	0.974	WNT2B
rs59075478	1	113,035,808	G	A	0.944	0.986	WNT2B
rs116684661	1	113,036,059	G	A	0.938	0.972	WNT2B
rs2749125	1	113,036,321	G	A	0.522	0.988	WNT2B
rs796828209	1	113,036,562	CA	C	0.281	0.936	WNT2B
rs115632187	1	113,036,576	C	T	0.939	0.969	WNT2B
rs79832592	1	113,036,648	C	T	0.836	0.992	WNT2B
rs57660177	1	113,037,015	C	G	0.944	0.985	WNT2B
rs78032946	1	113,037,364	G	C	0.97	0.975	WNT2B
rs72699046	1	113,038,761	G	C	0.929	0.99	WNT2B
rs351354	1	113,038,795	A	G	0.179	0.985	WNT2B
rs351355	1	113,038,812	T	C	0.46	0.987	WNT2B
rs75594559	1	113,039,049	C	T	0.934	0.977	WNT2B
rs71584741	1	113,039,227	G	GT	0.525	0.982	WNT2B
rs351356	1	113,039,291	T	C	0.404	0.986	WNT2B
rs351357	1	113,039,363	C	G	0.461	0.988	WNT2B
rs351358	1	113,039,993	G	A	0.179	0.989	WNT2B
rs202213862	1	113,040,041	G	T	0.958	0.549	WNT2B
rs351359	1	113,040,277	A	G	0.406	0.99	WNT2B
rs61820476	1	113,040,659	C	T	0.944	0.986	WNT2B
rs2798239	1	113,040,832	G	C	0.184	0.989	WNT2B
rs80006641	1	113,040,996	C	T	0.933	0.927	WNT2B
rs2749126	1	113,041,015	A	C	0.582	0.994	WNT2B
rs61820477	1	113,041,294	T	A	0.898	0.979	WNT2B
rs61820523	1	113,041,556	G	A	0.883	0.972	WNT2B
rs11412510	1	113,042,527	A	AT	0.52	0.985	WNT2B
rs11807828	1	113,042,549	G	A	0.944	0.985	WNT2B
rs351361	1	113,042,558	T	C	0.188	0.987	WNT2B
rs59063619	1	113,042,938	A	G	0.933	0.997	WNT2B
1:113043226_TA_T	1	113,043,226	TA	T	0.937	0.965	WNT2B
rs114309419	1	113,043,242	T	A	0.944	0.911	WNT2B
rs12038119	1	113,043,423	A	G	0.928	0.999	WNT2B
rs55633098	1	113,043,669	G	T	0.987	0.944	WNT2B
rs139450442	1	113,043,823	A	G	0.976	0.993	WNT2B
rs34840384	1	113,044,275	A	ATG	0.591	0.982	WNT2B
rs10776752	1	113,044,328	G	T	0.927	0.997	WNT2B
rs351362	1	113,044,731	C	T	0.592	0.987	WNT2B
rs351363	1	113,044,888	C	T	0.336	0.984	WNT2B
rs351364	1	113,045,061	A	T	0.252	0.989	WNT2B
rs351365	1	113,046,395	T	C	0.251	0.989	WNT2B
rs72993043	1	113,046,707	C	T	0.975	0.994	WNT2B
1:113046730_GT_G	1	113,046,730	GT	G	0.19	0.978	WNT2B
rs3790604	1	113,046,879	C	A	0.926	0.994	WNT2B
rs72993045	1	113,047,178	T	C	0.968	0.978	WNT2B
rs183114813	1	113,047,858	T	C	0.989	0.96	WNT2B
rs351366	1	113,049,651	C	T	0.972	0.907	WNT2B
rs351367	1	113,049,862	T	C	0.525	0.989	WNT2B

SNP	Chromosome	Position	Effect Allele	Other Allele	Effect Allele Frequency	Imputation quality	Gene
rs351368	1	113,049,941	A	G	0.525	0.99	WNT2B
rs3828074	1	113,050,985	C	T	0.681	0.999	WNT2B
rs11102484	1	113,051,730	C	G	0.681	0.996	WNT2B
rs148494162	1	113,051,781	C	T	0.989	0.91	WNT2B
rs3790605	1	113,052,155	C	G	0.679	0.994	WNT2B
rs3790606	1	113,052,186	G	C	0.679	0.994	WNT2B
rs11102485	1	113,052,923	C	G	0.922	0.994	WNT2B
rs3790607	1	113,053,023	A	C	0.922	0.993	WNT2B
rs351370	1	113,054,659	C	T	0.414	1	WNT2B
rs149630635	1	113,054,701	A	G	0.967	0.991	WNT2B
rs3790608	1	113,055,023	G	A	0.848	0.992	WNT2B
rs12138754	1	113,055,653	G	A	0.786	0.991	WNT2B
1:113055752_CA_C	1	113,055,752	CA	C	0.934	0.966	WNT2B
rs58118553	1	113,055,758	A	C	0.933	0.971	WNT2B
rs3790609	1	113,056,990	C	T	0.82	0.987	WNT2B
rs147162637	1	113,057,446	A	G	0.985	0.991	WNT2B
rs150759507	1	113,057,856	G	A	0.985	0.991	WNT2B
rs351372	1	113,059,220	T	A	0.476	0.995	WNT2B
rs3737136	1	113,060,432	A	G	0.798	0.995	WNT2B
rs146898695	1	113,061,478	A	G	0.986	0.994	WNT2B
rs551965872	1	113,061,654	C	G	0.968	0.707	WNT2B
rs1006298	1	113,061,959	G	A	0.984	0.903	WNT2B
1:113062694_ATGT_A	1	113,062,694	ATGT	A	0.805	0.99	WNT2B
rs910697	1	113,063,125	A	G	0.536	0.996	WNT2B
rs2273368	1	113,063,771	C	T	0.802	0.995	WNT2B
rs201498541	1	113,063,954	C	T	0.564	0.761	WNT2B
rs72699053	1	113,064,293	T	C	0.988	0.947	WNT2B
rs11102489	1	113,064,350	A	G	0.936	0.995	WNT2B
rs6668533	1	113,064,468	G	A	0.742	0.998	WNT2B
rs149187988	1	113,064,529	C	A	0.98	0.989	WNT2B
rs41283054	1	113,064,778	C	T	0.985	0.995	WNT2B
rs41283056	1	113,064,891	A	G	0.986	0.968	WNT2B
rs201433929	1	113,066,632	C	A	0.978	0.877	WNT2B
rs556883943	1	113,066,639	C	A	0.977	0.905	WNT2B
rs114323109	1	113,067,708	C	T	0.985	0.996	WNT2B
rs3790611	1	113,067,792	A	G	0.739	0.998	WNT2B
rs55664948	1	113,068,067	G	A	0.985	0.997	WNT2B
rs115875134	1	113,068,281	T	C	0.987	0.968	WNT2B
rs35147679	1	113,068,577	C	T	0.796	0.998	WNT2B
rs112806345	1	113,068,880	C	T	0.985	0.997	WNT2B
1:113069361_CA_C	1	113,069,361	CA	C	0.536	0.974	WNT2B
rs72699055	1	113,069,979	A	G	0.988	0.988	WNT2B
rs189961808	1	113,071,081	A	G	0.989	0.946	WNT2B
1:113071774_CTGAG_C	1	113,071,774	CTGAG	C	0.795	0.998	WNT2B
rs12401734	1	113,071,834	T	C	0.741	0.999	WNT2B
rs12402929	1	113,072,293	G	C	0.742	0.999	WNT2B
rs2273369	1	113,072,477	A	G	0.74	0.999	WNT2B
rs145598400	1	113,072,558	C	T	0.985	0.997	WNT2B
rs765351658	1	173,874,603	AT	A	0.889	0.99	SERPINC1
rs58759841	1	173,874,862	G	GA	0.889	0.968	SERPINC1
rs2227612	1	173,875,401	T	G	0.887	0.996	SERPINC1
rs677	1	173,876,561	C	G	0.886	0.994	SERPINC1
rs2759328	1	173,876,705	C	T	0.898	0.995	SERPINC1
rs374740349	1	173,877,122	AAATAATAATAATAATAATAATAATAAT	A	0.807	0.933	SERPINC1
rs148783362	1	173,877,862	C	A	0.965	0.957	SERPINC1
rs61827936	1	173,877,886	G	C	0.887	0.997	SERPINC1
rs141516930	1	173,878,109	C	T	0.984	0.936	SERPINC1
rs1799876	1	173,878,471	A	G	0.688	0.995	SERPINC1
rs5878	1	173,878,832	T	C	0.669	0.99	SERPINC1
rs5877	1	173,878,862	T	C	0.673	0.996	SERPINC1
rs184472294	1	173,879,454	T	C	0.989	0.811	SERPINC1
rs2227609	1	173,879,562	C	A	0.889	0.993	SERPINC1
rs2227607	1	173,880,752	C	A	0.89	0.994	SERPINC1
rs941989	1	173,881,871	C	T	0.674	0.996	SERPINC1
rs941988	1	173,881,893	C	T	0.898	0.996	SERPINC1
rs2227603	1	173,882,548	A	C	0.972	0.99	SERPINC1
rs2227601	1	173,882,730	T	C	0.886	0.996	SERPINC1
rs6695940	1	173,882,980	G	A	0.886	0.996	SERPINC1
1:173882992_CA_C	1	173,882,992	CA	C	0.788	0.85	SERPINC1
rs59197371	1	173,883,215	C	CA	0.786	0.915	SERPINC1
rs56288561	1	173,883,517	A	T	0.89	0.994	SERPINC1
rs2227597	1	173,884,739	C	G	0.886	0.996	SERPINC1
rs2227596	1	173,884,793	T	C	0.855	0.995	SERPINC1
rs146692719	1	173,885,113	G	A	0.983	0.926	SERPINC1
rs2227595	1	173,885,275	A	T	0.888	0.996	SERPINC1
rs2227594	1	173,885,481	C	T	0.886	0.996	SERPINC1
rs2227593	1	173,885,536	G	T	0.886	0.996	SERPINC1
rs2227592	1	173,885,712	C	T	0.898	0.995	SERPINC1
rs2227590	1	173,886,037	G	A	0.886	0.996	SERPINC1
rs2227589	1	173,886,216	C	T	0.898	0.995	SERPINC1
rs10482718	1	218,518,742	A	G	0.964	1	TGFB2
rs112369231	1	218,519,367	T	C	0.735	0.979	TGFB2
rs550941534	1	218,519,398	GCA	G	0.947	0.869	TGFB2
rs113711540	1	218,519,928	A	AAAAC	0.731	0.987	TGFB2
rs10482724	1	218,520,843	G	A	0.837	0.971	TGFB2
rs6658835	1	218,520,995	A	G	0.731	0.992	TGFB2
rs146675233	1	218,521,275	AT	A	0.779	0.768	TGFB2
rs2799098	1	218,521,609	G	A	0.177	1	TGFB2
rs79781677	1	218,522,690	G	A	0.964	1	TGFB2
rs17047682	1	218,523,025	A	G	0.933	0.948	TGFB2
rs181594871	1	218,523,164	A	G	0.98	0.945	TGFB2
rs1418555	1	218,523,650	G	A	0.77	0.997	TGFB2
rs1417488	1	218,523,730	C	T	0.745	0.988	TGFB2
rs11581605	1	218,524,304	A	G	0.77	0.998	TGFB2
rs2799097	1	218,524,632	A	G	0.151	0.99	TGFB2
rs11579807	1	218,524,641	T	C	0.838	0.976	TGFB2
rs4846476	1	218,526,228	G	C	0.77	0.999	TGFB2
rs115593700	1	218,526,843	T	G	0.987	0.907	TGFB2
rs79918460	1	218,527,064	T	C	0.987	0.879	TGFB2
rs141465001	1	218,530,031	G	A	0.986	0.959	TGFB2
1:218530561_GA_G	1	218,530,561	GA	G	0.88	0.751	TGFB2
rs2796825	1	218,531,768	T	A	0.142	0.942	TGFB2
rs184695731	1	218,531,913	C	G	0.985	0.937	TGFB2
rs2796824	1	218,531,914	A	G	0.257	0.931	TGFB2
1:218532315_TGGTCAAGGAACGGTCCCCCCAG_T	1	218,532,315	TGGTCAAGGAACGGTCCCCCCAG	T	0.989	0.919	TGFB2
1:218532318_TCAAGGAACGGTCCCCCCAGAGG_T	1	218,532,318	TCAAGGAACGGTCCCCCCAGAGG	T	0.989	0.919	TGFB2
rs141573895	1	218,534,855	G	A	0.978	0.903	TGFB2
rs10482741	1	218,536,350	T	C	0.988	0.854	TGFB2
rs12078363	1	218,537,310	T	C	0.68	0.995	TGFB2
rs183677281	1	218,537,632	T	C	0.975	0.912	TGFB2
rs4846266	1	218,541,707	A	G	0.679	0.999	TGFB2
rs61823391	1	218,541,992	T	C	0.682	0.998	TGFB2
rs12088448	1	218,546,170	A	C	0.643	0.989	TGFB2
1:218546226_GAA_G	1	218,546,226	GAA	G	0.944	0.879	TGFB2
rs149269977	1	218,546,474	C	T	0.976	0.951	TGFB2
rs10482744	1	218,546,969	A	G	0.989	0.86	TGFB2
rs17558745	1	218,548,521	C	T	0.688	0.989	TGFB2
rs549035841	1	218,548,749	C	CT	0.708	0.93	TGFB2
rs567928499	1	218,548,780	T	TA	0.469	0.898	TGFB2
rs35981664	1	218,549,354	A	T	0.688	0.977	TGFB2
rs2796817	1	218,551,008	T	G	0.865	0.877	TGFB2
rs13374308	1	218,551,463	C	A	0.926	0.9	TGFB2
rs7546539	1	218,551,587	T	C	0.366	0.853	TGFB2
rs7546632	1	218,551,698	T	C	0.925	0.897	TGFB2
rs148503993	1	218,551,726	C	G	0.908	0.863	TGFB2
rs113772426	1	218,551,774	C	A	0.775	0.927	TGFB2
rs2799084	1	218,552,078	C	T	0.483	0.888	TGFB2
rs541320667	1	218,552,115	G	GT	0.442	0.667	TGFB2
rs2799085	1	218,552,455	A	C	0.368	0.942	TGFB2
rs59028457	1	218,552,472	C	T	0.368	0.907	TGFB2
rs199959478	1	218,552,476	T	C	0.809	0.935	TGFB2
rs144784743	1	218,552,535	T	C	0.846	0.803	TGFB2
rs12066658	1	218,552,543	C	T	0.55	0.854	TGFB2
rs139371558	1	218,552,570	G	C	0.81	0.948	TGFB2
rs11118093	1	218,552,584	C	G	0.797	0.824	TGFB2
rs61823393	1	218,552,666	A	G	0.808	0.875	TGFB2
rs10863394	1	218,552,725	C	T	0.173	0.941	TGFB2
rs10863395	1	218,552,729	G	A	0.173	0.941	TGFB2
rs115601903	1	218,552,772	G	A	0.963	0.926	TGFB2
rs375611057	1	218,552,829	C	CTT	0.427	0.855	TGFB2
rs2009112	1	218,553,529	C	T	0.58	0.928	TGFB2
rs563359869	1	218,554,600	G	GT	0.674	0.858	TGFB2
1:218554632_CTG_C	1	218,554,632	CTG	C	0.973	0.968	TGFB2
rs57618228	1	218,554,832	G	T	0.833	0.892	TGFB2
rs10482750	1	218,556,036	T	C	0.898	0.962	TGFB2
rs2799086	1	218,556,202	C	T	0.867	0.982	TGFB2
rs10482751	1	218,556,297	T	C	0.287	0.986	TGFB2
1:218556719_CT_C	1	218,556,719	CT	C	0.968	0.995	TGFB2
rs77307356	1	218,557,279	G	T	0.974	0.998	TGFB2
1:218557465_GA_G	1	218,557,465	GA	G	0.346	0.887	TGFB2
rs4335431	1	218,557,609	T	C	0.923	0.993	TGFB2
rs74143054	1	218,557,907	C	T	0.969	0.999	TGFB2
rs139995755	1	218,558,185	G	A	0.984	0.883	TGFB2
rs2027566	1	218,558,327	C	A	0.319	0.998	TGFB2
rs11466381	1	218,558,445	A	G	0.922	0.996	TGFB2

SNP	Chromosome	Position	Effect Allele	Other Allele	Effect Allele Frequency	Imputation quality	Gene
rs2027567	1	218,558,588	G	A	0.22	0.997	TGFB2
rs12029576	1	218,559,475	C	A	0.288	0.998	TGFB2
rs4846267	1	218,560,165	C	T	0.287	0.983	TGFB2
rs2796814	1	218,561,581	C	G	0.777	0.923	TGFB2
1:218561669_GC_G	1	218,561,669	GC	G	0.979	0.832	TGFB2
rs79731206	1	218,562,558	A	G	0.985	0.963	TGFB2
rs7536600	1	218,563,036	C	T	0.946	0.996	TGFB2
rs12129220	1	218,563,693	C	A	0.87	0.986	TGFB2
rs947712	1	218,564,881	C	T	0.661	0.964	TGFB2
rs947711	1	218,564,970	C	G	0.737	0.961	TGFB2
rs6656048	1	218,564,977	T	C	0.946	0.999	TGFB2
rs1539399	1	218,565,324	G	A	0.661	0.964	TGFB2
rs185779148	1	218,565,650	A	G	0.98	0.869	TGFB2
rs191080405	1	218,565,651	T	C	0.98	0.869	TGFB2
rs149147748	1	218,567,442	G	A	0.962	0.952	TGFB2
rs2799089	1	218,567,494	G	A	0.663	0.943	TGFB2
rs76916003	1	218,568,359	G	A	0.956	0.909	TGFB2
rs6604608	1	218,568,473	G	A	0.946	1	TGFB2
rs6604609	1	218,568,482	T	A	0.946	1	TGFB2
rs2799090	1	218,568,829	A	G	0.739	0.964	TGFB2
rs2799091	1	218,569,027	C	A	0.809	0.956	TGFB2
rs113038979	1	218,569,114	T	C	0.946	1	TGFB2
rs189869614	1	218,569,158	A	G	0.981	0.945	TGFB2
rs111305122	1	218,569,208	G	A	0.946	1	TGFB2
rs11118099	1	218,569,247	C	T	0.85	0.951	TGFB2
rs13375381	1	218,570,470	C	G	0.984	0.982	TGFB2
rs138400337	1	218,570,643	T	G	0.847	0.991	TGFB2
rs77795240	1	218,571,561	G	T	0.959	0.977	TGFB2
rs61823428	1	218,571,757	A	T	0.87	0.995	TGFB2
rs11118102	1	218,572,004	G	A	0.87	0.995	TGFB2
rs76880472	1	218,572,580	C	G	0.864	0.947	TGFB2
rs7517802	1	218,572,768	T	G	0.946	1	TGFB2
rs10779329	1	218,573,741	T	C	0.776	0.995	TGFB2
rs11401661	1	218,574,584	A	AT	0.947	0.999	TGFB2
rs1317681	1	218,575,202	G	A	0.846	0.996	TGFB2
rs2796812	1	218,575,472	A	G	0.584	0.974	TGFB2
1:218576549_GT_G	1	218,576,549	GT	G	0.946	0.996	TGFB2
rs6688036	1	218,577,295	G	C	0.928	0.995	TGFB2
rs767792778	1	218,577,393	CA	C	0.329	0.806	TGFB2
rs11118103	1	218,579,644	G	A	0.927	0.997	TGFB2
rs150055803	1	218,579,680	A	AT	0.927	0.996	TGFB2
rs10482771	1	218,579,751	G	A	0.928	0.998	TGFB2
1:218579900_TTAAATACA_T	1	218,579,900	TTAAATACA	T	0.968	0.97	TGFB2
rs2796819	1	218,580,370	A	G	0.584	0.976	TGFB2
rs10482775	1	218,580,459	A	T	0.944	0.998	TGFB2
rs75114228	1	218,580,516	T	C	0.958	0.922	TGFB2
rs79017906	1	218,581,102	G	T	0.944	0.996	TGFB2
rs28540714	1	218,581,430	A	T	0.984	0.989	TGFB2
rs2796820	1	218,581,447	T	G	0.791	0.969	TGFB2
rs1797071	1	218,581,509	T	C	0.664	0.975	TGFB2
rs12035819	1	218,581,595	A	G	0.944	0.996	TGFB2
rs2799083	1	218,581,617	T	C	0.736	0.973	TGFB2
rs17047804	1	218,582,778	T	C	0.89	0.995	TGFB2
1:218583007_GA_G	1	218,583,007	GA	G	0.79	0.945	TGFB2
rs142586277	1	218,584,375	A	ATCC	0.834	0.94	TGFB2
rs2799103	1	218,584,419	C	T	0.791	0.97	TGFB2
rs2799102	1	218,584,952	A	C	0.719	0.974	TGFB2
rs143126125	1	218,585,154	C	CAGAG	0.944	0.988	TGFB2
rs10863397	1	218,585,248	C	T	0.944	0.995	TGFB2
rs2796821	1	218,585,821	C	T	0.736	0.974	TGFB2
rs2796822	1	218,586,132	A	G	0.609	0.977	TGFB2
rs2796823	1	218,586,214	A	T	0.736	0.975	TGFB2
rs11466397	1	218,586,522	T	C	0.944	0.995	TGFB2
rs566215021	1	218,587,569	G	GCA	0.949	0.823	TGFB2
rs796922311	1	218,587,570	C	CAC	0.928	0.771	TGFB2
rs2799095	1	218,587,571	A	G	0.781	0.939	TGFB2
rs12406538	1	218,587,573	A	G	0.834	0.936	TGFB2
rs2000220	1	218,587,636	A	G	0.609	0.978	TGFB2
rs748476023	1	218,588,138	CATGCAG	C	0.736	0.959	TGFB2
rs10863398	1	218,588,279	G	A	0.889	0.994	TGFB2
rs10482777	1	218,588,968	C	G	0.984	0.989	TGFB2
1:218589039_AAG_A	1	218,589,039	AAG	A	0.722	0.822	TGFB2
rs2799094	1	218,589,041	G	A	0.724	0.693	TGFB2
rs2796813	1	218,589,197	T	C	0.587	0.978	TGFB2
1:218589466_TG_T	1	218,589,466	TG	T	0.792	0.955	TGFB2
rs541029920	1	218,589,634	C	CT	0.891	0.753	TGFB2
rs2096038	1	218,590,155	A	G	0.79	0.971	TGFB2
rs12128032	1	218,590,655	G	A	0.794	0.975	TGFB2
rs11466399	1	218,591,623	G	A	0.714	0.996	TGFB2
rs12405805	1	218,592,115	G	A	0.945	0.997	TGFB2
rs143136585	1	218,592,160	G	T	0.984	0.995	TGFB2
rs12048582	1	218,593,037	C	T	0.784	0.997	TGFB2
rs181424343	1	218,594,484	G	A	0.99	0.946	TGFB2
rs79285949	1	218,594,588	T	C	0.984	0.998	TGFB2
rs796123246	1	218,594,715	GT	G	0.776	0.982	TGFB2
rs200128203	1	218,594,719	T	G	0.776	0.982	TGFB2
rs57316043	1	218,594,748	A	AT	0.945	0.997	TGFB2
rs1934852	1	218,594,920	G	T	0.713	0.999	TGFB2
1:218594995_TA_T	1	218,594,995	TA	T	0.984	0.998	TGFB2
rs6657275	1	218,596,461	A	G	0.713	0.999	TGFB2
rs56141119	1	218,596,619	A	T	0.979	0.922	TGFB2
rs6668651	1	218,596,641	G	T	0.713	0.999	TGFB2
rs116437503	1	218,596,935	A	G	0.988	0.939	TGFB2
rs6671370	1	218,597,044	G	A	0.713	0.999	TGFB2
rs12048049	1	218,597,297	C	G	0.713	0.999	TGFB2
rs72738830	1	218,597,632	G	A	0.784	0.999	TGFB2
rs1342586	1	218,597,859	C	T	0.784	0.999	TGFB2
rs4846478	1	218,598,328	C	G	0.729	0.999	TGFB2
rs4846479	1	218,598,410	G	T	0.729	1	TGFB2
rs4846480	1	218,598,469	A	T	0.729	1	TGFB2
rs4846481	1	218,598,484	C	T	0.713	1	TGFB2
rs11466401	1	218,598,674	C	G	0.984	0.999	TGFB2
rs11466404	1	218,599,115	G	A	0.984	0.83	TGFB2
rs6658473	1	218,600,419	C	T	0.729	1	TGFB2
1:218601905_GTT_G	1	218,601,905	GTT	G	0.797	0.96	TGFB2
rs12408756	1	218,602,134	T	C	0.793	0.98	TGFB2
rs145699773	1	218,602,571	C	A	0.982	0.999	TGFB2
rs150121444	1	218,602,948	A	AT	0.971	1	TGFB2
rs7554934	1	218,604,392	C	A	0.945	0.999	TGFB2
rs1890995	1	218,604,678	G	A	0.729	1	TGFB2
rs1890994	1	218,604,709	C	T	0.785	0.999	TGFB2
rs10482791	1	218,605,305	T	C	0.945	0.999	TGFB2
rs10482792	1	218,605,461	G	A	0.729	1	TGFB2
rs10482795	1	218,605,609	G	A	0.714	1	TGFB2
rs10482796	1	218,605,635	T	C	0.784	0.999	TGFB2
rs9308385	1	218,605,956	T	C	0.785	0.999	TGFB2
rs10482803	1	218,606,468	T	G	0.984	0.999	TGFB2
rs10482807	1	218,606,948	G	A	0.961	0.934	TGFB2
rs10482819	1	218,609,736	G	A	0.948	0.975	TGFB2
rs1418553	1	218,610,254	C	T	0.714	1	TGFB2
rs6678287	1	218,611,475	C	G	0.945	0.999	TGFB2
rs2798631	1	218,611,878	A	G	0.507	0.987	TGFB2
rs10482827	1	218,612,012	C	T	0.981	0.962	TGFB2
rs10482830	1	218,612,277	C	T	0.984	0.999	TGFB2
1:218612449_CAA_C	1	218,612,449	CAA	C	0.472	0.954	TGFB2
rs10482833	1	218,612,625	G	A	0.956	0.973	TGFB2
1:218613687_CT_C	1	218,613,687	CT	C	0.709	0.982	TGFB2
rs10482837	1	218,614,266	G	GA	0.945	0.998	TGFB2
rs11466412	1	218,614,743	G	A	0.984	0.999	TGFB2
rs900	1	218,614,905	A	T	0.73	1	TGFB2
rs991967	1	218,615,451	A	C	0.714	1	TGFB2
1:218615492_GT_G	1	218,615,492	GT	G	0.973	0.874	TGFB2
rs1418556	1	218,616,014	C	T	0.72	0.983	TGFB2
rs17500758	1	218,616,206	T	C	0.948	0.974	TGFB2
rs3737977	1	218,616,328	T	A	0.945	0.999	TGFB2
rs17047869	1	218,616,892	T	C	0.984	0.999	TGFB2
1:218617051_GTT_G	1	218,617,051	GTT	G	0.725	0.983	TGFB2
rs1046017	1	218,617,135	C	G	0.714	1	TGFB2
rs77808075	1	218,617,268	A	G	0.984	0.999	TGFB2
rs6704255	1	218,617,543	G	A	0.714	0.999	TGFB2
rs6683598	1	218,617,900	C	T	0.714	0.999	TGFB2
rs12725747	1	228,106,921	G	A	0.88	0.997	WNT9A
rs4266877	1	228,107,031	G	A	0.805	0.993	WNT9A
rs12726048	1	228,107,136	G	A	0.88	0.997	WNT9A
rs4233231	1	228,107,139	A	G	0.409	0.997	WNT9A
rs3820623	1	228,107,262	G	A	0.804	0.993	WNT9A
rs3820622	1	228,107,281	G	A	0.409	0.996	WNT9A
rs3820621	1	228,107,365	A	G	0.282	0.995	WNT9A
1:228107387_TAC_T	1	228,107,387	TAC	T	0.462	0.889	WNT9A
rs77824589	1	228,107,421	C	G	0.893	0.791	WNT9A
rs79735944	1	228,107,423	C	G	0.75	0.895	WNT9A
rs12040881	1	228,107,430	T	C	0.596	0.994	WNT9A
1:228107487_GCACTCACAA_G	1	228,107,487	GCACTCACAA	G	0.806	0.99	WNT9A
rs71559823	1	228,107,554	A	AACAC	0.604	0.99	WNT9A
rs3856330	1	228,107,643	T	C	0.29	0.994	WNT9A

SNP	Chromosome	Position	Effect Allele	Other Allele	Effect Allele Frequency	Imputation quality	Gene
1:228108135_GA_G	1	228,108,135	GA	G	0.806	0.989	WNT9A
rs61825078	1	228,108,290	G	A	0.978	0.927	WNT9A
rs34253719	1	228,108,584	G	T	0.88	1	WNT9A
rs10916244	1	228,108,703	C	T	0.611	0.991	WNT9A
rs3795767	1	228,108,932	A	G	0.409	0.994	WNT9A
rs35718701	1	228,109,137	A	G	0.88	0.998	WNT9A
rs8192633	1	228,109,471	C	T	0.977	0.94	WNT9A
rs114666290	1	228,110,063	C	G	0.979	0.914	WNT9A
rs10127943	1	228,110,064	G	A	0.68	0.97	WNT9A
rs144237932	1	228,110,290	G	A	0.977	0.814	WNT9A
rs8192630	1	228,112,034	C	T	0.988	0.883	WNT9A
rs4653889	1	228,112,121	A	G	0.467	0.987	WNT9A
rs10916245	1	228,112,280	T	C	0.471	0.98	WNT9A
rs1418422	1	228,112,465	A	G	0.571	0.986	WNT9A
rs28665607	1	228,112,716	G	A	0.916	0.976	WNT9A
rs115903339	1	228,112,732	G	C	0.883	0.972	WNT9A
rs8192629	1	228,113,046	C	T	0.896	0.989	WNT9A
rs3795768	1	228,113,163	A	G	0.294	0.982	WNT9A
rs74140812	1	228,113,436	T	C	0.895	0.989	WNT9A
rs74140813	1	228,114,294	C	A	0.89	0.992	WNT9A
rs72754204	1	228,114,402	C	T	0.959	0.973	WNT9A
rs74140814	1	228,114,406	A	C	0.89	0.993	WNT9A
rs621631	1	228,114,728	A	G	0.466	0.995	WNT9A
rs640440	1	228,115,006	G	T	0.575	0.994	WNT9A
rs3949294	1	228,115,129	G	T	0.899	0.994	WNT9A
rs71650368	1	228,115,172	G	A	0.948	0.985	WNT9A
rs74425643	1	228,115,190	G	A	0.899	0.994	WNT9A
rs116216380	1	228,115,191	G	C	0.988	0.932	WNT9A
rs1774755	1	228,115,282	T	G	0.466	0.996	WNT9A
rs1774754	1	228,115,299	C	G	0.565	0.993	WNT9A
rs1636194	1	228,115,309	G	T	0.575	0.992	WNT9A
rs79348703	1	228,115,368	C	T	0.899	0.994	WNT9A
rs1636193	1	228,115,393	G	A	0.567	0.995	WNT9A
rs605410	1	228,116,281	A	G	0.466	0.997	WNT9A
rs592736	1	228,116,831	T	C	0.466	0.996	WNT9A
rs591582	1	228,117,360	C	A	0.466	0.997	WNT9A
rs590657	1	228,117,581	A	C	0.466	0.997	WNT9A
rs596675	1	228,117,892	G	A	0.466	0.997	WNT9A
rs596208	1	228,117,982	G	C	0.466	0.997	WNT9A
rs2313118	1	228,118,084	T	C	0.466	0.997	WNT9A
rs3901230	1	228,118,177	G	T	0.937	0.961	WNT9A
rs2527616	1	228,118,469	C	A	0.466	0.997	WNT9A
rs528283186	1	228,118,700	C	CA	0.55	0.883	WNT9A
rs2485254	1	228,118,829	C	T	0.577	0.996	WNT9A
rs2796055	1	228,118,934	A	T	0.577	0.996	WNT9A
rs36104676	1	228,119,269	T	C	0.982	0.951	WNT9A
rs2527615	1	228,119,283	T	C	0.466	0.998	WNT9A
rs536514624	1	228,119,581	C	T	0.761	0.722	WNT9A
rs777403846	1	228,119,581	CT	C	0.712	0.761	WNT9A
rs686533	1	228,119,604	A	G	0.474	0.997	WNT9A
rs4399155	1	228,120,050	G	A	0.889	0.995	WNT9A
rs673726	1	228,120,173	T	C	0.466	0.998	WNT9A
rs113765760	1	228,120,224	A	G	0.946	0.98	WNT9A
rs672977	1	228,120,288	A	C	0.466	0.998	WNT9A
rs672907	1	228,120,345	G	A	0.466	0.998	WNT9A
rs658238	1	228,121,291	A	G	0.466	0.998	WNT9A
rs634106	1	228,121,548	C	T	0.474	0.998	WNT9A
rs33928676	1	228,121,574	C	CT	0.466	0.997	WNT9A
rs545753679	1	228,121,664	T	G	0.985	0.62	WNT9A
rs632832	1	228,121,838	C	A	0.465	0.998	WNT9A
rs61825079	1	228,122,469	G	C	0.984	0.971	WNT9A
1:228122740_CAG_C	1	228,122,740	CAG	C	0.791	0.973	WNT9A
rs2796056	1	228,122,901	G	A	0.466	0.999	WNT9A
rs144163823	1	228,123,781	C	A	0.975	0.95	WNT9A
rs538972408	1	228,123,897	C	CT	0.948	0.879	WNT9A
rs621963	1	228,124,214	T	C	0.466	0.999	WNT9A
rs556507647	1	228,124,335	C	CTT	0.47	0.988	WNT9A
rs2796057	1	228,124,414	G	C	0.465	0.999	WNT9A
rs1636192	1	228,124,688	A	G	0.465	0.999	WNT9A
rs112310460	1	228,124,767	G	A	0.981	0.903	WNT9A
rs80096292	1	228,125,436	G	T	0.901	0.997	WNT9A
rs680997	1	228,127,227	T	C	0.053	0.963	WNT9A
rs680148	1	228,127,391	G	A	0.577	0.998	WNT9A
rs631768	1	228,129,197	T	C	0.408	0.989	WNT9A
rs638877	1	228,130,579	T	C	0.565	0.999	WNT9A
rs143134076	1	228,130,691	G	GC	0.902	0.996	WNT9A
rs587000	1	228,132,290	C	T	0.576	0.998	WNT9A
rs35799012	1	228,133,322	C	T	0.828	0.993	WNT9A
rs594916	1	228,133,544	C	A	0.466	0.999	WNT9A
rs144748024	1	228,134,977	A	C	0.983	0.945	WNT9A
rs201286255	2	85,360,654	G	A	0.971	0.976	TCF7L1
rs181491150	2	85,360,763	C	A	0.928	0.976	TCF7L1
rs769216273	2	85,360,828	GGGC	G	0.59	0.975	TCF7L1
rs571356698	2	85,361,361	T	G	0.985	0.661	TCF7L1
rs77668226	2	85,361,852	G	A	0.939	0.981	TCF7L1
rs140057681	2	85,362,825	G	C	0.986	0.963	TCF7L1
rs70953928	2	85,362,913	A	AGGTC	0.644	0.984	TCF7L1
rs13003453	2	85,362,933	T	G	0.9	0.985	TCF7L1
2:85363054_AGG_A	2	85,363,054	AGG	A	0.399	0.914	TCF7L1
2:85363064_GGA_G	2	85,363,064	GGA	G	0.678	0.883	TCF7L1
rs72838188	2	85,364,138	C	T	0.959	0.98	TCF7L1
rs72838189	2	85,364,307	T	G	0.742	0.998	TCF7L1
rs12474138	2	85,364,537	G	A	0.889	0.975	TCF7L1
rs55984939	2	85,364,689	T	C	0.589	0.986	TCF7L1
rs76710632	2	85,364,753	G	C	0.742	0.997	TCF7L1
rs62162824	2	85,364,769	A	G	0.919	0.975	TCF7L1
rs111462901	2	85,364,805	C	T	0.742	0.996	TCF7L1
rs7584403	2	85,365,367	C	T	0.742	0.994	TCF7L1
rs1835812	2	85,365,498	A	G	0.487	0.98	TCF7L1
rs62162825	2	85,365,569	G	A	0.919	0.974	TCF7L1
rs72838190	2	85,365,711	C	T	0.742	0.992	TCF7L1
rs145488973	2	85,366,826	C	G	0.983	0.865	TCF7L1
rs6754757	2	85,366,971	T	G	0.583	0.971	TCF7L1
rs56168805	2	85,366,987	T	G	0.878	0.973	TCF7L1
rs56213912	2	85,367,081	G	A	0.879	0.972	TCF7L1
rs55974301	2	85,367,278	C	T	0.966	0.973	TCF7L1
rs560846484	2	85,367,595	C	CTTTCT	0.401	0.969	TCF7L1
rs5832634	2	85,367,603	T	TC	0.4	0.969	TCF7L1
2:85367742_TG_T	2	85,367,742	TG	T	0.224	0.965	TCF7L1
rs62162827	2	85,367,808	G	A	0.889	0.978	TCF7L1
rs1807310	2	85,367,855	G	A	0.512	0.98	TCF7L1
rs71245379	2	85,368,036	C	CAGAG	0.438	0.97	TCF7L1
rs62162828	2	85,368,090	G	C	0.889	0.979	TCF7L1
rs62162830	2	85,368,328	A	T	0.924	0.982	TCF7L1
rs10652685	2	85,368,343	C	CAG	0.327	0.97	TCF7L1
rs57097797	2	85,368,748	A	T	0.925	0.984	TCF7L1
rs72838192	2	85,369,260	C	T	0.963	0.963	TCF7L1
rs2583566	2	85,369,381	T	C	0.439	0.986	TCF7L1
rs75632482	2	85,369,605	A	T	0.925	0.984	TCF7L1
rs578099999	2	85,369,620	T	C	0.988	0.869	TCF7L1
rs17762108	2	85,370,407	T	C	0.925	0.993	TCF7L1
2:85370565_TA_T	2	85,370,565	TA	T	0.965	0.972	TCF7L1
rs78514033	2	85,371,442	G	A	0.984	0.887	TCF7L1
2:85371540_CA_C	2	85,371,540	CA	C	0.442	0.932	TCF7L1
rs199583204	2	85,371,838	C	G	0.639	0.842	TCF7L1
rs71337779	2	85,371,840	C	G	0.879	0.582	TCF7L1
rs59412890	2	85,371,842	C	G	0.506	0.886	TCF7L1
rs6759827	2	85,371,844	G	C	0.943	0.88	TCF7L1
rs56311108	2	85,371,870	G	T	0.493	0.965	TCF7L1
rs767439897	2	85,371,872	GTA	G	0.493	0.965	TCF7L1
rs145487523	2	85,371,939	G	A	0.946	0.998	TCF7L1
rs190871356	2	85,371,977	C	T	0.976	0.948	TCF7L1
rs2568216	2	85,372,094	G	A	0.473	0.999	TCF7L1
rs2583541	2	85,372,139	C	T	0.473	0.998	TCF7L1
rs72838194	2	85,373,007	A	T	0.965	0.984	TCF7L1
rs113082906	2	85,373,615	T	C	0.925	0.996	TCF7L1
2:85373620_CTTT_C	2	85,373,620	CTTT	C	0.467	0.974	TCF7L1
rs1808640	2	85,373,749	T	C	0.363	0.994	TCF7L1
rs1808639	2	85,373,817	A	G	0.473	1	TCF7L1
rs151147084	2	85,373,827	G	A	0.965	0.984	TCF7L1
rs72838196	2	85,373,976	C	T	0.965	0.984	TCF7L1
2:85374261_GATAA_G	2	85,374,261	GATAA	G	0.896	0.998	TCF7L1
rs2248068	2	85,374,315	A	C	0.473	1	TCF7L1
rs2248072	2	85,374,417	G	A	0.473	1	TCF7L1
rs2248075	2	85,374,480	C	T	0.363	0.997	TCF7L1
rs2248076	2	85,374,482	A	G	0.363	0.997	TCF7L1
rs2248079	2	85,374,526	A	G	0.473	0.999	TCF7L1
rs4832144	2	85,374,778	C	T	0.96	0.997	TCF7L1
rs2568217	2	85,375,001	T	C	0.363	0.997	TCF7L1
rs2568218	2	85,375,132	T	G	0.363	0.997	TCF7L1
rs17025940	2	85,375,282	G	A	0.925	0.998	TCF7L1
rs2568219	2	85,375,574	G	A	0.473	0.999	TCF7L1
rs114507663	2	85,375,596	A	T	0.949	0.914	TCF7L1
rs6754217	2	85,375,676	C	T	0.925	0.998	TCF7L1
rs181710737	2	85,375,990	C	G	0.979	0.915	TCF7L1

SNP	Chromosome	Position	Effect Allele	Other Allele	Effect Allele Frequency	Imputation quality	Gene
rs2436192	2	85,376,121	T	C	0.363	0.994	TCF7L1
rs55886208	2	85,376,194	G	A	0.925	0.998	TCF7L1
rs557122754	2	85,376,449	C	CAT	0.463	0.98	TCF7L1
rs142535507	2	85,376,940	G	A	0.985	0.98	TCF7L1
rs35035277	2	85,376,970	T	C	0.896	0.997	TCF7L1
rs72838199	2	85,377,070	G	A	0.965	0.983	TCF7L1
rs7570315	2	85,377,179	T	C	0.925	0.998	TCF7L1
rs2568221	2	85,377,576	C	T	0.485	0.998	TCF7L1
rs75470307	2	85,377,810	T	C	0.96	0.998	TCF7L1
rs2568222	2	85,377,979	A	G	0.484	0.998	TCF7L1
rs6723581	2	85,378,151	T	A	0.925	0.998	TCF7L1
rs72838202	2	85,378,248	T	A	0.965	0.983	TCF7L1
rs62162851	2	85,378,295	C	T	0.925	0.999	TCF7L1
rs2583540	2	85,378,663	T	A	0.484	0.998	TCF7L1
rs7560555	2	85,378,666	C	A	0.925	0.999	TCF7L1
rs2568223	2	85,378,821	C	G	0.374	0.995	TCF7L1
rs2583539	2	85,378,894	C	A	0.524	0.998	TCF7L1
rs34742498	2	85,379,560	C	T	0.445	0.903	TCF7L1
rs113537459	2	85,379,599	C	T	0.96	0.999	TCF7L1
rs2568224	2	85,379,656	C	G	0.409	0.997	TCF7L1
rs62162852	2	85,379,729	C	T	0.849	0.994	TCF7L1
rs2568225	2	85,379,914	T	C	0.485	0.997	TCF7L1
2:85380315_AG_A	2	85,380,315	AG	A	0.989	0.903	TCF7L1
rs55733107	2	85,380,504	A	G	0.965	0.97	TCF7L1
rs79983032	2	85,380,847	A	T	0.966	0.995	TCF7L1
rs17762406	2	85,381,416	T	C	0.896	0.994	TCF7L1
rs72840107	2	85,381,467	A	G	0.965	0.982	TCF7L1
rs72840108	2	85,381,994	C	T	0.965	0.97	TCF7L1
rs138315854	2	85,382,011	C	G	0.965	0.972	TCF7L1
rs2568226	2	85,382,730	A	T	0.529	0.979	TCF7L1
2:85383540_AG_A	2	85,383,540	AG	A	0.944	0.949	TCF7L1
rs55880914	2	85,383,913	T	C	0.965	0.961	TCF7L1
rs75438253	2	85,384,179	C	T	0.961	0.967	TCF7L1
rs72840110	2	85,384,953	C	A	0.893	0.929	TCF7L1
rs62162853	2	85,385,438	C	T	0.932	0.943	TCF7L1
rs62162854	2	85,387,493	A	G	0.695	0.961	TCF7L1
rs72840111	2	85,387,505	G	A	0.962	0.973	TCF7L1
rs147704304	2	85,387,952	G	A	0.961	0.979	TCF7L1
rs11679513	2	85,388,186	G	A	0.928	0.936	TCF7L1
rs77707396	2	85,388,993	C	T	0.939	0.992	TCF7L1
rs59117861	2	85,389,168	A	G	0.953	0.975	TCF7L1
rs72840112	2	85,389,180	T	A	0.963	0.951	TCF7L1
rs74504675	2	85,389,185	A	G	0.94	0.995	TCF7L1
rs34891553	2	85,389,297	G	T	0.978	0.959	TCF7L1
rs6547596	2	85,389,305	G	C	0.953	0.979	TCF7L1
rs6547597	2	85,389,685	T	C	0.953	0.98	TCF7L1
rs1560586	2	85,389,714	T	A	0.94	0.999	TCF7L1
rs72932658	2	85,389,975	T	C	0.954	0.982	TCF7L1
rs55801685	2	85,390,041	C	A	0.914	0.989	TCF7L1
rs72840115	2	85,390,782	G	A	0.961	0.998	TCF7L1
rs10186759	2	85,391,255	C	T	0.898	0.991	TCF7L1
rs112013331	2	85,391,286	C	T	0.985	0.915	TCF7L1
rs66856432	2	85,391,308	G	A	0.915	0.991	TCF7L1
rs76117226	2	85,391,375	G	A	0.956	0.993	TCF7L1
rs3943814	2	85,391,745	A	T	0.94	0.995	TCF7L1
rs11684525	2	85,391,875	G	A	0.71	0.971	TCF7L1
rs72932662	2	85,392,009	T	C	0.896	0.993	TCF7L1
rs1560587	2	85,392,093	A	T	0.375	0.966	TCF7L1
rs72840116	2	85,392,111	T	C	0.944	0.995	TCF7L1
rs72840118	2	85,392,199	C	T	0.897	0.993	TCF7L1
rs11691660	2	85,392,216	T	G	0.606	0.975	TCF7L1
rs762968967	2	85,392,485	AACAAGC	A	0.839	0.928	TCF7L1
rs62162858	2	85,392,494	A	T	0.831	0.959	TCF7L1
rs75241717	2	85,392,975	A	G	0.941	0.992	TCF7L1
rs56262531	2	85,393,761	A	G	0.95	0.991	TCF7L1
rs17762507	2	85,393,984	G	C	0.94	0.991	TCF7L1
rs56270929	2	85,394,082	C	T	0.946	0.998	TCF7L1
rs137882758	2	85,394,111	T	C	0.954	0.994	TCF7L1
rs11686370	2	85,394,114	T	C	0.575	0.985	TCF7L1
rs2583555	2	85,394,146	C	T	0.881	0.959	TCF7L1
rs114664588	2	85,394,291	C	T	0.971	0.948	TCF7L1
rs13398222	2	85,394,572	C	G	0.895	0.991	TCF7L1
rs11691849	2	85,394,611	A	G	0.63	0.985	TCF7L1
rs11682047	2	85,395,104	C	T	0.638	0.986	TCF7L1
rs35369436	2	85,395,205	T	A	0.972	0.951	TCF7L1
rs11693369	2	85,395,357	A	T	0.578	0.987	TCF7L1
rs77433347	2	85,396,058	G	T	0.94	0.988	TCF7L1
rs74457100	2	85,396,147	A	G	0.947	0.978	TCF7L1
rs75450748	2	85,396,267	G	A	0.955	0.992	TCF7L1
rs11126981	2	85,396,407	C	A	0.69	0.986	TCF7L1
rs144738899	2	85,398,277	G	A	0.955	0.991	TCF7L1
rs17711688	2	85,399,316	C	A	0.962	0.975	TCF7L1
rs79222457	2	85,399,367	C	T	0.962	0.973	TCF7L1
rs151040069	2	85,400,062	G	A	0.983	0.934	TCF7L1
rs4832145	2	85,400,584	C	T	0.986	0.916	TCF7L1
rs115684026	2	85,400,585	G	A	0.959	0.975	TCF7L1
rs34729115	2	85,400,866	C	CT	0.697	0.98	TCF7L1
rs2583542	2	85,400,977	G	A	0.698	0.982	TCF7L1
rs142717815	2	85,401,100	C	T	0.948	0.986	TCF7L1
rs115237876	2	85,401,478	A	C	0.988	0.927	TCF7L1
rs2568197	2	85,401,958	C	T	0.487	0.989	TCF7L1
rs17025989	2	85,402,493	C	T	0.963	0.989	TCF7L1
rs150110748	2	85,402,644	G	T	0.985	0.935	TCF7L1
rs72840119	2	85,402,814	T	C	0.948	0.99	TCF7L1
rs2583543	2	85,402,974	A	C	0.638	0.99	TCF7L1
rs76415850	2	85,403,244	G	T	0.976	0.926	TCF7L1
rs2568198	2	85,403,546	A	G	0.639	0.99	TCF7L1
rs148134106	2	85,404,024	C	T	0.984	0.978	TCF7L1
rs2568199	2	85,404,622	A	G	0.188	0.962	TCF7L1
rs2568200	2	85,405,032	A	G	0.638	0.99	TCF7L1
rs1864804	2	85,405,262	G	A	0.907	0.978	TCF7L1
rs142192858	2	85,405,776	A	G	0.988	0.896	TCF7L1
rs78183773	2	85,406,122	C	T	0.986	0.911	TCF7L1
rs11401926	2	85,406,860	C	CT	0.963	0.981	TCF7L1
rs113353353	2	85,407,570	G	A	0.963	0.989	TCF7L1
rs7594695	2	85,408,298	G	A	0.911	0.99	TCF7L1
rs7594708	2	85,408,364	G	A	0.963	0.989	TCF7L1
rs2568201	2	85,408,551	A	G	0.601	0.991	TCF7L1
rs62162859	2	85,408,759	G	A	0.981	0.98	TCF7L1
rs72840120	2	85,408,966	G	A	0.911	0.99	TCF7L1
rs78109461	2	85,409,060	T	C	0.963	0.989	TCF7L1
rs147597376	2	85,409,239	G	A	0.976	0.97	TCF7L1
rs76050564	2	85,409,300	G	A	0.963	0.989	TCF7L1
rs77708381	2	85,409,359	C	T	0.963	0.989	TCF7L1
rs2583545	2	85,410,089	G	A	0.602	0.992	TCF7L1
rs10167557	2	85,410,104	G	A	0.883	0.991	TCF7L1
rs2568203	2	85,410,933	C	T	0.692	0.993	TCF7L1
rs6758096	2	85,411,399	C	T	0.963	0.99	TCF7L1
rs11126982	2	85,412,776	T	C	0.958	0.983	TCF7L1
rs2568204	2	85,413,068	G	A	0.586	0.994	TCF7L1
rs61342907	2	85,413,175	T	C	0.894	0.996	TCF7L1
rs143738779	2	85,413,566	C	CT	0.894	0.993	TCF7L1
rs2583546	2	85,413,663	T	C	0.521	0.997	TCF7L1
rs72840121	2	85,413,898	T	C	0.964	0.988	TCF7L1
rs2568205	2	85,413,972	T	C	0.68	1	TCF7L1
rs2583547	2	85,414,140	G	A	0.479	0.996	TCF7L1
rs13432342	2	85,414,647	G	A	0.894	0.998	TCF7L1
rs72840124	2	85,415,238	C	T	0.99	0.91	TCF7L1
rs2568207	2	85,415,429	A	T	0.391	0.99	TCF7L1
rs74935588	2	85,415,435	C	A	0.907	0.979	TCF7L1
rs72840125	2	85,415,473	G	A	0.948	0.993	TCF7L1
2:85415938_CT_C	2	85,415,938	CT	C	0.948	0.99	TCF7L1
rs72840126	2	85,416,296	C	G	0.948	0.992	TCF7L1
rs72840127	2	85,418,491	G	A	0.964	0.956	TCF7L1
rs2583548	2	85,418,906	T	C	0.59	0.997	TCF7L1
rs2568208	2	85,419,524	T	A	0.643	0.997	TCF7L1
rs138580387	2	85,419,621	A	G	0.985	0.974	TCF7L1
rs200483452	2	85,419,647	A	AT	0.948	0.99	TCF7L1
rs2583549	2	85,419,784	G	T	0.68	0.998	TCF7L1
2:85419814_CT_C	2	85,419,814	CT	C	0.934	0.743	TCF7L1
rs1432264	2	85,419,900	T	C	0.194	0.969	TCF7L1
rs182387236	2	85,419,972	A	T	0.967	0.937	TCF7L1
2:85420052_TC_T	2	85,420,052	TC	T	0.984	0.97	TCF7L1
rs2568210	2	85,420,124	C	T	0.59	0.997	TCF7L1
rs2568211	2	85,420,482	A	G	0.484	0.997	TCF7L1
rs4374376	2	85,420,702	T	C	0.963	0.991	TCF7L1
rs880092	2	85,420,870	T	C	0.842	0.997	TCF7L1
rs56178814	2	85,421,017	T	G	0.948	0.994	TCF7L1
rs880093	2	85,421,092	A	T	0.895	0.993	TCF7L1
rs874838	2	85,421,691	A	G	0.894	0.999	TCF7L1
rs74556212	2	85,421,793	A	G	0.979	0.973	TCF7L1
rs753814	2	85,422,422	C	T	0.68	0.998	TCF7L1
rs2007125	2	85,422,818	C	T	0.574	0.998	TCF7L1
rs13423910	2	85,422,983	T	C	0.894	0.999	TCF7L1
rs747078	2	85,422,993	A	C	0.679	0.998	TCF7L1

SNP	Chromosome	Position	Effect Allele	Other Allele	Effect Allele Frequency	Imputation quality	Gene
rs115748171	2	85,423,054	C	T	0.976	0.977	TCF7L1
rs6728057	2	85,423,414	C	T	0.963	0.992	TCF7L1
rs140249057	2	85,423,756	G	T	0.987	0.915	TCF7L1
rs2439703	2	85,424,018	C	T	0.697	0.993	TCF7L1
rs2439704	2	85,424,067	C	T	0.697	0.993	TCF7L1
rs149256246	2	85,424,459	G	A	0.967	0.934	TCF7L1
rs61276534	2	85,424,825	A	G	0.963	0.991	TCF7L1
rs77718305	2	85,424,907	C	T	0.964	0.977	TCF7L1
rs57871178	2	85,425,048	T	C	0.824	0.991	TCF7L1
rs6733735	2	85,426,269	T	C	0.963	0.992	TCF7L1
rs72840132	2	85,426,409	G	C	0.948	0.994	TCF7L1
rs6747032	2	85,426,463	A	G	0.963	0.992	TCF7L1
rs6722568	2	85,427,109	G	A	0.538	0.997	TCF7L1
rs72840133	2	85,427,285	T	C	0.988	0.967	TCF7L1
rs7576858	2	85,428,320	C	A	0.948	0.976	TCF7L1
rs56691726	2	85,428,394	A	G	0.963	0.992	TCF7L1
rs115385669	2	85,428,843	G	A	0.986	0.983	TCF7L1
rs10192927	2	85,428,860	C	T	0.878	0.989	TCF7L1
2:85429060_TTTTATTTA_T	2	85,429,060	TTTTATTTA	T	0.537	0.995	TCF7L1
rs56044202	2	85,429,065	T	G	0.959	0.882	TCF7L1
rs11126983	2	85,429,198	G	A	0.697	0.993	TCF7L1
rs12478357	2	85,429,625	T	C	0.697	0.993	TCF7L1
rs62165563	2	85,430,588	G	A	0.877	0.989	TCF7L1
rs139266725	2	85,430,699	T	A	0.948	0.994	TCF7L1
rs200641081	2	85,431,420	C	CA	0.861	0.939	TCF7L1
2:85431741_CA_C	2	85,431,741	CA	C	0.697	0.989	TCF7L1
rs148854874	2	85,431,873	T	C	0.989	0.911	TCF7L1
rs550939144	2	85,432,006	T	G	0.948	0.992	TCF7L1
rs569897021	2	85,432,008	G	T	0.941	0.947	TCF7L1
rs59112392	2	85,432,012	G	T	0.486	0.994	TCF7L1
rs143431590	2	85,432,054	C	G	0.982	0.909	TCF7L1
rs17026015	2	85,432,404	A	C	0.963	0.993	TCF7L1
rs17026022	2	85,432,460	T	C	0.963	0.993	TCF7L1
rs72840137	2	85,432,589	G	C	0.948	0.995	TCF7L1
rs139745348	2	85,432,864	G	A	0.96	0.939	TCF7L1
rs72840138	2	85,432,941	G	C	0.948	0.995	TCF7L1
rs11126984	2	85,432,950	T	C	0.697	0.993	TCF7L1
rs11126985	2	85,433,270	G	A	0.697	0.993	TCF7L1
rs72932687	2	85,433,402	G	A	0.963	0.992	TCF7L1
rs72840140	2	85,433,793	G	T	0.948	0.994	TCF7L1
rs775054737	2	85,434,089	AAC	A	0.876	0.978	TCF7L1
rs79226341	2	85,434,104	C	T	0.948	0.995	TCF7L1
rs4832148	2	85,434,540	C	T	0.195	0.969	TCF7L1
rs57783218	2	85,434,569	T	C	0.948	0.995	TCF7L1
rs4831995	2	85,434,612	A	G	0.877	0.989	TCF7L1
rs17026026	2	85,434,777	G	A	0.948	0.994	TCF7L1
rs72840142	2	85,434,822	T	G	0.948	0.995	TCF7L1
rs57015835	2	85,435,039	T	G	0.946	0.983	TCF7L1
rs148346112	2	85,435,224	T	A	0.948	0.995	TCF7L1
rs57595681	2	85,435,281	G	A	0.948	0.995	TCF7L1
rs72840143	2	85,435,697	A	G	0.948	0.995	TCF7L1
rs7557425	2	85,435,944	A	G	0.485	0.998	TCF7L1
2:85436285_GT_G	2	85,436,285	GT	G	0.486	0.995	TCF7L1
rs12476165	2	85,436,884	C	T	0.697	0.993	TCF7L1
rs11692829	2	85,437,420	G	T	0.537	0.997	TCF7L1
rs11681532	2	85,437,437	A	G	0.537	0.997	TCF7L1
rs17026034	2	85,437,484	G	A	0.963	0.993	TCF7L1
rs6742305	2	85,437,945	C	T	0.538	0.997	TCF7L1
rs72840148	2	85,438,000	G	A	0.948	0.995	TCF7L1
rs72840149	2	85,438,065	C	T	0.948	0.995	TCF7L1
2:85438347_TA_T	2	85,438,347	TA	T	0.574	0.996	TCF7L1
rs62165582	2	85,438,540	C	T	0.697	0.993	TCF7L1
rs10208803	2	85,438,909	G	T	0.486	0.998	TCF7L1
rs11695920	2	85,439,169	C	T	0.697	0.993	TCF7L1
rs77690004	2	85,439,343	C	T	0.914	0.971	TCF7L1
rs2043228	2	85,439,553	T	C	0.485	0.999	TCF7L1
rs6721730	2	85,439,871	A	G	0.697	0.993	TCF7L1
rs72840150	2	85,440,836	T	C	0.948	0.996	TCF7L1
rs12619496	2	85,441,399	A	G	0.804	0.969	TCF7L1
rs72840151	2	85,441,851	G	A	0.964	0.99	TCF7L1
rs72840152	2	85,441,871	A	G	0.948	0.996	TCF7L1
rs72840154	2	85,442,180	C	T	0.948	0.997	TCF7L1
rs55868746	2	85,442,740	G	A	0.952	0.995	TCF7L1
rs556140985	2	85,442,900	T	G	0.98	0.699	TCF7L1
rs72840156	2	85,443,170	G	C	0.947	0.997	TCF7L1
rs6709118	2	85,443,650	G	A	0.444	0.99	TCF7L1
rs72840158	2	85,443,748	G	A	0.948	0.998	TCF7L1
rs146707102	2	85,444,248	C	CCTCA	0.982	0.919	TCF7L1
rs138867414	2	85,444,550	A	G	0.984	0.981	TCF7L1
rs62165583	2	85,444,579	C	T	0.724	0.974	TCF7L1
rs72840159	2	85,444,727	C	G	0.947	0.998	TCF7L1
rs140493464	2	85,445,492	C	T	0.95	0.939	TCF7L1
rs112515051	2	85,445,772	A	G	0.958	0.904	TCF7L1
2:85446208_AGG_A	2	85,446,208	AGG	A	0.949	0.958	TCF7L1
rs11440593	2	85,446,284	A	AT	0.948	0.992	TCF7L1
rs80092311	2	85,446,292	T	C	0.964	0.995	TCF7L1
rs115432706	2	85,446,376	A	G	0.987	0.888	TCF7L1
rs72932696	2	85,446,444	A	G	0.963	0.995	TCF7L1
rs6547598	2	85,446,486	A	G	0.91	0.998	TCF7L1
rs79311788	2	85,446,665	T	C	0.984	0.981	TCF7L1
rs6715431	2	85,447,024	T	A	0.964	0.995	TCF7L1
rs58953021	2	85,447,443	T	G	0.947	0.996	TCF7L1
rs6547599	2	85,447,484	A	T	0.397	0.975	TCF7L1
rs115746180	2	85,447,516	T	C	0.947	0.996	TCF7L1
rs201905759	2	85,447,572	A	ACCT	0.949	0.984	TCF7L1
rs140509284	2	85,447,709	G	A	0.947	0.995	TCF7L1
rs6547600	2	85,447,830	C	T	0.566	0.985	TCF7L1
rs55915336	2	85,448,225	C	T	0.948	0.995	TCF7L1
rs55879203	2	85,448,356	G	A	0.947	0.995	TCF7L1
rs55750438	2	85,448,855	C	G	0.947	0.994	TCF7L1
rs549470334	2	85,449,054	T	TA	0.867	0.843	TCF7L1
rs528216365	2	85,449,079	A	G	0.988	0.927	TCF7L1
rs150545389	2	85,449,379	G	A	0.963	0.988	TCF7L1
2:85449484_AAAC_A	2	85,449,484	AAAC	A	0.923	0.845	TCF7L1
rs537417295	2	85,449,487	C	A	0.933	0.75	TCF7L1
rs4494743	2	85,449,498	A	C	0.936	0.994	TCF7L1
rs141464165	2	85,451,246	G	C	0.959	0.992	TCF7L1
rs138027317	2	85,451,361	A	G	0.959	0.992	TCF7L1
rs143950280	2	85,451,547	A	C	0.959	0.992	TCF7L1
rs190138851	2	85,452,480	T	C	0.962	0.915	TCF7L1
rs139122780	2	85,453,111	T	C	0.959	0.991	TCF7L1
rs6743132	2	85,453,593	T	C	0.895	0.993	TCF7L1
rs10647217	2	85,453,713	T	TCTC	0.387	0.97	TCF7L1
rs62165586	2	85,453,860	A	G	0.671	0.98	TCF7L1
2:85453939_CT_C	2	85,453,939	CT	C	0.682	0.883	TCF7L1
rs72840167	2	85,454,257	G	A	0.959	0.991	TCF7L1
rs147871436	2	85,454,674	C	T	0.976	0.966	TCF7L1
rs72840168	2	85,454,861	A	G	0.959	0.99	TCF7L1
rs141582213	2	85,454,984	G	A	0.959	0.99	TCF7L1
rs72840170	2	85,455,171	A	G	0.959	0.985	TCF7L1
rs56042310	2	85,455,377	T	C	0.959	0.99	TCF7L1
rs72934610	2	85,455,675	C	T	0.937	0.995	TCF7L1
rs11902445	2	85,455,966	A	G	0.895	0.994	TCF7L1
rs10198011	2	85,456,138	T	C	0.196	0.94	TCF7L1
rs113230662	2	85,456,241	G	A	0.949	0.967	TCF7L1
rs11689554	2	85,456,321	T	C	0.669	0.981	TCF7L1
rs142398182	2	85,456,422	A	G	0.959	0.989	TCF7L1
rs6751413	2	85,457,738	A	G	0.959	0.989	TCF7L1
rs11674371	2	85,457,952	A	G	0.669	0.981	TCF7L1
rs6751795	2	85,458,054	A	G	0.959	0.989	TCF7L1
rs72840174	2	85,458,223	C	T	0.959	0.99	TCF7L1
rs79217476	2	85,458,263	T	C	0.951	0.961	TCF7L1
rs144707613	2	85,458,499	A	C	0.989	0.975	TCF7L1
rs56356236	2	85,458,657	T	G	0.959	0.99	TCF7L1
rs55742496	2	85,458,778	G	C	0.959	0.99	TCF7L1
rs56284888	2	85,459,036	C	T	0.959	0.99	TCF7L1
rs723465	2	85,459,682	G	T	0.67	0.981	TCF7L1
rs1347039	2	85,459,880	A	G	0.937	0.999	TCF7L1
2:85460439_AT_A	2	85,460,439	AT	A	0.282	0.901	TCF7L1
rs6749940	2	85,460,505	T	C	0.197	0.96	TCF7L1
rs6749942	2	85,460,510	T	C	0.896	0.995	TCF7L1
rs6750175	2	85,460,679	T	C	0.895	0.995	TCF7L1
rs189715444	2	85,460,684	G	A	0.985	0.968	TCF7L1
rs116175388	2	85,460,950	C	T	0.986	0.991	TCF7L1
rs72934620	2	85,461,217	C	G	0.938	0.999	TCF7L1
rs7588165	2	85,461,359	G	A	0.897	0.995	TCF7L1
rs72840178	2	85,461,832	A	G	0.959	0.989	TCF7L1
rs72840180	2	85,461,856	C	T	0.959	0.989	TCF7L1
rs72840181	2	85,462,009	T	G	0.959	0.989	TCF7L1
rs72840182	2	85,462,247	A	C	0.959	0.989	TCF7L1
rs72840183	2	85,462,248	G	A	0.959	0.989	TCF7L1
rs12477490	2	85,462,602	C	T	0.817	0.962	TCF7L1
rs56199411	2	85,462,717	C	T	0.976	0.918	TCF7L1
rs72840184	2	85,462,762	G	A	0.959	0.989	TCF7L1
rs12477587	2	85,462,773	G	A	0.817	0.962	TCF7L1

SNP	Chromosome	Position	Effect Allele	Other Allele	Effect Allele Frequency	Imputation quality	Gene
rs72840185	2	85,463,011	T	C	0.959	0.989	TCF7L1
rs562934236	2	85,463,287	T	TC	0.959	0.984	TCF7L1
rs34088919	2	85,463,296	C	CA	0.936	0.992	TCF7L1
rs369596256	2	85,463,299	A	AAT	0.966	0.89	TCF7L1
rs76321449	2	85,463,382	T	C	0.959	0.989	TCF7L1
rs75567130	2	85,464,155	C	G	0.941	0.99	TCF7L1
rs72840190	2	85,464,493	G	A	0.959	0.989	TCF7L1
rs72831497	2	85,464,682	C	T	0.959	0.989	TCF7L1
2:85464830_CAG_C	2	85,464,830	CAG	C	0.981	0.946	TCF7L1
rs114086642	2	85,465,250	G	C	0.959	0.989	TCF7L1
rs72831498	2	85,465,531	G	A	0.959	0.99	TCF7L1
2:85465773_CAG_C	2	85,465,773	CAG	C	0.969	0.988	TCF7L1
rs72831499	2	85,466,275	T	A	0.959	0.989	TCF7L1
rs72831500	2	85,466,278	A	G	0.959	0.99	TCF7L1
rs72831501	2	85,466,380	T	C	0.959	0.99	TCF7L1
rs62165588	2	85,466,864	G	A	0.682	0.977	TCF7L1
rs9653568	2	85,466,885	A	G	0.553	0.979	TCF7L1
rs62165589	2	85,466,925	G	A	0.682	0.977	TCF7L1
2:85467067_TAA_T	2	85,467,067	TAA	T	0.711	0.865	TCF7L1
rs146933767	2	85,467,875	G	A	0.94	0.986	TCF7L1
rs10205484	2	85,468,002	G	A	0.921	0.988	TCF7L1
rs541704128	2	85,468,570	T	G	0.929	0.992	TCF7L1
rs72840003	2	85,468,871	G	A	0.923	0.99	TCF7L1
rs11428091	2	85,469,146	C	CG	0.29	0.947	TCF7L1
rs6547602	2	85,470,087	A	G	0.57	0.986	TCF7L1
rs6753357	2	85,470,105	C	T	0.928	0.999	TCF7L1
rs17712582	2	85,470,163	C	T	0.94	0.988	TCF7L1
rs11675205	2	85,470,281	G	A	0.721	0.985	TCF7L1
rs17026089	2	85,470,383	C	G	0.978	0.962	TCF7L1
2:85470596_AGGGGAACATGCCCAGCGACTCCACGT_A	2	85,470,596	AGGGGAACATGCCCAGCGACTCCACGT	A	0.984	0.933	TCF7L1
rs6725799	2	85,470,987	A	G	0.465	0.985	TCF7L1
rs7566763	2	85,471,459	T	C	0.761	0.967	TCF7L1
2:85471639_AG_A	2	85,471,639	AG	A	0.923	0.994	TCF7L1
rs6758778	2	85,471,980	G	A	0.929	0.995	TCF7L1
rs6758805	2	85,472,055	G	A	0.929	0.995	TCF7L1
rs72840008	2	85,472,598	T	C	0.921	0.992	TCF7L1
rs561341831	2	85,472,956	TA	T	0.94	0.734	TCF7L1
rs6708270	2	85,473,260	C	A	0.567	0.992	TCF7L1
2:85473888_AT_A	2	85,473,888	AT	A	0.72	0.995	TCF7L1
rs72840009	2	85,474,340	C	A	0.92	0.992	TCF7L1
rs72840010	2	85,474,432	T	C	0.92	0.992	TCF7L1
rs6752527	2	85,475,397	C	T	0.719	0.995	TCF7L1
rs72840013	2	85,475,706	C	T	0.92	0.99	TCF7L1
rs17763700	2	85,475,827	T	C	0.937	0.985	TCF7L1
rs567891498	2	85,476,123	G	GA	0.632	0.975	TCF7L1
rs6547603	2	85,476,180	T	C	0.233	0.965	TCF7L1
rs72840015	2	85,476,558	G	A	0.92	0.989	TCF7L1
rs72840016	2	85,477,212	A	C	0.92	0.99	TCF7L1
rs72840017	2	85,477,413	A	C	0.92	0.99	TCF7L1
rs531810927	2	85,477,565	A	AT	0.72	0.977	TCF7L1
rs187252478	2	85,477,717	G	A	0.92	0.99	TCF7L1
rs6719095	2	85,477,720	T	C	0.566	0.991	TCF7L1
rs145805984	2	85,477,839	G	T	0.961	0.965	TCF7L1
rs55971915	2	85,478,670	C	G	0.921	0.987	TCF7L1
rs111534217	2	85,479,006	G	A	0.98	0.923	TCF7L1
rs72840018	2	85,479,130	C	T	0.922	0.989	TCF7L1
rs72840019	2	85,479,224	G	T	0.922	0.989	TCF7L1
rs72840022	2	85,479,948	T	G	0.939	0.983	TCF7L1
rs112561287	2	85,479,989	A	C	0.97	0.946	TCF7L1
rs62165594	2	85,480,011	T	C	0.569	0.992	TCF7L1
rs17763748	2	85,480,107	T	G	0.647	0.99	TCF7L1
rs17712901	2	85,480,425	G	A	0.57	0.992	TCF7L1
rs62165595	2	85,480,661	G	A	0.569	0.992	TCF7L1
rs17712942	2	85,480,698	T	G	0.922	0.989	TCF7L1
rs17763853	2	85,480,715	A	G	0.648	0.99	TCF7L1
rs150226983	2	85,480,774	G	A	0.986	0.934	TCF7L1
rs7581133	2	85,480,817	T	G	0.569	0.992	TCF7L1
rs17713036	2	85,481,399	G	A	0.922	0.989	TCF7L1
rs11684211	2	85,481,524	G	A	0.647	0.99	TCF7L1
rs55936700	2	85,482,107	C	T	0.922	0.989	TCF7L1
rs17713060	2	85,482,369	C	G	0.922	0.989	TCF7L1
rs78012323	2	85,482,746	G	A	0.779	0.998	TCF7L1
rs2043229	2	85,483,003	G	A	0.348	0.991	TCF7L1
rs114877623	2	85,483,061	G	A	0.984	0.914	TCF7L1
rs17026108	2	85,483,145	G	A	0.779	0.998	TCF7L1
rs2043230	2	85,483,350	A	T	0.426	0.99	TCF7L1
rs79026474	2	85,483,396	T	C	0.863	0.994	TCF7L1
rs72840024	2	85,483,704	G	T	0.648	0.989	TCF7L1
rs7592438	2	85,483,903	T	A	0.908	0.99	TCF7L1
rs543967503	2	85,484,105	T	TA	0.359	0.969	TCF7L1
rs11904127	2	85,484,818	G	A	0.427	0.989	TCF7L1
rs11904105	2	85,484,959	C	T	0.427	0.989	TCF7L1
rs80322989	2	85,485,251	G	A	0.962	0.967	TCF7L1
rs62165597	2	85,485,326	T	C	0.906	0.985	TCF7L1
rs11674662	2	85,485,915	G	A	0.478	0.985	TCF7L1
rs72840025	2	85,486,212	T	A	0.922	0.985	TCF7L1
rs11681706	2	85,486,354	T	G	0.232	0.989	TCF7L1
rs17713150	2	85,486,572	A	C	0.921	0.984	TCF7L1
rs76273648	2	85,486,702	G	A	0.982	0.948	TCF7L1
rs147494534	2	85,486,889	A	AT	0.978	0.899	TCF7L1
rs6547606	2	85,488,248	T	G	0.557	0.871	TCF7L1
rs774175062	2	85,488,250	T	G	0.953	0.842	TCF7L1
rs7584666	2	85,488,451	A	T	0.466	0.993	TCF7L1
rs115550005	2	85,488,826	C	G	0.975	0.986	TCF7L1
2:85488849_TA_T	2	85,488,849	TA	T	0.466	0.988	TCF7L1
rs2366264	2	85,489,011	G	T	0.466	0.993	TCF7L1
rs1991738	2	85,489,280	A	G	0.466	0.993	TCF7L1
rs11686410	2	85,489,297	T	A	0.466	0.993	TCF7L1
rs35687745	2	85,489,391	C	CT	0.465	0.989	TCF7L1
rs11126989	2	85,490,112	T	A	0.545	0.99	TCF7L1
rs72840027	2	85,490,167	G	A	0.922	0.984	TCF7L1
rs148908709	2	85,490,431	G	A	0.984	0.961	TCF7L1
rs142307693	2	85,490,442	A	AG	0.551	0.977	TCF7L1
rs11689667	2	85,491,365	T	C	0.545	0.991	TCF7L1
rs6547607	2	85,491,420	T	C	0.466	0.994	TCF7L1
rs883650	2	85,491,747	T	C	0.545	0.99	TCF7L1
rs883651	2	85,491,770	G	A	0.466	0.994	TCF7L1
rs908301	2	85,492,141	C	T	0.466	0.994	TCF7L1
rs882883	2	85,492,391	G	T	0.466	0.994	TCF7L1
rs565376298	2	85,492,415	T	TA	0.476	0.97	TCF7L1
rs1554109	2	85,493,002	A	C	0.466	0.994	TCF7L1
rs56124642	2	85,494,386	G	A	0.813	0.972	TCF7L1
rs750512672	2	85,494,772	CA	C	0.906	0.792	TCF7L1
rs17026122	2	85,495,570	C	G	0.975	0.992	TCF7L1
rs554305734	2	85,495,594	C	CTT	0.394	0.814	TCF7L1
2:85495890_TA_T	2	85,495,890	TA	T	0.243	0.931	TCF7L1
rs72840029	2	85,495,931	G	T	0.922	0.983	TCF7L1
rs55637419	2	85,497,104	C	T	0.921	0.983	TCF7L1
rs7340255	2	85,497,900	G	A	0.466	0.995	TCF7L1
rs150417168	2	85,498,299	G	A	0.987	0.982	TCF7L1
rs11675489	2	85,498,783	A	G	0.544	0.992	TCF7L1
rs62162671	2	85,498,886	C	T	0.652	0.983	TCF7L1
rs11686782	2	85,498,968	G	A	0.466	0.995	TCF7L1
rs10195229	2	85,499,642	G	A	0.746	0.998	TCF7L1
rs7340132	2	85,500,278	A	G	0.464	0.994	TCF7L1
rs10201321	2	85,501,073	G	A	0.747	0.999	TCF7L1
rs56152018	2	85,501,154	G	A	0.921	0.981	TCF7L1
rs10201483	2	85,501,384	C	A	0.748	0.994	TCF7L1
rs10201660	2	85,501,390	G	A	0.748	0.994	TCF7L1
rs10201489	2	85,501,409	C	T	0.748	0.994	TCF7L1
rs10201753	2	85,501,467	G	C	0.747	0.998	TCF7L1
rs4441469	2	85,501,999	G	A	0.747	0.999	TCF7L1
rs62162674	2	85,502,236	G	C	0.544	0.986	TCF7L1
rs12714137	2	85,502,264	A	C	0.134	0.983	TCF7L1
rs12714138	2	85,502,419	G	A	0.291	0.983	TCF7L1
rs536963605	2	85,503,042	C	CA	0.79	0.813	TCF7L1
rs6745529	2	85,503,139	G	T	0.544	0.986	TCF7L1
rs72840035	2	85,503,193	C	G	0.921	0.981	TCF7L1
rs10184690	2	85,503,568	A	C	0.747	0.999	TCF7L1
rs10184810	2	85,503,674	A	C	0.747	0.999	TCF7L1
rs4832151	2	85,503,996	A	G	0.211	0.986	TCF7L1
rs4832152	2	85,504,191	G	A	0.747	0.999	TCF7L1
rs908300	2	85,504,704	G	A	0.746	0.999	TCF7L1
rs882831	2	85,504,877	A	G	0.746	1	TCF7L1
rs4346385	2	85,504,989	A	G	0.29	0.982	TCF7L1
rs6747629	2	85,505,562	T	C	0.746	1	TCF7L1
rs6732834	2	85,505,602	C	T	0.933	0.938	TCF7L1
rs6733190	2	85,505,920	C	T	0.544	0.985	TCF7L1
rs6748258	2	85,505,980	T	A	0.746	1	TCF7L1
rs6733515	2	85,506,000	G	A	0.746	1	TCF7L1
rs6736348	2	85,506,104	G	A	0.211	0.986	TCF7L1
rs6736154	2	85,506,142	C	T	0.747	0.999	TCF7L1
rs6736366	2	85,506,160	G	A	0.291	0.981	TCF7L1
rs78294339	2	85,506,263	G	A	0.981	0.898	TCF7L1
rs60624652	2	85,506,500	A	ACGTT	0.746	0.998	TCF7L1

SNP	Chromosome	Position	Effect Allele	Other Allele	Effect Allele Frequency	Imputation quality	Gene
2:85506532_AAAAT_A	2	85,506,532	AAAAT	A	0.751	0.989	TCF7L1
rs79567947	2	85,506,687	G	A	0.981	0.939	TCF7L1
rs11126990	2	85,507,334	T	C	0.291	0.982	TCF7L1
rs10204252	2	85,507,657	G	A	0.746	0.999	TCF7L1
rs10180579	2	85,507,788	A	T	0.746	0.997	TCF7L1
rs10206876	2	85,508,065	C	A	0.746	0.995	TCF7L1
2:85508143_CTTT_C	2	85,508,143	CTTT	C	0.186	0.944	TCF7L1
rs4832153	2	85,508,303	T	C	0.211	0.981	TCF7L1
rs4832154	2	85,508,399	T	C	0.746	0.993	TCF7L1
rs4831998	2	85,508,454	T	C	0.745	0.993	TCF7L1
rs6745204	2	85,508,825	G	A	0.756	0.982	TCF7L1
rs6547608	2	85,508,850	G	A	0.608	0.976	TCF7L1
rs553108115	2	85,509,112	C	CT	0.912	0.943	TCF7L1
rs62162676	2	85,509,813	G	A	0.945	0.959	TCF7L1
rs7598047	2	85,510,018	G	A	0.913	0.967	TCF7L1
rs7600828	2	85,510,295	C	G	0.604	0.979	TCF7L1
rs34644194	2	85,510,400	A	G	0.604	0.967	TCF7L1
rs7601117	2	85,510,425	G	A	0.604	0.978	TCF7L1
rs7574856	2	85,510,777	A	G	0.517	0.981	TCF7L1
rs7574999	2	85,510,965	A	G	0.375	0.978	TCF7L1
rs55979428	2	85,510,978	C	T	0.843	0.997	TCF7L1
rs62162678	2	85,511,021	T	C	0.913	0.965	TCF7L1
rs72840042	2	85,511,594	A	G	0.842	0.996	TCF7L1
rs17713693	2	85,512,062	A	G	0.843	0.996	TCF7L1
rs112134288	2	85,512,095	G	A	0.974	0.977	TCF7L1
rs72840044	2	85,512,493	G	A	0.985	0.868	TCF7L1
rs6547609	2	85,512,675	T	C	0.532	0.976	TCF7L1
rs6719501	2	85,512,961	T	G	0.532	0.969	TCF7L1
rs72840046	2	85,512,979	G	T	0.843	0.985	TCF7L1
rs62162679	2	85,513,238	C	T	0.632	0.958	TCF7L1
rs28474865	2	85,513,486	C	A	0.918	0.886	TCF7L1
rs553158707	2	85,513,493	A	C	0.963	0.882	TCF7L1
rs577990883	2	85,513,494	C	A	0.963	0.882	TCF7L1
rs113385089	2	85,513,496	A	C	0.963	0.882	TCF7L1
rs7559779	2	85,513,804	G	C	0.915	0.945	TCF7L1
rs62162680	2	85,514,058	C	T	0.967	0.9	TCF7L1
rs62162681	2	85,514,124	A	G	0.944	0.918	TCF7L1
rs76277927	2	85,514,576	A	G	0.976	0.989	TCF7L1
rs56254758	2	85,516,217	C	A	0.847	0.892	TCF7L1
rs79115793	2	85,516,317	A	G	0.976	0.902	TCF7L1
rs6756335	2	85,517,039	C	T	0.932	0.87	TCF7L1
rs6759268	2	85,517,090	C	G	0.674	0.831	TCF7L1
2:85517099_GT_G	2	85,517,099	GT	G	0.548	0.783	TCF7L1
rs113194344	2	85,517,194	G	A	0.934	0.873	TCF7L1
rs62162682	2	85,517,200	A	G	0.931	0.867	TCF7L1
rs7607282	2	85,517,292	C	T	0.678	0.877	TCF7L1
rs56278313	2	85,517,386	G	A	0.801	0.884	TCF7L1
rs56379572	2	85,517,553	T	A	0.799	0.883	TCF7L1
rs10185095	2	85,517,590	T	C	0.596	0.859	TCF7L1
rs56389676	2	85,517,750	A	G	0.704	0.851	TCF7L1
rs56409903	2	85,517,769	G	A	0.744	0.852	TCF7L1
rs72840047	2	85,517,783	A	G	0.972	0.852	TCF7L1
rs7572226	2	85,518,483	T	C	0.908	0.971	TCF7L1
2:85518671_TGTTTTGGTTTTA_T	2	85,518,671	TGTTTTGGTTTTA	T	0.954	0.816	TCF7L1
rs78126551	2	85,518,714	G	T	0.932	0.971	TCF7L1
rs113392199	2	85,519,077	T	C	0.93	0.985	TCF7L1
rs139922251	2	85,519,840	C	A	0.942	0.979	TCF7L1
rs77616159	2	85,520,188	C	T	0.963	0.948	TCF7L1
rs59353316	2	85,521,088	A	C	0.895	0.979	TCF7L1
rs6718291	2	85,521,105	C	A	0.541	0.984	TCF7L1
rs573352561	2	85,521,171	T	C	0.944	0.905	TCF7L1
2:85521194_CTGTCGCCCAGGCTGG_C	2	85,521,194	CTGTCGCCCAGGCTGG	C	0.932	0.982	TCF7L1
rs13405014	2	85,521,196	G	C	0.519	0.956	TCF7L1
rs12470602	2	85,521,223	G	A	0.952	0.917	TCF7L1
rs145757488	2	85,521,252	C	T	0.99	0.909	TCF7L1
rs147633555	2	85,521,255	A	G	0.932	0.981	TCF7L1
rs7569653	2	85,521,523	G	A	0.539	0.99	TCF7L1
rs7583983	2	85,521,912	T	C	0.539	0.99	TCF7L1
rs76812146	2	85,522,163	T	A	0.93	0.993	TCF7L1
rs56191302	2	85,522,193	C	G	0.632	0.997	TCF7L1
rs137942571	2	85,522,510	A	G	0.931	0.994	TCF7L1
rs12471961	2	85,522,926	C	A	0.368	0.998	TCF7L1
rs56329464	2	85,523,164	A	G	0.931	0.995	TCF7L1
rs4832155	2	85,523,220	G	A	0.368	0.999	TCF7L1
rs4832156	2	85,523,276	A	G	0.539	0.993	TCF7L1
rs4832157	2	85,523,305	A	G	0.368	0.999	TCF7L1
rs4832158	2	85,523,476	G	A	0.542	0.993	TCF7L1
rs6737773	2	85,524,369	A	G	0.963	0.955	TCF7L1
rs76003864	2	85,524,822	C	T	0.931	0.999	TCF7L1
rs74619566	2	85,524,849	A	G	0.931	0.999	TCF7L1
rs10178604	2	85,524,876	C	T	0.541	0.989	TCF7L1
rs10178705	2	85,525,035	C	T	0.374	0.995	TCF7L1
rs116596126	2	85,525,119	C	T	0.931	0.998	TCF7L1
rs75912143	2	85,525,339	G	A	0.545	0.987	TCF7L1
rs7564589	2	85,525,490	C	T	0.461	0.994	TCF7L1
rs73943084	2	85,525,710	A	G	0.931	0.998	TCF7L1
2:85525822_GGC_G	2	85,525,822	GGC	G	0.913	0.994	TCF7L1
rs10184914	2	85,526,399	G	A	0.374	0.994	TCF7L1
rs55993057	2	85,526,578	G	A	0.931	0.998	TCF7L1
2:85526615_CA_C	2	85,526,615	CA	C	0.914	0.923	TCF7L1
rs9753285	2	85,526,806	A	G	0.913	0.996	TCF7L1
rs8179703	2	85,526,913	T	C	0.46	0.993	TCF7L1
rs73943085	2	85,527,451	T	C	0.913	0.998	TCF7L1
rs73943086	2	85,527,557	A	T	0.913	0.999	TCF7L1
rs73943087	2	85,527,701	C	T	0.914	0.998	TCF7L1
rs55943620	2	85,528,269	T	C	0.931	0.998	TCF7L1
rs56297212	2	85,528,384	G	A	0.931	0.998	TCF7L1
rs872470	2	85,528,643	A	G	0.931	0.998	TCF7L1
rs5832642	2	85,528,695	T	TTTTC	0.931	0.997	TCF7L1
rs141553072	2	85,529,019	C	T	0.968	0.969	TCF7L1
rs13001442	2	85,529,981	A	G	0.551	0.985	TCF7L1
rs73943088	2	85,530,027	T	C	0.902	0.988	TCF7L1
rs13033165	2	85,530,065	T	C	0.37	0.986	TCF7L1
rs72934690	2	85,530,399	G	A	0.921	0.992	TCF7L1
rs10174520	2	85,530,561	A	G	0.373	0.984	TCF7L1
rs73943089	2	85,530,676	T	C	0.93	0.995	TCF7L1
rs11900333	2	85,531,579	C	A	0.546	0.982	TCF7L1
rs77680559	2	85,531,790	T	C	0.917	0.924	TCF7L1
rs12714139	2	85,531,807	C	A	0.32	0.984	TCF7L1
rs6741339	2	85,532,205	C	G	0.32	0.986	TCF7L1
rs41288841	2	85,533,949	G	A	0.987	0.947	TCF7L1
rs41288843	2	85,534,039	G	A	0.902	0.989	TCF7L1
rs73943090	2	85,534,203	G	T	0.93	0.998	TCF7L1
rs112617052	2	85,534,296	T	TAACCC	0.498	0.983	TCF7L1
rs76090477	2	85,534,366	A	G	0.93	0.999	TCF7L1
rs59059406	2	85,534,392	T	C	0.875	0.998	TCF7L1
rs61326180	2	85,534,567	G	A	0.945	0.995	TCF7L1
rs73943091	2	85,534,569	G	A	0.872	0.996	TCF7L1
rs59653199	2	85,534,574	T	C	0.875	0.997	TCF7L1
rs4497880	2	85,534,950	G	A	0.875	0.998	TCF7L1
rs183819123	2	85,535,091	T	C	0.988	0.986	TCF7L1
rs4641936	2	85,535,187	C	T	0.944	0.998	TCF7L1
rs78480770	2	85,535,501	T	A	0.962	0.835	TCF7L1
rs554024559	2	85,535,521	T	C	0.977	0.917	TCF7L1
rs72936604	2	85,535,540	T	C	0.373	0.91	TCF7L1
rs61440596	2	85,535,570	C	G	0.285	0.863	TCF7L1
2:85535579_CCT_C	2	85,535,579	CCT	C	0.725	0.883	TCF7L1
2:85535616_TC_T	2	85,535,616	TC	T	0.933	0.927	TCF7L1
rs9248	2	85,537,312	G	A	0.496	0.995	TCF7L1
rs9333287	2	187,455,278	C	T	0.25	0.998	ITGAV
rs61763608	2	187,455,911	T	C	0.933	0.996	ITGAV
rs3768777	2	187,456,121	A	G	0.317	0.998	ITGAV
rs61763609	2	187,456,603	A	G	0.984	0.921	ITGAV
2:187457301_GT_G	2	187,457,301	GT	G	0.253	0.951	ITGAV
rs12693454	2	187,457,588	A	G	0.254	0.999	ITGAV
rs61142253	2	187,458,188	G	GT	0.289	0.905	ITGAV
rs35294409	2	187,458,513	G	GT	0.253	0.997	ITGAV
rs116190329	2	187,459,077	T	A	0.987	0.968	ITGAV
rs3754584	2	187,459,497	C	G	0.933	0.998	ITGAV
2:187460415_TA_T	2	187,460,415	TA	T	0.251	0.983	ITGAV
rs61763614	2	187,460,849	A	G	0.988	0.919	ITGAV
rs61763615	2	187,461,099	T	G	0.984	0.99	ITGAV
rs61763616	2	187,461,542	T	C	0.972	0.955	ITGAV
rs61763617	2	187,462,133	T	C	0.989	0.999	ITGAV
rs4233788	2	187,462,581	A	G	0.254	0.999	ITGAV
rs3845710	2	187,466,028	T	A	0.135	0.634	ITGAV
rs3845712	2	187,466,180	G	C	0.254	0.999	ITGAV
rs200501166	2	187,466,921	A	AT	0.935	0.893	ITGAV
2:187467603_TA_T	2	187,467,603	TA	T	0.257	0.722	ITGAV
rs3768778	2	187,467,750	A	G	0.238	0.999	ITGAV
rs61763625	2	187,468,005	T	C	0.984	0.987	ITGAV
rs72903148	2	187,470,190	T	G	0.746	1	ITGAV
rs3911239	2	187,470,311	C	T	0.322	1	ITGAV
rs3911238	2	187,470,344	G	C	0.741	0.989	ITGAV
2:187470467_GTATA_G	2	187,470,467	GTATA	G	0.083	0.968	ITGAV

SNP	Chromosome	Position	Effect Allele	Other Allele	Effect Allele Frequency	Imputation quality	Gene
rs2887827	2	187,471,481	G	C	0.746	1	ITGAV
rs79030355	2	187,473,139	A	G	0.978	0.976	ITGAV
rs34432414	2	187,474,520	C	CAT	0.261	0.981	ITGAV
rs2370693	2	187,475,104	G	T	0.254	1	ITGAV
rs10172499	2	187,476,862	C	T	0.254	1	ITGAV
rs546219169	2	187,477,096	A	AT	0.749	0.942	ITGAV
rs10804007	2	187,477,691	A	C	0.254	1	ITGAV
rs3768780	2	187,478,500	A	G	0.254	1	ITGAV
rs34712474	2	187,479,648	A	G	0.852	0.977	ITGAV
rs66630114	2	187,479,756	C	A	0.747	1	ITGAV
rs62197748	2	187,480,006	T	C	0.933	1	ITGAV
rs146492209	2	187,480,200	G	T	0.987	0.813	ITGAV
rs79077104	2	187,480,302	T	A	0.882	0.999	ITGAV
2:187481764_GT_G	2	187,481,764	GT	G	0.249	0.986	ITGAV
rs3768781	2	187,481,835	A	G	0.741	0.99	ITGAV
rs756729874	2	187,483,471	GC	G	0.254	1	ITGAV
2:187483838_TTA_T	2	187,483,838	TTA	T	0.731	0.97	ITGAV
rs115066755	2	187,484,018	A	G	0.932	1	ITGAV
rs2370696	2	187,484,040	A	T	0.882	0.998	ITGAV
2:187484293_CA_C	2	187,484,293	CA	C	0.254	0.999	ITGAV
rs2165656	2	187,484,854	G	T	0.26	0.997	ITGAV
rs115272668	2	187,484,927	C	T	0.968	0.984	ITGAV
rs61763627	2	187,486,642	G	A	0.963	0.964	ITGAV
rs9333288	2	187,487,160	G	A	0.254	1	ITGAV
rs6741369	2	187,487,269	G	T	0.747	0.999	ITGAV
2:187488028_AT_A	2	187,488,028	AT	A	0.921	0.95	ITGAV
rs150751185	2	187,488,319	A	G	0.979	0.997	ITGAV
rs12620429	2	187,489,647	C	A	0.832	0.993	ITGAV
rs2197787	2	187,490,371	T	G	0.253	0.999	ITGAV
rs530701191	2	187,490,418	T	G	0.967	0.531	ITGAV
rs1992898	2	187,491,140	G	A	0.253	0.999	ITGAV
rs10171481	2	187,492,460	G	A	0.253	0.999	ITGAV
rs147151379	2	187,492,465	G	C	0.989	0.875	ITGAV
rs10174098	2	187,492,742	G	A	0.321	0.999	ITGAV
rs13006571	2	187,492,964	A	G	0.253	0.999	ITGAV
rs2197138	2	187,493,841	A	G	0.253	0.999	ITGAV
rs7596996	2	187,494,427	G	A	0.253	0.998	ITGAV
rs3835763	2	187,497,146	T	TG	0.791	0.824	ITGAV
rs34429410	2	187,497,376	A	G	0.979	0.932	ITGAV
rs139232448	2	187,497,818	C	T	0.97	0.979	ITGAV
rs56088351	2	187,497,988	A	G	0.891	0.987	ITGAV
rs9333289	2	187,498,107	T	C	0.721	0.993	ITGAV
rs4667107	2	187,498,254	T	C	0.765	0.993	ITGAV
rs7585442	2	187,498,492	A	T	0.763	0.996	ITGAV
rs7585471	2	187,498,562	A	G	0.763	0.996	ITGAV
rs4016811	2	187,499,009	CAT	C	0.762	0.99	ITGAV
rs5836990	2	187,499,187	G	GA	0.29	0.995	ITGAV
rs28616716	2	187,500,029	A	G	0.891	0.992	ITGAV
rs35540241	2	187,501,429	C	A	0.763	0.996	ITGAV
rs34980163	2	187,501,447	A	C	0.291	0.998	ITGAV
rs13430921	2	187,501,648	A	G	0.891	0.994	ITGAV
rs56136540	2	187,501,682	G	A	0.401	0.997	ITGAV
2:187502208_CA_C	2	187,502,208	CA	C	0.416	0.943	ITGAV
rs13422838	2	187,502,846	T	C	0.891	0.994	ITGAV
rs4667108	2	187,503,226	T	C	0.627	0.995	ITGAV
2:187503358_CGT_C	2	187,503,358	CGT	C	0.577	0.928	ITGAV
rs41270221	2	187,503,399	A	G	0.889	0.984	ITGAV
rs77238834	2	187,505,146	G	A	0.979	0.967	ITGAV
rs3768784	2	187,505,167	G	A	0.774	0.997	ITGAV
rs3816375	2	187,505,486	A	G	0.627	0.996	ITGAV
rs3768785	2	187,506,786	A	G	0.764	0.996	ITGAV
rs79257155	2	187,506,998	G	A	0.987	0.957	ITGAV
2:187507108_CTTG_C	2	187,507,108	CTTG	C	0.639	0.996	ITGAV
2:187507262_CT_C	2	187,507,262	CT	C	0.392	0.898	ITGAV
rs141126622	2	187,507,428	A	AT	0.639	0.999	ITGAV
rs1448424	2	187,507,972	C	T	0.293	0.998	ITGAV
rs1839121	2	187,508,198	C	T	0.293	0.998	ITGAV
rs56222785	2	187,508,997	A	G	0.763	0.996	ITGAV
2:187509016_AT_A	2	187,509,016	AT	A	0.984	0.955	ITGAV
rs55781198	2	187,509,041	G	A	0.764	0.997	ITGAV
rs565971146	2	187,509,547	T	TA	0.644	0.983	ITGAV
rs6748509	2	187,509,664	T	C	0.293	0.998	ITGAV
rs61765186	2	187,509,703	G	A	0.774	0.997	ITGAV
rs75252638	2	187,509,788	T	A	0.984	0.975	ITGAV
rs10178617	2	187,510,049	T	C	0.891	0.995	ITGAV
rs140837318	2	187,510,592	G	A	0.979	0.967	ITGAV
rs16828148	2	187,511,072	G	A	0.774	0.997	ITGAV
rs71017334	2	187,511,965	A	AT	0.443	0.883	ITGAV
rs7575516	2	187,513,281	G	A	0.708	0.999	ITGAV
rs6753438	2	187,513,341	A	G	0.293	0.999	ITGAV
rs79863731	2	187,513,730	C	T	0.638	0.957	ITGAV
2:187513735_TTGTG_T	2	187,513,735	TTGTG	T	0.627	0.995	ITGAV
2:187513750_TAC_T	2	187,513,750	TAC	T	0.627	0.994	ITGAV
rs776010184	2	187,513,763	G	GTA	0.891	0.993	ITGAV
rs9753297	2	187,513,810	C	T	0.292	0.996	ITGAV
rs61762241	2	187,513,858	A	G	0.639	0.999	ITGAV
rs3820739	2	187,514,182	T	C	0.639	0.999	ITGAV
rs116135748	2	187,515,088	G	A	0.968	0.904	ITGAV
rs4667109	2	187,517,655	T	C	0.628	0.996	ITGAV
rs13007986	2	187,517,721	A	G	0.401	0.997	ITGAV
rs569994762	2	187,518,148	G	A	0.989	0.762	ITGAV
rs3768787	2	187,518,260	A	G	0.774	0.998	ITGAV
rs61762251	2	187,518,715	T	C	0.979	0.935	ITGAV
rs17406098	2	187,520,330	G	T	0.891	0.995	ITGAV
rs3738919	2	187,521,260	C	A	0.639	0.999	ITGAV
2:187521742_CACACACACAG_C	2	187,521,742	CACACACACAG	C	0.764	0.995	ITGAV
rs66470626	2	187,521,752	G	A	0.741	0.935	ITGAV
rs7589470	2	187,522,406	G	A	0.401	0.998	ITGAV
rs7589682	2	187,522,612	G	C	0.639	0.999	ITGAV
2:187522680_GA_G	2	187,522,680	GA	G	0.767	0.987	ITGAV
rs7565633	2	187,522,681	A	G	0.594	0.944	ITGAV
rs145908871	2	187,522,690	A	C	0.98	0.552	ITGAV
rs13006483	2	187,522,750	G	T	0.722	0.998	ITGAV
rs34840521	2	187,522,788	A	G	0.763	0.997	ITGAV
rs58757504	2	187,525,193	C	T	0.774	0.998	ITGAV
rs2682855	2	187,525,962	A	T	0.891	0.997	ITGAV
rs35348196	2	187,526,090	T	C	0.763	0.998	ITGAV
rs11903048	2	187,526,180	A	T	0.639	0.999	ITGAV
rs5011663	2	187,526,331	T	G	0.627	0.855	ITGAV
rs150913282	2	187,526,332	T	G	0.677	0.984	ITGAV
rs1839124	2	187,526,614	A	G	0.291	0.996	ITGAV
rs1448427	2	187,526,672	G	A	0.763	0.999	ITGAV
rs3816386	2	187,528,635	A	G	0.707	0.999	ITGAV
rs55767908	2	187,528,695	A	G	0.865	0.999	ITGAV
rs2595392	2	187,530,116	C	G	0.891	0.999	ITGAV
rs2084448	2	187,530,520	T	C	0.707	1	ITGAV
rs3213963	2	187,532,234	G	A	0.774	1	ITGAV
rs139810557	2	187,533,016	G	C	0.773	0.998	ITGAV
rs11685758	2	187,533,368	C	T	0.707	0.999	ITGAV
rs2290083	2	187,533,741	T	C	0.629	1	ITGAV
rs2595389	2	187,534,183	T	G	0.529	1	ITGAV
rs2682857	2	187,535,271	G	A	0.891	0.999	ITGAV
rs377749051	2	187,536,672	C	CT	0.363	0.859	ITGAV
rs143705891	2	187,537,422	C	A	0.985	0.981	ITGAV
rs12151442	2	187,537,847	T	C	0.708	1	ITGAV
rs115786254	2	187,538,664	C	A	0.979	0.964	ITGAV
rs116287053	2	187,539,229	A	T	0.962	0.975	ITGAV
rs3213964	2	187,540,732	G	A	0.481	1	ITGAV
rs10931252	2	187,540,967	G	T	0.774	1	ITGAV
rs12620821	2	187,541,318	G	A	0.481	1	ITGAV
rs2887831	2	187,541,810	T	C	0.774	0.999	ITGAV
2:187542936_TAGTA_T	2	187,542,936	TAGTA	T	0.707	0.997	ITGAV
rs11891461	2	187,543,123	A	T	0.722	0.999	ITGAV
rs11902171	2	187,543,227	G	C	0.722	0.999	ITGAV
rs1839123	2	187,543,374	C	T	0.708	0.999	ITGAV
rs3747	2	218,664,633	T	G	0.497	0.957	TNS1
rs13454	2	218,665,073	G	A	0.979	0.902	TNS1
rs4674215	2	218,666,889	G	A	0.694	0.918	TNS1
rs3791979	2	218,667,372	T	C	0.397	0.949	TNS1
rs3088214	2	218,667,836	G	A	0.4	0.981	TNS1
rs1063281	2	218,668,732	C	T	0.399	0.984	TNS1
rs539814780	2	218,669,146	T	C	0.983	0.669	TNS1
rs75156815	2	218,669,558	A	G	0.855	0.976	TNS1
rs74897150	2	218,669,929	A	G	0.927	0.972	TNS1
2:218670455_ACACCACACATAC_A	2	218,670,455	ACACCACACATAC	A	0.702	0.943	TNS1
rs2552520	2	218,670,771	C	T	0.4	0.991	TNS1
rs2571461	2	218,670,945	T	G	0.4	0.991	TNS1
rs77538871	2	218,670,991	G	A	0.894	0.985	TNS1
rs972229	2	218,671,328	T	G	0.711	0.987	TNS1
rs972230	2	218,671,349	A	G	0.711	0.987	TNS1
rs972231	2	218,671,599	T	C	0.034	0.943	TNS1
rs2241451	2	218,672,657	C	T	0.855	0.987	TNS1
rs34831382	2	218,672,782	A	G	0.899	0.969	TNS1
rs35136714	2	218,673,147	T	C	0.69	0.994	TNS1
rs13025249	2	218,673,804	T	A	0.69	0.995	TNS1

SNP	Chromosome	Position	Effect Allele	Other Allele	Effect Allele Frequency	Imputation quality	Gene
rs34307680	2	218,673,857	G	A	0.921	0.988	TNS1
rs759871122	2	218,673,893	GGGAT	G	0.401	0.997	TNS1
rs559313208	2	218,673,952	A	G	0.972	0.91	TNS1
rs1035671	2	218,674,484	C	T	0.934	0.995	TNS1
rs13389312	2	218,674,514	A	T	0.921	0.989	TNS1
rs1035672	2	218,675,199	G	A	0.4	1	TNS1
rs113311422	2	218,675,226	A	T	0.982	0.9	TNS1
rs62183753	2	218,675,383	C	T	0.921	0.991	TNS1
rs1035673	2	218,675,533	T	C	0.4	0.999	TNS1
2:218676003_GT_G	2	218,676,003	GT	G	0.968	0.999	TNS1
rs11692329	2	218,676,704	C	T	0.689	0.997	TNS1
rs1991161	2	218,676,890	C	T	0.433	0.997	TNS1
rs76043829	2	218,677,586	G	A	0.894	0.999	TNS1
rs10490755	2	218,677,659	G	A	0.923	0.981	TNS1
rs7562261	2	218,678,749	G	A	0.968	0.997	TNS1
rs11695740	2	218,678,883	G	A	0.722	0.991	TNS1
rs79798163	2	218,679,225	C	G	0.968	1	TNS1
rs2059408	2	218,679,599	T	C	0.815	0.986	TNS1
rs76302586	2	218,680,017	A	G	0.856	0.985	TNS1
rs2161967	2	218,680,529	T	G	0.426	0.992	TNS1
rs2161968	2	218,680,545	G	A	0.687	0.984	TNS1
rs2161969	2	218,680,570	G	A	0.522	0.984	TNS1
rs41383244	2	218,680,591	A	G	0.654	0.995	TNS1
rs35955543	2	218,680,778	C	T	0.723	0.99	TNS1
rs4674216	2	218,681,340	C	T	0.633	0.993	TNS1
rs4674217	2	218,681,575	G	C	0.595	0.998	TNS1
rs12476660	2	218,682,162	C	G	0.674	0.999	TNS1
rs13424201	2	218,682,248	C	A	0.921	0.999	TNS1
rs149255260	2	218,682,255	C	T	0.987	0.901	TNS1
rs2571445	2	218,683,154	A	G	0.396	1	TNS1
rs34144104	2	218,683,368	C	T	0.712	0.995	TNS1
rs12466972	2	218,683,907	T	C	0.592	0.998	TNS1
rs13394030	2	218,684,259	T	C	0.921	0.998	TNS1
rs62183755	2	218,684,798	G	C	0.917	0.995	TNS1
rs2571434	2	218,685,627	T	C	0.626	0.992	TNS1
rs111429146	2	218,685,663	C	T	0.988	0.897	TNS1
rs13022333	2	218,685,664	T	C	0.661	0.993	TNS1
rs34681173	2	218,686,038	G	A	0.918	0.995	TNS1
rs34572447	2	218,686,119	T	A	0.977	0.931	TNS1
rs35833791	2	218,687,381	G	A	0.918	0.993	TNS1
rs71415814	2	218,687,471	C	T	0.989	0.842	TNS1
rs141581041	2	218,687,563	G	A	0.989	0.834	TNS1
rs35949566	2	218,687,711	G	A	0.918	0.993	TNS1
rs2552527	2	218,688,596	T	G	0.591	0.995	TNS1
rs34085205	2	218,688,883	C	G	0.918	0.992	TNS1
rs11897815	2	218,690,487	T	C	0.704	0.986	TNS1
rs7582112	2	218,691,327	T	C	0.788	0.927	TNS1
rs80062932	2	218,691,374	G	A	0.932	0.888	TNS1
rs4674218	2	218,691,633	T	C	0.782	0.99	TNS1
rs2571435	2	218,691,759	C	T	0.753	0.991	TNS1
rs2571438	2	218,693,550	G	A	0.717	0.989	TNS1
2:218693944_CAA_C	2	218,693,944	CAA	C	0.217	0.896	TNS1
rs6729308	2	218,694,153	G	A	0.56	0.99	TNS1
rs6744219	2	218,694,192	T	C	0.56	0.991	TNS1
rs6729330	2	218,694,296	C	T	0.561	0.99	TNS1
rs6729561	2	218,694,357	G	A	0.56	0.991	TNS1
rs867161128	2	218,694,510	CT	C	0.314	0.986	TNS1
rs10188605	2	218,694,691	A	G	0.643	0.996	TNS1
rs4674219	2	218,694,842	C	A	0.754	0.997	TNS1
rs10165160	2	218,694,962	C	G	0.671	0.991	TNS1
rs2571440	2	218,695,686	T	C	0.175	0.969	TNS1
rs2552529	2	218,695,798	A	G	0.356	0.983	TNS1
rs10168611	2	218,695,846	G	A	0.604	0.984	TNS1
rs10168454	2	218,695,848	C	A	0.605	0.984	TNS1
rs2552531	2	218,698,323	A	G	0.948	0.961	TNS1
rs2571443	2	218,698,621	T	C	0.896	0.995	TNS1
rs16858320	2	218,699,505	G	A	0.896	0.997	TNS1
rs200245788	2	218,703,790	T	TAC	0.895	0.962	TNS1
rs77986730	2	218,703,868	G	A	0.99	0.864	TNS1
rs2571449	2	218,704,101	T	C	0.501	0.965	TNS1
rs1008835	2	218,704,238	A	G	0.894	0.974	TNS1
rs73992637	2	218,704,455	T	C	0.894	0.974	TNS1
rs34466485	2	218,704,476	A	C	0.894	0.974	TNS1
rs61616923	2	218,704,480	A	G	0.604	0.966	TNS1
rs1122986	2	218,704,674	G	A	0.494	0.967	TNS1
rs1122987	2	218,704,894	T	G	0.894	0.971	TNS1
rs2571452	2	218,704,978	T	G	0.619	0.972	TNS1
rs55764538	2	218,705,648	C	T	0.658	0.973	TNS1
rs762245657	2	218,705,650	TG	T	0.658	0.973	TNS1
rs1000980	2	218,705,948	T	C	0.656	0.982	TNS1
rs1000979	2	218,705,987	C	T	0.663	0.982	TNS1
rs1000978	2	218,706,167	C	A	0.656	0.984	TNS1
rs2571454	2	218,706,259	G	A	0.656	0.985	TNS1
rs918951	2	218,706,723	G	T	0.691	0.993	TNS1
rs918952	2	218,706,857	T	C	0.656	0.986	TNS1
rs60916851	2	218,707,083	A	AC	0.655	0.982	TNS1
rs11899788	2	218,707,213	T	C	0.656	0.987	TNS1
rs116651932	2	218,707,548	G	A	0.974	0.968	TNS1
rs41497947	2	218,707,753	T	G	0.655	0.989	TNS1
rs55840817	2	218,707,806	C	A	0.656	0.989	TNS1
rs563698232	2	218,707,856	T	TA	0.674	0.958	TNS1
rs55763753	2	218,708,089	T	C	0.656	0.991	TNS1
rs4643515	2	218,708,214	G	C	0.656	0.991	TNS1
rs1364637	2	218,708,432	A	G	0.629	0.989	TNS1
rs1364638	2	218,708,503	A	G	0.656	0.991	TNS1
rs1364639	2	218,708,524	G	A	0.691	0.999	TNS1
rs1364640	2	218,708,632	G	C	0.691	0.999	TNS1
rs929937	2	218,709,215	T	C	0.629	0.989	TNS1
rs59997697	2	218,709,462	C	T	0.691	1	TNS1
rs12474617	2	218,709,753	G	A	0.691	1	TNS1
rs7566566	2	218,710,264	G	C	0.656	0.992	TNS1
rs7566504	2	218,710,380	C	T	0.691	1	TNS1
rs1559590	2	218,710,631	G	A	0.691	1	TNS1
rs1559591	2	218,710,723	A	C	0.694	0.995	TNS1
rs1559592	2	218,710,727	C	T	0.694	0.995	TNS1
rs1559593	2	218,710,730	A	T	0.694	0.995	TNS1
rs929938	2	218,710,924	T	C	0.629	0.989	TNS1
rs58343915	2	218,711,440	A	G	0.629	0.989	TNS1
rs987336	2	218,711,611	T	C	0.629	0.989	TNS1
rs4519498	2	218,711,612	A	G	0.629	0.989	TNS1
rs987337	2	218,711,692	T	G	0.664	0.997	TNS1
rs987338	2	218,711,738	C	A	0.664	0.997	TNS1
rs987339	2	218,711,818	G	C	0.664	0.997	TNS1
rs55763335	2	218,712,102	A	G	0.629	0.99	TNS1
rs1004814	2	218,712,306	C	T	0.692	1	TNS1
rs3796031	2	218,712,918	C	T	0.592	0.987	TNS1
rs3796032	2	218,712,936	C	T	0.692	1	TNS1
rs7587518	2	218,713,924	A	G	0.631	0.983	TNS1
rs11902263	2	218,714,201	T	G	0.695	0.985	TNS1
rs11902267	2	218,714,218	T	C	0.66	0.978	TNS1
rs10170040	2	218,714,330	A	T	0.561	0.953	TNS1
rs12998464	2	218,714,408	C	T	0.965	0.946	TNS1
rs16858371	2	218,714,496	C	T	0.742	0.972	TNS1
rs3791978	2	218,714,686	A	C	0.526	0.971	TNS1
rs3791977	2	218,714,845	G	A	0.598	0.959	TNS1
rs3791976	2	218,714,851	T	A	0.411	0.964	TNS1
rs3791975	2	218,715,071	A	G	0.885	0.953	TNS1
rs13005083	2	218,715,292	A	G	0.885	0.954	TNS1
rs3791974	2	218,715,344	A	G	0.897	0.861	TNS1
rs56391849	2	218,715,351	C	T	0.579	0.954	TNS1
rs17733847	2	218,715,406	A	C	0.83	0.973	TNS1
rs764993782	2	218,715,862	CAT	C	0.953	0.932	TNS1
rs76930125	2	218,715,955	T	C	0.988	0.849	TNS1
rs3791973	2	218,716,190	A	G	0.887	0.955	TNS1
rs56074520	2	218,716,875	A	C	0.739	0.991	TNS1
rs56096813	2	218,717,053	A	G	0.691	0.984	TNS1
rs10171651	2	218,717,638	T	A	0.824	0.942	TNS1
rs3791972	2	218,717,645	A	G	0.952	0.958	TNS1
rs3791970	2	218,719,132	A	G	0.952	0.959	TNS1
rs3791969	2	218,719,300	T	A	0.739	0.995	TNS1
rs3791968	2	218,719,330	A	C	0.739	0.995	TNS1
rs3828291	2	218,719,588	C	T	0.739	0.995	TNS1
rs2059409	2	218,720,042	C	T	0.952	0.96	TNS1
rs3791967	2	218,720,087	A	G	0.739	0.996	TNS1
rs3791966	2	218,720,268	G	A	0.952	0.96	TNS1
rs72950126	2	218,720,298	T	C	0.988	0.504	TNS1
rs3791965	2	218,720,319	G	A	0.739	0.996	TNS1
rs3791964	2	218,720,394	G	A	0.739	0.996	TNS1
rs3791963	2	218,720,641	G	A	0.952	0.961	TNS1
rs3791962	2	218,720,671	T	C	0.739	0.996	TNS1
rs7581920	2	218,721,241	T	C	0.739	0.999	TNS1
rs7568256	2	218,721,394	G	A	0.739	0.998	TNS1
rs3791961	2	218,721,834	A	C	0.691	0.99	TNS1
rs3791960	2	218,722,427	C	A	0.739	0.991	TNS1
rs3791959	2	218,722,506	C	G	0.697	0.986	TNS1

SNP	Chromosome	Position	Effect Allele	Other Allele	Effect Allele Frequency	Imputation quality	Gene
rs141862535	2	218,722,526	G	A	0.987	0.882	TNS1
rs3791958	2	218,722,896	G	A	0.941	0.967	TNS1
rs3791957	2	218,722,905	A	G	0.495	0.981	TNS1
rs3791956	2	218,723,064	A	G	0.823	0.999	TNS1
rs74771548	2	218,723,252	G	A	0.966	0.941	TNS1
rs6436017	2	218,723,455	A	G	0.504	0.991	TNS1
rs6725482	2	218,723,580	C	A	0.504	0.991	TNS1
rs57319322	2	218,723,766	A	G	0.552	0.994	TNS1
rs3791953	2	218,725,480	C	A	0.824	0.993	TNS1
rs3828288	2	218,725,568	T	A	0.565	0.993	TNS1
rs749386	2	218,726,025	G	A	0.743	0.992	TNS1
rs749387	2	218,726,026	T	C	0.823	0.993	TNS1
rs78580768	2	218,726,772	C	T	0.986	0.884	TNS1
rs1424915	2	218,726,998	G	A	0.566	0.993	TNS1
rs2373223	2	218,728,562	A	G	0.951	0.972	TNS1
rs145591275	2	218,729,078	A	G	0.978	0.915	TNS1
rs140946803	2	218,729,783	G	A	0.965	0.99	TNS1
rs1863132	2	218,731,915	A	G	0.85	0.993	TNS1
rs1863133	2	218,732,208	T	C	0.455	0.998	TNS1
rs2113768	2	218,732,405	G	C	0.936	0.989	TNS1
rs56203324	2	218,732,524	T	C	0.669	0.999	TNS1
rs77147469	2	218,732,620	A	G	0.936	0.989	TNS1
rs17790748	2	218,732,851	A	G	0.669	1	TNS1
rs1035674	2	218,733,126	A	G	0.076	0.966	TNS1
rs17790821	2	218,733,667	G	A	0.937	0.988	TNS1
rs112941127	2	218,733,965	G	A	0.97	0.971	TNS1
rs144787402	2	218,734,288	G	A	0.986	0.847	TNS1
rs3791949	2	218,734,362	T	C	0.67	0.996	TNS1
rs3828287	2	218,734,653	G	A	0.987	0.92	TNS1
2:218734803_AC_A	2	218,734,803	AC	A	0.942	0.984	TNS1
rs10204348	2	218,734,953	A	G	0.625	0.995	TNS1
rs17790839	2	218,735,122	T	A	0.67	0.995	TNS1
rs2042541	2	218,736,178	T	C	0.668	0.995	TNS1
rs2042542	2	218,736,292	A	G	0.668	0.995	TNS1
rs13407109	2	218,736,908	G	T	0.627	0.993	TNS1
rs1991162	2	218,738,078	T	C	0.479	0.996	TNS1
2:218738746_AAC_A	2	218,738,746	AAC	A	0.609	0.972	TNS1
rs3791948	2	218,739,035	C	T	0.627	0.994	TNS1
rs3791947	2	218,739,125	A	G	0.668	0.994	TNS1
rs1424916	2	218,739,300	T	C	0.481	0.997	TNS1
rs1424917	2	218,739,732	C	G	0.826	0.988	TNS1
rs1424918	2	218,741,495	G	A	0.467	0.999	TNS1
rs3791944	2	218,743,211	A	T	0.958	0.98	TNS1
rs3791943	2	218,743,903	A	G	0.897	0.98	TNS1
rs890048	2	218,744,676	C	T	0.502	0.974	TNS1
rs56886627	2	218,744,795	A	G	0.898	0.981	TNS1
rs11898107	2	218,744,803	C	T	0.958	0.983	TNS1
rs890049	2	218,744,886	C	T	0.542	0.977	TNS1
rs2288164	2	218,745,477	G	T	0.963	0.984	TNS1
rs7421896	2	218,745,862	A	G	0.953	0.988	TNS1
rs2888521	2	218,746,277	C	T	0.918	0.991	TNS1
rs3791941	2	218,746,431	G	A	0.718	0.965	TNS1
rs2303381	2	218,746,990	A	T	0.957	0.987	TNS1
rs11680854	2	218,747,073	A	C	0.982	0.873	TNS1
rs2303383	2	218,747,190	T	C	0.918	0.996	TNS1
rs2373224	2	218,747,312	T	C	0.674	0.983	TNS1
rs13427550	2	218,747,816	G	T	0.917	0.998	TNS1
rs13390002	2	218,747,828	T	C	0.917	0.998	TNS1
rs13428061	2	218,748,278	G	A	0.917	0.999	TNS1
rs4674221	2	218,748,835	C	T	0.793	0.982	TNS1
rs4672853	2	218,748,870	G	A	0.893	0.681	TNS1
rs562041033	2	218,748,870	G	GTGGA	0.918	0.995	TNS1
rs4674222	2	218,749,036	G	A	0.917	0.999	TNS1
rs3791940	2	218,749,379	G	A	0.917	0.998	TNS1
rs2288165	2	218,749,626	T	C	0.917	0.997	TNS1
rs6742531	2	218,750,136	A	G	0.917	0.996	TNS1
rs6729299	2	218,750,154	T	A	0.917	0.996	TNS1
rs6714429	2	218,750,239	G	A	0.917	0.996	TNS1
rs6714587	2	218,750,359	G	A	0.917	0.996	TNS1
rs12990490	2	218,750,377	C	T	0.917	0.996	TNS1
rs2288166	2	218,750,703	C	T	0.917	0.995	TNS1
rs2288167	2	218,750,731	G	C	0.917	0.994	TNS1
rs2288168	2	218,750,734	T	A	0.917	0.994	TNS1
rs10211238	2	218,750,817	T	C	0.917	0.993	TNS1
rs116819734	2	218,753,780	A	G	0.973	0.873	TNS1
rs55837823	2	218,753,893	T	A	0.96	0.971	TNS1
rs10174633	2	218,754,326	T	C	0.898	0.981	TNS1
rs975441	2	218,756,117	C	T	0.896	0.978	TNS1
rs6436018	2	218,756,550	A	G	0.065	0.931	TNS1
rs1345989	2	218,757,417	T	G	0.895	0.975	TNS1
rs56217129	2	218,758,070	C	A	0.782	0.971	TNS1
rs3835057	2	218,758,125	A	G	0.88	0.753	TNS1
rs74270365	2	218,759,065	C	T	0.989	0.953	TNS1
rs1808466	2	218,759,104	A	G	0.895	0.971	TNS1
rs79783479	2	218,759,407	C	A	0.953	0.964	TNS1
rs2288169	2	218,759,674	G	C	0.905	0.96	TNS1
rs12616228	2	218,760,307	C	T	0.99	0.959	TNS1
rs140194066	2	218,761,617	C	T	0.984	0.906	TNS1
rs7567473	2	218,761,618	G	A	0.98	0.829	TNS1
rs3791936	2	218,768,422	T	G	0.625	0.985	TNS1
rs3791935	2	218,768,581	G	A	0.87	0.973	TNS1
rs78047671	2	218,768,847	C	G	0.971	0.933	TNS1
rs7600493	2	218,770,003	G	A	0.628	0.993	TNS1
rs1863798	2	218,770,783	A	G	0.621	0.994	TNS1
rs2288170	2	218,770,984	C	T	0.894	0.99	TNS1
rs4674223	2	218,771,544	C	T	0.628	0.999	TNS1
rs6739316	2	218,772,403	G	C	0.735	0.99	TNS1
rs4674225	2	218,772,518	C	A	0.618	0.995	TNS1
rs3791930	2	218,772,682	A	T	0.334	0.986	TNS1
rs2888522	2	218,773,309	G	C	0.989	0.816	TNS1
rs77847632	2	218,773,847	C	G	0.959	0.947	TNS1
rs58839628	2	218,773,852	C	A	0.987	0.995	TNS1
rs566458511	2	218,774,904	T	C	0.986	0.617	TNS1
rs6436019	2	218,775,054	T	C	0.987	0.992	TNS1
rs6755375	2	218,776,372	C	T	0.638	0.988	TNS1
2:218777176_GC_G	2	218,777,176	GC	G	0.647	0.988	TNS1
rs114250753	2	218,777,809	G	A	0.966	0.913	TNS1
rs113495808	2	218,777,831	A	G	0.93	0.988	TNS1
rs3791922	2	218,778,549	C	T	0.648	0.997	TNS1
rs918947	2	218,778,966	C	T	0.418	0.991	TNS1
rs918948	2	218,778,972	C	T	0.418	0.991	TNS1
rs3828282	2	218,779,144	C	G	0.432	0.992	TNS1
rs4672855	2	218,780,196	T	C	0.065	0.982	TNS1
rs10199479	2	218,781,481	G	A	0.658	0.979	TNS1
rs890051	2	218,782,054	C	T	0.631	0.94	TNS1
rs1863796	2	218,782,423	G	A	0.855	0.925	TNS1
rs28403309	2	218,782,540	T	C	0.795	0.865	TNS1
rs2888530	2	218,782,578	C	A	0.186	0.865	TNS1
rs112067359	2	218,782,688	G	C	0.988	0.657	TNS1
rs13426335	2	218,782,760	G	A	0.754	0.895	TNS1
rs890052	2	218,783,078	G	A	0.039	0.966	TNS1
rs35855182	2	218,783,188	C	T	0.927	0.997	TNS1
rs16858459	2	218,783,227	G	C	0.982	0.993	TNS1
rs113921243	2	218,783,399	C	T	0.986	0.721	TNS1
rs10208644	2	218,783,606	G	A	0.942	0.999	TNS1
rs76730390	2	218,783,659	G	A	0.956	0.996	TNS1
rs3791919	2	218,783,751	A	G	0.942	0.994	TNS1
rs3791918	2	218,783,760	T	C	0.942	0.994	TNS1
rs3791917	2	218,783,774	C	T	0.942	0.993	TNS1
rs3791916	2	218,784,050	A	G	0.942	0.992	TNS1
rs3791915	2	218,784,146	C	T	0.989	0.988	TNS1
rs3791914	2	218,784,207	G	C	0.979	0.997	TNS1
rs3828280	2	218,784,225	T	C	0.942	0.991	TNS1
rs2288171	2	218,784,377	C	T	0.963	0.987	TNS1
rs2288172	2	218,784,378	C	G	0.979	0.998	TNS1
rs2288173	2	218,784,401	G	C	0.942	0.991	TNS1
rs2288174	2	218,784,409	G	A	0.988	0.985	TNS1
rs2288175	2	218,784,480	A	G	0.942	0.991	TNS1
rs4672856	2	218,784,778	C	G	0.988	0.981	TNS1
rs4672857	2	218,784,781	G	A	0.988	0.981	TNS1
rs7590152	2	218,784,877	A	G	0.976	0.978	TNS1
rs74988442	2	218,785,245	C	T	0.988	0.962	TNS1
rs115100271	2	218,787,428	A	G	0.978	0.999	TNS1
rs3791913	2	218,787,676	A	G	0.721	0.971	TNS1
rs6725612	2	218,788,543	G	A	0.792	0.993	TNS1
rs77109122	2	218,789,041	A	G	0.978	0.999	TNS1
rs34134227	2	218,789,105	C	G	0.791	0.996	TNS1
rs4672859	2	218,789,397	C	T	0.767	0.996	TNS1
rs150404355	2	218,789,837	T	G	0.984	0.914	TNS1
rs35593161	2	218,790,349	C	T	0.99	0.967	TNS1
rs115443079	2	218,791,285	C	T	0.978	0.998	TNS1
rs10197671	2	218,791,398	A	G	0.759	0.985	TNS1
rs79879064	2	218,793,552	A	T	0.99	0.918	TNS1
rs115528434	2	218,793,753	A	G	0.971	0.916	TNS1
rs3838559	2	218,793,843	A	AC	0.8	0.953	TNS1

SNP	Chromosome	Position	Effect Allele	Other Allele	Effect Allele Frequency	Imputation quality	Gene
rs3791911	2	218,793,983	G	A	0.826	0.958	TNS1
rs142780135	2	218,794,532	T	C	0.984	0.827	TNS1
rs1427669	2	218,795,356	T	G	0.775	0.977	TNS1
rs4672861	2	218,795,391	C	T	0.984	0.943	TNS1
rs4674231	2	218,795,497	G	A	0.983	0.941	TNS1
rs1427668	2	218,795,718	G	C	0.827	0.984	TNS1
rs7585036	2	218,795,794	C	G	0.797	0.986	TNS1
rs115286133	2	218,795,839	A	G	0.971	0.974	TNS1
rs56372827	2	218,795,891	A	T	0.792	0.791	TNS1
rs142110237	2	218,796,146	C	T	0.989	0.918	TNS1
rs114706538	2	218,796,270	C	A	0.966	0.939	TNS1
rs62182182	2	218,796,947	G	A	0.938	0.942	TNS1
rs11898315	2	218,797,766	G	A	0.786	0.988	TNS1
rs3791910	2	218,798,147	G	A	0.815	0.981	TNS1
rs4672862	2	218,798,645	C	T	0.786	0.982	TNS1
rs1365964	2	218,799,554	G	A	0.819	0.964	TNS1
rs1365963	2	218,799,846	G	C	0.785	0.966	TNS1
rs146359078	2	218,799,933	G	A	0.989	0.83	TNS1
rs113077169	2	218,800,912	T	G	0.986	0.859	TNS1
rs61203898	2	218,801,271	A	G	0.989	0.905	TNS1
rs77385765	2	218,801,585	C	T	0.988	0.931	TNS1
2:218801898_TCTACTCCC_T	2	218,801,898	TCTACTCCC	T	0.842	0.945	TNS1
rs3791906	2	218,803,329	T	C	0.837	0.99	TNS1
rs3791904	2	218,804,984	T	C	0.916	0.988	TNS1
2:218805581_AC_A	2	218,805,581	AC	A	0.96	0.989	TNS1
rs1820321	2	218,806,111	G	C	0.726	0.835	TNS1
rs3791902	2	218,806,748	A	C	0.755	0.989	TNS1
rs3791901	2	218,807,547	A	G	0.959	0.998	TNS1
rs76846520	2	218,808,967	G	T	0.96	0.993	TNS1
rs116246811	2	218,810,167	G	A	0.986	0.947	TNS1
rs6753621	2	218,810,908	G	A	0.776	0.992	TNS1
rs12998693	2	218,810,969	T	C	0.952	0.852	TNS1
rs1549589	2	218,811,135	G	A	0.77	0.995	TNS1
rs2373238	2	218,811,584	T	C	0.774	0.992	TNS1
rs7566469	2	218,811,962	T	A	0.893	0.992	TNS1
rs3791896	2	218,812,081	T	C	0.769	0.994	TNS1
rs72951936	2	218,812,507	C	A	0.892	0.992	TNS1
rs16858477	2	218,812,840	C	G	0.893	0.992	TNS1
rs147513135	2	218,813,275	G	A	0.98	0.762	TNS1
rs768905390	2	218,813,621	AAGG	A	0.959	0.995	TNS1
rs558785055	2	218,814,462	C	CA	0.947	0.889	TNS1
rs11676885	2	218,815,138	C	T	0.774	0.99	TNS1
rs139712284	2	218,815,241	G	A	0.986	0.769	TNS1
rs11676955	2	218,815,258	C	G	0.925	0.99	TNS1
rs744753	2	218,816,078	C	T	0.925	0.997	TNS1
rs6436020	2	218,816,342	C	T	0.925	0.997	TNS1
rs10177639	2	218,816,702	C	T	0.966	0.993	TNS1
rs56277119	2	218,817,762	A	G	0.893	0.993	TNS1
rs3828277	2	218,818,468	C	T	0.959	0.998	TNS1
rs138452642	2	218,819,061	A	G	0.971	0.817	TNS1
rs10932734	2	218,819,136	A	C	0.923	0.991	TNS1
rs3791893	2	218,819,396	G	A	0.881	0.994	TNS1
rs3791892	2	218,819,498	C	T	0.959	0.997	TNS1
rs3791891	2	218,819,527	C	T	0.959	0.997	TNS1
rs41481251	2	218,819,599	T	C	0.959	0.997	TNS1
rs72951947	2	218,819,944	G	A	0.893	0.994	TNS1
rs145672528	2	218,820,699	G	A	0.988	0.83	TNS1
2:218820751_GC_G	2	218,820,751	GC	G	0.967	0.989	TNS1
rs10932735	2	218,820,895	G	A	0.885	0.996	TNS1
rs72951949	2	218,820,897	A	T	0.959	0.997	TNS1
rs72951950	2	218,821,022	G	T	0.959	0.997	TNS1
rs111480425	2	218,821,152	C	CACAGAAGAGTCAG	0.78	0.967	TNS1
rs6147174	2	218,821,153	G	A	0.941	0.836	TNS1
rs2059739	2	218,821,157	A	G	0.913	0.875	TNS1
rs2059737	2	218,821,384	G	A	0.879	0.999	TNS1
rs3791890	2	218,821,586	A	G	0.92	0.998	TNS1
rs2059736	2	218,821,721	C	T	0.959	0.998	TNS1
rs3791889	2	218,822,007	T	C	0.754	0.997	TNS1
rs56246725	2	218,822,008	G	A	0.959	0.998	TNS1
rs3791888	2	218,822,050	A	G	0.92	0.997	TNS1
rs78641098	2	218,822,108	C	T	0.988	0.719	TNS1
rs11685533	2	218,822,239	T	C	0.92	0.997	TNS1
rs79921729	2	218,822,417	A	G	0.88	0.998	TNS1
rs80306247	2	218,822,690	T	A	0.88	0.999	TNS1
rs59189064	2	218,823,371	G	A	0.959	0.997	TNS1
rs3791887	2	218,823,564	C	G	0.921	0.993	TNS1
rs28660348	2	218,824,025	A	G	0.874	0.992	TNS1
rs16858496	2	218,824,450	G	A	0.959	0.995	TNS1
rs11682925	2	218,824,529	C	T	0.922	0.988	TNS1
rs11892423	2	218,824,573	C	T	0.959	0.995	TNS1
rs11904083	2	218,824,711	A	G	0.959	0.995	TNS1
rs56120913	2	218,825,097	G	A	0.959	0.994	TNS1
rs75746429	2	218,825,350	C	T	0.877	0.995	TNS1
rs55915879	2	218,825,470	T	G	0.792	0.995	TNS1
rs76633721	2	218,825,489	C	T	0.955	0.985	TNS1
rs16858505	2	218,825,490	A	G	0.79	0.997	TNS1
rs16858509	2	218,825,607	G	C	0.959	0.994	TNS1
rs55910223	2	218,825,941	G	C	0.808	0.954	TNS1
rs3055031	2	218,825,960	A	T	0.972	0.605	TNS1
rs3838557	2	218,825,983	C	G	0.939	0.739	TNS1
rs3791886	2	218,826,077	C	A	0.79	0.999	TNS1
rs78578073	2	218,826,591	T	C	0.958	0.985	TNS1
rs66692436	2	218,826,849	T	C	0.79	0.997	TNS1
rs6436021	2	218,826,937	C	T	0.959	0.992	TNS1
rs6436022	2	218,827,026	C	T	0.79	0.996	TNS1
rs74422933	2	218,827,548	A	G	0.877	0.988	TNS1
rs11688938	2	218,827,778	G	A	0.919	0.969	TNS1
rs11694996	2	218,827,913	T	C	0.919	0.968	TNS1
rs148291228	2	218,828,015	C	A	0.972	0.81	TNS1
rs16858515	2	218,828,035	G	T	0.959	0.976	TNS1
rs55997849	2	218,828,131	G	A	0.959	0.967	TNS1
rs13417602	2	218,828,434	C	T	0.903	0.902	TNS1
2:218828468_GA_G	2	218,828,468	GA	G	0.833	0.608	TNS1
rs77748418	2	218,828,635	G	A	0.87	0.897	TNS1
rs13013766	2	218,829,527	G	C	0.855	0.934	TNS1
rs13018351	2	218,829,609	C	A	0.851	0.954	TNS1
rs35303461	2	218,830,626	C	T	0.887	0.998	TNS1
rs35638311	2	218,830,685	G	A	0.885	0.992	TNS1
rs55828421	2	218,831,029	T	C	0.664	0.876	TNS1
rs55789159	2	218,831,031	T	C	0.664	0.876	TNS1
rs552342148	2	218,831,039	C	T	0.962	0.801	TNS1
rs62182221	2	218,831,041	C	T	0.896	0.954	TNS1
rs79766819	2	218,831,043	C	T	0.888	0.961	TNS1
2:218831644_TG_T	2	218,831,644	TG	T	0.885	0.972	TNS1
rs12619872	2	218,832,951	C	T	0.74	0.969	TNS1
rs200063541	2	218,834,162	C	A	0.833	0.535	TNS1
rs112895563	2	218,834,282	C	T	0.982	0.775	TNS1
rs2373239	2	218,835,438	C	G	0.883	0.974	TNS1
rs11674313	2	218,838,347	C	T	0.727	0.976	TNS1
rs147451459	2	218,839,924	A	T	0.977	0.811	TNS1
rs750132	2	218,840,362	G	A	0.89	0.965	TNS1
rs140384809	2	218,840,854	A	G	0.981	0.93	TNS1
rs13022979	2	218,841,301	G	A	0.892	0.97	TNS1
rs1863794	2	218,841,330	C	T	0.718	0.977	TNS1
rs3791882	2	218,841,333	T	G	0.711	0.979	TNS1
rs11903364	2	218,842,207	C	G	0.891	0.968	TNS1
rs62182224	2	218,843,880	A	T	0.885	0.978	TNS1
rs16858524	2	218,843,998	C	A	0.883	0.975	TNS1
rs62182225	2	218,845,229	C	G	0.871	0.867	TNS1
rs150610225	2	218,845,548	T	C	0.989	0.686	TNS1
rs11678396	2	218,845,937	C	A	0.724	0.991	TNS1
rs881745	2	218,846,455	C	G	0.721	0.99	TNS1
rs2081777	2	218,846,821	G	T	0.719	0.99	TNS1
rs1579406	2	218,847,092	C	A	0.885	0.973	TNS1
rs1863793	2	218,848,610	G	A	0.976	0.954	TNS1
2:218851453_TACACACAC_T	2	218,851,453	TACACACAC	T	0.621	0.934	TNS1
rs114002218	2	218,851,720	C	T	0.984	0.882	TNS1
rs112669932	2	218,851,928	C	T	0.976	0.94	TNS1
rs934039	2	218,853,391	G	A	0.719	0.962	TNS1
rs112326794	2	218,854,793	C	T	0.936	0.663	TNS1
rs111163654	2	218,854,860	A	G	0.973	0.889	TNS1
rs149308317	2	218,855,775	T	C	0.976	0.833	TNS1
rs77778441	2	218,856,891	C	G	0.949	0.925	TNS1
rs1365962	2	218,857,832	T	C	0.543	0.941	TNS1
rs55678041	2	218,858,046	T	C	0.715	0.996	TNS1
rs72613754	2	218,858,377	G	A	0.855	0.978	TNS1
rs71403019	2	218,858,881	G	GCAGGGTACTGCC	0.841	0.898	TNS1
rs4674233	2	218,859,428	G	C	0.828	0.981	TNS1
rs4674234	2	218,859,478	C	T	0.828	0.984	TNS1
rs74726669	2	218,860,201	T	C	0.916	0.909	TNS1
rs10932736	2	218,860,606	C	G	0.772	0.959	TNS1
rs2042825	2	218,860,694	G	A	0.799	0.977	TNS1
rs77939743	2	218,861,859	A	G	0.962	0.811	TNS1
rs139652990	2	218,861,966	A	C	0.989	0.897	TNS1
rs115673664	2	218,862,065	G	A	0.974	0.955	TNS1

SNP	Chromosome	Position	Effect Allele	Other Allele	Effect Allele Frequency	Imputation quality	Gene
rs1001098	2	218,862,241	C	T	0.829	0.997	TNS1
rs13003127	2	218,863,197	C	T	0.767	0.984	TNS1
rs1122921	2	218,863,891	T	C	0.854	0.992	TNS1
rs6712327	2	218,864,319	T	C	0.622	0.991	TNS1
rs62182230	2	218,865,806	G	A	0.797	0.997	TNS1
2:218865889_AT_A	2	218,865,889	AT	A	0.797	0.996	TNS1
rs76480788	2	218,866,486	C	T	0.971	0.979	TNS1
rs13382750	2	218,867,708	C	A	0.979	0.934	TNS1
rs3771547	2	242,296,273	G	C	0.808	0.999	FARP2
rs1476698	2	242,296,449	A	G	0.631	0.999	FARP2
rs13413380	2	242,297,091	C	T	0.776	0.999	FARP2
rs60624480	2	242,297,118	G	A	0.639	0.999	FARP2
rs138768348	2	242,297,340	A	G	0.989	0.98	FARP2
rs79443259	2	242,297,624	A	T	0.933	0.979	FARP2
rs115779893	2	242,297,841	A	G	0.978	0.994	FARP2
rs35064954	2	242,298,111	C	T	0.957	0.952	FARP2
rs16843590	2	242,298,228	G	A	0.838	0.999	FARP2
rs62193199	2	242,300,076	G	T	0.838	0.999	FARP2
rs57878067	2	242,300,088	C	CA	0.829	0.904	FARP2
2:242300443_GA_G	2	242,300,443	GA	G	0.74	0.952	FARP2
rs60178325	2	242,300,651	A	T	0.814	1	FARP2
rs73002144	2	242,301,037	C	T	0.97	0.989	FARP2
rs62193201	2	242,301,363	G	A	0.814	1	FARP2
rs111309489	2	242,301,982	T	C	0.838	0.999	FARP2
rs73002147	2	242,302,934	T	C	0.838	0.999	FARP2
rs62193202	2	242,303,717	G	A	0.838	0.999	FARP2
rs543957880	2	242,305,389	C	CT	0.988	0.674	FARP2
rs375900701	2	242,305,538	GTA	G	0.214	0.998	FARP2
rs7560216	2	242,308,039	A	T	0.214	0.999	FARP2
2:242308625_GTTTT_G	2	242,308,625	GTTTT	G	0.919	0.991	FARP2
rs145068921	2	242,309,218	T	G	0.987	0.924	FARP2
rs138811002	2	242,309,492	C	T	0.987	0.96	FARP2
rs116433700	2	242,309,863	C	T	0.983	0.978	FARP2
rs11684105	2	242,310,174	T	C	0.776	0.999	FARP2
2:242311505_CAT_C	2	242,311,505	CAT	C	0.837	0.998	FARP2
rs190383987	2	242,311,509	G	A	0.978	0.993	FARP2
rs151280108	2	242,312,353	C	CT	0.814	0.999	FARP2
rs201276541	2	242,312,477	G	GT	0.823	0.968	FARP2
rs61739702	2	242,312,572	G	A	0.978	0.996	FARP2
rs80142668	2	242,315,023	A	G	0.975	0.941	FARP2
rs62193204	2	242,315,443	A	G	0.838	0.999	FARP2
rs3771551	2	242,315,636	G	A	0.907	0.999	FARP2
2:242315941_TC_T	2	242,315,941	TC	T	0.214	0.998	FARP2
rs62193205	2	242,316,512	C	T	0.838	0.999	FARP2
rs62193206	2	242,316,843	G	A	0.838	0.999	FARP2
2:242317685_AT_A	2	242,317,685	AT	A	0.813	0.986	FARP2
rs11681267	2	242,317,777	C	G	0.776	0.999	FARP2
rs148410604	2	242,317,921	G	A	0.984	0.97	FARP2
rs6728083	2	242,317,952	T	C	0.214	0.999	FARP2
rs62193207	2	242,318,501	G	C	0.838	0.999	FARP2
2:242320105_ATATAT_A	2	242,320,105	ATATAT	A	0.775	0.996	FARP2
rs138207977	2	242,320,602	T	C	0.989	0.981	FARP2
rs3771553	2	242,321,026	G	A	0.214	0.999	FARP2
rs987559	2	242,321,083	G	A	0.176	0.995	FARP2
rs6761444	2	242,321,358	C	T	0.776	0.999	FARP2
rs73002160	2	242,321,707	A	C	0.814	0.999	FARP2
rs112347514	2	242,321,764	C	T	0.907	0.999	FARP2
rs10169743	2	242,321,890	G	T	0.778	0.993	FARP2
rs10206261	2	242,321,898	A	G	0.778	0.993	FARP2
rs62193208	2	242,322,430	G	A	0.838	0.999	FARP2
rs10172825	2	242,322,586	G	A	0.776	0.999	FARP2
rs10197237	2	242,322,640	C	T	0.776	0.999	FARP2
rs6723363	2	242,322,667	A	G	0.176	0.995	FARP2
rs3771555	2	242,322,680	T	C	0.639	0.999	FARP2
rs116680784	2	242,323,333	C	T	0.973	0.978	FARP2
rs62193209	2	242,323,523	A	G	0.838	0.999	FARP2
rs62193210	2	242,323,616	G	A	0.838	0.999	FARP2
rs112476475	2	242,323,637	A	G	0.983	0.949	FARP2
rs4675964	2	242,324,760	A	C	0.826	0.995	FARP2
rs4675963	2	242,324,763	G	A	0.826	0.995	FARP2
rs10172060	2	242,325,381	T	A	0.776	0.999	FARP2
rs1580908	2	242,325,823	A	G	0.214	0.999	FARP2
rs73002170	2	242,325,980	G	A	0.814	1	FARP2
rs3771557	2	242,326,323	A	G	0.214	0.999	FARP2
rs13395218	2	242,326,869	C	T	0.214	0.999	FARP2
rs62193211	2	242,327,076	T	A	0.838	0.999	FARP2
rs76342332	2	242,327,218	A	G	0.907	0.998	FARP2
rs9967861	2	242,328,078	G	A	0.776	0.999	FARP2
rs140832924	2	242,329,642	G	A	0.989	0.911	FARP2
rs1532788	2	242,330,192	G	T	0.214	0.999	FARP2
rs62193212	2	242,332,146	G	A	0.99	0.936	FARP2
rs577909105	2	242,332,364	G	GA	0.899	0.965	FARP2
rs73002176	2	242,332,438	T	C	0.814	0.999	FARP2
rs4675962	2	242,332,715	G	T	0.907	0.999	FARP2
rs11690426	2	242,333,889	C	A	0.576	0.998	FARP2
2:242333932_CA_C	2	242,333,932	CA	C	0.895	0.86	FARP2
rs3771559	2	242,334,335	G	A	0.576	0.999	FARP2
rs3821280	2	242,334,508	A	G	0.907	0.999	FARP2
rs13423900	2	242,335,424	T	C	0.776	0.999	FARP2
rs62193213	2	242,336,614	G	A	0.837	0.999	FARP2
rs6724088	2	242,336,856	C	T	0.223	0.999	FARP2
2:242337246_CT_C	2	242,337,246	CT	C	0.628	0.94	FARP2
rs11686991	2	242,337,299	C	T	0.799	0.998	FARP2
rs11886549	2	242,337,722	G	C	0.214	0.999	FARP2
2:242338395_ATAC_A	2	242,338,395	ATAC	A	0.957	0.978	FARP2
rs137942246	2	242,339,103	A	G	0.989	0.977	FARP2
rs73002180	2	242,339,585	G	C	0.95	0.993	FARP2
rs10687319	2	242,339,644	A	AAG	0.521	0.992	FARP2
rs3755398	2	242,339,649	T	G	0.214	0.999	FARP2
rs3755399	2	242,339,656	A	G	0.825	0.999	FARP2
rs6746127	2	242,340,540	G	A	0.776	0.999	FARP2
rs13426326	2	242,340,674	C	T	0.776	0.999	FARP2
rs36065346	2	242,341,233	C	CTT	0.214	0.999	FARP2
rs11678364	2	242,342,253	A	T	0.393	0.993	FARP2
rs73002181	2	242,342,379	C	G	0.818	0.998	FARP2
rs61615527	2	242,342,768	T	A	0.813	0.999	FARP2
rs12695001	2	242,342,984	T	C	0.214	0.999	FARP2
2:242343090_CT_C	2	242,343,090	CT	C	0.988	0.9	FARP2
rs3732276	2	242,343,404	C	T	0.893	0.999	FARP2
rs114169239	2	242,343,795	C	A	0.979	0.993	FARP2
rs11681497	2	242,344,333	A	G	0.893	0.999	FARP2
rs60132543	2	242,344,599	T	C	0.813	0.999	FARP2
rs139354822	2	242,344,695	T	C	0.972	0.941	FARP2
rs142489389	2	242,344,922	A	G	0.956	0.989	FARP2
rs62193216	2	242,345,130	C	T	0.814	0.999	FARP2
rs13001766	2	242,345,534	T	C	0.733	0.948	FARP2
rs370920525	2	242,345,543	T	C	0.988	0.826	FARP2
2:242345782_TTA_T	2	242,345,782	TTA	T	0.863	0.903	FARP2
rs13019759	2	242,345,837	G	T	0.504	0.928	FARP2
rs59488662	2	242,345,839	G	T	0.802	0.99	FARP2
rs55751610	2	242,346,057	C	T	0.837	1	FARP2
rs10180733	2	242,346,246	G	A	0.776	0.998	FARP2
rs111410563	2	242,346,291	C	T	0.907	0.999	FARP2
rs183529608	2	242,346,424	T	C	0.988	0.971	FARP2
rs60890112	2	242,346,471	C	G	0.613	0.995	FARP2
rs113231082	2	242,346,505	G	C	0.906	0.998	FARP2
rs115271170	2	242,347,614	C	T	0.893	0.999	FARP2
rs80085676	2	242,347,907	G	A	0.893	0.999	FARP2
2:242347956_CA_C	2	242,347,956	CA	C	0.616	0.809	FARP2
rs73004339	2	242,348,171	A	G	0.95	0.994	FARP2
rs76824337	2	242,348,250	G	A	0.907	0.999	FARP2
2:242348614_AT_A	2	242,348,614	AT	A	0.837	0.999	FARP2
rs181334212	2	242,348,660	T	A	0.985	0.99	FARP2
rs7563873	2	242,349,803	A	G	0.776	0.999	FARP2
rs11369953	2	242,349,848	C	CT	0.391	0.96	FARP2
rs28386824	2	242,349,893	A	G	0.813	0.999	FARP2
rs2055566	2	242,350,937	A	G	0.114	0.983	FARP2
rs2074771	2	242,350,944	A	G	0.892	0.999	FARP2
rs2108330	2	242,351,066	A	G	0.214	0.999	FARP2
rs16843642	2	242,351,379	T	G	0.814	0.999	FARP2
rs60604866	2	242,352,257	A	G	0.837	1	FARP2
rs3815262	2	242,352,582	A	T	0.825	0.999	FARP2
rs141753202	2	242,352,633	A	AT	0.893	0.999	FARP2
rs62193220	2	242,353,493	G	A	0.813	0.999	FARP2
rs139336329	2	242,353,655	T	TA	0.985	0.963	FARP2
rs3771561	2	242,353,704	G	A	0.813	0.999	FARP2
rs116292290	2	242,353,940	A	G	0.979	0.998	FARP2
rs77472953	2	242,354,846	C	G	0.972	0.998	FARP2
rs16842449	2	242,354,979	C	T	0.814	0.999	FARP2
rs62193222	2	242,355,406	G	C	0.591	0.96	FARP2
rs202006446	2	242,355,408	G	C	0.953	0.83	FARP2
rs200130230	2	242,355,410	G	C	0.937	0.874	FARP2
2:242355534_CTG_C	2	242,355,534	CTG	C	0.741	0.999	FARP2
rs3771564	2	242,355,701	G	A	0.837	1	FARP2
rs73004354	2	242,356,239	G	T	0.837	1	FARP2

SNP	Chromosome	Position	Effect Allele	Other Allele	Effect Allele Frequency	Imputation quality	Gene
rs142749041	2	242,356,491	G	A	0.986	0.97	FARP2
rs11690176	2	242,356,887	G	A	0.683	0.999	FARP2
rs11679356	2	242,356,894	C	T	0.799	0.998	FARP2
rs34385857	2	242,357,057	A	AT	0.223	0.998	FARP2
2:242357909_GTC_G	2	242,357,909	GTC	G	0.813	0.997	FARP2
rs3771566	2	242,358,299	G	T	0.814	0.999	FARP2
rs10469795	2	242,358,416	A	G	0.776	0.999	FARP2
rs3771567	2	242,358,619	G	A	0.813	0.999	FARP2
rs11693454	2	242,358,940	G	A	0.776	0.999	FARP2
rs3771568	2	242,360,101	A	G	0.907	0.998	FARP2
rs1006309	2	242,360,245	T	G	0.223	0.999	FARP2
2:242361133_GAT_G	2	242,361,133	GAT	G	0.222	0.995	FARP2
rs62193255	2	242,361,298	C	T	0.837	1	FARP2
rs6731054	2	242,361,330	G	A	0.776	0.999	FARP2
rs11675654	2	242,361,597	G	A	0.972	0.995	FARP2
rs6437259	2	242,361,615	A	G	0.223	1	FARP2
rs564645230	2	242,361,817	C	CA	0.97	0.747	FARP2
rs7608676	2	242,361,889	C	T	0.776	0.999	FARP2
rs62193256	2	242,362,168	C	T	0.837	1	FARP2
rs11693232	2	242,362,240	A	G	0.892	1	FARP2
rs113094026	2	242,363,139	C	T	0.907	0.999	FARP2
rs2379199	2	242,363,564	A	C	0.223	1	FARP2
rs4254498	2	242,363,611	G	C	0.814	0.999	FARP2
rs56222556	2	242,364,119	G	A	0.837	1	FARP2
rs10167707	2	242,364,295	T	C	0.223	1	FARP2
rs530395257	2	242,365,342	A	AT	0.988	0.772	FARP2
rs73004362	2	242,365,353	A	T	0.822	0.985	FARP2
rs59715783	2	242,366,262	C	T	0.837	0.999	FARP2
rs62190361	2	242,366,375	C	T	0.814	0.999	FARP2
rs150546758	2	242,366,500	A	G	0.907	0.999	FARP2
rs62190362	2	242,366,604	C	T	0.814	0.998	FARP2
rs73004368	2	242,367,129	T	A	0.946	0.99	FARP2
rs139768259	2	242,367,207	G	A	0.982	0.96	FARP2
rs10195106	2	242,367,402	C	T	0.215	0.995	FARP2
rs59018619	2	242,367,461	A	G	0.986	0.917	FARP2
rs79581922	2	242,367,489	C	T	0.916	0.994	FARP2
rs370381328	2	242,367,649	C	CT	0.518	0.914	FARP2
rs10171117	2	242,367,709	G	A	0.776	0.999	FARP2
2:242367713_AG_A	2	242,367,713	AG	A	0.978	0.992	FARP2
rs149988723	2	242,367,866	C	T	0.983	0.977	FARP2
rs73116394	2	242,367,915	A	G	0.892	1	FARP2
2:242368005_GT_G	2	242,368,005	GT	G	0.814	0.998	FARP2
rs10171440	2	242,368,070	G	A	0.223	1	FARP2
rs141417612	2	242,368,090	T	C	0.989	0.968	FARP2
rs73116396	2	242,368,693	A	G	0.892	1	FARP2
rs79953312	2	242,368,767	C	T	0.968	0.995	FARP2
rs76543564	2	242,369,047	A	G	0.907	0.999	FARP2
rs12612038	2	242,369,685	A	G	0.814	0.999	FARP2
rs59916403	2	242,370,751	G	T	0.647	0.999	FARP2
rs116660098	2	242,370,787	G	A	0.95	0.975	FARP2
rs148538153	2	242,371,245	A	T	0.945	0.989	FARP2
rs113760094	2	242,371,338	G	A	0.814	0.999	FARP2
rs757977	2	242,371,468	T	C	0.214	0.999	FARP2
rs17386555	2	242,371,825	G	A	0.837	0.998	FARP2
rs71951290	2	242,372,544	T	TA	0.82	0.969	FARP2
2:242372918_TATACC_T	2	242,372,918	TATACC	T	0.837	0.999	FARP2
rs35614125	2	242,374,215	C	T	0.776	0.999	FARP2
rs56352948	2	242,374,238	G	A	0.814	0.999	FARP2
rs113453396	2	242,374,248	C	G	0.956	0.996	FARP2
rs16843671	2	242,374,358	T	C	0.814	0.999	FARP2
rs34882686	2	242,374,519	C	T	0.776	0.999	FARP2
rs62190365	2	242,375,008	G	A	0.837	1	FARP2
rs191994647	2	242,375,013	G	A	0.907	0.999	FARP2
rs62190366	2	242,375,429	G	A	0.814	0.999	FARP2
rs7601738	2	242,375,813	A	T	0.892	1	FARP2
rs2074776	2	242,376,186	G	T	0.907	0.999	FARP2
rs62190367	2	242,376,276	G	A	0.837	0.999	FARP2
2:242376499_TACTA_T	2	242,376,499	TACTA	T	0.892	0.999	FARP2
rs929409	2	242,376,507	A	G	0.813	0.996	FARP2
rs6706458	2	242,377,089	T	A	0.817	0.955	FARP2
rs375468916	2	242,377,093	T	A	0.928	0.807	FARP2
rs55990413	2	242,377,174	T	C	0.814	0.999	FARP2
rs6706691	2	242,377,240	T	C	0.214	1	FARP2
rs56107857	2	242,377,356	A	G	0.814	0.999	FARP2
rs115454180	2	242,377,379	C	T	0.982	0.96	FARP2
rs12467294	2	242,377,699	A	G	0.223	1	FARP2
rs189539042	2	242,377,901	G	A	0.974	0.993	FARP2
rs4675958	2	242,377,996	G	T	0.223	1	FARP2
rs73004374	2	242,378,000	T	C	0.813	0.999	FARP2
rs143996503	2	242,378,045	T	G	0.987	0.984	FARP2
rs147264793	2	242,378,053	T	G	0.908	0.996	FARP2
rs139160162	2	242,378,057	T	G	0.908	0.996	FARP2
rs62190368	2	242,378,138	G	A	0.83	0.998	FARP2
rs142107819	2	242,378,593	G	T	0.958	0.973	FARP2
rs138453563	2	242,378,594	C	T	0.958	0.972	FARP2
rs111943976	2	242,378,905	T	C	0.892	1	FARP2
rs2005765	2	242,379,248	G	C	0.124	0.99	FARP2
2:242379522_CCTA_C	2	242,379,522	CCTA	C	0.987	0.864	FARP2
rs17386695	2	242,379,751	G	A	0.837	1	FARP2
rs3755400	2	242,379,795	G	T	0.814	0.999	FARP2
rs57696929	2	242,380,382	T	C	0.814	0.999	FARP2
rs11679955	2	242,382,133	C	T	0.775	0.997	FARP2
rs73004382	2	242,382,192	C	T	0.837	1	FARP2
rs146733519	2	242,382,519	C	T	0.988	0.963	FARP2
rs3771569	2	242,382,606	A	T	0.814	0.999	FARP2
rs3771570	2	242,382,864	C	T	0.837	1	FARP2
rs766683749	2	242,383,583	TTGA	T	0.815	0.997	FARP2
rs929408	2	242,383,928	G	T	0.907	0.999	FARP2
rs62190370	2	242,384,194	C	G	0.837	0.999	FARP2
rs11902896	2	242,384,368	G	A	0.907	0.999	FARP2
rs1142802	2	242,384,581	C	A	0.223	1	FARP2
rs3821281	2	242,385,340	A	G	0.814	0.999	FARP2
rs75005269	2	242,385,726	T	C	0.977	0.983	FARP2
rs7556786	2	242,386,870	C	T	0.776	0.999	FARP2
rs3771573	2	242,387,346	G	A	0.814	0.999	FARP2
rs10192057	2	242,387,984	A	G	0.776	0.999	FARP2
rs62190373	2	242,388,685	G	A	0.837	0.999	FARP2
rs4675813	2	242,388,790	G	T	0.223	1	FARP2
2:242388930_CTT_C	2	242,388,930	CTT	C	0.774	0.993	FARP2
rs55812424	2	242,389,226	A	G	0.814	0.999	FARP2
rs4675955	2	242,389,865	G	T	0.232	0.997	FARP2
rs73006350	2	242,391,062	G	A	0.973	0.965	FARP2
rs140340851	2	242,391,075	A	G	0.969	0.991	FARP2
rs3771577	2	242,391,183	T	G	0.73	0.997	FARP2
rs3771578	2	242,391,212	C	T	0.73	0.998	FARP2
rs113191968	2	242,391,840	A	G	0.979	0.955	FARP2
rs2051964	2	242,392,230	T	C	0.223	0.998	FARP2
rs28446330	2	242,392,759	T	C	0.896	0.988	FARP2
rs77726357	2	242,392,853	T	C	0.694	0.958	FARP2
rs76825718	2	242,392,854	G	A	0.795	0.976	FARP2
rs187443429	2	242,392,862	C	T	0.989	0.672	FARP2
rs193275154	2	242,392,865	G	A	0.987	0.91	FARP2
rs185425703	2	242,392,866	T	C	0.989	0.674	FARP2
rs188651178	2	242,393,018	A	C	0.959	0.973	FARP2
rs546113172	2	242,393,024	G	A	0.962	0.991	FARP2
2:242393182_TG_T	2	242,393,182	TG	T	0.634	0.993	FARP2
2:242393373_AT_A	2	242,393,373	AT	A	0.214	0.996	FARP2
rs2302012	2	242,393,795	G	A	0.665	0.987	FARP2
rs2302011	2	242,393,798	A	G	0.721	0.987	FARP2
rs17387035	2	242,394,003	T	C	0.797	0.998	FARP2
rs764081	2	242,394,141	G	T	0.709	0.996	FARP2
rs2302010	2	242,394,142	G	A	0.785	0.996	FARP2
rs56057909	2	242,395,315	C	T	0.777	0.994	FARP2
rs1468445	2	242,395,554	A	G	0.873	0.998	FARP2
rs6737246	2	242,395,586	T	G	0.798	0.998	FARP2
rs4675812	2	242,395,674	G	A	0.413	0.998	FARP2
rs2074775	2	242,396,033	A	G	0.632	0.998	FARP2
rs578097918	2	242,396,072	T	G	0.827	0.683	FARP2
rs2074774	2	242,396,087	A	T	0.639	0.997	FARP2
rs184603323	2	242,396,146	C	T	0.985	0.93	FARP2
rs886814	2	242,396,666	T	C	0.527	0.935	FARP2
rs886813	2	242,396,693	G	T	0.557	0.93	FARP2
rs886812	2	242,396,694	T	C	0.555	0.967	FARP2
rs28630927	2	242,396,804	T	C	0.703	0.967	FARP2
rs28743050	2	242,396,833	A	G	0.891	0.804	FARP2
rs28604895	2	242,396,838	G	A	0.927	0.781	FARP2
rs12622256	2	242,396,874	A	G	0.382	0.958	FARP2
rs62190378	2	242,397,237	C	T	0.811	0.997	FARP2
rs886811	2	242,397,292	G	A	0.581	0.998	FARP2
rs886810	2	242,397,319	A	C	0.379	0.998	FARP2
rs886809	2	242,397,460	A	G	0.784	0.998	FARP2
rs7597299	2	242,398,074	T	C	0.798	0.999	FARP2
rs201705243	2	242,398,096	C	CA	0.988	0.774	FARP2
rs3771582	2	242,399,275	C	G	0.928	0.996	FARP2
rs3771583	2	242,399,445	A	G	0.928	0.996	FARP2

SNP	Chromosome	Position	Effect Allele	Other Allele	Effect Allele Frequency	Imputation quality	Gene
rs3771584	2	242,399,493	C	G	0.785	0.995	FARP2
rs7585107	2	242,399,518	C	G	0.798	0.999	FARP2
rs71406460	2	242,399,862	C	CT	0.817	0.937	FARP2
rs2010169	2	242,399,863	T	G	0.831	0.999	FARP2
rs757974	2	242,399,927	G	A	0.629	0.999	FARP2
rs35937695	2	242,400,100	A	AG	0.784	0.993	FARP2
rs13388333	2	242,400,597	A	G	0.633	0.999	FARP2
rs3771585	2	242,400,723	G	A	0.831	0.999	FARP2
rs886808	2	242,400,745	C	G	0.214	0.994	FARP2
2:242400748_CCT_C	2	242,400,748	CCT	C	0.835	0.994	FARP2
2:242400847_TC_T	2	242,400,847	TC	T	0.95	0.928	FARP2
rs3771586	2	242,401,002	T	C	0.629	0.999	FARP2
rs62190404	2	242,401,422	C	T	0.784	0.998	FARP2
rs2055569	2	242,401,439	C	G	0.873	0.998	FARP2
rs886807	2	242,401,493	T	G	0.214	0.997	FARP2
rs13011791	2	242,402,061	T	A	0.798	0.999	FARP2
rs4675811	2	242,402,062	A	T	0.831	0.999	FARP2
rs41266961	2	242,402,087	G	C	0.928	0.996	FARP2
rs35319025	2	242,402,463	C	G	0.873	0.999	FARP2
rs10182982	2	242,403,921	T	A	0.557	0.998	FARP2
rs10182990	2	242,403,940	T	G	0.567	0.92	FARP2
rs75917565	2	242,404,003	G	T	0.928	0.991	FARP2
rs10191488	2	242,404,135	G	T	0.632	0.999	FARP2
rs73006377	2	242,404,415	C	G	0.975	0.987	FARP2
rs34123564	2	242,404,437	A	G	0.873	1	FARP2
rs6752327	2	242,404,661	T	C	0.629	0.999	FARP2
rs2240482	2	242,405,021	C	T	0.784	0.998	FARP2
rs62190407	2	242,405,772	G	A	0.831	0.999	FARP2
rs116571422	2	242,406,107	C	T	0.814	0.975	FARP2
rs115979345	2	242,406,108	C	G	0.814	0.974	FARP2
rs55811034	2	242,406,245	C	T	0.835	0.999	FARP2
rs574098642	2	242,406,362	T	TAAA	0.853	0.937	FARP2
rs73006381	2	242,406,654	G	C	0.834	0.999	FARP2
rs112557213	2	242,406,981	C	T	0.928	0.995	FARP2
rs763860057	2	242,407,056	CA	C	0.934	0.754	FARP2
rs13026988	2	242,407,179	G	A	0.798	1	FARP2
rs55764424	2	242,407,467	C	T	0.834	0.999	FARP2
rs10933558	2	242,407,478	A	G	0.798	1	FARP2
rs2240480	2	242,408,219	G	A	0.214	0.997	FARP2
rs570980000	2	242,408,507	G	GT	0.836	0.981	FARP2
rs6711704	2	242,408,981	T	C	0.798	1	FARP2
rs3771587	2	242,409,701	A	G	0.835	0.999	FARP2
rs11682299	2	242,409,895	A	G	0.629	1	FARP2
rs11682333	2	242,409,933	A	G	0.629	1	FARP2
rs77509545	2	242,410,018	C	T	0.928	0.995	FARP2
rs11683555	2	242,410,081	T	C	0.629	1	FARP2
rs11687481	2	242,410,161	G	C	0.798	1	FARP2
rs768166270	2	242,410,257	CAGG	C	0.629	1	FARP2
rs6737152	2	242,410,421	C	G	0.794	1	FARP2
rs62190410	2	242,410,621	C	T	0.834	1	FARP2
rs6707883	2	242,410,784	G	A	0.629	1	FARP2
rs6752480	2	242,410,948	A	G	0.632	1	FARP2
rs111894608	2	242,411,033	C	T	0.799	0.999	FARP2
rs4503983	2	242,411,069	T	C	0.63	0.998	FARP2
rs5016140	2	242,411,071	T	G	0.63	0.998	FARP2
rs4386311	2	242,411,190	A	G	0.629	1	FARP2
rs437022	2	242,411,407	A	G	0.629	1	FARP2
rs6712206	2	242,411,836	G	A	0.629	1	FARP2
rs3771588	2	242,412,109	T	C	0.557	0.999	FARP2
rs10190033	2	242,412,358	A	C	0.829	0.999	FARP2
rs79706799	2	242,412,672	A	G	0.928	0.995	FARP2
2:242413732_CAAAAAAAAAAAAAAAAAAA_C	2	242,413,732	CAAAAAAAAAAAAAAAAAAA	C	0.609	0.945	FARP2
rs149679796	2	242,414,091	C	T	0.977	0.827	FARP2
rs374193358	2	242,414,216	T	TAATA	0.459	0.984	FARP2
rs78318556	2	242,414,531	A	C	0.967	0.975	FARP2
rs10210090	2	242,414,826	G	A	0.798	1	FARP2
rs11684403	2	242,414,874	C	T	0.873	0.999	FARP2
rs13422793	2	242,415,086	T	C	0.798	1	FARP2
rs73006397	2	242,415,523	C	T	0.784	0.997	FARP2
rs10184910	2	242,415,617	A	G	0.629	1	FARP2
rs62190411	2	242,415,872	G	A	0.784	0.997	FARP2
rs10173174	2	242,416,009	C	T	0.629	1	FARP2
rs79378453	2	242,416,361	G	C	0.971	0.976	FARP2
rs115812193	2	242,416,419	T	C	0.972	0.99	FARP2
rs80189034	2	242,416,917	C	G	0.981	0.98	FARP2
rs17453555	2	242,417,808	C	T	0.798	0.999	FARP2
rs62190413	2	242,417,950	G	A	0.834	0.999	FARP2
rs16843765	2	242,418,253	C	G	0.928	0.995	FARP2
rs740314	2	242,418,618	T	C	0.214	0.997	FARP2
rs147476185	2	242,418,673	A	T	0.941	0.994	FARP2
rs740313	2	242,418,808	G	T	0.629	1	FARP2
rs757262277	2	242,419,149	GT	G	0.798	0.999	FARP2
2:242419662_CTCA_C	2	242,419,662	CTCA	C	0.928	0.989	FARP2
2:242419947_GT_G	2	242,419,947	GT	G	0.343	0.992	FARP2
rs75936830	2	242,420,394	G	A	0.928	0.995	FARP2
rs12988792	2	242,420,422	C	G	0.798	1	FARP2
rs3821285	2	242,421,314	G	A	0.831	0.999	FARP2
rs871375	2	242,421,866	G	A	0.343	0.996	FARP2
rs146420876	2	242,422,404	C	T	0.859	0.924	FARP2
rs2012385	2	242,422,405	G	A	0.344	0.995	FARP2
rs73008106	2	242,422,446	C	T	0.849	0.949	FARP2
rs146566663	2	242,423,928	G	A	0.987	0.985	FARP2
rs4675947	2	242,424,110	T	C	0.629	0.999	FARP2
rs62190414	2	242,424,687	A	G	0.784	0.997	FARP2
rs78127357	2	242,425,392	C	T	0.9	0.995	FARP2
rs34505866	2	242,425,585	T	G	0.629	1	FARP2
rs71406464	2	242,425,702	C	CT	0.937	0.902	FARP2
rs60071288	2	242,425,939	C	CATT	0.215	0.99	FARP2
rs11689496	2	242,426,399	G	A	0.873	0.999	FARP2
rs770146527	2	242,426,420	CCT	C	0.798	0.997	FARP2
rs62190415	2	242,426,692	A	G	0.834	0.999	FARP2
rs200798199	2	242,427,081	C	CA	0.3	0.885	FARP2
rs62190416	2	242,427,461	C	G	0.951	0.982	FARP2
rs56934473	2	242,427,581	G	GA	0.784	0.994	FARP2
rs56882309	2	242,428,116	A	G	0.783	0.99	FARP2
rs6745970	2	242,428,513	A	G	0.935	0.989	FARP2
rs4675945	2	242,428,809	G	T	0.629	0.999	FARP2
rs140601953	2	242,428,848	G	A	0.987	0.984	FARP2
rs7607425	2	242,428,910	G	A	0.629	0.999	FARP2
rs35743491	2	242,429,072	C	T	0.798	0.999	FARP2
rs773303298	2	242,429,858	CCCAGCCCAG	C	0.785	0.992	FARP2
rs60988810	2	242,430,084	C	A	0.784	0.997	FARP2
rs56697150	2	242,430,085	C	A	0.784	0.997	FARP2
rs4675944	2	242,430,170	C	T	0.831	0.999	FARP2
rs13405290	2	242,430,245	C	G	0.834	0.999	FARP2
rs869943	2	242,431,585	C	G	0.835	0.999	FARP2
rs76939852	2	242,431,990	C	A	0.928	0.993	FARP2
rs4075818	2	242,432,142	T	C	0.834	0.999	FARP2
rs2271030	2	242,432,704	G	C	0.63	0.999	FARP2
2:242433012_GT_G	2	242,433,012	GT	G	0.662	0.951	FARP2
rs2018761	2	242,433,348	A	G	0.213	0.995	FARP2
rs2163910	3	13,857,892	C	A	0.916	0.872	WNT7A
rs12487265	3	13,858,093	C	G	0.913	0.74	WNT7A
rs1549973	3	13,858,232	G	A	0.377	0.861	WNT7A
rs9840635	3	13,858,241	T	C	0.66	0.857	WNT7A
rs1946620	3	13,858,799	C	T	0.638	0.939	WNT7A
rs77474099	3	13,858,878	G	A	0.846	0.933	WNT7A
rs3796317	3	13,859,027	A	C	0.823	0.949	WNT7A
rs3796316	3	13,859,173	A	C	0.575	0.953	WNT7A
rs3796315	3	13,859,276	G	A	0.989	0.932	WNT7A
rs3796314	3	13,859,332	C	T	0.823	0.953	WNT7A
3:13859709_CCTCT_C	3	13,859,709	CCTCT	C	0.822	0.935	WNT7A
rs575789769	3	13,859,848	C	CAAAAAAAAAAAAAAA	0.562	0.914	WNT7A
rs79020924	3	13,860,957	A	G	0.789	0.961	WNT7A
rs34950685	3	13,861,707	C	T	0.986	0.948	WNT7A
3:13861902_GA_G	3	13,861,902	GA	G	0.415	0.922	WNT7A
rs6795093	3	13,862,399	T	G	0.569	0.97	WNT7A
rs73817629	3	13,862,495	C	T	0.982	0.99	WNT7A
rs2163911	3	13,863,210	A	G	0.385	0.974	WNT7A
rs73017519	3	13,863,501	C	T	0.986	0.96	WNT7A
rs11926637	3	13,863,538	G	A	0.819	0.974	WNT7A
rs4343608	3	13,863,651	C	T	0.798	0.974	WNT7A
rs873853	3	13,864,338	G	A	0.81	0.987	WNT7A
rs6789214	3	13,864,625	C	A	0.97	0.985	WNT7A
rs13326925	3	13,865,044	G	A	0.81	0.991	WNT7A
rs9840696	3	13,865,075	A	G	0.382	0.985	WNT7A
rs61173309	3	13,865,776	C	T	0.988	0.98	WNT7A
rs2341044	3	13,866,145	A	G	0.383	0.992	WNT7A
rs4685039	3	13,866,499	G	A	0.81	0.998	WNT7A
rs4340685	3	13,866,802	C	T	0.81	0.997	WNT7A
rs58015908	3	13,866,984	C	T	0.97	0.994	WNT7A
rs9681737	3	13,867,179	T	C	0.6	0.996	WNT7A
rs12492620	3	13,867,255	A	G	0.789	0.994	WNT7A
rs73017526	3	13,867,579	C	G	0.986	0.977	WNT7A
rs35137353	3	13,867,671	C	T	0.81	0.995	WNT7A

SNP	Chromosome	Position	Effect Allele	Other Allele	Effect Allele Frequency	Imputation quality	Gene
rs80246216	3	13,867,882	C	G	0.88	0.993	WNT7A
rs9819358	3	13,868,090	G	A	0.891	0.998	WNT7A
rs2196030	3	13,868,514	T	C	0.382	0.995	WNT7A
rs62234561	3	13,868,776	C	T	0.683	0.997	WNT7A
rs4380401	3	13,869,083	C	T	0.891	0.998	WNT7A
rs79676987	3	13,869,186	C	G	0.98	0.987	WNT7A
rs867606	3	13,869,301	T	C	0.809	0.992	WNT7A
rs934453	3	13,870,052	T	C	0.891	0.995	WNT7A
rs4685040	3	13,870,101	G	A	0.809	0.991	WNT7A
rs113615561	3	13,870,180	G	A	0.981	0.981	WNT7A
rs12053930	3	13,870,462	T	C	0.795	0.988	WNT7A
rs113163145	3	13,870,727	T	G	0.978	0.945	WNT7A
rs6778046	3	13,871,679	C	T	0.386	0.987	WNT7A
rs112424286	3	13,872,041	C	G	0.982	0.944	WNT7A
rs12634816	3	13,872,270	A	G	0.676	0.989	WNT7A
rs34160038	3	13,872,831	C	A	0.809	0.99	WNT7A
rs734176	3	13,873,451	G	A	0.809	0.991	WNT7A
3:13874150_CT_C	3	13,874,150	CT	C	0.701	0.978	WNT7A
rs115084196	3	13,874,258	A	T	0.982	0.98	WNT7A
rs34620223	3	13,874,639	C	T	0.982	0.994	WNT7A
rs13080685	3	13,875,055	C	T	0.69	0.994	WNT7A
rs62234562	3	13,875,187	G	A	0.971	0.951	WNT7A
rs10510424	3	13,875,328	C	G	0.704	0.995	WNT7A
rs56925963	3	13,876,277	C	T	0.905	0.994	WNT7A
rs78395721	3	13,876,883	G	T	0.987	0.988	WNT7A
rs17038684	3	13,877,133	T	C	0.881	0.997	WNT7A
rs73017597	3	13,877,326	C	T	0.831	0.999	WNT7A
rs4685041	3	13,877,399	T	C	0.794	0.999	WNT7A
rs73017601	3	13,878,261	C	A	0.989	0.934	WNT7A
rs12634112	3	13,878,889	C	T	0.882	0.998	WNT7A
rs752516126	3	13,879,039	A	G	0.985	0.625	WNT7A
rs17038695	3	13,879,203	C	T	0.891	0.989	WNT7A
rs11922919	3	13,879,616	G	T	0.822	0.999	WNT7A
rs76256831	3	13,879,650	T	TG	0.823	0.994	WNT7A
rs73019605	3	13,879,927	G	A	0.987	0.988	WNT7A
rs114693999	3	13,880,251	C	G	0.973	0.991	WNT7A
rs11709521	3	13,880,374	C	T	0.793	0.998	WNT7A
rs73019607	3	13,880,477	C	A	0.987	0.987	WNT7A
rs73019609	3	13,880,690	C	A	0.987	0.987	WNT7A
3:13882211_CTATT_C	3	13,882,211	CTATT	C	0.984	0.945	WNT7A
rs76412067	3	13,882,787	G	A	0.882	0.999	WNT7A
rs62234564	3	13,882,836	A	T	0.831	0.999	WNT7A
rs9862841	3	13,883,197	T	G	0.822	0.999	WNT7A
rs9863149	3	13,883,360	T	C	0.704	1	WNT7A
rs73143994	3	13,883,371	A	G	0.891	0.99	WNT7A
rs764732514	3	13,883,573	CA	C	0.883	0.994	WNT7A
rs62234566	3	13,883,791	G	A	0.691	0.998	WNT7A
rs77229188	3	13,884,343	G	T	0.883	0.998	WNT7A
rs533873345	3	13,885,187	G	GA	0.725	0.982	WNT7A
3:13885243_CT_C	3	13,885,243	CT	C	0.887	0.974	WNT7A
rs538992325	3	13,885,255	T	C	0.884	0.99	WNT7A
rs112984251	3	13,885,260	C	G	0.884	0.988	WNT7A
rs112085630	3	13,885,533	C	T	0.982	0.948	WNT7A
rs75298918	3	13,885,546	G	C	0.882	0.997	WNT7A
rs77362318	3	13,885,859	C	T	0.952	0.991	WNT7A
rs59385529	3	13,886,623	A	G	0.822	0.997	WNT7A
rs934450	3	13,887,729	C	T	0.689	0.995	WNT7A
rs145797134	3	13,887,835	A	G	0.986	0.932	WNT7A
rs73019620	3	13,887,847	G	C	0.987	0.984	WNT7A
rs76372646	3	13,889,153	A	G	0.98	0.991	WNT7A
rs1368577	3	13,889,674	A	G	0.702	0.988	WNT7A
rs1368576	3	13,889,848	G	A	0.44	0.989	WNT7A
rs6800498	3	13,890,273	A	C	0.438	0.987	WNT7A
rs73019626	3	13,890,275	A	C	0.802	0.992	WNT7A
rs76957753	3	13,890,390	C	T	0.987	0.948	WNT7A
rs12492784	3	13,890,485	G	A	0.645	0.987	WNT7A
rs1433355	3	13,890,844	G	T	0.93	0.988	WNT7A
rs4638942	3	13,890,859	A	T	0.929	0.988	WNT7A
rs12493660	3	13,891,361	G	C	0.905	0.996	WNT7A
rs9858382	3	13,891,864	C	T	0.986	0.98	WNT7A
rs56300905	3	13,892,341	G	A	0.718	0.99	WNT7A
rs17038710	3	13,892,543	C	A	0.805	0.993	WNT7A
rs6765373	3	13,892,846	C	T	0.929	0.988	WNT7A
rs73019633	3	13,893,883	G	A	0.874	0.988	WNT7A
rs4257529	3	13,894,116	G	C	0.812	0.987	WNT7A
rs4479574	3	13,894,242	G	T	0.717	0.99	WNT7A
rs3044861	3	13,894,599	A	AGAACCAT	0.43	0.981	WNT7A
rs13095340	3	13,895,139	G	A	0.986	0.985	WNT7A
rs9878521	3	13,895,187	C	A	0.985	0.982	WNT7A
rs34322765	3	13,895,499	C	T	0.986	0.985	WNT7A
rs62234580	3	13,895,542	G	A	0.717	0.99	WNT7A
rs3762721	3	13,895,806	T	C	0.903	0.986	WNT7A
rs3762720	3	13,895,817	C	G	0.425	0.989	WNT7A
rs3762719	3	13,896,140	A	G	0.804	0.994	WNT7A
rs12639607	3	13,896,284	C	T	0.804	0.994	WNT7A
rs13059544	3	13,896,846	A	C	0.711	0.999	WNT7A
rs34390487	3	13,896,871	T	TAA	0.426	0.983	WNT7A
rs1433353	3	13,897,406	A	G	0.711	0.999	WNT7A
rs12486266	3	13,897,410	G	A	0.905	0.996	WNT7A
rs55936644	3	13,897,502	A	G	0.711	0.999	WNT7A
rs9856758	3	13,898,176	A	G	0.711	0.999	WNT7A
rs188934535	3	13,898,377	G	A	0.974	0.796	WNT7A
rs527607046	3	13,898,427	A	G	0.985	0.721	WNT7A
rs547652033	3	13,898,433	A	G	0.988	0.733	WNT7A
rs60907244	3	13,898,463	A	G	0.98	0.641	WNT7A
3:13898475_AAGAAGAAGG_A	3	13,898,475	AAGAAGAAGG	A	0.779	0.881	WNT7A
rs201036014	3	13,898,478	A	G	0.976	0.666	WNT7A
rs57938058	3	13,898,481	A	G	0.953	0.837	WNT7A
rs9819887	3	13,898,618	C	T	0.711	0.999	WNT7A
rs7631306	3	13,898,848	C	G	0.711	0.999	WNT7A
rs7653849	3	13,899,147	A	G	0.088	0.946	WNT7A
rs148287905	3	13,899,275	G	C	0.98	0.978	WNT7A
rs56042123	3	13,901,115	C	A	0.799	0.99	WNT7A
rs12498049	3	13,901,699	G	A	0.906	0.992	WNT7A
rs7635961	3	13,901,791	T	A	0.408	0.983	WNT7A
rs73021628	3	13,901,819	C	G	0.972	0.965	WNT7A
rs9849631	3	13,902,000	A	G	0.93	0.983	WNT7A
rs113066860	3	13,902,253	C	T	0.982	0.944	WNT7A
rs13069140	3	13,903,303	G	A	0.892	0.989	WNT7A
rs7641735	3	13,903,474	T	C	0.925	0.984	WNT7A
rs139405143	3	13,904,118	A	G	0.969	0.963	WNT7A
rs6788472	3	13,904,754	C	T	0.412	0.975	WNT7A
rs9861765	3	13,904,939	A	G	0.925	0.976	WNT7A
rs56347393	3	13,904,940	T	C	0.596	0.972	WNT7A
rs9828013	3	13,905,000	C	T	0.52	0.975	WNT7A
rs6442414	3	13,906,838	A	G	0.406	0.987	WNT7A
rs6795744	3	13,906,850	G	A	0.852	0.987	WNT7A
3:13906916_AT_A	3	13,906,916	AT	A	0.258	0.976	WNT7A
rs112911837	3	13,906,925	C	T	0.853	0.977	WNT7A
rs6442415	3	13,907,066	T	G	0.265	0.988	WNT7A
rs6442416	3	13,907,100	C	T	0.359	0.989	WNT7A
rs6442417	3	13,907,157	T	C	0.265	0.989	WNT7A
rs67138915	3	13,907,483	T	C	0.823	0.99	WNT7A
rs75391621	3	13,907,954	C	T	0.974	0.985	WNT7A
rs9864031	3	13,908,071	T	G	0.823	0.992	WNT7A
rs142957347	3	13,908,109	T	C	0.988	0.952	WNT7A
rs78664077	3	13,908,772	G	A	0.974	0.983	WNT7A
rs150350028	3	13,909,118	A	G	0.968	0.973	WNT7A
rs58454582	3	13,909,858	T	C	0.264	0.991	WNT7A
rs60718855	3	13,909,985	G	A	0.316	0.919	WNT7A
rs547573343	3	13,910,007	G	A	0.947	0.793	WNT7A
3:13910168_AG_A	3	13,910,168	AG	A	0.615	0.881	WNT7A
rs564609945	3	13,910,173	G	A	0.47	0.991	WNT7A
3:13910177_GA_G	3	13,910,177	GA	G	0.47	0.991	WNT7A
3:13910275_GAGGA_G	3	13,910,275	GAGGA	G	0.601	0.88	WNT7A
rs200113153	3	13,910,475	A	G	0.98	0.866	WNT7A
3:13910491_GAA_G	3	13,910,491	GAA	G	0.976	0.93	WNT7A
3:13910509_GAGAGAGAA_G	3	13,910,509	GAGAGAGAA	G	0.983	0.805	WNT7A
rs111064676	3	13,910,660	C	T	0.449	0.981	WNT7A
3:13910780_GAGAA_G	3	13,910,780	GAGAA	G	0.974	0.973	WNT7A
rs111064677	3	13,910,879	G	A	0.263	0.993	WNT7A
rs146414175	3	13,910,936	A	AAAAG	0.418	0.986	WNT7A
rs115177590	3	13,910,946	G	A	0.99	0.696	WNT7A
3:13911067_AAG_A	3	13,911,067	AAG	A	0.984	0.881	WNT7A
rs143524351	3	13,911,190	T	TAGAG	0.478	0.919	WNT7A
3:13911214_GA_G	3	13,911,214	GA	G	0.909	0.961	WNT7A
3:13911219_AAAGG_A	3	13,911,219	AAAGG	A	0.971	0.998	WNT7A
rs73021643	3	13,911,719	T	C	0.967	0.973	WNT7A
rs61187548	3	13,912,123	A	G	0.974	0.985	WNT7A
rs12632968	3	13,912,197	G	A	0.468	0.997	WNT7A
rs12635908	3	13,912,221	T	C	0.442	0.996	WNT7A
rs59835926	3	13,912,413	A	G	0.974	0.986	WNT7A
rs12635960	3	13,912,488	T	C	0.413	0.996	WNT7A
rs11128662	3	13,912,705	T	G	0.468	0.997	WNT7A
rs11128663	3	13,912,850	C	G	0.413	0.996	WNT7A

SNP	Chromosome	Position	Effect Allele	Other Allele	Effect Allele Frequency	Imputation quality	Gene
rs59258584	3	13,913,126	T	C	0.869	0.987	WNT7A
rs60290726	3	13,913,246	A	G	0.869	0.987	WNT7A
rs4685042	3	13,913,277	G	C	0.282	0.99	WNT7A
rs116196588	3	13,913,312	C	T	0.97	0.961	WNT7A
rs74464529	3	13,913,328	C	T	0.974	0.985	WNT7A
rs146931927	3	13,913,375	C	G	0.987	0.949	WNT7A
rs76960233	3	13,913,453	C	G	0.945	0.993	WNT7A
rs78657113	3	13,913,471	C	T	0.974	0.986	WNT7A
rs4685043	3	13,913,521	T	C	0.282	0.991	WNT7A
rs75472006	3	13,913,525	C	T	0.974	0.986	WNT7A
rs77587338	3	13,913,627	C	G	0.986	0.976	WNT7A
rs80020698	3	13,913,756	T	C	0.945	0.993	WNT7A
rs74799154	3	13,913,864	C	T	0.973	0.985	WNT7A
rs59694948	3	13,914,033	C	A	0.968	0.975	WNT7A
rs10865715	3	13,914,440	C	T	0.44	0.998	WNT7A
3:13914506_TTG_T	3	13,914,506	TTG	T	0.44	0.994	WNT7A
rs10865716	3	13,914,684	A	C	0.44	0.999	WNT7A
rs9868948	3	13,914,826	T	G	0.282	0.991	WNT7A
rs9869134	3	13,914,938	T	G	0.282	0.992	WNT7A
rs11128664	3	13,914,976	C	G	0.44	0.999	WNT7A
rs4685045	3	13,915,177	G	C	0.44	0.999	WNT7A
rs4685046	3	13,915,249	G	C	0.44	0.999	WNT7A
rs4684166	3	13,915,465	G	A	0.44	0.999	WNT7A
rs4684167	3	13,915,569	G	A	0.469	0.999	WNT7A
rs62234581	3	13,915,785	A	T	0.443	0.993	WNT7A
rs13090095	3	13,915,787	T	C	0.443	0.993	WNT7A
rs56010638	3	13,915,800	C	A	0.869	0.988	WNT7A
rs56347683	3	13,915,863	G	A	0.842	0.988	WNT7A
rs11128665	3	13,915,884	C	T	0.44	1	WNT7A
rs12497175	3	13,916,089	T	A	0.469	0.999	WNT7A
rs79363893	3	13,916,233	C	CCTCTGTCA	0.442	0.998	WNT7A
rs41284007	3	13,916,794	G	T	0.974	0.985	WNT7A
rs7624679	3	13,917,333	T	C	0.417	0.994	WNT7A
rs13317352	3	13,917,918	A	G	0.844	0.993	WNT7A
rs13318092	3	13,918,034	A	G	0.844	0.991	WNT7A
rs113255057	3	13,918,233	T	TG	0.818	0.986	WNT7A
rs59562904	3	13,918,234	C	G	0.818	0.988	WNT7A
rs79931850	3	13,918,252	G	T	0.974	0.985	WNT7A
rs13433875	3	13,918,381	A	G	0.974	0.985	WNT7A
rs13433727	3	13,918,447	T	G	0.974	0.985	WNT7A
3:13918609_GC_G	3	13,918,609	GC	G	0.973	0.972	WNT7A
rs75106037	3	13,918,751	G	A	0.973	0.985	WNT7A
rs9815077	3	13,918,848	T	C	0.82	0.979	WNT7A
rs9870638	3	13,918,849	G	T	0.819	0.988	WNT7A
rs9832711	3	13,918,956	A	C	0.253	0.971	WNT7A
rs116408986	3	13,919,049	G	T	0.99	0.896	WNT7A
rs11456104	3	13,919,189	T	TC	0.848	0.995	WNT7A
rs9874847	3	13,919,309	C	T	0.858	0.964	WNT7A
rs114106861	3	13,919,310	G	A	0.858	0.965	WNT7A
rs9874850	3	13,919,315	C	T	0.857	0.965	WNT7A
rs66577627	3	13,919,357	C	T	0.85	0.993	WNT7A
rs1836061	3	13,919,551	T	C	0.312	0.988	WNT7A
rs73151668	3	13,920,594	G	T	0.848	0.995	WNT7A
rs2008350	3	13,920,650	A	C	0.974	0.985	WNT7A
rs73023640	3	13,921,032	C	G	0.984	0.986	WNT7A
rs73151669	3	13,921,477	G	T	0.974	0.985	WNT7A
rs4254610	3	24,871,296	C	G	0.143	0.992	RARB
rs78092855	3	24,871,336	C	T	0.866	0.951	RARB
rs114554052	3	24,871,423	G	T	0.963	0.994	RARB
rs6550919	3	24,871,505	A	G	0.143	0.992	RARB
rs139664838	3	24,871,911	C	CGTGTGTGTGTGTGTGT	0.21	0.932	RARB
rs9826602	3	24,871,992	A	T	0.916	0.969	RARB
rs139128351	3	24,872,271	G	C	0.986	0.864	RARB
rs4432599	3	24,872,536	C	T	0.107	0.989	RARB
rs4496443	3	24,872,585	A	T	0.107	0.988	RARB
rs7640730	3	24,872,778	T	G	0.664	0.989	RARB
rs9835858	3	24,873,143	A	T	0.935	0.984	RARB
rs9818530	3	24,873,303	T	C	0.935	0.983	RARB
rs6796944	3	24,873,364	A	G	0.104	0.984	RARB
3:24874118_CAA_C	3	24,874,118	CAA	C	0.665	0.982	RARB
rs1317197	3	24,874,119	A	C	0.673	0.963	RARB
rs4994013	3	24,874,121	T	C	0.665	0.982	RARB
rs75494136	3	24,874,194	A	T	0.963	0.994	RARB
rs142373493	3	24,874,239	G	A	0.987	0.906	RARB
rs1317175	3	24,874,750	C	T	0.963	0.994	RARB
rs1319985	3	24,875,084	A	C	0.107	0.986	RARB
rs9809784	3	24,875,426	G	A	0.527	0.982	RARB
rs75134207	3	24,875,511	A	T	0.934	0.93	RARB
rs114522903	3	24,875,737	T	C	0.963	0.992	RARB
rs201329155	3	24,875,828	G	GT	0.987	0.894	RARB
rs13060345	3	24,875,942	A	C	0.543	0.981	RARB
rs79069309	3	24,875,947	A	G	0.963	0.993	RARB
rs6794654	3	24,876,261	C	T	0.443	0.98	RARB
rs13065331	3	24,876,423	C	T	0.674	0.981	RARB
rs937266	3	24,877,296	G	A	0.667	0.969	RARB
3:24877643_GAT_G	3	24,877,643	GAT	G	0.987	0.859	RARB
rs113884022	3	24,878,561	C	G	0.961	0.965	RARB
rs142281013	3	24,879,126	T	G	0.987	0.95	RARB
rs7615624	3	24,879,239	A	G	0.936	0.983	RARB
rs12634914	3	24,879,499	T	C	0.541	0.961	RARB
rs9831237	3	24,879,598	G	C	0.938	0.979	RARB
rs140433699	3	24,879,604	C	T	0.99	0.989	RARB
rs73033508	3	24,879,843	T	C	0.667	0.955	RARB
rs186387352	3	24,880,183	T	C	0.99	0.991	RARB
rs13079969	3	24,880,255	T	C	0.541	0.958	RARB
rs4858654	3	24,880,542	T	C	0.081	0.946	RARB
3:24881026_GA_G	3	24,881,026	GA	G	0.569	0.877	RARB
rs62228441	3	24,882,037	T	C	0.908	0.959	RARB
rs6550921	3	24,883,013	C	A	0.873	0.969	RARB
rs79007286	3	24,883,209	C	T	0.966	0.989	RARB
rs67530303	3	24,885,336	C	G	0.67	0.955	RARB
rs73820094	3	24,886,717	G	C	0.96	0.971	RARB
rs6783978	3	24,888,172	T	C	0.54	0.997	RARB
rs1994909	3	24,888,242	T	C	0.102	0.974	RARB
rs73045692	3	24,888,822	G	A	0.602	0.988	RARB
rs74433766	3	24,889,399	G	A	0.98	0.974	RARB
rs868569	3	24,889,632	C	G	0.582	0.992	RARB
rs77030106	3	24,889,856	A	G	0.969	0.974	RARB
rs10865798	3	24,891,014	T	C	0.373	0.977	RARB
rs74548634	3	24,891,416	G	A	0.977	0.988	RARB
3:24892121_TA_T	3	24,892,121	TA	T	0.968	0.974	RARB
rs73152442	3	24,892,556	G	C	0.961	0.959	RARB
rs116238698	3	24,892,990	G	A	0.962	0.961	RARB
rs115439358	3	24,893,096	C	T	0.962	0.96	RARB
rs2068901	3	24,893,218	A	T	0.58	0.982	RARB
rs13067118	3	24,894,242	C	T	0.581	0.983	RARB
rs139922270	3	24,895,282	C	T	0.975	0.947	RARB
rs2362767	3	24,895,707	G	C	0.391	0.984	RARB
rs9864319	3	24,896,058	C	T	0.391	0.985	RARB
rs114901110	3	24,896,293	A	G	0.962	0.964	RARB
rs116324006	3	24,896,537	C	T	0.962	0.965	RARB
rs9832148	3	24,897,268	A	T	0.39	0.979	RARB
3:24897554_TACA_T	3	24,897,554	TACA	T	0.582	0.974	RARB
rs12638345	3	24,898,233	C	T	0.415	0.984	RARB
rs9880358	3	24,899,123	C	T	0.415	0.985	RARB
rs115013261	3	24,899,575	T	C	0.962	0.965	RARB
rs143623009	3	24,900,422	G	A	0.962	0.965	RARB
3:24900549_GA_G	3	24,900,549	GA	G	0.387	0.887	RARB
rs13095442	3	24,901,018	T	C	0.572	0.985	RARB
rs62228443	3	24,901,361	T	C	0.863	0.996	RARB
rs62228444	3	24,901,418	C	T	0.865	0.996	RARB
rs11709242	3	24,901,632	A	G	0.572	0.985	RARB
rs11709276	3	24,901,731	A	T	0.573	0.985	RARB
rs111324276	3	24,901,881	A	T	0.962	0.965	RARB
rs13064710	3	24,902,942	T	G	0.578	0.985	RARB
rs115434521	3	24,902,979	G	A	0.989	0.882	RARB
3:24903085_TAAAAA_T	3	24,903,085	TAAAAA	T	0.374	0.983	RARB
rs13089297	3	24,903,138	C	T	0.579	0.985	RARB
rs7651101	3	24,903,229	G	A	0.973	0.98	RARB
rs763155725	3	24,903,329	AG	A	0.975	0.976	RARB
rs28514537	3	24,903,424	G	C	0.973	0.979	RARB
rs937262	3	24,903,770	A	C	0.411	0.986	RARB
rs116582860	3	24,903,776	A	G	0.962	0.965	RARB
rs1319984	3	24,903,913	C	T	0.376	0.987	RARB
rs937263	3	24,904,212	A	G	0.962	0.961	RARB
rs937264	3	24,904,213	C	T	0.962	0.961	RARB
rs7610607	3	24,904,904	G	A	0.798	0.981	RARB
rs146168306	3	24,905,100	G	A	0.987	0.97	RARB
rs202245619	3	24,905,335	G	GC	0.961	0.947	RARB
rs112512729	3	24,905,460	T	G	0.929	0.963	RARB
rs147061118	3	24,905,995	C	T	0.979	0.915	RARB
3:24906213_AT_A	3	24,906,213	AT	A	0.358	0.971	RARB
rs17787403	3	24,906,406	C	T	0.95	0.975	RARB
rs116183338	3	24,906,492	A	G	0.96	0.961	RARB
rs1994905	3	24,906,827	A	G	0.373	0.986	RARB

SNP	Chromosome	Position	Effect Allele	Other Allele	Effect Allele Frequency	Imputation quality	Gene
rs11707113	3	24,906,858	G	A	0.745	0.945	RARB
rs1994906	3	24,906,906	A	G	0.373	0.987	RARB
rs12635876	3	24,907,242	A	G	0.862	0.988	RARB
rs114660818	3	24,907,502	A	G	0.96	0.962	RARB
rs34606772	3	24,908,376	C	T	0.58	0.982	RARB
rs79233370	3	24,908,464	T	G	0.969	0.962	RARB
rs116268817	3	24,908,813	A	C	0.97	0.951	RARB
rs113792544	3	24,908,816	G	A	0.975	0.981	RARB
rs114956412	3	24,908,821	T	G	0.97	0.951	RARB
rs2885593	3	24,909,003	C	T	0.37	0.991	RARB
rs2885594	3	24,909,090	G	T	0.4	0.988	RARB
rs78846640	3	24,909,274	T	G	0.969	0.963	RARB
rs76897961	3	24,909,785	G	A	0.989	0.985	RARB
rs191020764	3	24,910,288	A	G	0.989	0.926	RARB
rs115532694	3	24,910,449	C	G	0.969	0.963	RARB
rs9310762	3	24,911,917	A	G	0.89	0.992	RARB
rs116581474	3	24,912,598	T	G	0.969	0.964	RARB
rs9856392	3	24,912,716	A	G	0.891	0.993	RARB
rs4521187	3	24,912,782	A	G	0.974	0.983	RARB
rs11921453	3	24,913,343	T	C	0.974	0.983	RARB
rs75607755	3	24,913,677	C	A	0.984	0.975	RARB
rs2362770	3	24,913,724	G	C	0.364	0.995	RARB
rs2362771	3	24,913,845	T	C	0.362	0.995	RARB
rs4507219	3	24,913,853	G	A	0.97	0.965	RARB
rs116667029	3	24,914,214	C	A	0.97	0.965	RARB
rs77822947	3	24,914,378	C	T	0.97	0.965	RARB
rs11129167	3	24,914,550	A	G	0.403	0.992	RARB
rs77749050	3	24,914,575	G	A	0.989	0.99	RARB
3:24914796_CTCATTTAACT_C	3	24,914,796	CTCATTTAACT	C	0.978	0.893	RARB
rs76826802	3	24,915,137	G	A	0.959	0.97	RARB
rs9854078	3	24,915,283	T	C	0.363	0.997	RARB
rs12715063	3	24,915,790	A	G	0.361	0.997	RARB
rs116007039	3	24,916,298	A	G	0.972	0.99	RARB
rs6777344	3	24,916,595	T	C	0.958	0.971	RARB
rs2885595	3	24,917,378	C	A	0.535	0.991	RARB
rs113316605	3	24,917,479	C	T	0.968	0.967	RARB
rs2885596	3	24,917,498	G	A	0.145	0.984	RARB
rs78436026	3	24,917,666	C	T	0.968	0.966	RARB
rs79561482	3	24,917,803	A	G	0.975	0.989	RARB
rs62228445	3	24,917,970	G	C	0.984	0.967	RARB
rs10510549	3	24,918,190	A	G	0.989	0.988	RARB
rs2362772	3	24,918,232	C	A	0.943	0.976	RARB
rs7629624	3	24,918,441	T	C	0.989	0.99	RARB
rs76200377	3	24,918,442	G	T	0.968	0.967	RARB
rs17015371	3	24,918,765	G	A	0.975	0.99	RARB
rs116210311	3	24,918,828	G	A	0.971	0.899	RARB
rs761559168	3	24,919,002	CCTA	C	0.969	0.956	RARB
rs12493197	3	24,919,200	C	G	0.648	0.979	RARB
rs115270783	3	24,919,521	C	T	0.968	0.964	RARB
3:24919701_ATT_A	3	24,919,701	ATT	A	0.391	0.99	RARB
3:24919713_TTTG_T	3	24,919,713	TTTG	T	0.9	0.628	RARB
rs553342983	3	24,919,853	A	AT	0.984	0.884	RARB
rs2362773	3	24,920,080	G	A	0.989	0.99	RARB
rs2362774	3	24,920,083	A	G	0.362	0.996	RARB
rs76163765	3	24,920,435	T	C	0.975	0.99	RARB
rs7622169	3	24,920,520	C	A	0.904	0.997	RARB
rs7633613	3	24,920,557	A	T	0.931	0.977	RARB
rs7636108	3	24,920,601	T	C	0.903	0.997	RARB
rs149326661	3	24,920,732	A	G	0.968	0.966	RARB
rs11417503	3	24,920,925	T	TA	0.431	0.988	RARB
rs200111736	3	24,920,995	C	T	0.796	0.562	RARB
rs4342074	3	24,921,071	C	G	0.527	0.991	RARB
rs138020469	3	24,921,165	A	C	0.968	0.966	RARB
rs11711711	3	24,921,207	G	A	0.975	0.991	RARB
rs11920399	3	24,921,407	G	A	0.975	0.99	RARB
3:24921439_TTG_T	3	24,921,439	TTG	T	0.911	0.992	RARB
rs10865799	3	24,921,468	C	T	0.932	0.979	RARB
rs10865800	3	24,921,483	C	A	0.932	0.968	RARB
rs11711907	3	24,921,767	G	A	0.975	0.991	RARB
rs11711882	3	24,921,858	C	T	0.964	0.991	RARB
rs74448439	3	24,922,192	T	C	0.975	0.99	RARB
rs11921445	3	24,922,506	C	T	0.907	0.995	RARB
rs114736675	3	24,922,573	C	T	0.968	0.965	RARB
rs745752684	3	24,922,798	ACAAGAT	A	0.989	0.982	RARB
rs116695205	3	24,922,884	G	C	0.968	0.966	RARB
rs4858659	3	24,922,896	A	G	0.525	0.99	RARB
rs11129169	3	24,923,743	G	A	0.79	0.984	RARB
rs74278553	3	24,923,883	A	G	0.957	0.971	RARB
rs145266239	3	24,924,130	T	TA	0.969	0.956	RARB
rs2362775	3	24,924,421	T	C	0.525	0.989	RARB
rs6550923	3	24,924,495	G	A	0.638	0.986	RARB
rs11715275	3	24,924,970	G	A	0.975	0.989	RARB
rs115463265	3	24,925,070	C	T	0.968	0.966	RARB
rs144651876	3	24,925,183	A	T	0.984	0.916	RARB
rs2885597	3	24,925,595	T	G	0.362	0.994	RARB
rs148825795	3	24,925,982	A	AT	0.969	0.957	RARB
rs2362776	3	24,926,114	C	G	0.362	0.994	RARB
rs147933117	3	24,926,166	C	A	0.987	0.956	RARB
rs74757562	3	24,926,240	G	A	0.958	0.979	RARB
rs79110116	3	24,926,401	A	G	0.975	0.99	RARB
rs10510550	3	24,926,454	T	C	0.975	0.99	RARB
rs10510551	3	24,927,203	T	C	0.523	0.989	RARB
rs7340729	3	24,927,540	G	C	0.988	0.99	RARB
rs1874053	3	24,927,590	C	T	0.975	0.988	RARB
rs2362777	3	24,927,919	T	G	0.36	0.994	RARB
rs548167872	3	24,927,938	C	CT	0.46	0.886	RARB
rs79777976	3	24,928,048	T	G	0.968	0.965	RARB
rs114004619	3	24,928,085	G	A	0.968	0.965	RARB
rs77130072	3	24,928,241	G	T	0.975	0.993	RARB
rs10510552	3	24,928,286	T	C	0.492	0.993	RARB
rs9819866	3	24,928,351	T	C	0.906	0.992	RARB
rs114646344	3	24,928,399	G	T	0.968	0.965	RARB
rs4858131	3	24,928,564	G	C	0.363	0.994	RARB
rs114429523	3	24,928,804	A	G	0.968	0.964	RARB
rs17787433	3	24,929,152	T	C	0.524	0.989	RARB
rs34562559	3	24,929,198	G	C	0.482	0.992	RARB
rs10865801	3	24,929,802	C	A	0.499	0.992	RARB
rs6804057	3	24,929,990	A	G	0.903	0.99	RARB
rs2362778	3	24,930,241	A	G	0.361	0.994	RARB
rs2885598	3	24,930,256	T	C	0.989	0.996	RARB
rs115721835	3	24,931,048	C	G	0.969	0.964	RARB
rs4858661	3	24,931,083	C	A	0.365	0.994	RARB
rs79052348	3	24,931,092	T	C	0.989	0.996	RARB
rs561646651	3	24,931,173	G	GTGTGTC	0.974	0.928	RARB
rs9883880	3	24,931,873	T	C	0.364	0.994	RARB
rs1874054	3	24,931,889	T	C	0.361	0.992	RARB
rs113210748	3	24,932,339	T	TA	0.986	0.879	RARB
rs4622866	3	24,932,349	T	A	0.874	0.984	RARB
rs4269051	3	24,932,435	G	A	0.975	0.991	RARB
rs115776393	3	24,933,037	C	G	0.969	0.967	RARB
rs4270472	3	24,933,086	C	T	0.368	0.993	RARB
rs17195038	3	24,933,191	A	G	0.534	0.989	RARB
rs6550924	3	24,933,275	A	C	0.368	0.993	RARB
rs57461799	3	24,933,299	A	G	0.534	0.988	RARB
rs11711279	3	24,933,325	A	G	0.974	0.988	RARB
3:24933367_AC_A	3	24,933,367	AC	A	0.535	0.985	RARB
rs7646119	3	24,933,966	T	C	0.368	0.993	RARB
rs13095194	3	24,934,201	A	T	0.531	0.989	RARB
rs7644137	3	24,934,399	A	G	0.989	0.993	RARB
rs9879771	3	24,934,809	C	T	0.906	0.99	RARB
rs11129170	3	24,934,827	T	C	0.913	0.991	RARB
rs78895054	3	24,934,871	C	T	0.979	0.917	RARB
rs115663997	3	24,934,887	G	C	0.969	0.967	RARB
3:24935175_AT_A	3	24,935,175	AT	A	0.769	0.864	RARB
rs114165594	3	24,935,200	G	A	0.968	0.968	RARB
3:24935221_GCCT_G	3	24,935,221	GCCT	G	0.989	0.713	RARB
rs9754535	3	24,935,229	G	A	0.367	0.993	RARB
3:24935502_TAACTG_T	3	24,935,502	TAACTG	T	0.906	0.983	RARB
rs2885802	3	24,935,664	A	C	0.969	0.968	RARB
rs13059636	3	24,935,790	A	G	0.526	0.989	RARB
rs11376602	3	24,937,048	C	CTT	0.969	0.957	RARB
rs5847323	3	24,937,115	T	TA	0.475	0.981	RARB
rs565853556	3	24,937,118	T	C	0.987	0.968	RARB
rs79534532	3	24,937,119	C	A	0.906	0.982	RARB
rs11916565	3	24,937,499	C	T	0.975	0.995	RARB
rs11924369	3	24,937,733	A	G	0.974	0.989	RARB
rs35018962	3	24,937,966	G	A	0.907	0.989	RARB
rs186520943	3	24,937,985	C	T	0.968	0.967	RARB
rs2885803	3	24,938,117	A	G	0.374	0.994	RARB
rs11709004	3	24,938,210	C	G	0.975	0.995	RARB
rs62230565	3	24,938,652	C	T	0.984	0.959	RARB
rs2363515	3	24,938,673	G	T	0.408	0.99	RARB
rs2885804	3	24,938,807	C	T	0.365	0.994	RARB
rs115438030	3	24,938,988	C	G	0.968	0.967	RARB
rs7617593	3	24,939,057	T	G	0.365	0.994	RARB

SNP	Chromosome	Position	Effect Allele	Other Allele	Effect Allele Frequency	Imputation quality	Gene
rs17015422	3	24,939,147	C	T	0.986	0.985	RARB
rs71965007	3	24,939,243	C	CT	0.398	0.916	RARB
rs77820348	3	24,939,471	G	A	0.976	0.969	RARB
rs11718085	3	24,939,786	A	C	0.986	0.994	RARB
3:24939812_TATA_T	3	24,939,812	TATA	T	0.974	0.962	RARB
rs78028828	3	24,940,010	T	C	0.964	0.922	RARB
rs116116275	3	24,940,061	G	A	0.969	0.968	RARB
rs17015426	3	24,940,074	T	C	0.989	0.99	RARB
rs7620738	3	24,940,115	T	C	0.974	0.991	RARB
rs2363516	3	24,940,175	G	A	0.325	0.993	RARB
rs7651471	3	24,940,236	C	T	0.974	0.991	RARB
rs9883020	3	24,940,495	C	T	0.361	0.993	RARB
rs73047672	3	24,940,850	C	A	0.881	0.946	RARB
rs138887308	3	24,940,866	G	A	0.989	0.861	RARB
rs5847324	3	24,941,415	C	CT	0.364	0.987	RARB
rs937265	3	24,941,613	C	T	0.294	0.999	RARB
rs2363518	3	24,942,213	G	A	0.31	0.993	RARB
rs2068192	3	24,942,605	T	A	0.834	0.987	RARB
rs11129171	3	24,943,104	T	C	0.528	0.989	RARB
rs116266233	3	24,943,338	G	T	0.969	0.968	RARB
rs111594275	3	24,943,893	G	GT	0.988	0.908	RARB
rs6769702	3	24,943,995	T	C	0.834	0.987	RARB
rs76638371	3	24,944,223	T	A	0.989	0.988	RARB
rs76131459	3	24,944,321	G	A	0.989	0.983	RARB
rs6770148	3	24,944,412	T	A	0.944	0.981	RARB
rs200317935	3	24,944,498	A	AAG	0.982	0.814	RARB
rs7617167	3	24,944,555	T	G	0.834	0.987	RARB
rs13062430	3	24,945,017	T	C	0.521	0.985	RARB
rs6550926	3	24,945,215	C	T	0.288	0.993	RARB
rs11927180	3	24,945,318	A	C	0.975	0.998	RARB
rs62230566	3	24,945,568	G	A	0.841	0.987	RARB
rs115146931	3	24,945,665	C	T	0.985	0.893	RARB
rs111423152	3	24,945,886	A	G	0.989	0.988	RARB
rs140575625	3	24,946,223	G	C	0.968	0.969	RARB
rs13093492	3	24,946,421	C	T	0.517	0.978	RARB
rs114621115	3	24,946,517	A	G	0.968	0.969	RARB
rs138066423	3	24,946,533	G	A	0.987	0.959	RARB
rs115671570	3	24,947,259	A	C	0.968	0.968	RARB
rs2363519	3	24,947,323	C	T	0.016	0.9	RARB
rs115237808	3	24,948,048	C	T	0.984	0.937	RARB
rs141243485	3	24,948,104	C	T	0.975	0.998	RARB
rs2363520	3	24,948,515	C	T	0.496	0.991	RARB
rs17015441	3	24,948,569	A	G	0.989	0.988	RARB
rs76507337	3	24,948,625	C	A	0.989	0.987	RARB
rs4858132	3	24,948,990	T	C	0.293	0.996	RARB
rs6784756	3	24,949,327	A	C	0.798	0.987	RARB
rs6772205	3	24,949,391	C	T	0.293	0.995	RARB
rs6796065	3	24,949,392	G	T	0.293	0.995	RARB
rs116492817	3	24,949,399	C	T	0.987	0.901	RARB
rs6787501	3	24,949,432	T	C	0.293	0.995	RARB
rs75663988	3	24,949,623	G	T	0.98	0.929	RARB
rs74430411	3	24,950,017	A	G	0.968	0.969	RARB
rs59436851	3	24,950,188	A	G	0.989	0.985	RARB
rs13085130	3	24,950,251	A	C	0.293	0.993	RARB
rs13085461	3	24,950,387	C	G	0.465	0.987	RARB
rs60757912	3	24,950,496	A	T	0.834	0.985	RARB
rs59964722	3	24,950,518	A	G	0.968	0.965	RARB
rs6791253	3	24,950,567	T	C	0.963	0.986	RARB
rs114103185	3	24,950,732	A	G	0.987	0.978	RARB
rs116349242	3	24,951,156	C	G	0.982	0.913	RARB
rs75462943	3	24,951,336	G	A	0.975	0.987	RARB
rs6807549	3	24,951,404	T	G	0.302	0.989	RARB
rs34733520	3	24,951,426	G	A	0.528	0.983	RARB
rs115832749	3	24,951,438	A	G	0.969	0.967	RARB
rs35357706	3	24,951,592	T	C	0.526	0.983	RARB
rs77743430	3	24,951,744	A	G	0.975	0.985	RARB
rs4568101	3	24,951,858	A	C	0.329	0.988	RARB
rs1994907	3	24,952,076	G	A	0.362	0.984	RARB
rs4858663	3	24,953,025	C	G	0.989	0.939	RARB
3:24953029_GTTTTTA_G	3	24,953,029	GTTTTTA	G	0.968	0.955	RARB
rs2363521	3	24,953,417	A	T	0.528	0.978	RARB
rs35913336	3	24,953,489	C	T	0.841	0.981	RARB
rs11714001	3	24,953,512	G	A	0.948	0.98	RARB
rs765654701	3	24,953,896	CTGGTACACAGTAAAATCTCAATAAG	C	0.989	0.952	RARB
rs67922835	3	24,954,294	C	G	0.625	0.968	RARB
rs13067108	3	24,954,320	G	A	0.489	0.972	RARB
rs13067142	3	24,954,466	C	T	0.977	0.589	RARB
rs144144225	3	24,954,502	C	T	0.975	0.951	RARB
rs112600300	3	24,954,648	T	C	0.903	0.93	RARB
rs9854321	3	24,954,687	C	A	0.99	0.97	RARB
rs147074988	3	24,954,711	C	G	0.971	0.963	RARB
rs138287624	3	24,954,765	G	A	0.985	0.943	RARB
rs77878264	3	24,954,771	A	G	0.989	0.968	RARB
rs143872173	3	24,954,846	A	G	0.977	0.983	RARB
rs200994631	3	24,954,894	T	TCTC	0.802	0.948	RARB
rs73823035	3	24,954,895	T	C	0.808	0.946	RARB
rs66779362	3	24,954,896	T	TC	0.498	0.965	RARB
rs35781969	3	24,955,022	T	C	0.793	0.981	RARB
rs73049464	3	24,955,518	G	A	0.718	0.984	RARB
rs35342260	3	24,955,790	A	G	0.507	0.99	RARB
rs9808989	3	24,956,222	T	C	0.443	0.989	RARB
rs115900404	3	24,956,484	T	A	0.977	0.972	RARB
rs9827612	3	24,956,953	A	G	0.442	0.994	RARB
rs9810222	3	24,956,972	T	G	0.441	0.994	RARB
rs9827642	3	24,957,017	A	G	0.441	0.995	RARB
rs13086160	3	24,957,994	C	T	0.715	0.994	RARB
rs75133559	3	24,958,007	T	C	0.943	0.985	RARB
rs563304522	3	24,958,258	C	CAA	0.562	0.897	RARB
rs13091209	3	24,958,459	A	G	0.823	0.992	RARB
rs116060469	3	24,958,725	T	G	0.976	0.99	RARB
rs73049478	3	24,958,946	A	G	0.942	0.985	RARB
rs60998309	3	24,959,272	C	A	0.943	0.986	RARB
rs9843036	3	24,959,603	A	G	0.443	0.994	RARB
rs73049480	3	24,959,615	G	C	0.944	0.987	RARB
rs11712813	3	24,959,651	A	G	0.471	0.995	RARB
rs9881002	3	24,959,693	C	A	0.478	0.995	RARB
rs35999749	3	24,959,873	G	C	0.813	0.992	RARB
rs143809073	3	24,960,090	A	G	0.98	0.952	RARB
rs150547926	3	24,960,770	G	T	0.988	0.887	RARB
rs56761866	3	24,960,824	C	T	0.945	0.986	RARB
rs6775359	3	24,960,868	C	G	0.754	0.99	RARB
rs36025902	3	24,960,890	T	A	0.715	0.994	RARB
rs6772273	3	24,961,039	A	G	0.443	0.994	RARB
rs6774983	3	24,961,167	T	C	0.468	0.995	RARB
rs6772493	3	24,961,293	A	G	0.474	0.993	RARB
rs76836904	3	24,961,474	G	A	0.955	0.986	RARB
rs13326208	3	24,961,849	A	G	0.508	0.994	RARB
rs60488460	3	24,961,890	T	G	0.955	0.985	RARB
3:24962064_AAG_A	3	24,962,064	AAG	A	0.832	0.979	RARB
rs11716615	3	24,962,071	T	G	0.117	0.961	RARB
rs13096040	3	24,962,306	A	G	0.7	0.991	RARB
rs2363522	3	24,962,684	A	T	0.989	0.99	RARB
rs11716868	3	24,962,928	T	G	0.746	0.993	RARB
rs9846880	3	24,963,067	C	G	0.57	0.995	RARB
rs73049488	3	24,963,081	T	C	0.955	0.983	RARB
rs11713674	3	24,963,383	C	T	0.746	0.993	RARB
rs17015448	3	24,963,629	T	C	0.954	0.987	RARB
rs6779838	3	24,963,711	A	G	0.481	0.993	RARB
rs6782972	3	24,964,350	A	G	0.786	0.993	RARB
rs79717129	3	24,964,428	G	C	0.989	0.99	RARB
rs148226184	3	24,964,473	A	G	0.974	0.995	RARB
rs115138986	3	24,964,615	A	G	0.978	0.976	RARB
rs17015451	3	24,964,676	A	G	0.811	0.993	RARB
rs73049492	3	24,964,709	G	A	0.955	0.987	RARB
rs140874856	3	24,964,781	C	CT	0.822	0.991	RARB
rs2363523	3	24,965,287	T	A	0.511	0.995	RARB
rs4456829	3	24,966,406	C	T	0.989	0.993	RARB
rs12636772	3	24,966,419	G	A	0.746	0.995	RARB
rs115776289	3	24,966,587	A	T	0.974	0.996	RARB
rs12636778	3	24,966,590	C	T	0.747	0.995	RARB
rs10510553	3	24,966,768	T	C	0.959	0.989	RARB
rs9310765	3	24,967,044	C	G	0.482	0.995	RARB
rs564076072	3	24,967,134	C	CT	0.631	0.93	RARB
rs74891453	3	24,967,618	A	T	0.989	0.994	RARB
3:24967801_TAGGGTAC_T	3	24,967,801	TAGGGTAC	T	0.828	0.982	RARB
3:24967801_TAGGG_T	3	24,967,801	TAGGG	T	0.824	0.994	RARB
3:24967802_AGGGTAC_A	3	24,967,802	AGGGTAC	A	0.96	0.621	RARB
3:24967806_TAC_T	3	24,967,806	TAC	T	0.824	0.994	RARB
rs79906988	3	24,967,925	T	G	0.989	0.994	RARB
rs17015467	3	24,967,955	C	G	0.955	0.989	RARB
rs141801104	3	24,968,251	T	TG	0.989	0.98	RARB
rs73051205	3	24,968,560	C	G	0.955	0.99	RARB
rs17015473	3	24,968,678	T	C	0.989	0.994	RARB
rs12638313	3	24,968,752	G	C	0.519	0.995	RARB
rs73051208	3	24,968,871	C	T	0.956	0.99	RARB

SNP	Chromosome	Position	Effect Allele	Other Allele	Effect Allele Frequency	Imputation quality	Gene
rs17787464	3	24,969,023	A	G	0.83	0.997	RARB
rs148031463	3	24,969,522	G	A	0.987	0.96	RARB
rs73051211	3	24,969,605	T	C	0.956	0.991	RARB
rs17015482	3	24,969,655	G	C	0.989	0.995	RARB
rs116111357	3	24,970,018	T	A	0.989	0.995	RARB
rs35867841	3	24,970,025	T	C	0.823	0.997	RARB
rs114840867	3	24,970,820	A	G	0.989	0.905	RARB
rs34125616	3	24,971,292	T	A	0.985	0.94	RARB
rs80062089	3	24,971,294	C	T	0.987	0.973	RARB
rs2363524	3	24,971,540	C	T	0.493	0.997	RARB
rs747187769	3	24,971,818	CG	C	0.989	0.989	RARB
rs2363525	3	24,971,843	G	A	0.531	0.997	RARB
rs79419997	3	24,972,052	C	G	0.989	0.996	RARB
rs60936546	3	24,972,305	G	C	0.955	0.991	RARB
rs61176331	3	24,972,338	C	A	0.955	0.991	RARB
rs12637240	3	24,972,398	C	T	0.747	0.997	RARB
rs2363526	3	24,972,416	C	A	0.493	0.997	RARB
rs59986678	3	24,972,582	G	T	0.955	0.991	RARB
rs6795387	3	24,972,665	T	C	0.747	0.997	RARB
rs2363527	3	24,972,792	T	C	0.828	0.998	RARB
rs2363528	3	24,972,894	T	A	0.493	0.997	RARB
rs4368469	3	24,973,043	A	C	0.53	0.997	RARB
rs58727158	3	24,973,495	T	C	0.955	0.991	RARB
rs9872534	3	24,973,500	C	G	0.529	0.997	RARB
rs77286698	3	24,973,564	C	CT	0.955	0.987	RARB
rs146345404	3	24,973,643	G	A	0.974	0.997	RARB
rs200416235	3	24,974,353	A	AT	0.974	0.996	RARB
rs4858665	3	24,974,644	A	T	0.493	0.998	RARB
rs139999549	3	24,974,753	A	G	0.974	0.998	RARB
rs13074839	3	24,974,849	T	C	0.746	0.997	RARB
rs9878781	3	24,974,882	G	A	0.53	0.998	RARB
rs115124081	3	24,974,999	C	T	0.989	0.997	RARB
rs78809919	3	24,975,009	C	T	0.971	0.996	RARB
rs73051231	3	24,975,015	C	T	0.957	0.993	RARB
rs12330318	3	24,975,207	T	G	0.524	0.998	RARB
rs12330519	3	24,975,241	A	G	0.53	0.998	RARB
rs9845970	3	24,975,828	A	T	0.519	0.998	RARB
rs56881270	3	24,975,841	T	C	0.785	0.998	RARB
rs9883836	3	24,975,890	C	T	0.493	0.998	RARB
rs9812847	3	24,976,080	G	A	0.518	0.998	RARB
rs76091220	3	24,976,317	A	C	0.989	0.997	RARB
rs9818150	3	24,977,259	C	T	0.015	0.91	RARB
rs114648224	3	24,977,261	T	A	0.974	0.998	RARB
rs75862768	3	24,977,356	T	G	0.974	0.998	RARB
rs2363529	3	24,977,639	T	C	0.487	0.997	RARB
rs13070123	3	24,977,884	A	G	0.785	0.998	RARB
rs12715064	3	24,977,922	C	T	0.529	0.998	RARB
rs199745367	3	24,978,268	A	AAT	0.974	0.996	RARB
rs59570870	3	24,978,711	T	C	0.785	0.998	RARB
rs73820319	3	24,978,766	T	C	0.988	0.992	RARB
rs57982938	3	24,979,087	T	C	0.786	0.998	RARB
rs115223511	3	24,979,089	C	A	0.989	0.997	RARB
rs2363530	3	24,979,142	C	T	0.487	0.998	RARB
rs115234072	3	24,979,384	A	G	0.974	0.998	RARB
3:24979532_TGC_T	3	24,979,532	TGC	T	0.515	0.996	RARB
rs11710017	3	24,979,746	C	A	0.489	0.998	RARB
rs79141242	3	24,979,807	T	C	0.974	0.998	RARB
rs75547993	3	24,980,122	C	T	0.989	0.998	RARB
rs115954774	3	24,980,352	T	C	0.989	0.997	RARB
rs9843826	3	24,980,450	A	T	0.489	0.999	RARB
rs71057691	3	24,980,483	T	TG	0.988	0.974	RARB
rs116076985	3	24,980,535	G	A	0.989	0.998	RARB
rs147108206	3	24,980,576	C	T	0.956	0.993	RARB
rs9881799	3	24,980,618	C	T	0.501	0.999	RARB
rs9882350	3	24,980,763	G	A	0.528	0.999	RARB
rs13061245	3	24,981,224	A	G	0.744	0.998	RARB
rs12629369	3	24,981,362	C	T	0.516	0.999	RARB
rs7614721	3	24,981,533	G	A	0.517	0.999	RARB
rs7648839	3	24,981,622	A	T	0.502	0.999	RARB
rs7637559	3	24,981,651	C	T	0.529	0.999	RARB
rs77349451	3	24,981,782	G	A	0.989	0.998	RARB
3:24981944_AGG_A	3	24,981,944	AGG	A	0.489	0.997	RARB
rs73820331	3	24,982,684	C	G	0.973	0.996	RARB
rs775270092	3	24,982,702	ATAGTGATTAT	A	0.962	0.989	RARB
rs567240324	3	24,982,865	C	CT	0.977	0.848	RARB
rs149918235	3	24,982,908	T	C	0.989	0.997	RARB
rs13068931	3	24,982,947	G	A	0.786	0.997	RARB
rs115416759	3	24,983,150	G	A	0.955	0.991	RARB
rs74690548	3	24,983,156	A	G	0.961	0.992	RARB
rs4858666	3	24,983,994	A	C	0.989	0.998	RARB
rs34744634	3	24,984,103	A	T	0.785	0.997	RARB
rs33953116	3	24,984,390	T	C	0.782	0.996	RARB
rs71311516	3	24,984,609	A	G	0.83	0.999	RARB
rs141484916	3	24,984,918	C	T	0.987	0.914	RARB
rs9833261	3	24,985,557	G	A	0.492	0.998	RARB
rs56369650	3	24,986,337	A	G	0.746	0.997	RARB
rs55964723	3	24,986,415	T	G	0.746	0.997	RARB
rs4858667	3	24,986,891	G	T	0.518	0.998	RARB
rs116835570	3	24,987,294	A	G	0.974	0.999	RARB
rs33999057	3	24,987,597	A	C	0.827	0.997	RARB
rs13098681	3	24,987,736	C	A	0.517	0.996	RARB
3:24987754_CAAA_C	3	24,987,754	CAAA	C	0.516	0.995	RARB
rs11129172	3	24,988,244	C	T	0.746	0.998	RARB
rs4414811	3	24,988,311	T	G	0.972	0.996	RARB
rs71311517	3	24,988,540	A	G	0.828	0.997	RARB
rs116206999	3	24,989,270	A	T	0.974	0.999	RARB
rs9816233	3	24,989,387	A	G	0.745	0.995	RARB
rs145324749	3	24,989,638	A	G	0.987	0.961	RARB
3:24989727_TC_T	3	24,989,727	TC	T	0.747	0.992	RARB
rs115828914	3	24,990,218	C	T	0.974	0.999	RARB
rs13063815	3	24,990,282	T	C	0.828	0.997	RARB
rs6798808	3	24,990,630	G	C	0.487	0.995	RARB
rs78535493	3	24,991,077	G	C	0.974	0.998	RARB
rs17195094	3	24,991,227	C	T	0.829	0.996	RARB
rs115297239	3	24,991,274	T	C	0.974	0.997	RARB
rs9874157	3	24,991,454	A	G	0.746	0.99	RARB
rs9874164	3	24,991,457	A	T	0.746	0.99	RARB
rs11716581	3	24,992,122	T	G	0.742	0.996	RARB
rs369734201	3	24,992,217	A	AAAGT	0.744	0.994	RARB
rs41396146	3	24,992,497	T	C	0.944	0.99	RARB
rs35025157	3	24,993,084	A	C	0.719	0.996	RARB
rs35306981	3	24,993,111	G	C	0.745	0.996	RARB
rs74815672	3	24,993,363	G	T	0.971	0.988	RARB
rs9847199	3	24,993,401	C	T	0.49	0.995	RARB
rs34619201	3	24,993,415	C	T	0.745	0.996	RARB
rs115016339	3	24,993,432	T	C	0.974	0.997	RARB
rs80021103	3	24,993,595	A	G	0.974	0.996	RARB
rs6550927	3	24,993,663	A	G	0.141	0.974	RARB
rs76816986	3	24,993,729	T	C	0.974	0.996	RARB
rs36052996	3	24,993,762	C	G	0.718	0.996	RARB
rs116692682	3	24,993,891	T	C	0.974	0.997	RARB
rs34373965	3	24,993,904	A	G	0.718	0.996	RARB
rs13325698	3	24,994,235	T	C	0.688	0.977	RARB
rs182824892	3	24,994,371	T	G	0.972	0.966	RARB
rs774522404	3	24,994,374	TG	T	0.951	0.973	RARB
rs56397709	3	24,995,765	C	T	0.95	0.985	RARB
rs4858669	3	24,996,208	T	G	0.546	0.997	RARB
rs34101887	3	24,996,978	T	C	0.828	0.996	RARB
rs35180270	3	24,997,106	A	T	0.818	0.997	RARB
rs13083646	3	24,997,330	G	A	0.828	0.997	RARB
rs6780762	3	24,997,512	C	A	0.497	0.995	RARB
rs9873747	3	24,998,357	C	A	0.548	0.998	RARB
rs6799487	3	24,998,500	T	A	0.547	0.999	RARB
rs6797081	3	24,998,723	A	G	0.547	0.999	RARB
rs6802478	3	24,999,165	T	G	0.554	0.982	RARB
rs6762263	3	24,999,166	G	A	0.554	0.982	RARB
rs57183231	3	24,999,356	T	C	0.989	0.992	RARB
rs11708922	3	24,999,739	T	A	0.733	0.992	RARB
rs4564926	3	24,999,801	G	T	0.549	0.997	RARB
rs73034848	3	24,999,806	T	C	0.734	0.995	RARB
rs9879934	3	24,999,912	C	T	0.548	0.999	RARB
rs77322497	3	25,000,433	G	A	0.989	0.994	RARB
rs12635833	3	25,000,664	G	A	0.547	0.999	RARB
rs937269	3	25,001,127	T	C	0.497	0.996	RARB
rs201338499	3	25,001,804	CT	C	0.154	0.969	RARB
rs9866647	3	25,002,065	G	A	0.548	0.997	RARB
rs6792021	3	25,002,078	A	G	0.498	0.995	RARB
3:25002251_AT_A	3	25,002,251	AT	A	0.486	0.968	RARB
rs145111609	3	25,003,089	C	T	0.987	0.848	RARB
rs10510548	3	25,003,280	A	G	0.732	0.994	RARB
rs4299443	3	25,003,444	A	G	0.154	0.967	RARB
rs67338732	3	25,003,451	T	C	0.557	0.987	RARB
rs11718952	3	25,003,606	G	A	0.599	0.984	RARB
rs13068239	3	25,003,683	T	G	0.597	0.984	RARB
rs112182879	3	25,004,005	C	T	0.979	0.982	RARB

SNP	Chromosome	Position	Effect Allele	Other Allele	Effect Allele Frequency	Imputation quality	Gene
rs12490559	3	25,004,351	A	G	0.594	0.985	RARB
rs12487571	3	25,004,369	T	G	0.594	0.985	RARB
rs12490596	3	25,004,487	A	G	0.592	0.985	RARB
rs1319986	3	25,004,596	C	G	0.152	0.975	RARB
rs937267	3	25,004,804	C	A	0.151	0.977	RARB
rs4356783	3	25,004,834	C	G	0.592	0.985	RARB
3:25004908_CTTG_C	3	25,004,908	CTTG	C	0.731	0.988	RARB
rs11386072	3	25,005,380	A	AG	0.682	0.963	RARB
rs28532700	3	25,006,187	C	G	0.581	0.988	RARB
rs4858670	3	25,006,453	C	T	0.317	0.987	RARB
rs4858134	3	25,006,659	T	C	0.581	0.99	RARB
rs62230582	3	25,006,903	G	A	0.586	0.989	RARB
rs6550928	3	25,007,199	G	T	0.144	0.98	RARB
rs9838340	3	25,007,590	T	C	0.966	0.553	RARB
3:25007599_TTTTG_T	3	25,007,599	TTTTG	T	0.441	0.922	RARB
rs750736311	3	25,007,600	TTTG	T	0.404	0.954	RARB
rs2362766	3	25,007,658	C	A	0.139	0.983	RARB
3:25007732_AG_A	3	25,007,732	AG	A	0.975	0.947	RARB
rs35229906	3	25,008,268	T	G	0.738	0.997	RARB
rs4858671	3	25,008,333	G	C	0.573	0.995	RARB
rs77984318	3	25,008,350	T	C	0.975	0.971	RARB
rs4858672	3	25,008,524	T	A	0.574	0.995	RARB
rs4858673	3	25,008,633	C	G	0.138	0.986	RARB
rs4458332	3	25,009,423	G	A	0.74	0.997	RARB
rs141198363	3	25,009,801	G	A	0.988	0.957	RARB
rs9849459	3	25,009,827	T	C	0.573	0.996	RARB
rs9880826	3	25,010,046	C	T	0.574	0.996	RARB
rs9825544	3	25,010,101	T	G	0.573	0.996	RARB
rs4287890	3	25,010,677	A	T	0.138	0.989	RARB
rs4510351	3	25,010,710	A	G	0.573	0.997	RARB
rs4299446	3	25,011,043	T	C	0.119	0.988	RARB
rs6550929	3	25,011,510	G	T	0.138	0.99	RARB
rs11129173	3	25,011,823	A	G	0.574	0.999	RARB
3:25012065_AACAC_A	3	25,012,065	AACAC	A	0.592	0.906	RARB
rs7651592	3	25,012,182	A	G	0.574	0.999	RARB
rs35332564	3	25,012,272	T	C	0.737	1	RARB
rs11707375	3	25,012,357	G	C	0.738	1	RARB
rs11707378	3	25,012,395	G	A	0.738	1	RARB
rs4858135	3	25,012,867	G	A	0.574	0.999	RARB
rs34006813	3	25,012,996	A	T	0.738	1	RARB
rs35145790	3	25,013,199	A	G	0.828	0.992	RARB
rs144943313	3	25,013,483	T	TTACTC	0.315	0.994	RARB
rs6768850	3	25,014,034	T	G	0.574	0.999	RARB
rs35120038	3	25,014,049	C	A	0.738	1	RARB
rs6802439	3	25,014,117	C	G	0.313	0.999	RARB
rs9847103	3	25,014,239	T	C	0.313	0.999	RARB
rs115715352	3	25,015,060	C	G	0.974	0.963	RARB
rs7428127	3	25,015,188	T	A	0.739	0.997	RARB
rs4378933	3	25,015,368	A	G	0.574	1	RARB
rs4498000	3	25,015,406	C	T	0.311	0.997	RARB
rs4502548	3	25,015,541	C	T	0.399	0.994	RARB
rs114731642	3	25,015,651	T	C	0.977	0.947	RARB
rs59515749	3	25,015,708	A	G	0.826	0.99	RARB
rs6779328	3	25,017,218	T	C	0.573	0.999	RARB
rs4318520	3	25,017,344	C	T	0.138	0.989	RARB
rs138518359	3	25,017,506	C	T	0.98	0.963	RARB
rs184187351	3	25,017,625	A	G	0.983	0.924	RARB
rs4524245	3	25,017,764	T	C	0.311	0.996	RARB
rs13099127	3	25,017,947	G	A	0.739	0.996	RARB
rs141508426	3	25,018,344	G	T	0.828	0.99	RARB
rs76172826	3	25,018,628	G	A	0.738	0.996	RARB
rs13062314	3	25,018,886	G	A	0.738	0.996	RARB
rs35910170	3	25,019,178	T	G	0.739	0.996	RARB
rs148173901	3	25,019,293	T	C	0.739	0.996	RARB
rs58645980	3	25,019,539	T	A	0.575	0.999	RARB
rs9682454	3	25,019,631	G	A	0.575	0.999	RARB
rs9682457	3	25,019,748	C	T	0.314	0.996	RARB
rs9681151	3	25,019,817	A	G	0.313	0.996	RARB
rs12635527	3	25,019,935	C	G	0.739	0.996	RARB
rs9683105	3	25,019,980	T	G	0.313	0.996	RARB
rs6787247	3	25,020,386	A	G	0.313	0.996	RARB
rs7632913	3	25,020,919	A	G	0.313	0.996	RARB
rs7618785	3	25,021,117	A	G	0.313	0.996	RARB
rs9713888	3	25,021,178	T	A	0.738	0.996	RARB
rs76122184	3	25,021,422	G	A	0.827	0.989	RARB
rs12638252	3	25,021,635	A	G	0.738	0.996	RARB
rs12632864	3	25,021,636	C	G	0.737	0.994	RARB
rs12635887	3	25,021,998	T	G	0.738	0.996	RARB
rs114029718	3	25,022,048	G	A	0.988	0.925	RARB
3:25022158_AC_A	3	25,022,158	AC	A	0.982	0.981	RARB
rs112455323	3	25,022,283	A	T	0.738	0.996	RARB
rs11928747	3	25,022,387	A	T	0.312	0.996	RARB
rs62230584	3	25,022,439	T	C	0.575	0.998	RARB
rs62230585	3	25,022,465	C	T	0.312	0.996	RARB
3:25022501_TAACA_T	3	25,022,501	TAACA	T	0.827	0.985	RARB
rs77971845	3	25,022,583	C	T	0.738	0.995	RARB
rs111900689	3	25,023,042	A	G	0.977	0.911	RARB
rs4511858	3	25,023,137	T	C	0.138	0.988	RARB
rs4075414	3	25,023,150	G	C	0.911	0.946	RARB
rs4254625	3	25,023,212	T	C	0.139	0.987	RARB
rs34587243	3	25,023,335	C	G	0.738	0.995	RARB
rs4075413	3	25,023,381	C	T	0.576	0.998	RARB
rs73820369	3	25,023,760	A	G	0.826	0.989	RARB
rs75856529	3	25,024,131	C	T	0.979	0.995	RARB
rs4468943	3	25,024,169	T	A	0.737	0.995	RARB
rs35302568	3	25,024,688	C	T	0.737	0.995	RARB
rs9857441	3	25,025,087	G	A	0.139	0.986	RARB
rs9857350	3	25,025,216	C	A	0.311	0.993	RARB
rs12636044	3	25,025,695	G	A	0.738	0.994	RARB
rs12636070	3	25,025,832	G	C	0.736	0.994	RARB
3:25026259_GT_G	3	25,026,259	GT	G	0.138	0.979	RARB
rs112346536	3	25,026,604	G	T	0.978	0.911	RARB
rs7637426	3	25,026,930	A	C	0.738	0.993	RARB
rs7639868	3	25,027,071	T	C	0.737	0.993	RARB
rs62228466	3	25,027,228	G	A	0.981	0.93	RARB
rs4858136	3	25,028,289	A	C	0.312	0.99	RARB
rs60379191	3	25,028,461	G	GGTC	0.574	0.979	RARB
rs778973337	3	25,028,735	TC	T	0.573	0.983	RARB
rs71311518	3	25,028,995	T	C	0.824	0.983	RARB
rs55987296	3	25,029,140	T	C	0.827	0.971	RARB
rs11928741	3	25,029,350	G	A	0.137	0.98	RARB
rs11928745	3	25,029,366	G	A	0.577	0.988	RARB
3:25029457_AT_A	3	25,029,457	AT	A	0.127	0.937	RARB
rs796408480	3	25,029,469	TC	T	0.962	0.544	RARB
rs4858675	3	25,029,904	A	G	0.577	0.985	RARB
rs73036518	3	25,029,960	T	C	0.737	0.984	RARB
rs4858676	3	25,030,060	T	A	0.576	0.984	RARB
rs34079268	3	25,030,075	T	C	0.738	0.983	RARB
rs145557908	3	25,030,613	G	A	0.983	0.911	RARB
rs55835885	3	25,030,730	C	T	0.825	0.978	RARB
rs13070860	3	25,031,001	G	A	0.756	0.844	RARB
3:25031616_CT_C	3	25,031,616	CT	C	0.155	0.946	RARB
rs13099198	3	25,031,899	T	C	0.533	0.799	RARB
rs200410395	3	25,032,247	A	T	0.579	0.965	RARB
rs201667772	3	25,032,248	C	A	0.579	0.965	RARB
rs199819395	3	25,032,249	T	C	0.579	0.965	RARB
rs7639453	3	25,032,614	T	C	0.577	0.976	RARB
rs62228467	3	25,033,007	T	C	0.964	0.908	RARB
rs12496073	3	25,033,188	G	A	0.685	0.956	RARB
rs368514180	3	25,033,252	G	GTT	0.922	0.885	RARB
rs6783221	3	25,033,602	C	T	0.978	0.938	RARB
rs4417830	3	25,034,135	A	G	0.698	0.979	RARB
rs76478639	3	25,034,743	T	G	0.948	0.98	RARB
rs60870237	3	25,035,804	A	ATGTT	0.234	0.978	RARB
3:25035993_AT_A	3	25,035,993	AT	A	0.979	0.924	RARB
rs572051156	3	25,035,995	G	A	0.979	0.925	RARB
rs113456698	3	25,036,132	G	C	0.98	0.975	RARB
rs71311519	3	25,036,480	A	G	0.979	0.978	RARB
rs58230981	3	25,036,722	G	GT	0.287	0.974	RARB
rs62228469	3	25,036,737	T	G	0.769	0.986	RARB
3:25036862_TAG_T	3	25,036,862	TAG	T	0.956	0.94	RARB
rs6550930	3	25,036,930	C	A	0.575	0.989	RARB
rs12629729	3	25,037,015	G	C	0.77	0.987	RARB
rs6771524	3	25,037,250	G	A	0.235	0.986	RARB
rs6771624	3	25,037,358	G	A	0.573	0.989	RARB
rs6771747	3	25,037,457	G	A	0.573	0.989	RARB
rs6797290	3	25,037,798	C	A	0.576	0.99	RARB
rs6550931	3	25,038,152	A	G	0.287	0.987	RARB
rs4410417	3	25,038,558	A	C	0.285	0.988	RARB
rs146495493	3	25,038,641	C	CAT	0.287	0.98	RARB
rs6769630	3	25,039,017	T	A	0.803	0.987	RARB
rs6769712	3	25,039,063	T	A	0.571	0.991	RARB
rs71622792	3	25,039,768	G	GT	0.489	0.959	RARB
rs116055741	3	25,039,772	T	G	0.947	0.974	RARB
rs10631179	3	25,040,312	C	CAAA	0.286	0.983	RARB

SNP	Chromosome	Position	Effect Allele	Other Allele	Effect Allele Frequency	Imputation quality	Gene
rs9826014	3	25,040,502	T	A	0.286	0.99	RARB
rs62228471	3	25,040,595	C	T	0.77	0.99	RARB
rs12497737	3	25,040,601	G	C	0.7	0.987	RARB
rs62228472	3	25,040,670	T	C	0.77	0.99	RARB
rs78346189	3	25,040,983	A	G	0.949	0.99	RARB
rs10630651	3	25,041,494	T	TCA	0.285	0.987	RARB
rs149721118	3	25,041,557	C	T	0.988	0.915	RARB
rs7610231	3	25,042,846	T	C	0.573	0.998	RARB
rs6799310	3	25,043,342	C	G	0.949	0.994	RARB
rs6774166	3	25,043,523	G	A	0.237	0.995	RARB
rs4324440	3	25,043,711	T	C	0.804	0.994	RARB
rs12487626	3	25,043,829	C	G	0.711	0.994	RARB
3:25044239_CTGTG_C	3	25,044,239	CTGTG	C	0.583	0.979	RARB
rs60010645	3	25,044,251	G	A	0.803	0.991	RARB
rs12490228	3	25,046,463	C	T	0.716	0.996	RARB
rs4561795	3	25,046,468	T	G	0.77	0.995	RARB
rs4547662	3	25,046,477	T	G	0.487	0.994	RARB
rs9842801	3	25,047,228	C	G	0.523	0.997	RARB
rs9842936	3	25,047,254	C	A	0.239	0.996	RARB
rs75485375	3	25,047,361	A	C	0.948	0.998	RARB
rs4858678	3	25,047,446	A	G	0.715	0.998	RARB
rs4858679	3	25,047,621	C	G	0.236	0.997	RARB
rs4858137	3	25,047,730	A	C	0.288	0.997	RARB
rs11129175	3	25,048,086	C	G	0.435	0.997	RARB
rs62228474	3	25,048,097	G	A	0.718	0.997	RARB
rs4858138	3	25,048,185	A	T	0.434	0.997	RARB
rs11706159	3	25,048,518	A	C	0.434	0.998	RARB
rs79285081	3	25,048,675	C	T	0.948	0.997	RARB
rs9853245	3	25,049,042	C	T	0.802	0.998	RARB
rs377143477	3	25,049,095	AC	A	0.761	0.988	RARB
rs9853280	3	25,049,096	C	A	0.439	0.929	RARB
rs7629031	3	25,049,196	A	C	0.801	0.999	RARB
rs7617653	3	25,049,205	C	T	0.235	0.998	RARB
rs11129176	3	25,049,310	G	A	0.716	0.998	RARB
rs4574253	3	25,049,351	C	G	0.801	0.999	RARB
rs113919371	3	25,049,378	C	T	0.989	0.967	RARB
rs4325893	3	25,049,583	A	T	0.235	0.998	RARB
rs9868031	3	25,050,175	A	T	0.801	0.999	RARB
rs9830542	3	25,050,218	C	T	0.801	0.999	RARB
rs11711307	3	25,051,076	G	A	0.77	0.996	RARB
rs4302350	3	25,051,117	G	A	0.435	0.996	RARB
rs73036555	3	25,051,204	C	G	0.893	0.976	RARB
rs11129177	3	25,051,352	G	C	0.717	0.995	RARB
rs4564927	3	25,051,556	C	T	0.717	0.995	RARB
rs4566498	3	25,051,630	C	T	0.486	0.994	RARB
rs139141659	3	25,051,636	C	CAGA	0.8	0.989	RARB
rs4566497	3	25,051,674	C	T	0.8	0.993	RARB
rs188449412	3	25,051,707	T	C	0.985	0.949	RARB
rs56399650	3	25,051,770	G	A	0.771	0.994	RARB
rs7650520	3	25,051,793	T	C	0.518	0.994	RARB
rs4858680	3	25,052,001	T	C	0.235	0.995	RARB
rs9874671	3	25,052,005	A	G	0.61	0.991	RARB
rs4858681	3	25,052,109	C	T	0.236	0.996	RARB
rs7639387	3	25,052,274	C	T	0.901	0.986	RARB
rs6550934	3	25,052,333	G	A	0.525	0.991	RARB
rs4241541	3	25,052,548	G	C	0.244	0.996	RARB
rs4241542	3	25,052,754	C	T	0.469	0.994	RARB
rs4482636	3	25,053,043	C	T	0.348	0.996	RARB
rs4280597	3	25,053,482	G	A	0.348	0.996	RARB
rs62228498	3	25,053,617	T	C	0.895	0.995	RARB
rs4298013	3	25,053,643	A	C	0.572	0.995	RARB
rs4241543	3	25,053,752	G	A	0.244	0.997	RARB
rs62228499	3	25,053,824	A	C	0.895	0.995	RARB
rs4241544	3	25,054,011	A	G	0.348	0.997	RARB
rs9872920	3	25,054,852	T	G	0.895	0.996	RARB
rs7649007	3	25,055,566	C	T	0.244	0.997	RARB
rs1875062	3	25,055,926	G	C	0.244	0.997	RARB
rs76723485	3	25,056,383	A	G	0.913	0.988	RARB
rs62228500	3	25,057,579	T	C	0.895	0.998	RARB
rs7611163	3	25,057,622	C	A	0.35	0.998	RARB
rs4447721	3	25,057,881	C	G	0.238	0.998	RARB
rs9310766	3	25,058,174	G	A	0.894	0.998	RARB
rs9310767	3	25,058,176	T	G	0.894	0.998	RARB
rs9310768	3	25,058,407	G	C	0.895	0.999	RARB
rs9818222	3	25,058,436	T	A	0.895	0.999	RARB
rs182609666	3	25,058,706	C	A	0.986	0.934	RARB
rs1021701	3	25,058,757	G	A	0.244	0.999	RARB
rs1021702	3	25,059,227	A	G	0.575	0.997	RARB
rs79145497	3	25,059,325	A	G	0.935	0.97	RARB
rs762606633	3	25,059,742	TAGATC	T	0.576	0.99	RARB
rs7620111	3	25,060,254	C	A	0.245	0.998	RARB
rs62228501	3	25,061,194	C	G	0.895	0.998	RARB
rs1587424	3	25,061,407	C	T	0.918	0.992	RARB
rs1580813	3	25,061,595	C	T	0.252	0.98	RARB
rs772974335	3	25,061,626	TATTATTATTATTATTA	T	0.288	0.943	RARB
rs13087453	3	25,061,808	C	G	0.913	0.975	RARB
rs17015595	3	25,062,752	G	C	0.895	0.995	RARB
rs9821849	3	25,062,783	T	C	0.572	0.995	RARB
rs59538143	3	25,062,956	A	C	0.895	0.995	RARB
rs58108862	3	25,063,190	T	C	0.895	0.995	RARB
rs1909521	3	25,063,595	C	T	0.573	0.994	RARB
rs1909520	3	25,063,701	G	C	0.573	0.994	RARB
rs7637315	3	25,064,021	G	A	0.574	0.994	RARB
rs62228504	3	25,064,579	A	G	0.882	0.991	RARB
rs5847336	3	25,064,995	A	AG	0.066	0.992	RARB
rs873429	3	25,065,129	G	A	0.791	0.997	RARB
rs869857	3	25,065,371	T	C	0.802	0.996	RARB
rs873391	3	25,065,649	T	A	0.593	0.998	RARB
rs873390	3	25,065,662	A	G	0.791	0.997	RARB
rs4858682	3	25,066,225	C	G	0.473	0.997	RARB
rs9842381	3	25,066,274	T	C	0.939	0.97	RARB
rs114152889	3	25,066,330	G	A	0.986	0.987	RARB
rs4858683	3	25,066,439	C	T	0.473	0.994	RARB
3:25066444_CAAAAAA_C	3	25,066,444	CAAAAAA	C	0.322	0.897	RARB
rs4858684	3	25,066,483	A	T	0.479	0.988	RARB
rs17195136	3	25,066,711	A	G	0.79	0.998	RARB
rs62228505	3	25,067,275	T	A	0.923	0.993	RARB
rs17195143	3	25,067,374	T	C	0.923	0.993	RARB
rs62228506	3	25,067,685	A	C	0.79	0.999	RARB
3:25068120_GA_G	3	25,068,120	GA	G	0.53	0.994	RARB
rs6806291	3	25,068,162	G	A	0.622	0.997	RARB
rs1604005	3	25,068,450	A	G	0.332	0.993	RARB
rs1604004	3	25,068,459	A	G	0.332	0.992	RARB
3:25068484_TTAA_T	3	25,068,484	TTAA	T	0.333	0.992	RARB
3:25068546_CAA_C	3	25,068,546	CAA	C	0.803	0.992	RARB
rs1604003	3	25,068,617	A	C	0.332	0.999	RARB
rs779458134	3	25,068,720	TAAGAG	T	0.332	0.999	RARB
rs1604002	3	25,068,871	T	C	0.333	0.999	RARB
rs1604001	3	25,068,929	A	T	0.333	0.999	RARB
rs1604000	3	25,069,001	T	C	0.334	0.999	RARB
3:25069180_CT_C	3	25,069,180	CT	C	0.332	0.997	RARB
3:25069465_ATT_A	3	25,069,465	ATT	A	0.331	0.994	RARB
rs75773180	3	25,069,471	T	C	0.53	0.995	RARB
rs4437100	3	25,069,685	C	T	0.943	0.998	RARB
rs62228507	3	25,070,298	A	G	0.791	0.999	RARB
rs4568084	3	25,070,549	C	T	0.332	0.999	RARB
rs4410416	3	25,070,585	G	A	0.123	1	RARB
rs4458331	3	25,070,608	G	C	0.123	0.999	RARB
rs4345036	3	25,070,715	A	G	0.427	0.998	RARB
rs4482635	3	25,070,898	G	A	0.123	1	RARB
rs7621283	3	25,071,107	C	T	0.123	0.999	RARB
rs1603999	3	25,071,449	G	A	0.332	0.998	RARB
rs13072769	3	25,071,616	C	T	0.49	0.99	RARB
rs6550935	3	25,071,653	C	T	0.125	0.987	RARB
rs6550936	3	25,071,654	C	A	0.332	0.997	RARB
rs1603997	3	25,072,531	C	T	0.275	0.997	RARB
rs74920883	3	25,072,769	C	T	0.985	0.863	RARB
rs9869567	3	25,073,659	A	G	0.275	0.996	RARB
rs6782447	3	25,073,710	C	A	0.868	0.99	RARB
rs138514634	3	25,073,712	G	A	0.981	0.959	RARB
rs1603996	3	25,074,015	C	T	0.275	0.996	RARB
rs4858139	3	25,074,806	G	A	0.429	0.996	RARB
rs4858687	3	25,074,853	C	G	0.371	0.996	RARB
rs4858688	3	25,074,885	T	A	0.371	0.996	RARB
rs4858689	3	25,074,966	T	C	0.371	0.996	RARB
rs4858690	3	25,075,058	G	A	0.373	0.996	RARB
rs4858691	3	25,075,137	C	A	0.371	0.996	RARB
rs4858692	3	25,075,167	C	T	0.371	0.996	RARB
rs74697960	3	25,075,193	T	A	0.982	0.888	RARB
rs11712643	3	25,075,252	G	A	0.372	0.996	RARB
rs12494417	3	25,075,286	T	C	0.371	0.996	RARB
rs13076332	3	25,075,307	T	C	0.371	0.996	RARB
rs1846556	3	25,075,591	T	C	0.375	0.993	RARB
rs1846555	3	25,075,632	C	G	0.375	0.99	RARB
rs1846554	3	25,075,645	A	T	0.375	0.989	RARB

SNP	Chromosome	Position	Effect Allele	Other Allele	Effect Allele Frequency	Imputation quality	Gene
rs116602499	3	25,075,999	A	G	0.986	0.917	RARB
rs79543019	3	25,076,324	T	C	0.888	0.925	RARB
rs13083089	3	25,076,680	T	A	0.915	0.921	RARB
rs149530731	3	25,076,810	G	A	0.986	0.887	RARB
3:25076912_CTTTTTTTTTTTTT_C	3	25,076,912	CTTTTTTTTTTTTT	C	0.038	0.771	RARB
rs4858693	3	25,077,259	A	G	0.572	0.963	RARB
rs7640521	3	25,077,674	C	T	0.821	0.985	RARB
rs1603993	3	25,077,788	T	A	0.573	0.981	RARB
rs1603992	3	25,077,887	T	C	0.721	0.981	RARB
rs13067187	3	25,077,932	G	A	0.857	0.968	RARB
rs773499571	3	25,078,750	C	CT	0.767	0.926	RARB
rs6777624	3	25,079,398	G	C	0.85	0.98	RARB
rs74539999	3	25,079,678	C	T	0.85	0.981	RARB
rs79190378	3	25,079,695	A	G	0.85	0.981	RARB
rs10628337	3	25,079,877	C	CTT	0.571	0.982	RARB
rs9840442	3	25,080,041	G	A	0.821	0.994	RARB
rs71311520	3	25,080,372	A	G	0.922	0.966	RARB
rs62228512	3	25,081,075	A	T	0.901	0.985	RARB
rs9847186	3	25,081,857	G	A	0.572	0.994	RARB
rs9866836	3	25,081,881	T	G	0.85	0.988	RARB
rs9809401	3	25,082,016	A	G	0.85	0.988	RARB
rs13064047	3	25,082,088	G	C	0.85	0.988	RARB
rs17787525	3	25,082,405	A	C	0.901	0.988	RARB
rs949641	3	25,082,904	G	T	0.901	0.988	RARB
rs6550938	3	25,084,111	T	C	0.525	0.983	RARB
rs62228513	3	25,085,098	G	A	0.905	0.99	RARB
rs142461368	3	25,085,306	A	AG	0.905	0.985	RARB
rs145869117	3	25,085,517	A	AT	0.855	0.986	RARB
rs62228514	3	25,086,080	G	A	0.759	0.993	RARB
rs17015670	3	25,086,152	C	T	0.852	0.991	RARB
rs7652292	3	25,086,647	C	T	0.901	0.99	RARB
rs61043032	3	25,086,961	A	G	0.758	0.993	RARB
rs62228516	3	25,087,193	A	T	0.901	0.99	RARB
rs4484159	3	25,087,777	A	G	0.852	0.988	RARB
rs115565242	3	25,088,096	C	A	0.963	0.988	RARB
3:25088877_AG_A	3	25,088,877	AG	A	0.897	0.987	RARB
rs75659483	3	25,089,130	C	G	0.977	0.987	RARB
rs73040992	3	25,089,864	C	A	0.824	0.987	RARB
rs17787531	3	25,089,979	T	C	0.893	0.989	RARB
rs11717464	3	25,090,277	C	T	0.273	0.988	RARB
rs13060459	3	25,090,299	T	A	0.273	0.988	RARB
rs35779991	3	25,090,477	T	C	0.469	0.987	RARB
rs115054009	3	25,090,538	C	G	0.978	0.986	RARB
rs6786779	3	25,090,904	A	G	0.252	0.992	RARB
rs6550939	3	25,091,481	T	C	0.252	0.992	RARB
rs6550940	3	25,091,534	C	T	0.274	0.991	RARB
rs6550941	3	25,091,536	G	A	0.274	0.991	RARB
rs149603370	3	25,091,777	A	AGCT	0.976	0.972	RARB
rs9820531	3	25,092,086	T	C	0.252	0.992	RARB
rs9820537	3	25,092,101	T	C	0.274	0.991	RARB
rs9310769	3	25,092,767	T	A	0.252	0.992	RARB
rs141372907	3	25,093,035	G	GT	0.897	0.987	RARB
rs1604007	3	25,093,056	C	T	0.145	0.994	RARB
rs147383197	3	25,093,086	A	ACATT	0.274	0.989	RARB
rs12639346	3	25,093,530	G	T	0.275	0.992	RARB
rs141225342	3	25,094,122	G	C	0.983	0.902	RARB
rs9811936	3	25,094,294	G	A	0.275	0.992	RARB
3:25094378_GA_G	3	25,094,378	GA	G	0.921	0.729	RARB
rs74690902	3	25,094,471	C	G	0.978	0.986	RARB
rs75561657	3	25,094,828	C	G	0.978	0.988	RARB
rs17515260	3	25,095,090	G	C	0.893	0.991	RARB
rs62228517	3	25,095,380	G	A	0.872	0.991	RARB
rs146890043	3	25,095,487	C	T	0.979	0.967	RARB
3:25095737_GT_G	3	25,095,737	GT	G	0.893	0.988	RARB
rs1609783	3	25,095,911	G	A	0.472	0.987	RARB
3:25096191_ATGGAGCACAAACTG_A	3	25,096,191	ATGGAGCACAAACTG	A	0.894	0.981	RARB
rs9823025	3	25,096,541	G	A	0.278	0.991	RARB
rs74617668	3	25,096,913	C	T	0.978	0.988	RARB
rs62228518	3	25,097,135	G	A	0.893	0.992	RARB
rs144613414	3	25,097,669	T	A	0.979	0.969	RARB
rs4858695	3	25,097,838	T	C	0.275	0.993	RARB
rs141052693	3	25,098,373	T	C	0.988	0.897	RARB
rs76186595	3	25,098,736	A	T	0.894	0.992	RARB
rs11129178	3	25,099,021	T	C	0.275	0.992	RARB
rs10510554	3	25,099,776	T	C	0.431	0.992	RARB
rs10510556	3	25,099,964	C	G	0.894	0.993	RARB
rs1603974	3	25,100,097	T	C	0.893	0.992	RARB
rs62228519	3	25,100,539	A	G	0.893	0.992	RARB
rs67612105	3	25,100,658	G	A	0.844	0.993	RARB
rs4858696	3	25,101,270	C	T	0.437	0.99	RARB
rs988268	3	25,101,448	C	G	0.146	0.996	RARB
rs62228520	3	25,101,875	A	G	0.893	0.992	RARB
rs1122714	3	25,102,269	A	G	0.869	0.988	RARB
rs60628801	3	25,102,766	T	C	0.976	0.97	RARB
rs17515622	3	25,103,029	T	C	0.893	0.992	RARB
rs62228521	3	25,103,108	T	C	0.893	0.992	RARB
rs12632128	3	25,103,258	G	T	0.437	0.99	RARB
rs191398869	3	25,105,092	A	G	0.985	0.901	RARB
rs73821722	3	25,105,215	T	A	0.199	0.929	RARB
rs56207240	3	25,105,216	T	A	0.158	0.975	RARB
rs13327067	3	25,105,266	A	C	0.893	0.993	RARB
rs12715065	3	25,105,288	T	A	0.297	0.996	RARB
rs73034719	3	25,105,548	C	T	0.849	0.995	RARB
rs62228538	3	25,106,119	T	C	0.893	0.991	RARB
rs6804842	3	25,106,437	A	G	0.426	0.993	RARB
3:25106613_AT_A	3	25,106,613	AT	A	0.682	0.966	RARB
rs6767671	3	25,106,760	G	T	0.436	0.989	RARB
3:25107700_TTTCAATTTCTCCAA_T	3	25,107,700	TTTCAATTTCTCCAA	T	0.983	0.967	RARB
rs6808355	3	25,107,714	A	G	0.886	0.984	RARB
rs6808550	3	25,107,866	A	G	0.427	0.993	RARB
rs1603977	3	25,108,179	T	A	0.299	0.994	RARB
rs1603978	3	25,108,236	C	A	0.305	0.993	RARB
rs62228539	3	25,109,123	C	T	0.894	0.994	RARB
rs6550943	3	25,109,249	T	A	0.146	0.997	RARB
3:25109624_CAAAAAAAAAAAAAAAAAAAAAAAAAAAA_C	3	25,109,624	CAAAAAAAAAAAAAAAAAAAAAAAAAAAA	C	0.708	0.907	RARB
rs6777955	3	25,109,802	G	C	0.145	0.997	RARB
rs73149145	3	25,109,872	A	C	0.976	0.971	RARB
rs7619139	3	25,110,415	T	A	0.412	0.993	RARB
rs779898709	3	25,110,504	GA	G	0.915	0.707	RARB
rs150012066	3	25,110,701	A	AT	0.894	0.992	RARB
rs73034730	3	25,110,955	A	G	0.951	0.949	RARB
rs17015753	3	25,111,128	C	A	0.867	0.991	RARB
rs6766372	3	25,111,472	G	T	0.146	0.998	RARB
rs992844	3	25,111,628	C	G	0.279	0.996	RARB
rs6791983	3	25,111,890	C	A	0.251	0.999	RARB
rs201390361	3	25,112,029	AC	A	0.279	0.995	RARB
rs993803	3	25,112,118	C	T	0.28	0.996	RARB
rs993805	3	25,112,399	C	T	0.28	0.995	RARB
rs77023523	3	25,112,427	G	A	0.975	0.969	RARB
rs76614475	3	25,112,434	G	C	0.983	0.912	RARB
rs6772970	3	25,113,247	G	A	0.852	0.983	RARB
rs6798255	3	25,113,358	C	A	0.867	0.991	RARB
rs11715684	3	25,113,555	G	C	0.278	0.994	RARB
rs116825104	3	25,113,706	A	G	0.974	0.895	RARB
rs17015760	3	25,114,032	C	G	0.871	0.991	RARB
rs200445618	3	25,114,207	A	AT	0.976	0.916	RARB
rs186086181	3	25,114,260	C	T	0.987	0.894	RARB
rs112366692	3	25,114,478	C	T	0.874	0.989	RARB
rs17015764	3	25,114,861	C	G	0.867	0.992	RARB
rs6419850	3	25,114,992	G	A	0.149	0.997	RARB
rs994267	3	25,115,194	G	C	0.149	0.997	RARB
rs771794778	3	25,115,322	C	CTT	0.925	0.962	RARB
rs1574901	3	25,115,413	A	G	0.148	0.997	RARB
rs73149174	3	25,115,463	A	C	0.976	0.973	RARB
rs59663978	3	25,115,551	T	G	0.867	0.992	RARB
rs62228543	3	25,115,699	A	C	0.892	0.996	RARB
rs7626290	3	25,115,826	G	A	0.868	0.993	RARB
rs75409596	3	25,115,948	A	T	0.98	0.99	RARB
rs7618700	3	25,116,099	A	G	0.979	0.994	RARB
rs7618802	3	25,116,196	A	G	0.869	0.995	RARB
rs4858697	3	25,116,582	A	G	0.419	0.992	RARB
rs6775433	3	25,116,858	T	G	0.148	0.998	RARB
rs73141440	3	25,117,004	G	A	0.979	0.993	RARB
rs150873790	3	25,117,043	T	A	0.979	0.971	RARB
rs56026803	3	25,117,155	T	C	0.819	0.986	RARB
3:25117165_GA_G	3	25,117,165	GA	G	0.292	0.937	RARB
rs13353418	3	25,117,417	T	A	0.239	0.998	RARB
rs13353419	3	25,117,418	T	C	0.239	0.998	RARB
rs143909588	3	25,117,668	T	A	0.981	0.988	RARB
rs4858698	3	25,118,453	G	A	0.151	0.999	RARB
rs7429279	3	25,118,637	A	C	0.411	0.994	RARB
rs1603988	3	25,118,851	C	A	0.24	1	RARB
rs1603989	3	25,118,874	G	C	0.239	0.999	RARB
rs62228545	3	25,119,231	A	G	0.933	0.997	RARB
rs1587427	3	25,119,467	C	T	0.24	0.999	RARB

SNP	Chromosome	Position	Effect Allele	Other Allele	Effect Allele Frequency	Imputation quality	Gene
rs4858699	3	25,119,759	C	T	0.24	0.999	RARB
rs115311244	3	25,120,040	C	T	0.582	0.777	RARB
rs9809916	3	25,120,103	T	C	0.15	0.999	RARB
rs6797206	3	25,120,269	G	A	0.912	0.997	RARB
rs6773755	3	25,120,571	C	T	0.979	0.994	RARB
rs73034767	3	25,120,674	T	A	0.958	0.888	RARB
rs62228546	3	25,121,397	T	C	0.933	0.997	RARB
rs12629972	3	25,121,444	T	C	0.413	0.993	RARB
rs7628200	3	25,121,493	G	A	0.238	0.999	RARB
rs77805204	3	25,121,510	C	A	0.975	0.886	RARB
rs13325558	3	25,121,752	C	G	0.912	0.997	RARB
rs7630907	3	25,122,044	G	C	0.238	0.998	RARB
rs142365824	3	25,122,083	T	A	0.986	0.94	RARB
rs12638455	3	25,122,201	C	A	0.421	0.989	RARB
rs12630658	3	25,122,238	T	C	0.421	0.99	RARB
rs1587428	3	25,122,428	A	T	0.15	0.999	RARB
rs62228547	3	25,122,524	T	A	0.933	0.996	RARB
rs1587429	3	25,122,586	G	A	0.15	0.999	RARB
rs62228548	3	25,122,917	G	A	0.912	0.997	RARB
rs62228549	3	25,123,390	C	T	0.912	0.996	RARB
3:25123714_TTA_T	3	25,123,714	TTA	T	0.979	0.987	RARB
rs9882518	3	25,123,738	G	T	0.15	0.999	RARB
rs77789283	3	25,124,331	A	G	0.979	0.993	RARB
rs7430038	3	25,124,384	T	G	0.151	1	RARB
rs9831718	3	25,124,508	T	C	0.151	0.999	RARB
rs10446449	3	25,124,621	A	G	0.944	0.888	RARB
rs34416675	3	25,124,630	C	CT	0.148	0.988	RARB
rs1603991	3	25,124,646	G	C	0.239	0.998	RARB
3:25124932_ATT_A	3	25,124,932	ATT	A	0.148	0.986	RARB
rs1587430	3	25,125,365	T	A	0.151	1	RARB
rs2062487	3	25,125,494	C	T	0.151	1	RARB
rs12486699	3	25,125,530	C	A	0.24	0.998	RARB
3:25126035_TC_T	3	25,126,035	TC	T	0.176	0.927	RARB
rs34836731	3	25,126,036	CCCTT	C	0.83	0.983	RARB
rs1609936	3	25,126,174	T	C	0.24	0.998	RARB
rs1609937	3	25,126,175	T	C	0.151	1	RARB
rs1609938	3	25,126,225	G	C	0.239	0.998	RARB
rs62230266	3	25,126,708	A	T	0.944	0.991	RARB
rs4568087	3	25,127,018	C	T	0.239	0.998	RARB
rs9864928	3	25,127,319	A	G	0.238	0.998	RARB
rs62230267	3	25,127,334	A	G	0.933	0.996	RARB
rs9827724	3	25,127,498	C	T	0.238	0.997	RARB
rs75442968	3	25,127,583	T	G	0.944	0.991	RARB
rs4858700	3	25,127,689	T	A	0.239	0.997	RARB
rs4858701	3	25,127,785	G	A	0.239	0.997	RARB
rs4858702	3	25,128,061	A	C	0.15	0.999	RARB
rs73141467	3	25,128,355	C	A	0.968	0.986	RARB
rs79154524	3	25,128,661	C	T	0.968	0.986	RARB
rs148236211	3	25,128,856	T	C	0.989	0.936	RARB
rs62230268	3	25,129,297	A	G	0.934	0.995	RARB
rs115990088	3	25,129,720	C	A	0.981	0.977	RARB
rs9310770	3	25,130,181	A	G	0.15	0.998	RARB
rs62230269	3	25,130,540	T	A	0.912	0.995	RARB
rs201360705	3	25,130,985	AG	A	0.15	0.995	RARB
rs11294076	3	25,130,986	G	A	0.939	0.533	RARB
rs9820612	3	25,131,344	C	G	0.237	0.995	RARB
rs13072262	3	25,131,570	A	G	0.237	0.995	RARB
rs10212193	3	25,131,827	G	A	0.821	0.986	RARB
rs4858703	3	25,133,273	C	A	0.152	0.991	RARB
rs62230270	3	25,133,656	G	A	0.937	0.993	RARB
rs116717000	3	25,133,804	T	C	0.947	0.966	RARB
rs189543805	3	25,133,877	C	T	0.983	0.92	RARB
rs114620699	3	25,134,055	A	G	0.964	0.932	RARB
rs73141468	3	25,134,475	A	T	0.978	0.988	RARB
rs6801041	3	25,134,883	T	C	0.911	0.993	RARB
3:25134987_TC_T	3	25,134,987	TC	T	0.156	0.98	RARB
rs138070837	3	25,135,049	T	C	0.989	0.966	RARB
rs1104724	3	25,135,109	A	G	0.241	0.99	RARB
rs1587431	3	25,135,443	C	T	0.246	0.987	RARB
rs79161528	3	25,136,365	T	C	0.979	0.99	RARB
rs116801142	3	25,136,443	A	G	0.983	0.904	RARB
rs62230271	3	25,137,157	C	G	0.934	0.993	RARB
rs55774515	3	25,137,376	C	A	0.835	0.987	RARB
rs2036270	3	25,137,896	C	T	0.152	0.99	RARB
rs149675360	3	25,138,167	C	CTT	0.937	0.99	RARB
rs4858141	3	25,138,502	C	G	0.239	0.993	RARB
rs972016	3	25,139,652	G	A	0.151	0.995	RARB
rs79752743	3	25,139,846	A	T	0.979	0.988	RARB
rs76163873	3	25,140,065	C	T	0.979	0.987	RARB
rs1603987	3	25,140,536	T	C	0.15	0.994	RARB
rs62230272	3	25,141,032	A	G	0.933	0.992	RARB
rs17516267	3	25,141,279	G	A	0.933	0.991	RARB
rs17575546	3	25,141,612	T	C	0.933	0.992	RARB
rs115878604	3	25,141,627	G	T	0.957	0.905	RARB
rs4444672	3	25,141,695	G	T	0.153	0.989	RARB
rs4353774	3	25,141,824	A	C	0.392	0.984	RARB
rs62230273	3	25,142,296	C	A	0.933	0.992	RARB
rs73044580	3	25,142,430	C	A	0.855	0.985	RARB
rs73044582	3	25,142,502	G	C	0.864	0.985	RARB
rs6807196	3	25,142,571	A	G	0.171	0.992	RARB
rs79652314	3	25,142,879	G	A	0.979	0.987	RARB
rs149612801	3	25,143,026	C	T	0.969	0.9	RARB
rs4858704	3	25,143,390	G	A	0.237	0.991	RARB
rs4858142	3	25,143,427	T	C	0.237	0.991	RARB
rs9861541	3	25,144,041	G	A	0.912	0.989	RARB
rs6550947	3	25,144,071	T	A	0.237	0.991	RARB
rs1609491	3	25,144,210	A	G	0.237	0.991	RARB
rs116025074	3	25,144,269	G	A	0.979	0.986	RARB
rs6550948	3	25,144,409	A	G	0.149	0.992	RARB
rs2012334	3	25,144,472	C	G	0.238	0.991	RARB
rs73044589	3	25,144,549	T	G	0.976	0.976	RARB
rs6776699	3	25,144,648	G	A	0.238	0.99	RARB
rs17516350	3	25,145,143	A	C	0.933	0.99	RARB
rs62230274	3	25,145,595	T	C	0.933	0.99	RARB
rs62230275	3	25,145,747	G	A	0.933	0.989	RARB
rs1580817	3	25,146,601	G	T	0.142	0.988	RARB
rs9877729	3	25,147,386	C	T	0.591	0.996	RARB
rs7622091	3	25,147,497	A	G	0.456	0.995	RARB
rs6778399	3	25,147,501	T	C	0.587	0.993	RARB
rs138362689	3	25,147,752	A	ATGGTAATGTATCACAATTTTAGAAATTTGC	0.455	0.998	RARB
rs766616313	3	25,147,940	TTG	T	0.728	0.779	RARB
rs13326956	3	25,147,942	G	T	0.362	0.875	RARB
rs55870533	3	25,147,945	T	G	0.769	0.989	RARB
rs558896822	3	25,147,949	G	T	0.795	0.916	RARB
rs55972565	3	25,147,953	T	G	0.782	0.97	RARB
rs146335834	3	25,147,957	T	G	0.782	0.97	RARB
3:25147958_TG_T	3	25,147,958	TG	T	0.795	0.916	RARB
rs56166498	3	25,147,959	G	T	0.242	0.967	RARB
3:25147963_TG_T	3	25,147,963	TG	T	0.795	0.916	RARB
rs74956539	3	25,147,964	G	T	0.458	0.996	RARB
rs138719869	3	25,147,980	G	T	0.965	0.79	RARB
rs66831722	3	25,148,054	A	G	0.46	0.999	RARB
rs71622797	3	25,148,144	A	AT	0.464	0.991	RARB
rs35311413	3	25,148,391	A	AT	0.461	0.997	RARB
rs34850898	3	25,148,517	G	A	0.46	0.999	RARB
rs114123975	3	25,148,787	C	T	0.939	0.984	RARB
rs1603986	3	25,148,811	C	T	0.46	1	RARB
rs1603985	3	25,148,868	C	G	0.537	0.997	RARB
rs62230277	3	25,148,989	A	G	0.763	0.997	RARB
rs1603984	3	25,149,092	A	G	0.46	0.999	RARB
rs6550949	3	25,149,419	T	G	0.223	0.997	RARB
rs6550950	3	25,149,436	A	G	0.461	0.999	RARB
rs1603983	3	25,149,547	A	C	0.223	0.997	RARB
rs139606761	3	25,149,927	C	G	0.983	0.964	RARB
rs1587426	3	25,149,971	C	G	0.224	0.997	RARB
3:25150220_GT_G	3	25,150,220	GT	G	0.973	0.859	RARB
rs56098835	3	25,150,654	A	C	0.762	0.995	RARB
rs36023080	3	25,150,712	T	C	0.826	0.998	RARB
rs142270845	3	25,151,728	C	T	0.987	0.949	RARB
rs62230292	3	25,152,222	T	C	0.794	0.994	RARB
rs1580816	3	25,152,496	A	G	0.043	0.994	RARB
rs10510557	3	25,152,944	A	G	0.921	0.987	RARB
rs9826164	3	25,153,406	T	A	0.137	0.99	RARB
rs4858707	3	25,153,554	T	C	0.148	0.989	RARB
rs184527792	3	25,153,746	A	C	0.989	0.962	RARB
rs4858708	3	25,154,112	A	T	0.533	0.994	RARB
rs4858709	3	25,154,268	A	C	0.33	0.995	RARB
rs67987013	3	25,154,327	G	A	0.827	1	RARB
rs62230293	3	25,155,041	G	T	0.792	0.994	RARB
rs1039293	3	25,155,294	G	A	0.531	0.994	RARB
rs1909527	3	25,155,624	G	A	0.531	0.994	RARB
rs1909526	3	25,155,831	C	T	0.535	0.994	RARB
rs41530749	3	25,155,871	A	C	0.792	0.994	RARB
rs55847903	3	25,155,917	G	GT	0.792	0.987	RARB
rs34155045	3	25,155,979	G	A	0.792	0.994	RARB

SNP	Chromosome	Position	Effect Allele	Other Allele	Effect Allele Frequency	Imputation quality	Gene
rs57828525	3	25,156,370	A	G	0.827	0.999	RARB
rs73047766	3	25,156,445	A	G	0.792	0.994	RARB
rs148250501	3	25,156,475	G	A	0.989	0.952	RARB
rs11129180	3	25,156,808	T	G	0.322	0.996	RARB
3:25157922_CA_C	3	25,157,922	CA	C	0.324	0.991	RARB
rs35806066	3	25,158,016	G	A	0.827	0.999	RARB
rs1604008	3	25,158,339	A	G	0.034	0.993	RARB
rs41441147	3	25,158,418	A	G	0.792	0.994	RARB
rs80221584	3	25,158,584	T	C	0.989	0.945	RARB
rs1909525	3	25,158,890	C	A	0.15	0.992	RARB
rs1909524	3	25,159,062	G	A	0.539	0.994	RARB
rs1604012	3	25,159,627	A	T	0.539	0.994	RARB
rs1604011	3	25,159,884	C	T	0.538	0.994	RARB
rs1604010	3	25,160,154	C	T	0.538	0.994	RARB
rs56023574	3	25,160,450	C	G	0.793	0.994	RARB
rs1609782	3	25,160,867	T	G	0.149	0.992	RARB
rs1609781	3	25,160,899	A	T	0.148	0.993	RARB
rs1604009	3	25,161,167	C	A	0.625	0.976	RARB
rs1580819	3	25,161,230	A	G	0.148	0.993	RARB
rs1580818	3	25,161,476	A	G	0.321	0.996	RARB
rs62230297	3	25,161,792	A	G	0.785	0.994	RARB
rs73049908	3	25,163,124	A	G	0.95	0.987	RARB
rs1909523	3	25,163,747	G	A	0.541	0.993	RARB
rs1909522	3	25,164,085	G	A	0.985	0.972	RARB
rs17576064	3	25,164,223	C	G	0.827	0.998	RARB
rs58436684	3	25,164,268	A	G	0.827	0.998	RARB
rs35684523	3	25,164,604	A	G	0.983	0.956	RARB
rs17576085	3	25,164,625	T	C	0.789	0.992	RARB
rs143096912	3	25,164,708	C	T	0.986	0.933	RARB
rs17516853	3	25,164,806	G	A	0.827	0.998	RARB
rs34237251	3	25,164,884	C	G	0.789	0.992	RARB
rs34302149	3	25,165,126	A	G	0.827	0.998	RARB
rs62230298	3	25,165,615	T	C	0.79	0.992	RARB
rs6550951	3	25,165,957	A	G	0.54	0.994	RARB
rs62230299	3	25,166,537	A	G	0.79	0.993	RARB
rs7643079	3	25,166,596	G	A	0.541	0.994	RARB
rs7646062	3	25,167,421	G	A	0.542	0.993	RARB
rs62230300	3	25,168,332	C	G	0.79	0.994	RARB
rs74797183	3	25,168,408	C	G	0.983	0.953	RARB
rs11129181	3	25,168,640	C	T	0.322	0.997	RARB
rs12637631	3	25,168,796	A	C	0.471	0.997	RARB
rs148945977	3	25,168,869	C	T	0.988	0.99	RARB
rs34293324	3	25,168,964	T	C	0.827	0.996	RARB
rs13085249	3	25,169,018	A	G	0.472	0.997	RARB
rs1587422	3	25,169,306	A	G	0.472	0.996	RARB
rs1587423	3	25,169,347	G	A	0.472	0.996	RARB
rs1603972	3	25,170,134	G	A	0.472	0.995	RARB
rs1603973	3	25,170,135	C	G	0.472	0.995	RARB
rs7634050	3	25,170,464	C	G	0.322	0.998	RARB
rs79885851	3	25,170,484	C	T	0.923	0.982	RARB
rs56034169	3	25,170,697	G	T	0.862	0.997	RARB
rs11129182	3	25,171,297	T	C	0.149	0.994	RARB
rs2062483	3	25,171,625	A	C	0.472	0.998	RARB
rs17517019	3	25,171,653	T	C	0.827	0.996	RARB
rs1587425	3	25,171,988	A	G	0.471	0.998	RARB
rs10646076	3	25,172,391	A	ATATT	0.471	0.994	RARB
rs71057699	3	25,172,428	A	ATAT	0.378	0.839	RARB
rs200596555	3	25,172,442	T	C	0.927	0.764	RARB
3:25172450_ATAT_A	3	25,172,450	ATAT	A	0.473	0.972	RARB
3:25172487_TTTATTTA_T	3	25,172,487	TTTATTTA	T	0.471	0.997	RARB
rs11129183	3	25,172,544	A	G	0.471	0.999	RARB
rs11129184	3	25,172,600	A	G	0.471	0.999	RARB
rs11129185	3	25,172,730	A	C	0.472	0.997	RARB
rs11129186	3	25,172,734	C	A	0.472	0.997	RARB
rs11129187	3	25,172,786	G	C	0.472	0.999	RARB
rs13077701	3	25,172,942	C	A	0.471	0.999	RARB
rs6804502	3	25,173,349	G	A	0.472	0.999	RARB
rs200929464	3	25,173,931	G	T	0.971	0.528	RARB
rs80229740	3	25,173,988	T	C	0.98	0.96	RARB
rs7643392	3	25,174,063	T	A	0.472	0.999	RARB
rs7640943	3	25,174,073	A	G	0.462	0.997	RARB
rs7643587	3	25,174,243	T	C	0.527	0.988	RARB
rs7641118	3	25,174,244	A	G	0.472	0.997	RARB
rs10676489	3	25,174,434	C	CTAG	0.475	0.988	RARB
rs35550696	3	25,174,443	C	T	0.475	0.989	RARB
rs73049948	3	25,174,558	T	C	0.827	0.996	RARB
rs4393870	3	25,174,673	A	G	0.471	0.999	RARB
rs4542987	3	25,174,769	T	C	0.471	0.999	RARB
rs4586772	3	25,174,818	G	A	0.471	0.999	RARB
rs4300979	3	25,174,899	T	G	0.471	0.998	RARB
rs4583609	3	25,174,926	T	A	0.471	0.998	RARB
rs9877845	3	25,175,181	A	T	0.471	0.999	RARB
rs11425351	3	25,175,322	G	GA	0.471	0.998	RARB
rs780917749	3	25,175,454	T	TAAA	0.866	0.971	RARB
rs73049956	3	25,175,594	G	A	0.862	0.998	RARB
rs9861111	3	25,175,690	T	C	0.471	0.999	RARB
rs80141288	3	25,175,781	G	C	0.859	1	RARB
rs75245788	3	25,176,065	T	A	0.859	1	RARB
rs4858710	3	25,176,091	T	C	0.149	0.995	RARB
rs146286902	3	25,176,223	A	AT	0.89	0.989	RARB
rs11404120	3	25,176,321	G	GC	0.471	0.996	RARB
rs7636344	3	25,176,449	C	A	0.678	0.997	RARB
3:25176545_C_CT	3	25,176,545	C	CT	0.683	0.98	RARB
rs7636453	3	25,176,609	C	T	0.149	0.994	RARB
rs997309	3	25,176,954	G	A	0.471	0.999	RARB
rs4386457	3	25,177,050	G	A	0.149	0.994	RARB
rs2062486	3	25,177,101	C	A	0.149	0.994	RARB
rs4312625	3	25,177,378	C	T	0.149	0.994	RARB
rs11457527	3	25,177,435	A	AT	0.473	0.993	RARB
rs9871379	3	25,177,454	T	C	0.471	0.998	RARB
rs7653318	3	25,177,516	T	G	0.472	0.997	RARB
rs7653321	3	25,177,523	T	C	0.472	0.997	RARB
rs7616818	3	25,177,713	G	A	0.471	0.998	RARB
rs115812317	3	25,177,902	G	A	0.986	0.954	RARB
rs1580814	3	25,178,568	G	C	0.471	0.998	RARB
rs6764917	3	25,178,609	T	A	0.471	0.998	RARB
rs13076408	3	25,179,376	G	A	0.827	0.994	RARB
rs73048404	3	25,179,533	T	G	0.851	0.996	RARB
rs75332644	3	25,179,608	C	T	0.859	0.997	RARB
rs36109861	3	25,179,902	G	C	0.819	0.993	RARB
rs6804869	3	25,180,288	C	G	0.827	0.993	RARB
rs6771604	3	25,180,487	T	G	0.827	0.993	RARB
rs9840225	3	25,181,030	G	A	0.858	0.996	RARB
rs13098413	3	25,181,113	A	T	0.472	0.995	RARB
rs73048410	3	25,181,446	T	G	0.859	0.995	RARB
rs13099080	3	25,181,453	A	G	0.678	0.992	RARB
rs4426644	3	25,181,563	T	C	0.528	0.985	RARB
rs58452061	3	25,181,575	G	GT	0.69	0.972	RARB
rs58565307	3	25,181,715	T	C	0.984	0.926	RARB
rs73048413	3	25,181,980	T	C	0.862	0.994	RARB
rs9869089	3	25,182,072	T	C	0.677	0.993	RARB
rs76441085	3	25,182,140	T	G	0.945	0.954	RARB
rs55809331	3	25,182,217	T	A	0.864	0.988	RARB
rs56104647	3	25,182,219	T	C	0.687	0.989	RARB
rs56103690	3	25,182,237	A	T	0.679	0.989	RARB
rs9812604	3	25,182,762	A	G	0.827	0.992	RARB
rs12495790	3	25,182,781	T	C	0.471	0.993	RARB
rs71311525	3	25,183,369	T	A	0.827	0.99	RARB
rs7611139	3	25,183,414	T	C	0.691	0.989	RARB
rs56189892	3	25,183,594	G	A	0.864	0.99	RARB
rs56150061	3	25,183,701	T	C	0.862	0.986	RARB
3:25184079_AT_A	3	25,184,079	AT	A	0.499	0.944	RARB
rs57259009	3	25,184,150	T	A	0.883	0.964	RARB
rs6764521	3	25,184,389	A	G	0.342	0.984	RARB
rs115751098	3	25,184,672	A	T	0.988	0.884	RARB
rs6765032	3	25,184,885	A	G	0.166	0.981	RARB
rs1603980	3	25,184,918	C	G	0.487	0.982	RARB
rs1603981	3	25,184,956	A	T	0.173	0.981	RARB
rs73048430	3	25,185,220	A	G	0.865	0.981	RARB
rs1603982	3	25,185,261	C	T	0.487	0.982	RARB
rs75182937	3	25,185,411	C	T	0.865	0.981	RARB
rs9310772	3	25,185,448	T	C	0.167	0.982	RARB
rs35183943	3	25,185,481	A	G	0.821	0.984	RARB
rs73048438	3	25,185,534	T	C	0.974	0.957	RARB
rs12495130	3	25,185,716	G	T	0.488	0.982	RARB
rs7620023	3	25,186,001	T	C	0.169	0.98	RARB
rs73048442	3	25,186,624	T	C	0.864	0.987	RARB
rs73048443	3	25,187,193	A	G	0.821	0.984	RARB
rs113067263	3	25,187,298	C	G	0.864	0.987	RARB
rs201067352	3	25,187,332	CA	C	0.035	0.975	RARB
rs62228592	3	25,187,362	G	A	0.485	0.986	RARB
rs62228593	3	25,187,461	T	C	0.349	0.986	RARB
rs567665268	3	25,187,493	T	TA	0.716	0.948	RARB
rs13093241	3	25,187,602	G	A	0.485	0.986	RARB
rs17576595	3	25,187,702	T	C	0.865	0.987	RARB

SNP	Chromosome	Position	Effect Allele	Other Allele	Effect Allele Frequency	Imputation quality	Gene
rs56092834	3	25,187,994	G	T	0.357	0.968	RARB
rs113504650	3	25,187,998	G	T	0.356	0.969	RARB
rs6789000	3	25,188,002	G	T	0.355	0.969	RARB
rs28534915	3	25,188,006	T	G	0.867	0.961	RARB
rs13077885	3	25,188,037	T	C	0.981	0.969	RARB
rs17576629	3	25,188,433	A	G	0.863	0.985	RARB
rs4858144	3	25,188,640	G	A	0.357	0.988	RARB
rs73048447	3	25,189,287	T	C	0.863	0.988	RARB
rs767823	3	25,189,325	C	T	0.357	0.988	RARB
rs73048450	3	25,189,526	A	T	0.867	0.988	RARB
rs776881289	3	25,189,752	GTTCAA	G	0.493	0.979	RARB
rs17517413	3	25,189,889	A	C	0.867	0.988	RARB
rs17517455	3	25,190,008	A	G	0.863	0.988	RARB
rs4681039	3	25,190,023	A	G	0.494	0.988	RARB
rs4681040	3	25,190,068	G	A	0.494	0.988	RARB
rs66692109	3	25,190,209	C	T	0.865	0.985	RARB
rs10641932	3	25,190,436	C	CTCTG	0.357	0.979	RARB
rs321537	3	25,190,819	T	C	0.823	0.987	RARB
rs321536	3	25,190,954	C	G	0.831	0.987	RARB
rs321535	3	25,191,224	G	C	0.045	0.998	RARB
rs11129188	3	25,191,314	G	A	0.357	0.988	RARB
rs1836840	3	25,191,628	G	T	0.49	0.988	RARB
3:25191691_CA_C	3	25,191,691	CA	C	0.335	0.959	RARB
rs11710620	3	25,192,012	A	G	0.357	0.988	RARB
rs144856404	3	25,192,032	A	ATC	0.866	0.973	RARB
rs73050322	3	25,192,060	A	G	0.864	0.986	RARB
rs13070019	3	25,192,079	T	C	0.358	0.988	RARB
rs80289376	3	25,192,083	G	C	0.864	0.986	RARB
rs17576864	3	25,192,389	A	G	0.863	0.989	RARB
rs904593	3	25,193,047	T	C	0.222	0.989	RARB
rs904592	3	25,193,102	C	T	0.517	0.983	RARB
3:25193408_AT_A	3	25,193,408	AT	A	0.486	0.975	RARB
rs2043620	3	25,194,091	C	T	0.222	0.99	RARB
rs35069077	3	25,194,171	T	A	0.872	0.986	RARB
rs13060347	3	25,194,301	G	A	0.872	0.986	RARB
rs73050329	3	25,194,522	A	G	0.863	0.988	RARB
rs143822311	3	25,194,626	A	AGTGT	0.439	0.958	RARB
rs2043619	3	25,194,739	G	C	0.222	0.989	RARB
rs145779320	3	25,194,959	T	TGG	0.864	0.981	RARB
rs2363595	3	25,194,969	T	C	0.222	0.989	RARB
rs13088163	3	25,195,094	T	G	0.865	0.986	RARB
rs2885836	3	25,195,123	A	G	0.222	0.99	RARB
rs17517778	3	25,195,164	G	C	0.867	0.988	RARB
rs17517792	3	25,195,684	G	A	0.872	0.985	RARB
rs13067015	3	25,195,966	A	C	0.494	0.989	RARB
rs74376340	3	25,196,258	C	T	0.973	0.952	RARB
rs73050336	3	25,196,305	C	T	0.863	0.988	RARB
rs73050338	3	25,196,419	T	C	0.863	0.988	RARB
rs4681089	3	25,196,785	C	T	0.495	0.989	RARB
rs73050339	3	25,197,148	A	G	0.863	0.988	RARB
rs1436256	3	25,197,663	G	A	0.843	0.987	RARB
3:25198007_CAAAAAAAAAAAAAAAAAA_C	3	25,198,007	CAAAAAAAAAAAAAAAAAA	C	0.125	0.897	RARB
rs73050340	3	25,198,421	T	C	0.865	0.986	RARB
rs2649617	3	25,198,473	A	T	0.951	0.979	RARB
rs73050342	3	25,198,604	A	C	0.874	0.986	RARB
rs371828718	3	25,198,869	G	GTA	0.171	0.896	RARB
rs56141112	3	25,198,871	G	A	0.171	0.897	RARB
rs34490775	3	25,199,060	T	TG	0.35	0.983	RARB
rs55642444	3	25,199,097	A	G	0.896	0.974	RARB
rs11276564	3	25,199,403	C	CAAACACACCAG	0.352	0.98	RARB
rs4258924	3	25,199,584	A	G	0.224	0.992	RARB
rs11711519	3	25,199,622	C	T	0.495	0.99	RARB
rs6550953	3	25,199,766	C	T	0.224	0.992	RARB
rs71311526	3	25,200,769	T	C	0.874	0.987	RARB
rs321534	3	25,200,846	G	T	0.836	0.991	RARB
rs564374187	3	25,200,903	A	AC	0.389	0.928	RARB
rs2196466	3	25,201,584	C	A	0.495	0.991	RARB
rs73050349	3	25,201,696	T	C	0.863	0.989	RARB
3:25202669_AATTATTATT_A	3	25,202,669	AATTATTATT	A	0.021	0.907	RARB
rs55750806	3	25,202,802	T	G	0.863	0.989	RARB
rs6793141	3	25,202,977	A	G	0.361	0.99	RARB
rs321533	3	25,203,052	G	A	0.017	0.976	RARB
rs34112261	3	25,203,618	C	T	0.874	0.987	RARB
rs9830836	3	25,203,848	G	A	0.873	0.986	RARB
3:25204995_AATAT_A	3	25,204,995	AATAT	A	0.427	0.926	RARB
rs12638845	3	25,205,922	A	C	0.492	0.989	RARB
rs321517	3	25,206,864	A	T	0.045	0.993	RARB
rs321518	3	25,206,975	T	A	0.047	0.993	RARB
rs321519	3	25,207,173	C	A	0.697	0.991	RARB
rs146088714	3	25,207,218	C	T	0.983	0.962	RARB
rs321520	3	25,207,502	T	C	0.046	0.994	RARB
rs73050358	3	25,207,621	C	T	0.863	0.989	RARB
rs139586873	3	25,207,933	A	AGGGAG	0.863	0.985	RARB
rs321521	3	25,208,164	C	T	0.823	0.994	RARB
rs13092408	3	25,208,246	T	C	0.495	0.992	RARB
rs17517875	3	25,208,270	C	G	0.863	0.99	RARB
rs17577191	3	25,208,341	A	G	0.863	0.99	RARB
rs34370374	3	25,209,224	A	G	0.873	0.988	RARB
rs17517931	3	25,209,362	T	A	0.873	0.988	RARB
rs3111673	3	25,209,556	A	T	0.047	0.992	RARB
rs7645872	3	25,209,801	C	T	0.517	0.99	RARB
rs7623185	3	25,209,889	G	A	0.495	0.993	RARB
rs114241817	3	25,210,198	G	A	0.949	0.961	RARB
rs73050366	3	25,210,326	A	G	0.867	0.989	RARB
rs79104861	3	25,211,520	T	C	0.863	0.989	RARB
rs12491526	3	25,211,834	G	A	0.495	0.993	RARB
rs321546	3	25,211,909	C	T	0.045	0.991	RARB
rs321545	3	25,212,161	C	G	0.045	0.992	RARB
rs321544	3	25,212,352	A	G	0.045	0.992	RARB
rs321543	3	25,212,878	A	C	0.695	0.992	RARB
rs73050373	3	25,213,043	A	T	0.863	0.99	RARB
rs145656351	3	25,213,424	A	ATC	0.863	0.985	RARB
rs13068891	3	25,213,659	G	A	0.874	0.989	RARB
rs321542	3	25,213,963	A	G	0.829	0.994	RARB
rs6550956	3	25,214,024	T	A	0.494	0.993	RARB
rs321541	3	25,214,134	G	C	0.045	0.991	RARB
rs35045659	3	25,214,847	T	C	0.873	0.989	RARB
rs12152294	3	25,215,670	A	G	0.863	0.99	RARB
rs17577540	3	25,216,312	C	G	0.867	0.99	RARB
rs114398546	3	25,217,046	T	A	0.977	0.9	RARB
rs34283888	3	25,217,050	C	T	0.874	0.989	RARB
rs1347559	3	25,217,129	T	C	0.35	0.993	RARB
rs321540	3	25,217,197	A	G	0.819	0.997	RARB
rs11711829	3	25,217,491	A	T	0.494	0.994	RARB
rs71622827	3	25,217,908	G	A	0.873	0.99	RARB
rs321539	3	25,218,165	G	A	0.82	0.998	RARB
rs73052497	3	25,218,298	T	G	0.863	0.991	RARB
rs35383710	3	25,218,803	T	G	0.873	0.99	RARB
rs2649618	3	25,218,842	T	C	0.693	0.994	RARB
rs34051998	3	25,219,107	G	A	0.986	0.949	RARB
rs13061437	3	25,219,196	C	T	0.873	0.99	RARB
rs2574991	3	25,219,269	A	G	0.043	0.993	RARB
rs2649619	3	25,219,310	A	G	0.819	0.998	RARB
rs186190145	3	25,220,038	A	G	0.987	0.947	RARB
rs148475017	3	25,220,444	G	A	0.981	0.952	RARB
rs77277307	3	25,220,482	C	T	0.979	0.964	RARB
rs7640571	3	25,220,520	G	A	0.509	0.988	RARB
rs10865803	3	25,222,051	T	C	0.494	0.995	RARB
rs11129189	3	25,222,184	T	C	0.494	0.995	RARB
rs321525	3	25,222,513	A	G	0.821	0.999	RARB
rs1436238	3	25,222,937	C	T	0.494	0.995	RARB
rs1436237	3	25,223,295	T	C	0.494	0.995	RARB
rs67093028	3	25,223,333	C	T	0.866	0.99	RARB
rs1436236	3	25,223,368	A	G	0.494	0.995	RARB
rs6788715	3	25,223,791	G	A	0.494	0.995	RARB
rs73054309	3	25,224,062	A	G	0.87	0.99	RARB
rs1369874	3	25,224,112	A	G	0.494	0.995	RARB
rs1369873	3	25,224,262	T	G	0.494	0.995	RARB
rs34960742	3	25,224,306	T	A	0.867	0.989	RARB
rs170396	3	25,224,465	A	G	0.051	0.993	RARB
rs321523	3	25,224,718	C	T	0.053	0.992	RARB
rs78945203	3	25,225,021	T	C	0.983	0.964	RARB
rs11711099	3	25,225,163	T	C	0.516	0.992	RARB
rs376382874	3	25,225,627	G	GT	0.729	0.985	RARB
rs17518622	3	25,227,079	A	G	0.863	0.991	RARB
rs34601403	3	25,227,620	A	AT	0.017	0.948	RARB
rs6796800	3	25,228,113	T	C	0.494	0.998	RARB
rs6796899	3	25,228,212	T	C	0.223	0.997	RARB
rs7639228	3	25,228,722	A	T	0.357	0.996	RARB
rs978142	3	25,228,989	C	T	0.23	0.997	RARB
rs17518678	3	25,229,382	C	G	0.867	0.992	RARB
rs75009464	3	25,229,987	G	A	0.979	0.968	RARB
rs321532	3	25,230,017	T	C	0.044	0.999	RARB
3:25230252_TA_T	3	25,230,252	TA	T	0.044	0.998	RARB
rs17578042	3	25,230,419	C	G	0.873	0.992	RARB

SNP	Chromosome	Position	Effect Allele	Other Allele	Effect Allele Frequency	Imputation quality	Gene
rs17518818	3	25,230,869	G	A	0.863	0.993	RARB
rs321530	3	25,231,091	T	C	0.821	0.997	RARB
rs1392117	3	25,231,542	T	G	0.494	0.999	RARB
rs321529	3	25,231,571	T	C	0.689	0.996	RARB
3:25231699_TA_T	3	25,231,699	TA	T	0.053	0.993	RARB
rs931697	3	25,232,253	T	C	0.493	1	RARB
rs924400	3	25,232,267	T	C	0.046	0.998	RARB
3:25232378_AT_A	3	25,232,378	AT	A	0.988	0.815	RARB
rs931696	3	25,232,389	C	T	0.493	1	RARB
rs73054320	3	25,232,588	C	A	0.836	0.995	RARB
rs17578259	3	25,232,823	C	T	0.873	0.993	RARB
rs2649616	3	25,233,025	G	T	0.824	0.996	RARB
rs17578294	3	25,233,081	T	C	0.864	0.993	RARB
rs73054326	3	25,233,578	A	G	0.868	0.993	RARB
rs73054328	3	25,234,172	T	C	0.865	0.992	RARB
rs13087919	3	25,234,228	C	T	0.873	0.991	RARB
rs2649622	3	25,234,533	G	A	0.046	0.994	RARB
rs17578413	3	25,234,967	A	G	0.865	0.991	RARB
rs765552794	3	25,235,401	CAGTT	C	0.182	0.985	RARB
rs1499644	3	25,235,503	T	C	0.182	0.99	RARB
rs1499643	3	25,235,531	T	C	0.182	0.99	RARB
rs79231268	3	25,235,739	G	GGA	0.055	0.955	RARB
rs1436250	3	25,235,862	T	C	0.823	0.992	RARB
rs1499642	3	25,235,947	A	G	0.046	0.991	RARB
3:25236107_CTTAA_C	3	25,236,107	CTTAA	C	0.98	0.913	RARB
rs13100362	3	25,236,154	G	A	0.869	0.986	RARB
rs56039950	3	25,236,195	A	C	0.871	0.99	RARB
rs2068130	3	25,236,833	T	G	0.867	0.985	RARB
rs73054340	3	25,237,049	T	C	0.871	0.99	RARB
rs116758622	3	25,237,355	T	C	0.954	0.989	RARB
rs147461756	3	25,238,137	A	C	0.871	0.99	RARB
rs145667290	3	25,238,283	G	C	0.983	0.974	RARB
rs321528	3	25,238,772	T	C	0.841	0.99	RARB
rs55942234	3	25,239,083	G	C	0.871	0.99	RARB
rs115102647	3	25,239,768	C	G	0.954	0.99	RARB
rs321527	3	25,239,899	C	A	0.829	0.992	RARB
rs73054343	3	25,240,015	G	C	0.871	0.99	RARB
rs62235886	3	25,240,128	T	G	0.95	0.899	RARB
rs321526	3	25,240,188	C	A	0.514	0.991	RARB
rs149399053	3	25,240,855	G	T	0.954	0.989	RARB
rs144793288	3	25,240,892	C	T	0.954	0.988	RARB
rs58509571	3	25,241,447	C	T	0.652	0.985	RARB
rs9868930	3	25,242,188	T	G	0.514	0.987	RARB
rs193157378	3	25,242,409	G	A	0.987	0.817	RARB
rs150775903	3	25,242,828	C	T	0.832	0.986	RARB
rs57970147	3	25,242,896	G	A	0.953	0.987	RARB
rs146212119	3	25,243,195	G	A	0.974	0.884	RARB
rs56299415	3	25,243,250	C	G	0.871	0.989	RARB
rs74672917	3	25,243,334	C	T	0.514	0.987	RARB
3:25243396_GTTTC_G	3	25,243,396	GTTTC	G	0.522	0.972	RARB
rs4533613	3	25,243,669	A	G	0.173	0.988	RARB
rs116456374	3	25,243,844	C	G	0.976	0.951	RARB
rs147690223	3	25,244,048	G	C	0.824	0.984	RARB
rs79247682	3	25,244,357	A	G	0.867	0.985	RARB
rs148179283	3	25,244,653	A	T	0.824	0.984	RARB
rs9755421	3	25,244,654	A	G	0.311	0.989	RARB
rs199689905	3	25,245,035	C	T	0.861	0.976	RARB
rs200554868	3	25,245,038	C	T	0.869	0.979	RARB
rs149679297	3	25,245,319	C	T	0.96	0.961	RARB
rs144662966	3	25,245,549	G	A	0.866	0.973	RARB
rs147620119	3	25,245,640	A	G	0.954	0.989	RARB
rs4302348	3	25,245,684	G	T	0.487	0.99	RARB
rs7625172	3	25,245,752	G	A	0.487	0.99	RARB
rs116218604	3	25,245,916	C	T	0.97	0.965	RARB
rs141143598	3	25,246,180	A	T	0.87	0.989	RARB
3:25246374_TGAG_T	3	25,246,374	TGAG	T	0.954	0.979	RARB
rs147181580	3	25,246,406	A	T	0.87	0.989	RARB
rs191606197	3	25,246,411	C	T	0.985	0.88	RARB
rs140495365	3	25,246,565	C	T	0.988	0.911	RARB
rs148963914	3	25,246,613	T	C	0.871	0.984	RARB
rs112707433	3	25,246,903	G	A	0.851	0.98	RARB
rs67076259	3	25,247,004	T	C	0.824	0.989	RARB
rs112527162	3	25,247,300	T	C	0.824	0.989	RARB
rs7631296	3	25,247,675	G	C	0.171	0.992	RARB
rs6789083	3	25,248,243	G	A	0.485	0.99	RARB
rs140151913	3	25,248,493	A	G	0.988	0.941	RARB
rs143394613	3	25,248,762	T	C	0.984	0.944	RARB
rs67563294	3	25,248,848	A	G	0.824	0.989	RARB
rs143548739	3	25,249,383	G	A	0.981	0.946	RARB
rs73042328	3	25,249,459	A	G	0.87	0.989	RARB
rs114827698	3	25,249,556	C	T	0.954	0.988	RARB
rs7629870	3	25,249,631	T	G	0.487	0.99	RARB
rs67854203	3	25,249,668	C	T	0.869	0.986	RARB
rs12485789	3	25,249,743	C	T	0.487	0.99	RARB
rs75132433	3	25,249,953	G	A	0.953	0.986	RARB
rs920137	3	25,250,196	A	G	0.312	0.988	RARB
rs73042334	3	25,250,355	A	G	0.87	0.989	RARB
rs9826795	3	25,250,866	A	G	0.171	0.992	RARB
rs67128462	3	25,251,162	T	C	0.824	0.989	RARB
rs1436243	3	25,251,946	G	C	0.171	0.991	RARB
rs1436244	3	25,252,641	T	C	0.173	0.992	RARB
rs12108120	3	25,253,387	C	T	0.824	0.988	RARB
rs12497476	3	25,253,485	G	C	0.487	0.989	RARB
rs9880778	3	25,253,999	C	A	0.495	0.972	RARB
rs9881050	3	25,254,002	G	C	0.179	0.972	RARB
3:25254246_CT_C	3	25,254,246	CT	C	0.31	0.981	RARB
rs12107090	3	25,254,288	T	A	0.961	0.983	RARB
rs34445511	3	25,254,452	G	A	0.925	0.959	RARB
rs13088581	3	25,255,519	T	C	0.868	0.985	RARB
rs74877313	3	25,255,669	A	T	0.955	0.982	RARB
rs73042341	3	25,255,711	C	G	0.868	0.984	RARB
rs1561114	3	25,255,958	T	C	0.484	0.989	RARB
rs73042342	3	25,256,506	A	G	0.873	0.989	RARB
rs13072311	3	25,256,769	A	G	0.869	0.984	RARB
rs2164473	3	25,256,862	G	C	0.013	0.938	RARB
rs4681077	3	25,256,919	G	A	0.484	0.989	RARB
rs34549650	3	25,256,951	C	T	0.869	0.984	RARB
rs34296249	3	25,256,996	G	A	0.824	0.986	RARB
rs2196464	3	25,258,158	C	T	0.218	0.989	RARB
rs2196465	3	25,258,280	G	A	0.218	0.989	RARB
rs1595159	3	25,258,505	T	A	0.484	0.99	RARB
rs1595160	3	25,258,602	C	T	0.484	0.99	RARB
rs66483133	3	25,258,731	T	G	0.867	0.99	RARB
rs11927621	3	25,259,265	A	G	0.477	0.991	RARB
rs9856563	3	25,259,472	T	A	0.173	0.99	RARB
rs73042350	3	25,259,653	A	G	0.867	0.992	RARB
rs75474913	3	25,260,140	T	C	0.962	0.987	RARB
rs7618208	3	25,260,281	C	T	0.478	0.992	RARB
rs1561115	3	25,260,453	A	G	0.867	0.987	RARB
rs73042351	3	25,260,568	A	G	0.865	0.993	RARB
rs117777908	3	25,260,646	A	C	0.962	0.986	RARB
rs80106461	3	25,260,709	T	C	0.978	0.935	RARB
rs563840541	3	25,260,756	T	TA	0.386	0.95	RARB
rs73042352	3	25,260,961	T	C	0.866	0.992	RARB
rs55864034	3	25,261,443	A	G	0.866	0.992	RARB
rs9868267	3	25,261,504	G	A	0.176	0.994	RARB
rs9830578	3	25,261,724	A	C	0.172	0.995	RARB
rs558325713	3	25,262,506	T	TAC	0.494	0.977	RARB
rs17015971	3	25,263,036	T	C	0.87	0.992	RARB
rs147652028	3	25,263,120	G	GTATAA	0.86	0.987	RARB
rs6776490	3	25,263,692	A	G	0.048	0.98	RARB
rs149013242	3	25,263,987	T	C	0.989	0.886	RARB
rs13096074	3	25,264,007	A	G	0.867	0.988	RARB
rs35888700	3	25,264,622	C	G	0.869	0.988	RARB
rs73042357	3	25,264,821	A	G	0.873	0.993	RARB
rs17015978	3	25,264,841	T	G	0.869	0.989	RARB
rs958737	3	25,265,533	C	T	0.484	0.994	RARB
rs73042364	3	25,265,790	A	G	0.873	0.993	RARB
rs73042365	3	25,266,238	A	G	0.873	0.993	RARB
rs112422509	3	25,266,324	A	T	0.968	0.895	RARB
rs137967058	3	25,266,566	T	C	0.955	0.984	RARB
rs1436246	3	25,266,621	C	T	0.87	0.993	RARB
rs1369875	3	25,266,622	C	G	0.172	0.997	RARB
rs4521188	3	25,266,659	A	G	0.873	0.993	RARB
rs1369876	3	25,266,704	C	T	0.87	0.993	RARB
rs77761860	3	25,266,797	T	TGA	0.482	0.992	RARB
rs4681038	3	25,267,016	T	C	0.482	0.996	RARB
rs77243731	3	25,267,167	C	T	0.962	0.986	RARB
rs9310773	3	25,267,578	G	A	0.172	0.997	RARB
rs34709547	3	25,267,897	A	G	0.87	0.993	RARB
rs9825855	3	25,268,008	G	C	0.173	0.997	RARB
rs73042374	3	25,268,234	C	T	0.87	0.989	RARB
rs73042375	3	25,268,235	T	C	0.87	0.99	RARB
rs6781087	3	25,268,550	C	G	0.175	0.996	RARB
rs35336027	3	25,268,797	G	A	0.87	0.993	RARB

SNP	Chromosome	Position	Effect Allele	Other Allele	Effect Allele Frequency	Imputation quality	Gene
rs7432016	3	25,268,910	T	G	0.87	0.993	RARB
3:25269708_GTT_G	3	25,269,708	GTT	G	0.336	0.964	RARB
rs10510558	3	25,269,758	A	G	0.869	0.989	RARB
rs10510559	3	25,269,928	T	C	0.869	0.989	RARB
rs2363594	3	25,270,064	C	T	0.173	0.998	RARB
rs73042380	3	25,270,350	T	C	0.869	0.99	RARB
rs10510560	3	25,270,543	G	A	0.869	0.99	RARB
rs67880640	3	25,270,670	T	G	0.867	0.988	RARB
rs66807073	3	25,270,961	A	G	0.869	0.988	RARB
rs67150539	3	25,270,970	T	A	0.869	0.99	RARB
rs376375418	3	25,271,147	C	CT	0.869	0.983	RARB
rs2060565	3	25,271,265	T	C	0.173	0.999	RARB
rs184107201	3	25,271,923	A	T	0.986	0.926	RARB
rs11924843	3	25,272,108	A	G	0.483	0.995	RARB
rs34620038	3	25,272,595	T	G	0.482	0.997	RARB
rs13097976	3	25,272,668	T	C	0.483	0.995	RARB
rs6779499	3	25,273,007	C	T	0.87	0.989	RARB
rs6792184	3	25,273,014	A	G	0.87	0.989	RARB
3:25273501_GT_G	3	25,273,501	GT	G	0.955	0.981	RARB
rs79920912	3	25,273,820	G	A	0.955	0.989	RARB
rs56127830	3	25,273,994	A	G	0.867	0.995	RARB
rs6798155	3	25,274,007	T	A	0.173	0.999	RARB
rs74684476	3	25,274,151	G	C	0.967	0.933	RARB
rs2885835	3	25,274,250	G	A	0.173	0.999	RARB
rs535260294	3	25,274,464	C	T	0.688	0.751	RARB
rs2363593	3	25,274,665	T	G	0.484	0.995	RARB
rs114973791	3	25,275,001	C	T	0.987	0.941	RARB
rs78134079	3	25,275,035	T	C	0.961	0.987	RARB
rs34708987	3	25,275,106	C	A	0.925	0.974	RARB
rs13092896	3	25,275,474	A	C	0.869	0.99	RARB
rs9876917	3	25,275,670	A	C	0.173	0.999	RARB
rs138891905	3	25,276,137	C	CT	0.828	0.988	RARB
rs73044253	3	25,276,397	G	C	0.867	0.996	RARB
rs6765179	3	25,276,416	G	A	0.689	0.994	RARB
rs73044255	3	25,276,554	A	G	0.867	0.996	RARB
rs73044259	3	25,276,720	T	G	0.867	0.996	RARB
rs73044260	3	25,277,232	C	T	0.975	0.963	RARB
rs73044261	3	25,277,494	A	G	0.867	0.995	RARB
rs35097065	3	25,277,514	C	T	0.925	0.974	RARB
rs61691090	3	25,279,173	A	G	0.981	0.997	RARB
rs322720	3	25,279,201	A	G	0.483	0.997	RARB
rs147337377	3	25,279,711	T	A	0.987	0.951	RARB
rs118014198	3	25,280,277	C	T	0.962	0.991	RARB
rs322717	3	25,280,339	G	A	0.483	0.997	RARB
rs60540126	3	25,280,455	G	A	0.981	0.997	RARB
3:25280461_CTG_C	3	25,280,461	CTG	C	0.981	0.993	RARB
rs79821792	3	25,281,216	C	A	0.981	0.996	RARB
rs35984506	3	25,281,553	C	T	0.71	0.996	RARB
rs66939282	3	25,281,793	A	G	0.87	0.995	RARB
rs2164475	3	25,282,004	C	T	0.73	0.994	RARB
rs2164474	3	25,282,013	T	G	0.73	0.994	RARB
rs55793955	3	25,282,598	A	G	0.981	0.994	RARB
rs78760206	3	25,282,808	C	G	0.968	0.873	RARB
rs2117969	3	25,282,868	G	A	0.672	0.997	RARB
rs9872973	3	25,283,171	T	A	0.672	0.998	RARB
rs148121266	3	25,283,233	T	C	0.867	0.996	RARB
rs150915364	3	25,283,771	A	C	0.867	0.996	RARB
rs116757684	3	25,284,012	T	C	0.962	0.989	RARB
rs1656859	3	25,284,225	A	G	0.518	0.998	RARB
rs143805943	3	25,285,416	G	A	0.93	0.984	RARB
rs111652469	3	25,285,442	T	G	0.868	0.996	RARB
rs200215353	3	25,285,763	AT	A	0.71	0.861	RARB
rs115408246	3	25,286,074	C	T	0.962	0.993	RARB
rs116094662	3	25,286,318	T	C	0.978	0.965	RARB
rs139813101	3	25,286,479	C	T	0.959	0.937	RARB
rs200254347	3	25,286,623	G	GT	0.961	0.979	RARB
rs73044279	3	25,286,686	T	C	0.867	0.996	RARB
rs13085280	3	25,286,731	G	A	0.87	0.996	RARB
rs142207974	3	25,286,972	T	A	0.93	0.984	RARB
rs322716	3	25,287,156	G	C	0.846	0.998	RARB
rs13090740	3	25,287,466	C	G	0.87	0.996	RARB
rs116367284	3	25,287,611	T	G	0.874	0.997	RARB
rs144653702	3	25,287,640	G	T	0.874	0.996	RARB
rs167734	3	25,288,486	G	A	0.828	0.998	RARB
rs34985047	3	25,288,934	A	T	0.87	0.996	RARB
rs322710	3	25,288,951	A	G	0.518	0.998	RARB
rs7427426	3	25,289,516	C	G	0.87	0.996	RARB
rs322709	3	25,289,909	C	T	0.519	0.999	RARB
rs1601161	3	25,290,005	A	G	0.87	0.996	RARB
rs1601162	3	25,290,069	C	G	0.87	0.996	RARB
rs1595161	3	25,290,252	G	A	0.672	0.999	RARB
rs34197182	3	25,290,905	G	A	0.851	0.996	RARB
rs35769460	3	25,290,986	T	G	0.68	0.998	RARB
rs73044294	3	25,291,735	C	T	0.87	0.996	RARB
rs73044296	3	25,291,916	A	G	0.868	0.997	RARB
rs1601163	3	25,292,241	C	T	0.672	0.999	RARB
rs1601164	3	25,292,248	A	G	0.672	0.999	RARB
rs2117968	3	25,292,487	T	C	0.672	0.999	RARB
rs141068580	3	25,292,837	T	C	0.955	0.992	RARB
rs6778300	3	25,292,966	T	C	0.981	0.995	RARB
rs34403304	3	25,293,140	T	G	0.869	0.997	RARB
3:25293282_CTTCTT_C	3	25,293,282	CTTCTT	C	0.87	0.993	RARB
rs322715	3	25,293,713	A	G	0.827	0.998	RARB
rs1992060	3	25,294,517	A	G	0.87	0.996	RARB
rs77824431	3	25,294,564	A	T	0.981	0.964	RARB
rs322714	3	25,294,588	T	C	0.827	0.998	RARB
rs73045965	3	25,294,732	A	G	0.87	0.996	RARB
rs322713	3	25,295,241	G	T	0.827	0.998	RARB
rs145722390	3	25,295,271	T	C	0.962	0.993	RARB
rs322712	3	25,295,588	T	A	0.517	0.997	RARB
rs322711	3	25,295,765	T	G	0.827	0.997	RARB
rs35646412	3	25,295,869	C	G	0.87	0.995	RARB
rs148312892	3	25,296,278	T	C	0.987	0.944	RARB
rs73045970	3	25,296,328	T	G	0.866	0.997	RARB
rs188923	3	25,296,960	A	G	0.025	0.994	RARB
3:25297129_GA_G	3	25,297,129	GA	G	0.963	0.989	RARB
rs114266842	3	25,297,458	C	T	0.976	0.953	RARB
rs1992059	3	25,298,087	A	G	0.869	0.995	RARB
rs79680798	3	25,298,213	A	G	0.956	0.992	RARB
3:25298296_GA_G	3	25,298,296	GA	G	0.966	0.715	RARB
rs9829952	3	25,298,395	G	C	0.197	0.995	RARB
rs77368242	3	25,298,404	T	C	0.982	0.898	RARB
rs13082318	3	25,298,421	C	T	0.87	0.995	RARB
rs13082854	3	25,298,725	C	G	0.977	0.951	RARB
rs111255065	3	25,299,011	G	T	0.99	0.941	RARB
rs13087573	3	25,299,079	A	C	0.869	0.995	RARB
rs322722	3	25,299,134	C	T	0.329	0.996	RARB
rs73045975	3	25,299,989	C	G	0.843	0.997	RARB
rs17016060	3	25,300,048	G	C	0.869	0.995	RARB
rs322721	3	25,300,486	A	T	0.37	0.993	RARB
rs77754376	3	25,300,498	T	A	0.982	0.966	RARB
rs56825831	3	25,300,550	A	G	0.954	0.989	RARB
rs59205781	3	25,300,860	T	C	0.954	0.988	RARB
rs75112588	3	25,301,185	A	G	0.989	0.923	RARB
rs13074533	3	25,302,484	A	G	0.869	0.995	RARB
rs1483855	3	25,302,539	C	A	0.196	0.995	RARB
rs145474042	3	25,302,634	G	A	0.987	0.934	RARB
rs6780105	3	25,303,705	C	G	0.83	0.998	RARB
rs35508620	3	25,303,715	T	G	0.87	0.995	RARB
rs77580283	3	25,304,060	T	G	0.837	0.997	RARB
rs67681308	3	25,304,167	C	T	0.867	0.995	RARB
rs12632391	3	25,304,288	G	A	0.537	0.995	RARB
rs10510561	3	25,304,382	A	G	0.87	0.995	RARB
rs17016078	3	25,305,008	T	C	0.869	0.995	RARB
rs73151295	3	25,305,272	G	A	0.963	0.992	RARB
rs5005738	3	25,305,522	C	T	0.547	0.994	RARB
rs13093059	3	25,305,567	C	T	0.869	0.995	RARB
rs760235940	3	25,305,601	GA	G	0.213	0.971	RARB
rs76620846	3	25,305,602	A	T	0.561	0.884	RARB
rs73151296	3	25,305,813	T	C	0.962	0.991	RARB
rs574664942	3	25,305,855	A	ATT	0.87	0.991	RARB
rs61228958	3	25,306,004	A	T	0.869	0.995	RARB
rs57402663	3	25,306,222	C	T	0.869	0.995	RARB
rs57967352	3	25,306,533	C	A	0.963	0.992	RARB
rs117878134	3	25,306,647	C	T	0.963	0.993	RARB
rs73045997	3	25,306,684	G	C	0.831	0.998	RARB
rs73045999	3	25,306,770	A	G	0.831	0.998	RARB
rs7635517	3	25,306,801	C	A	0.198	0.995	RARB
rs35974053	3	25,306,974	A	G	0.537	0.995	RARB
rs138512407	3	25,306,998	C	T	0.987	0.943	RARB
rs2082932	3	25,307,260	T	G	0.198	0.995	RARB
rs34537630	3	25,307,331	G	C	0.869	0.995	RARB
rs67912541	3	25,307,571	A	C	0.869	0.995	RARB
rs68073275	3	25,307,941	G	C	0.869	0.995	RARB
rs144147893	3	25,307,983	A	G	0.989	0.945	RARB

SNP	Chromosome	Position	Effect Allele	Other Allele	Effect Allele Frequency	Imputation quality	Gene
rs13068143	3	25,308,482	C	T	0.869	0.995	RARB
rs56399652	3	25,308,676	A	T	0.831	0.998	RARB
rs56116603	3	25,308,880	G	T	0.794	0.997	RARB
rs4681043	3	25,308,895	C	T	0.198	0.995	RARB
rs13091754	3	25,308,961	T	C	0.869	0.995	RARB
rs1483851	3	25,309,054	A	G	0.538	0.995	RARB
rs1483850	3	25,309,159	G	A	0.198	0.995	RARB
rs4234353	3	25,309,706	C	T	0.198	0.995	RARB
rs322704	3	25,309,745	C	T	0.955	0.974	RARB
rs73047810	3	25,309,748	T	C	0.831	0.999	RARB
rs17016117	3	25,309,808	G	A	0.869	0.995	RARB
3:25309881_GA_G	3	25,309,881	GA	G	0.242	0.936	RARB
rs4681014	3	25,309,997	G	C	0.538	0.996	RARB
rs17016120	3	25,310,063	G	T	0.869	0.995	RARB
rs35860270	3	25,310,114	A	G	0.869	0.995	RARB
rs17579478	3	25,310,339	T	C	0.538	0.996	RARB
rs9875315	3	25,310,763	A	G	0.659	0.996	RARB
rs79002254	3	25,310,840	A	G	0.831	0.999	RARB
rs1865750	3	25,311,020	G	A	0.199	0.994	RARB
rs73047816	3	25,311,552	A	G	0.842	0.998	RARB
rs79807077	3	25,311,647	G	A	0.961	0.992	RARB
rs13077354	3	25,311,685	T	C	0.846	0.944	RARB
rs73047879	3	25,311,767	T	C	0.869	0.994	RARB
rs13059799	3	25,312,094	C	G	0.869	0.994	RARB
rs322705	3	25,312,108	A	G	0.357	0.995	RARB
rs13082440	3	25,312,157	T	C	0.869	0.994	RARB
rs79376772	3	25,312,381	G	A	0.955	0.99	RARB
rs762238727	3	25,312,538	AC	A	0.552	0.986	RARB
rs34820979	3	25,313,017	T	C	0.869	0.995	RARB
rs17016133	3	25,313,167	T	C	0.869	0.996	RARB
rs322706	3	25,313,398	T	G	0.321	0.995	RARB
rs35180299	3	25,313,554	A	T	0.869	0.996	RARB
rs9853652	3	25,313,709	C	T	0.958	0.992	RARB
rs9853990	3	25,313,888	C	T	0.705	0.995	RARB
rs6777760	3	25,315,376	G	A	0.534	0.999	RARB
rs1872144	3	25,315,828	A	G	0.208	0.996	RARB
rs75260183	3	25,315,902	G	A	0.961	0.972	RARB
rs1872143	3	25,315,977	C	T	0.535	1	RARB
rs13084418	3	25,316,314	G	A	0.869	0.997	RARB
rs13084608	3	25,316,406	G	A	0.87	0.996	RARB
rs13090773	3	25,317,646	A	G	0.536	0.997	RARB
rs73047894	3	25,317,863	T	C	0.847	0.995	RARB
rs72619933	3	25,317,904	T	C	0.96	0.996	RARB
rs4681015	3	25,318,014	G	A	0.535	0.997	RARB
rs17016135	3	25,318,290	C	T	0.96	0.997	RARB
rs322707	3	25,319,260	T	C	0.319	0.994	RARB
rs1483847	3	25,319,676	T	G	0.208	0.992	RARB
rs35398947	3	25,319,681	G	A	0.872	0.997	RARB
rs1483846	3	25,319,827	G	C	0.532	0.994	RARB
rs4681044	3	25,320,221	G	A	0.361	0.994	RARB
rs1483845	3	25,320,836	T	A	0.208	0.99	RARB
rs17016141	3	25,320,960	T	C	0.872	0.997	RARB
rs167731	3	25,321,489	C	T	0.011	0.979	RARB
rs200478627	3	25,322,077	G	GA	0.988	0.962	RARB
rs12497184	3	25,323,371	G	A	0.248	0.987	RARB
rs35264689	3	25,323,387	A	G	0.872	0.998	RARB
rs7632587	3	25,323,662	G	C	0.72	0.994	RARB
rs6550960	3	25,323,954	T	C	0.697	0.992	RARB
rs10627438	3	25,323,971	C	CTTAA	0.72	0.99	RARB
rs6766784	3	25,324,132	C	G	0.248	0.986	RARB
rs322660	3	25,324,577	A	G	0.955	0.963	RARB
rs77185119	3	25,324,762	A	T	0.982	0.963	RARB
rs150387247	3	25,324,998	G	A	0.988	0.91	RARB
rs7430212	3	25,325,289	T	C	0.721	0.995	RARB
rs322659	3	25,325,452	A	T	0.026	0.985	RARB
rs1436240	3	25,325,453	A	T	0.722	0.995	RARB
3:25325954_TTTTA_T	3	25,325,954	TTTTA	T	0.872	0.962	RARB
rs13069258	3	25,326,083	A	G	0.208	0.961	RARB
rs56062751	3	25,326,535	C	T	0.872	0.968	RARB
rs78923339	3	25,326,609	C	T	0.982	0.966	RARB
rs67889340	3	25,326,927	A	G	0.724	0.976	RARB
rs73049706	3	25,327,020	T	A	0.838	0.998	RARB
rs13075476	3	25,327,112	C	G	0.728	0.972	RARB
rs148836838	3	25,327,407	A	C	0.984	0.853	RARB
rs7635116	3	25,327,649	A	G	0.949	0.955	RARB
rs150489340	3	25,327,682	C	CCAT	0.736	0.9	RARB
rs7623731	3	25,327,699	C	T	0.949	0.956	RARB
rs6790387	3	25,327,793	A	C	0.794	0.916	RARB
rs78789840	3	25,328,056	T	C	0.975	0.947	RARB
rs79507751	3	25,328,394	T	C	0.944	0.966	RARB
rs72619934	3	25,328,458	C	T	0.944	0.955	RARB
rs322683	3	25,328,838	C	T	0.658	0.976	RARB
rs2885837	3	25,329,131	A	C	0.943	0.971	RARB
rs322682	3	25,329,478	G	A	0.657	0.98	RARB
rs322681	3	25,329,792	C	G	0.647	0.984	RARB
rs322680	3	25,329,833	G	A	0.654	0.984	RARB
3:25329853_CTCTCCCTAG_C	3	25,329,853	CTCTCCCTAG	C	0.948	0.981	RARB
rs322679	3	25,330,593	T	G	0.76	0.982	RARB
rs322678	3	25,330,898	T	C	0.639	0.995	RARB
rs322677	3	25,331,181	G	C	0.82	0.989	RARB
rs322676	3	25,331,204	A	G	0.82	0.989	RARB
rs35019857	3	25,331,231	A	AT	0.82	0.981	RARB
rs322675	3	25,331,285	A	G	0.82	0.99	RARB
rs11916207	3	25,331,908	C	T	0.943	0.977	RARB
rs188922	3	25,332,028	G	A	0.866	0.972	RARB
rs74640398	3	25,332,073	C	T	0.945	0.992	RARB
rs76800056	3	25,332,173	A	G	0.944	0.992	RARB
rs78505400	3	25,332,201	A	G	0.944	0.992	RARB
rs77473806	3	25,332,387	T	G	0.944	0.991	RARB
rs322673	3	25,332,423	G	A	0.764	0.993	RARB
rs75262580	3	25,332,663	T	C	0.944	0.992	RARB
rs322672	3	25,332,732	T	C	0.649	0.996	RARB
rs778400870	3	25,332,967	C	CTTTTT	0.868	0.814	RARB
rs322671	3	25,332,992	A	G	0.821	0.989	RARB
rs80344539	3	25,333,204	A	G	0.944	0.993	RARB
rs79389677	3	25,333,239	C	T	0.944	0.993	RARB
rs541586937	3	25,333,538	C	CTT	0.77	0.97	RARB
rs748951882	3	25,333,550	G	GTG	0.87	0.761	RARB
rs322670	3	25,333,575	T	A	0.821	0.99	RARB
rs75036686	3	25,333,964	G	A	0.944	0.994	RARB
rs78337440	3	25,334,456	C	T	0.946	0.996	RARB
rs9833984	3	25,334,640	G	A	0.83	0.988	RARB
rs184429	3	25,335,068	G	A	0.682	0.983	RARB
rs557784646	3	25,335,088	T	TAAAAAAAAAAAAAAAAA	0.333	0.943	RARB
rs74783597	3	25,335,114	A	G	0.948	0.993	RARB
rs6801559	3	25,335,157	T	G	0.828	0.988	RARB
rs80049233	3	25,335,207	A	C	0.942	0.977	RARB
rs1436252	3	25,335,435	A	G	0.655	0.994	RARB
rs6810082	3	25,335,519	G	A	0.83	0.988	RARB
rs77153830	3	25,335,711	G	A	0.942	0.977	RARB
rs11928224	3	25,335,804	A	C	0.827	0.987	RARB
rs1681551	3	25,335,854	A	C	0.656	0.994	RARB
3:25336183_TAGCTTCTCTCCTTCCAACGGCCCGTTGGCGGA_T	3	25,336,183	TAGCTTCTCTCCTTCCAACGGCCCGTTGGCGGA	T	0.826	0.974	RARB
rs111445894	3	25,336,232	T	G	0.827	0.979	RARB
rs1656878	3	25,336,240	T	C	0.648	0.989	RARB
rs78410335	3	25,336,241	G	A	0.835	0.965	RARB
rs76157493	3	25,336,300	A	G	0.945	0.998	RARB
rs73154970	3	25,336,323	A	G	0.827	0.988	RARB
rs67227419	3	25,336,388	G	C	0.753	0.978	RARB
rs73154972	3	25,336,445	T	G	0.827	0.988	RARB
rs73154973	3	25,336,466	T	C	0.935	0.976	RARB
rs9844329	3	25,336,575	C	A	0.827	0.988	RARB
rs77558726	3	25,336,973	A	G	0.946	0.997	RARB
rs1681550	3	25,337,264	C	T	0.829	0.992	RARB
rs1681549	3	25,337,402	A	G	0.276	0.961	RARB
rs76745977	3	25,337,472	T	C	0.941	0.976	RARB
rs139593804	3	25,337,473	C	G	0.98	0.906	RARB
rs977226	3	25,337,483	C	T	0.217	0.959	RARB
rs77949784	3	25,337,678	A	G	0.945	0.995	RARB
rs977225	3	25,337,783	T	C	0.829	0.992	RARB
rs977224	3	25,337,874	G	A	0.276	0.961	RARB
rs111815133	3	25,338,297	C	A	0.947	0.99	RARB
rs75751556	3	25,338,549	C	T	0.941	0.976	RARB
rs370805112	3	25,338,893	CTTTTTT	C	0.413	0.906	RARB
rs17016150	3	25,338,961	T	C	0.945	0.994	RARB
rs322669	3	25,338,994	T	G	0.763	0.991	RARB
rs78040398	3	25,339,137	T	G	0.946	0.994	RARB
rs80074360	3	25,339,211	C	T	0.946	0.994	RARB
rs2164478	3	25,339,647	G	A	0.452	0.97	RARB
3:25339675_CT_C	3	25,339,675	CT	C	0.544	0.941	RARB
rs78525119	3	25,340,298	A	G	0.941	0.976	RARB
rs116557838	3	25,340,371	C	T	0.947	0.989	RARB
rs2363596	3	25,340,452	C	T	0.445	0.97	RARB
rs113014757	3	25,340,618	G	A	0.947	0.989	RARB
rs185990320	3	25,341,255	T	A	0.988	0.771	RARB

SNP	Chromosome	Position	Effect Allele	Other Allele	Effect Allele Frequency	Imputation quality	Gene
rs167732	3	25,341,796	C	A	0.658	0.991	RARB
rs76242587	3	25,342,586	G	A	0.945	0.99	RARB
rs775824682	3	25,342,927	GCTGGTGATATAAC	G	0.763	0.986	RARB
rs78672041	3	25,343,317	T	C	0.945	0.991	RARB
rs322668	3	25,343,615	G	A	0.328	0.966	RARB
rs75107883	3	25,343,861	A	G	0.945	0.989	RARB
rs75808553	3	25,344,357	T	C	0.945	0.99	RARB
rs322667	3	25,344,851	G	A	0.772	0.992	RARB
rs145330094	3	25,345,090	G	A	0.973	0.914	RARB
rs149025838	3	25,345,253	T	A	0.989	0.912	RARB
rs542386743	3	25,347,397	G	C	0.985	0.706	RARB
rs4681045	3	25,347,400	G	C	0.561	0.97	RARB
rs322688	3	25,347,555	G	A	0.772	0.991	RARB
rs322689	3	25,347,602	G	A	0.268	0.95	RARB
rs322691	3	25,348,420	T	C	0.763	0.992	RARB
rs17580540	3	25,348,459	C	G	0.947	0.987	RARB
rs17016164	3	25,348,463	G	A	0.943	0.972	RARB
rs322692	3	25,348,620	G	A	0.655	0.988	RARB
rs322693	3	25,349,369	C	G	0.827	0.999	RARB
rs9809773	3	25,349,414	C	T	0.558	0.971	RARB
rs322694	3	25,349,434	C	A	0.212	0.957	RARB
rs142779713	3	25,349,572	G	C	0.988	0.947	RARB
rs78766919	3	25,349,752	A	G	0.943	0.978	RARB
rs7636859	3	25,349,925	G	A	0.948	0.985	RARB
rs758752943	3	25,349,939	CTT	C	0.815	0.966	RARB
rs322696	3	25,350,004	G	A	0.83	0.995	RARB
rs322697	3	25,350,049	C	T	0.829	0.994	RARB
rs7629182	3	25,350,191	A	G	0.948	0.985	RARB
rs138341692	3	25,350,873	A	G	0.961	0.899	RARB
rs114023837	3	25,350,925	C	T	0.943	0.972	RARB
rs17016167	3	25,351,447	T	A	0.943	0.971	RARB
rs75215193	3	25,351,513	T	C	0.953	0.98	RARB
rs79723475	3	25,351,663	G	C	0.943	0.971	RARB
rs17016169	3	25,352,019	T	A	0.943	0.971	RARB
rs17016172	3	25,352,619	C	T	0.943	0.971	RARB
rs79910788	3	25,352,915	C	T	0.943	0.971	RARB
rs754975	3	25,353,796	C	T	0.822	0.982	RARB
rs322698	3	25,354,249	A	G	0.761	0.979	RARB
rs80046677	3	25,354,318	C	T	0.99	0.851	RARB
rs1483829	3	25,354,349	C	T	0.944	0.987	RARB
rs322699	3	25,354,513	C	T	0.702	0.981	RARB
rs79389509	3	25,354,557	C	G	0.944	0.988	RARB
rs322700	3	25,354,688	G	A	0.702	0.981	RARB
rs322701	3	25,355,041	A	T	0.764	0.982	RARB
rs113613454	3	25,355,239	A	G	0.944	0.988	RARB
rs322702	3	25,355,458	C	A	0.694	0.982	RARB
3:25355740_TTCA_T	3	25,355,740	TTCA	T	0.944	0.985	RARB
rs78998540	3	25,355,984	C	G	0.943	0.988	RARB
rs322703	3	25,356,009	C	A	0.762	0.984	RARB
rs172823	3	25,356,328	C	T	0.694	0.983	RARB
rs9838713	3	25,356,465	T	C	0.693	0.983	RARB
rs322662	3	25,356,873	G	A	0.694	0.984	RARB
3:25357271_AT_A	3	25,357,271	AT	A	0.696	0.975	RARB
rs322663	3	25,357,347	G	T	0.699	0.985	RARB
rs322664	3	25,357,449	C	T	0.977	0.91	RARB
rs9862068	3	25,357,637	A	C	0.461	0.993	RARB
rs9844533	3	25,357,638	T	G	0.461	0.993	RARB
rs1483839	3	25,358,202	G	A	0.943	0.991	RARB
rs17016178	3	25,358,403	C	T	0.45	0.997	RARB
rs61678814	3	25,358,511	T	G	0.451	0.996	RARB
3:25358567_ATAAGTCGTG_A	3	25,358,567	ATAAGTCGTG	A	0.954	0.555	RARB
rs56213898	3	25,358,573	C	A	0.546	0.95	RARB
rs200091739	3	25,358,574	G	T	0.548	0.949	RARB
rs116355465	3	25,358,576	G	A	0.548	0.949	RARB
rs9834664	3	25,359,386	C	T	0.457	0.996	RARB
rs72314552	3	25,359,417	A	ATT	0.45	0.993	RARB
rs35706464	3	25,359,488	T	C	0.45	0.997	RARB
rs1872142	3	25,359,615	C	T	0.451	0.995	RARB
rs1872141	3	25,359,621	G	C	0.514	0.993	RARB
rs7617171	3	25,360,230	C	T	0.451	0.996	RARB
rs75622708	3	25,360,366	T	A	0.902	0.992	RARB
rs1483838	3	25,360,766	A	G	0.45	0.995	RARB
rs11373206	3	25,361,140	A	AT	0.513	0.988	RARB
rs17016182	3	25,361,452	G	A	0.943	0.992	RARB
rs116669101	3	25,361,456	A	G	0.982	0.856	RARB
rs17016185	3	25,361,568	C	T	0.943	0.992	RARB
rs9867006	3	25,361,852	C	G	0.902	0.993	RARB
rs9815989	3	25,362,254	T	C	0.507	0.992	RARB
rs76695848	3	25,362,866	T	G	0.943	0.993	RARB
rs11129192	3	25,362,915	A	G	0.507	0.991	RARB
rs11129193	3	25,362,959	A	G	0.507	0.991	RARB
rs11711833	3	25,363,081	A	G	0.45	0.992	RARB
rs11711864	3	25,363,163	A	G	0.444	0.991	RARB
rs11708300	3	25,363,332	T	C	0.839	0.996	RARB
rs542980653	3	25,363,360	T	TA	0.831	0.963	RARB
rs9877461	3	25,363,620	G	A	0.902	0.996	RARB
rs9822411	3	25,363,943	T	A	0.896	0.997	RARB
rs11917967	3	25,364,176	T	C	0.839	0.997	RARB
rs76908521	3	25,364,233	C	T	0.902	0.997	RARB
rs17016187	3	25,364,289	A	G	0.838	0.998	RARB
rs76081546	3	25,364,292	G	A	0.943	0.997	RARB
rs752610547	3	25,364,619	GGGCTCAAGGCAAAAACATGGA	G	0.896	0.891	RARB
rs1564744	3	25,364,972	A	G	0.943	0.999	RARB
rs2164476	3	25,365,011	A	G	0.832	0.997	RARB
rs76243897	3	25,365,275	T	A	0.839	0.999	RARB
rs77309070	3	25,365,286	T	C	0.839	0.999	RARB
rs322666	3	25,366,144	C	G	0.199	0.978	RARB
rs1561116	3	25,366,205	T	G	0.838	1	RARB
rs9837851	3	25,366,733	T	C	0.838	0.999	RARB
rs9818293	3	25,366,887	C	G	0.838	0.999	RARB
3:25367054_TG_T	3	25,367,054	TG	T	0.896	0.978	RARB
rs376999957	3	25,367,059	GT	G	0.933	0.977	RARB
rs58377501	3	25,367,059	G	T	0.986	0.749	RARB
rs17016204	3	25,367,141	G	A	0.839	0.999	RARB
rs17016205	3	25,367,229	T	A	0.943	0.999	RARB
rs76757605	3	25,368,006	G	T	0.943	0.998	RARB
rs76158621	3	25,368,200	T	C	0.943	0.998	RARB
3:25368319_AT_A	3	25,368,319	AT	A	0.441	0.961	RARB
rs57386420	3	25,368,355	C	G	0.839	0.997	RARB
rs62235569	3	25,368,413	T	A	0.613	0.988	RARB
rs114692329	3	25,368,585	G	A	0.943	0.997	RARB
rs116613940	3	25,368,638	G	C	0.983	0.922	RARB
rs76407238	3	25,368,667	A	G	0.943	0.997	RARB
rs59087853	3	25,368,723	T	A	0.894	0.993	RARB
rs78519469	3	25,368,782	A	G	0.943	0.997	RARB
rs77994504	3	25,368,945	A	G	0.943	0.997	RARB
rs9832914	3	25,369,060	C	T	0.838	0.996	RARB
rs322665	3	25,369,246	C	G	0.207	0.981	RARB
rs9833653	3	25,369,348	G	A	0.896	0.997	RARB
rs6806988	3	25,369,694	G	A	0.838	0.997	RARB
rs6799057	3	25,370,042	A	G	0.833	0.995	RARB
rs113198741	3	25,370,158	T	C	0.943	0.995	RARB
rs147990371	3	25,370,372	G	A	0.988	0.944	RARB
rs138818958	3	25,370,623	C	G	0.896	0.996	RARB
rs147470847	3	25,370,828	G	T	0.943	0.995	RARB
rs144224197	3	25,370,880	C	T	0.901	0.996	RARB
rs9848967	3	25,370,895	A	G	0.838	0.994	RARB
rs777742007	3	25,370,998	TA	T	0.943	0.993	RARB
rs12053850	3	25,371,025	G	C	0.735	0.961	RARB
rs144426475	3	25,371,040	A	T	0.943	0.995	RARB
rs9852984	3	25,371,094	A	T	0.838	0.996	RARB
rs9815892	3	25,371,185	G	A	0.896	0.996	RARB
rs9816512	3	25,371,602	G	A	0.838	0.995	RARB
rs9816521	3	25,371,612	G	C	0.838	0.995	RARB
rs11707346	3	25,371,675	G	A	0.839	0.995	RARB
rs6796134	3	25,371,735	G	A	0.833	0.994	RARB
rs9858501	3	25,372,202	A	G	0.895	0.995	RARB
rs6790649	3	25,372,361	T	C	0.838	0.995	RARB
rs6787933	3	25,372,402	A	G	0.943	0.993	RARB
rs6788260	3	25,372,672	A	G	0.833	0.991	RARB
rs9859468	3	25,372,833	A	G	0.895	0.995	RARB
rs9859796	3	25,373,027	A	G	0.896	0.994	RARB
rs141912220	3	25,373,228	C	G	0.944	0.994	RARB
rs147840687	3	25,373,341	C	T	0.944	0.994	RARB
rs150383099	3	25,373,547	T	C	0.943	0.991	RARB
rs6792022	3	25,373,905	A	G	0.904	0.99	RARB
rs6794637	3	25,373,906	T	C	0.838	0.991	RARB
rs6781934	3	25,374,032	C	T	0.833	0.991	RARB
rs6794641	3	25,374,119	A	C	0.844	0.985	RARB
rs112344888	3	25,374,189	A	G	0.943	0.991	RARB
rs149825507	3	25,374,338	C	T	0.941	0.982	RARB
rs140416195	3	25,374,434	A	C	0.944	0.993	RARB
rs370646695	3	25,374,622	C	T	0.943	0.99	RARB
rs9852354	3	25,374,704	T	C	0.895	0.991	RARB
rs9870001	3	25,374,790	A	C	0.895	0.993	RARB

SNP	Chromosome	Position	Effect Allele	Other Allele	Effect Allele Frequency	Imputation quality	Gene
rs9874712	3	25,375,355	A	G	0.896	0.993	RARB
rs145741795	3	25,375,436	C	T	0.944	0.99	RARB
rs9837715	3	25,375,621	G	A	0.838	0.992	RARB
rs9837763	3	25,375,791	C	T	0.838	0.993	RARB
rs141502759	3	25,375,957	G	A	0.944	0.992	RARB
rs322686	3	25,376,034	C	G	0.322	0.981	RARB
rs9842356	3	25,376,101	G	A	0.838	0.992	RARB
rs147879997	3	25,376,200	A	AAAAT	0.943	0.986	RARB
rs322685	3	25,376,510	G	A	0.763	0.982	RARB
rs1483837	3	25,376,537	T	C	0.838	0.993	RARB
rs116788560	3	25,376,740	T	C	0.945	0.978	RARB
rs199933543	3	25,376,750	C	CTT	0.942	0.984	RARB
rs375461074	3	25,376,753	A	T	0.896	0.992	RARB
rs112683576	3	25,376,754	T	G	0.942	0.984	RARB
rs534434634	3	25,376,756	T	G	0.898	0.985	RARB
rs554216867	3	25,376,757	C	G	0.898	0.985	RARB
3:25376758_TCTC_T	3	25,376,758	TCTC	T	0.898	0.984	RARB
3:25376762_ATG_A	3	25,376,762	ATG	A	0.898	0.985	RARB
rs11720675	3	25,376,779	A	G	0.904	0.977	RARB
rs145058358	3	25,376,953	A	C	0.938	0.951	RARB
rs322684	3	25,377,223	G	A	0.763	0.982	RARB
rs1436251	3	25,377,424	T	C	0.839	0.993	RARB
rs74368804	3	25,377,510	C	T	0.944	0.991	RARB
rs9848513	3	25,377,589	G	A	0.838	0.992	RARB
rs78022710	3	25,377,637	C	T	0.902	0.993	RARB
rs1905465	3	25,377,760	G	T	0.839	0.993	RARB
rs7649070	3	25,377,786	A	G	0.895	0.993	RARB
rs12492794	3	25,378,086	C	T	0.638	0.983	RARB
rs17016213	3	25,378,343	C	T	0.948	0.979	RARB
rs74766083	3	25,378,454	G	A	0.943	0.99	RARB
rs6550961	3	25,378,486	G	A	0.833	0.992	RARB
rs1483836	3	25,378,658	A	G	0.641	0.978	RARB
rs78913643	3	25,378,798	T	C	0.94	0.992	RARB
rs77435197	3	25,378,969	C	G	0.94	0.993	RARB
rs6799363	3	25,379,134	C	G	0.837	0.993	RARB
rs6774199	3	25,379,231	G	T	0.837	0.995	RARB
rs780781676	3	25,379,566	GTCAC	G	0.943	0.997	RARB
rs78497376	3	25,380,868	G	C	0.967	0.998	RARB
rs11920003	3	25,381,021	C	A	0.653	0.992	RARB
rs559916016	3	25,381,072	G	GT	0.706	0.913	RARB
rs11920829	3	25,381,124	C	A	0.596	0.993	RARB
rs74347859	3	25,381,352	A	G	0.894	0.999	RARB
rs9861454	3	25,381,636	T	C	0.59	0.993	RARB
rs141534656	3	25,381,829	G	T	0.979	0.966	RARB
rs10510562	3	25,381,956	G	C	0.701	0.992	RARB
rs6550962	3	25,381,964	A	G	0.894	1	RARB
rs139426471	3	25,382,044	C	T	0.977	0.931	RARB
rs6550964	3	25,382,189	A	T	0.596	0.993	RARB
rs10510563	3	25,382,623	G	A	0.759	0.992	RARB
rs7613965	3	25,382,659	G	C	0.596	0.994	RARB
rs79287399	3	25,382,692	G	A	0.9	0.999	RARB
rs77820862	3	25,382,817	A	T	0.894	1	RARB
rs4681017	3	25,382,822	T	C	0.596	0.994	RARB
rs76173053	3	25,383,121	A	G	0.943	0.999	RARB
rs148922239	3	25,383,254	G	A	0.988	0.919	RARB
rs4681047	3	25,383,258	A	G	0.598	0.989	RARB
rs4681048	3	25,383,268	C	G	0.598	0.989	RARB
rs75852777	3	25,383,273	C	G	0.944	0.994	RARB
rs6550965	3	25,383,587	C	A	0.591	0.994	RARB
rs1483831	3	25,383,666	G	A	0.765	0.993	RARB
rs1483830	3	25,383,713	A	G	0.595	0.994	RARB
rs10510564	3	25,383,892	A	G	0.961	0.982	RARB
rs57697446	3	25,384,139	G	C	0.894	0.999	RARB
rs79016453	3	25,384,451	G	C	0.943	0.998	RARB
rs4681018	3	25,384,461	A	G	0.603	0.995	RARB
3:25384515_TTGTG_T	3	25,384,515	TTGTG	T	0.62	0.971	RARB
rs74768553	3	25,384,746	G	A	0.943	0.998	RARB
rs531239937	3	25,384,947	C	CTT	0.894	0.993	RARB
rs114681300	3	25,384,967	A	G	0.948	0.97	RARB
rs7615094	3	25,385,111	T	C	0.9	0.998	RARB
rs78104505	3	25,385,246	C	G	0.943	0.998	RARB
rs7623030	3	25,385,415	G	C	0.899	0.998	RARB
rs7615609	3	25,385,649	T	C	0.843	0.998	RARB
rs80323488	3	25,385,739	C	A	0.945	0.988	RARB
rs113627705	3	25,385,746	C	T	0.944	0.987	RARB
rs78063326	3	25,385,756	A	C	0.945	0.988	RARB
rs78883327	3	25,385,879	T	G	0.964	0.973	RARB
rs11709169	3	25,386,018	A	G	0.843	0.998	RARB
rs11129194	3	25,386,058	A	T	0.609	0.996	RARB
rs73051691	3	25,386,103	G	T	0.938	0.959	RARB
rs75225035	3	25,386,149	C	T	0.944	0.998	RARB
rs34015957	3	25,386,194	G	A	0.766	0.996	RARB
rs11129195	3	25,386,315	G	A	0.609	0.996	RARB
rs11717848	3	25,386,325	C	T	0.9	0.998	RARB
rs58464421	3	25,386,438	G	GTTTGTA	0.597	0.994	RARB
rs6771956	3	25,386,592	T	C	0.842	0.998	RARB
rs11718046	3	25,386,646	G	T	0.9	0.999	RARB
rs6769438	3	25,386,696	A	G	0.943	0.997	RARB
rs11129196	3	25,386,814	C	G	0.664	0.998	RARB
rs114591161	3	25,386,866	C	A	0.943	0.998	RARB
rs12496273	3	25,387,394	G	A	0.766	0.998	RARB
rs145867879	3	25,387,473	G	A	0.985	0.854	RARB
rs12496365	3	25,387,754	G	C	0.764	0.999	RARB
rs6775569	3	25,387,962	A	G	0.607	0.998	RARB
rs79871826	3	25,388,173	G	A	0.943	0.998	RARB
rs12497123	3	25,388,313	C	A	0.764	0.999	RARB
rs6550966	3	25,388,856	G	C	0.9	0.999	RARB
rs10510565	3	25,388,890	A	G	0.943	0.997	RARB
rs10510566	3	25,389,249	C	T	0.763	0.998	RARB
rs11721271	3	25,389,504	C	T	0.9	0.998	RARB
rs1075410	3	25,389,853	C	T	0.707	0.999	RARB
rs729236	3	25,390,429	C	G	0.666	0.998	RARB
rs75986625	3	25,390,634	C	T	0.944	0.997	RARB
rs13080165	3	25,391,116	A	G	0.763	0.998	RARB
rs11709732	3	25,391,348	A	C	0.901	0.998	RARB
rs75755090	3	25,392,238	A	G	0.944	0.997	RARB
rs17016253	3	25,392,379	G	T	0.944	0.997	RARB
rs17016257	3	25,392,513	A	G	0.943	0.997	RARB
rs17016260	3	25,392,580	A	G	0.894	0.998	RARB
rs11718836	3	25,392,772	G	A	0.894	0.998	RARB
rs13093379	3	25,393,671	A	C	0.763	0.997	RARB
rs13073683	3	25,393,837	T	C	0.6	0.997	RARB
rs11712065	3	25,393,860	A	G	0.763	0.997	RARB
3:25395075_AT_A	3	25,395,075	AT	A	0.265	0.93	RARB
rs6784604	3	25,395,403	T	G	0.22	0.995	RARB
rs55876605	3	25,395,520	A	T	0.763	0.995	RARB
rs61694119	3	25,395,695	C	T	0.763	0.995	RARB
rs17588514	3	25,396,077	A	T	0.938	0.974	RARB
rs4681049	3	25,396,313	C	T	0.223	0.993	RARB
rs112055165	3	25,396,513	A	G	0.988	0.86	RARB
rs77529283	3	25,396,655	G	A	0.944	0.994	RARB
rs6550967	3	25,396,760	G	T	0.673	0.97	RARB
rs17588596	3	25,396,865	C	T	0.9	0.997	RARB
rs576386494	3	25,398,009	C	CT	0.895	0.982	RARB
rs55702004	3	25,398,113	A	G	0.796	0.977	RARB
rs113532687	3	25,398,123	T	G	0.879	0.977	RARB
rs7646918	3	25,398,138	G	A	0.222	0.991	RARB
rs12631631	3	25,398,175	G	A	0.667	0.988	RARB
rs146054248	3	25,398,458	A	G	0.947	0.995	RARB
rs7637182	3	25,398,513	A	T	0.895	0.997	RARB
rs7637258	3	25,398,610	A	T	0.515	0.995	RARB
rs144478167	3	25,398,721	C	T	0.982	0.9	RARB
rs142001230	3	25,398,881	C	T	0.9	0.999	RARB
rs144473687	3	25,398,907	T	C	0.951	0.994	RARB
rs141297383	3	25,399,304	G	A	0.953	0.968	RARB
rs6798613	3	25,399,594	T	C	0.613	0.996	RARB
rs145872326	3	25,399,687	A	C	0.951	0.995	RARB
rs9871477	3	25,399,736	A	G	0.507	0.995	RARB
rs139930246	3	25,399,769	G	A	0.977	0.93	RARB
rs9853916	3	25,399,781	T	C	0.514	0.996	RARB
rs9834082	3	25,399,881	C	T	0.9	0.999	RARB
rs140244335	3	25,400,006	A	G	0.9	0.999	RARB
rs9810741	3	25,400,476	C	A	0.618	0.995	RARB
rs35714766	3	25,400,548	G	A	0.216	0.993	RARB
rs111563930	3	25,400,903	T	A	0.659	0.984	RARB
rs13084031	3	25,400,911	T	A	0.264	0.989	RARB
rs150398888	3	25,401,030	C	A	0.764	0.994	RARB
rs59816658	3	25,401,099	A	G	0.9	0.999	RARB
rs9815423	3	25,401,139	C	G	0.265	0.994	RARB
rs62235603	3	25,401,174	C	A	0.758	0.994	RARB
rs9835390	3	25,401,189	T	G	0.508	0.996	RARB
rs9835396	3	25,401,196	T	G	0.508	0.996	RARB
rs9835679	3	25,401,399	T	G	0.614	0.996	RARB
rs9853484	3	25,401,528	A	T	0.216	0.994	RARB
rs146736650	3	25,401,595	G	A	0.951	0.995	RARB

SNP	Chromosome	Position	Effect Allele	Other Allele	Effect Allele Frequency	Imputation quality	Gene
rs2200244	3	25,401,957	C	G	0.755	0.994	RARB
rs116605026	3	25,402,082	T	G	0.9	0.999	RARB
3:25402216_CCTTCACTTATA_C	3	25,402,216	CCTTCACTTATA	C	0.894	0.987	RARB
3:25402225_A_C	3	25,402,225	A	C	0.986	0.577	RARB
3:25402227_A_C	3	25,402,227	A	C	0.986	0.576	RARB
rs11916922	3	25,402,449	G	T	0.757	0.995	RARB
rs7633308	3	25,402,758	A	G	0.894	0.998	RARB
rs138496078	3	25,402,941	G	A	0.989	0.888	RARB
rs11716202	3	25,402,974	A	G	0.894	0.998	RARB
rs7645983	3	25,403,182	G	A	0.219	0.995	RARB
rs11713396	3	25,403,207	T	G	0.9	0.999	RARB
rs116133348	3	25,403,358	G	A	0.951	0.995	RARB
rs7638495	3	25,403,379	T	C	0.22	0.994	RARB
rs116607405	3	25,403,380	G	A	0.951	0.99	RARB
rs7646182	3	25,403,391	G	A	0.464	0.997	RARB
rs11709436	3	25,403,574	C	A	0.9	0.999	RARB
rs7646375	3	25,403,588	G	C	0.621	0.996	RARB
rs7638687	3	25,403,597	T	A	0.621	0.996	RARB
rs8179975	3	25,403,740	C	T	0.62	0.998	RARB
rs11709623	3	25,403,944	G	A	0.9	0.999	RARB
rs11925814	3	25,403,962	A	G	0.95	0.994	RARB
rs60917922	3	25,404,008	G	T	0.951	0.995	RARB
rs8180044	3	25,404,023	T	C	0.462	0.997	RARB
rs57064248	3	25,404,026	G	A	0.951	0.995	RARB
rs13325064	3	25,404,136	A	G	0.52	0.998	RARB
rs11714481	3	25,404,153	T	A	0.9	0.999	RARB
rs73156056	3	25,404,565	C	T	0.951	0.995	RARB
rs76917500	3	25,404,773	A	G	0.894	0.998	RARB
rs79834251	3	25,404,952	G	T	0.9	0.999	RARB
rs2086790	3	25,405,074	G	T	0.95	0.993	RARB
rs35569984	3	25,405,732	A	G	0.761	0.996	RARB
rs1384899	3	25,405,921	A	G	0.46	0.998	RARB
rs9310778	3	25,406,188	G	A	0.569	0.999	RARB
rs4681051	3	25,406,260	A	G	0.219	0.996	RARB
rs9310779	3	25,406,266	C	G	0.619	0.999	RARB
rs1016786	3	25,406,476	G	A	0.569	0.999	RARB
rs1016787	3	25,406,510	T	C	0.567	0.999	RARB
rs9880591	3	25,406,750	A	T	0.569	1	RARB
rs77674036	3	25,406,776	A	C	0.9	1	RARB
rs7636904	3	25,406,981	C	G	0.619	0.998	RARB
rs7648222	3	25,407,002	A	C	0.569	0.998	RARB
rs77018420	3	25,407,046	A	G	0.951	0.996	RARB
rs1531871	3	25,407,268	A	G	0.46	0.998	RARB
rs1026929	3	25,407,294	G	C	0.568	0.999	RARB
rs1026930	3	25,407,502	T	A	0.939	0.993	RARB
rs7614710	3	25,407,596	G	A	0.618	0.999	RARB
rs7648812	3	25,407,613	A	G	0.618	0.999	RARB
rs1483842	3	25,407,695	T	C	0.518	0.998	RARB
rs1473452	3	25,407,845	C	G	0.619	0.999	RARB
rs9853460	3	25,408,451	G	A	0.618	0.999	RARB
rs922942	3	25,408,621	T	C	0.569	1	RARB
rs11707139	3	25,408,701	A	C	0.9	1	RARB
rs17588729	3	25,408,709	A	G	0.757	0.996	RARB
rs9310780	3	25,409,021	G	A	0.569	1	RARB
3:25409143_TA_T	3	25,409,143	TA	T	0.365	0.936	RARB
rs1351782	3	25,409,211	G	C	0.567	1	RARB
rs1351783	3	25,409,319	C	A	0.568	0.998	RARB
rs1351784	3	25,409,393	A	G	0.219	0.996	RARB
rs1351785	3	25,409,443	C	T	0.619	0.999	RARB
rs1483843	3	25,409,842	T	C	0.219	0.995	RARB
rs7623744	3	25,410,250	G	C	0.616	0.998	RARB
rs17588847	3	25,410,341	C	T	0.969	0.964	RARB
rs35946440	3	25,410,868	C	T	0.764	0.995	RARB
rs9836368	3	25,411,023	C	T	0.22	0.994	RARB
rs79353748	3	25,411,066	G	A	0.951	0.995	RARB
rs13094358	3	25,411,118	A	T	0.763	0.995	RARB
rs150177281	3	25,411,327	C	T	0.902	0.999	RARB
rs9860987	3	25,411,443	T	C	0.616	0.998	RARB
rs76838940	3	25,411,612	G	T	0.951	0.995	RARB
rs4681019	3	25,411,681	C	G	0.763	0.994	RARB
3:25411759_TAAATTA_T	3	25,411,759	TAAATTA	T	0.951	0.992	RARB
rs900656	3	25,412,388	C	G	0.57	0.997	RARB
rs1108138	3	25,412,484	C	T	0.764	0.993	RARB
rs900657	3	25,412,533	G	T	0.57	0.995	RARB
rs900658	3	25,412,538	C	A	0.57	0.995	RARB
rs1809485	3	25,412,563	C	T	0.57	0.995	RARB
rs74737739	3	25,412,890	A	G	0.96	0.928	RARB
rs752152	3	25,413,218	A	G	0.567	0.996	RARB
rs6769856	3	25,413,451	G	T	0.568	0.996	RARB
rs1905467	3	25,413,760	A	G	0.567	0.995	RARB
rs75363640	3	25,414,353	T	A	0.949	0.99	RARB
rs13070614	3	25,414,588	C	G	0.783	0.99	RARB
rs78627339	3	25,414,724	C	T	0.949	0.992	RARB
rs78099948	3	25,415,019	T	G	0.951	0.997	RARB
rs60519915	3	25,415,144	T	C	0.95	0.997	RARB
rs80118256	3	25,415,541	T	C	0.951	0.997	RARB
rs61165804	3	25,415,704	A	G	0.951	0.995	RARB
rs116416082	3	25,415,710	A	C	0.951	0.995	RARB
rs76279422	3	25,415,739	G	C	0.951	0.996	RARB
rs17016308	3	25,415,782	C	G	0.95	0.997	RARB
rs73156093	3	25,416,106	A	G	0.95	0.996	RARB
rs2363597	3	25,416,205	A	G	0.154	0.995	RARB
rs1072823	3	25,416,508	C	G	0.56	0.991	RARB
rs79145553	3	25,416,516	C	A	0.951	0.999	RARB
rs113882109	3	25,416,536	C	CA	0.951	0.998	RARB
rs113727973	3	25,416,586	C	T	0.951	0.997	RARB
rs113264205	3	25,416,712	C	T	0.951	0.994	RARB
rs116270656	3	25,416,725	A	C	0.951	0.994	RARB
rs111899953	3	25,416,729	C	T	0.951	0.994	RARB
rs79160865	3	25,416,865	C	T	0.951	1	RARB
rs150644112	3	25,417,310	A	G	0.984	0.97	RARB
rs74869619	3	25,417,601	A	G	0.951	0.999	RARB
rs60112210	3	25,417,811	T	G	0.95	0.998	RARB
rs59064731	3	25,417,842	C	G	0.95	0.998	RARB
rs17016320	3	25,417,956	C	T	0.951	0.998	RARB
rs60207100	3	25,417,984	G	GT	0.95	0.994	RARB
rs1864911	3	25,418,008	G	A	0.171	0.995	RARB
rs61558701	3	25,418,298	C	T	0.95	0.998	RARB
rs73157807	3	25,418,462	A	G	0.95	0.997	RARB
rs111690181	3	25,418,503	G	GTTTT	0.95	0.995	RARB
rs900659	3	25,418,507	A	T	0.176	0.98	RARB
rs73157810	3	25,418,543	T	G	0.95	0.997	RARB
rs56397774	3	25,418,643	A	C	0.951	0.99	RARB
rs4681053	3	25,418,646	T	C	0.22	0.994	RARB
rs111908571	3	25,418,649	G	C	0.95	0.995	RARB
3:25418654_TAAC_T	3	25,418,654	TAAC	T	0.952	0.979	RARB
rs9823425	3	25,418,758	T	C	0.554	0.99	RARB
rs73820454	3	25,418,764	G	A	0.951	0.997	RARB
rs17016328	3	25,418,899	T	C	0.951	0.997	RARB
rs56167545	3	25,419,177	A	G	0.951	0.996	RARB
rs77889354	3	25,419,433	C	G	0.951	0.996	RARB
rs12628995	3	25,419,675	G	C	0.56	0.989	RARB
rs2195919	3	25,420,903	T	A	0.566	0.99	RARB
rs76129294	3	25,421,173	C	G	0.95	0.995	RARB
rs77678090	3	25,421,319	G	A	0.95	0.994	RARB
rs6768396	3	25,421,707	A	G	0.17	0.997	RARB
rs17016334	3	25,421,996	T	C	0.95	0.995	RARB
rs60838657	3	25,422,032	C	T	0.95	0.995	RARB
rs749471535	3	25,422,087	AAAAT	A	0.84	0.981	RARB
rs7628351	3	25,422,475	G	C	0.22	0.996	RARB
rs7628541	3	25,422,696	G	A	0.22	0.996	RARB
rs7618590	3	25,422,776	A	G	0.17	0.997	RARB
rs34075235	3	25,422,886	G	A	0.828	0.985	RARB
rs17016343	3	25,423,072	G	C	0.95	0.995	RARB
rs9876345	3	25,423,265	C	T	0.565	0.99	RARB
rs17523645	3	25,423,431	G	A	0.839	0.987	RARB
rs9839276	3	25,423,766	A	G	0.175	0.998	RARB
rs9839284	3	25,423,781	A	G	0.564	0.991	RARB
rs142139973	3	25,423,902	A	AC	0.174	0.996	RARB
rs11442172	3	25,423,903	T	C	0.696	0.658	RARB
rs145578536	3	25,424,075	G	A	0.95	0.994	RARB
rs148954150	3	25,424,226	G	C	0.966	0.893	RARB
rs1159899	3	25,424,428	G	A	0.175	0.999	RARB
rs139007908	3	25,424,548	C	G	0.986	0.867	RARB
rs2306623	3	25,424,929	T	C	0.334	0.994	RARB
rs191610296	3	25,425,156	C	T	0.986	0.869	RARB
rs6781619	3	25,425,217	A	G	0.158	0.996	RARB
rs6784498	3	25,425,373	T	A	0.508	0.991	RARB
rs11401651	3	25,425,421	C	CA	0.521	0.963	RARB
rs7627850	3	25,425,703	A	G	0.161	0.995	RARB
3:25425769_CTTCTCTTTCTCCCTTCA_C	3	25,425,769	CTTCTCTTTCTCCCTTCA	C	0.951	0.992	RARB
rs74928855	3	25,425,858	G	A	0.944	0.99	RARB
rs2291296	3	25,425,882	G	A	0.827	0.988	RARB
rs1483844	3	25,426,279	C	G	0.515	0.99	RARB
rs11129198	3	25,426,975	T	C	0.824	0.983	RARB

SNP	Chromosome	Position	Effect Allele	Other Allele	Effect Allele Frequency	Imputation quality	Gene
rs142267360	3	25,427,132	T	TAA	0.987	0.988	RARB
rs17016356	3	25,427,985	G	T	0.95	0.99	RARB
rs1384901	3	25,428,403	A	C	0.39	0.984	RARB
rs79116543	3	25,429,212	T	A	0.984	0.881	RARB
rs11714772	3	25,429,291	T	C	0.302	0.981	RARB
rs56059341	3	25,429,528	G	T	0.579	0.982	RARB
rs34768755	3	25,429,545	G	GA	0.357	0.972	RARB
rs17523903	3	25,429,659	A	G	0.829	0.995	RARB
rs7653107	3	25,430,069	G	T	0.835	0.995	RARB
rs148181205	3	25,430,197	G	A	0.985	0.968	RARB
rs1351786	3	25,431,140	A	C	0.354	0.98	RARB
rs115734842	3	25,431,279	A	G	0.95	0.986	RARB
rs114261076	3	25,431,296	G	A	0.966	0.936	RARB
rs6784301	3	25,431,389	A	G	0.357	0.981	RARB
rs17016367	3	25,431,473	G	A	0.95	0.985	RARB
rs116470044	3	25,431,831	C	T	0.948	0.992	RARB
rs1991786	3	25,432,062	G	A	0.579	0.98	RARB
3:25432079_CA_C	3	25,432,079	CA	C	0.828	0.989	RARB
rs2363598	3	25,432,090	A	G	0.579	0.98	RARB
rs57185311	3	25,432,108	T	C	0.829	0.996	RARB
rs151043487	3	25,432,374	A	G	0.969	0.983	RARB
rs144944506	3	25,432,389	A	AT	0.776	0.979	RARB
rs1155109	3	25,432,395	G	T	0.163	0.973	RARB
rs74593305	3	25,432,520	A	G	0.95	0.984	RARB
rs1155108	3	25,432,636	T	C	0.985	0.868	RARB
rs62237587	3	25,432,790	T	C	0.83	0.997	RARB
rs13073246	3	25,432,885	C	T	0.83	0.997	RARB
rs13073255	3	25,432,899	C	G	0.835	0.997	RARB
rs9821864	3	25,432,961	C	A	0.565	0.988	RARB
rs4681054	3	25,433,164	G	A	0.83	0.998	RARB
rs4681055	3	25,433,310	A	G	0.83	0.997	RARB
rs4681056	3	25,433,368	A	G	0.83	0.998	RARB
rs4681057	3	25,433,594	G	A	0.83	0.998	RARB
3:25433864_CT_C	3	25,433,864	CT	C	0.829	0.995	RARB
rs116069623	3	25,433,895	T	C	0.951	0.984	RARB
rs11717239	3	25,433,899	A	G	0.83	0.999	RARB
rs115620421	3	25,433,924	G	A	0.988	0.963	RARB
rs6797088	3	25,434,154	T	C	0.83	0.998	RARB
rs6794508	3	25,434,161	A	C	0.83	0.998	RARB
rs6797302	3	25,434,385	T	C	0.83	0.999	RARB
rs6794839	3	25,434,491	A	G	0.729	0.991	RARB
rs6794841	3	25,434,498	A	G	0.83	0.999	RARB
rs11710506	3	25,434,643	C	G	0.83	0.999	RARB
rs12489308	3	25,434,761	G	A	0.83	0.999	RARB
rs1961005	3	25,435,001	T	G	0.95	0.984	RARB
rs12489364	3	25,435,027	G	A	0.83	0.999	RARB
rs11715691	3	25,435,570	T	C	0.829	1	RARB
rs1843028	3	25,435,979	G	C	0.158	0.988	RARB
rs1384900	3	25,436,126	T	C	0.158	0.988	RARB
rs56252700	3	25,436,215	T	C	0.83	0.998	RARB
rs11712725	3	25,436,670	C	G	0.969	0.933	RARB
rs7634400	3	25,436,797	C	T	0.829	0.998	RARB
rs202144596	3	25,437,094	TC	T	0.946	0.982	RARB
rs7650761	3	25,437,201	T	C	0.829	0.998	RARB
rs59034168	3	25,437,267	A	G	0.95	0.981	RARB
rs62237590	3	25,437,648	A	C	0.829	0.998	RARB
rs114319530	3	25,437,737	G	A	0.945	0.992	RARB
rs77649742	3	25,437,943	G	A	0.95	0.981	RARB
rs78339709	3	25,438,107	G	A	0.945	0.991	RARB
rs144594145	3	25,438,248	T	A	0.988	0.84	RARB
rs13088742	3	25,438,508	T	C	0.829	0.996	RARB
rs75815317	3	25,438,515	G	A	0.947	0.989	RARB
rs2363599	3	25,438,617	T	G	0.158	0.987	RARB
rs73820467	3	25,438,644	T	C	0.896	0.986	RARB
rs74694178	3	25,438,840	C	CT	0.157	0.979	RARB
rs6799176	3	25,439,154	C	A	0.895	0.986	RARB
rs73055789	3	25,439,235	T	C	0.938	0.929	RARB
rs566176640	3	25,439,376	T	TAG	0.357	0.777	RARB
rs11370692	3	25,439,378	G	GA	0.154	0.963	RARB
rs74718943	3	25,439,489	A	G	0.95	0.98	RARB
rs12330681	3	25,439,543	G	C	0.402	0.984	RARB
rs12374126	3	25,439,846	C	G	0.869	0.983	RARB
rs4681058	3	25,440,041	A	G	0.751	0.985	RARB
rs9834839	3	25,440,105	C	T	0.354	0.983	RARB
rs79827017	3	25,440,108	C	T	0.896	0.985	RARB
3:25440516_CTCT_C	3	25,440,516	CTCT	C	0.895	0.975	RARB
rs6550969	3	25,440,835	T	C	0.895	0.985	RARB
rs6550970	3	25,440,883	A	G	0.895	0.985	RARB
rs6802733	3	25,441,039	T	C	0.895	0.985	RARB
rs12490801	3	25,441,463	G	A	0.862	0.986	RARB
rs12374188	3	25,441,802	A	G	0.868	0.983	RARB
rs2116701	3	25,441,977	A	G	0.158	0.986	RARB
rs2116702	3	25,442,051	A	C	0.402	0.984	RARB
rs922941	3	25,442,508	C	T	0.353	0.983	RARB
rs962926	3	25,442,809	T	A	0.354	0.983	RARB
rs2885838	3	25,443,204	G	T	0.353	0.982	RARB
rs34042056	3	25,443,307	G	A	0.805	0.983	RARB
rs2885839	3	25,443,350	C	A	0.354	0.982	RARB
rs7616561	3	25,443,361	G	A	0.354	0.982	RARB
rs59648542	3	25,443,467	C	A	0.869	0.983	RARB
rs7639526	3	25,443,552	C	G	0.354	0.982	RARB
rs7651077	3	25,443,645	A	G	0.353	0.982	RARB
rs6550971	3	25,443,691	T	C	0.353	0.982	RARB
rs4681059	3	25,443,997	C	A	0.159	0.985	RARB
rs9876246	3	25,444,148	T	A	0.354	0.982	RARB
rs73055798	3	25,444,367	T	A	0.701	0.981	RARB
rs148110029	3	25,444,641	G	C	0.948	0.963	RARB
rs73141611	3	25,445,019	C	T	0.895	0.983	RARB
3:25445167_CTG_C	3	25,445,167	CTG	C	0.987	0.93	RARB
rs4395373	3	25,445,347	C	T	0.808	0.968	RARB
rs546197894	3	25,445,377	T	TAA	0.507	0.911	RARB
rs6768560	3	25,445,760	T	A	0.585	0.981	RARB
rs36013124	3	25,446,190	C	T	0.812	0.98	RARB
rs6550972	3	25,446,352	C	T	0.584	0.981	RARB
rs716161	3	25,446,586	T	G	0.27	0.983	RARB
rs716160	3	25,446,952	T	C	0.583	0.981	RARB
rs11711018	3	25,447,145	A	G	0.869	0.983	RARB
rs79113274	3	25,447,420	C	T	0.969	0.984	RARB
rs3074645	3	25,447,456	T	TGTTGA	0.212	0.977	RARB
rs7619119	3	25,447,521	A	G	0.584	0.982	RARB
rs6783726	3	25,447,603	G	A	0.687	0.984	RARB
rs80181836	3	25,447,633	C	T	0.95	0.975	RARB
rs1864906	3	25,448,217	A	G	0.156	0.982	RARB
rs1483841	3	25,448,823	A	G	0.843	0.989	RARB
rs9840999	3	25,448,930	A	G	0.374	0.989	RARB
rs56032509	3	25,448,931	C	G	0.619	0.989	RARB
rs75043279	3	25,449,080	G	T	0.918	0.993	RARB
3:25449249_CT_C	3	25,449,249	CT	C	0.211	0.95	RARB
rs9845401	3	25,449,279	A	G	0.612	0.993	RARB
rs9883318	3	25,449,409	C	A	0.629	0.994	RARB
rs1351781	3	25,449,961	G	A	0.22	0.996	RARB
rs76135691	3	25,450,046	G	A	0.919	0.995	RARB
rs1991787	3	25,450,072	C	T	0.789	0.997	RARB
rs1991788	3	25,450,392	A	G	0.222	0.996	RARB
rs142737624	3	25,450,439	C	T	0.987	0.944	RARB
rs6550974	3	25,450,943	G	A	0.413	1	RARB
rs7630502	3	25,451,024	G	T	0.413	1	RARB
rs11716604	3	25,451,031	C	T	0.806	0.998	RARB
rs7620529	3	25,451,077	A	C	0.413	1	RARB
rs2364117	3	25,451,254	T	C	0.413	1	RARB
rs2364118	3	25,451,461	G	A	0.219	0.999	RARB
rs13085878	3	25,452,087	C	T	0.806	0.997	RARB
rs4681061	3	25,452,392	T	C	0.806	0.997	RARB
rs79025451	3	25,453,856	C	T	0.888	0.995	RARB
rs1994176	3	25,453,857	A	G	0.219	1	RARB
rs4681020	3	25,454,126	T	C	0.025	0.951	RARB
rs142731314	3	25,454,199	A	C	0.984	0.963	RARB
rs4681021	3	25,454,265	T	C	0.219	0.999	RARB
rs4681022	3	25,454,281	C	T	0.805	0.996	RARB
rs4681023	3	25,454,308	T	C	0.219	0.999	RARB
rs7618948	3	25,454,458	C	T	0.608	0.997	RARB
3:25454767_ACATT_A	3	25,454,767	ACATT	A	0.413	0.994	RARB
rs1384898	3	25,454,948	A	G	0.495	0.996	RARB
rs1483828	3	25,455,402	T	C	0.413	0.996	RARB
rs547408846	3	25,455,557	G	A	0.963	0.928	RARB
rs5847354	3	25,455,566	T	TC	0.412	0.991	RARB
rs11706561	3	25,455,843	G	A	0.826	0.988	RARB
rs147117204	3	25,455,979	A	G	0.981	0.963	RARB
rs147708035	3	25,456,251	G	A	0.985	0.947	RARB
rs1483827	3	25,457,121	G	A	0.24	0.989	RARB
rs143025006	3	25,457,244	C	T	0.984	0.917	RARB
rs1483826	3	25,457,426	G	A	0.239	0.988	RARB
rs1897500	3	25,457,895	T	A	0.239	0.986	RARB
rs17016385	3	25,458,018	G	C	0.918	0.992	RARB
rs1897501	3	25,458,100	G	T	0.239	0.984	RARB

SNP	Chromosome	Position	Effect Allele	Other Allele	Effect Allele Frequency	Imputation quality	Gene
rs551542519	3	25,458,689	T	TA	0.645	0.894	RARB
rs75348284	3	25,458,742	G	A	0.919	0.956	RARB
rs75305803	3	25,458,778	C	T	0.919	0.954	RARB
rs2014178	3	25,458,936	C	A	0.22	0.949	RARB
rs1812890	3	25,459,108	G	A	0.621	0.938	RARB
rs7634488	3	25,459,393	C	G	0.422	0.93	RARB
rs7648325	3	25,459,497	T	C	0.424	0.923	RARB
3:25460103_GA_G	3	25,460,103	GA	G	0.935	0.831	RARB
rs556021982	3	25,460,435	C	CAAA	0.867	0.799	RARB
rs35494234	3	25,460,535	A	ATT	0.623	0.783	RARB
rs6805482	3	25,460,596	A	G	0.357	0.856	RARB
rs9865116	3	25,461,807	C	T	0.923	0.944	RARB
rs6778608	3	25,461,836	C	T	0.245	0.95	RARB
3:25462480_GC_G	3	25,462,480	GC	G	0.246	0.932	RARB
rs140303743	3	25,462,999	T	C	0.923	0.953	RARB
rs28760501	3	25,463,030	G	A	0.57	0.963	RARB
rs150167226	3	25,463,146	C	T	0.923	0.954	RARB
rs138570080	3	25,463,379	G	A	0.986	0.888	RARB
rs76679572	3	25,463,725	C	T	0.923	0.957	RARB
rs17016408	3	25,463,842	G	C	0.871	0.949	RARB
rs922939	3	25,465,008	C	A	0.348	0.972	RARB
rs922938	3	25,465,136	A	T	0.106	0.968	RARB
rs78180575	3	25,466,176	G	A	0.803	0.975	RARB
rs74825314	3	25,466,677	T	C	0.922	0.963	RARB
rs12630664	3	25,467,155	C	T	0.922	0.963	RARB
rs12630816	3	25,467,748	C	T	0.935	0.967	RARB
3:25472479_CT_C	3	25,472,479	CT	C	0.511	0.901	RARB
rs78311930	3	25,473,206	C	T	0.936	0.979	RARB
rs146373842	3	25,473,617	T	C	0.978	0.973	RARB
rs1483856	3	25,473,945	G	T	0.114	0.99	RARB
rs12635733	3	25,475,836	T	C	0.932	0.98	RARB
rs4681024	3	25,475,882	T	C	0.112	0.992	RARB
rs4681025	3	25,475,924	G	A	0.112	0.991	RARB
rs143711060	3	25,475,928	A	G	0.932	0.977	RARB
rs76532059	3	25,475,980	G	C	0.932	0.979	RARB
3:25476020_CTT_C	3	25,476,020	CTT	C	0.38	0.91	RARB
rs140942783	3	25,476,217	G	C	0.976	0.933	RARB
rs114786935	3	25,476,804	G	A	0.932	0.98	RARB
rs76969640	3	25,477,003	T	C	0.747	0.989	RARB
rs79599137	3	25,477,369	A	T	0.786	0.99	RARB
rs7613553	3	25,477,412	C	A	0.536	0.989	RARB
rs74471895	3	25,478,606	A	G	0.784	0.987	RARB
3:25478608_CT_C	3	25,478,608	CT	C	0.665	0.904	RARB
rs77815743	3	25,478,674	G	A	0.918	0.985	RARB
rs74934780	3	25,479,218	G	C	0.787	0.979	RARB
rs4416353	3	25,479,778	G	A	0.163	0.995	RARB
rs755141480	3	25,479,818	GCCCAGAT	G	0.919	0.981	RARB
rs9284855	3	25,479,838	T	A	0.977	0.998	RARB
rs9284856	3	25,479,983	G	A	0.895	0.99	RARB
rs74471411	3	25,480,001	C	G	0.918	0.988	RARB
rs9871002	3	25,480,125	A	T	0.851	0.993	RARB
rs6550975	3	25,480,368	C	G	0.746	0.984	RARB
rs6550976	3	25,480,678	C	T	0.377	0.998	RARB
3:25480800_TA_T	3	25,480,800	TA	T	0.853	0.99	RARB
rs150000184	3	25,480,928	G	T	0.967	0.927	RARB
rs6776706	3	25,481,222	T	A	0.665	0.996	RARB
rs6785386	3	25,481,239	G	A	0.977	0.999	RARB
rs748439554	3	25,481,584	ATGTATTTGAC	A	0.669	0.988	RARB
rs6762157	3	25,481,699	C	T	0.895	0.989	RARB
rs6785809	3	25,481,709	G	A	0.977	0.998	RARB
rs6762247	3	25,481,797	C	T	0.895	0.989	RARB
rs2364119	3	25,481,968	A	T	0.508	0.959	RARB
rs114165336	3	25,482,135	G	A	0.964	0.988	RARB
rs9310781	3	25,482,147	C	G	0.778	0.977	RARB
rs73042239	3	25,482,432	G	T	0.416	0.987	RARB
rs112174475	3	25,482,579	C	T	0.788	0.977	RARB
rs112081909	3	25,483,599	T	A	0.732	0.969	RARB
rs533266314	3	25,483,607	T	A	0.789	0.969	RARB
rs59704791	3	25,483,690	T	A	0.518	0.976	RARB
rs78847535	3	25,484,676	A	G	0.912	0.972	RARB
3:25484912_CT_C	3	25,484,912	CT	C	0.634	0.964	RARB
rs534388511	3	25,485,113	T	TA	0.941	0.827	RARB
rs184415741	3	25,485,115	A	T	0.96	0.924	RARB
rs6775404	3	25,485,229	C	G	0.699	0.957	RARB
rs113402714	3	25,485,391	C	T	0.964	0.978	RARB
rs12636426	3	25,485,721	C	G	0.918	0.982	RARB
rs12631063	3	25,485,756	A	G	0.918	0.982	RARB
rs6799734	3	25,485,811	G	C	0.633	0.977	RARB
rs76913742	3	25,486,760	C	G	0.918	0.981	RARB
rs9870744	3	25,487,117	C	T	0.822	0.97	RARB
rs6795711	3	25,487,914	A	G	0.941	0.952	RARB
3:25488293_CAAAAAAAAAAAAAA_C	3	25,488,293	CAAAAAAAAAAAAAA	C	0.57	0.857	RARB
3:25488509_GA_G	3	25,488,509	GA	G	0.787	0.956	RARB
rs77784227	3	25,488,617	T	C	0.964	0.977	RARB
rs9822134	3	25,488,997	T	G	0.963	0.981	RARB
3:25489888_GA_G	3	25,489,888	GA	G	0.81	0.947	RARB
rs13316073	3	25,490,039	A	G	0.703	0.971	RARB
rs77052411	3	25,490,193	G	C	0.92	0.981	RARB
rs59052470	3	25,490,258	G	T	0.736	0.967	RARB
rs776690763	3	25,490,893	CCTTAA	C	0.476	0.952	RARB
rs6550978	3	25,490,969	G	A	0.401	0.966	RARB
rs6550979	3	25,490,978	G	A	0.514	0.966	RARB
rs9860017	3	25,491,005	C	T	0.965	0.968	RARB
rs6777544	3	25,491,006	C	G	0.514	0.967	RARB
rs6550980	3	25,491,214	G	C	0.26	0.99	RARB
3:25491247_CT_C	3	25,491,247	CT	C	0.528	0.958	RARB
rs111266002	3	25,491,444	C	T	0.903	0.984	RARB
rs9814190	3	25,492,302	T	C	0.38	0.998	RARB
rs56066644	3	25,492,303	G	A	0.767	0.979	RARB
rs148723030	3	25,492,322	A	G	0.922	0.997	RARB
rs78327786	3	25,492,502	C	T	0.922	0.997	RARB
rs9869928	3	25,492,614	C	G	0.964	0.981	RARB
rs147729254	3	25,493,108	T	A	0.987	0.861	RARB
rs13319140	3	25,493,366	A	T	0.964	0.982	RARB
rs149037743	3	25,493,500	A	G	0.975	0.932	RARB
rs57444137	3	25,493,786	A	G	0.653	0.982	RARB
rs149810231	3	25,493,918	A	AGT	0.427	0.99	RARB
rs9880128	3	25,494,089	G	C	0.352	0.993	RARB
rs796115433	3	25,494,163	GT	G	0.901	0.977	RARB
rs77091013	3	25,494,192	G	A	0.903	0.988	RARB
rs149765580	3	25,494,865	A	G	0.958	0.993	RARB
rs13314219	3	25,495,637	C	A	0.384	0.994	RARB
rs145669256	3	25,495,746	C	T	0.983	0.873	RARB
rs57789086	3	25,496,176	G	GA	0.757	0.94	RARB
rs77273598	3	25,496,386	C	T	0.922	0.992	RARB
rs957950	3	25,496,674	A	G	0.964	0.98	RARB
rs17016431	3	25,497,035	G	T	0.966	0.983	RARB
rs17524838	3	25,497,325	G	T	0.965	0.991	RARB
rs75478711	3	25,497,768	A	G	0.965	0.993	RARB
rs58305592	3	25,498,282	C	T	0.849	0.978	RARB
rs12636934	3	25,498,953	A	G	0.934	0.998	RARB
rs78512285	3	25,498,980	G	A	0.965	0.996	RARB
rs75511149	3	25,499,334	T	C	0.934	0.999	RARB
rs76534935	3	25,499,452	G	A	0.966	0.999	RARB
rs77257361	3	25,499,522	T	A	0.99	0.994	RARB
rs74521791	3	25,499,541	G	A	0.934	0.999	RARB
rs76269161	3	25,500,108	T	C	0.965	0.996	RARB
rs77790606	3	25,500,138	A	G	0.965	0.995	RARB
rs67306275	3	25,500,316	T	C	0.857	0.978	RARB
rs147065635	3	25,500,362	T	TA	0.934	0.999	RARB
rs75893335	3	25,500,418	A	C	0.985	0.99	RARB
rs74670970	3	25,500,729	C	T	0.965	0.989	RARB
rs74472949	3	25,500,732	A	G	0.934	0.998	RARB
rs7629902	3	25,500,881	G	A	0.858	0.979	RARB
rs1864907	3	25,502,318	C	T	0.058	0.94	RARB
rs74820342	3	25,502,496	C	T	0.985	0.99	RARB
rs77748152	3	25,502,851	G	A	0.965	0.991	RARB
rs2067964	3	25,502,869	T	G	0.89	0.996	RARB
rs6764866	3	25,503,235	A	T	0.95	0.991	RARB
rs9824936	3	25,503,325	G	C	0.748	0.985	RARB
rs13077577	3	25,503,679	G	A	0.944	0.989	RARB
rs80108542	3	25,504,564	A	G	0.985	0.978	RARB
rs60107328	3	25,504,657	C	G	0.749	0.978	RARB
rs9830504	3	25,504,662	C	T	0.871	0.962	RARB
rs57446688	3	25,504,666	A	G	0.749	0.978	RARB
rs113845344	3	25,504,948	G	A	0.966	0.982	RARB
rs77338872	3	25,505,013	G	C	0.934	0.985	RARB
rs2053024	3	25,505,827	C	T	0.351	0.971	RARB
rs2053023	3	25,506,141	T	C	0.89	0.974	RARB
rs36085910	3	25,506,240	A	G	0.889	0.973	RARB
rs76303288	3	25,506,242	T	G	0.884	0.977	RARB
rs34761979	3	25,506,414	T	C	0.89	0.972	RARB
rs185421698	3	25,506,440	A	G	0.984	0.885	RARB
rs6778350	3	25,507,030	A	G	0.151	0.932	RARB
rs11929566	3	25,507,303	A	G	0.89	0.965	RARB

SNP	Chromosome	Position	Effect Allele	Other Allele	Effect Allele Frequency	Imputation quality	Gene
rs111340856	3	25,507,321	T	TA	0.848	0.972	RARB
rs76815724	3	25,507,550	A	C	0.967	0.961	RARB
rs6550981	3	25,508,064	G	C	0.477	0.975	RARB
rs75857886	3	25,508,253	G	A	0.892	0.954	RARB
rs146322522	3	25,508,670	A	G	0.987	0.902	RARB
rs541737888	3	25,508,987	A	G	0.989	0.769	RARB
rs756616331	3	25,508,994	TACAATCATAGCAAGCCAACGCCACTTATCCAGTGAACCA	T	0.519	0.886	RARB
rs35796394	3	25,509,109	T	C	0.657	0.896	RARB
rs2033447	3	25,509,351	A	G	0.877	0.886	RARB
rs80013794	3	25,509,809	A	T	0.952	0.88	RARB
3:25510046_TA_T	3	25,510,046	TA	T	0.459	0.858	RARB
rs9872000	3	25,510,755	A	G	0.965	0.944	RARB
rs9839981	3	25,511,786	G	T	0.965	0.997	RARB
rs74614327	3	25,512,767	A	G	0.987	0.89	RARB
rs871963	3	25,512,768	A	T	0.38	0.952	RARB
rs34752259	3	25,513,089	G	GT	0.376	0.944	RARB
rs4607073	3	25,514,276	G	T	0.448	0.979	RARB
rs11715516	3	25,514,982	G	C	0.187	0.986	RARB
rs35460000	3	25,515,004	T	TG	0.186	0.98	RARB
rs6775425	3	25,515,361	C	T	0.281	0.988	RARB
rs533183147	3	25,515,412	G	GAAAAAAAAAA	0.08	0.909	RARB
rs67222658	3	25,515,545	T	C	0.84	0.99	RARB
rs116075240	3	25,516,575	C	T	0.982	0.887	RARB
rs9866679	3	25,516,821	G	T	0.236	0.997	RARB
rs566628095	3	25,516,892	A	AG	0.986	0.931	RARB
rs187849382	3	25,516,894	G	T	0.922	0.959	RARB
rs4681065	3	25,516,922	A	G	0.235	0.998	RARB
rs62237634	3	25,518,030	C	A	0.984	0.953	RARB
rs78818123	3	25,518,058	A	G	0.989	0.901	RARB
rs67036887	3	25,518,472	C	T	0.851	0.983	RARB
rs10212330	3	25,518,833	A	T	0.233	0.98	RARB
rs67634405	3	25,519,032	T	C	0.839	0.97	RARB
rs1864903	3	25,519,583	A	G	0.599	0.935	RARB
rs1991784	3	25,519,638	C	T	0.945	0.901	RARB
rs1529670	3	25,520,054	T	C	0.786	0.962	RARB
rs13072719	3	25,520,302	G	C	0.897	0.969	RARB
rs1368262	3	25,520,565	A	G	0.83	0.98	RARB
rs1529672	3	25,520,582	C	A	0.827	0.989	RARB
3:25520627_TTGTC_T	3	25,520,627	TTGTC	T	0.825	0.981	RARB
rs201692330	3	25,520,646	C	CT	0.941	0.958	RARB
rs73820500	3	25,520,884	A	G	0.942	0.98	RARB
rs76601762	3	25,521,655	G	A	0.941	0.965	RARB
rs2364120	3	25,521,703	G	A	0.098	0.989	RARB
rs4681066	3	25,521,848	T	A	0.652	0.991	RARB
rs6796669	3	25,522,416	T	A	0.443	0.99	RARB
rs1864909	3	25,522,717	C	A	0.946	0.989	RARB
rs1835901	3	25,522,914	T	A	0.398	0.993	RARB
3:25523497_TAAC_T	3	25,523,497	TAAC	T	0.989	0.994	RARB
rs58220846	3	25,524,196	A	G	0.933	0.99	RARB
rs6550982	3	25,524,568	A	G	0.399	0.996	RARB
rs13314209	3	25,525,545	G	A	0.932	0.993	RARB
rs6767056	3	25,525,730	G	C	0.437	0.995	RARB
rs1286656	3	25,526,372	G	T	0.098	0.995	RARB
3:25526611_ATAAAC_A	3	25,526,611	ATAAAC	A	0.984	0.871	RARB
rs56327128	3	25,526,875	G	A	0.945	0.994	RARB
rs13072276	3	25,527,253	G	A	0.435	0.996	RARB
rs113947608	3	25,527,362	G	A	0.988	0.998	RARB
rs13072006	3	25,527,368	A	G	0.435	0.996	RARB
rs56212712	3	25,527,393	A	G	0.858	0.993	RARB
rs1286657	3	25,527,653	C	G	0.674	0.997	RARB
rs1286658	3	25,527,839	T	C	0.109	0.998	RARB
rs1286659	3	25,527,923	A	C	0.109	0.998	RARB
rs1286660	3	25,527,929	C	T	0.109	0.998	RARB
rs1286662	3	25,528,376	A	G	0.816	0.998	RARB
rs6768669	3	25,528,574	T	C	0.43	0.998	RARB
rs1286663	3	25,528,608	T	C	0.11	0.999	RARB
rs1296402	3	25,528,660	C	G	0.11	0.999	RARB
3:25528963_CT_C	3	25,528,963	CT	C	0.094	0.85	RARB
rs1299408	3	25,529,287	G	A	0.73	0.998	RARB
rs7616278	3	25,529,507	A	G	0.429	0.997	RARB
rs34502886	3	25,530,412	T	C	0.436	0.999	RARB
rs73046109	3	25,530,790	G	T	0.913	0.996	RARB
rs11705839	3	25,530,887	C	T	0.437	0.999	RARB
rs146400172	3	25,531,106	T	TTG	0.872	0.733	RARB
rs11714401	3	25,531,110	A	G	0.541	0.888	RARB
rs11706727	3	25,531,111	G	T	0.541	0.888	RARB
rs11714404	3	25,531,117	A	G	0.436	0.999	RARB
rs1286654	3	25,531,265	C	A	0.729	0.998	RARB
rs73046112	3	25,531,374	G	T	0.913	0.996	RARB
rs66875718	3	25,531,386	C	T	0.945	0.998	RARB
rs11706799	3	25,531,452	C	T	0.437	0.999	RARB
rs34566813	3	25,531,601	G	A	0.439	0.998	RARB
rs73046116	3	25,531,605	A	T	0.911	0.994	RARB
rs1286653	3	25,531,673	G	A	0.097	0.997	RARB
rs1299017	3	25,531,884	C	T	0.569	0.997	RARB
rs62237653	3	25,531,906	G	A	0.988	0.996	RARB
rs1299407	3	25,531,924	G	A	0.569	0.997	RARB
rs1286652	3	25,532,025	A	T	0.569	0.997	RARB
rs73822907	3	25,532,112	A	G	0.91	0.996	RARB
3:25532190_AT_A	3	25,532,190	AT	A	0.91	0.985	RARB
rs112275048	3	25,532,210	A	G	0.91	0.996	RARB
rs11916491	3	25,532,425	G	A	0.844	0.995	RARB
rs1286651	3	25,532,503	G	C	0.575	0.996	RARB
rs1286650	3	25,532,657	A	T	0.57	0.997	RARB
3:25533431_CAA_C	3	25,533,431	CAA	C	0.638	0.976	RARB
3:25533457_AT_A	3	25,533,457	AT	A	0.913	0.98	RARB
rs73046130	3	25,533,571	A	G	0.842	0.994	RARB
rs75583414	3	25,533,771	A	T	0.948	0.99	RARB
3:25534159_AT_A	3	25,534,159	AT	A	0.911	0.987	RARB
3:25534179_GT_G	3	25,534,179	GT	G	0.913	0.989	RARB
rs147001666	3	25,534,472	C	T	0.986	0.935	RARB
rs35536382	3	25,534,688	G	T	0.943	0.988	RARB
rs75722923	3	25,535,089	G	C	0.913	0.995	RARB
rs79785747	3	25,535,429	T	G	0.911	0.996	RARB
rs79651109	3	25,535,712	A	C	0.912	0.996	RARB
rs142658742	3	25,535,741	T	TACACACAC	0.705	0.904	RARB
rs5004167	3	25,535,744	G	A	0.703	0.918	RARB
rs3074677	3	25,535,752	A	G	0.832	0.914	RARB
rs2568878	3	25,535,771	T	C	0.73	0.995	RARB
rs4021037	3	25,535,870	G	T	0.834	0.993	RARB
rs1286648	3	25,537,503	T	G	0.519	0.994	RARB
rs1286647	3	25,537,669	G	A	0.817	0.998	RARB
rs1997351	3	25,538,124	G	C	0.521	0.994	RARB
rs2568876	3	25,538,410	T	C	0.725	0.999	RARB
rs2698516	3	25,538,432	G	A	0.565	0.996	RARB
rs149886693	3	25,538,524	C	T	0.908	0.991	RARB
rs112050806	3	25,538,675	C	T	0.912	0.997	RARB
rs2698517	3	25,538,743	G	C	0.564	0.996	RARB
rs34717873	3	25,538,769	T	TCA	0.725	0.999	RARB
rs75165170	3	25,539,115	G	T	0.912	0.997	RARB
rs79054380	3	25,539,265	C	T	0.912	0.996	RARB
rs78277435	3	25,540,264	A	G	0.912	0.994	RARB
rs2056777	3	25,540,391	T	C	0.816	0.996	RARB
rs139677191	3	25,540,818	G	A	0.985	0.943	RARB
rs75277010	3	25,540,912	G	C	0.913	0.993	RARB
rs542334886	3	25,541,147	GTGTT	G	0.57	0.986	RARB
rs1406575	3	25,541,371	A	G	0.656	0.992	RARB
rs1368264	3	25,541,478	A	T	0.568	0.993	RARB
rs1368265	3	25,541,570	A	G	0.568	0.993	RARB
rs546969600	3	25,541,963	T	TGG	0.99	0.666	RARB
rs548928450	3	25,541,964	T	TGG	0.99	0.666	RARB
rs547209912	3	25,541,980	G	T	0.99	0.666	RARB
rs1297795	3	25,541,991	G	A	0.824	0.995	RARB
rs9843863	3	25,542,091	C	T	0.825	0.995	RARB
rs2116703	3	25,542,571	G	A	0.836	0.991	RARB
rs6768285	3	25,543,168	C	T	0.832	0.991	RARB
rs1153582	3	25,543,275	C	T	0.825	0.994	RARB
3:25543313_TC_T	3	25,543,313	TC	T	0.99	0.779	RARB
rs1153583	3	25,543,356	G	A	0.569	0.991	RARB
rs77466283	3	25,543,765	C	T	0.913	0.991	RARB
rs1286641	3	25,543,870	A	T	0.655	0.991	RARB
rs1153584	3	25,544,118	G	A	0.568	0.991	RARB
rs17590510	3	25,544,273	A	T	0.913	0.991	RARB
rs17590545	3	25,544,438	C	A	0.913	0.991	RARB
rs111879033	3	25,544,595	T	TTGGCCACA	0.563	0.987	RARB
rs75222858	3	25,544,666	G	A	0.913	0.99	RARB
rs4446182	3	25,545,393	A	T	0.914	0.988	RARB
rs4260361	3	25,545,468	A	G	0.914	0.989	RARB
rs1303630	3	25,545,500	A	C	0.527	0.981	RARB
rs4470477	3	25,545,507	G	A	0.938	0.852	RARB
rs4527339	3	25,545,512	T	G	0.938	0.852	RARB
rs4530495	3	25,545,537	C	T	0.915	0.978	RARB
rs17016566	3	25,546,219	C	G	0.932	0.992	RARB
rs112385750	3	25,546,310	G	A	0.914	0.988	RARB
rs144551189	3	25,546,336	C	T	0.986	0.926	RARB

SNP	Chromosome	Position	Effect Allele	Other Allele	Effect Allele Frequency	Imputation quality	Gene
rs1298216	3	25,546,376	G	A	0.655	0.99	RARB
rs1286642	3	25,546,536	C	T	0.821	0.992	RARB
rs5847359	3	25,547,035	A	AT	0.581	0.963	RARB
rs76134077	3	25,547,209	G	A	0.914	0.986	RARB
rs75074271	3	25,547,612	C	T	0.915	0.984	RARB
rs112801134	3	25,547,660	C	G	0.915	0.985	RARB
rs1286643	3	25,547,688	A	G	0.601	0.988	RARB
rs1286644	3	25,548,205	A	G	0.551	0.989	RARB
rs1560633	3	25,548,555	T	C	0.362	0.984	RARB
rs1286645	3	25,548,574	G	A	0.818	0.993	RARB
rs79383693	3	25,548,591	C	A	0.939	0.975	RARB
rs1286646	3	25,548,731	G	A	0.103	0.998	RARB
3:25548798_CT_C	3	25,548,798	CT	C	0.435	0.989	RARB
rs1997353	3	25,548,962	C	T	0.435	0.993	RARB
rs73048024	3	25,549,250	G	A	0.942	0.994	RARB
rs1153586	3	25,549,389	T	C	0.101	0.998	RARB
rs62235660	3	25,550,240	C	A	0.432	0.993	RARB
rs1153587	3	25,550,359	A	G	0.813	0.994	RARB
rs11369682	3	25,550,385	T	TA	0.811	0.966	RARB
rs6767543	3	25,550,423	G	A	0.3	0.992	RARB
rs1881706	3	25,550,452	A	G	0.3	0.993	RARB
rs1153588	3	25,550,609	C	G	0.101	0.992	RARB
rs115286324	3	25,550,662	G	A	0.985	0.974	RARB
rs1881705	3	25,550,743	C	T	0.375	0.993	RARB
rs17016584	3	25,551,807	C	G	0.931	0.993	RARB
rs111515348	3	25,551,922	C	T	0.977	0.931	RARB
rs111529831	3	25,552,059	T	G	0.913	0.995	RARB
rs796833346	3	25,552,116	CA	C	0.944	0.977	RARB
rs11926758	3	25,552,252	G	T	0.924	0.996	RARB
rs62235661	3	25,552,656	C	T	0.988	0.995	RARB
rs1286749	3	25,552,836	A	G	0.867	0.997	RARB
rs112966165	3	25,552,896	A	G	0.924	0.997	RARB
rs1286751	3	25,553,310	A	G	0.656	0.999	RARB
rs1286752	3	25,553,617	C	T	0.787	0.997	RARB
rs58312942	3	25,553,634	A	C	0.988	0.996	RARB
rs138588118	3	25,553,952	T	TGTTA	0.923	0.997	RARB
rs1153589	3	25,554,363	G	A	0.778	0.998	RARB
rs10446417	3	25,554,733	G	A	0.922	0.995	RARB
rs1303629	3	25,555,106	T	G	0.777	0.998	RARB
3:25555240_CG_C	3	25,555,240	CG	C	0.982	0.961	RARB
rs17590924	3	25,555,410	A	G	0.923	0.999	RARB
rs7645775	3	25,556,566	G	C	0.923	0.999	RARB
rs79537404	3	25,556,932	A	T	0.923	1	RARB
rs6781550	3	25,557,486	C	A	0.922	0.999	RARB
rs1153591	3	25,557,516	G	A	0.872	0.998	RARB
rs17591006	3	25,557,684	A	G	0.923	1	RARB
rs58929393	3	25,558,172	C	T	0.94	0.992	RARB
rs57195207	3	25,558,240	C	A	0.939	0.998	RARB
rs80051246	3	25,558,438	A	G	0.988	0.998	RARB
rs115056838	3	25,559,516	A	G	0.984	0.901	RARB
rs1153594	3	25,559,928	C	T	0.876	0.998	RARB
rs34821999	3	25,559,944	C	A	0.926	0.993	RARB
3:25559985_GTAGT_G	3	25,559,985	GTAGT	G	0.939	0.999	RARB
rs1153595	3	25,560,037	C	G	0.799	0.999	RARB
rs1435703	3	25,560,231	G	T	0.939	0.999	RARB
rs62235692	3	25,560,591	C	G	0.927	0.998	RARB
rs113013298	3	25,560,773	A	T	0.939	0.999	RARB
rs4021038	3	25,560,806	C	CAT	0.926	0.996	RARB
rs1347458	3	25,561,049	A	G	0.926	0.997	RARB
rs62235693	3	25,561,339	G	A	0.988	0.996	RARB
rs1153596	3	25,561,488	A	G	0.855	0.997	RARB
rs7644940	3	25,561,669	G	A	0.939	0.998	RARB
rs7626008	3	25,562,070	C	T	0.939	0.998	RARB
rs77898348	3	25,562,108	A	G	0.939	0.998	RARB
rs7647611	3	25,562,211	G	A	0.939	0.998	RARB
rs182594225	3	25,562,445	T	C	0.989	0.887	RARB
rs1435704	3	25,562,516	C	G	0.939	0.998	RARB
rs201750844	3	25,563,081	A	AAT	0.941	0.988	RARB
3:25563495_AC_A	3	25,563,495	AC	A	0.939	0.993	RARB
rs62235694	3	25,563,841	G	A	0.988	0.993	RARB
rs17525635	3	25,564,163	T	A	0.988	0.993	RARB
rs1286753	3	25,564,603	G	A	0.891	0.991	RARB
rs6787891	3	25,564,898	C	T	0.938	0.999	RARB
rs6805350	3	25,564,929	T	G	0.938	0.999	RARB
rs17591410	3	25,565,576	A	C	0.923	0.998	RARB
3:25565731_TC_T	3	25,565,731	TC	T	0.939	0.996	RARB
rs541603677	3	25,565,733	C	A	0.949	0.918	RARB
rs561820664	3	25,565,734	A	G	0.949	0.918	RARB
rs386659485	3	25,565,735	G	GA	0.94	0.989	RARB
rs530480170	3	25,565,735	G	A	0.95	0.918	RARB
rs7615754	3	25,566,046	G	A	0.94	0.99	RARB
rs78926911	3	25,566,074	G	T	0.943	0.979	RARB
3:25566101_CT_C	3	25,566,101	CT	C	0.94	0.986	RARB
rs1286754	3	25,566,195	C	T	0.579	0.996	RARB
rs113704042	3	25,566,313	T	C	0.939	0.997	RARB
rs1286755	3	25,566,368	T	G	0.579	0.997	RARB
rs112775192	3	25,566,430	G	A	0.939	0.997	RARB
rs77618484	3	25,566,526	A	G	0.939	0.996	RARB
rs62235696	3	25,567,009	A	G	0.899	0.993	RARB
rs148567765	3	25,567,044	G	GAC	0.867	0.996	RARB
rs111591203	3	25,567,241	T	TAAATAA	0.902	0.993	RARB
rs55637130	3	25,567,352	A	G	0.902	0.996	RARB
rs1286757	3	25,567,812	C	T	0.577	0.998	RARB
rs77692995	3	25,567,817	G	A	0.946	0.997	RARB
rs1286758	3	25,567,938	T	C	0.865	0.998	RARB
rs1286759	3	25,567,971	T	C	0.577	0.998	RARB
rs76397643	3	25,567,979	T	C	0.946	0.997	RARB
rs13099641	3	25,568,512	T	A	0.871	0.998	RARB
rs1435705	3	25,568,871	G	A	0.903	0.997	RARB
rs1153598	3	25,569,043	A	G	0.866	0.999	RARB
rs1153600	3	25,569,565	C	T	0.678	0.999	RARB
rs1153601	3	25,569,761	A	C	0.663	0.998	RARB
rs17525900	3	25,569,881	T	C	0.884	0.993	RARB
rs202112286	3	25,570,329	C	CATTCTTTGTAAAACGA	0.946	0.995	RARB
3:25570352_AG_A	3	25,570,352	AG	A	0.795	0.992	RARB
rs1299018	3	25,570,554	C	T	0.665	0.997	RARB
rs3074713	3	25,570,892	G	GATTCATC	0.959	0.993	RARB
rs1153602	3	25,571,152	T	A	0.678	0.997	RARB
rs1153603	3	25,571,725	C	T	0.682	0.996	RARB
rs11714409	3	25,571,939	A	G	0.966	0.966	RARB
rs76337654	3	25,571,955	C	T	0.945	0.994	RARB
3:25572879_GTAGATAGATAGA_G	3	25,572,879	GTAGATAGATAGA	G	0.697	0.986	RARB
rs1286760	3	25,573,009	C	T	0.832	0.995	RARB
rs1153604	3	25,573,327	G	A	0.656	0.994	RARB
rs1153605	3	25,573,551	C	T	0.832	0.996	RARB
rs113313452	3	25,573,813	G	T	0.946	0.992	RARB
rs73048048	3	25,574,034	T	A	0.959	0.996	RARB
rs17526019	3	25,574,850	C	T	0.929	0.991	RARB
rs1286665	3	25,575,675	C	T	0.719	0.99	RARB
rs1286666	3	25,575,829	T	C	0.718	0.99	RARB
rs1286667	3	25,576,312	T	C	0.718	0.989	RARB
rs1992005	3	25,576,865	G	A	0.919	0.986	RARB
3:25577882_AG_A	3	25,577,882	AG	A	0.919	0.98	RARB
rs1881703	3	25,578,552	A	C	0.506	0.98	RARB
rs7616062	3	25,578,892	C	T	0.788	0.984	RARB
rs7631979	3	25,578,938	T	C	0.632	0.975	RARB
rs138887498	3	25,579,112	T	G	0.988	0.921	RARB
rs1286773	3	25,579,202	C	G	0.874	0.986	RARB
rs1656452	3	25,579,777	C	T	0.788	0.985	RARB
rs11707637	3	25,579,915	A	G	0.548	0.979	RARB
rs114958234	3	25,579,952	G	A	0.987	0.979	RARB
rs1286772	3	25,580,776	C	G	0.405	0.977	RARB
rs750401243	3	25,581,023	C	CTT	0.762	0.79	RARB
rs34726255	3	25,581,072	C	CT	0.51	0.916	RARB
rs13070526	3	25,581,088	T	C	0.739	0.976	RARB
3:25581312_AT_A	3	25,581,312	AT	A	0.901	0.978	RARB
rs1286771	3	25,581,512	T	C	0.873	0.988	RARB
rs145718609	3	25,581,681	C	T	0.985	0.887	RARB
rs1286770	3	25,581,798	C	T	0.874	0.989	RARB
3:25581866_CTT_C	3	25,581,866	CTT	C	0.874	0.977	RARB
rs111406124	3	25,582,387	G	GTCTGATTTAC	0.531	0.965	RARB
3:25582450_GA_G	3	25,582,450	GA	G	0.567	0.977	RARB
rs1865613	3	25,582,594	A	G	0.943	0.99	RARB
3:25584407_ATAGT_A	3	25,584,407	ATAGT	A	0.943	0.988	RARB
rs12638229	3	25,584,834	T	A	0.861	0.991	RARB
rs111229109	3	25,585,103	T	TG	0.958	0.997	RARB
rs1286769	3	25,585,166	G	A	0.56	0.987	RARB
rs17016718	3	25,586,106	T	C	0.834	0.984	RARB
rs7623826	3	25,586,165	C	G	0.905	0.972	RARB
rs7645308	3	25,586,166	G	C	0.905	0.972	RARB
rs7635240	3	25,586,207	A	G	0.903	0.98	RARB
rs115634233	3	25,586,210	C	T	0.971	0.954	RARB
rs7635247	3	25,586,212	A	T	0.903	0.98	RARB
rs1286768	3	25,586,255	A	C	0.499	0.987	RARB
rs7638066	3	25,586,638	T	G	0.838	0.988	RARB

SNP	Chromosome	Position	Effect Allele	Other Allele	Effect Allele Frequency	Imputation quality	Gene
rs75264900	3	25,586,733	G	T	0.935	0.992	RARB
rs61286411	3	25,586,839	T	C	0.838	0.988	RARB
rs76186856	3	25,586,875	A	G	0.935	0.991	RARB
rs78192825	3	25,587,324	C	G	0.935	0.992	RARB
rs73820517	3	25,587,363	T	C	0.935	0.992	RARB
rs77703228	3	25,587,466	T	A	0.935	0.992	RARB
rs114933439	3	25,587,644	A	G	0.963	0.981	RARB
rs77285114	3	25,587,751	C	T	0.935	0.992	RARB
rs74502621	3	25,589,037	A	G	0.935	0.992	RARB
rs1435706	3	25,589,196	A	T	0.726	0.99	RARB
rs74786879	3	25,589,215	A	G	0.935	0.992	RARB
3:25589592_CTT_C	3	25,589,592	CTT	C	0.713	0.979	RARB
rs75205624	3	25,590,878	A	G	0.935	0.993	RARB
3:25591573_ATACTGTCTTATGG_A	3	25,591,573	ATACTGTCTTATGG	A	0.936	0.983	RARB
rs76194157	3	25,592,103	T	C	0.935	0.993	RARB
rs78455190	3	25,592,864	G	C	0.935	0.994	RARB
rs1626875	3	25,593,127	G	A	0.825	0.998	RARB
rs78967133	3	25,593,161	A	G	0.935	0.994	RARB
rs1286766	3	25,593,651	A	T	0.506	0.994	RARB
rs1286765	3	25,593,872	G	A	0.506	0.994	RARB
rs77233893	3	25,593,907	C	A	0.935	0.994	RARB
3:25593929_TG_T	3	25,593,929	TG	T	0.94	0.959	RARB
rs7609566	3	25,594,185	G	C	0.935	0.994	RARB
rs5847364	3	25,594,236	A	AATACTT	0.508	0.988	RARB
3:25594645_CT_C	3	25,594,645	CT	C	0.56	0.967	RARB
rs76906394	3	25,594,988	G	C	0.935	0.995	RARB
rs79988241	3	25,595,140	C	G	0.986	0.986	RARB
rs76378902	3	25,595,616	G	A	0.935	0.995	RARB
rs73048068	3	25,595,631	G	A	0.796	0.995	RARB
rs80206941	3	25,596,152	A	G	0.935	0.996	RARB
rs74862209	3	25,596,524	A	T	0.935	0.996	RARB
rs1561032	3	25,596,613	A	C	0.739	0.996	RARB
rs77682964	3	25,597,480	G	A	0.919	0.998	RARB
rs35199932	3	25,597,498	A	AT	0.564	0.953	RARB
rs78550724	3	25,597,616	T	G	0.919	0.998	RARB
rs140817964	3	25,597,932	C	T	0.972	0.987	RARB
rs1286762	3	25,598,909	G	A	0.83	0.998	RARB
rs1286761	3	25,599,575	G	C	0.81	0.996	RARB
rs141655475	3	25,599,929	C	G	0.988	0.844	RARB
3:25600020_CTTT_C	3	25,600,020	CTTT	C	0.509	0.989	RARB
rs17016738	3	25,600,646	C	T	0.742	0.996	RARB
rs17592183	3	25,600,761	A	G	0.927	0.993	RARB
rs34214973	3	25,600,836	A	G	0.739	0.992	RARB
rs33986016	3	25,600,838	G	T	0.74	0.991	RARB
rs147703165	3	25,601,292	G	A	0.986	0.993	RARB
rs1290443	3	25,601,835	T	C	0.833	0.999	RARB
rs78488610	3	25,602,017	G	A	0.925	0.919	RARB
rs7610827	3	25,602,648	T	A	0.688	0.994	RARB
rs7610831	3	25,602,662	T	C	0.672	0.993	RARB
rs10510568	3	25,602,732	G	C	0.687	0.995	RARB
rs4681067	3	25,602,890	T	C	0.829	0.998	RARB
rs17526721	3	25,603,319	A	G	0.919	0.993	RARB
rs17029657	3	25,603,432	T	G	0.734	0.996	RARB
3:25603487_TCCCTGCTCAACTTTTTTA_T	3	25,603,487	TCCCTGCTCAACTTTTTTA	T	0.94	0.964	RARB
rs113170207	3	25,603,726	CT	C	0.758	0.984	RARB
rs34359044	3	25,603,733	T	C	0.759	0.997	RARB
rs1297817	3	25,603,749	G	A	0.523	0.994	RARB
rs17016773	3	25,604,599	C	T	0.8	0.999	RARB
rs17526839	3	25,605,101	A	C	0.919	0.988	RARB
rs17016778	3	25,605,282	A	G	0.766	0.989	RARB
3:25605438_CT_C	3	25,605,438	CT	C	0.677	0.911	RARB
rs76931802	3	25,605,965	G	A	0.938	0.944	RARB
rs137910801	3	25,606,785	T	C	0.985	0.955	RARB
rs1730240	3	25,607,513	A	C	0.576	0.981	RARB
rs182761355	3	25,608,205	C	T	0.99	0.899	RARB
rs1286729	3	25,608,576	C	T	0.881	0.989	RARB
rs77132200	3	25,608,843	T	G	0.926	0.986	RARB
3:25608878_AT_A	3	25,608,878	AT	A	0.97	0.986	RARB
rs6795340	3	25,609,011	G	A	0.772	0.987	RARB
rs138970809	3	25,609,020	T	G	0.96	0.884	RARB
rs73050040	3	25,609,170	C	T	0.868	0.985	RARB
rs1286730	3	25,610,379	C	G	0.866	0.991	RARB
rs766802500	3	25,610,481	GA	G	0.978	0.996	RARB
rs1286731	3	25,610,968	T	C	0.895	0.99	RARB
rs1656455	3	25,611,626	C	T	0.895	0.99	RARB
rs111473988	3	25,612,397	G	A	0.988	0.932	RARB
3:25612435_GA_G	3	25,612,435	GA	G	0.705	0.967	RARB
rs3773445	3	25,612,565	A	G	0.566	0.983	RARB
rs1296109	3	25,612,586	A	C	0.583	0.985	RARB
rs75390270	3	25,612,785	G	A	0.933	0.992	RARB
rs1294660	3	25,612,981	C	T	0.878	0.992	RARB
rs1298215	3	25,613,223	C	T	0.811	0.996	RARB
rs1286733	3	25,613,372	T	C	0.62	0.987	RARB
rs59175453	3	25,613,619	G	A	0.97	1	RARB
rs1286734	3	25,613,889	A	C	0.879	0.995	RARB
rs1529875	3	25,613,896	T	C	0.806	0.983	RARB
rs1286735	3	25,613,901	T	A	0.588	0.989	RARB
rs62235700	3	25,614,126	G	A	0.988	0.922	RARB
rs12635379	3	25,614,448	G	A	0.809	0.984	RARB
rs80114424	3	25,614,514	T	G	0.933	0.994	RARB
rs1286738	3	25,614,532	C	T	0.782	0.999	RARB
rs1286739	3	25,614,607	G	A	0.895	0.995	RARB
rs80307993	3	25,614,910	A	C	0.933	0.993	RARB
rs55684655	3	25,614,943	G	A	0.821	0.985	RARB
rs74341355	3	25,614,997	G	C	0.933	0.993	RARB
rs1286740	3	25,615,163	G	C	0.633	0.989	RARB
rs188565780	3	25,615,274	T	A	0.933	0.988	RARB
rs191888046	3	25,615,275	A	G	0.933	0.988	RARB
rs1286741	3	25,615,642	A	G	0.88	0.995	RARB
rs12636182	3	25,615,777	C	T	0.864	0.994	RARB
rs1881704	3	25,616,109	G	C	0.882	0.995	RARB
rs55801354	3	25,616,252	G	A	0.866	0.995	RARB
rs75767585	3	25,616,629	A	G	0.933	0.992	RARB
rs17526942	3	25,617,403	C	T	0.883	0.995	RARB
rs3773439	3	25,617,775	T	C	0.648	0.999	RARB
rs1656463	3	25,618,159	G	A	0.816	0.999	RARB
rs77582389	3	25,618,177	T	A	0.933	0.994	RARB
rs61629289	3	25,618,183	C	A	0.837	0.999	RARB
rs3821634	3	25,618,230	G	A	0.837	0.999	RARB
rs1730218	3	25,618,251	C	G	0.653	0.999	RARB
3:25618266_AC_A	3	25,618,266	AC	A	0.817	0.996	RARB
rs66482848	3	25,618,347	G	A	0.653	1	RARB
rs1730220	3	25,618,368	C	G	0.651	1	RARB
rs1730222	3	25,618,692	G	A	0.653	0.999	RARB
rs1656465	3	25,618,700	C	T	0.884	0.996	RARB
rs1730223	3	25,618,814	G	A	0.653	0.999	RARB
3:25619359_CAT_C	3	25,619,359	CAT	C	0.653	0.996	RARB
rs111982812	3	25,619,399	A	G	0.933	0.992	RARB
rs6803265	3	25,619,414	T	A	0.834	0.997	RARB
rs1968481	3	25,619,531	A	G	0.933	0.991	RARB
rs1656467	3	25,619,556	C	T	0.816	0.996	RARB
rs1529876	3	25,619,630	G	C	0.816	0.996	RARB
rs141814538	3	25,619,892	G	A	0.985	0.865	RARB
rs1631354	3	25,619,920	G	C	0.606	0.988	RARB
rs1631395	3	25,619,935	G	A	0.816	0.993	RARB
rs3821629	3	25,620,132	T	C	0.789	0.983	RARB
rs75201484	3	25,620,207	C	T	0.933	0.988	RARB
rs1730226	3	25,620,233	T	A	0.579	0.986	RARB
rs1730230	3	25,620,485	T	G	0.883	0.989	RARB
rs1730231	3	25,620,717	T	A	0.575	0.983	RARB
rs2364121	3	25,620,861	C	G	0.791	0.98	RARB
rs73151333	3	25,621,135	C	G	0.97	0.988	RARB
rs7634756	3	25,621,762	A	G	0.973	0.996	RARB
rs41285069	3	25,622,447	G	T	0.935	0.98	RARB
rs77046429	3	25,623,090	T	C	0.901	0.971	RARB
rs2886124	3	25,623,254	C	G	0.861	0.977	RARB
rs11917304	3	25,624,634	T	C	0.873	0.997	RARB
rs192241122	3	25,624,922	T	C	0.981	0.981	RARB
rs3773432	3	25,625,053	G	A	0.891	0.964	RARB
rs3773431	3	25,625,058	C	T	0.972	0.999	RARB
rs74859600	3	25,625,776	G	C	0.911	0.988	RARB
rs9809535	3	25,625,821	G	A	0.882	0.987	RARB
rs7638736	3	25,626,441	C	G	0.918	0.893	RARB
rs7652621	3	25,626,444	T	C	0.893	0.922	RARB
rs140974385	3	25,627,540	G	A	0.975	0.989	RARB
rs58992195	3	25,627,653	G	C	0.919	0.99	RARB
rs79443185	3	25,628,435	G	A	0.946	0.842	RARB
rs4681028	3	25,628,749	G	T	0.791	0.979	RARB
rs6765189	3	25,628,769	A	G	0.975	0.987	RARB
rs10661318	3	25,628,873	A	ACT	0.975	0.984	RARB
rs113692336	3	25,628,879	T	G	0.975	0.989	RARB
rs4681029	3	25,628,889	A	G	0.801	0.985	RARB
3:25629460_CAATAAATATTCATTA_C	3	25,629,460	CAATAAATATTCATTA	C	0.854	0.976	RARB
rs7620852	3	25,630,531	T	C	0.816	0.984	RARB
rs7621140	3	25,630,857	T	C	0.879	0.987	RARB

SNP	Chromosome	Position	Effect Allele	Other Allele	Effect Allele Frequency	Imputation quality	Gene
rs60997895	3	25,632,023	G	T	0.799	0.986	RARB
rs60609026	3	25,632,113	A	C	0.793	0.987	RARB
rs56254120	3	25,632,253	T	C	0.793	0.989	RARB
rs2164360	3	25,632,894	C	T	0.795	0.99	RARB
rs115603493	3	25,634,844	A	C	0.986	0.975	RARB
rs2278480	3	25,635,743	T	C	0.712	0.987	RARB
rs3773427	3	25,636,344	A	C	0.821	0.986	RARB
rs567400066	3	25,638,860	T	TAA	0.197	0.548	RARB
rs146684489	3	25,638,928	A	ATGAT	0.978	0.932	RARB
rs371314595	3	25,639,130	C	CAACT	0.789	0.967	RARB
rs3832215	3	25,639,141	G	GAAAC	0.728	0.982	RARB
rs10638799	3	25,639,266	A	AT	0.668	0.961	RARB
rs1058378	3	25,639,394	T	G	0.904	0.996	RARB
3:119540178_ATT_A	3	119,540,178	ATT	A	0.83	0.995	GSK3B
rs747442101	3	119,540,977	T	TAT	0.392	0.813	GSK3B
rs60393216	3	119,542,124	A	T	0.831	0.998	GSK3B
rs56728675	3	119,542,190	G	A	0.831	0.998	GSK3B
rs3732361	3	119,542,297	A	G	0.376	0.998	GSK3B
rs55644728	3	119,542,716	C	T	0.875	0.998	GSK3B
3:119543007_CTT_C	3	119,543,007	CTT	C	0.988	0.967	GSK3B
3:119543176_GCACA_G	3	119,543,176	GCACA	G	0.212	0.99	GSK3B
rs55910624	3	119,543,921	T	C	0.875	0.998	GSK3B
rs73175828	3	119,544,144	G	T	0.875	0.998	GSK3B
rs2037547	3	119,544,615	T	C	0.207	0.998	GSK3B
rs72548718	3	119,544,883	T	C	0.978	0.959	GSK3B
rs68194015	3	119,545,152	C	T	0.875	0.996	GSK3B
rs4261913	3	119,546,197	C	T	0.377	0.998	GSK3B
rs56254723	3	119,546,455	A	AT	0.096	0.978	GSK3B
rs4552394	3	119,546,483	G	A	0.969	0.716	GSK3B
rs746118327	3	119,546,716	C	CA	0.717	0.97	GSK3B
rs2873950	3	119,547,062	A	C	0.831	0.998	GSK3B
rs77666560	3	119,550,770	G	T	0.87	0.999	GSK3B
rs9826659	3	119,551,111	A	G	0.682	0.982	GSK3B
rs73175830	3	119,552,319	A	C	0.87	0.999	GSK3B
rs76776670	3	119,554,514	C	T	0.97	0.982	GSK3B
rs78238396	3	119,554,530	T	A	0.977	0.979	GSK3B
rs114873194	3	119,555,777	T	C	0.975	0.981	GSK3B
rs12494560	3	119,556,090	C	T	0.213	0.999	GSK3B
3:119556168_AT_A	3	119,556,168	AT	A	0.19	0.954	GSK3B
rs150016807	3	119,556,203	G	A	0.87	0.999	GSK3B
rs9784393	3	119,556,346	T	G	0.081	0.999	GSK3B
rs142371272	3	119,556,921	G	A	0.989	0.984	GSK3B
rs1546753	3	119,557,075	A	C	0.381	1	GSK3B
rs6808874	3	119,557,851	A	T	0.831	0.999	GSK3B
3:119559071_AT_A	3	119,559,071	AT	A	0.248	0.89	GSK3B
rs144827132	3	119,559,285	C	T	0.99	0.978	GSK3B
rs6805251	3	119,560,606	T	C	0.381	1	GSK3B
rs62264706	3	119,560,856	G	T	0.831	0.999	GSK3B
rs76263256	3	119,560,990	C	T	0.985	0.905	GSK3B
rs57939147	3	119,561,395	T	A	0.831	0.999	GSK3B
rs6790751	3	119,561,600	T	C	0.212	0.999	GSK3B
rs4605584	3	119,562,794	C	A	0.081	0.998	GSK3B
rs148828914	3	119,563,213	T	TA	0.219	0.973	GSK3B
rs151223694	3	119,563,438	A	G	0.98	0.98	GSK3B
rs62264708	3	119,565,857	C	T	0.831	0.999	GSK3B
rs149989740	3	119,565,985	G	C	0.966	0.947	GSK3B
3:119566160_TA_T	3	119,566,160	TA	T	0.871	0.996	GSK3B
rs3107669	3	119,567,101	C	A	0.392	0.999	GSK3B
3:119567267_CT_C	3	119,567,267	CT	C	0.87	0.994	GSK3B
rs12633127	3	119,567,553	T	G	0.985	0.966	GSK3B
rs10934500	3	119,568,123	T	C	0.831	0.999	GSK3B
rs564850407	3	119,568,897	GATGTATGT	G	0.381	1	GSK3B
rs16830567	3	119,570,175	T	C	0.831	0.999	GSK3B
rs60440577	3	119,571,279	T	A	0.986	0.991	GSK3B
rs4624596	3	119,571,541	T	C	0.831	0.999	GSK3B
rs7641045	3	119,571,859	G	C	0.392	0.999	GSK3B
rs145720524	3	119,572,258	T	C	0.989	0.984	GSK3B
rs139350186	3	119,572,441	C	T	0.83	0.999	GSK3B
rs4450859	3	119,573,681	C	T	0.212	0.999	GSK3B
rs9990259	3	119,574,790	G	A	0.946	0.992	GSK3B
3:119575615_TGTCCTA_T	3	119,575,615	TGTCCTA	T	0.979	0.982	GSK3B
rs141048190	3	119,575,962	C	T	0.986	0.967	GSK3B
rs1626696	3	119,576,769	C	A	0.081	0.998	GSK3B
rs146888713	3	119,576,813	C	CACTT	0.965	0.967	GSK3B
rs61623681	3	119,577,788	T	TA	0.081	0.996	GSK3B
rs1719891	3	119,577,862	G	A	0.381	0.997	GSK3B
rs1719890	3	119,577,868	A	G	0.381	0.998	GSK3B
rs11914561	3	119,577,871	A	G	0.87	0.999	GSK3B
rs3031797	3	119,578,031	T	TA	0.247	0.93	GSK3B
rs1719889	3	119,578,788	A	T	0.212	0.999	GSK3B
rs1719888	3	119,578,880	C	T	0.081	0.999	GSK3B
rs11927667	3	119,579,379	T	C	0.87	1	GSK3B
rs11923782	3	119,579,816	C	G	0.87	1	GSK3B
rs62264709	3	119,580,110	C	T	0.977	0.988	GSK3B
rs146857994	3	119,580,244	G	A	0.989	0.983	GSK3B
rs6800622	3	119,580,678	A	C	0.381	1	GSK3B
rs6773751	3	119,581,086	T	C	0.83	0.993	GSK3B
rs1969412	3	119,581,302	T	C	0.081	0.997	GSK3B
rs60389731	3	119,581,345	C	T	0.831	0.999	GSK3B
rs376995408	3	119,581,371	T	TA	0.527	0.924	GSK3B
rs1732170	3	119,583,676	T	C	0.381	1	GSK3B
rs187489241	3	119,584,338	A	G	0.87	0.999	GSK3B
rs1719887	3	119,584,627	A	T	0.082	0.998	GSK3B
rs7633279	3	119,585,690	T	A	0.831	0.999	GSK3B
rs6808975	3	119,588,051	A	C	0.381	1	GSK3B
rs544175713	3	119,588,070	C	CAAAAAA	0.093	0.98	GSK3B
rs79095971	3	119,588,936	T	C	0.985	0.978	GSK3B
rs1719894	3	119,590,322	A	G	0.381	1	GSK3B
rs9829763	3	119,590,788	T	C	0.211	0.999	GSK3B
rs4688042	3	119,590,880	A	G	0.831	0.999	GSK3B
rs9813864	3	119,590,974	C	T	0.381	1	GSK3B
rs141552834	3	119,591,235	C	G	0.989	0.98	GSK3B
rs4688043	3	119,591,248	A	T	0.081	0.999	GSK3B
rs73175844	3	119,592,221	T	C	0.87	1	GSK3B
rs115230267	3	119,593,064	T	G	0.989	0.957	GSK3B
rs770673501	3	119,593,541	AC	A	0.382	0.998	GSK3B
rs180911031	3	119,593,871	G	A	0.982	0.961	GSK3B
rs3108749	3	119,594,488	A	G	0.082	0.998	GSK3B
rs45567135	3	119,595,051	A	G	0.967	0.99	GSK3B
rs1719895	3	119,595,503	A	G	0.081	0.998	GSK3B
rs12634889	3	119,596,514	A	G	0.831	0.999	GSK3B
rs55658250	3	119,597,558	T	C	0.87	1	GSK3B
rs145579239	3	119,598,101	C	T	0.986	0.943	GSK3B
rs2461827	3	119,598,280	A	G	0.211	0.999	GSK3B
rs62264713	3	119,598,409	A	T	0.88	0.996	GSK3B
rs4491945	3	119,598,725	A	G	0.381	1	GSK3B
rs4438691	3	119,598,790	C	T	0.081	0.998	GSK3B
rs148381189	3	119,598,892	C	T	0.987	0.972	GSK3B
rs551427351	3	119,601,002	G	GT	0.87	0.987	GSK3B
rs78288325	3	119,601,353	T	G	0.979	0.981	GSK3B
3:119602292_TAAAA_T	3	119,602,292	TAAAA	T	0.87	0.999	GSK3B
rs146524028	3	119,603,534	T	TA	0.831	0.999	GSK3B
3:119604095_CAAATA_C	3	119,604,095	CAAATA	C	0.83	0.997	GSK3B
rs115221101	3	119,604,146	G	A	0.975	0.997	GSK3B
rs62264714	3	119,604,732	T	C	0.831	0.999	GSK3B
rs1732169	3	119,604,935	G	A	0.082	0.998	GSK3B
rs6765004	3	119,605,243	C	T	0.87	1	GSK3B
rs16830594	3	119,606,078	A	G	0.879	0.996	GSK3B
rs73175849	3	119,606,829	G	A	0.87	1	GSK3B
3:119607323_CTTTTT_C	3	119,607,323	CTTTTT	C	0.081	0.997	GSK3B
rs2319206	3	119,607,327	T	A	0.387	0.851	GSK3B
rs183511131	3	119,607,328	T	C	0.085	0.982	GSK3B
rs1732165	3	119,607,605	A	G	0.082	0.998	GSK3B
rs1732168	3	119,608,141	C	T	0.381	0.998	GSK3B
rs111703820	3	119,608,142	G	A	0.87	1	GSK3B
rs7624540	3	119,608,947	C	A	0.789	0.999	GSK3B
rs13320980	3	119,609,160	T	C	0.679	0.991	GSK3B
rs138229381	3	119,609,426	T	G	0.976	0.941	GSK3B
rs376636354	3	119,609,591	TA	T	0.699	0.915	GSK3B
rs7639388	3	119,609,953	T	A	0.831	0.999	GSK3B
rs78327070	3	119,610,375	G	A	0.922	0.995	GSK3B
rs562496798	3	119,610,454	A	AC	0.987	0.948	GSK3B
rs6795653	3	119,610,565	T	C	0.619	0.999	GSK3B
rs6782799	3	119,610,793	C	T	0.619	0.999	GSK3B
rs11921360	3	119,612,055	C	A	0.368	0.986	GSK3B
rs2319398	3	119,612,942	C	A	0.619	0.999	GSK3B
3:119613452_CTTT_C	3	119,613,452	CTTT	C	0.87	0.999	GSK3B
3:119613634_TTG_T	3	119,613,634	TTG	T	0.977	0.998	GSK3B
rs56076631	3	119,613,781	A	C	0.978	0.956	GSK3B
rs75609636	3	119,614,145	A	G	0.919	0.998	GSK3B
rs201771958	3	119,614,279	AT	A	0.619	0.999	GSK3B
rs13321783	3	119,615,375	T	C	0.608	0.998	GSK3B
rs753834633	3	119,615,394	ACAT	A	0.871	0.998	GSK3B
rs73175853	3	119,615,795	G	T	0.87	1	GSK3B
rs147166874	3	119,616,544	T	G	0.986	0.991	GSK3B

SNP	Chromosome	Position	Effect Allele	Other Allele	Effect Allele Frequency	Imputation quality	Gene
rs73175854	3	119,616,555	C	G	0.981	0.945	GSK3B
rs74469930	3	119,617,971	G	T	0.919	0.999	GSK3B
rs12629015	3	119,618,053	A	G	0.831	0.999	GSK3B
rs7617372	3	119,619,330	A	G	0.788	0.999	GSK3B
rs75385613	3	119,619,987	T	A	0.919	0.998	GSK3B
rs28516957	3	119,620,753	T	C	0.788	0.999	GSK3B
rs778777159	3	119,620,824	GAAAGAAAATGA	G	0.832	0.998	GSK3B
rs79164020	3	119,621,286	C	T	0.919	0.999	GSK3B
rs149185685	3	119,621,835	T	TA	0.987	0.945	GSK3B
rs4340737	3	119,622,014	C	T	0.831	0.999	GSK3B
3:119622320_AC_A	3	119,622,320	AC	A	0.87	1	GSK3B
3:119623017_GA_G	3	119,623,017	GA	G	0.679	0.997	GSK3B
rs56114552	3	119,624,221	G	A	0.87	1	GSK3B
rs9856817	3	119,625,419	G	A	0.679	0.996	GSK3B
rs1574154	3	119,627,247	T	C	0.831	0.999	GSK3B
rs115964768	3	119,627,774	G	T	0.985	0.982	GSK3B
rs183651570	3	119,628,139	T	C	0.989	0.931	GSK3B
rs78268305	3	119,628,412	G	A	0.919	0.998	GSK3B
rs1579031	3	119,629,921	G	T	0.788	0.999	GSK3B
rs78264027	3	119,630,479	T	C	0.919	0.999	GSK3B
rs73175857	3	119,630,502	G	A	0.964	0.962	GSK3B
rs75218511	3	119,630,650	C	T	0.919	0.999	GSK3B
rs13314595	3	119,631,098	C	T	0.788	0.999	GSK3B
rs6438552	3	119,631,814	A	G	0.619	1	GSK3B
rs546200535	3	119,632,507	C	CA	0.951	0.785	GSK3B
rs79946748	3	119,632,832	A	T	0.977	0.981	GSK3B
rs145325471	3	119,633,858	G	C	0.986	0.971	GSK3B
rs115290719	3	119,633,961	T	A	0.975	0.987	GSK3B
rs78889159	3	119,634,227	T	C	0.986	0.991	GSK3B
rs73175858	3	119,634,453	C	T	0.87	1	GSK3B
rs4072520	3	119,635,393	C	A	0.788	0.999	GSK3B
rs6792572	3	119,636,096	A	C	0.619	1	GSK3B
rs2319399	3	119,636,207	G	A	0.619	1	GSK3B
rs2319400	3	119,636,519	A	G	0.679	0.965	GSK3B
rs200611894	3	119,639,305	C	CTT	0.989	0.965	GSK3B
rs6438553	3	119,640,029	A	T	0.619	1	GSK3B
rs146699348	3	119,640,252	C	T	0.979	0.976	GSK3B
3:119641075_GGGAA_G	3	119,641,075	GGGAA	G	0.583	0.83	GSK3B
rs1980718	3	119,641,266	T	G	0.831	0.999	GSK3B
rs539174362	3	119,641,367	C	CTAT	0.985	0.967	GSK3B
rs7431209	3	119,641,442	A	G	0.287	0.997	GSK3B
rs148365397	3	119,641,732	T	C	0.974	0.938	GSK3B
rs7620750	3	119,643,181	G	A	0.788	0.999	GSK3B
3:119643386_CAG_C	3	119,643,386	CAG	C	0.919	0.998	GSK3B
rs77830330	3	119,643,710	G	A	0.919	0.999	GSK3B
rs9815699	3	119,644,005	A	G	0.678	0.998	GSK3B
rs373724636	3	119,645,175	CCA	C	0.784	0.941	GSK3B
rs550807964	3	119,645,177	A	C	0.986	0.785	GSK3B
rs73175860	3	119,645,598	T	A	0.87	1	GSK3B
rs9873477	3	119,645,976	A	G	0.619	1	GSK3B
rs188639452	3	119,647,261	G	C	0.986	0.894	GSK3B
rs9879295	3	119,647,313	A	G	0.619	1	GSK3B
rs73175862	3	119,647,691	A	G	0.87	1	GSK3B
rs34759275	3	119,648,015	A	G	0.989	0.976	GSK3B
rs148503172	3	119,648,451	C	CA	0.87	1	GSK3B
rs112737874	3	119,648,471	C	A	0.87	1	GSK3B
rs148961755	3	119,648,761	A	T	0.914	0.953	GSK3B
rs12106691	3	119,648,764	A	T	0.461	0.951	GSK3B
rs13064921	3	119,648,875	G	A	0.981	0.864	GSK3B
rs377492414	3	119,648,972	G	A	0.983	0.81	GSK3B
rs369429759	3	119,648,973	T	C	0.983	0.81	GSK3B
rs9878473	3	119,650,788	T	C	0.619	1	GSK3B
3:119652027_ATAAT_A	3	119,652,027	ATAAT	A	0.977	0.986	GSK3B
3:119653045_GA_G	3	119,653,045	GA	G	0.989	0.954	GSK3B
rs201865278	3	119,655,201	A	AT	0.816	0.99	GSK3B
rs55765160	3	119,655,201	A	T	0.965	0.545	GSK3B
rs6797459	3	119,655,338	C	T	0.831	0.999	GSK3B
rs66650215	3	119,657,271	C	A	0.789	0.999	GSK3B
rs62264734	3	119,657,519	C	A	0.831	0.998	GSK3B
rs149862075	3	119,657,930	G	A	0.987	0.927	GSK3B
rs77733145	3	119,658,323	T	C	0.986	0.989	GSK3B
rs6807868	3	119,658,705	C	T	0.919	0.999	GSK3B
rs781772362	3	119,659,861	ATT	A	0.872	0.864	GSK3B
rs6775397	3	119,661,373	A	C	0.798	1	GSK3B
rs4688046	3	119,663,870	T	C	0.831	1	GSK3B
rs62264737	3	119,665,041	T	C	0.969	0.983	GSK3B
rs13312998	3	119,666,266	C	T	0.919	0.999	GSK3B
rs140888610	3	119,667,022	C	CA	0.841	0.99	GSK3B
rs73175864	3	119,667,858	T	G	0.881	1	GSK3B
rs12054090	3	119,668,285	T	C	0.831	1	GSK3B
rs10934502	3	119,669,136	T	C	0.831	1	GSK3B
rs148243075	3	119,669,640	A	G	0.985	0.936	GSK3B
rs7649661	3	119,670,140	A	T	0.79	0.996	GSK3B
rs142892355	3	119,671,227	C	T	0.987	0.947	GSK3B
rs750123919	3	119,671,334	CA	C	0.744	0.989	GSK3B
rs77178661	3	119,671,656	A	G	0.881	0.999	GSK3B
rs142307992	3	119,672,000	G	A	0.989	0.971	GSK3B
rs747451988	3	119,672,135	TATAAC	T	0.798	0.996	GSK3B
3:119672303_CACAA_C	3	119,672,303	CACAA	C	0.881	0.999	GSK3B
rs74788098	3	119,673,154	A	T	0.982	0.923	GSK3B
3:119673157_AAACAG_A	3	119,673,157	AAACAG	A	0.883	0.956	GSK3B
3:119673163_AACAG_A	3	119,673,163	AACAG	A	0.905	0.899	GSK3B
3:119673269_GT_G	3	119,673,269	GT	G	0.629	0.999	GSK3B
rs763696648	3	119,674,081	TA	T	0.983	0.729	GSK3B
3:119674496_TTTGTTG_T	3	119,674,496	TTTGTTG	T	0.831	1	GSK3B
rs115950158	3	119,674,978	G	C	0.947	0.973	GSK3B
rs6785050	3	119,676,592	G	C	0.919	0.999	GSK3B
rs570754109	3	119,677,229	G	GGGGT	0.919	0.997	GSK3B
3:119677611_AATGAAAAGAGTGTTCACTTTTTTC_A	3	119,677,611	AATGAAAAGAGTGTTCACTTTTTTC	A	0.892	0.892	GSK3B
rs7636600	3	119,678,595	C	G	0.799	1	GSK3B
rs1381841	3	119,678,802	A	G	0.881	0.999	GSK3B
rs12634476	3	119,678,825	G	A	0.832	0.998	GSK3B
3:119678998_CA_C	3	119,678,998	CA	C	0.693	0.882	GSK3B
rs57695730	3	119,679,400	A	G	0.629	1	GSK3B
rs77830433	3	119,680,558	T	C	0.919	0.999	GSK3B
rs6774210	3	119,680,742	A	T	0.294	0.995	GSK3B
rs146499616	3	119,682,380	G	A	0.984	0.983	GSK3B
rs4688047	3	119,682,761	C	T	0.831	1	GSK3B
rs4688048	3	119,682,965	G	C	0.88	0.996	GSK3B
rs78289063	3	119,683,018	T	C	0.971	0.99	GSK3B
rs147161569	3	119,683,885	CT	C	0.747	0.994	GSK3B
rs111968754	3	119,684,100	G	A	0.881	1	GSK3B
rs116101751	3	119,684,218	T	A	0.881	0.999	GSK3B
rs6769435	3	119,684,407	A	C	0.798	1	GSK3B
rs3853592	3	119,685,021	A	G	0.919	0.999	GSK3B
rs17810302	3	119,685,477	A	G	0.881	1	GSK3B
rs78812449	3	119,686,237	T	A	0.918	0.998	GSK3B
rs28536662	3	119,688,191	G	A	0.629	1	GSK3B
rs62264739	3	119,688,402	A	G	0.831	1	GSK3B
rs73175870	3	119,688,659	C	T	0.881	1	GSK3B
rs6762640	3	119,689,588	C	T	0.919	0.999	GSK3B
rs140430600	3	119,689,653	C	CA	0.831	1	GSK3B
rs79669032	3	119,689,657	A	C	0.877	0.987	GSK3B
rs138264352	3	119,690,704	C	T	0.919	0.997	GSK3B
rs149176944	3	119,690,711	G	C	0.919	0.997	GSK3B
rs10934503	3	119,691,167	T	C	0.831	1	GSK3B
rs7643875	3	119,691,388	C	T	0.629	1	GSK3B
rs7611736	3	119,691,712	T	C	0.63	1	GSK3B
rs6438554	3	119,691,737	T	C	0.919	0.999	GSK3B
rs10433404	3	119,693,240	C	G	0.832	0.999	GSK3B
rs6771023	3	119,693,611	T	C	0.799	1	GSK3B
rs62264740	3	119,694,893	A	G	0.953	0.996	GSK3B
rs76067602	3	119,696,632	T	A	0.919	0.999	GSK3B
rs17204542	3	119,696,709	C	T	0.987	0.98	GSK3B
rs58605236	3	119,697,032	T	A	0.63	0.999	GSK3B
rs6795874	3	119,697,054	A	G	0.798	1	GSK3B
rs78639749	3	119,697,586	G	A	0.919	0.999	GSK3B
rs11921053	3	119,698,226	T	C	0.799	1	GSK3B
rs62264742	3	119,699,490	C	T	0.832	0.999	GSK3B
rs141177552	3	119,700,073	G	A	0.986	0.978	GSK3B
rs115098250	3	119,700,371	C	T	0.987	0.933	GSK3B
rs142778240	3	119,701,080	T	C	0.985	0.985	GSK3B
rs73175873	3	119,701,503	G	C	0.881	1	GSK3B
rs28831519	3	119,702,857	T	C	0.832	0.999	GSK3B
rs62264743	3	119,703,083	T	G	0.832	0.999	GSK3B
rs140899221	3	119,703,176	T	C	0.986	0.955	GSK3B
3:119703242_TA_T	3	119,703,242	TA	T	0.639	0.987	GSK3B
rs9680935	3	119,703,243	A	C	0.88	0.66	GSK3B
rs75843409	3	119,703,244	A	C	0.639	0.987	GSK3B
rs146382553	3	119,703,900	C	T	0.987	0.974	GSK3B
rs9853677	3	119,704,352	C	T	0.644	0.997	GSK3B
rs148927146	3	119,704,521	G	A	0.922	0.978	GSK3B
rs144518957	3	119,704,594	C	T	0.986	0.99	GSK3B
rs115653567	3	119,705,384	C	G	0.919	0.999	GSK3B
rs56126162	3	119,705,459	A	G	0.813	0.997	GSK3B

SNP	Chromosome	Position	Effect Allele	Other Allele	Effect Allele Frequency	Imputation quality	Gene
rs9847101	3	119,706,081	A	C	0.679	0.991	GSK3B
rs142373164	3	119,706,083	T	G	0.919	0.999	GSK3B
rs80165144	3	119,706,673	C	T	0.881	0.999	GSK3B
rs75044742	3	119,707,307	G	A	0.933	0.994	GSK3B
3:119707574_AG_A	3	119,707,574	AG	A	0.98	0.986	GSK3B
rs11915717	3	119,707,618	C	T	0.832	0.999	GSK3B
rs116751642	3	119,708,683	C	T	0.922	0.98	GSK3B
rs7635604	3	119,709,304	T	C	0.919	0.999	GSK3B
rs143900961	3	119,709,706	C	T	0.985	0.983	GSK3B
rs73175877	3	119,710,977	T	C	0.881	0.999	GSK3B
rs540364070	3	119,711,992	G	GA	0.8	0.994	GSK3B
rs9879992	3	119,712,721	A	G	0.798	1	GSK3B
rs9866394	3	119,712,875	T	A	0.63	1	GSK3B
3:119713907_AC_A	3	119,713,907	AC	A	0.914	0.865	GSK3B
3:119713909_AC_A	3	119,713,909	AC	A	0.881	1	GSK3B
rs7645180	3	119,714,502	A	G	0.881	0.999	GSK3B
rs77531822	3	119,714,517	C	T	0.919	0.999	GSK3B
3:119714584_AT_A	3	119,714,584	AT	A	0.764	0.918	GSK3B
rs139298095	3	119,714,660	C	G	0.989	0.916	GSK3B
3:119715443_GT_G	3	119,715,443	GT	G	0.935	0.83	GSK3B
rs73175879	3	119,715,576	T	C	0.881	0.999	GSK3B
rs6784239	3	119,716,742	G	C	0.919	0.999	GSK3B
rs149018603	3	119,716,802	T	G	0.987	0.971	GSK3B
rs759479742	3	119,716,906	TA	T	0.881	0.998	GSK3B
rs368442210	3	119,719,668	CA	C	0.92	0.789	GSK3B
rs79115692	3	119,719,896	C	T	0.919	0.999	GSK3B
rs747471558	3	119,720,015	G	T	0.966	0.541	GSK3B
rs12108149	3	119,721,171	T	C	0.831	1	GSK3B
rs201267724	3	119,721,195	T	TC	0.97	0.928	GSK3B
rs113465663	3	119,722,960	G	A	0.98	0.981	GSK3B
rs73175880	3	119,723,117	A	C	0.881	0.999	GSK3B
rs190230247	3	119,724,566	C	A	0.979	0.986	GSK3B
rs144840851	3	119,724,769	A	G	0.962	0.98	GSK3B
rs6796420	3	119,724,994	G	A	0.629	0.999	GSK3B
rs4688053	3	119,725,016	G	A	0.831	0.999	GSK3B
rs11915898	3	119,725,153	A	G	0.881	0.999	GSK3B
rs58043853	3	119,726,417	C	T	0.799	0.999	GSK3B
rs148898792	3	119,726,437	C	T	0.94	0.993	GSK3B
rs187045295	3	119,727,515	A	G	0.985	0.938	GSK3B
rs80087735	3	119,728,170	C	G	0.919	0.998	GSK3B
rs575769812	3	119,729,603	C	CTAAA	0.98	0.958	GSK3B
rs6774693	3	119,729,656	T	C	0.799	0.999	GSK3B
rs60272712	3	119,732,508	G	A	0.919	0.999	GSK3B
3:119732699_TA_T	3	119,732,699	TA	T	0.746	0.95	GSK3B
rs17204605	3	119,733,664	G	A	0.88	0.999	GSK3B
rs4688054	3	119,733,710	T	C	0.831	0.999	GSK3B
rs116755725	3	119,734,170	T	C	0.955	0.993	GSK3B
3:119734496_CAAAAAAAAAAAAAA_C	3	119,734,496	CAAAAAAAAAAAAAA	C	0.767	0.979	GSK3B
rs76330913	3	119,734,836	T	C	0.919	0.998	GSK3B
rs1154595	3	119,736,594	C	T	0.631	0.999	GSK3B
rs1154596	3	119,736,713	C	T	0.919	0.999	GSK3B
rs78796384	3	119,736,734	T	C	0.967	0.986	GSK3B
rs1154597	3	119,736,833	A	G	0.919	0.999	GSK3B
rs9289138	3	119,739,297	T	C	0.798	0.999	GSK3B
rs7613197	3	119,740,940	C	T	0.798	0.999	GSK3B
rs62264747	3	119,741,298	T	C	0.831	0.999	GSK3B
rs6770314	3	119,742,721	G	A	0.798	0.999	GSK3B
rs75008340	3	119,742,867	C	T	0.969	0.995	GSK3B
3:119742991_AAT_A	3	119,742,991	AAT	A	0.831	0.999	GSK3B
rs1903765	3	119,743,122	A	G	0.63	0.999	GSK3B
rs73175886	3	119,744,018	C	G	0.881	0.999	GSK3B
rs79250871	3	119,745,187	G	A	0.919	0.999	GSK3B
3:119745359_TACACACAC_T	3	119,745,359	TACACACAC	T	0.661	0.975	GSK3B
rs13082848	3	119,745,641	G	A	0.96	0.952	GSK3B
rs11923854	3	119,746,569	T	C	0.881	0.999	GSK3B
rs11919783	3	119,746,633	G	A	0.881	0.999	GSK3B
rs142682932	3	119,747,367	C	T	0.989	0.972	GSK3B
rs78497202	3	119,747,633	C	T	0.919	0.999	GSK3B
rs62264749	3	119,747,919	T	C	0.831	0.999	GSK3B
rs9851174	3	119,749,411	G	A	0.798	0.999	GSK3B
rs12635018	3	119,749,770	C	T	0.831	0.999	GSK3B
rs10433339	3	119,750,146	G	A	0.88	0.996	GSK3B
rs73175888	3	119,750,172	A	G	0.881	0.999	GSK3B
rs142570552	3	119,750,255	T	TGGGTCCAGGAAGCA	0.987	0.979	GSK3B
rs6797689	3	119,750,636	C	T	0.918	0.997	GSK3B
3:119751323_GA_G	3	119,751,323	GA	G	0.771	0.91	GSK3B
rs7628426	3	119,751,387	G	A	0.918	0.997	GSK3B
rs73175889	3	119,752,151	C	T	0.881	0.998	GSK3B
rs73175891	3	119,752,152	A	T	0.881	0.999	GSK3B
rs78883820	3	119,752,368	T	A	0.927	0.995	GSK3B
rs2873968	3	119,752,528	A	C	0.056	0.976	GSK3B
3:119752955_GTTC_G	3	119,752,955	GTTC	G	0.831	0.999	GSK3B
rs74588474	3	119,753,103	T	C	0.987	0.981	GSK3B
rs6802301	3	119,753,324	T	A	0.798	0.999	GSK3B
rs2126583	3	119,753,820	G	A	0.629	0.998	GSK3B
rs75352604	3	119,755,468	C	T	0.918	0.997	GSK3B
rs142495292	3	119,755,887	C	T	0.989	0.976	GSK3B
rs77263312	3	119,755,977	T	C	0.918	0.997	GSK3B
rs12630829	3	119,756,170	A	G	0.829	0.998	GSK3B
rs115327285	3	119,756,238	A	T	0.987	0.99	GSK3B
rs10934504	3	119,756,947	T	C	0.987	0.99	GSK3B
rs1870931	3	119,757,013	C	G	0.829	0.998	GSK3B
rs138202179	3	119,757,265	C	T	0.981	0.974	GSK3B
rs112502828	3	119,757,827	G	GAACTCTTAA	0.034	0.914	GSK3B
rs6781942	3	119,758,049	A	G	0.629	0.999	GSK3B
rs7650554	3	119,758,796	C	T	0.918	0.997	GSK3B
rs11719490	3	119,758,897	G	C	0.909	0.972	GSK3B
rs4687890	3	119,759,173	A	G	0.629	0.999	GSK3B
rs75912592	3	119,759,222	G	A	0.955	0.995	GSK3B
rs79273052	3	119,759,942	C	T	0.918	0.997	GSK3B
rs16830683	3	119,760,168	C	T	0.918	0.996	GSK3B
rs73175893	3	119,760,665	T	C	0.926	0.96	GSK3B
rs115158667	3	119,760,671	G	C	0.962	0.954	GSK3B
rs55645853	3	119,762,756	T	C	0.963	0.954	GSK3B
rs6438555	3	119,762,844	T	C	0.799	0.999	GSK3B
rs9859778	3	119,763,607	A	C	0.799	0.998	GSK3B
rs140021862	3	119,763,839	G	A	0.986	0.97	GSK3B
rs7652172	3	119,764,086	C	T	0.798	0.999	GSK3B
rs75299225	3	119,767,243	C	A	0.987	0.859	GSK3B
rs74646194	3	119,767,244	A	T	0.987	0.859	GSK3B
rs10934505	3	119,767,322	A	T	0.831	0.999	GSK3B
rs12489805	3	119,767,693	T	A	0.989	0.975	GSK3B
rs12630592	3	119,768,246	G	T	0.63	0.999	GSK3B
rs79546368	3	119,770,054	G	C	0.919	0.996	GSK3B
rs150893077	3	119,770,450	C	T	0.987	0.988	GSK3B
rs145027294	3	119,770,613	C	CA	0.86	0.955	GSK3B
rs10934506	3	119,770,652	T	C	0.831	0.998	GSK3B
rs10934507	3	119,770,660	T	A	0.831	0.998	GSK3B
rs16830689	3	119,771,271	G	C	0.798	0.998	GSK3B
3:119771642_AATT_A	3	119,771,642	AATT	A	0.948	0.973	GSK3B
rs968824	3	119,773,040	A	G	0.799	0.998	GSK3B
rs142112671	3	119,773,160	A	C	0.986	0.982	GSK3B
rs62266273	3	119,774,346	G	A	0.976	0.996	GSK3B
rs13080945	3	119,774,858	A	G	0.98	0.981	GSK3B
rs6779828	3	119,775,147	C	T	0.798	0.998	GSK3B
rs4687891	3	119,775,177	T	C	0.88	0.995	GSK3B
rs4687892	3	119,775,428	T	C	0.63	0.998	GSK3B
rs4687893	3	119,775,514	T	A	0.63	0.998	GSK3B
rs143086175	3	119,775,584	G	C	0.98	0.968	GSK3B
rs17204878	3	119,776,880	G	T	0.63	0.998	GSK3B
rs76302983	3	119,777,330	A	T	0.955	0.994	GSK3B
rs2199503	3	119,778,489	T	C	0.288	0.99	GSK3B
rs34740713	3	119,778,601	C	T	0.971	0.998	GSK3B
rs79729654	3	119,779,115	G	A	0.919	0.997	GSK3B
rs35945043	3	119,779,683	A	G	0.971	0.998	GSK3B
rs114741212	3	119,780,415	G	A	0.982	0.932	GSK3B
rs17810676	3	119,780,549	T	C	0.629	0.998	GSK3B
rs200808389	3	119,780,569	A	AATTT	0.969	0.984	GSK3B
rs114210378	3	119,780,789	C	T	0.881	0.998	GSK3B
rs62266274	3	119,780,828	C	G	0.832	0.998	GSK3B
3:119782277_AC_A	3	119,782,277	AC	A	0.959	0.966	GSK3B
rs334560	3	119,782,470	G	A	0.799	0.998	GSK3B
rs145174301	3	119,782,801	T	C	0.987	0.988	GSK3B
rs73175897	3	119,783,487	G	A	0.881	0.998	GSK3B
rs73175899	3	119,784,330	G	A	0.918	0.985	GSK3B
rs73175900	3	119,784,516	G	C	0.985	0.97	GSK3B
rs6805931	3	119,784,746	T	C	0.919	0.998	GSK3B
rs140654546	3	119,784,792	G	A	0.989	0.978	GSK3B
rs334561	3	119,784,996	C	A	0.919	0.997	GSK3B
rs58863896	3	119,785,527	C	A	0.832	0.998	GSK3B
rs75142045	3	119,785,723	C	T	0.955	0.994	GSK3B
rs139126145	3	119,785,792	G	A	0.986	0.989	GSK3B
3:119786083_CA_C	3	119,786,083	CA	C	0.617	0.979	GSK3B
rs140882855	3	119,786,105	G	T	0.987	0.976	GSK3B
rs76241694	3	119,786,489	C	A	0.987	0.988	GSK3B

SNP	Chromosome	Position	Effect Allele	Other Allele	Effect Allele Frequency	Imputation quality	Gene
rs334563	3	119,786,887	C	A	0.631	0.998	GSK3B
rs10934508	3	119,786,973	C	T	0.677	0.991	GSK3B
rs334564	3	119,787,022	A	G	0.631	0.998	GSK3B
rs139168580	3	119,787,111	G	A	0.976	0.951	GSK3B
rs533506521	3	119,787,194	C	T	0.98	0.957	GSK3B
rs112434478	3	119,787,276	G	A	0.881	0.998	GSK3B
rs334566	3	119,787,993	T	C	0.799	0.998	GSK3B
rs570238285	3	119,788,512	G	GA	0.645	0.959	GSK3B
3:119789087_CA_C	3	119,789,087	CA	C	0.629	0.987	GSK3B
rs76892778	3	119,789,099	AC	A	0.987	0.529	GSK3B
3:119789125_TA_T	3	119,789,125	TA	T	0.99	0.653	GSK3B
rs182839	3	119,790,194	T	C	0.919	0.997	GSK3B
rs334536	3	119,790,712	A	C	0.799	0.998	GSK3B
rs73177903	3	119,791,047	T	G	0.954	0.992	GSK3B
rs147052362	3	119,792,288	G	A	0.987	0.922	GSK3B
rs334535	3	119,792,304	C	T	0.798	0.997	GSK3B
rs17205095	3	119,792,474	G	A	0.881	0.997	GSK3B
rs334534	3	119,792,609	C	T	0.919	0.996	GSK3B
rs62266276	3	119,792,869	T	C	0.986	0.979	GSK3B
rs334533	3	119,792,882	C	T	0.631	0.997	GSK3B
rs62266277	3	119,793,026	T	A	0.832	0.996	GSK3B
rs114692161	3	119,793,109	C	T	0.987	0.985	GSK3B
3:119793199_CT_C	3	119,793,199	CT	C	0.752	0.961	GSK3B
rs74840836	3	119,794,125	C	T	0.985	0.979	GSK3B
rs58959678	3	119,794,284	C	T	0.954	0.996	GSK3B
rs334551	3	119,795,518	G	A	0.919	0.993	GSK3B
rs13096914	3	119,795,586	G	A	0.971	0.997	GSK3B
rs113642756	3	119,795,616	G	A	0.88	0.993	GSK3B
rs75192446	3	119,795,926	C	T	0.926	0.993	GSK3B
rs141718232	3	119,796,120	T	C	0.982	0.915	GSK3B
rs334552	3	119,796,455	G	A	0.919	0.996	GSK3B
3:119797392_CT_C	3	119,797,392	CT	C	0.818	0.939	GSK3B
rs112003116	3	119,797,761	T	TACAC	0.791	0.977	GSK3B
rs334553	3	119,797,879	G	C	0.63	0.996	GSK3B
rs141116688	3	119,797,983	T	G	0.974	0.994	GSK3B
rs73177904	3	119,798,122	C	G	0.88	0.997	GSK3B
3:119798152_TACACAC_T	3	119,798,152	TACACAC	T	0.206	0.967	GSK3B
3:119798206_TTTA_T	3	119,798,206	TTTA	T	0.926	0.927	GSK3B
rs11916594	3	119,798,310	C	T	0.88	0.997	GSK3B
rs73177905	3	119,799,365	C	T	0.88	0.997	GSK3B
rs112120669	3	119,799,539	G	A	0.88	0.997	GSK3B
rs116103058	3	119,800,170	C	T	0.918	0.983	GSK3B
rs141014865	3	119,801,015	C	A	0.987	0.984	GSK3B
rs62266280	3	119,801,267	A	C	0.976	0.998	GSK3B
rs143571073	3	119,802,217	A	C	0.986	0.983	GSK3B
rs334554	3	119,802,373	T	C	0.919	0.996	GSK3B
rs334555	3	119,804,136	G	C	0.157	0.976	GSK3B
rs62266281	3	119,804,462	C	T	0.833	0.995	GSK3B
rs73177907	3	119,805,093	A	G	0.88	0.996	GSK3B
rs16830699	3	119,805,898	G	A	0.986	0.988	GSK3B
3:119807130_CTGAG_C	3	119,807,130	CTGAG	C	0.919	0.989	GSK3B
rs146359072	3	119,807,828	C	G	0.987	0.983	GSK3B
rs60235167	3	119,808,256	G	A	0.832	0.993	GSK3B
3:119809077_TA_T	3	119,809,077	TA	T	0.63	0.985	GSK3B
rs334559	3	119,809,605	G	A	0.798	0.994	GSK3B
rs141233431	3	119,810,048	A	C	0.987	0.983	GSK3B
rs112234287	3	119,810,580	G	A	0.971	0.98	GSK3B
rs11918691	3	119,810,617	C	T	0.955	0.991	GSK3B
3:119810671_GA_G	3	119,810,671	GA	G	0.883	0.888	GSK3B
rs148384822	3	119,810,839	T	A	0.984	0.968	GSK3B
rs1387095	3	119,811,467	G	A	0.989	0.978	GSK3B
rs4688059	3	119,811,568	G	A	0.833	0.99	GSK3B
rs41271403	3	119,812,822	C	T	0.966	0.93	GSK3B
rs334558	3	119,813,282	A	G	0.664	0.993	GSK3B
rs9878092	3	124,480,843	A	G	0.823	0.999	ITGB5
rs14386	3	124,481,052	G	A	0.823	0.999	ITGB5
rs849019	3	124,481,461	A	T	0.4	0.996	ITGB5
rs116182279	3	124,481,506	G	A	0.988	0.851	ITGB5
rs112020282	3	124,481,542	A	ACAAGGGAGGGAGAC	0.41	0.981	ITGB5
rs138438169	3	124,481,609	G	GTATC	0.824	0.994	ITGB5
rs17282078	3	124,481,760	T	A	0.869	0.996	ITGB5
rs2676	3	124,481,987	A	G	0.823	0.998	ITGB5
rs2675	3	124,482,113	T	G	0.823	0.998	ITGB5
rs2674	3	124,482,222	C	T	0.859	0.988	ITGB5
rs745372859	3	124,482,494	TTTGTTGAAC	T	0.87	0.986	ITGB5
rs585021	3	124,482,869	C	T	0.868	0.919	ITGB5
rs10934691	3	124,483,056	C	G	0.784	0.987	ITGB5
rs61761681	3	124,483,190	G	A	0.871	0.987	ITGB5
rs61761680	3	124,483,230	G	A	0.987	0.942	ITGB5
rs61761679	3	124,483,420	G	C	0.963	0.931	ITGB5
rs582170	3	124,483,527	T	A	0.967	0.987	ITGB5
rs6782056	3	124,483,541	G	A	0.833	0.998	ITGB5
rs6795142	3	124,483,697	T	C	0.833	0.999	ITGB5
rs3772812	3	124,483,833	C	T	0.809	0.998	ITGB5
rs3821536	3	124,483,952	T	C	0.833	0.999	ITGB5
rs3772813	3	124,484,073	T	C	0.833	0.999	ITGB5
rs1877568	3	124,484,187	G	T	0.797	0.999	ITGB5
rs3772815	3	124,484,319	A	G	0.833	0.999	ITGB5
rs1877567	3	124,484,429	G	T	0.833	0.998	ITGB5
rs760059985	3	124,484,549	ATTCATCT	A	0.833	0.998	ITGB5
3:124484906_CAA_C	3	124,484,906	CAA	C	0.906	0.746	ITGB5
rs2291081	3	124,485,235	A	G	0.833	0.999	ITGB5
rs3772816	3	124,485,800	G	A	0.833	0.999	ITGB5
rs3772817	3	124,485,870	A	G	0.833	0.999	ITGB5
rs16835962	3	124,485,948	G	A	0.975	0.976	ITGB5
rs3772818	3	124,486,026	A	G	0.833	0.999	ITGB5
rs3772820	3	124,486,343	G	A	0.833	0.999	ITGB5
rs614664	3	124,486,993	C	A	0.628	0.995	ITGB5
rs6792936	3	124,487,100	C	T	0.833	0.999	ITGB5
rs6768588	3	124,487,335	A	G	0.797	1	ITGB5
rs74885273	3	124,487,530	C	T	0.974	0.989	ITGB5
rs3816693	3	124,487,829	G	A	0.988	0.999	ITGB5
rs2291082	3	124,487,849	A	G	0.797	1	ITGB5
rs2291083	3	124,488,101	T	A	0.988	0.999	ITGB5
rs2840061	3	124,488,267	G	A	0.833	0.999	ITGB5
rs115080852	3	124,488,557	G	A	0.98	0.983	ITGB5
rs2035265	3	124,488,610	T	G	0.797	0.999	ITGB5
3:124489739_CG_C	3	124,489,739	CG	C	0.852	0.99	ITGB5
3:124489934_TG_T	3	124,489,934	TG	T	0.833	0.998	ITGB5
rs12488326	3	124,490,088	A	G	0.797	0.999	ITGB5
rs76720875	3	124,490,139	A	G	0.988	0.999	ITGB5
rs138675306	3	124,490,204	T	C	0.982	0.999	ITGB5
rs12494846	3	124,490,250	C	T	0.833	0.999	ITGB5
rs12494985	3	124,490,575	G	A	0.809	0.999	ITGB5
3:124490926_GT_G	3	124,490,926	GT	G	0.833	0.998	ITGB5
rs3772822	3	124,491,029	G	A	0.988	0.999	ITGB5
rs751758785	3	124,491,532	ACTC	A	0.833	0.997	ITGB5
rs1980078	3	124,491,659	T	C	0.797	0.999	ITGB5
rs7648120	3	124,491,874	T	C	0.833	0.999	ITGB5
rs1871397	3	124,492,000	C	T	0.833	0.999	ITGB5
rs16835978	3	124,492,734	T	C	0.979	0.978	ITGB5
rs4558801	3	124,493,428	A	G	0.833	0.999	ITGB5
rs11720803	3	124,493,542	G	A	0.988	0.999	ITGB5
rs657571	3	124,493,561	T	C	0.967	0.991	ITGB5
rs5852429	3	124,493,629	A	AG	0.833	0.997	ITGB5
rs74708445	3	124,493,860	T	C	0.964	0.998	ITGB5
rs621209	3	124,494,030	G	A	0.788	0.991	ITGB5
rs774266528	3	124,494,121	ATGTT	A	0.964	0.894	ITGB5
3:124494140_AGT_A	3	124,494,140	AGT	A	0.987	0.849	ITGB5
rs547326252	3	124,494,150	A	AGT	0.833	0.991	ITGB5
rs3755706	3	124,494,286	C	T	0.833	0.999	ITGB5
rs11710274	3	124,494,363	T	G	0.964	0.998	ITGB5
3:124495110_GCAC_G	3	124,495,110	GCAC	G	0.982	0.983	ITGB5
rs56750599	3	124,495,252	T	G	0.976	0.997	ITGB5
rs755064256	3	124,495,717	CTTTT	C	0.835	0.993	ITGB5
rs76303575	3	124,495,727	A	G	0.833	0.998	ITGB5
rs77810504	3	124,495,732	C	G	0.833	0.999	ITGB5
rs3836311	3	124,495,851	G	GC	0.833	0.995	ITGB5
rs28553402	3	124,496,158	G	A	0.833	0.999	ITGB5
rs76718781	3	124,496,327	C	T	0.97	0.99	ITGB5
rs117204689	3	124,496,443	T	C	0.988	0.997	ITGB5
rs13064025	3	124,496,477	T	C	0.833	0.999	ITGB5
rs62265637	3	124,496,529	T	C	0.851	0.993	ITGB5
rs2089982	3	124,496,869	T	C	0.982	0.999	ITGB5
rs2083425	3	124,496,888	G	T	0.833	0.999	ITGB5
rs185721832	3	124,497,427	G	T	0.989	0.863	ITGB5
rs3772825	3	124,497,606	G	A	0.833	0.999	ITGB5
rs16835991	3	124,497,966	C	T	0.976	0.997	ITGB5
rs11425382	3	124,498,095	C	CA	0.833	0.998	ITGB5
rs35473994	3	124,498,105	G	A	0.833	0.999	ITGB5
rs9879289	3	124,498,173	G	A	0.851	0.998	ITGB5
rs9879197	3	124,498,276	C	T	0.833	0.999	ITGB5
rs3772826	3	124,498,743	T	C	0.833	0.999	ITGB5
3:124498939_CGAGGCCTCTGAGGCAGGGCACGGGGACCCTCAGG_C	3	124,498,939	CGAGGCCTCTGAGGCAGGGCACGGGGACCCTCAGG	C	0.964	0.989	ITGB5
3:124498975_AGGCCTCTGAGGCAGGGCACAGGGACCATGGGGACCCTCAGCAG_A	3	124,498,975	AGGCCTCTGAGGCAGGGCACAGGGACCATGGGGACCCTCAGCAG	A	0.964	0.987	ITGB5

SNP	Chromosome	Position	Effect Allele	Other Allele	Effect Allele Frequency	Imputation quality	Gene
rs375611601	3	124,499,017	A	G	0.965	0.979	ITGB5
rs370245157	3	124,499,018	G	A	0.965	0.979	ITGB5
rs2035264	3	124,499,498	T	C	0.666	0.994	ITGB5
3:124499556_TC_T	3	124,499,556	TC	T	0.778	0.952	ITGB5
rs3772827	3	124,499,685	C	T	0.984	0.995	ITGB5
rs16835998	3	124,500,094	C	T	0.976	0.997	ITGB5
rs702037	3	124,500,541	C	G	0.263	0.986	ITGB5
rs6787433	3	124,500,787	G	A	0.964	0.998	ITGB5
rs4677943	3	124,500,820	C	T	0.797	0.999	ITGB5
rs11707859	3	124,500,913	C	T	0.988	0.998	ITGB5
rs702036	3	124,501,072	A	G	0.627	0.995	ITGB5
rs702035	3	124,501,216	G	A	0.829	0.99	ITGB5
rs2619328	3	124,501,222	G	A	0.625	0.993	ITGB5
rs148337750	3	124,501,343	T	A	0.982	0.909	ITGB5
rs11708070	3	124,501,357	G	A	0.988	0.999	ITGB5
rs145834612	3	124,501,433	G	A	0.988	0.999	ITGB5
rs201328542	3	124,501,550	C	CA	0.988	0.982	ITGB5
rs138390202	3	124,501,582	G	A	0.97	0.999	ITGB5
rs9820662	3	124,501,687	G	A	0.98	0.988	ITGB5
rs117272604	3	124,501,743	G	A	0.988	0.999	ITGB5
rs115372785	3	124,502,072	A	G	0.97	0.999	ITGB5
rs9825554	3	124,502,331	G	A	0.98	0.989	ITGB5
rs144529212	3	124,503,210	G	A	0.97	0.999	ITGB5
rs3772829	3	124,503,574	G	C	0.988	0.999	ITGB5
rs117508167	3	124,503,774	C	T	0.988	0.999	ITGB5
rs2002238	3	124,504,285	A	C	0.814	0.989	ITGB5
rs2333023	3	124,504,430	T	C	0.966	0.964	ITGB5
rs147249322	3	124,504,755	G	GA	0.962	0.94	ITGB5
rs11708547	3	124,505,038	C	T	0.988	0.999	ITGB5
rs11712700	3	124,505,140	T	C	0.988	0.999	ITGB5
rs73197640	3	124,505,143	G	C	0.911	0.982	ITGB5
rs11716443	3	124,505,395	A	G	0.97	0.999	ITGB5
rs148389170	3	124,505,528	T	A	0.982	0.999	ITGB5
rs79524555	3	124,506,269	G	A	0.97	0.999	ITGB5
rs16836013	3	124,506,688	C	A	0.97	0.999	ITGB5
rs3821537	3	124,506,984	C	G	0.827	0.999	ITGB5
rs188439108	3	124,508,124	T	C	0.988	0.998	ITGB5
rs4678158	3	124,508,235	C	T	0.904	0.682	ITGB5
rs4677944	3	124,508,269	C	T	0.843	0.929	ITGB5
rs4677945	3	124,508,298	C	T	0.834	0.944	ITGB5
rs527238991	3	124,508,820	C	T	0.328	0.937	ITGB5
rs144195265	3	124,509,367	G	A	0.97	0.994	ITGB5
rs113432179	3	124,509,431	A	G	0.981	0.906	ITGB5
rs77145011	3	124,509,556	G	C	0.988	0.997	ITGB5
rs114263121	3	124,509,792	G	T	0.982	0.998	ITGB5
rs3755707	3	124,509,944	C	T	0.988	1	ITGB5
rs3821538	3	124,510,211	A	G	0.827	0.999	ITGB5
rs3772831	3	124,511,052	C	A	0.651	0.996	ITGB5
rs12493125	3	124,511,176	C	A	0.963	0.889	ITGB5
rs11707559	3	124,511,717	A	G	0.97	1	ITGB5
rs138255474	3	124,511,720	C	CA	0.98	0.977	ITGB5
rs116779029	3	124,511,743	G	A	0.982	0.999	ITGB5
rs4678160	3	124,512,774	G	C	0.776	0.998	ITGB5
rs115770471	3	124,513,058	A	T	0.987	0.806	ITGB5
rs16836019	3	124,513,142	C	T	0.97	1	ITGB5
rs62265639	3	124,513,344	T	C	0.884	0.983	ITGB5
rs138769569	3	124,513,578	G	A	0.98	0.989	ITGB5
rs572325847	3	124,513,644	C	CAAGA	0.802	0.999	ITGB5
rs111891999	3	124,514,104	T	TGAG	0.775	0.995	ITGB5
rs149541320	3	124,514,128	G	A	0.986	0.902	ITGB5
rs11718590	3	124,514,444	C	T	0.988	1	ITGB5
rs2291088	3	124,515,509	G	A	0.833	1	ITGB5
rs3772832	3	124,515,876	A	G	0.819	0.992	ITGB5
rs3821539	3	124,516,231	G	A	0.833	0.999	ITGB5
rs4678162	3	124,516,695	G	A	0.833	0.999	ITGB5
rs9874118	3	124,516,793	T	C	0.882	0.98	ITGB5
rs77378823	3	124,516,888	C	T	0.97	0.999	ITGB5
rs56085841	3	124,516,954	C	T	0.981	0.978	ITGB5
rs143481369	3	124,518,320	T	C	0.97	0.998	ITGB5
rs148004126	3	124,518,321	G	A	0.97	0.998	ITGB5
rs3772833	3	124,518,362	G	A	0.833	0.998	ITGB5
3:124518518_AGAAG_A	3	124,518,518	AGAAG	A	0.97	0.998	ITGB5
rs560740417	3	124,519,457	G	T	0.989	0.786	ITGB5
rs575134439	3	124,519,457	G	GCCTGATAACCAGAGAATGTTCTGTCAGATT	0.982	0.853	ITGB5
rs114337727	3	124,520,778	A	C	0.98	0.993	ITGB5
rs12487905	3	124,521,035	T	C	0.792	0.995	ITGB5
rs17282085	3	124,521,263	G	A	0.882	0.992	ITGB5
rs147776415	3	124,521,315	G	A	0.98	0.991	ITGB5
rs12695446	3	124,521,357	G	A	0.792	0.996	ITGB5
rs2619330	3	124,521,518	G	T	0.867	0.98	ITGB5
rs2650067	3	124,521,519	C	T	0.867	0.981	ITGB5
rs13314004	3	124,521,882	G	A	0.954	0.983	ITGB5
rs144787904	3	124,522,398	C	T	0.98	0.99	ITGB5
rs9830425	3	124,522,606	T	G	0.98	0.992	ITGB5
rs117034882	3	124,522,755	G	A	0.988	0.995	ITGB5
rs7646358	3	124,523,209	A	G	0.969	0.996	ITGB5
rs55787121	3	124,523,376	A	C	0.969	0.995	ITGB5
rs56243047	3	124,523,429	A	T	0.969	0.995	ITGB5
rs11707187	3	124,523,675	C	T	0.969	0.993	ITGB5
3:124523821_TTTCC_T	3	124,523,821	TTTCC	T	0.979	0.889	ITGB5
3:124523837_CTTCT_C	3	124,523,837	CTTCT	C	0.979	0.909	ITGB5
3:124523876_TTGCCC_T	3	124,523,876	TTGCCC	T	0.975	0.875	ITGB5
rs201689739	3	124,523,980	TTC	T	0.88	0.981	ITGB5
rs111230426	3	124,524,092	C	T	0.76	0.968	ITGB5
rs566057262	3	124,524,303	C	CT	0.981	0.979	ITGB5
rs72972551	3	124,525,011	T	A	0.982	0.996	ITGB5
rs16836031	3	124,525,049	A	G	0.968	0.993	ITGB5
rs4678164	3	124,525,071	G	A	0.816	0.995	ITGB5
rs6798096	3	124,525,189	T	C	0.98	0.994	ITGB5
rs147299316	3	124,525,217	C	T	0.988	0.986	ITGB5
rs575081043	3	124,525,643	G	GT	0.652	0.974	ITGB5
rs66468076	3	124,526,186	C	T	0.816	0.995	ITGB5
rs62265641	3	124,526,217	C	T	0.882	0.994	ITGB5
rs73199509	3	124,526,459	G	C	0.911	0.984	ITGB5
rs7614019	3	124,526,488	A	G	0.962	0.993	ITGB5
rs185447638	3	124,526,716	G	A	0.981	0.993	ITGB5
rs17282092	3	124,526,998	T	C	0.982	0.996	ITGB5
rs7617098	3	124,527,530	A	C	0.962	0.993	ITGB5
rs848788	3	124,528,424	T	C	0.64	0.994	ITGB5
rs6798233	3	124,528,488	C	T	0.982	0.992	ITGB5
rs114540702	3	124,528,770	G	A	0.982	0.996	ITGB5
3:124529453_TAAG_T	3	124,529,453	TAAG	T	0.987	0.987	ITGB5
rs72972563	3	124,529,513	T	G	0.982	0.996	ITGB5
rs58308063	3	124,530,014	G	C	0.98	0.993	ITGB5
rs7646206	3	124,530,245	C	T	0.981	0.993	ITGB5
rs115291145	3	124,531,016	T	C	0.982	0.995	ITGB5
rs11927550	3	124,532,774	A	C	0.962	0.993	ITGB5
rs78025535	3	124,532,912	A	C	0.988	0.993	ITGB5
rs72972570	3	124,532,997	C	T	0.982	0.995	ITGB5
rs7639092	3	124,533,137	G	C	0.981	0.99	ITGB5
rs7650893	3	124,533,315	T	C	0.981	0.989	ITGB5
3:124533325_TA_T	3	124,533,325	TA	T	0.745	0.884	ITGB5
3:124533342_GAGAC_G	3	124,533,342	GAGAC	G	0.988	0.973	ITGB5
rs6438854	3	124,533,387	A	G	0.98	0.994	ITGB5
rs140883560	3	124,533,703	G	A	0.982	0.996	ITGB5
rs139185737	3	124,534,778	T	C	0.988	0.993	ITGB5
rs116402921	3	124,535,199	G	A	0.982	0.988	ITGB5
rs3755709	3	124,535,278	G	A	0.962	0.992	ITGB5
rs3772836	3	124,535,635	C	A	0.962	0.993	ITGB5
rs3772837	3	124,535,904	A	C	0.789	0.993	ITGB5
rs61761666	3	124,535,988	A	T	0.982	0.994	ITGB5
rs2086272	3	124,536,149	C	T	0.816	0.995	ITGB5
rs61760579	3	124,537,059	C	T	0.982	0.997	ITGB5
3:124537349_CTTTTTTTTTTTTTTT_C	3	124,537,349	CTTTTTTTTTTTTTTT	C	0.167	0.939	ITGB5
rs12490538	3	124,538,764	C	A	0.816	0.997	ITGB5
rs78563193	3	124,539,325	G	A	0.98	0.996	ITGB5
rs6769379	3	124,539,834	C	G	0.797	0.998	ITGB5
rs61760577	3	124,539,881	G	A	0.982	0.998	ITGB5
rs4678166	3	124,540,556	C	T	0.876	1	ITGB5
rs3755710	3	124,540,702	T	A	0.876	1	ITGB5
3:124540737_GGCTA_G	3	124,540,737	GGCTA	G	0.673	0.995	ITGB5
rs145788378	3	124,540,774	T	TC	0.895	0.923	ITGB5
rs4678167	3	124,540,832	C	T	0.857	1	ITGB5
rs73199514	3	124,540,907	A	G	0.988	0.882	ITGB5
3:124540916_TAA_T	3	124,540,916	TAA	T	0.876	1	ITGB5
rs66464554	3	124,541,756	G	A	0.876	1	ITGB5
rs9879407	3	124,541,854	T	G	0.816	0.998	ITGB5
rs6438856	3	124,542,531	C	T	0.653	1	ITGB5
rs9860625	3	124,542,575	C	T	0.816	0.998	ITGB5
rs9861248	3	124,542,848	G	A	0.816	0.999	ITGB5
rs4678168	3	124,543,026	C	G	0.653	1	ITGB5
rs763396670	3	124,543,369	CT	C	0.797	0.998	ITGB5
rs143644448	3	124,543,827	C	T	0.987	0.991	ITGB5
rs9814959	3	124,544,018	T	C	0.98	0.993	ITGB5
3:124544043_CA_C	3	124,544,043	CA	C	0.855	0.94	ITGB5

SNP	Chromosome	Position	Effect Allele	Other Allele	Effect Allele Frequency	Imputation quality	Gene
rs73199516	3	124,544,088	T	G	0.912	0.982	ITGB5
rs7624392	3	124,544,266	T	C	0.654	0.995	ITGB5
rs76986274	3	124,544,628	C	T	0.988	0.989	ITGB5
rs115645977	3	124,544,683	T	C	0.982	0.995	ITGB5
rs138918013	3	124,545,007	A	G	0.982	0.994	ITGB5
rs6771896	3	124,545,750	G	A	0.804	0.99	ITGB5
rs4677946	3	124,546,047	A	G	0.789	0.985	ITGB5
rs4677947	3	124,546,058	G	A	0.789	0.985	ITGB5
rs73199520	3	124,546,462	G	A	0.963	0.988	ITGB5
rs147410928	3	124,546,823	C	T	0.982	0.992	ITGB5
rs56857499	3	124,546,835	G	C	0.835	0.982	ITGB5
rs115754498	3	124,547,001	G	A	0.961	0.992	ITGB5
rs114029173	3	124,547,064	G	A	0.961	0.992	ITGB5
rs848787	3	124,547,607	C	G	0.705	0.98	ITGB5
rs2076722	3	124,547,871	T	C	0.753	0.988	ITGB5
rs75951369	3	124,547,976	T	C	0.968	0.992	ITGB5
rs16836078	3	124,548,599	T	C	0.862	0.995	ITGB5
rs16836080	3	124,548,684	G	A	0.715	0.991	ITGB5
rs73199524	3	124,548,860	C	T	0.754	0.991	ITGB5
rs115385073	3	124,549,071	C	T	0.969	0.993	ITGB5
rs148907027	3	124,549,079	A	AC	0.986	0.923	ITGB5
3:124549835_GAAC_G	3	124,549,835	GAAC	G	0.961	0.987	ITGB5
rs79029604	3	124,549,856	A	C	0.776	0.993	ITGB5
rs115255352	3	124,549,926	G	A	0.961	0.994	ITGB5
rs3772840	3	124,550,364	C	T	0.581	0.995	ITGB5
rs17236654	3	124,550,546	G	A	0.961	0.994	ITGB5
rs114045708	3	124,550,580	C	T	0.961	0.994	ITGB5
rs34441446	3	124,551,037	T	TA	0.582	0.99	ITGB5
rs7618074	3	124,551,326	C	T	0.778	0.994	ITGB5
rs4141663	3	124,551,967	C	T	0.582	0.995	ITGB5
rs7635307	3	124,552,512	T	C	0.182	0.998	ITGB5
rs79285968	3	124,553,092	A	C	0.961	0.994	ITGB5
rs531185445	3	124,553,400	C	G	0.978	0.941	ITGB5
rs114950737	3	124,553,573	T	G	0.961	0.994	ITGB5
rs3836315	3	124,554,043	T	TC	0.862	0.996	ITGB5
rs3772841	3	124,554,130	C	A	0.183	0.997	ITGB5
rs5852430	3	124,555,060	T	TA	0.446	0.969	ITGB5
rs75471351	3	124,556,218	G	A	0.961	0.995	ITGB5
rs78954719	3	124,556,380	T	C	0.961	0.995	ITGB5
rs3772842	3	124,556,418	T	C	0.862	0.999	ITGB5
rs73199529	3	124,556,420	G	T	0.977	0.982	ITGB5
rs149641950	3	124,556,498	T	TC	0.961	0.988	ITGB5
rs143478694	3	124,556,535	A	T	0.987	0.984	ITGB5
rs56108819	3	124,557,534	C	T	0.802	0.996	ITGB5
rs6438857	3	124,557,643	T	C	0.582	0.996	ITGB5
rs115242923	3	124,558,304	C	T	0.969	0.995	ITGB5
rs144266243	3	124,558,468	T	A	0.969	0.995	ITGB5
rs3772843	3	124,558,741	G	A	0.823	0.998	ITGB5
3:124559257_GT_G	3	124,559,257	GT	G	0.827	0.985	ITGB5
rs551749365	3	124,559,280	G	GT	0.856	0.95	ITGB5
rs71270408	3	124,559,290	T	TG	0.97	0.983	ITGB5
rs533782703	3	124,559,509	C	CT	0.608	0.961	ITGB5
rs149819282	3	124,559,598	G	A	0.987	0.985	ITGB5
rs3772844	3	124,559,660	G	A	0.862	1	ITGB5
rs61760572	3	124,559,711	C	T	0.969	0.995	ITGB5
rs55857135	3	124,559,908	T	C	0.909	0.995	ITGB5
rs61760570	3	124,560,505	C	A	0.961	0.995	ITGB5
rs61760569	3	124,560,809	G	T	0.963	0.977	ITGB5
rs61760568	3	124,560,842	C	T	0.985	0.982	ITGB5
rs3772845	3	124,560,890	T	C	0.215	0.997	ITGB5
rs77019735	3	124,561,216	A	T	0.968	0.886	ITGB5
rs6438858	3	124,561,480	C	G	0.823	0.999	ITGB5
rs17309105	3	124,561,481	C	T	0.8	0.997	ITGB5
rs116764374	3	124,561,593	C	A	0.961	0.995	ITGB5
rs58331374	3	124,561,844	C	T	0.8	0.997	ITGB5
rs12631961	3	124,561,848	C	T	0.592	0.996	ITGB5
rs116060150	3	124,561,863	C	T	0.961	0.995	ITGB5
rs3772847	3	124,562,515	T	C	0.823	0.999	ITGB5
rs17469	3	124,562,919	C	A	0.969	0.995	ITGB5
rs10934692	3	124,563,075	A	G	0.943	0.51	ITGB5
rs139480831	3	124,563,233	G	A	0.961	0.995	ITGB5
rs11712229	3	124,563,274	G	C	0.69	0.994	ITGB5
rs115854654	3	124,565,495	C	T	0.969	0.994	ITGB5
rs9843012	3	124,565,538	T	C	0.862	0.999	ITGB5
rs9865359	3	124,566,227	A	G	0.822	0.998	ITGB5
rs192404871	3	124,566,683	A	T	0.982	0.944	ITGB5
rs17309224	3	124,566,804	G	T	0.801	0.996	ITGB5
rs61757101	3	124,567,347	C	T	0.989	0.938	ITGB5
3:124567638_AT_A	3	124,567,638	AT	A	0.216	0.993	ITGB5
rs3772848	3	124,568,226	A	G	0.862	0.998	ITGB5
rs76833369	3	124,568,286	A	C	0.801	0.997	ITGB5
rs848807	3	124,568,811	C	T	0.217	0.998	ITGB5
rs3772850	3	124,568,967	C	G	0.823	0.998	ITGB5
3:124569558_CA_C	3	124,569,558	CA	C	0.416	0.993	ITGB5
rs1499965	3	124,569,949	G	A	0.862	0.999	ITGB5
rs9289237	3	124,570,455	A	AAC	0.371	0.884	ITGB5
rs545775054	3	124,570,490	G	A	0.728	0.725	ITGB5
rs3772851	3	124,570,818	T	G	0.595	0.998	ITGB5
rs3772852	3	124,570,959	C	G	0.595	0.998	ITGB5
rs56322733	3	124,571,011	G	A	0.937	0.949	ITGB5
rs13322493	3	124,571,469	G	T	0.862	0.999	ITGB5
rs3772853	3	124,572,219	G	A	0.596	0.998	ITGB5
rs13074444	3	124,572,562	C	A	0.596	0.998	ITGB5
rs10212596	3	124,572,615	T	C	0.823	0.998	ITGB5
rs1265649	3	124,572,650	A	G	0.217	0.998	ITGB5
rs1265650	3	124,572,999	G	A	0.217	0.998	ITGB5
rs1265651	3	124,573,405	T	C	0.799	1	ITGB5
rs114231678	3	124,573,502	T	C	0.969	0.995	ITGB5
rs10934693	3	124,573,826	G	C	0.595	0.998	ITGB5
rs112570685	3	124,573,885	C	A	0.987	0.983	ITGB5
rs848790	3	124,573,898	A	G	0.823	0.998	ITGB5
rs9289238	3	124,573,989	G	A	0.823	0.998	ITGB5
rs72974523	3	124,574,177	T	G	0.961	0.996	ITGB5
rs848792	3	124,574,342	T	C	0.799	1	ITGB5
rs848793	3	124,574,719	C	T	0.217	0.998	ITGB5
rs9809420	3	124,574,861	A	G	0.823	0.998	ITGB5
rs848794	3	124,575,061	A	G	0.217	0.998	ITGB5
rs3772856	3	124,575,410	C	T	0.823	0.998	ITGB5
rs1499961	3	124,575,643	C	G	0.594	0.998	ITGB5
rs848795	3	124,576,282	G	A	0.8	0.999	ITGB5
rs848796	3	124,576,355	T	C	0.8	0.999	ITGB5
rs35994893	3	124,576,743	G	A	0.989	0.912	ITGB5
rs13071338	3	124,576,929	C	G	0.823	0.998	ITGB5
3:124576980_CA_C	3	124,576,980	CA	C	0.831	0.806	ITGB5
rs12695447	3	124,577,077	T	G	0.845	0.999	ITGB5
rs138428144	3	124,577,126	C	T	0.985	0.994	ITGB5
rs11928547	3	124,577,290	T	C	0.593	0.998	ITGB5
rs9858709	3	124,577,464	C	T	0.823	0.998	ITGB5
rs9878701	3	124,577,525	T	C	0.823	0.998	ITGB5
rs11928651	3	124,577,555	T	C	0.594	0.998	ITGB5
rs848797	3	124,577,671	T	C	0.217	0.998	ITGB5
rs9859090	3	124,577,746	C	T	0.823	0.998	ITGB5
rs6778643	3	124,577,826	C	T	0.594	0.998	ITGB5
rs145927866	3	124,578,643	T	C	0.976	0.981	ITGB5
rs848798	3	124,578,895	G	C	0.799	1	ITGB5
rs13089681	3	124,580,009	C	T	0.823	0.998	ITGB5
rs542277325	3	124,580,346	C	T	0.977	0.758	ITGB5
rs848800	3	124,580,376	G	C	0.272	0.91	ITGB5
3:124580411_AT_A	3	124,580,411	AT	A	0.4	0.975	ITGB5
rs142604803	3	124,580,539	G	A	0.96	0.914	ITGB5
rs3772860	3	124,580,977	G	A	0.823	0.998	ITGB5
rs6787638	3	124,582,319	T	C	0.823	0.998	ITGB5
rs10934694	3	124,582,560	T	A	0.823	0.998	ITGB5
3:124582816_TAAAAAAAGAA_T	3	124,582,816	TAAAAAAAGAA	T	0.926	0.82	ITGB5
rs848801	3	124,583,117	T	C	0.217	0.998	ITGB5
rs11706982	3	124,583,139	G	C	0.823	0.998	ITGB5
rs11707014	3	124,583,183	G	A	0.845	0.999	ITGB5
rs848802	3	124,583,393	T	C	0.217	0.998	ITGB5
rs142360922	3	124,583,485	G	T	0.985	0.994	ITGB5
rs9815349	3	124,583,553	C	G	0.823	0.998	ITGB5
rs3772861	3	124,583,789	C	T	0.823	0.998	ITGB5
rs74553200	3	124,584,318	C	T	0.823	0.996	ITGB5
rs187872156	3	124,584,398	A	T	0.966	0.965	ITGB5
rs10934695	3	124,584,719	C	T	0.823	0.998	ITGB5
rs4234222	3	124,584,787	G	A	0.823	0.998	ITGB5
rs114915840	3	124,584,968	G	A	0.978	0.995	ITGB5
rs1846612	3	124,585,077	G	A	0.823	0.998	ITGB5
rs1499966	3	124,585,214	G	A	0.823	0.998	ITGB5
rs848803	3	124,586,308	T	C	0.217	0.998	ITGB5
rs3772864	3	124,587,069	T	C	0.823	0.998	ITGB5
rs113333456	3	124,587,139	G	GGGCTGAGAAGCT	0.826	0.987	ITGB5
rs9813129	3	124,587,917	G	T	0.595	0.998	ITGB5
rs848804	3	124,588,201	T	G	0.799	0.999	ITGB5
rs13091878	3	124,588,929	T	G	0.823	0.998	ITGB5
rs2127304	3	124,589,229	A	G	0.823	0.998	ITGB5

SNP	Chromosome	Position	Effect Allele	Other Allele	Effect Allele Frequency	Imputation quality	Gene
rs3772865	3	124,589,284	C	A	0.595	0.999	ITGB5
rs2169971	3	124,589,518	A	C	0.823	0.998	ITGB5
rs2169972	3	124,589,524	C	G	0.823	0.998	ITGB5
rs201695913	3	124,589,577	G	A	0.64	0.72	ITGB5
rs35271949	3	124,589,827	C	A	0.823	0.998	ITGB5
rs1265643	3	124,589,949	A	G	0.217	0.998	ITGB5
rs1265644	3	124,590,073	G	A	0.792	0.992	ITGB5
rs139771241	3	124,590,195	C	T	0.985	0.994	ITGB5
rs6802275	3	124,590,334	C	G	0.593	0.999	ITGB5
rs6777866	3	124,590,375	A	C	0.601	0.971	ITGB5
rs9824614	3	124,590,376	G	A	0.601	0.97	ITGB5
rs142662785	3	124,590,490	T	G	0.982	0.86	ITGB5
rs1270666	3	124,590,515	A	T	0.217	0.998	ITGB5
3:124590701_TAATA_T	3	124,590,701	TAATA	T	0.816	0.981	ITGB5
rs7632893	3	124,591,419	G	A	0.822	0.997	ITGB5
rs111484769	3	124,591,879	A	AT	0.823	0.996	ITGB5
rs1017813	3	124,591,985	T	C	0.823	0.998	ITGB5
rs1265645	3	124,592,017	T	C	0.801	0.998	ITGB5
rs1017814	3	124,592,139	G	C	0.823	0.998	ITGB5
rs1265646	3	124,592,700	A	G	0.394	0.998	ITGB5
3:124592719_AAAAAG_A	3	124,592,719	AAAAAG	A	0.802	0.994	ITGB5
rs9835619	3	124,592,724	G	A	0.82	0.98	ITGB5
rs62265645	3	124,592,812	T	C	0.858	0.97	ITGB5
rs1265647	3	124,592,838	C	T	0.217	0.998	ITGB5
rs114312729	3	124,592,901	T	C	0.912	0.95	ITGB5
3:124592910_TA_T	3	124,592,910	TA	T	0.183	0.901	ITGB5
rs7373991	3	124,593,137	C	G	0.823	0.998	ITGB5
rs542948451	3	124,593,170	A	G	0.983	0.604	ITGB5
rs2650070	3	124,593,671	T	C	0.217	0.998	ITGB5
rs12491559	3	124,594,061	G	A	0.845	0.998	ITGB5
rs2221149	3	124,594,590	C	G	0.2	0.998	ITGB5
rs2047574	3	124,594,803	G	A	0.2	0.998	ITGB5
rs1948696	3	124,594,955	T	C	0.376	0.998	ITGB5
rs62265647	3	124,595,058	T	C	0.783	0.998	ITGB5
rs62265648	3	124,595,339	G	C	0.783	0.998	ITGB5
rs12631064	3	124,595,673	G	C	0.199	0.998	ITGB5
3:124595774_CTCAG_C	3	124,595,774	CTCAG	C	0.785	0.989	ITGB5
rs73199545	3	124,595,778	G	C	0.946	0.59	ITGB5
3:124595782_GTTTTC_G	3	124,595,782	GTTTTC	G	0.978	0.99	ITGB5
rs565297539	3	124,595,818	G	GT	0.782	0.993	ITGB5
rs11717274	3	124,595,876	A	G	0.823	0.998	ITGB5
rs527544621	3	124,595,984	A	ATT	0.625	0.929	ITGB5
rs142834501	3	124,596,038	C	T	0.978	0.994	ITGB5
rs62265657	3	124,596,424	A	C	0.783	0.998	ITGB5
rs1499959	3	124,596,640	T	C	0.199	0.998	ITGB5
rs4502626	3	124,596,652	A	G	0.783	0.998	ITGB5
3:124596698_AT_A	3	124,596,698	AT	A	0.592	0.989	ITGB5
rs1499960	3	124,596,990	T	C	0.199	0.998	ITGB5
rs9870507	3	124,597,223	A	C	0.971	0.957	ITGB5
rs57747981	3	124,597,413	T	TG	0.589	0.997	ITGB5
rs116279017	3	124,597,447	T	C	0.978	0.995	ITGB5
rs9875516	3	124,598,026	A	T	0.593	0.999	ITGB5
rs10804563	3	124,598,499	T	C	0.689	0.991	ITGB5
rs10804564	3	124,598,526	A	G	0.593	0.998	ITGB5
rs62265658	3	124,599,401	C	T	0.783	0.997	ITGB5
rs55996060	3	124,599,916	G	A	0.799	0.997	ITGB5
rs117047974	3	124,600,416	T	C	0.987	0.979	ITGB5
rs138016814	3	124,600,652	T	TA	0.783	0.996	ITGB5
rs9812212	3	124,600,794	A	G	0.2	0.998	ITGB5
rs9968182	3	124,601,972	C	T	0.594	0.999	ITGB5
rs2173634	3	124,602,072	T	G	0.199	0.998	ITGB5
rs13323528	3	124,602,584	G	A	0.596	0.999	ITGB5
rs1007611	3	124,602,723	A	G	0.862	0.998	ITGB5
rs4422355	3	124,602,882	G	C	0.596	0.999	ITGB5
rs2047571	3	124,603,018	T	C	0.596	0.999	ITGB5
3:124603058_CAA_C	3	124,603,058	CAA	C	0.596	0.998	ITGB5
rs373808875	3	124,603,065	A	T	0.598	0.995	ITGB5
rs200034715	3	124,603,071	GA	G	0.596	0.998	ITGB5
rs2047573	3	124,603,127	G	C	0.596	0.999	ITGB5
rs12488397	3	124,603,247	A	G	0.862	0.998	ITGB5
rs13079779	3	124,603,287	C	T	0.596	0.999	ITGB5
rs13079799	3	124,603,318	C	A	0.596	0.999	ITGB5
rs13081005	3	124,603,759	G	A	0.596	0.999	ITGB5
rs35900531	3	124,603,863	A	T	0.596	0.999	ITGB5
rs36071060	3	124,603,947	A	G	0.595	0.999	ITGB5
rs1007857	3	124,604,689	C	A	0.862	0.998	ITGB5
rs577608856	3	124,605,223	G	A	0.988	0.994	ITGB5
rs6438860	3	124,605,531	C	G	0.217	0.998	ITGB5
rs6438861	3	124,605,736	T	C	0.217	0.997	ITGB5
rs61760562	3	124,606,509	G	A	0.96	0.995	ITGB5
rs4679113	3	124,606,753	T	A	0.363	0.997	ITGB5
rs1499963	3	124,607,055	T	C	0.321	0.997	ITGB5
rs7430164	3	124,607,760	G	A	0.34	0.997	ITGB5
rs10934700	3	124,608,589	G	A	0.862	0.997	ITGB5
rs79606234	3	124,608,656	T	C	0.968	0.994	ITGB5
3:124609047_CTT_C	3	124,609,047	CTT	C	0.221	0.987	ITGB5
rs12633721	3	124,609,338	G	A	0.597	0.997	ITGB5
rs9847614	3	124,609,945	G	A	0.862	0.997	ITGB5
rs7614708	3	124,609,966	A	G	0.199	0.997	ITGB5
rs7373878	3	124,610,238	G	T	0.378	0.997	ITGB5
rs140191827	3	124,610,420	G	A	0.96	0.995	ITGB5
rs755883370	3	124,610,440	CA	C	0.939	0.747	ITGB5
rs59000678	3	124,611,096	T	C	0.32	0.996	ITGB5
rs770474354	3	124,611,538	TA	T	0.531	0.856	ITGB5
3:124611614_CA_C	3	124,611,614	CA	C	0.784	0.99	ITGB5
rs144099275	3	124,612,060	G	A	0.963	0.969	ITGB5
rs76603866	3	124,612,378	A	T	0.968	0.995	ITGB5
rs6783734	3	124,612,539	C	G	0.183	0.995	ITGB5
rs189588713	3	124,613,045	T	C	0.961	0.995	ITGB5
rs6784520	3	124,613,271	C	A	0.585	0.995	ITGB5
rs62267663	3	124,613,390	G	T	0.783	0.997	ITGB5
rs62267664	3	124,613,635	T	A	0.783	0.997	ITGB5
rs9864770	3	124,613,752	G	A	0.857	0.995	ITGB5
rs62267665	3	124,614,164	T	C	0.743	0.997	ITGB5
rs9868969	3	124,614,214	C	T	0.856	0.995	ITGB5
rs9869147	3	124,614,317	C	T	0.602	0.996	ITGB5
rs10804565	3	124,615,364	A	G	0.184	0.995	ITGB5
rs547608481	3	124,615,593	T	TAA	0.549	0.978	ITGB5
rs572398071	3	124,615,610	A	T	0.987	0.956	ITGB5
rs556523188	3	124,615,763	C	CA	0.734	0.941	ITGB5
rs375431006	3	124,615,778	T	A	0.963	0.894	ITGB5
rs72974592	3	124,615,950	C	T	0.96	0.996	ITGB5
rs6810148	3	124,616,516	T	A	0.583	0.995	ITGB5
rs188282921	3	124,616,517	T	A	0.963	0.908	ITGB5
rs1909584	3	124,617,275	C	G	0.198	0.992	ITGB5
rs560796244	3	124,617,490	C	CAAAAAA	0.547	0.955	ITGB5
rs146169609	3	124,617,738	C	T	0.96	0.996	ITGB5
3:124617922_CAAAAAAAAAAGAAA_C	3	124,617,922	CAAAAAAAAAAGAAA	C	0.794	0.951	ITGB5
3:124617924_AAAAAAAAAGAAAAG_A	3	124,617,924	AAAAAAAAAGAAAAG	A	0.789	0.963	ITGB5
rs113177082	3	124,618,140	T	G	0.977	0.924	ITGB5
rs190214952	3	124,618,155	G	A	0.986	0.842	ITGB5
rs148467120	3	124,618,777	G	GGA	0.96	0.99	ITGB5
rs144024253	3	124,618,795	C	T	0.988	0.992	ITGB5
rs9829173	3	124,618,868	A	T	0.199	0.995	ITGB5
rs62267667	3	124,618,915	C	T	0.742	0.997	ITGB5
rs76131088	3	124,619,280	T	G	0.961	0.995	ITGB5
rs62267668	3	124,619,483	C	T	0.742	0.997	ITGB5
rs72974597	3	124,619,513	C	A	0.961	0.994	ITGB5
rs75057516	3	124,619,704	C	T	0.961	0.995	ITGB5
rs115036636	3	124,619,850	C	T	0.961	0.995	ITGB5
rs7374999	3	124,619,998	C	T	0.782	0.997	ITGB5
rs17880408	4	1,795,076	G	C	0.976	0.915	FGFR3
rs4450996	4	1,795,321	C	A	0.278	0.975	FGFR3
rs17879199	4	1,795,333	C	T	0.829	0.977	FGFR3
rs3135835	4	1,796,180	G	A	0.216	0.98	FGFR3
rs3135836	4	1,796,197	G	A	0.985	0.996	FGFR3
rs3135838	4	1,796,310	C	T	0.984	0.997	FGFR3
rs17881768	4	1,796,432	C	G	0.831	0.99	FGFR3
rs3135840	4	1,796,539	A	T	0.733	0.991	FGFR3
rs3135841	4	1,796,629	C	T	0.907	0.989	FGFR3
rs3135842	4	1,796,636	G	C	0.733	0.992	FGFR3
rs3135845	4	1,797,358	G	A	0.829	0.996	FGFR3
rs3135846	4	1,797,484	C	G	0.732	0.998	FGFR3
rs743682	4	1,797,852	G	A	0.907	0.994	FGFR3
rs3135850	4	1,798,176	C	T	0.988	0.899	FGFR3
rs3135853	4	1,798,263	C	G	0.984	0.993	FGFR3
rs62286988	4	1,798,737	G	C	0.985	0.895	FGFR3
rs3135857	4	1,799,119	G	A	0.98	0.949	FGFR3
rs3135859	4	1,799,490	G	T	0.829	0.979	FGFR3
rs3135863	4	1,800,201	G	A	0.973	0.722	FGFR3
4:1800363_CTCCT_C	4	1,800,363	CTCCT	C	0.961	0.931	FGFR3
rs2301293	4	1,801,866	C	T	0.872	0.994	FGFR3
rs3135871	4	1,802,211	T	C	0.871	0.995	FGFR3
rs2305183	4	1,803,307	T	C	0.863	0.998	FGFR3
rs2234909	4	1,803,704	T	C	0.863	0.998	FGFR3

SNP	Chromosome	Position	Effect Allele	Other Allele	Effect Allele Frequency	Imputation quality	Gene
rs2305184	4	1,803,824	C	G	0.863	0.998	FGFR3
rs3135875	4	1,803,970	G	A	0.864	0.998	FGFR3
rs3135877	4	1,804,276	G	A	0.958	0.943	FGFR3
rs3135878	4	1,804,377	G	A	0.209	0.979	FGFR3
rs3135886	4	1,805,593	G	C	0.942	0.951	FGFR3
rs750031483	4	1,806,012	TG	T	0.619	0.74	FGFR3
rs3135890	4	1,806,519	C	T	0.886	0.943	FGFR3
rs3135898	4	1,807,922	G	A	0.986	0.902	FGFR3
rs3135899	4	1,808,523	G	A	0.983	0.956	FGFR3
rs3135900	4	1,809,025	A	G	0.967	0.897	FGFR3
4:1809192_CAG_C	4	1,809,192	CAG	C	0.989	0.874	FGFR3
rs115528417	4	55,944,891	T	C	0.98	0.993	KDR
4:55945822_TA_T	4	55,945,822	TA	T	0.965	0.996	KDR
rs35961234	4	55,946,171	G	A	0.97	0.94	KDR
rs10006115	4	55,946,354	G	T	0.965	0.999	KDR
rs4864950	4	55,946,902	T	A	0.817	0.995	KDR
rs4864951	4	55,947,155	A	C	0.817	0.995	KDR
rs78146111	4	55,947,272	A	G	0.965	0.997	KDR
rs4864952	4	55,947,338	T	C	0.817	0.994	KDR
rs150001331	4	55,948,068	G	T	0.988	0.822	KDR
rs10434441	4	55,950,161	C	T	0.965	0.932	KDR
rs10434442	4	55,950,162	A	G	0.816	0.927	KDR
rs12498529	4	55,950,198	A	T	0.817	0.927	KDR
rs28387674	4	55,951,300	T	C	0.966	0.919	KDR
rs28478981	4	55,951,728	G	T	0.966	0.916	KDR
rs76360687	4	55,951,823	A	G	0.976	0.841	KDR
rs28690664	4	55,951,868	T	C	0.96	0.91	KDR
rs12642334	4	55,952,299	T	A	0.66	0.903	KDR
rs143100790	4	55,952,454	A	G	0.979	0.989	KDR
rs17085257	4	55,953,330	C	T	0.654	0.96	KDR
rs28633455	4	55,953,380	A	G	0.649	0.958	KDR
rs2125489	4	55,953,483	G	A	0.885	0.964	KDR
rs75860138	4	55,954,379	C	T	0.977	0.923	KDR
rs1458819	4	55,954,882	T	C	0.886	0.979	KDR
rs1531289	4	55,955,232	T	C	0.293	0.974	KDR
rs3217703	4	55,955,737	A	AT	0.651	0.96	KDR
rs11940163	4	55,957,315	T	C	0.643	0.996	KDR
rs13127286	4	55,957,316	G	A	0.76	0.994	KDR
rs17709898	4	55,957,723	A	G	0.643	0.995	KDR
rs1458830	4	55,958,002	A	G	0.643	0.995	KDR
rs1458831	4	55,958,208	T	C	0.886	0.988	KDR
rs77527870	4	55,958,240	A	G	0.885	0.945	KDR
rs17085262	4	55,959,141	C	T	0.76	0.994	KDR
rs17085265	4	55,959,152	A	G	0.761	0.994	KDR
rs17085267	4	55,959,862	C	T	0.761	0.995	KDR
rs35624269	4	55,960,095	G	A	0.758	0.993	KDR
rs35399838	4	55,960,142	G	A	0.758	0.993	KDR
rs9799762	4	55,960,179	C	T	0.758	0.993	KDR
rs6811269	4	55,960,205	A	G	0.759	0.985	KDR
4:55960212_CA_C	4	55,960,212	CA	C	0.764	0.932	KDR
rs4864953	4	55,960,244	A	C	0.758	0.993	KDR
rs4864532	4	55,960,404	A	C	0.752	0.991	KDR
rs2219471	4	55,961,159	T	C	0.758	0.994	KDR
rs73234300	4	55,962,165	A	T	0.653	0.987	KDR
rs41279531	4	55,962,166	C	T	0.653	0.987	KDR
rs6838752	4	55,964,101	T	C	0.761	0.999	KDR
rs2305946	4	55,964,605	C	T	0.761	1	KDR
4:55965031_AT_A	4	55,965,031	AT	A	0.988	0.864	KDR
rs58415820	4	55,965,434	G	A	0.759	0.995	KDR
rs1870379	4	55,966,304	A	C	0.761	0.995	KDR
rs1870378	4	55,966,453	C	T	0.761	0.995	KDR
rs116670019	4	55,966,573	C	T	0.987	0.885	KDR
rs6815929	4	55,967,178	T	A	0.649	0.998	KDR
rs2168945	4	55,967,483	A	C	0.649	0.998	KDR
rs7677779	4	55,967,684	C	T	0.764	0.998	KDR
rs1968770	4	55,967,806	A	G	0.649	0.998	KDR
rs7655964	4	55,968,053	A	C	0.649	0.998	KDR
rs189677709	4	55,968,807	C	G	0.99	0.897	KDR
rs62304711	4	55,968,820	G	A	0.982	0.927	KDR
rs13136007	4	55,969,006	C	A	0.76	0.998	KDR
rs6848933	4	55,969,711	C	G	0.649	0.999	KDR
rs11732292	4	55,970,101	A	C	0.649	0.999	KDR
rs10016064	4	55,970,205	G	C	0.752	0.995	KDR
rs34176444	4	55,971,400	G	A	0.65	1	KDR
rs2305945	4	55,971,846	G	T	0.65	0.998	KDR
rs139652678	4	55,973,511	C	G	0.982	0.923	KDR
rs6846151	4	55,974,290	C	G	0.409	0.987	KDR
rs555113166	4	55,974,365	C	CAA	0.681	0.934	KDR
rs12507807	4	55,974,536	A	G	0.648	0.985	KDR
4:55974862_TCCTC_T	4	55,974,862	TCCTC	T	0.431	0.951	KDR
rs561722074	4	55,975,258	C	CT	0.406	0.958	KDR
rs7673274	4	55,975,719	A	G	0.405	0.973	KDR
rs7654599	4	55,976,168	C	T	0.413	0.969	KDR
rs3828550	4	55,976,451	G	A	0.665	0.955	KDR
rs17085326	4	55,977,376	C	T	0.92	0.943	KDR
rs3943404	4	55,977,759	A	T	0.56	0.912	KDR
rs2034965	4	55,977,800	A	G	0.258	0.926	KDR
rs2034966	4	55,977,866	A	T	0.78	0.915	KDR
rs2034967	4	55,977,961	C	T	0.605	0.923	KDR
rs36151314	4	55,978,629	G	A	0.667	0.909	KDR
rs766201920	4	55,978,690	ATTT	A	0.527	0.864	KDR
rs10020464	4	55,979,070	C	T	0.708	0.965	KDR
rs7692791	4	55,980,239	C	T	0.464	0.959	KDR
rs2305949	4	55,980,456	C	T	0.786	0.966	KDR
rs754709937	4	55,982,584	CAA	C	0.817	0.99	KDR
rs34420560	4	55,983,179	G	GC	0.853	0.995	KDR
rs78590050	4	55,983,235	T	C	0.815	0.974	KDR
rs78508765	4	55,983,376	C	T	0.82	0.974	KDR
rs1962460	4	55,985,142	A	G	0.447	0.917	KDR
rs1962459	4	55,985,149	A	G	0.592	0.916	KDR
rs1962458	4	55,985,209	C	G	0.805	0.914	KDR
rs140832758	4	55,985,262	G	C	0.977	0.893	KDR
rs111434117	4	55,985,373	C	CA	0.819	0.857	KDR
rs6837735	4	55,985,815	C	T	0.821	0.895	KDR
rs2054246	4	55,987,002	G	T	0.184	0.934	KDR
rs116245354	4	55,987,778	G	A	0.983	0.917	KDR
rs80018124	4	55,987,886	G	A	0.939	0.979	KDR
rs115400416	4	55,987,920	C	T	0.978	0.963	KDR
rs77317629	4	55,987,943	T	A	0.834	0.957	KDR
4:55988082_CT_C	4	55,988,082	CT	C	0.708	0.942	KDR
rs1380057	4	55,989,675	T	A	0.504	0.969	KDR
rs140213451	4	55,990,938	G	A	0.978	0.993	KDR
rs73236104	4	55,990,954	G	C	0.744	0.984	KDR
rs12502008	4	55,991,042	G	T	0.638	0.968	KDR
rs56233104	4	55,991,461	C	A	0.938	0.995	KDR
rs7667298	4	55,991,731	T	C	0.464	0.989	KDR
rs12507427	4	145,567,471	T	A	0.573	0.999	HHIP
rs147015513	4	145,568,844	T	TTAAC	0.573	0.997	HHIP
4:145569140_TA_T	4	145,569,140	TA	T	0.566	0.984	HHIP
rs148367024	4	145,569,602	T	C	0.989	0.919	HHIP
rs13146972	4	145,569,692	C	T	0.573	0.999	HHIP
rs34147694	4	145,570,387	G	GA	0.618	0.985	HHIP
rs13136840	4	145,571,694	A	C	0.573	0.999	HHIP
rs7675744	4	145,572,046	T	C	0.573	0.999	HHIP
4:145572396_TAA_T	4	145,572,396	TAA	T	0.984	0.977	HHIP
rs7676399	4	145,572,601	A	T	0.573	0.999	HHIP
rs11464673	4	145,572,913	A	AT	0.573	0.998	HHIP
rs751262600	4	145,573,954	GA	G	0.613	0.998	HHIP
rs1844428	4	145,574,196	A	G	0.867	0.999	HHIP
rs1489758	4	145,574,239	G	T	0.613	0.999	HHIP
rs13150962	4	145,575,179	C	G	0.986	0.991	HHIP
rs2220514	4	145,576,558	G	A	0.567	0.999	HHIP
rs199898383	4	145,576,904	A	ACTCTT	0.514	0.998	HHIP
rs2035742	4	145,577,370	A	G	0.964	0.999	HHIP
rs2035900	4	145,577,636	T	C	0.513	0.999	HHIP
4:145577701_AAC_A	4	145,577,701	AAC	A	0.968	0.923	HHIP
rs62346130	4	145,578,799	C	T	0.567	1	HHIP
rs139338114	4	145,579,503	G	GTATT	0.924	0.823	HHIP
rs7680782	4	145,579,590	T	C	0.513	0.999	HHIP
rs6537301	4	145,579,907	G	A	0.513	0.999	HHIP
rs114633728	4	145,580,172	T	C	0.957	0.965	HHIP
rs1355601	4	145,580,290	C	A	0.567	0.999	HHIP
rs1355602	4	145,580,585	A	C	0.513	0.999	HHIP
rs78905966	4	145,581,458	C	A	0.933	0.985	HHIP
rs1387631	4	145,581,596	T	C	0.513	0.999	HHIP
rs60684117	4	145,581,994	A	T	0.983	0.997	HHIP
rs2306924	4	145,582,536	A	G	0.513	0.999	HHIP
rs13145247	4	145,582,820	T	C	0.513	0.999	HHIP
rs58797593	4	145,584,175	T	G	0.964	1	HHIP
rs7687599	4	145,584,207	T	G	0.567	0.999	HHIP
rs6852346	4	145,585,354	A	T	0.567	0.998	HHIP
rs6825848	4	145,585,521	G	A	0.567	0.998	HHIP
rs12501043	4	145,586,043	T	C	0.567	0.998	HHIP
rs982903	4	145,586,459	A	G	0.53	0.998	HHIP
rs982902	4	145,586,900	G	A	0.567	0.998	HHIP

SNP	Chromosome	Position	Effect Allele	Other Allele	Effect Allele Frequency	Imputation quality	Gene
rs6537302	4	145,588,116	A	T	0.567	0.998	HHIP
rs57498151	4	145,588,905	T	A	0.964	1	HHIP
rs141986880	4	145,589,253	G	T	0.987	0.985	HHIP
rs6537303	4	145,589,844	C	A	0.567	0.998	HHIP
rs6537304	4	145,590,005	C	T	0.514	0.997	HHIP
rs4834989	4	145,590,445	A	T	0.983	0.996	HHIP
rs7340949	4	145,590,933	A	G	0.514	0.997	HHIP
4:145590957_AG_A	4	145,590,957	AG	A	0.566	0.993	HHIP
rs34701002	4	145,590,958	G	A	0.936	0.559	HHIP
rs116052672	4	145,590,959	T	A	0.566	0.993	HHIP
rs6537305	4	145,591,061	A	G	0.567	0.997	HHIP
rs147496190	4	145,591,130	T	TA	0.303	0.81	HHIP
rs6537306	4	145,591,211	G	A	0.567	0.997	HHIP
rs61086348	4	145,591,258	A	G	0.983	0.996	HHIP
rs558591689	4	145,591,294	C	CA	0.578	0.97	HHIP
rs6832061	4	145,591,426	C	A	0.567	0.997	HHIP
rs371047948	4	145,591,607	C	CA	0.838	0.808	HHIP
rs540536105	4	145,591,859	G	GA	0.95	0.807	HHIP
4:145592470_GT_G	4	145,592,470	GT	G	0.514	0.976	HHIP
rs1844429	4	145,593,028	T	C	0.568	0.997	HHIP
rs139559529	4	145,593,082	G	T	0.964	1	HHIP
rs11938839	4	145,593,674	G	C	0.569	0.996	HHIP
rs58034003	4	145,593,953	A	G	0.983	0.997	HHIP
rs57740751	4	145,595,760	C	T	0.987	0.958	HHIP
rs71614518	4	145,596,885	A	C	0.969	0.989	HHIP
rs2055059	4	145,597,759	A	G	0.419	0.994	HHIP
rs6826476	4	145,598,315	C	T	0.705	0.993	HHIP
rs11946517	4	145,599,534	G	C	0.42	0.994	HHIP
rs2131354	4	145,599,908	G	A	0.473	0.994	HHIP
rs138169526	4	145,600,080	G	A	0.969	0.987	HHIP
rs140678451	4	145,600,128	A	G	0.964	0.997	HHIP
rs77243209	4	145,600,375	G	A	0.984	0.994	HHIP
rs6537307	4	145,601,863	A	G	0.507	0.995	HHIP
rs2639583	4	145,602,497	T	C	0.864	0.993	HHIP
rs372547705	4	145,602,573	CTT	C	0.505	0.807	HHIP
rs72948477	4	145,602,768	G	T	0.963	1	HHIP
rs7665268	4	145,602,827	G	A	0.507	0.996	HHIP
rs6537308	4	145,604,853	G	T	0.438	0.999	HHIP
rs2575580	4	145,605,025	A	G	0.865	0.994	HHIP
rs6819412	4	145,605,033	T	C	0.507	0.998	HHIP
rs6537309	4	145,605,075	T	C	0.438	0.999	HHIP
rs141349721	4	145,605,213	T	TA	0.508	0.992	HHIP
rs6819982	4	145,605,273	T	C	0.438	1	HHIP
rs151049842	4	145,605,720	C	T	0.964	1	HHIP
rs7699480	4	145,607,846	C	T	0.439	0.999	HHIP
rs7698158	4	145,607,902	G	A	0.439	0.999	HHIP
4:145608903_CTT_C	4	145,608,903	CTT	C	0.558	0.863	HHIP
rs762527406	4	145,611,936	CTA	C	0.476	0.998	HHIP
rs150177892	4	145,612,344	A	T	0.988	0.908	HHIP
rs12507436	4	145,612,551	C	A	0.478	0.994	HHIP
rs12511407	4	145,612,552	A	T	0.481	0.979	HHIP
4:145613048_GA_G	4	145,613,048	GA	G	0.965	0.963	HHIP
rs13146352	4	145,613,551	A	G	0.507	0.998	HHIP
rs6812830	4	145,613,807	G	A	0.476	0.999	HHIP
rs547330127	4	145,614,043	A	AGAG	0.439	0.997	HHIP
rs13131837	4	145,614,984	G	A	0.508	0.998	HHIP
rs62343968	4	145,615,586	T	C	0.968	0.991	HHIP
rs140762847	4	145,616,955	C	T	0.964	0.999	HHIP
rs7439494	4	145,617,748	G	A	0.441	1	HHIP
rs1907867	4	145,617,946	C	T	0.441	1	HHIP
rs17721701	4	145,618,719	T	C	0.443	0.997	HHIP
rs34958276	4	145,620,554	G	A	0.442	0.998	HHIP
rs75686861	4	145,621,328	G	A	0.908	0.986	HHIP
rs113084611	4	145,622,200	G	A	0.956	0.986	HHIP
rs556685383	4	145,622,474	T	C	0.982	0.635	HHIP
rs766855170	4	145,622,475	C	CT	0.445	0.985	HHIP
rs12502033	4	145,622,651	T	C	0.442	0.997	HHIP
rs6838704	4	145,623,107	G	A	0.439	0.997	HHIP
rs6822018	4	145,623,161	T	C	0.439	0.997	HHIP
rs568298987	4	145,624,366	C	CA	0.476	0.969	HHIP
rs7654947	4	145,625,730	C	T	0.429	0.997	HHIP
rs6812389	4	145,626,096	C	G	0.5	0.997	HHIP
rs139899712	4	145,626,375	C	T	0.986	0.973	HHIP
rs6817771	4	145,626,414	C	G	0.429	0.997	HHIP
rs6817556	4	145,627,012	G	A	0.429	0.997	HHIP
rs3733422	4	145,627,397	C	T	0.429	0.996	HHIP
rs3733423	4	145,627,405	G	T	0.429	0.996	HHIP
rs3733424	4	145,627,458	A	G	0.429	0.996	HHIP
rs6148709	4	145,628,798	CA	C	0.392	0.93	HHIP
rs115219631	4	145,628,926	T	C	0.99	0.954	HHIP
rs6846476	4	145,631,648	T	G	0.429	0.997	HHIP
rs111272307	4	145,632,198	T	TCCTA	0.428	0.996	HHIP
rs11100863	4	145,633,530	A	G	0.959	0.98	HHIP
rs71590472	4	145,634,037	G	GGTGT	0.495	0.962	HHIP
rs140568836	4	145,634,402	A	C	0.986	0.976	HHIP
rs6537310	4	145,636,732	T	C	0.428	0.999	HHIP
rs1907866	4	145,637,075	A	T	0.501	0.998	HHIP
rs33915726	4	145,637,449	T	C	0.428	0.999	HHIP
rs773393373	4	145,637,692	CAA	C	0.953	0.915	HHIP
rs140410139	4	145,637,836	C	A	0.959	0.981	HHIP
rs1389029	4	145,638,178	C	T	0.466	0.999	HHIP
rs186720043	4	145,638,728	T	C	0.989	0.909	HHIP
rs6815058	4	145,639,033	G	T	0.428	0.999	HHIP
rs2087826	4	145,639,318	G	A	0.466	0.999	HHIP
rs2276934	4	145,640,345	C	G	0.428	0.999	HHIP
rs78923942	4	145,640,618	C	T	0.976	0.978	HHIP
rs923783	4	145,640,985	A	G	0.428	0.999	HHIP
4:145642746_GA_G	4	145,642,746	GA	G	0.455	0.933	HHIP
rs6855202	4	145,643,326	G	C	0.428	1	HHIP
rs6843518	4	145,643,486	T	C	0.428	1	HHIP
rs62343971	4	145,644,599	A	G	0.983	0.976	HHIP
rs6826051	4	145,645,820	G	A	0.912	0.926	HHIP
rs750836420	4	145,645,847	TAA	T	0.428	0.998	HHIP
rs9759612	4	145,645,849	A	T	0.956	0.538	HHIP
rs1492818	4	145,646,554	G	A	0.428	1	HHIP
rs1845122	4	145,646,815	C	T	0.502	0.996	HHIP
rs923782	4	145,648,032	T	C	0.428	1	HHIP
rs1552792	4	145,648,783	C	T	0.428	1	HHIP
rs2353915	4	145,649,242	C	T	0.428	1	HHIP
rs141566866	4	145,649,621	G	T	0.988	0.907	HHIP
rs34100070	4	145,650,967	C	CA	0.428	0.998	HHIP
rs11100864	4	145,651,004	C	T	0.427	1	HHIP
rs11100865	4	145,651,147	A	G	0.428	1	HHIP
4:145651394_AT_A	4	145,651,394	AT	A	0.428	0.999	HHIP
rs6857302	4	145,651,663	A	G	0.428	0.999	HHIP
4:145651783_TC_T	4	145,651,783	TC	T	0.988	0.925	HHIP
rs6827641	4	145,653,694	T	C	0.466	0.998	HHIP
rs7692915	4	145,654,497	G	T	0.501	0.997	HHIP
rs11100866	4	145,654,913	T	C	0.428	0.998	HHIP
rs78534262	4	145,655,108	A	G	0.962	0.999	HHIP
rs55817790	4	145,655,389	A	T	0.433	0.986	HHIP
rs72718205	4	145,655,491	T	G	0.984	0.875	HHIP
rs1472174	4	145,655,532	T	G	0.428	0.997	HHIP
rs573780512	4	145,655,769	T	TA	0.479	0.956	HHIP
rs11100867	4	145,656,211	T	C	0.428	0.997	HHIP
rs200557409	4	145,656,834	CT	C	0.466	0.995	HHIP
rs35135458	4	145,656,894	C	T	0.981	0.939	HHIP
rs11100868	4	145,657,166	T	C	0.428	0.996	HHIP
rs6826012	4	145,658,286	C	T	0.501	0.995	HHIP
rs2639576	4	145,658,429	T	C	0.53	0.985	HHIP
rs11942655	4	145,661,651	G	C	0.899	0.995	HHIP
rs139706044	4	145,661,968	G	A	0.986	0.974	HHIP
rs7666450	4	145,662,842	C	T	0.557	0.994	HHIP
rs115282885	4	145,663,158	G	A	0.963	0.999	HHIP
rs72718211	4	145,663,798	G	A	0.557	0.994	HHIP
rs17030428	4	154,702,283	G	C	0.958	0.997	SFRP2
rs17030432	4	154,703,460	G	A	0.958	0.998	SFRP2
rs202013289	4	154,704,070	CTA	C	0.291	0.997	SFRP2
rs17030437	4	154,704,225	C	A	0.75	0.999	SFRP2
rs2405202	4	154,705,132	A	G	0.292	0.999	SFRP2
rs2405203	4	154,705,151	G	A	0.487	0.999	SFRP2
rs2405204	4	154,705,248	A	G	0.292	1	SFRP2
rs6853435	4	154,706,016	G	A	0.486	1	SFRP2
rs6853522	4	154,706,220	C	A	0.806	1	SFRP2
rs3039554	4	154,706,728	G	A	0.52	0.917	SFRP2
rs375440750	4	154,708,009	TAA	T	0.95	0.949	SFRP2
rs149325204	4	154,708,071	G	A	0.988	0.916	SFRP2
rs3810765	4	154,709,480	G	A	0.486	0.999	SFRP2
rs5867657	5	44,300,361	T	TTC	0.697	0.978	FGF10
rs3059323	5	44,300,362	T	TCT	0.698	0.974	FGF10
rs1979079	5	44,300,650	A	G	0.798	0.997	FGF10
rs10052683	5	44,300,655	T	C	0.981	0.999	FGF10
rs56107445	5	44,300,952	G	A	0.897	0.998	FGF10
rs11742761	5	44,301,167	A	G	0.83	0.991	FGF10
rs6881989	5	44,301,390	T	A	0.798	0.996	FGF10

SNP	Chromosome	Position	Effect Allele	Other Allele	Effect Allele Frequency	Imputation quality	Gene
5:44301565_AT_A	5	44,301,565	AT	A	0.897	0.996	FGF10
rs13186320	5	44,302,177	A	T	0.493	0.992	FGF10
5:44302380_CCTTG_C	5	44,302,380	CCTTG	C	0.967	0.501	FGF10
5:44302879_AC_A	5	44,302,879	AC	A	0.832	0.99	FGF10
rs1823251	5	44,303,717	C	G	0.799	0.996	FGF10
rs1374961	5	44,303,760	T	C	0.675	0.997	FGF10
rs17228283	5	44,303,769	C	T	0.897	0.998	FGF10
rs3087743	5	44,304,435	C	A	0.832	0.992	FGF10
rs17234646	5	44,304,720	G	A	0.981	0.999	FGF10
rs2290070	5	44,305,309	C	G	0.799	0.996	FGF10
rs6451758	5	44,305,515	T	A	0.798	0.996	FGF10
rs7715401	5	44,305,850	T	C	0.981	0.994	FGF10
5:44306631_GC_G	5	44,306,631	GC	G	0.694	0.993	FGF10
rs17234597	5	44,307,322	G	T	0.799	0.996	FGF10
rs10473352	5	44,308,252	A	G	0.799	0.996	FGF10
rs17234541	5	44,308,898	C	A	0.905	0.989	FGF10
rs17228137	5	44,309,684	C	T	0.793	0.996	FGF10
rs17228130	5	44,309,831	C	T	0.799	0.996	FGF10
5:44310318_TG_T	5	44,310,318	TG	T	0.987	0.957	FGF10
rs6881797	5	44,310,492	A	T	0.793	0.996	FGF10
rs1374962	5	44,311,070	A	G	0.69	0.997	FGF10
rs1448035	5	44,311,768	C	T	0.69	0.996	FGF10
rs17234485	5	44,312,303	C	T	0.897	0.994	FGF10
rs10060796	5	44,313,151	C	T	0.981	0.999	FGF10
rs1839090	5	44,313,282	G	T	0.671	0.997	FGF10
rs1839091	5	44,313,303	T	C	0.329	0.997	FGF10
rs6868853	5	44,313,623	A	G	0.981	0.999	FGF10
rs1816964	5	44,313,650	G	A	0.793	0.995	FGF10
rs576829398	5	44,313,690	G	GT	0.529	0.932	FGF10
rs10473353	5	44,315,108	T	C	0.69	0.997	FGF10
rs17316984	5	44,315,374	C	T	0.832	0.991	FGF10
rs12332431	5	44,315,807	C	A	0.981	0.999	FGF10
rs111506312	5	44,317,208	A	G	0.99	0.933	FGF10
rs980510	5	44,318,532	C	A	0.671	0.998	FGF10
rs13436788	5	44,318,624	A	G	0.981	0.999	FGF10
rs59436891	5	44,319,136	G	T	0.981	0.999	FGF10
rs72763176	5	44,319,330	T	A	0.897	0.998	FGF10
rs78877242	5	44,319,350	G	A	0.989	0.959	FGF10
rs114802370	5	44,319,740	T	G	0.974	0.931	FGF10
rs7704646	5	44,320,141	C	G	0.981	0.999	FGF10
rs72763179	5	44,320,519	T	C	0.897	0.997	FGF10
5:44320814_CT_C	5	44,320,814	CT	C	0.329	0.997	FGF10
rs10452475	5	44,320,968	C	T	0.981	0.999	FGF10
rs10452476	5	44,321,200	T	C	0.981	0.999	FGF10
rs2100155	5	44,321,636	G	A	0.98	0.969	FGF10
rs1816965	5	44,322,310	A	G	0.799	0.997	FGF10
rs72763180	5	44,323,014	C	T	0.897	0.997	FGF10
rs4498258	5	44,325,322	A	T	0.69	0.999	FGF10
rs13159800	5	44,325,469	T	C	0.329	0.998	FGF10
rs4410645	5	44,325,507	C	A	0.671	0.998	FGF10
rs4409099	5	44,325,537	C	T	0.799	0.995	FGF10
rs137972537	5	44,325,607	A	G	0.897	0.997	FGF10
5:44325983_TA_T	5	44,325,983	TA	T	0.671	0.997	FGF10
rs72763181	5	44,326,287	T	C	0.897	0.997	FGF10
rs73089937	5	44,326,604	G	A	0.981	0.992	FGF10
rs75008437	5	44,326,998	G	A	0.799	0.996	FGF10
rs10057630	5	44,327,864	T	C	0.981	0.999	FGF10
rs4866891	5	44,328,270	T	C	0.329	0.999	FGF10
rs13185276	5	44,328,325	T	C	0.329	0.999	FGF10
rs17234338	5	44,328,566	A	T	0.989	0.947	FGF10
rs10055317	5	44,328,627	A	G	0.981	0.999	FGF10
rs10055386	5	44,328,731	A	G	0.671	0.998	FGF10
rs13165847	5	44,328,829	A	C	0.329	0.999	FGF10
rs13170645	5	44,329,203	G	A	0.329	0.999	FGF10
rs6451760	5	44,329,432	C	T	0.671	0.998	FGF10
5:44329660_CT_C	5	44,329,660	CT	C	0.982	0.563	FGF10
rs58307385	5	44,330,857	C	T	0.99	0.988	FGF10
rs13180550	5	44,330,995	A	G	0.329	0.999	FGF10
rs12652957	5	44,331,751	T	A	0.329	0.999	FGF10
rs987642	5	44,331,905	C	T	0.69	0.998	FGF10
rs77429396	5	44,333,205	C	T	0.897	0.997	FGF10
rs67412235	5	44,333,212	C	A	0.69	0.998	FGF10
rs735083	5	44,334,051	A	G	0.793	0.998	FGF10
rs1448043	5	44,334,311	G	C	0.981	1	FGF10
rs13164276	5	44,335,012	C	G	0.989	0.916	FGF10
rs17234268	5	44,335,327	C	G	0.899	0.998	FGF10
rs983373	5	44,335,338	G	A	0.692	0.999	FGF10
rs1011814	5	44,335,820	T	C	0.327	1	FGF10
5:44337364_TA_T	5	44,337,364	TA	T	0.326	0.998	FGF10
rs72763188	5	44,337,690	C	T	0.692	0.999	FGF10
rs10512844	5	44,338,759	C	T	0.692	0.999	FGF10
rs72763190	5	44,339,429	T	C	0.899	0.998	FGF10
rs2330544	5	44,339,764	T	G	0.327	1	FGF10
rs72763193	5	44,339,785	T	C	0.899	0.998	FGF10
rs4866893	5	44,339,984	G	A	0.327	1	FGF10
rs1448041	5	44,340,198	C	T	0.981	0.999	FGF10
rs4866894	5	44,340,200	C	G	0.327	1	FGF10
rs16873982	5	44,340,261	A	C	0.692	0.999	FGF10
rs73089952	5	44,340,898	G	T	0.981	0.999	FGF10
rs16873983	5	44,340,975	T	G	0.692	0.999	FGF10
rs61032851	5	44,341,125	T	C	0.692	0.999	FGF10
rs10512845	5	44,341,336	A	G	0.692	0.999	FGF10
rs17317277	5	44,341,451	C	A	0.899	0.998	FGF10
5:44341596_TA_T	5	44,341,596	TA	T	0.98	0.973	FGF10
rs72763196	5	44,341,634	G	A	0.899	0.997	FGF10
rs6451761	5	44,341,979	A	G	0.308	1	FGF10
5:44342614_GA_G	5	44,342,614	GA	G	0.687	0.983	FGF10
rs116191041	5	44,343,040	T	A	0.985	0.923	FGF10
rs72763197	5	44,343,147	T	G	0.899	0.997	FGF10
rs1448040	5	44,345,200	A	T	0.327	1	FGF10
rs10073166	5	44,345,540	T	C	0.308	1	FGF10
rs7720510	5	44,345,922	C	G	0.981	0.999	FGF10
rs16874002	5	44,346,381	C	T	0.692	0.999	FGF10
rs6885842	5	44,346,636	C	T	0.981	0.998	FGF10
rs10069234	5	44,347,025	C	A	0.981	0.998	FGF10
rs7708529	5	44,347,131	T	C	0.327	1	FGF10
rs17234191	5	44,347,584	G	A	0.831	0.987	FGF10
rs12657059	5	44,347,791	C	T	0.692	0.999	FGF10
rs114841303	5	44,347,862	C	A	0.963	0.981	FGF10
rs139090003	5	44,348,067	A	G	0.987	0.912	FGF10
rs12523512	5	44,348,652	C	G	0.692	0.999	FGF10
rs769513714	5	44,348,776	GC	G	0.983	0.954	FGF10
rs199831527	5	44,348,778	T	A	0.981	0.998	FGF10
rs996935	5	44,348,789	A	C	0.308	1	FGF10
rs12516566	5	44,348,901	T	C	0.692	0.999	FGF10
rs12513830	5	44,348,987	C	A	0.692	0.999	FGF10
rs1448039	5	44,349,141	A	T	0.327	1	FGF10
rs72763201	5	44,349,311	T	C	0.899	0.998	FGF10
5:44349790_TA_T	5	44,349,790	TA	T	0.307	0.995	FGF10
rs72763202	5	44,350,684	T	C	0.692	0.999	FGF10
rs1448038	5	44,350,727	C	T	0.308	1	FGF10
rs11743802	5	44,351,154	T	C	0.83	0.987	FGF10
rs10056347	5	44,351,651	G	A	0.981	0.998	FGF10
rs78611334	5	44,353,398	T	G	0.98	0.958	FGF10
rs10060548	5	44,353,621	C	T	0.308	1	FGF10
rs1374970	5	44,353,985	G	A	0.327	0.999	FGF10
rs1374967	5	44,355,363	T	C	0.308	0.999	FGF10
rs4866895	5	44,355,621	A	T	0.799	0.998	FGF10
rs1550937	5	44,356,347	T	A	0.308	0.999	FGF10
5:44357220_TA_T	5	44,357,220	TA	T	0.309	0.995	FGF10
rs72764708	5	44,358,810	G	T	0.906	0.997	FGF10
rs593307	5	44,358,915	A	G	0.309	1	FGF10
rs1351635	5	44,359,299	A	G	0.328	1	FGF10
rs1482689	5	44,359,428	C	G	0.691	0.999	FGF10
rs339509	5	44,360,892	C	T	0.309	1	FGF10
rs339508	5	44,361,506	G	A	0.981	0.998	FGF10
rs10941664	5	44,362,134	C	T	0.691	0.999	FGF10
rs17234079	5	44,362,204	G	A	0.82	0.971	FGF10
rs1482671	5	44,362,538	G	A	0.328	1	FGF10
rs339505	5	44,362,560	A	G	0.309	1	FGF10
rs1482672	5	44,362,769	C	T	0.328	1	FGF10
rs339503	5	44,363,690	A	G	0.981	0.999	FGF10
rs339502	5	44,364,007	A	T	0.309	1	FGF10
rs34785719	5	44,365,452	C	CA	0.514	0.784	FGF10
rs16874036	5	44,366,552	G	A	0.691	0.999	FGF10
rs6859730	5	44,367,221	A	T	0.328	1	FGF10
5:44368100_AT_A	5	44,368,100	AT	A	0.965	0.939	FGF10
rs1482673	5	44,368,123	G	A	0.328	1	FGF10
5:44368475_CT_C	5	44,368,475	CT	C	0.989	0.982	FGF10
rs1482675	5	44,368,506	T	C	0.309	0.999	FGF10
rs1384448	5	44,368,775	G	T	0.98	0.987	FGF10
rs900379	5	44,369,656	C	T	0.328	0.999	FGF10
rs2128432	5	44,371,231	C	T	0.981	0.999	FGF10
rs2170582	5	44,371,669	A	G	0.328	0.999	FGF10
rs11750845	5	44,373,060	C	T	0.499	0.99	FGF10

SNP	Chromosome	Position	Effect Allele	Other Allele	Effect Allele Frequency	Imputation quality	Gene
rs2330550	5	44,373,759	A	G	0.981	0.995	FGF10
rs2128433	5	44,374,386	G	C	0.019	0.985	FGF10
rs72764774	5	44,374,651	T	C	0.98	0.947	FGF10
rs10805683	5	44,375,270	C	G	0.796	1	FGF10
rs6893398	5	44,376,222	C	T	0.892	0.975	FGF10
rs79290724	5	44,378,106	A	T	0.797	0.998	FGF10
rs773780440	5	44,378,181	AT	A	0.796	0.995	FGF10
rs62366720	5	44,379,936	C	G	0.985	0.926	FGF10
rs969520	5	44,380,206	G	T	0.799	0.998	FGF10
rs72764775	5	44,380,981	A	C	0.955	0.919	FGF10
rs16901816	5	44,381,698	T	G	0.799	0.997	FGF10
rs74701942	5	44,382,341	T	C	0.99	0.985	FGF10
rs143131329	5	44,382,701	G	A	0.977	0.954	FGF10
rs148280835	5	44,382,783	T	G	0.981	0.893	FGF10
rs6892212	5	44,382,842	C	A	0.223	0.997	FGF10
rs7727520	5	44,383,272	T	A	0.989	0.98	FGF10
5:44383385_GA_G	5	44,383,385	GA	G	0.981	0.997	FGF10
rs2973644	5	44,384,183	C	T	0.203	0.995	FGF10
rs116475073	5	44,384,206	A	T	0.973	0.923	FGF10
rs10512849	5	44,384,223	C	T	0.831	0.964	FGF10
rs1482679	5	44,385,415	G	A	0.331	0.987	FGF10
rs114626855	5	44,387,343	G	A	0.984	0.999	FGF10
rs2973646	5	44,387,537	A	C	0.204	0.996	FGF10
rs6891282	5	44,388,150	G	A	0.797	0.995	FGF10
rs17227508	5	44,389,468	C	G	0.861	0.945	FGF10
rs6198	5	142,657,621	T	C	0.838	1	NR3C1
rs6191	5	142,658,156	C	A	0.456	0.998	NR3C1
rs10482704	5	142,661,763	C	A	0.977	0.956	NR3C1
rs258750	5	142,661,889	A	G	0.669	0.999	NR3C1
rs148700478	5	142,664,035	C	CATTACTATACTACAGAAAATCTA	0.84	0.987	NR3C1
5:142665530_CTGAT_C	5	142,665,530	CTGAT	C	0.882	0.88	NR3C1
rs10482700	5	142,665,812	C	G	0.838	0.999	NR3C1
rs189363776	5	142,667,841	G	A	0.969	0.988	NR3C1
rs17209251	5	142,669,223	A	G	0.787	0.998	NR3C1
rs72801046	5	142,669,487	C	T	0.97	0.995	NR3C1
rs190488	5	142,670,308	T	G	0.672	0.999	NR3C1
rs150299999	5	142,671,092	A	ATTACTTTTCTTTAGCCTAAG	0.46	0.997	NR3C1
rs11750172	5	142,671,661	C	G	0.787	0.998	NR3C1
rs74888440	5	142,671,913	A	G	0.969	0.911	NR3C1
rs174050	5	142,672,316	G	A	0.834	0.999	NR3C1
rs79148397	5	142,673,089	C	A	0.974	0.909	NR3C1
rs17209258	5	142,673,397	A	G	0.787	0.998	NR3C1
rs758277133	5	142,673,939	AT	A	0.79	0.982	NR3C1
rs33944801	5	142,673,942	C	G	0.79	0.982	NR3C1
rs10482693	5	142,674,145	T	C	0.977	0.924	NR3C1
rs258813	5	142,674,690	G	A	0.672	1	NR3C1
rs10482689	5	142,675,823	C	T	0.838	1	NR3C1
5:142675946_ATAAAT_A	5	142,675,946	ATAAAT	A	0.834	0.996	NR3C1
5:142676181_AAAC_A	5	142,676,181	AAAC	A	0.834	0.996	NR3C1
rs258814	5	142,676,404	G	T	0.672	1	NR3C1
5:142678908_TA_T	5	142,678,908	TA	T	0.799	0.784	NR3C1
rs62375499	5	142,679,152	G	C	0.787	0.999	NR3C1
rs10482683	5	142,679,348	G	A	0.983	0.948	NR3C1
rs10482682	5	142,679,397	C	T	0.626	0.999	NR3C1
rs6188	5	142,680,344	C	A	0.672	1	NR3C1
rs10482675	5	142,681,087	A	ATAAAT	0.745	0.981	NR3C1
rs852975	5	142,683,741	T	C	0.671	0.999	NR3C1
rs852976	5	142,685,438	T	C	0.541	0.999	NR3C1
rs72801051	5	142,685,670	A	T	0.833	0.998	NR3C1
rs11395012	5	142,685,926	T	TA	0.788	0.995	NR3C1
rs79590198	5	142,686,936	G	A	0.943	0.976	NR3C1
rs141919131	5	142,686,991	T	C	0.983	0.95	NR3C1
rs72801052	5	142,687,699	T	C	0.837	1	NR3C1
rs72801054	5	142,687,742	T	C	0.834	1	NR3C1
rs2282800	5	142,688,871	C	T	0.923	0.989	NR3C1
rs852978	5	142,690,596	T	C	0.834	1	NR3C1
5:142691353_GT_G	5	142,691,353	GT	G	0.616	0.936	NR3C1
5:142691490_ACAGCCCATTTTCCTTGAGCCACAGTGCC_A	5	142,691,490	ACAGCCCATTTTCCTTGAGCCACAGTGCC	A	0.837	1	NR3C1
rs76794467	5	142,691,518	C	A	0.837	1	NR3C1
rs111616921	5	142,691,753	C	A	0.838	1	NR3C1
rs852979	5	142,693,398	T	C	0.671	1	NR3C1
rs4986593	5	142,693,846	A	G	0.787	0.998	NR3C1
rs10482665	5	142,694,301	A	G	0.838	1	NR3C1
5:142694665_GATAT_G	5	142,694,665	GATAT	G	0.98	0.916	NR3C1
rs860458	5	142,696,036	G	A	0.834	1	NR3C1
rs144365606	5	142,696,447	G	C	0.983	0.951	NR3C1
rs33388	5	142,697,295	A	T	0.54	0.999	NR3C1
rs116798177	5	142,697,929	A	G	0.95	0.989	NR3C1
rs72801058	5	142,699,197	G	A	0.969	0.988	NR3C1
rs33389	5	142,700,499	C	T	0.834	0.999	NR3C1
rs852980	5	142,700,856	G	C	0.541	0.999	NR3C1
rs10482659	5	142,701,967	G	A	0.983	0.95	NR3C1
rs10482658	5	142,702,077	C	T	0.987	0.948	NR3C1
rs852982	5	142,705,421	G	A	0.672	0.999	NR3C1
rs10482655	5	142,705,895	T	G	0.788	0.999	NR3C1
rs10482653	5	142,706,398	G	A	0.974	0.965	NR3C1
rs142327762	5	142,707,149	A	T	0.85	0.979	NR3C1
rs115108480	5	142,707,570	C	T	0.986	0.984	NR3C1
rs72801064	5	142,707,701	C	T	0.982	0.912	NR3C1
rs62375502	5	142,707,734	C	T	0.788	0.998	NR3C1
rs33380	5	142,708,469	G	C	0.672	1	NR3C1
rs33381	5	142,708,734	T	C	0.834	1	NR3C1
rs33382	5	142,708,852	G	A	0.834	0.999	NR3C1
rs17339455	5	142,709,723	T	C	0.788	0.999	NR3C1
rs33383	5	142,709,986	C	T	0.541	0.999	NR3C1
rs76657211	5	142,710,696	C	T	0.945	0.915	NR3C1
rs113988246	5	142,711,431	C	G	0.954	0.988	NR3C1
rs140269785	5	142,711,503	T	G	0.983	0.95	NR3C1
rs145566912	5	142,711,958	G	A	0.983	0.951	NR3C1
rs852983	5	142,712,064	G	A	0.541	0.999	NR3C1
rs852984	5	142,712,091	C	A	0.834	1	NR3C1
5:142712175_GT_G	5	142,712,175	GT	G	0.672	0.999	NR3C1
rs61752282	5	142,712,723	T	TA	0.672	1	NR3C1
rs145947048	5	142,712,879	C	A	0.97	0.99	NR3C1
rs540963589	5	142,713,780	C	G	0.983	0.706	NR3C1
rs11745958	5	142,716,357	C	T	0.787	0.997	NR3C1
rs145500647	5	142,716,601	C	G	0.983	0.938	NR3C1
rs2398628	5	142,717,725	C	A	0.839	0.996	NR3C1
rs2398629	5	142,717,726	A	C	0.857	0.952	NR3C1
rs2398630	5	142,717,727	T	A	0.853	0.97	NR3C1
rs72801070	5	142,718,261	A	G	0.838	1	NR3C1
rs2398631	5	142,718,290	G	C	0.788	0.998	NR3C1
5:142718805_TA_T	5	142,718,805	TA	T	0.834	0.999	NR3C1
rs142757510	5	142,718,938	C	T	0.983	0.951	NR3C1
rs141742535	5	142,719,914	C	CT	0.834	0.996	NR3C1
rs17100236	5	142,720,726	T	C	0.866	0.995	NR3C1
rs61752278	5	142,721,422	A	G	0.988	0.942	NR3C1
rs2918419	5	142,722,353	T	C	0.834	0.999	NR3C1
rs9324916	5	142,722,660	G	C	0.788	0.999	NR3C1
rs10482650	5	142,722,862	C	T	0.925	0.987	NR3C1
rs2918418	5	142,723,373	G	C	0.834	0.999	NR3C1
rs4912650	5	142,723,937	T	G	0.745	0.991	NR3C1
rs2963148	5	142,724,430	G	A	0.834	0.999	NR3C1
rs2963149	5	142,725,084	A	T	0.671	0.999	NR3C1
rs192127181	5	142,725,865	C	A	0.989	0.959	NR3C1
rs2918417	5	142,726,170	C	T	0.672	0.999	NR3C1
rs6877893	5	142,727,193	G	A	0.541	0.997	NR3C1
5:142727542_GT_G	5	142,727,542	GT	G	0.837	0.999	NR3C1
rs10482642	5	142,728,031	T	C	0.838	0.999	NR3C1
rs1438731	5	142,728,346	A	G	0.838	0.999	NR3C1
rs115130016	5	142,729,499	G	A	0.98	0.848	NR3C1
rs4912905	5	142,730,376	G	C	0.749	0.99	NR3C1
rs77877138	5	142,732,361	T	C	0.988	0.937	NR3C1
rs144497928	5	142,733,608	T	C	0.979	0.914	NR3C1
rs72801077	5	142,733,785	T	C	0.978	0.927	NR3C1
rs2963151	5	142,734,151	T	C	0.834	0.998	NR3C1
rs10042042	5	142,734,535	G	A	0.936	0.974	NR3C1
rs61752273	5	142,736,010	T	C	0.983	0.951	NR3C1
rs184652131	5	142,738,333	C	T	0.974	0.963	NR3C1
rs139043692	5	142,738,627	T	A	0.983	0.946	NR3C1
rs10515521	5	142,740,166	G	A	0.838	1	NR3C1
rs2918416	5	142,741,027	C	T	0.672	0.999	NR3C1
rs2963153	5	142,742,491	C	T	0.672	0.999	NR3C1
rs2963154	5	142,742,537	T	C	0.834	0.998	NR3C1
5:142743146_TA_T	5	142,743,146	TA	T	0.207	0.978	NR3C1
rs143331938	5	142,743,146	T	A	0.644	0.698	NR3C1
rs200007949	5	142,743,147	A	T	0.644	0.698	NR3C1
rs17339831	5	142,744,092	G	C	0.838	1	NR3C1
rs2918415	5	142,744,963	A	C	0.834	0.998	NR3C1
rs145347606	5	142,745,315	T	G	0.982	0.932	NR3C1
rs79713793	5	142,745,502	G	A	0.962	0.982	NR3C1
rs4912906	5	142,745,955	A	G	0.788	0.997	NR3C1
rs111440401	5	142,746,023	C	T	0.672	0.999	NR3C1
5:142746075_CA_C	5	142,746,075	CA	C	0.67	0.983	NR3C1
rs116641439	5	142,747,288	A	G	0.99	0.975	NR3C1

SNP	Chromosome	Position	Effect Allele	Other Allele	Effect Allele Frequency	Imputation quality	Gene
rs11740792	5	142,747,320	A	G	0.838	1	NR3C1
rs10482634	5	142,750,449	A	G	0.788	0.997	NR3C1
rs72801079	5	142,751,824	C	T	0.838	1	NR3C1
rs72801080	5	142,751,990	T	C	0.834	0.998	NR3C1
rs78885810	5	142,752,099	C	T	0.955	0.971	NR3C1
rs61752263	5	142,752,480	C	T	0.834	0.998	NR3C1
rs72801081	5	142,753,669	C	A	0.838	1	NR3C1
rs10076665	5	142,753,796	G	A	0.838	1	NR3C1
rs760524235	5	142,754,055	C	CA	0.705	0.919	NR3C1
rs17399352	5	142,754,690	T	C	0.788	0.996	NR3C1
rs72801083	5	142,754,904	T	C	0.838	1	NR3C1
rs2963155	5	142,756,004	A	G	0.756	0.995	NR3C1
rs1438732	5	142,756,266	G	C	0.834	0.998	NR3C1
5:142757641_CCT_C	5	142,757,641	CCT	C	0.838	0.994	NR3C1
rs10515522	5	142,758,394	T	C	0.834	0.998	NR3C1
rs2963156	5	142,758,496	T	C	0.212	0.996	NR3C1
rs143052982	5	142,759,354	C	G	0.983	0.952	NR3C1
rs1866388	5	142,759,785	A	G	0.672	0.998	NR3C1
rs116298742	5	142,760,306	C	T	0.987	0.882	NR3C1
rs62375506	5	142,760,382	T	C	0.98	0.995	NR3C1
rs72801086	5	142,761,039	C	T	0.837	0.999	NR3C1
rs1837262	5	142,761,527	C	A	0.328	0.999	NR3C1
rs4128428	5	142,761,813	T	C	0.838	0.997	NR3C1
5:142763030_TA_T	5	142,763,030	TA	T	0.925	0.954	NR3C1
rs11740747	5	142,763,880	G	A	0.838	0.999	NR3C1
rs7716360	5	142,764,741	T	C	0.54	0.997	NR3C1
rs187197125	5	142,765,032	C	T	0.987	0.859	NR3C1
rs56150733	5	142,765,099	T	C	0.834	0.998	NR3C1
rs10060545	5	142,765,135	G	A	0.674	0.993	NR3C1
rs10053679	5	142,765,136	A	T	0.672	0.999	NR3C1
rs55817235	5	142,765,733	C	T	0.834	0.998	NR3C1
rs10482628	5	142,766,656	T	A	0.983	0.952	NR3C1
rs10482626	5	142,766,993	T	C	0.983	0.953	NR3C1
rs9324918	5	142,767,160	T	C	0.829	0.992	NR3C1
rs151159692	5	142,768,674	C	T	0.99	0.925	NR3C1
rs145138434	5	142,769,748	T	C	0.988	0.959	NR3C1
rs61751250	5	142,770,225	C	T	0.983	0.948	NR3C1
rs184065886	5	142,770,848	T	G	0.983	0.94	NR3C1
rs72801088	5	142,772,590	T	C	0.838	1	NR3C1
rs77979848	5	142,772,652	G	C	0.838	1	NR3C1
rs72801090	5	142,774,692	C	T	0.835	0.996	NR3C1
5:142776256_CTTAATT_C	5	142,776,256	CTTAATT	C	0.839	0.996	NR3C1
rs61751173	5	142,776,433	A	G	0.911	0.913	NR3C1
rs10041520	5	142,776,684	T	C	0.541	0.997	NR3C1
rs41423247	5	142,778,575	G	C	0.619	0.996	NR3C1
rs10482623	5	142,779,000	T	C	0.99	0.926	NR3C1
rs56149945	5	142,779,317	T	C	0.968	0.919	NR3C1
rs4582314	5	142,780,695	C	A	0.542	0.996	NR3C1
rs4634384	5	142,780,697	C	T	0.542	0.995	NR3C1
rs10482616	5	142,781,567	C	T	0.869	0.994	NR3C1
rs543125075	5	142,782,335	C	T	0.987	0.872	NR3C1
rs10482614	5	142,782,402	C	T	0.834	0.997	NR3C1
rs10482605	5	142,783,521	A	G	0.836	0.997	NR3C1
rs5871845	5	142,783,949	C	CG	0.945	0.961	NR3C1
rs3806854	5	142,784,127	A	G	0.834	0.994	NR3C1
rs3806855	5	142,784,129	A	C	0.834	0.994	NR3C1
rs72801094	5	142,785,905	T	C	0.959	0.881	NR3C1
rs367680772	5	142,785,973	CA	C	0.755	0.868	NR3C1
rs10052957	5	142,786,701	G	A	0.672	0.992	NR3C1
5:142788097_AT_A	5	142,788,097	AT	A	0.946	0.963	NR3C1
rs5871846	5	142,788,147	G	GC	0.836	0.989	NR3C1
rs72801096	5	142,790,449	C	T	0.835	0.99	NR3C1
rs11747997	5	142,790,752	A	C	0.671	0.991	NR3C1
rs4912908	5	142,791,133	G	A	0.216	0.983	NR3C1
rs6865292	5	142,792,990	T	C	0.723	0.961	NR3C1
rs73797465	5	142,793,467	G	T	0.932	0.945	NR3C1
rs4244032	5	142,794,725	A	G	0.804	0.982	NR3C1
rs140977881	5	142,795,478	G	A	0.988	0.943	NR3C1
rs4607376	5	142,796,532	A	G	0.467	0.959	NR3C1
rs67355570	5	142,797,911	T	C	0.808	0.999	NR3C1
rs13166252	5	142,798,197	G	A	0.808	0.999	NR3C1
rs72802806	5	142,798,588	G	A	0.769	0.954	NR3C1
rs12653301	5	142,798,757	G	A	0.729	0.952	NR3C1
rs66504964	5	142,798,950	T	C	0.809	0.998	NR3C1
rs67962882	5	142,798,962	T	A	0.809	0.999	NR3C1
rs67002639	5	142,799,018	A	G	0.809	0.999	NR3C1
rs68065224	5	142,799,090	A	G	0.808	0.999	NR3C1
rs12515985	5	142,799,300	G	A	0.788	0.949	NR3C1
rs34113205	5	142,799,322	A	G	0.808	0.999	NR3C1
rs34449288	5	142,799,479	C	G	0.808	0.998	NR3C1
rs77857185	5	142,799,536	C	T	0.811	0.99	NR3C1
rs78461617	5	142,799,537	A	G	0.811	0.99	NR3C1
rs35515833	5	142,799,584	A	G	0.808	0.999	NR3C1
rs35314635	5	142,799,793	A	G	0.808	0.998	NR3C1
rs4388235	5	142,799,811	T	C	0.229	0.952	NR3C1
rs13173940	5	142,800,039	C	T	0.809	0.997	NR3C1
rs34226461	5	142,800,061	G	T	0.809	0.997	NR3C1
rs35224915	5	142,800,114	A	G	0.809	0.997	NR3C1
rs34519266	5	142,800,122	T	G	0.811	0.991	NR3C1
rs6894830	5	142,800,289	C	T	0.935	0.941	NR3C1
rs35290813	5	142,800,290	G	A	0.809	0.997	NR3C1
rs35612073	5	142,800,292	C	T	0.809	0.997	NR3C1
rs67752035	5	142,800,501	C	A	0.808	0.999	NR3C1
rs36029993	5	142,801,104	A	AT	0.808	0.999	NR3C1
rs386693071	5	142,801,356	C	CA	0.81	0.993	NR3C1
rs13182800	5	142,801,480	G	T	0.809	0.997	NR3C1
rs13169606	5	142,801,491	T	C	0.809	0.997	NR3C1
rs72802809	5	142,801,564	T	C	0.808	0.999	NR3C1
rs13170129	5	142,801,821	T	C	0.809	0.999	NR3C1
rs35500391	5	142,801,838	A	G	0.809	0.999	NR3C1
rs34396341	5	142,802,325	C	G	0.808	0.996	NR3C1
rs72802810	5	142,802,679	G	A	0.814	0.984	NR3C1
rs72802811	5	142,802,680	T	G	0.814	0.984	NR3C1
rs72802812	5	142,803,017	C	T	0.815	0.982	NR3C1
rs72802813	5	142,803,032	G	A	0.815	0.982	NR3C1
rs12513857	5	142,803,175	C	T	0.681	0.939	NR3C1
rs72802815	5	142,803,548	A	G	0.623	0.855	NR3C1
rs13155635	5	142,803,558	G	A	0.618	0.876	NR3C1
rs4434414	5	142,803,748	G	C	0.695	0.91	NR3C1
5:142803772_TATA_T	5	142,803,772	TATA	T	0.707	0.907	NR3C1
rs70995003	5	142,803,807	A	AG	0.238	0.808	NR3C1
rs139648785	5	142,803,826	G	A	0.984	0.947	NR3C1
rs148442596	5	142,804,673	T	A	0.968	0.951	NR3C1
5:142805275_TA_T	5	142,805,275	TA	T	0.95	0.91	NR3C1
rs62375510	5	142,805,867	G	A	0.825	0.962	NR3C1
rs10477211	5	142,805,874	G	A	0.267	0.971	NR3C1
rs12054797	5	142,805,902	C	T	0.733	0.971	NR3C1
rs4912910	5	142,806,890	A	G	0.333	0.97	NR3C1
rs4912911	5	142,807,032	G	A	0.333	0.97	NR3C1
rs184629364	5	142,807,127	G	A	0.977	0.949	NR3C1
rs4912912	5	142,807,150	T	C	0.551	0.968	NR3C1
rs17100289	5	142,808,642	T	A	0.333	0.971	NR3C1
rs12656106	5	142,808,947	G	C	0.55	0.97	NR3C1
rs12655166	5	142,809,272	T	C	0.733	0.976	NR3C1
rs113394833	5	142,809,410	C	G	0.934	0.952	NR3C1
rs17302697	5	142,809,717	A	G	0.667	0.971	NR3C1
rs7719514	5	142,810,124	G	A	0.949	0.954	NR3C1
rs150383054	5	142,812,956	A	AAT	0.359	0.918	NR3C1
rs144498485	5	142,813,017	TTATA	T	0.07	0.925	NR3C1
rs140355433	5	170,846,683	C	T	0.986	0.803	FGF18
rs6887323	5	170,848,124	G	A	0.463	0.974	FGF18
rs188028065	5	170,848,181	G	A	0.988	0.906	FGF18
rs76124313	5	170,848,209	C	T	0.988	0.904	FGF18
rs111287484	5	170,849,617	T	C	0.98	0.891	FGF18
rs12521055	5	170,851,553	C	T	0.658	0.947	FGF18
rs72829768	5	170,851,565	C	T	0.97	0.973	FGF18
rs4559013	5	170,851,624	A	G	0.49	0.984	FGF18
5:170852135_CAGG_C	5	170,852,135	CAGG	C	0.954	0.912	FGF18
rs62383987	5	170,852,673	T	A	0.822	0.997	FGF18
rs111779186	5	170,854,162	G	T	0.834	0.991	FGF18
rs77876641	5	170,854,757	A	C	0.966	0.974	FGF18
rs76314553	5	170,854,838	G	A	0.906	0.984	FGF18
rs79734481	5	170,855,126	C	T	0.907	0.983	FGF18
rs75831829	5	170,855,335	C	T	0.766	0.991	FGF18
rs7723046	5	170,856,083	T	A	0.822	0.997	FGF18
rs74951660	5	170,856,333	G	A	0.801	0.986	FGF18
rs115627605	5	170,856,519	G	A	0.976	0.964	FGF18
5:170857190_GTC_G	5	170,857,190	GTC	G	0.978	0.95	FGF18
rs10041856	5	170,857,277	A	G	0.822	0.997	FGF18
rs12186915	5	170,857,709	G	A	0.823	0.995	FGF18
rs778223954	5	170,857,798	CAAAAAAAAAAAA	C	0.738	0.896	FGF18
rs10434691	5	170,858,640	G	A	0.823	0.996	FGF18
rs12515997	5	170,859,002	G	A	0.823	0.996	FGF18
rs10475978	5	170,859,228	G	C	0.526	0.993	FGF18
rs10475979	5	170,859,351	T	C	0.822	0.995	FGF18

SNP	Chromosome	Position	Effect Allele	Other Allele	Effect Allele Frequency	Imputation quality	Gene
rs146075986	5	170,859,363	T	TTTC	0.727	0.985	FGF18
rs142041482	5	170,859,520	C	A	0.982	0.932	FGF18
rs148511414	5	170,859,686	G	A	0.987	0.931	FGF18
rs79711557	5	170,859,847	A	T	0.906	0.986	FGF18
rs80011133	5	170,860,017	G	A	0.98	0.965	FGF18
rs35261861	5	170,860,021	G	A	0.976	0.902	FGF18
rs11738946	5	170,861,437	C	T	0.835	0.991	FGF18
rs72829770	5	170,863,313	C	T	0.97	0.979	FGF18
rs4076077	5	170,863,509	C	T	0.518	0.993	FGF18
rs4074237	5	170,863,653	A	T	0.518	0.993	FGF18
rs4073717	5	170,864,021	G	T	0.799	0.994	FGF18
rs4073716	5	170,864,239	A	G	0.514	0.993	FGF18
rs11955153	5	170,864,548	T	C	0.765	0.993	FGF18
5:170865103_CT_C	5	170,865,103	CT	C	0.7	0.83	FGF18
rs111365325	5	170,865,229	C	T	0.769	0.993	FGF18
rs62383992	5	170,866,296	C	A	0.957	0.936	FGF18
rs10063512	5	170,866,758	G	C	0.764	0.99	FGF18
5:170867229_TG_T	5	170,867,229	TG	T	0.752	0.972	FGF18
rs61209923	5	170,867,231	G	T	0.894	0.988	FGF18
rs6555956	5	170,867,375	T	G	0.774	0.993	FGF18
rs3934591	5	170,867,577	A	G	0.481	0.996	FGF18
rs10065728	5	170,868,059	C	T	0.828	0.993	FGF18
rs144034067	5	170,868,464	A	C	0.988	0.962	FGF18
rs10067840	5	170,869,014	C	T	0.827	0.993	FGF18
rs12519454	5	170,869,173	C	T	0.865	0.978	FGF18
rs10077350	5	170,869,419	T	C	0.826	0.992	FGF18
rs10077440	5	170,869,582	T	C	0.594	0.997	FGF18
rs10069857	5	170,869,797	C	T	0.827	0.993	FGF18
rs551889632	5	170,870,139	C	CTTT	0.793	0.961	FGF18
rs111685984	5	170,870,300	A	AT	0.767	0.987	FGF18
rs112851629	5	170,870,505	A	T	0.767	0.987	FGF18
rs6886141	5	170,870,546	A	G	0.487	0.994	FGF18
rs10475560	5	170,870,645	C	T	0.828	0.991	FGF18
rs6867594	5	170,870,649	T	C	0.594	0.998	FGF18
rs80232002	5	170,870,847	C	T	0.894	0.996	FGF18
rs5873259	5	170,871,063	T	TC	0.49	0.996	FGF18
rs5005937	5	170,871,072	C	G	0.661	0.992	FGF18
rs78993631	5	170,872,332	A	G	0.832	0.987	FGF18
rs73327280	5	170,872,348	A	G	0.832	0.988	FGF18
rs140690032	5	170,872,538	G	GT	0.835	0.96	FGF18
rs149289505	5	170,872,907	G	A	0.979	0.942	FGF18
rs68176291	5	170,873,076	A	G	0.683	0.989	FGF18
rs62383994	5	170,874,138	T	C	0.654	0.985	FGF18
rs72829779	5	170,875,051	G	A	0.952	0.967	FGF18
rs4620037	5	170,875,097	A	C	0.786	0.983	FGF18
rs6891250	5	170,875,176	T	C	0.872	0.993	FGF18
rs11740426	5	170,875,445	A	G	0.949	0.973	FGF18
rs35786776	5	170,875,656	T	G	0.94	0.891	FGF18
rs72829781	5	170,877,246	T	G	0.766	0.985	FGF18
rs73327284	5	170,878,000	T	C	0.769	0.996	FGF18
rs142469471	5	170,878,247	G	A	0.949	0.887	FGF18
rs149660867	5	170,878,614	C	T	0.989	0.93	FGF18
rs73327286	5	170,879,222	A	G	0.768	0.999	FGF18
rs74629240	5	170,879,705	G	A	0.986	0.963	FGF18
rs62383999	5	170,880,615	C	T	0.768	0.999	FGF18
rs62384000	5	170,880,624	G	T	0.768	0.999	FGF18
rs74578427	5	170,882,151	A	G	0.986	0.96	FGF18
rs2043278	5	170,882,607	A	G	0.233	0.997	FGF18
rs34347344	5	170,883,734	G	A	0.953	0.964	FGF18
5:170884162_GA_G	5	170,884,162	GA	G	0.97	0.745	FGF18
rs3135911	5	176,513,896	C	A	0.709	0.979	FGFR4
rs2456173	5	176,514,595	T	C	0.214	0.996	FGFR4
5:176514781_ACAG_A	5	176,514,781	ACAG	A	0.971	0.993	FGFR4
rs3135913	5	176,515,113	G	A	0.972	0.995	FGFR4
rs3135914	5	176,515,158	A	G	0.971	0.994	FGFR4
rs11429362	5	176,515,591	A	AC	0.214	0.996	FGFR4
rs45603934	5	176,515,816	C	CCCA	0.215	0.995	FGFR4
rs766709074	5	176,515,866	AG	A	0.243	0.996	FGFR4
rs3135918	5	176,516,537	C	G	0.971	0.998	FGFR4
rs434434	5	176,516,953	A	G	0.214	1	FGFR4
rs755389246	5	176,517,136	C	CGTGT	0.25	0.984	FGFR4
rs446382	5	176,517,461	T	G	0.274	0.999	FGFR4
rs376618	5	176,517,797	C	T	0.243	1	FGFR4
rs439213	5	176,518,315	A	C	0.243	1	FGFR4
rs396007	5	176,518,426	T	C	0.214	1	FGFR4
rs393923	5	176,519,287	G	A	0.214	0.999	FGFR4
rs55658990	5	176,519,730	C	T	0.988	0.902	FGFR4
rs31778	5	176,522,036	C	T	0.708	0.996	FGFR4
rs3135928	5	176,522,062	G	A	0.941	0.996	FGFR4
rs31777	5	176,523,562	C	A	0.214	0.998	FGFR4
rs31776	5	176,523,597	A	G	0.274	0.998	FGFR4
rs72494545	5	176,523,861	C	T	0.942	0.9	FGFR4
rs764745618	5	176,524,103	G	C	0.818	0.615	FGFR4
rs113339725	5	176,524,110	T	C	0.948	0.553	FGFR4
rs146820395	5	176,524,465	C	T	0.98	0.898	FGFR4
rs9489	5	176,525,029	G	C	0.859	0.992	FGFR4
rs1076890	5	176,525,071	G	A	0.941	0.995	FGFR4
rs17133512	6	7,542,148	C	CA	0.866	0.908	DSP
rs36087964	6	7,542,274	T	C	0.959	0.762	DSP
6:7542511_TC_T	6	7,542,511	TC	T	0.782	0.929	DSP
rs115370329	6	7,542,553	G	A	0.979	0.962	DSP
rs2757624	6	7,542,881	C	T	0.946	0.973	DSP
rs73373373	6	7,542,903	C	G	0.946	0.974	DSP
rs2744389	6	7,543,123	A	C	0.819	0.991	DSP
rs6899532	6	7,543,433	G	C	0.917	0.983	DSP
rs2806167	6	7,543,783	T	G	0.623	0.995	DSP
rs7764001	6	7,543,987	T	C	0.624	0.998	DSP
rs7767989	6	7,544,001	T	C	0.624	0.998	DSP
rs7743128	6	7,544,073	G	A	0.624	0.999	DSP
rs2299032	6	7,544,230	C	T	0.624	0.999	DSP
rs7764163	6	7,544,288	A	G	0.624	0.999	DSP
rs9502619	6	7,544,451	C	G	0.624	0.997	DSP
rs72825058	6	7,545,081	C	T	0.903	0.984	DSP
6:7545870_TC_T	6	7,545,870	TC	T	0.844	0.993	DSP
rs11754974	6	7,545,921	G	T	0.84	0.981	DSP
rs72825059	6	7,545,931	C	T	0.964	0.976	DSP
rs1998323	6	7,546,404	A	G	0.374	0.987	DSP
rs62388072	6	7,546,842	C	T	0.84	0.982	DSP
6:7547834_ATT_A	6	7,547,834	ATT	A	0.691	0.959	DSP
rs11756882	6	7,548,415	G	A	0.912	0.961	DSP
rs2237101	6	7,548,619	C	T	0.916	0.992	DSP
rs2299033	6	7,548,899	G	C	0.383	0.985	DSP
rs2299034	6	7,549,056	C	A	0.776	0.993	DSP
rs2261169	6	7,549,125	T	G	0.374	0.986	DSP
rs2299035	6	7,549,161	G	A	0.776	0.993	DSP
rs74816192	6	7,549,223	G	A	0.979	0.935	DSP
rs2299036	6	7,549,628	C	T	0.777	0.993	DSP
rs2806166	6	7,550,345	A	G	0.758	0.998	DSP
rs4053176	6	7,550,457	G	T	0.373	0.978	DSP
rs874646	6	7,550,691	A	G	0.758	0.998	DSP
rs1998324	6	7,551,163	T	C	0.776	0.993	DSP
rs2744362	6	7,551,208	A	C	0.758	0.999	DSP
rs2008748	6	7,551,678	T	G	0.758	0.999	DSP
rs2008756	6	7,551,792	T	G	0.758	0.999	DSP
6:7551862_CA_C	6	7,551,862	CA	C	0.757	0.995	DSP
rs2237102	6	7,552,145	A	G	0.759	0.999	DSP
rs2237103	6	7,552,162	T	G	0.759	0.999	DSP
rs2237104	6	7,552,172	G	T	0.759	0.999	DSP
rs2237105	6	7,552,555	G	A	0.758	0.999	DSP
rs35879281	6	7,552,634	CAA	C	0.748	0.996	DSP
rs377022218	6	7,552,797	G	GAAA	0.903	0.959	DSP
rs561511771	6	7,552,895	G	GT	0.588	0.937	DSP
rs75945653	6	7,553,063	T	G	0.957	0.978	DSP
rs2806165	6	7,553,121	T	C	0.373	0.987	DSP
rs2078438	6	7,553,229	G	A	0.758	0.998	DSP
rs2744368	6	7,553,390	G	T	0.758	0.998	DSP
rs2744369	6	7,553,470	G	T	0.758	0.997	DSP
rs2744370	6	7,553,996	T	C	0.758	0.995	DSP
rs6913983	6	7,554,144	A	C	0.776	0.99	DSP
rs2744371	6	7,554,174	A	C	0.758	0.994	DSP
rs74692364	6	7,554,358	A	C	0.912	0.95	DSP
rs2744372	6	7,554,466	A	G	0.759	0.993	DSP
rs2744373	6	7,554,527	G	A	0.843	0.99	DSP
rs2744374	6	7,554,609	G	T	0.758	0.993	DSP
rs12195095	6	7,554,674	C	T	0.84	0.983	DSP
rs2744375	6	7,554,839	A	T	0.843	0.989	DSP
rs12203720	6	7,555,235	A	T	0.842	0.983	DSP
rs7774328	6	7,555,249	G	C	0.687	0.983	DSP
6:7555426_AT_A	6	7,555,426	AT	A	0.983	0.836	DSP
rs546806337	6	7,555,428	T	G	0.984	0.866	DSP
rs2842685	6	7,555,520	A	G	0.648	0.982	DSP
rs147152068	6	7,555,634	G	GTTTA	0.627	0.968	DSP
rs2076294	6	7,555,666	A	G	0.624	0.982	DSP
rs9392904	6	7,555,854	G	C	0.892	0.97	DSP
rs56148603	6	7,556,063	C	T	0.959	0.962	DSP

SNP	Chromosome	Position	Effect Allele	Other Allele	Effect Allele Frequency	Imputation quality	Gene
rs12198780	6	7,556,544	C	G	0.601	0.986	DSP
rs112535790	6	7,556,704	A	T	0.974	0.916	DSP
rs12207400	6	7,556,962	T	G	0.842	0.999	DSP
rs3799525	6	7,557,248	A	G	0.759	0.986	DSP
rs750750942	6	7,558,002	GTACCACT	G	0.573	0.985	DSP
6:7558010_GTAC_G	6	7,558,010	GTAC	G	0.573	0.985	DSP
rs10484326	6	7,558,318	T	C	0.759	0.987	DSP
rs36122115	6	7,558,772	G	A	0.852	0.99	DSP
rs115291054	6	7,560,548	A	G	0.979	0.938	DSP
rs139579924	6	7,560,824	A	G	0.961	0.937	DSP
rs2757629	6	7,561,345	A	C	0.581	0.984	DSP
rs2757630	6	7,561,359	C	T	0.579	0.985	DSP
rs2757631	6	7,561,533	G	A	0.974	0.978	DSP
rs2757632	6	7,561,661	A	G	0.58	0.99	DSP
rs2076295	6	7,563,232	T	G	0.549	0.993	DSP
rs565520948	6	7,563,546	CT	C	0.836	0.796	DSP
rs11754421	6	7,563,564	A	T	0.697	0.978	DSP
rs2806234	6	7,563,983	T	A	0.467	0.803	DSP
rs1358903	6	7,564,139	C	G	0.709	0.998	DSP
rs1358904	6	7,564,461	C	T	0.709	0.998	DSP
rs34533690	6	7,564,885	A	G	0.709	0.998	DSP
rs561618054	6	7,565,035	C	CAACTATAT	0.71	0.992	DSP
rs55938083	6	7,565,376	C	T	0.709	0.999	DSP
rs35266991	6	7,565,490	A	AT	0.584	0.985	DSP
rs3778338	6	7,566,168	T	A	0.71	0.999	DSP
rs2842683	6	7,566,386	T	C	0.529	0.996	DSP
rs2757633	6	7,566,415	T	C	0.238	0.996	DSP
rs200962599	6	7,566,745	G	GA	0.99	0.646	DSP
rs2076296	6	7,567,970	C	T	0.239	0.997	DSP
rs28763960	6	7,568,306	C	T	0.941	0.963	DSP
rs2076297	6	7,568,354	G	T	0.239	0.997	DSP
rs11755954	6	7,569,326	C	T	0.845	0.987	DSP
6:7570067_CA_C	6	7,570,067	CA	C	0.072	0.882	DSP
rs2076301	6	7,570,309	T	A	0.237	0.997	DSP
rs2076302	6	7,570,963	A	G	0.237	0.998	DSP
rs58040819	6	7,570,982	C	G	0.85	0.985	DSP
rs149295757	6	7,571,029	C	T	0.989	0.916	DSP
rs2806233	6	7,571,327	G	T	0.072	0.978	DSP
rs369265474	6	7,571,409	CA	C	0.577	0.781	DSP
rs926411	6	7,571,874	G	A	0.236	0.999	DSP
rs2076303	6	7,572,026	T	A	0.589	0.986	DSP
rs17143007	6	7,573,455	C	T	0.932	0.975	DSP
rs9505232	6	7,573,550	G	A	0.8	0.965	DSP
rs9791321	6	7,573,945	A	G	0.566	0.944	DSP
rs4960330	6	7,573,956	G	T	0.455	0.923	DSP
rs2259208	6	7,574,805	C	T	0.38	0.99	DSP
rs2806232	6	7,576,368	G	A	0.649	0.992	DSP
rs2260359	6	7,577,079	G	A	0.65	0.992	DSP
rs2064217	6	7,577,260	C	T	0.768	0.994	DSP
rs2064218	6	7,577,354	C	A	0.381	0.993	DSP
6:7577554_GTGTT_G	6	7,577,554	GTGTT	G	0.906	0.921	DSP
rs2806231	6	7,577,744	A	G	0.648	0.992	DSP
rs6942260	6	7,578,189	G	A	0.695	0.993	DSP
rs2806230	6	7,578,343	G	C	0.206	0.999	DSP
6:7578392_TGAGA_T	6	7,578,392	TGAGA	T	0.306	0.992	DSP
rs2806229	6	7,578,819	T	G	0.208	0.999	DSP
rs2842691	6	7,578,823	G	A	0.208	0.998	DSP
rs2744378	6	7,578,970	C	T	0.308	0.995	DSP
rs2806228	6	7,579,156	A	G	0.308	0.995	DSP
rs2757634	6	7,579,311	G	T	0.308	0.995	DSP
rs2806227	6	7,579,363	A	T	0.308	0.995	DSP
rs2744379	6	7,579,393	C	T	0.309	0.992	DSP
rs2806226	6	7,579,394	A	G	0.307	0.992	DSP
rs2806225	6	7,579,412	A	G	0.306	0.995	DSP
rs6905839	6	7,579,436	G	A	0.964	0.996	DSP
rs61731476	6	7,580,386	G	A	0.984	0.963	DSP
rs141534770	6	7,582,184	T	G	0.989	0.913	DSP
6:7582372_GGT_G	6	7,582,372	GGT	G	0.392	0.958	DSP
rs2757591	6	7,582,399	G	A	0.383	0.991	DSP
rs758188958	6	7,582,460	ATATTT	A	0.772	0.995	DSP
rs3799526	6	7,582,640	A	G	0.962	0.998	DSP
rs78652302	6	7,582,993	A	T	0.99	0.91	DSP
rs2076300	6	7,584,617	C	T	0.65	0.994	DSP
rs11558731	6	7,585,670	C	A	0.935	0.982	DSP
rs2744380	6	7,585,967	G	C	0.305	0.997	DSP
rs12250	6	7,586,308	C	T	0.77	0.997	DSP
6:7586386_TCTC_T	6	7,586,386	TCTC	T	0.976	0.921	DSP
rs3117579	6	31,633,496	G	T	0.802	1	CSNK2B
rs539322116	6	31,634,288	C	CT	0.986	0.935	CSNK2B
rs9267529	6	31,634,570	A	C	0.937	1	CSNK2B
rs36048548	6	31,634,971	C	T	0.962	0.977	CSNK2B
rs35149168	6	31,635,198	T	C	0.942	0.978	CSNK2B
6:31635958_CTAT_C	6	31,635,958	CTAT	C	0.822	0.998	CSNK2B
rs575597758	6	31,635,963	CCAAA	C	0.822	1	CSNK2B
rs3117578	6	31,636,031	A	G	0.15	1	CSNK2B
rs9267531	6	31,636,742	A	G	0.865	1	CSNK2B
rs3134941	6	32,149,537	C	G	0.799	1	AGER
rs55640627	6	32,149,756	C	T	0.919	0.973	AGER
rs9391855	6	32,149,801	C	T	0.936	0.999	AGER
rs3134940	6	32,149,816	T	C	0.799	1	AGER
rs35795092	6	32,151,420	G	C	0.942	0.996	AGER
rs2070600	6	32,151,443	C	T	0.935	1	AGER
rs11571502	6	35,310,411	G	C	0.981	0.999	PPARD
rs9658058	6	35,310,432	C	T	0.963	1	PPARD
rs11571504	6	35,310,749	T	A	0.94	0.999	PPARD
6:35311264_CACTCT_C	6	35,311,264	CACTCT	C	0.983	1	PPARD
rs11420189	6	35,311,760	C	CT	0.948	0.97	PPARD
rs6920146	6	35,312,267	G	A	0.983	1	PPARD
rs73411747	6	35,312,668	A	G	0.954	1	PPARD
rs13362808	6	35,313,348	G	C	0.95	1	PPARD
rs9658061	6	35,313,442	G	C	0.955	1	PPARD
rs9658067	6	35,313,868	A	G	0.951	1	PPARD
rs9368859	6	35,315,632	C	T	0.986	0.982	PPARD
rs9368860	6	35,315,637	C	T	0.986	0.999	PPARD
rs370033786	6	35,315,685	CT	C	0.969	0.648	PPARD
rs369167848	6	35,316,803	CT	C	0.957	0.804	PPARD
rs6457813	6	35,317,301	C	T	0.975	1	PPARD
rs111494920	6	35,317,464	G	GT	0.98	0.99	PPARD
rs6457814	6	35,317,603	C	G	0.975	1	PPARD
rs111244160	6	35,318,338	A	G	0.94	0.997	PPARD
rs59654131	6	35,318,692	C	T	0.955	1	PPARD
6:35318739_TTA_T	6	35,318,739	TTA	T	0.332	0.982	PPARD
rs61280851	6	35,319,043	T	C	0.955	1	PPARD
rs56987668	6	35,319,253	T	C	0.963	0.999	PPARD
rs73411755	6	35,319,290	G	C	0.983	0.998	PPARD
rs58114880	6	35,319,451	G	C	0.955	1	PPARD
rs113149304	6	35,319,793	G	GCACC	0.963	0.998	PPARD
rs7771323	6	35,320,448	A	G	0.955	1	PPARD
rs6937510	6	35,320,811	C	T	0.983	1	PPARD
rs112706550	6	35,321,978	C	T	0.931	0.992	PPARD
rs113176460	6	35,321,987	C	T	0.983	1	PPARD
rs150755816	6	35,322,156	C	A	0.983	1	PPARD
rs7744392	6	35,322,763	A	G	0.955	1	PPARD
rs9296147	6	35,323,488	G	A	0.028	0.99	PPARD
rs9942488	6	35,323,546	T	G	0.955	1	PPARD
rs9942489	6	35,323,709	T	A	0.963	1	PPARD
rs9942465	6	35,324,213	C	A	0.98	1	PPARD
rs9942501	6	35,324,514	T	C	0.955	1	PPARD
rs113775775	6	35,325,056	T	C	0.988	0.976	PPARD
rs9368861	6	35,325,374	T	C	0.027	0.97	PPARD
rs150495156	6	35,325,400	G	A	0.983	1	PPARD
rs61333739	6	35,326,284	A	G	0.955	1	PPARD
rs4713853	6	35,327,355	T	C	0.924	0.999	PPARD
rs9658077	6	35,327,361	G	A	0.988	0.946	PPARD
rs9658079	6	35,327,577	T	C	0.981	1	PPARD
rs113792819	6	35,329,241	G	A	0.986	0.953	PPARD
rs569444745	6	35,329,460	C	T	0.988	0.569	PPARD
rs147902147	6	35,329,477	G	A	0.99	0.86	PPARD
rs73411769	6	35,329,545	C	T	0.983	1	PPARD
rs6901410	6	35,330,030	T	C	0.94	1	PPARD
rs11961212	6	35,330,150	T	C	0.983	1	PPARD
rs34916659	6	35,330,223	C	CTT	0.981	0.941	PPARD
rs6902123	6	35,330,421	T	C	0.94	1	PPARD
rs150249429	6	35,331,300	G	A	0.981	1	PPARD
rs60987797	6	35,331,746	T	C	0.985	0.999	PPARD
6:35332208_CT_C	6	35,332,208	CT	C	0.978	0.819	PPARD
rs73411771	6	35,332,308	T	C	0.983	1	PPARD
rs112971007	6	35,332,501	G	GT	0.911	0.893	PPARD
rs9470000	6	35,332,733	G	A	0.985	0.999	PPARD
6:35332907_CTTTTT_C	6	35,332,907	CTTTTT	C	0.968	0.991	PPARD
rs9470001	6	35,333,741	G	C	0.94	1	PPARD
rs60889636	6	35,333,754	C	T	0.971	1	PPARD
rs375114675	6	35,334,332	C	CTT	0.977	0.988	PPARD
rs112464836	6	35,335,691	C	T	0.983	0.998	PPARD
rs9658080	6	35,337,197	A	G	0.955	1	PPARD

SNP	Chromosome	Position	Effect Allele	Other Allele	Effect Allele Frequency	Imputation quality	Gene
rs9658083	6	35,337,291	G	C	0.94	1	PPARD
rs9296148	6	35,337,467	G	A	0.94	0.999	PPARD
rs12173347	6	35,339,000	G	A	0.986	0.999	PPARD
rs111815981	6	35,340,115	C	T	0.975	0.942	PPARD
rs12173582	6	35,340,300	C	T	0.986	0.999	PPARD
rs7750906	6	35,341,021	G	T	0.983	1	PPARD
rs770226172	6	35,341,450	C	CT	0.277	0.943	PPARD
rs143520309	6	35,341,494	G	A	0.981	1	PPARD
rs6919334	6	35,341,875	G	A	0.94	1	PPARD
rs112424601	6	35,342,079	C	T	0.983	1	PPARD
rs116218317	6	35,342,090	C	G	0.948	0.98	PPARD
rs372824499	6	35,342,434	C	CA	0.983	0.915	PPARD
rs147425344	6	35,342,817	T	C	0.981	1	PPARD
rs10080411	6	35,343,055	T	C	0.924	1	PPARD
rs73411781	6	35,344,374	C	T	0.98	1	PPARD
rs9658085	6	35,344,426	T	C	0.97	1	PPARD
rs571968297	6	35,344,796	C	CA	0.981	0.83	PPARD
rs9296149	6	35,345,814	T	G	0.97	1	PPARD
rs73411784	6	35,346,277	A	G	0.983	1	PPARD
rs2284196	6	35,347,077	T	C	0.924	1	PPARD
rs7754871	6	35,347,118	G	A	0.94	1	PPARD
rs75604492	6	35,347,895	T	C	0.969	0.992	PPARD
rs367754633	6	35,348,245	T	G	0.977	0.962	PPARD
rs372617389	6	35,348,257	T	G	0.93	0.89	PPARD
rs73411790	6	35,349,006	C	T	0.983	1	PPARD
rs9470007	6	35,349,739	T	C	0.94	1	PPARD
rs7770619	6	35,350,042	C	T	0.971	1	PPARD
rs73411797	6	35,350,469	G	C	0.98	1	PPARD
rs373497464	6	35,351,397	G	GT	0.949	0.965	PPARD
rs56807241	6	35,351,450	G	C	0.975	1	PPARD
rs572196784	6	35,352,640	C	T	0.971	1	PPARD
rs374398211	6	35,352,791	A	G	0.941	0.953	PPARD
rs540721872	6	35,352,839	G	C	0.97	0.977	PPARD
rs375915634	6	35,353,280	C	A	0.971	1	PPARD
rs375464767	6	35,353,346	C	A	0.983	1	PPARD
rs745859439	6	35,353,423	TA	T	0.694	0.704	PPARD
rs113127047	6	35,353,433	A	AC	0.982	0.958	PPARD
rs73411802	6	35,353,726	C	T	0.975	1	PPARD
rs115128769	6	35,353,752	G	A	0.968	0.932	PPARD
rs77554427	6	35,353,945	C	G	0.987	0.999	PPARD
rs7763692	6	35,354,116	C	T	0.86	0.789	PPARD
rs73413704	6	35,354,600	G	A	0.975	1	PPARD
rs112754776	6	35,354,831	A	T	0.983	1	PPARD
rs57648364	6	35,354,838	C	G	0.965	0.999	PPARD
rs182188717	6	35,354,980	G	A	0.98	0.986	PPARD
rs59137976	6	35,355,091	C	G	0.965	0.999	PPARD
rs780822376	6	35,355,493	CT	C	0.836	0.919	PPARD
rs61116413	6	35,355,735	A	G	0.983	0.999	PPARD
rs140680201	6	35,355,792	C	A	0.981	0.997	PPARD
6:35355802_CT_C	6	35,355,802	CT	C	0.854	0.666	PPARD
rs150178349	6	35,355,867	A	G	0.988	0.904	PPARD
rs3777744	6	35,356,143	A	G	0.924	0.998	PPARD
rs9658100	6	35,356,640	T	G	0.94	0.998	PPARD
rs9296150	6	35,357,157	C	T	0.988	0.998	PPARD
rs9658103	6	35,357,249	C	T	0.959	0.99	PPARD
rs9380505	6	35,358,764	G	A	0.986	0.995	PPARD
rs13206255	6	35,359,978	G	A	0.964	0.976	PPARD
rs73413718	6	35,360,177	T	C	0.938	0.996	PPARD
rs9348974	6	35,360,614	A	T	0.986	0.994	PPARD
rs77790729	6	35,360,665	G	T	0.982	0.999	PPARD
rs11962521	6	35,361,873	T	C	0.981	0.999	PPARD
rs11962524	6	35,361,961	T	C	0.982	0.999	PPARD
rs7769719	6	35,362,525	G	A	0.953	0.999	PPARD
6:35362562_CA_C	6	35,362,562	CA	C	0.978	0.679	PPARD
rs9368865	6	35,363,736	C	T	0.986	0.991	PPARD
rs528715107	6	35,364,310	T	TA	0.964	0.776	PPARD
rs9658111	6	35,364,534	G	C	0.953	0.998	PPARD
rs9380506	6	35,366,890	A	C	0.9	0.971	PPARD
rs9380507	6	35,366,902	G	A	0.987	0.985	PPARD
rs7749165	6	35,367,049	T	C	0.181	0.998	PPARD
rs1040436	6	35,367,909	A	G	0.243	0.999	PPARD
rs72894707	6	35,368,094	C	G	0.967	0.988	PPARD
rs151330587	6	35,368,406	G	A	0.973	0.998	PPARD
rs59072066	6	35,368,853	G	A	0.973	0.998	PPARD
rs9470015	6	35,369,084	A	G	0.181	1	PPARD
6:35369346_CCAGGTCCT_C	6	35,369,346	CCAGGTCCT	C	0.973	0.999	PPARD
rs143853683	6	35,370,390	T	A	0.988	0.931	PPARD
rs148696172	6	35,370,400	C	T	0.981	1	PPARD
rs76254341	6	35,370,493	C	T	0.981	0.999	PPARD
rs2267666	6	35,370,728	A	T	0.242	1	PPARD
rs9658121	6	35,371,300	G	A	0.981	0.978	PPARD
rs9658125	6	35,371,434	C	T	0.974	1	PPARD
rs7751726	6	35,371,624	G	A	0.973	0.999	PPARD
rs9658127	6	35,372,053	G	A	0.981	0.996	PPARD
rs59875209	6	35,372,186	G	A	0.973	1	PPARD
rs151115763	6	35,373,228	T	C	0.962	0.97	PPARD
rs9470016	6	35,373,655	C	T	0.974	1	PPARD
rs6942357	6	35,373,877	G	A	0.946	0.999	PPARD
rs2038068	6	35,374,461	A	G	0.235	1	PPARD
rs2038067	6	35,374,466	G	A	0.181	1	PPARD
rs73413742	6	35,374,551	G	A	0.983	0.997	PPARD
6:35375576_TA_T	6	35,375,576	TA	T	0.957	0.756	PPARD
rs9462081	6	35,376,006	A	C	0.984	1	PPARD
rs140487082	6	35,376,207	C	G	0.981	1	PPARD
rs77934702	6	35,376,775	G	C	0.983	1	PPARD
rs72894719	6	35,376,852	C	G	0.986	0.926	PPARD
rs1003973	6	35,377,301	C	A	0.182	0.999	PPARD
rs770690203	6	35,377,341	GTGT	G	0.243	0.998	PPARD
rs9658129	6	35,377,714	A	G	0.981	1	PPARD
rs9658132	6	35,378,756	C	T	0.981	0.999	PPARD
rs9658134	6	35,378,798	G	A	0.984	0.99	PPARD
rs9658135	6	35,378,997	G	A	0.981	0.998	PPARD
rs9296151	6	35,379,770	G	A	0.965	0.999	PPARD
rs9296152	6	35,379,973	G	T	0.984	1	PPARD
rs9658142	6	35,379,998	G	A	0.869	0.978	PPARD
rs9296153	6	35,380,222	C	T	0.984	1	PPARD
rs58915043	6	35,380,919	A	C	0.984	1	PPARD
rs4713854	6	35,381,099	C	A	0.106	0.996	PPARD
rs62402061	6	35,381,192	G	A	0.919	0.936	PPARD
rs6899536	6	35,381,539	A	C	0.181	1	PPARD
rs746850352	6	35,382,137	TACC	T	0.234	0.997	PPARD
rs11389012	6	35,382,308	C	CA	0.984	1	PPARD
rs146874451	6	35,383,027	A	G	0.968	0.953	PPARD
rs150023069	6	35,383,291	T	A	0.981	0.998	PPARD
rs369825175	6	35,383,555	C	CTG	0.922	0.843	PPARD
rs145521939	6	35,383,624	T	C	0.984	0.901	PPARD
rs67056409	6	35,383,699	A	G	0.811	0.953	PPARD
rs9470020	6	35,383,957	A	G	0.168	0.999	PPARD
rs73413756	6	35,384,627	G	C	0.984	0.997	PPARD
rs146263047	6	35,385,134	G	A	0.981	0.998	PPARD
rs2299871	6	35,385,243	C	T	0.185	0.999	PPARD
rs9462082	6	35,386,041	A	G	0.169	0.999	PPARD
6:35386116_TA_T	6	35,386,116	TA	T	0.168	0.996	PPARD
rs199592359	6	35,386,487	CA	C	0.169	0.999	PPARD
rs9296154	6	35,387,378	G	A	0.984	0.999	PPARD
rs9658149	6	35,387,538	C	A	0.981	0.998	PPARD
rs7762809	6	35,387,740	T	C	0.983	0.999	PPARD
rs9658151	6	35,388,384	T	C	0.984	0.999	PPARD
rs2076169	6	35,388,479	A	G	0.892	0.994	PPARD
rs2395622	6	35,388,758	C	T	0.185	0.999	PPARD
rs2395623	6	35,389,031	T	C	0.23	0.999	PPARD
6:35389093_TTTTG_T	6	35,389,093	TTTTG	T	0.208	0.947	PPARD
rs77024784	6	35,389,097	G	T	0.907	0.814	PPARD
rs572502812	6	35,389,112	A	G	0.973	0.689	PPARD
rs147854177	6	35,389,217	C	T	0.981	0.994	PPARD
rs148895228	6	35,389,315	C	T	0.985	0.948	PPARD
rs141964480	6	35,390,631	G	A	0.987	0.953	PPARD
rs188504499	6	35,393,005	C	T	0.981	0.997	PPARD
rs181687969	6	35,393,009	C	A	0.981	0.997	PPARD
rs2076166	6	35,393,404	A	G	0.169	0.999	PPARD
rs9658167	6	35,394,080	G	A	0.986	0.94	PPARD
rs9658170	6	35,394,504	G	A	0.981	0.995	PPARD
rs9658172	6	35,394,610	G	A	0.987	0.913	PPARD
rs3734254	6	35,395,010	C	T	0.207	0.997	PPARD
rs1053049	6	35,395,618	C	T	0.234	0.995	PPARD
rs145364349	6	45,296,339	A	G	0.988	0.947	RUNX2
rs12205523	6	45,296,618	T	C	0.674	0.999	RUNX2
rs114414163	6	45,296,987	T	C	0.979	0.999	RUNX2
rs151007996	6	45,297,163	G	C	0.985	0.867	RUNX2
rs12201899	6	45,298,204	C	A	0.674	0.999	RUNX2
rs17288278	6	45,298,482	G	A	0.494	0.999	RUNX2
rs58769170	6	45,298,531	C	T	0.674	0.999	RUNX2
rs73735374	6	45,298,648	T	A	0.865	0.989	RUNX2
rs73735375	6	45,298,649	C	T	0.865	0.989	RUNX2
rs11968157	6	45,298,863	T	C	0.949	0.689	RUNX2

SNP	Chromosome	Position	Effect Allele	Other Allele	Effect Allele Frequency	Imputation quality	Gene
rs2021272	6	45,298,865	C	T	0.893	0.774	RUNX2
rs202179785	6	45,298,866	G	T	0.884	0.998	RUNX2
rs145324768	6	45,298,931	T	C	0.986	0.944	RUNX2
rs10948226	6	45,299,174	G	C	0.674	0.999	RUNX2
rs77404483	6	45,299,328	T	C	0.968	0.999	RUNX2
rs556998786	6	45,299,669	G	A	0.988	0.865	RUNX2
rs73444672	6	45,299,981	C	T	0.907	0.999	RUNX2
rs150948607	6	45,300,083	G	GTA	0.673	0.997	RUNX2
rs115695595	6	45,300,097	A	T	0.95	0.995	RUNX2
rs113847277	6	45,300,279	T	A	0.989	0.998	RUNX2
rs16873345	6	45,300,396	T	C	0.863	0.999	RUNX2
rs115819060	6	45,300,671	T	C	0.979	0.999	RUNX2
rs113692157	6	45,300,691	G	A	0.968	0.999	RUNX2
rs34677014	6	45,300,951	T	A	0.86	0.999	RUNX2
rs28360524	6	45,301,070	A	G	0.863	0.999	RUNX2
rs77375322	6	45,301,788	T	C	0.957	0.998	RUNX2
rs73444674	6	45,301,805	A	G	0.907	0.999	RUNX2
rs116482918	6	45,301,851	C	A	0.979	0.999	RUNX2
rs75324063	6	45,301,917	T	C	0.957	0.998	RUNX2
rs143308713	6	45,302,186	CA	C	0.482	0.918	RUNX2
rs779193161	6	45,302,528	AC	A	0.908	0.997	RUNX2
rs140332155	6	45,302,548	T	C	0.989	0.998	RUNX2
rs7757676	6	45,302,635	G	T	0.884	0.999	RUNX2
rs7757282	6	45,302,654	A	G	0.537	0.999	RUNX2
rs12199256	6	45,303,493	T	G	0.677	0.999	RUNX2
rs12524323	6	45,303,853	C	A	0.558	0.999	RUNX2
rs11412392	6	45,303,920	C	CT	0.881	0.999	RUNX2
rs114764157	6	45,304,325	T	C	0.968	0.999	RUNX2
rs191813108	6	45,304,566	C	T	0.989	0.998	RUNX2
rs185827092	6	45,305,594	T	C	0.979	0.999	RUNX2
6:45306081_TA_T	6	45,306,081	TA	T	0.557	0.991	RUNX2
rs181022355	6	45,306,093	A	T	0.957	0.998	RUNX2
rs10948227	6	45,306,107	C	G	0.86	0.999	RUNX2
rs10948228	6	45,306,232	T	C	0.86	0.999	RUNX2
rs10948229	6	45,306,371	A	G	0.494	0.999	RUNX2
rs967588	6	45,306,471	C	T	0.86	0.999	RUNX2
rs11963924	6	45,306,562	C	T	0.677	0.999	RUNX2
rs74773773	6	45,307,379	T	C	0.957	0.997	RUNX2
rs12199720	6	45,307,443	C	T	0.404	0.998	RUNX2
rs12199871	6	45,307,682	C	T	0.674	0.999	RUNX2
rs12214749	6	45,307,906	A	G	0.674	0.999	RUNX2
rs71566529	6	45,308,035	A	G	0.987	0.999	RUNX2
rs150815691	6	45,308,307	C	T	0.979	0.999	RUNX2
rs10948230	6	45,308,446	A	G	0.884	0.999	RUNX2
rs190134956	6	45,308,522	C	T	0.989	0.998	RUNX2
rs73735378	6	45,309,206	T	C	0.86	0.999	RUNX2
rs7760112	6	45,309,632	T	G	0.862	0.998	RUNX2
rs144071310	6	45,309,744	A	C	0.959	0.999	RUNX2
rs772521484	6	45,309,802	AC	A	0.884	0.997	RUNX2
rs111469485	6	45,310,155	C	T	0.952	0.986	RUNX2
rs12205312	6	45,310,265	C	G	0.401	0.999	RUNX2
rs12205468	6	45,310,285	G	A	0.674	0.999	RUNX2
rs748617764	6	45,310,477	TTAA	T	0.884	0.998	RUNX2
rs75077187	6	45,310,891	C	T	0.968	0.999	RUNX2
6:45310984_CA_C	6	45,310,984	CA	C	0.968	0.997	RUNX2
rs112675986	6	45,311,828	G	A	0.989	0.996	RUNX2
rs115410240	6	45,311,927	G	A	0.985	0.982	RUNX2
rs113438193	6	45,312,842	A	G	0.989	0.998	RUNX2
rs36043738	6	45,313,149	C	T	0.986	0.998	RUNX2
rs16873348	6	45,313,744	T	C	0.881	0.999	RUNX2
6:45313909_ATT_A	6	45,313,909	ATT	A	0.677	0.999	RUNX2
rs10456552	6	45,313,952	G	A	0.494	0.999	RUNX2
rs563075667	6	45,314,304	C	CT	0.587	0.965	RUNX2
rs12529295	6	45,315,328	T	C	0.86	0.999	RUNX2
rs16873351	6	45,315,350	G	C	0.907	0.999	RUNX2
rs201547368	6	45,315,495	C	CT	0.957	0.989	RUNX2
rs192736168	6	45,315,504	A	T	0.978	0.777	RUNX2
rs748967700	6	45,315,535	CTGTT	C	0.907	0.995	RUNX2
rs80130487	6	45,315,573	A	G	0.941	0.976	RUNX2
rs9472464	6	45,315,745	T	C	0.908	0.998	RUNX2
rs2396504	6	45,316,202	C	G	0.538	0.999	RUNX2
rs9463085	6	45,316,247	T	A	0.406	0.993	RUNX2
rs7748505	6	45,316,249	C	A	0.406	0.993	RUNX2
rs747766590	6	45,316,411	CA	C	0.866	0.75	RUNX2
rs76626857	6	45,316,668	C	A	0.957	0.998	RUNX2
rs10498759	6	45,316,721	A	T	0.907	0.999	RUNX2
rs76338915	6	45,317,163	T	C	0.957	0.998	RUNX2
rs2024787	6	45,318,002	A	T	0.86	0.999	RUNX2
rs78700141	6	45,318,425	C	T	0.957	0.998	RUNX2
rs2024786	6	45,318,721	A	G	0.881	0.999	RUNX2
rs76850596	6	45,319,681	C	T	0.968	0.998	RUNX2
rs6934594	6	45,319,876	C	T	0.401	0.999	RUNX2
rs145864433	6	45,319,994	A	G	0.989	0.997	RUNX2
rs74330972	6	45,320,091	G	A	0.979	0.999	RUNX2
6:45320492_GTA_G	6	45,320,492	GTA	G	0.884	0.997	RUNX2
rs7770401	6	45,320,500	A	G	0.387	0.999	RUNX2
rs12194682	6	45,320,520	T	A	0.675	0.999	RUNX2
rs116618716	6	45,320,849	T	C	0.968	0.998	RUNX2
rs142901204	6	45,321,077	T	C	0.989	0.998	RUNX2
rs114089085	6	45,321,138	T	C	0.968	0.998	RUNX2
rs17209769	6	45,321,205	C	A	0.677	0.999	RUNX2
rs62400330	6	45,321,455	G	A	0.677	0.999	RUNX2
rs72860185	6	45,321,462	A	G	0.975	0.988	RUNX2
rs9472465	6	45,321,546	T	C	0.907	0.999	RUNX2
rs115661112	6	45,321,734	A	G	0.979	0.999	RUNX2
rs6926638	6	45,322,013	T	C	0.881	0.999	RUNX2
rs141281860	6	45,322,062	T	C	0.985	0.954	RUNX2
rs6458440	6	45,322,223	C	T	0.863	0.999	RUNX2
rs761505983	6	45,322,281	TCA	T	0.908	0.998	RUNX2
6:45322367_TAC_T	6	45,322,367	TAC	T	0.747	0.891	RUNX2
rs75211739	6	45,322,377	C	T	0.986	0.579	RUNX2
rs73735386	6	45,322,383	C	T	0.863	0.998	RUNX2
rs13191493	6	45,322,891	T	C	0.986	0.997	RUNX2
rs745720898	6	45,323,154	AT	A	0.863	0.998	RUNX2
rs112514252	6	45,323,938	T	C	0.989	0.997	RUNX2
rs556384426	6	45,324,307	A	AT	0.977	0.96	RUNX2
rs12211923	6	45,324,344	G	A	0.677	1	RUNX2
rs111646702	6	45,324,836	T	C	0.968	0.998	RUNX2
rs9472466	6	45,324,844	C	T	0.907	0.999	RUNX2
rs12213644	6	45,324,893	G	T	0.677	1	RUNX2
rs6932804	6	45,324,991	A	G	0.537	1	RUNX2
rs112908827	6	45,325,039	C	T	0.957	0.998	RUNX2
rs6933449	6	45,325,151	G	C	0.494	0.999	RUNX2
6:45325186_GC_G	6	45,325,186	GC	G	0.677	0.998	RUNX2
rs4711814	6	45,326,051	T	C	0.881	0.999	RUNX2
rs9472468	6	45,326,114	C	T	0.908	0.998	RUNX2
rs114471738	6	45,326,544	G	A	0.979	0.999	RUNX2
rs1406848	6	45,326,862	T	C	0.86	0.999	RUNX2
rs6458441	6	45,327,401	G	T	0.401	0.999	RUNX2
rs140372065	6	45,327,692	A	G	0.979	0.999	RUNX2
rs16873364	6	45,328,157	G	C	0.884	0.999	RUNX2
rs11966878	6	45,328,265	A	G	0.674	0.999	RUNX2
rs77933458	6	45,328,407	T	C	0.957	0.998	RUNX2
rs9472470	6	45,328,947	T	C	0.907	0.999	RUNX2
rs12194311	6	45,329,048	C	A	0.494	0.999	RUNX2
rs16873366	6	45,329,193	A	G	0.881	0.999	RUNX2
rs12194628	6	45,329,376	G	C	0.677	1	RUNX2
rs1852986	6	45,329,489	C	G	0.494	0.999	RUNX2
rs1852985	6	45,329,656	C	T	0.881	0.999	RUNX2
rs12203466	6	45,330,263	T	A	0.677	1	RUNX2
rs560040870	6	45,330,580	G	GT	0.908	0.996	RUNX2
rs202016666	6	45,330,670	T	TTTAA	0.978	0.934	RUNX2
rs76333979	6	45,330,738	G	C	0.959	0.965	RUNX2
rs114502565	6	45,330,788	A	C	0.979	0.999	RUNX2
rs142025540	6	45,330,821	A	C	0.95	0.996	RUNX2
rs35113874	6	45,330,892	A	G	0.934	0.975	RUNX2
rs10807321	6	45,330,974	T	C	0.677	1	RUNX2
rs74483478	6	45,331,062	G	A	0.968	0.998	RUNX2
rs12663469	6	45,331,469	T	A	0.86	0.999	RUNX2
rs12205642	6	45,331,765	T	C	0.677	1	RUNX2
6:45331851_CA_C	6	45,331,851	CA	C	0.393	0.976	RUNX2
rs775303086	6	45,332,282	CTATG	C	0.907	0.997	RUNX2
rs6932926	6	45,332,305	A	G	0.884	0.999	RUNX2
rs2295296	6	45,333,184	C	T	0.863	0.999	RUNX2
rs3057619	6	45,333,621	T	TATG	0.493	0.998	RUNX2
rs145210594	6	45,333,727	T	C	0.979	0.972	RUNX2
rs138361696	6	45,333,731	T	C	0.979	0.974	RUNX2
6:45334107_TA_T	6	45,334,107	TA	T	0.677	0.999	RUNX2
rs16873373	6	45,335,701	T	G	0.881	0.999	RUNX2
rs760871272	6	45,335,826	TAC	T	0.883	0.999	RUNX2
rs773463619	6	45,336,723	GTCAA	G	0.86	0.997	RUNX2
rs111537379	6	45,337,450	C	G	0.989	0.998	RUNX2
rs142162695	6	45,337,632	C	A	0.989	0.998	RUNX2
rs115645449	6	45,337,784	G	T	0.985	0.97	RUNX2
rs112607097	6	45,337,936	G	T	0.989	0.998	RUNX2

SNP	Chromosome	Position	Effect Allele	Other Allele	Effect Allele Frequency	Imputation quality	Gene
rs79840815	6	45,337,982	G	A	0.968	0.999	RUNX2
rs75969313	6	45,338,632	C	T	0.979	1	RUNX2
rs17288320	6	45,338,703	T	C	0.703	0.998	RUNX2
rs9472472	6	45,339,067	T	C	0.908	0.998	RUNX2
rs114173323	6	45,339,097	A	G	0.984	0.962	RUNX2
rs76298881	6	45,339,181	T	G	0.968	0.998	RUNX2
rs16873379	6	45,339,851	T	C	0.881	0.999	RUNX2
rs72862907	6	45,339,943	C	T	0.972	0.99	RUNX2
rs6928753	6	45,340,070	T	C	0.494	0.999	RUNX2
rs112086400	6	45,340,073	T	C	0.957	0.998	RUNX2
rs7744673	6	45,340,400	A	G	0.907	0.999	RUNX2
rs74867681	6	45,341,284	G	T	0.979	1	RUNX2
rs111928541	6	45,341,391	G	A	0.95	0.996	RUNX2
rs12208924	6	45,342,051	C	T	0.537	0.999	RUNX2
rs12216308	6	45,342,305	T	G	0.677	1	RUNX2
6:45342394_CATAA_C	6	45,342,394	CATAA	C	0.916	0.954	RUNX2
rs138295153	6	45,342,454	A	G	0.978	0.993	RUNX2
rs148734537	6	45,342,672	G	A	0.982	0.954	RUNX2
rs150263698	6	45,343,335	G	GC	0.904	0.992	RUNX2
rs4714853	6	45,343,871	T	C	0.86	1	RUNX2
rs77934227	6	45,344,004	T	C	0.957	0.998	RUNX2
rs140230433	6	45,344,647	T	C	0.979	1	RUNX2
6:45344837_TCA_T	6	45,344,837	TCA	T	0.679	0.997	RUNX2
rs12210230	6	45,344,854	T	A	0.678	1	RUNX2
rs150659824	6	45,345,207	C	T	0.979	1	RUNX2
rs56125317	6	45,345,357	A	T	0.975	0.989	RUNX2
rs73444688	6	45,345,480	C	T	0.907	0.998	RUNX2
rs532035127	6	45,345,682	T	TG	0.96	0.982	RUNX2
rs2295295	6	45,345,731	T	G	0.881	1	RUNX2
rs139918944	6	45,346,066	A	G	0.985	0.955	RUNX2
rs114072667	6	45,346,156	G	A	0.979	1	RUNX2
rs113638915	6	45,346,565	C	A	0.989	0.999	RUNX2
rs76970282	6	45,346,835	C	T	0.933	0.998	RUNX2
rs3763190	6	45,347,644	G	A	0.881	0.999	RUNX2
rs62400335	6	45,347,782	G	A	0.677	1	RUNX2
rs184650002	6	45,348,537	C	T	0.979	1	RUNX2
rs11968637	6	45,348,633	G	A	0.677	0.999	RUNX2
rs9472477	6	45,349,509	A	G	0.949	0.997	RUNX2
rs4510673	6	45,350,107	A	G	0.401	0.999	RUNX2
rs112627708	6	45,350,890	G	A	0.974	0.946	RUNX2
rs77189473	6	45,350,963	G	T	0.979	1	RUNX2
rs79270087	6	45,351,369	A	G	0.964	0.992	RUNX2
rs200974406	6	45,351,371	A	AT	0.979	1	RUNX2
rs17288334	6	45,351,391	A	G	0.493	0.999	RUNX2
rs201855052	6	45,351,528	A	ATGT	0.979	0.999	RUNX2
rs199865896	6	45,351,530	A	AT	0.979	0.999	RUNX2
rs201028238	6	45,351,531	A	AT	0.979	0.999	RUNX2
rs528121345	6	45,351,532	G	T	0.979	0.999	RUNX2
rs116075671	6	45,351,606	T	G	0.979	1	RUNX2
rs115192108	6	45,351,751	T	C	0.979	0.999	RUNX2
rs76835605	6	45,351,896	T	C	0.979	1	RUNX2
rs73441910	6	45,352,011	T	G	0.949	0.997	RUNX2
rs76451089	6	45,352,015	G	T	0.879	0.995	RUNX2
rs138412886	6	45,352,031	C	G	0.979	1	RUNX2
rs182151942	6	45,352,611	A	T	0.968	0.997	RUNX2
rs113898207	6	45,352,999	C	T	0.957	0.998	RUNX2
rs139953256	6	45,353,032	C	T	0.964	0.991	RUNX2
rs12209727	6	45,353,100	C	T	0.494	0.999	RUNX2
rs62400336	6	45,353,430	T	A	0.494	0.998	RUNX2
rs12525950	6	45,354,814	A	G	0.881	0.996	RUNX2
rs1321081	6	45,355,609	T	C	0.401	0.994	RUNX2
rs10948231	6	45,355,824	T	C	0.881	0.994	RUNX2
rs148931785	6	45,356,026	G	A	0.978	0.989	RUNX2
rs112982128	6	45,356,210	G	A	0.968	0.983	RUNX2
rs115897336	6	45,356,300	G	A	0.979	0.992	RUNX2
rs113230441	6	45,356,676	C	G	0.968	0.978	RUNX2
rs717987	6	45,356,983	A	G	0.88	0.989	RUNX2
rs2396502	6	45,357,699	A	C	0.398	0.984	RUNX2
rs770307776	6	45,357,742	CA	C	0.191	0.893	RUNX2
6:45357980_TG_T	6	45,357,980	TG	T	0.678	0.979	RUNX2
rs6908650	6	45,358,107	G	A	0.547	0.975	RUNX2
rs1321080	6	45,358,323	G	T	0.73	0.993	RUNX2
rs1321079	6	45,358,403	T	A	0.736	0.996	RUNX2
rs11966902	6	45,358,413	A	G	0.883	0.993	RUNX2
rs9472482	6	45,358,429	G	T	0.949	0.99	RUNX2
rs11966930	6	45,358,565	A	T	0.953	0.992	RUNX2
rs190757416	6	45,358,923	A	G	0.989	0.958	RUNX2
rs12201563	6	45,359,394	T	C	0.737	0.996	RUNX2
rs11968177	6	45,359,876	A	C	0.882	0.994	RUNX2
rs9395099	6	45,360,194	C	T	0.593	0.988	RUNX2
rs77329398	6	45,360,433	T	C	0.882	0.994	RUNX2
6:45360444_AT_A	6	45,360,444	AT	A	0.116	0.897	RUNX2
rs12211510	6	45,361,189	A	G	0.736	0.997	RUNX2
rs111514840	6	45,361,210	C	T	0.882	0.994	RUNX2
rs138064235	6	45,361,260	A	G	0.883	0.992	RUNX2
rs149513790	6	45,361,291	C	T	0.884	0.985	RUNX2
rs144011454	6	45,361,298	C	T	0.884	0.986	RUNX2
rs148241428	6	45,361,304	C	T	0.955	0.975	RUNX2
rs56092589	6	45,361,312	G	A	0.971	0.981	RUNX2
rs191086690	6	45,361,674	T	C	0.979	0.932	RUNX2
rs151005683	6	45,361,838	C	T	0.737	0.997	RUNX2
rs57013196	6	45,361,874	C	T	0.882	0.994	RUNX2
rs72862930	6	45,361,883	C	T	0.737	0.997	RUNX2
rs1575060	6	45,362,055	T	C	0.619	0.995	RUNX2
rs111425365	6	45,363,556	G	C	0.903	1	RUNX2
rs10498760	6	45,363,796	C	A	0.903	1	RUNX2
rs10948233	6	45,364,096	A	T	0.737	0.998	RUNX2
rs75142593	6	45,364,117	T	C	0.903	1	RUNX2
rs72862932	6	45,364,175	C	A	0.976	0.97	RUNX2
rs115418113	6	45,364,270	A	G	0.903	1	RUNX2
rs111750022	6	45,364,879	C	T	0.903	1	RUNX2
rs1852984	6	45,364,940	A	G	0.737	0.998	RUNX2
rs1852983	6	45,365,106	A	G	0.737	0.998	RUNX2
rs11963804	6	45,365,928	G	A	0.903	1	RUNX2
rs11966462	6	45,366,207	T	C	0.903	1	RUNX2
rs750754420	6	45,366,728	GTTTACAAAAGCAAGC	G	0.948	0.981	RUNX2
rs17288355	6	45,366,946	A	G	0.903	0.999	RUNX2
rs113198989	6	45,367,025	C	T	0.989	0.988	RUNX2
rs111517073	6	45,367,128	G	A	0.903	0.998	RUNX2
rs149231830	6	45,367,326	A	G	0.989	0.871	RUNX2
rs148968352	6	45,367,496	A	AAAGG	0.668	0.993	RUNX2
rs72862937	6	45,367,617	T	C	0.638	0.998	RUNX2
6:45367779_GAAGGA_G	6	45,367,779	GAAGGA	G	0.922	0.854	RUNX2
rs548399771	6	45,367,786	A	G	0.989	0.675	RUNX2
rs62400349	6	45,367,922	A	T	0.74	0.982	RUNX2
rs62400350	6	45,367,923	T	G	0.735	0.996	RUNX2
rs1321078	6	45,368,214	G	T	0.735	0.998	RUNX2
rs61467394	6	45,368,456	T	G	0.903	0.999	RUNX2
rs9296459	6	45,368,797	G	A	0.637	0.997	RUNX2
rs767861798	6	45,370,884	GA	G	0.948	0.988	RUNX2
rs58571027	6	45,370,897	A	C	0.903	0.999	RUNX2
rs7749637	6	45,371,166	C	T	0.636	0.997	RUNX2
rs10456553	6	45,371,222	A	G	0.735	0.998	RUNX2
rs9472487	6	45,371,366	G	T	0.577	0.99	RUNX2
rs10456125	6	45,371,426	G	T	0.988	0.928	RUNX2
rs7770471	6	45,371,553	T	A	0.637	0.997	RUNX2
rs2396501	6	45,372,012	G	A	0.084	0.984	RUNX2
rs546454766	6	45,372,022	A	C	0.988	0.862	RUNX2
rs113901838	6	45,372,053	G	GA	0.637	0.993	RUNX2
rs17288376	6	45,372,218	G	A	0.903	0.998	RUNX2
rs79446501	6	45,372,276	G	T	0.968	0.962	RUNX2
rs112522132	6	45,372,842	G	A	0.989	0.989	RUNX2
rs199677146	6	45,373,532	A	AT	0.903	0.998	RUNX2
rs17209846	6	45,373,843	T	A	0.903	0.999	RUNX2
rs112400692	6	45,373,907	G	A	0.989	0.987	RUNX2
rs145328254	6	45,374,128	A	G	0.986	0.971	RUNX2
rs1997995	6	45,374,183	A	G	0.639	0.997	RUNX2
rs1977152	6	45,374,418	G	A	0.903	0.999	RUNX2
rs10948234	6	45,374,608	T	A	0.639	0.998	RUNX2
rs17209853	6	45,374,699	A	T	0.903	0.999	RUNX2
rs7742209	6	45,374,933	C	T	0.922	0.972	RUNX2
rs77735520	6	45,375,224	A	G	0.914	0.997	RUNX2
rs1321077	6	45,375,657	A	T	0.073	0.992	RUNX2
6:45375868_GA_G	6	45,375,868	GA	G	0.738	0.997	RUNX2
rs111264121	6	45,376,764	T	C	0.989	0.985	RUNX2
rs2064630	6	45,376,856	G	C	0.496	0.997	RUNX2
rs78488630	6	45,376,859	T	C	0.903	0.999	RUNX2
rs2064629	6	45,377,038	G	A	0.083	0.994	RUNX2
rs139143160	6	45,377,438	A	G	0.989	0.914	RUNX2
rs75864224	6	45,377,777	A	G	0.903	0.999	RUNX2
rs75988681	6	45,377,848	C	T	0.903	0.999	RUNX2
rs79805935	6	45,378,092	A	G	0.914	0.997	RUNX2
rs79359769	6	45,378,973	A	G	0.903	0.999	RUNX2
rs111760536	6	45,379,839	C	T	0.989	0.986	RUNX2
rs35549153	6	45,379,924	C	CA	0.338	0.863	RUNX2

SNP	Chromosome	Position	Effect Allele	Other Allele	Effect Allele Frequency	Imputation quality	Gene
rs13215406	6	45,380,124	A	C	0.851	0.965	RUNX2
rs549531306	6	45,380,411	C	CT	0.94	0.942	RUNX2
rs2064628	6	45,380,531	T	C	0.737	0.999	RUNX2
rs11962735	6	45,380,558	T	C	0.882	0.944	RUNX2
rs113673672	6	45,380,647	T	G	0.903	0.999	RUNX2
rs76954735	6	45,381,082	A	G	0.903	0.999	RUNX2
rs4413613	6	45,381,312	T	C	0.903	0.998	RUNX2
rs149334497	6	45,381,316	C	T	0.985	0.931	RUNX2
rs4413614	6	45,381,328	T	C	0.903	0.999	RUNX2
rs4370348	6	45,381,399	C	T	0.903	0.999	RUNX2
rs17209874	6	45,381,584	G	A	0.903	0.999	RUNX2
rs77252578	6	45,381,770	T	A	0.903	0.999	RUNX2
rs1004130	6	45,382,093	C	T	0.903	0.999	RUNX2
rs1467458	6	45,383,078	A	C	0.083	0.995	RUNX2
rs17288390	6	45,384,018	T	C	0.64	0.999	RUNX2
rs11964020	6	45,384,134	A	G	0.988	0.955	RUNX2
rs75205636	6	45,384,467	C	A	0.903	0.999	RUNX2
6:45385059_AT_A	6	45,385,059	AT	A	0.903	0.998	RUNX2
rs7768466	6	45,385,439	C	T	0.903	1	RUNX2
rs34783931	6	45,385,512	G	A	0.964	0.998	RUNX2
rs7768666	6	45,385,607	A	C	0.903	1	RUNX2
rs17288397	6	45,385,742	G	A	0.903	1	RUNX2
rs34787815	6	45,385,910	T	TAAC	0.134	0.995	RUNX2
rs79608764	6	45,386,202	T	C	0.948	0.993	RUNX2
rs74872917	6	45,387,663	G	A	0.903	0.999	RUNX2
rs62400353	6	45,387,899	G	A	0.738	1	RUNX2
6:45389142_CT_C	6	45,389,142	CT	C	0.903	0.997	RUNX2
rs7751427	6	45,389,234	A	G	0.903	0.999	RUNX2
rs7771980	6	45,389,289	T	C	0.903	0.999	RUNX2
rs146028707	6	45,390,088	G	T	0.977	0.938	RUNX2
rs557997453	6	45,390,233	A	C	0.961	0.659	RUNX2
rs6921145	6	45,390,511	G	A	0.903	0.998	RUNX2
rs3749864	6	45,390,733	G	C	0.903	0.998	RUNX2
rs11498195	6	45,390,949	G	C	0.903	0.998	RUNX2
rs11498196	6	45,390,962	C	T	0.585	0.982	RUNX2
rs3805810	6	45,391,284	C	G	0.923	0.971	RUNX2
rs3840375	6	45,391,336	C	CG	0.903	0.998	RUNX2
6:45391425_AG_A	6	45,391,425	AG	A	0.922	0.954	RUNX2
rs3805811	6	45,391,654	G	C	0.903	0.998	RUNX2
rs1321076	6	45,391,860	A	G	0.136	1	RUNX2
rs3792952	6	45,391,971	C	T	0.903	0.998	RUNX2
rs113308638	6	45,392,296	G	T	0.951	0.981	RUNX2
rs3828722	6	45,392,982	A	G	0.903	0.998	RUNX2
rs3828723	6	45,393,010	A	G	0.903	0.998	RUNX2
rs2894641	6	45,393,174	A	G	0.738	0.998	RUNX2
rs9369561	6	45,393,362	G	A	0.496	0.997	RUNX2
rs9395101	6	45,393,363	T	C	0.498	0.986	RUNX2
rs116515803	6	45,393,606	G	A	0.988	0.958	RUNX2
rs12192267	6	45,393,787	C	T	0.738	0.998	RUNX2
rs9472489	6	45,393,895	A	G	0.947	0.993	RUNX2
rs75870189	6	45,394,252	C	T	0.903	0.998	RUNX2
rs13437231	6	45,394,741	T	C	0.947	0.993	RUNX2
rs2224118	6	45,395,138	T	G	0.496	0.996	RUNX2
rs2819860	6	45,395,789	A	C	0.136	0.997	RUNX2
rs2677105	6	45,395,792	A	C	0.136	0.996	RUNX2
rs2677104	6	45,395,873	C	T	0.136	0.999	RUNX2
rs3805818	6	45,395,920	A	G	0.903	0.997	RUNX2
rs78645638	6	45,396,182	G	A	0.903	0.997	RUNX2
rs2819859	6	45,396,297	A	C	0.534	0.995	RUNX2
rs2819858	6	45,396,319	A	T	0.534	0.995	RUNX2
rs71566532	6	45,396,574	G	A	0.963	0.998	RUNX2
rs66878438	6	45,396,575	C	CT	0.556	0.868	RUNX2
rs565953603	6	45,396,592	T	C	0.953	0.962	RUNX2
rs112692947	6	45,396,996	C	T	0.989	0.979	RUNX2
rs2677102	6	45,397,050	C	T	0.533	0.996	RUNX2
rs2819857	6	45,397,558	C	T	0.533	0.996	RUNX2
rs112084677	6	45,397,605	C	T	0.96	0.948	RUNX2
rs147540671	6	45,397,646	C	T	0.971	0.927	RUNX2
rs59397804	6	45,397,695	G	A	0.903	0.997	RUNX2
rs2677101	6	45,398,157	C	T	0.533	0.996	RUNX2
rs12203052	6	45,398,958	C	G	0.738	0.997	RUNX2
rs78417039	6	45,399,017	G	A	0.903	0.997	RUNX2
rs2819856	6	45,399,458	A	G	0.969	0.936	RUNX2
rs17209881	6	45,400,146	A	G	0.903	0.995	RUNX2
rs2819855	6	45,400,394	A	G	0.444	0.993	RUNX2
rs1406846	6	45,400,940	T	A	0.496	0.992	RUNX2
rs114012879	6	45,401,200	T	C	0.981	0.935	RUNX2
6:45401227_CT_C	6	45,401,227	CT	C	0.74	0.98	RUNX2
rs9463087	6	45,401,698	G	A	0.947	0.983	RUNX2
rs17209895	6	45,402,445	T	C	0.727	0.985	RUNX2
rs143762356	6	45,402,553	G	A	0.988	0.91	RUNX2
rs140838642	6	45,403,374	C	T	0.911	0.972	RUNX2
rs17209902	6	45,403,562	C	G	0.976	0.952	RUNX2
rs2677108	6	45,403,774	C	T	0.397	0.971	RUNX2
rs113166183	6	45,404,089	A	AT	0.949	0.941	RUNX2
rs73441953	6	45,404,102	T	C	0.96	0.922	RUNX2
rs74587175	6	45,404,170	G	A	0.948	0.953	RUNX2
rs1997992	6	45,404,230	C	T	0.488	0.967	RUNX2
rs2819854	6	45,404,528	A	G	0.479	0.942	RUNX2
rs17288411	6	45,405,134	C	A	0.877	0.823	RUNX2
rs115354589	6	45,406,914	G	C	0.983	0.911	RUNX2
rs2677109	6	45,407,654	A	G	0.349	0.984	RUNX2
rs112552471	6	45,408,316	T	C	0.969	0.88	RUNX2
rs35842341	6	45,408,389	A	AT	0.348	0.977	RUNX2
rs200335909	6	45,408,403	T	A	0.977	0.678	RUNX2
rs6458443	6	45,408,440	T	G	0.348	0.985	RUNX2
rs142644523	6	45,408,831	G	A	0.983	0.907	RUNX2
rs75705520	6	45,410,260	T	C	0.975	0.969	RUNX2
rs910586	6	45,410,312	T	C	0.362	0.983	RUNX2
rs77519622	6	45,411,621	T	C	0.987	0.944	RUNX2
rs143073147	6	45,411,780	A	G	0.98	0.928	RUNX2
rs2819853	6	45,412,657	G	A	0.363	0.984	RUNX2
rs9472491	6	45,413,479	G	A	0.945	0.973	RUNX2
rs12216037	6	45,414,845	A	G	0.982	0.935	RUNX2
rs72862982	6	45,415,376	T	C	0.971	0.842	RUNX2
rs765724	6	45,417,118	T	C	0.363	0.987	RUNX2
rs115410542	6	45,417,358	A	G	0.989	0.898	RUNX2
rs2819852	6	45,417,663	C	A	0.363	0.987	RUNX2
rs2256819	6	45,418,402	C	T	0.363	0.988	RUNX2
rs140701658	6	45,418,752	G	T	0.986	0.911	RUNX2
rs2820339	6	45,419,110	C	G	0.365	0.987	RUNX2
rs2819867	6	45,419,428	A	C	0.363	0.988	RUNX2
rs2677099	6	45,419,922	T	C	0.363	0.988	RUNX2
rs2677100	6	45,420,847	C	T	0.363	0.988	RUNX2
rs2819863	6	45,421,048	G	C	0.109	0.92	RUNX2
rs1343799	6	45,421,630	C	T	0.363	0.988	RUNX2
rs147081589	6	45,422,199	C	G	0.989	0.883	RUNX2
rs2820340	6	45,424,498	G	A	0.364	0.989	RUNX2
rs2819861	6	45,424,940	A	G	0.36	0.987	RUNX2
rs532525833	6	45,425,759	AT	A	0.841	0.747	RUNX2
rs146272391	6	45,427,240	G	A	0.988	0.907	RUNX2
rs35405209	6	45,428,508	T	TA	0.641	0.974	RUNX2
rs2252718	6	45,429,139	C	A	0.65	0.992	RUNX2
rs76744369	6	45,429,489	A	G	0.985	0.822	RUNX2
rs74584915	6	45,429,621	T	C	0.974	0.977	RUNX2
rs2790100	6	45,430,336	C	A	0.65	0.993	RUNX2
rs9472492	6	45,431,260	C	T	0.98	0.918	RUNX2
rs2790101	6	45,431,372	T	A	0.011	0.851	RUNX2
rs115504581	6	45,431,652	C	T	0.974	0.979	RUNX2
6:45431928_TA_T	6	45,431,928	TA	T	0.574	0.889	RUNX2
rs148559305	6	45,431,970	C	T	0.988	0.905	RUNX2
rs2790102	6	45,432,214	G	A	0.649	0.992	RUNX2
rs2790103	6	45,432,265	C	T	0.65	0.993	RUNX2
rs9885645	6	45,434,212	C	T	0.65	0.993	RUNX2
rs144298037	6	45,434,746	C	T	0.982	0.933	RUNX2
rs56071391	6	45,434,864	T	C	0.987	0.938	RUNX2
rs2677098	6	45,436,249	C	T	0.65	0.993	RUNX2
rs2790093	6	45,437,484	A	G	0.649	0.994	RUNX2
rs2790094	6	45,438,472	A	G	0.649	0.994	RUNX2
rs2819864	6	45,438,874	T	C	0.649	0.994	RUNX2
rs2790095	6	45,439,307	C	G	0.645	0.987	RUNX2
rs2790097	6	45,439,686	A	G	0.649	0.994	RUNX2
rs1317777	6	45,441,323	T	C	0.65	0.994	RUNX2
rs114981045	6	45,441,593	T	C	0.981	0.973	RUNX2
rs2790098	6	45,442,450	T	C	0.65	0.998	RUNX2
6:45442860_TA_T	6	45,442,860	TA	T	0.621	0.935	RUNX2
rs2819865	6	45,443,176	A	G	0.321	0.998	RUNX2
rs2790099	6	45,443,349	T	C	0.65	1	RUNX2
rs35663521	6	45,444,083	G	GA	0.649	0.999	RUNX2
6:45444128_AT_A	6	45,444,128	AT	A	0.651	0.999	RUNX2
rs2819866	6	45,444,258	A	G	0.65	1	RUNX2
rs1934327	6	45,444,967	A	T	0.65	1	RUNX2
rs35064270	6	45,445,947	G	A	0.93	0.93	RUNX2
rs10214519	6	45,447,558	A	G	0.65	0.999	RUNX2
rs4714854	6	45,447,934	A	T	0.65	0.999	RUNX2

SNP	Chromosome	Position	Effect Allele	Other Allele	Effect Allele Frequency	Imputation quality	Gene
rs12210100	6	45,448,860	C	G	0.648	0.999	RUNX2
rs4711816	6	45,450,210	T	C	0.647	0.997	RUNX2
6:45451088_CT_C	6	45,451,088	CT	C	0.645	0.989	RUNX2
rs9472494	6	45,451,836	C	T	0.648	0.998	RUNX2
rs147460969	6	45,452,538	C	T	0.98	0.848	RUNX2
6:45452929_CT_C	6	45,452,929	CT	C	0.751	0.886	RUNX2
rs79966482	6	45,453,344	C	T	0.979	0.877	RUNX2
rs9463090	6	45,453,345	G	A	0.799	0.97	RUNX2
rs7756144	6	45,453,383	C	T	0.647	0.994	RUNX2
rs7756472	6	45,453,434	G	A	0.489	0.933	RUNX2
rs115587374	6	45,454,694	T	C	0.972	0.934	RUNX2
rs12197948	6	45,454,844	A	G	0.648	0.998	RUNX2
rs72864614	6	45,456,091	T	A	0.976	0.925	RUNX2
rs35441607	6	45,456,453	G	GA	0.647	0.993	RUNX2
rs12215249	6	45,456,564	G	T	0.648	0.998	RUNX2
rs2396442	6	45,456,981	T	C	0.648	0.998	RUNX2
rs73737435	6	45,458,903	C	T	0.945	0.992	RUNX2
rs34040641	6	45,459,359	GT	G	0.606	0.776	RUNX2
rs55930938	6	45,459,414	C	T	0.945	0.993	RUNX2
rs13208741	6	45,461,253	G	A	0.712	0.992	RUNX2
rs34362546	6	45,461,467	C	CTG	0.676	0.936	RUNX2
rs73737439	6	45,461,826	T	C	0.945	0.994	RUNX2
rs73737440	6	45,461,935	A	G	0.945	0.994	RUNX2
rs35653796	6	45,462,464	G	A	0.988	0.873	RUNX2
rs62400362	6	45,462,487	C	T	0.805	0.99	RUNX2
rs73737441	6	45,464,540	A	C	0.945	0.996	RUNX2
6:45464636_CTT_C	6	45,464,636	CTT	C	0.945	0.994	RUNX2
rs115283400	6	45,465,432	A	G	0.983	0.914	RUNX2
rs1934328	6	45,465,753	A	T	0.509	0.99	RUNX2
rs79102660	6	45,466,010	T	C	0.914	0.951	RUNX2
rs141254971	6	45,467,053	A	ATC	0.793	0.985	RUNX2
rs12198785	6	45,467,107	C	A	0.965	0.956	RUNX2
rs2396441	6	45,467,765	A	G	0.488	0.986	RUNX2
rs6914610	6	45,468,074	T	G	0.792	0.991	RUNX2
rs1316330	6	45,469,626	C	A	0.738	0.979	RUNX2
rs144771872	6	45,471,658	G	A	0.985	0.913	RUNX2
rs7773875	6	45,472,298	T	C	0.805	0.998	RUNX2
rs7754227	6	45,472,737	C	T	0.805	0.998	RUNX2
rs7750266	6	45,473,089	T	C	0.805	0.999	RUNX2
rs181880831	6	45,474,432	G	A	0.984	0.917	RUNX2
rs113810328	6	45,475,794	G	C	0.975	0.99	RUNX2
rs62400363	6	45,477,160	A	G	0.847	0.989	RUNX2
rs73737445	6	45,478,498	A	G	0.946	0.994	RUNX2
rs73737446	6	45,478,815	C	T	0.946	0.994	RUNX2
rs73737447	6	45,479,757	T	G	0.946	0.993	RUNX2
rs11498194	6	45,480,190	G	A	0.859	0.994	RUNX2
rs62400365	6	45,480,499	G	C	0.805	0.993	RUNX2
rs62400366	6	45,480,516	A	T	0.86	0.994	RUNX2
6:45480980_AC_A	6	45,480,980	AC	A	0.716	0.984	RUNX2
rs58268689	6	45,481,266	G	A	0.971	0.994	RUNX2
rs62400367	6	45,481,873	A	G	0.848	0.987	RUNX2
rs56790996	6	45,483,137	G	A	0.971	0.994	RUNX2
rs138464697	6	45,484,497	C	G	0.988	0.923	RUNX2
6:45484684_GT_G	6	45,484,684	GT	G	0.857	0.992	RUNX2
rs114012998	6	45,484,925	T	G	0.975	0.993	RUNX2
rs144729269	6	45,485,503	T	A	0.989	0.864	RUNX2
6:45485546_AACCCC_A	6	45,485,546	AACCCC	A	0.858	0.995	RUNX2
6:45486014_AC_A	6	45,486,014	AC	A	0.857	0.998	RUNX2
rs7771889	6	45,486,207	C	G	0.714	0.998	RUNX2
rs7754187	6	45,486,383	T	A	0.714	0.999	RUNX2
rs7772562	6	45,486,496	G	A	0.714	0.999	RUNX2
rs62400368	6	45,487,250	A	T	0.857	0.999	RUNX2
rs376345728	6	45,487,529	G	A	0.825	0.966	RUNX2
rs368479672	6	45,487,531	G	A	0.931	0.807	RUNX2
rs572021723	6	45,487,533	G	A	0.952	0.802	RUNX2
rs9463092	6	45,487,573	G	A	0.919	0.616	RUNX2
rs6905847	6	45,487,944	C	A	0.714	1	RUNX2
rs115147510	6	45,488,144	A	C	0.985	0.967	RUNX2
rs58977496	6	45,488,475	T	G	0.97	0.996	RUNX2
rs6930053	6	45,488,758	T	C	0.401	0.993	RUNX2
rs6911664	6	45,488,930	A	T	0.714	0.999	RUNX2
rs10485422	6	45,489,192	T	A	0.744	1	RUNX2
rs13200034	6	45,489,446	C	T	0.988	0.869	RUNX2
rs62400369	6	45,489,897	A	G	0.858	0.999	RUNX2
rs56146601	6	45,490,115	G	T	0.858	0.999	RUNX2
6:45490181_AC_A	6	45,490,181	AC	A	0.959	0.781	RUNX2
rs6458444	6	45,490,182	C	A	0.737	0.985	RUNX2
rs7755257	6	45,490,292	A	G	0.714	1	RUNX2
rs73448178	6	45,490,462	C	A	0.97	0.996	RUNX2
rs7756094	6	45,490,712	C	T	0.714	1	RUNX2
rs12195878	6	45,490,753	C	T	0.744	1	RUNX2
rs7760160	6	45,491,039	A	G	0.714	1	RUNX2
rs72864634	6	45,491,259	C	T	0.744	1	RUNX2
rs55910744	6	45,491,674	A	T	0.858	0.999	RUNX2
rs7766077	6	45,492,077	G	A	0.97	0.996	RUNX2
rs6932037	6	45,492,792	G	A	0.714	1	RUNX2
rs10223365	6	45,492,963	C	T	0.97	0.996	RUNX2
rs6904353	6	45,493,083	C	T	0.571	0.999	RUNX2
rs61243460	6	45,493,237	T	C	0.858	0.999	RUNX2
rs4714857	6	45,493,673	A	T	0.751	0.969	RUNX2
rs4714858	6	45,493,675	G	A	0.744	0.999	RUNX2
rs4714859	6	45,493,682	T	C	0.714	0.999	RUNX2
rs114467971	6	45,495,082	T	C	0.983	0.966	RUNX2
rs2031966	6	45,495,736	A	G	0.714	0.999	RUNX2
rs4438954	6	45,495,748	C	G	0.714	0.999	RUNX2
rs13207392	6	45,496,456	C	T	0.714	0.999	RUNX2
rs116788323	6	45,497,527	G	A	0.974	0.991	RUNX2
rs6915569	6	45,498,737	T	C	0.572	0.999	RUNX2
rs12202610	6	45,499,240	C	A	0.982	0.948	RUNX2
rs143683342	6	45,499,487	T	TA	0.863	0.981	RUNX2
rs1319069	6	45,499,511	A	G	0.858	1	RUNX2
rs35740963	6	45,499,613	A	AT	0.714	0.999	RUNX2
rs1934329	6	45,500,000	G	A	0.572	0.999	RUNX2
rs6458446	6	45,500,470	G	A	0.714	1	RUNX2
rs6458447	6	45,500,613	G	A	0.572	0.999	RUNX2
rs6905181	6	45,500,666	G	A	0.714	1	RUNX2
6:45500872_GGT_G	6	45,500,872	GGT	G	0.632	0.896	RUNX2
6:45501163_TA_T	6	45,501,163	TA	T	0.436	0.99	RUNX2
rs75874570	6	45,501,285	A	C	0.989	0.927	RUNX2
rs7748231	6	45,501,552	A	G	0.572	1	RUNX2
6:45501625_CTTTTT_C	6	45,501,625	CTTTTT	C	0.713	0.998	RUNX2
rs10948236	6	45,501,785	T	C	0.572	0.999	RUNX2
rs12208240	6	45,501,937	G	A	0.913	0.985	RUNX2
rs199684028	6	45,502,988	T	TA	0.858	0.997	RUNX2
6:45503340_CA_C	6	45,503,340	CA	C	0.569	0.989	RUNX2
rs140948411	6	45,505,123	C	T	0.985	0.834	RUNX2
rs62400374	6	45,505,920	G	C	0.859	0.998	RUNX2
rs12209785	6	45,506,122	A	G	0.744	0.998	RUNX2
rs116100221	6	45,506,705	G	C	0.974	0.97	RUNX2
rs113237859	6	45,506,737	G	A	0.971	0.862	RUNX2
rs36060587	6	45,506,948	G	GT	0.568	0.983	RUNX2
rs72864648	6	45,506,958	C	T	0.891	0.994	RUNX2
rs10948237	6	45,507,294	G	A	0.573	0.997	RUNX2
rs62400375	6	45,507,755	G	A	0.859	0.997	RUNX2
rs62400376	6	45,508,609	G	A	0.859	0.996	RUNX2
rs557111219	6	45,508,903	A	AT	0.97	0.884	RUNX2
rs1928533	6	45,509,824	A	G	0.59	0.992	RUNX2
rs142889686	6	45,509,847	G	A	0.984	0.863	RUNX2
rs112819862	6	45,509,857	C	T	0.974	0.99	RUNX2
rs12204049	6	45,510,107	C	T	0.732	0.991	RUNX2
rs10948238	6	45,511,541	C	T	0.597	0.987	RUNX2
rs13201287	6	45,511,945	G	A	0.732	0.985	RUNX2
rs12333172	6	45,512,215	C	T	0.805	0.971	RUNX2
rs148872139	6	45,512,671	C	T	0.989	0.955	RUNX2
rs143512072	6	45,512,673	A	G	0.989	0.956	RUNX2
rs75481685	6	45,513,250	C	T	0.976	0.957	RUNX2
rs13199910	6	45,513,780	T	C	0.976	0.882	RUNX2
rs11498198	6	45,515,007	G	A	0.987	0.936	RUNX2
6:45515633_GT_G	6	45,515,633	GT	G	0.873	0.971	RUNX2
rs45585135	6	45,515,641	T	G	0.873	0.971	RUNX2
rs140371943	6	45,516,576	T	C	0.981	0.884	RUNX2
6:45517739_CT_C	6	45,517,739	CT	C	0.552	0.899	RUNX2
rs1200428	6	45,518,202	G	T	0.767	0.977	RUNX2
rs62400377	6	45,518,634	A	G	0.886	0.981	RUNX2
rs148882702	6	142,454,996	C	T	0.989	0.935	GJE1
rs225607	6	142,455,130	C	T	0.54	0.998	GJE1
rs11966646	6	155,585,154	G	A	0.898	1	CLDN20
rs9397236	6	155,585,233	A	G	0.308	0.998	CLDN20
rs2985700	6	155,585,278	C	T	0.5	0.999	CLDN20
rs9480098	6	155,585,325	C	T	0.741	0.999	CLDN20
rs1012276	6	155,585,868	G	A	0.957	0.999	CLDN20
rs1041098	6	155,586,131	C	T	0.188	0.983	CLDN20
rs12524284	6	155,586,132	A	G	0.898	1	CLDN20
rs76637693	6	155,586,596	G	A	0.957	0.999	CLDN20
rs4869850	6	155,586,598	A	C	0.398	1	CLDN20

SNP	Chromosome	Position	Effect Allele	Other Allele	Effect Allele Frequency	Imputation quality	Gene
6:155586637_CAGA_C	6	155,586,637	CAGA	C	0.382	0.996	CLDN20
rs113732082	6	155,587,071	C	T	0.987	0.956	CLDN20
rs2019152	6	155,587,197	C	A	0.5	1	CLDN20
rs113817188	6	155,587,245	C	A	0.985	1	CLDN20
rs912718	6	155,587,422	A	G	0.383	0.999	CLDN20
rs912719	6	155,587,596	A	C	0.383	1	CLDN20
rs13220614	6	155,587,702	T	C	0.398	0.996	CLDN20
rs13220615	6	155,587,709	T	G	0.398	0.996	CLDN20
rs2352573	6	155,587,747	A	G	0.383	0.996	CLDN20
rs62427163	6	155,587,776	C	T	0.748	0.999	CLDN20
rs113984694	6	155,587,868	C	T	0.985	1	CLDN20
rs12665860	6	155,587,905	T	C	0.383	0.999	CLDN20
rs113819521	6	155,587,975	C	T	0.957	0.998	CLDN20
6:155588104_CT_C	6	155,588,104	CT	C	0.73	0.986	CLDN20
rs112187332	6	155,588,158	C	T	0.985	1	CLDN20
rs78813843	6	155,588,268	C	T	0.957	0.999	CLDN20
rs7741233	6	155,588,375	T	C	0.383	0.999	CLDN20
rs7775467	6	155,588,389	A	G	0.383	0.999	CLDN20
rs137901753	6	155,588,391	G	A	0.985	1	CLDN20
rs1547244	6	155,588,461	G	A	0.383	0.999	CLDN20
rs12191327	6	155,588,484	T	C	0.801	0.999	CLDN20
rs1547245	6	155,588,682	T	C	0.748	0.999	CLDN20
rs112274989	6	155,588,864	C	T	0.985	1	CLDN20
rs9384305	6	155,589,773	G	A	0.383	1	CLDN20
rs11156018	6	155,589,832	G	A	0.733	0.999	CLDN20
rs112175041	6	155,589,844	C	T	0.985	1	CLDN20
rs9384306	6	155,589,870	G	A	0.801	0.999	CLDN20
rs9397795	6	155,589,893	C	T	0.65	0.999	CLDN20
rs112974423	6	155,589,945	C	T	0.985	0.998	CLDN20
rs9397796	6	155,590,031	A	G	0.801	0.999	CLDN20
6:155590120_TA_T	6	155,590,120	TA	T	0.388	0.977	CLDN20
rs796335095	6	155,590,458	C	CTTT	0.786	0.948	CLDN20
rs12214833	6	155,590,518	C	T	0.801	0.999	CLDN20
rs188074953	6	155,590,581	G	A	0.988	0.942	CLDN20
rs2989456	6	155,590,693	C	T	0.383	1	CLDN20
rs9322507	6	155,590,809	A	T	0.448	1	CLDN20
rs9322508	6	155,591,084	C	T	0.448	1	CLDN20
rs117742314	6	155,591,086	A	G	0.977	0.993	CLDN20
rs9371876	6	155,591,404	G	A	0.801	1	CLDN20
rs9397237	6	155,591,551	A	G	0.801	1	CLDN20
rs7776133	6	155,591,635	C	T	0.898	1	CLDN20
6:155592078_TA_T	6	155,592,078	TA	T	0.952	0.959	CLDN20
rs77845485	6	155,592,084	T	G	0.935	0.999	CLDN20
rs111594627	6	155,592,525	A	G	0.985	0.999	CLDN20
rs67421405	6	155,593,001	G	A	0.899	1	CLDN20
rs75979782	6	155,593,399	G	A	0.95	0.999	CLDN20
rs927717	6	155,594,029	A	G	0.383	1	CLDN20
rs62427184	6	155,594,258	A	C	0.748	1	CLDN20
rs9397798	6	155,594,775	A	T	0.383	1	CLDN20
rs11963195	6	155,594,926	C	T	0.898	1	CLDN20
rs9371877	6	155,594,944	T	C	0.383	1	CLDN20
rs117653067	6	155,595,119	T	G	0.976	0.931	CLDN20
rs6557414	6	155,595,482	G	C	0.384	1	CLDN20
rs73008652	6	155,595,552	A	G	0.898	1	CLDN20
rs9397799	6	155,595,698	G	T	0.801	1	CLDN20
rs73008655	6	155,596,073	C	T	0.952	0.997	CLDN20
rs1969863	6	155,596,127	C	T	0.384	1	CLDN20
rs9371878	6	155,596,285	A	G	0.382	0.998	CLDN20
rs9371879	6	155,596,346	T	C	0.383	1	CLDN20
rs1014664	6	155,596,700	T	C	0.95	1	CLDN20
rs6925410	6	155,597,644	A	G	0.384	1	CLDN20
rs117011404	7	27,133,496	C	G	0.989	0.949	HOXA1
rs111259912	7	27,134,475	T	A	0.987	0.916	HOXA1
rs74458426	7	27,134,638	C	T	0.95	0.975	HOXA1
rs45571645	7	27,135,096	G	T	0.98	0.91	HOXA1
rs766665411	7	27,135,313	GCGA	G	0.975	0.685	HOXA1
rs10951154	7	27,135,314	C	T	0.144	0.918	HOXA1
rs55855101	7	73,443,015	G	A	0.596	0.994	ELN
rs7784595	7	73,443,132	G	T	0.938	0.999	ELN
rs3823878	7	73,443,245	G	A	0.938	0.999	ELN
rs1859763	7	73,443,281	T	G	0.047	0.993	ELN
rs56331128	7	73,444,116	C	T	0.987	0.92	ELN
rs868005	7	73,445,114	T	C	0.595	0.994	ELN
rs5884929	7	73,445,306	G	GT	0.938	0.997	ELN
rs56133616	7	73,445,706	T	C	0.987	0.983	ELN
rs884843	7	73,445,789	A	G	0.532	0.994	ELN
rs2356532	7	73,445,942	A	G	0.938	0.998	ELN
rs58411014	7	73,446,397	A	G	0.938	0.998	ELN
rs60883031	7	73,446,436	T	C	0.938	0.998	ELN
rs873647	7	73,447,506	G	A	0.938	0.998	ELN
rs870424	7	73,447,658	T	C	0.938	0.998	ELN
rs55675706	7	73,448,264	C	T	0.986	0.882	ELN
rs55772967	7	73,448,499	A	G	0.959	0.959	ELN
rs200488952	7	73,448,825	T	TGAGC	0.938	0.994	ELN
rs56017804	7	73,448,994	T	C	0.938	0.998	ELN
rs55863875	7	73,449,246	C	A	0.917	0.993	ELN
rs17146001	7	73,449,846	G	A	0.938	0.997	ELN
rs144049843	7	73,450,110	T	TA	0.938	0.996	ELN
rs56289587	7	73,451,680	C	T	0.984	0.947	ELN
rs2301995	7	73,452,140	G	A	0.938	0.997	ELN
rs2301994	7	73,452,263	G	A	0.938	0.997	ELN
rs56027431	7	73,452,862	C	T	0.988	0.862	ELN
rs3801458	7	73,452,911	G	A	0.938	0.997	ELN
rs141346927	7	73,454,065	G	A	0.99	0.871	ELN
rs3801459	7	73,454,409	T	G	0.938	0.997	ELN
rs3801460	7	73,454,474	C	T	0.938	0.997	ELN
rs1859761	7	73,454,629	T	G	0.13	0.997	ELN
rs3823879	7	73,454,791	G	A	0.938	0.997	ELN
rs3815251	7	73,455,276	T	C	0.938	0.997	ELN
rs2286257	7	73,455,384	G	A	0.938	0.997	ELN
rs112375055	7	73,456,368	C	G	0.986	0.919	ELN
rs2856729	7	73,456,648	C	G	0.967	0.943	ELN
rs13239907	7	73,456,850	G	A	0.919	0.999	ELN
rs28763981	7	73,457,255	G	A	0.936	0.981	ELN
rs55868272	7	73,457,506	C	T	0.986	0.878	ELN
rs2528796	7	73,457,590	G	C	0.967	0.945	ELN
rs28763982	7	73,457,701	G	A	0.954	0.912	ELN
7:73458575_CAAAGAA_C	7	73,458,575	CAAAGAA	C	0.987	0.846	ELN
rs555241353	7	73,461,430	G	GA	0.94	0.972	ELN
rs111064941	7	73,463,729	T	C	0.533	0.993	ELN
rs4717127	7	73,466,810	A	G	0.081	0.985	ELN
rs2239691	7	73,469,169	C	T	0.597	0.993	ELN
rs148846464	7	73,469,287	C	T	0.981	0.961	ELN
rs111403727	7	73,470,198	G	C	0.961	0.951	ELN
7:73470421_AT_A	7	73,470,421	AT	A	0.984	0.824	ELN
rs28763986	7	73,472,050	C	T	0.465	0.899	ELN
rs147742727	7	73,472,579	T	TTTC	0.734	0.86	ELN
rs2528795	7	73,473,494	T	C	0.776	0.966	ELN
rs17855988	7	73,474,825	G	C	0.901	0.966	ELN
rs56240724	7	73,474,985	C	T	0.971	0.921	ELN
rs12535378	7	73,475,775	G	A	0.845	0.972	ELN
rs810556	7	73,476,236	T	C	0.897	0.932	ELN
rs810555	7	73,476,249	C	T	0.858	0.936	ELN
rs541803683	7	73,476,873	G	GTTGT	0.97	0.755	ELN
rs34703969	7	73,478,476	T	C	0.97	0.819	ELN
rs113395463	7	73,478,524	A	G	0.888	0.969	ELN
rs62476385	7	73,478,562	A	G	0.856	0.919	ELN
rs34325713	7	73,478,574	T	C	0.963	0.82	ELN
rs201104104	7	73,478,581	T	TA	0.777	0.895	ELN
rs193224617	7	73,478,624	A	T	0.976	0.898	ELN
rs810554	7	73,478,753	A	T	0.607	0.777	ELN
rs188598311	7	73,478,755	T	A	0.843	0.947	ELN
rs192259261	7	73,478,757	T	A	0.879	0.928	ELN
rs370616120	7	73,479,395	A	AC	0.903	0.898	ELN
rs78804634	7	73,479,402	C	A	0.945	0.842	ELN
7:73479946_GA_G	7	73,479,946	GA	G	0.614	0.669	ELN
rs45618836	7	73,480,258	A	G	0.967	0.943	ELN
rs10949834	7	73,480,569	G	A	0.868	0.996	ELN
rs2528794	7	73,480,805	G	A	0.883	0.982	ELN
rs3757587	7	73,481,028	C	T	0.868	0.996	ELN
rs71539239	7	73,481,549	A	G	0.867	0.997	ELN
7:73481604_CT_C	7	73,481,604	CT	C	0.865	0.986	ELN
rs113017693	7	73,481,889	T	A	0.868	0.997	ELN
rs184014645	7	73,481,924	C	T	0.868	0.997	ELN
rs55675961	7	73,482,621	G	A	0.987	0.996	ELN
rs11760714	7	73,482,701	A	G	0.868	0.998	ELN
rs34208922	7	73,483,531	C	CA	0.749	0.983	ELN
rs10233395	7	73,484,224	A	G	0.934	0.996	ELN
rs1135169	9	94,484,928	A	C	0.933	0.993	ROR2
rs1135150	9	94,485,232	G	A	0.933	0.992	ROR2
9:94485768_GCTCT_G	9	94,485,768	GCTCT	G	0.557	0.994	ROR2
rs2230578	9	94,485,928	C	T	0.31	0.998	ROR2
rs2230577	9	94,486,622	G	A	0.933	0.99	ROR2
rs10992063	9	94,486,688	G	A	0.556	0.993	ROR2
rs10992065	9	94,487,459	C	T	0.721	0.884	ROR2

SNP	Chromosome	Position	Effect Allele	Other Allele	Effect Allele Frequency	Imputation quality	Gene
rs200236339	9	94,487,492	A	AG	0.982	0.711	ROR2
rs77618210	9	94,487,765	T	C	0.962	0.968	ROR2
rs4073736	9	94,488,400	T	C	0.551	0.984	ROR2
rs4073735	9	94,488,415	C	T	0.552	0.984	ROR2
rs10992066	9	94,489,212	C	T	0.873	0.994	ROR2
rs76060308	9	94,489,597	G	C	0.989	0.971	ROR2
rs11436642	9	94,489,683	A	AT	0.772	0.943	ROR2
rs533778903	9	94,489,697	T	A	0.948	0.972	ROR2
rs72744454	9	94,489,964	C	T	0.939	0.987	ROR2
rs9409649	9	94,490,131	T	C	0.45	0.99	ROR2
9:94490707_GTGTT_G	9	94,490,707	GTGTT	G	0.91	0.955	ROR2
9:94490713_GTTTA_G	9	94,490,713	GTTTA	G	0.911	0.954	ROR2
rs9409650	9	94,490,838	G	A	0.51	0.992	ROR2
rs113284213	9	94,490,993	G	A	0.956	0.978	ROR2
rs78995057	9	94,491,163	C	T	0.98	0.982	ROR2
rs11415635	9	94,491,371	TA	T	0.442	0.947	ROR2
rs79041539	9	94,492,503	G	A	0.979	0.976	ROR2
rs10992068	9	94,492,678	T	C	0.873	0.996	ROR2
rs10992069	9	94,492,679	T	A	0.873	0.996	ROR2
rs10992070	9	94,493,470	G	A	0.873	0.997	ROR2
rs117340689	9	94,493,972	G	A	0.905	0.998	ROR2
rs10992071	9	94,494,471	C	T	0.855	0.993	ROR2
rs9409456	9	94,494,561	C	A	0.508	0.996	ROR2
rs117316382	9	94,494,758	T	C	0.928	0.991	ROR2
rs10992072	9	94,495,005	C	T	0.81	0.995	ROR2
rs79697373	9	94,495,285	G	A	0.939	0.988	ROR2
rs9409457	9	94,495,286	G	A	0.509	0.996	ROR2
rs77626429	9	94,496,038	T	C	0.905	0.999	ROR2
rs117501376	9	94,496,112	C	A	0.985	0.995	ROR2
rs72744455	9	94,496,357	C	T	0.871	0.998	ROR2
rs7869182	9	94,497,042	C	T	0.449	0.998	ROR2
rs4237215	9	94,497,680	A	C	0.353	0.998	ROR2
rs148517898	9	94,498,030	C	A	0.984	0.918	ROR2
rs9409458	9	94,498,370	A	G	0.509	0.995	ROR2
rs9409651	9	94,498,550	C	T	0.509	0.995	ROR2
rs7855522	9	94,499,618	G	A	0.509	0.995	ROR2
rs16907720	9	94,499,797	A	G	0.939	0.989	ROR2
rs9409459	9	94,499,964	T	C	0.021	0.996	ROR2
rs79766334	9	94,499,965	G	A	0.904	0.997	ROR2
rs9409652	9	94,500,776	A	G	0.451	0.992	ROR2
rs143584454	9	94,501,795	T	A	0.909	0.987	ROR2
rs10992075	9	94,501,891	A	G	0.726	0.992	ROR2
rs147248574	9	94,502,616	G	A	0.982	0.914	ROR2
rs10992076	9	94,502,812	T	C	0.911	0.994	ROR2
rs4604505	9	94,502,838	G	C	0.42	0.99	ROR2
rs149094761	9	94,502,842	G	C	0.969	0.96	ROR2
rs142209460	9	94,502,867	T	C	0.964	0.859	ROR2
rs4500141	9	94,502,883	T	A	0.376	0.985	ROR2
rs4500142	9	94,503,309	T	C	0.249	0.99	ROR2
rs4493962	9	94,503,390	T	C	0.442	0.988	ROR2
rs112583695	9	94,503,589	A	G	0.979	0.588	ROR2
rs7875973	9	94,504,400	G	A	0.894	0.992	ROR2
rs4744095	9	94,504,657	T	C	0.295	0.99	ROR2
rs59418928	9	94,504,780	C	T	0.912	0.993	ROR2
rs72744464	9	94,505,093	C	A	0.938	0.994	ROR2
rs72744465	9	94,505,232	G	A	0.938	0.994	ROR2
rs72744467	9	94,505,575	C	T	0.938	0.995	ROR2
rs9409653	9	94,506,153	A	G	0.25	0.994	ROR2
rs9409654	9	94,506,614	T	C	0.25	0.994	ROR2
rs79209566	9	94,506,705	A	G	0.983	0.985	ROR2
rs10992077	9	94,506,712	G	T	0.911	0.993	ROR2
rs7031729	9	94,506,930	C	A	0.443	0.994	ROR2
rs3935846	9	94,506,959	A	T	0.25	0.995	ROR2
rs17585790	9	94,506,983	A	C	0.983	0.987	ROR2
rs4401931	9	94,507,454	G	C	0.25	0.995	ROR2
rs118143261	9	94,507,567	C	T	0.966	0.977	ROR2
9:94507708_ATTTTTTT_A	9	94,507,708	ATTTTTTT	A	0.714	0.922	ROR2
rs7860353	9	94,507,793	C	T	0.912	0.993	ROR2
rs3935053	9	94,508,114	A	G	0.25	0.996	ROR2
rs10992078	9	94,508,126	G	A	0.912	0.993	ROR2
rs74306057	9	94,508,337	T	G	0.983	0.985	ROR2
9:94508344_TTTTG_T	9	94,508,344	TTTTG	T	0.981	0.878	ROR2
rs3935603	9	94,508,505	C	T	0.506	0.994	ROR2
rs3935602	9	94,508,663	C	T	0.251	0.996	ROR2
rs3935601	9	94,508,700	T	C	0.444	0.994	ROR2
rs79830490	9	94,508,903	G	A	0.983	0.987	ROR2
rs10125728	9	94,509,206	G	T	0.982	0.982	ROR2
rs72744472	9	94,509,629	A	G	0.937	0.998	ROR2
rs72744473	9	94,509,743	T	A	0.937	0.998	ROR2
rs5899141	9	94,509,986	C	CTTTG	0.249	0.993	ROR2
rs73651503	9	94,510,040	G	A	0.895	0.992	ROR2
rs16907725	9	94,510,145	G	A	0.895	0.992	ROR2
rs60810449	9	94,510,588	C	CT	0.817	0.992	ROR2
rs9409655	9	94,510,635	A	G	0.25	0.997	ROR2
rs9409656	9	94,510,673	C	T	0.25	0.997	ROR2
rs62563404	9	94,510,700	A	G	0.983	0.957	ROR2
rs72744474	9	94,511,584	C	T	0.948	0.995	ROR2
rs10992081	9	94,511,959	C	T	0.834	0.996	ROR2
rs75060403	9	94,512,889	T	C	0.983	0.988	ROR2
rs755984386	9	94,513,093	GAATCTGAA	G	0.974	0.862	ROR2
rs151086484	9	94,513,152	C	T	0.985	0.97	ROR2
rs112474427	9	94,513,321	T	C	0.989	0.951	ROR2
rs16907728	9	94,513,448	A	G	0.983	0.986	ROR2
rs10739919	9	94,514,322	A	G	0.251	1	ROR2
rs72744477	9	94,514,925	G	C	0.948	0.995	ROR2
rs148448968	9	94,515,257	C	T	0.99	0.981	ROR2
rs4372069	9	94,515,416	C	T	0.247	0.998	ROR2
rs4585802	9	94,515,565	G	A	0.247	0.998	ROR2
rs77958412	9	94,516,231	T	A	0.983	0.987	ROR2
rs770779673	9	94,516,335	TGAC	T	0.457	0.979	ROR2
rs4595185	9	94,516,377	G	A	0.385	0.997	ROR2
rs7047937	9	94,516,768	G	A	0.402	0.995	ROR2
rs7849691	9	94,517,034	C	T	0.948	0.991	ROR2
rs11365403	9	94,517,445	GA	G	0.863	0.994	ROR2
rs80124250	9	94,517,556	C	T	0.952	0.977	ROR2
rs4467996	9	94,517,715	C	A	0.41	0.991	ROR2
rs12683181	9	94,518,328	C	T	0.262	0.988	ROR2
rs3824426	9	94,518,789	C	T	0.985	0.986	ROR2
rs3824427	9	94,518,796	A	G	0.985	0.984	ROR2
rs4631528	9	94,518,800	A	T	0.836	0.986	ROR2
rs76100717	9	94,519,002	T	G	0.985	0.984	ROR2
rs374979318	9	94,519,025	CTTTTTTT	C	0.787	0.95	ROR2
rs142049312	9	94,519,178	G	A	0.972	0.986	ROR2
rs71494474	9	94,519,348	C	G	0.888	0.985	ROR2
rs35022100	9	94,519,361	C	T	0.888	0.986	ROR2
rs60617711	9	94,519,956	C	T	0.978	0.992	ROR2
rs7872615	9	94,520,921	G	A	0.888	0.998	ROR2
rs7872865	9	94,521,117	G	A	0.888	0.998	ROR2
rs80189775	9	94,521,457	G	T	0.972	0.996	ROR2
9:94521508_GGC_G	9	94,521,508	GGC	G	0.978	0.992	ROR2
rs34134725	9	94,521,525	G	A	0.888	0.998	ROR2
rs71494475	9	94,522,403	T	C	0.888	0.998	ROR2
rs58462029	9	94,522,643	C	T	0.978	0.993	ROR2
rs62563406	9	94,522,686	C	T	0.984	0.946	ROR2
rs60092492	9	94,522,692	G	T	0.978	0.996	ROR2
rs7852408	9	94,522,902	C	T	0.888	0.998	ROR2
rs80147245	9	94,523,804	T	G	0.972	0.997	ROR2
rs57810120	9	94,523,848	G	A	0.978	0.996	ROR2
rs149221962	9	94,524,307	T	G	0.968	0.937	ROR2
9:94524673_CAAT_C	9	94,524,673	CAAT	C	0.888	0.996	ROR2
rs73651504	9	94,525,021	C	T	0.888	0.999	ROR2
rs71494476	9	94,525,131	T	A	0.888	0.999	ROR2
rs60099576	9	94,525,161	C	T	0.837	0.998	ROR2
rs7860482	9	94,525,452	G	A	0.888	0.999	ROR2
rs4295719	9	94,525,596	C	T	0.972	0.996	ROR2
rs140606517	9	94,525,606	A	G	0.959	0.929	ROR2
rs17585941	9	94,525,991	A	C	0.86	0.999	ROR2
rs71494477	9	94,526,194	G	A	0.888	0.999	ROR2
rs35810339	9	94,526,325	G	A	0.888	0.999	ROR2
rs35238595	9	94,526,649	T	C	0.888	0.999	ROR2
rs201566562	9	94,526,840	T	A	0.968	0.947	ROR2
rs4595186	9	94,527,618	T	C	0.949	0.998	ROR2
rs4452868	9	94,527,657	G	A	0.978	0.997	ROR2
rs79560736	9	94,528,012	C	T	0.972	0.997	ROR2
rs73651508	9	94,528,029	A	C	0.978	0.997	ROR2
rs34854279	9	94,528,382	C	G	0.837	0.999	ROR2
rs10992085	9	94,528,401	G	A	0.846	0.993	ROR2
rs77270173	9	94,528,527	C	A	0.972	0.997	ROR2
rs10512217	9	94,528,569	T	C	0.949	0.999	ROR2
rs58210546	9	94,528,744	G	A	0.978	0.997	ROR2
rs16907741	9	94,529,318	C	A	0.949	0.998	ROR2
rs7855073	9	94,529,362	G	A	0.847	0.977	ROR2
rs7855079	9	94,529,385	G	T	0.839	0.992	ROR2
rs12235606	9	94,529,388	C	T	0.951	0.982	ROR2
rs12235611	9	94,529,473	C	T	0.949	0.999	ROR2

SNP	Chromosome	Position	Effect Allele	Other Allele	Effect Allele Frequency	Imputation quality	Gene
rs12237459	9	94,529,704	G	A	0.949	0.999	ROR2
rs73511086	9	94,530,050	C	T	0.978	0.997	ROR2
rs12685849	9	94,530,820	G	A	0.978	0.997	ROR2
rs9409461	9	94,530,989	G	A	0.522	0.977	ROR2
rs7035950	9	94,531,330	G	A	0.888	0.998	ROR2
rs35557004	9	94,531,833	G	A	0.888	0.998	ROR2
rs145993592	9	94,531,986	C	A	0.986	0.886	ROR2
rs34447092	9	94,532,260	C	A	0.888	0.998	ROR2
rs12685213	9	94,532,306	A	G	0.978	0.997	ROR2
rs7026175	9	94,532,382	A	C	0.837	0.998	ROR2
rs16907754	9	94,532,487	C	G	0.949	0.999	ROR2
rs3935544	9	94,532,795	G	A	0.977	0.997	ROR2
rs10992087	9	94,533,121	G	A	0.948	0.995	ROR2
rs7872353	9	94,533,245	C	G	0.888	0.998	ROR2
rs10992088	9	94,533,489	T	C	0.949	0.998	ROR2
rs10992089	9	94,533,566	T	C	0.949	0.998	ROR2
rs571307972	9	94,533,995	C	T	0.969	0.949	ROR2
rs745750113	9	94,534,004	T	TTC	0.852	0.937	ROR2
rs768639837	9	94,534,005	T	TCT	0.847	0.909	ROR2
rs76475985	9	94,534,065	G	A	0.989	0.965	ROR2
rs73511102	9	94,534,355	G	T	0.949	0.998	ROR2
rs73513203	9	94,534,656	T	C	0.977	0.997	ROR2
rs17586150	9	94,534,777	G	C	0.888	0.997	ROR2
rs16907761	9	94,534,812	T	A	0.949	0.998	ROR2
rs16907764	9	94,534,917	T	G	0.949	0.998	ROR2
rs17586213	9	94,535,010	T	C	0.972	0.996	ROR2
rs12238428	9	94,535,412	T	C	0.949	0.997	ROR2
rs7039620	9	94,535,632	A	G	0.837	0.996	ROR2
9:94535636_CGTGCTTCACA_C	9	94,535,636	CGTGCTTCACA	C	0.976	0.894	ROR2
rs75153534	9	94,536,191	C	T	0.972	0.992	ROR2
rs10992090	9	94,536,329	G	A	0.977	0.996	ROR2
rs35725000	9	94,536,815	G	T	0.888	0.997	ROR2
rs7863557	9	94,538,115	C	G	0.745	0.999	ROR2
rs3802377	9	94,538,167	T	C	0.977	0.995	ROR2
rs3802378	9	94,538,200	C	T	0.977	0.995	ROR2
rs3802379	9	94,538,304	C	T	0.972	0.994	ROR2
rs10992091	9	94,538,388	G	A	0.745	0.999	ROR2
rs10992092	9	94,538,483	G	A	0.745	0.999	ROR2
rs10992093	9	94,538,500	C	T	0.745	0.999	ROR2
rs4467997	9	94,539,018	C	A	0.694	0.998	ROR2
rs71494478	9	94,539,035	C	T	0.888	0.997	ROR2
rs10659675	9	94,539,118	C	CAT	0.745	0.998	ROR2
rs16907776	9	94,539,436	A	G	0.977	0.995	ROR2
rs150076293	9	94,539,492	A	G	0.98	0.96	ROR2
rs112876626	9	94,539,671	C	CCA	0.784	0.936	ROR2
rs7021529	9	94,539,902	C	T	0.683	0.997	ROR2
rs78790186	9	94,540,005	C	T	0.972	0.993	ROR2
rs34559953	9	94,540,095	A	G	0.888	0.997	ROR2
rs12336151	9	94,540,344	G	T	0.964	0.912	ROR2
rs7041425	9	94,540,436	T	A	0.745	1	ROR2
rs10992094	9	94,541,047	T	C	0.857	0.997	ROR2
rs79414554	9	94,541,382	T	A	0.972	0.993	ROR2
rs77960295	9	94,541,495	G	A	0.972	0.993	ROR2
rs10992095	9	94,541,512	T	C	0.977	0.996	ROR2
rs10992096	9	94,541,734	C	A	0.857	0.998	ROR2
rs12237573	9	94,541,813	C	T	0.977	0.994	ROR2
rs10992097	9	94,542,019	A	C	0.745	1	ROR2
rs7029673	9	94,542,162	G	A	0.745	1	ROR2
rs7029809	9	94,542,262	G	A	0.745	1	ROR2
rs7046894	9	94,542,528	A	G	0.745	1	ROR2
rs7046896	9	94,542,531	A	C	0.745	1	ROR2
9:94542667_AT_A	9	94,542,667	AT	A	0.987	0.932	ROR2
rs10992099	9	94,542,889	G	A	0.857	0.997	ROR2
rs142243104	9	94,542,920	A	G	0.965	0.907	ROR2
rs4744097	9	94,543,365	G	A	0.694	0.999	ROR2
rs7862819	9	94,543,518	C	T	0.745	1	ROR2
rs17514461	9	94,543,673	C	T	0.972	0.992	ROR2
rs4744098	9	94,544,282	G	C	0.69	0.997	ROR2
rs79902509	9	94,544,622	G	A	0.972	0.991	ROR2
rs74483489	9	94,544,782	T	G	0.972	0.991	ROR2
rs550009635	9	94,544,866	CA	C	0.354	0.916	ROR2
rs139561969	9	94,544,909	A	AGTGTAC	0.742	0.99	ROR2
rs72744489	9	94,545,320	T	C	0.74	0.995	ROR2
rs10992100	9	94,545,363	A	G	0.69	0.995	ROR2
rs34466526	9	94,545,542	A	G	0.74	0.994	ROR2
rs148470779	9	94,545,879	T	TCAGCTG	0.765	0.948	ROR2
rs9409657	9	94,546,314	T	C	0.05	0.981	ROR2
rs74321654	9	94,546,432	G	A	0.983	0.982	ROR2
rs3935382	9	94,546,564	C	T	0.845	0.988	ROR2
rs9409462	9	94,546,619	T	C	0.191	0.991	ROR2
rs4595189	9	94,546,983	A	T	0.361	0.999	ROR2
rs10992104	9	94,547,368	C	T	0.956	0.993	ROR2
rs4497030	9	94,547,455	T	C	0.317	0.998	ROR2
rs4601388	9	94,547,923	A	G	0.978	0.999	ROR2
rs78052819	9	94,547,997	G	A	0.978	0.987	ROR2
9:94548144_ATC_A	9	94,548,144	ATC	A	0.888	0.988	ROR2
rs35972600	9	94,548,440	C	CA	0.987	0.742	ROR2
rs9409658	9	94,548,490	A	T	0.321	0.998	ROR2
rs113758412	9	94,548,505	A	AGACTGAGG	0.978	0.999	ROR2
9:94548604_GTCAA_G	9	94,548,604	GTCAA	G	0.974	0.936	ROR2
rs202079139	9	94,548,636	GTAAA	G	0.556	0.82	ROR2
rs139645972	9	94,548,642	A	C	0.918	0.769	ROR2
rs78823154	9	94,548,648	A	G	0.905	0.807	ROR2
rs10992105	9	94,548,906	C	T	0.858	0.992	ROR2
rs10992106	9	94,548,908	T	C	0.956	0.993	ROR2
rs10992107	9	94,548,909	G	A	0.956	0.993	ROR2
rs10820902	9	94,549,141	T	A	0.978	1	ROR2
rs10820903	9	94,549,157	A	C	0.956	0.993	ROR2
rs79653884	9	94,549,447	A	G	0.978	0.986	ROR2
rs4400454	9	94,549,545	C	T	0.978	0.998	ROR2
rs112959888	9	94,549,643	A	G	0.972	0.909	ROR2
rs10992108	9	94,550,084	G	T	0.982	0.911	ROR2
9:94550090_GA_G	9	94,550,090	GA	G	0.963	0.923	ROR2
rs59149734	9	94,550,091	A	G	0.986	0.79	ROR2
rs114661477	9	94,550,093	A	G	0.986	0.786	ROR2
rs10761130	9	94,550,186	A	C	0.365	0.993	ROR2
rs4744100	9	94,550,214	G	A	0.067	0.98	ROR2
rs74934138	9	94,550,439	G	C	0.978	0.98	ROR2
rs78338960	9	94,550,700	G	A	0.865	0.983	ROR2
rs183241955	9	94,550,725	G	A	0.97	0.914	ROR2
rs186525158	9	94,550,726	C	A	0.97	0.915	ROR2
rs73513246	9	94,550,746	A	G	0.978	0.987	ROR2
rs112992938	9	94,550,824	A	G	0.978	0.827	ROR2
rs112531673	9	94,550,905	G	A	0.978	0.986	ROR2
rs12553067	9	94,551,016	G	A	0.751	0.984	ROR2
9:94551142_CA_C	9	94,551,142	CA	C	0.642	0.872	ROR2
rs10992109	9	94,551,392	T	C	0.956	0.982	ROR2
rs1406201	9	94,551,682	G	C	0.865	0.979	ROR2
rs2178196	9	94,552,501	A	G	0.956	0.981	ROR2
rs2312730	9	94,552,749	G	A	0.978	0.982	ROR2
rs4322069	9	94,552,755	G	A	0.978	0.983	ROR2
rs773009039	9	94,552,794	TA	T	0.84	0.922	ROR2
rs2312731	9	94,553,076	A	G	0.855	0.978	ROR2
rs4237216	9	94,553,169	G	A	0.865	0.977	ROR2
rs35705862	9	94,554,039	A	AC	0.955	0.969	ROR2
rs10992110	9	94,554,249	C	T	0.957	0.98	ROR2
rs10992111	9	94,554,614	G	C	0.978	0.975	ROR2
rs542872742	9	94,554,648	A	G	0.977	0.874	ROR2
rs556430423	9	94,554,650	T	C	0.977	0.874	ROR2
rs7856496	9	94,554,651	G	A	0.849	0.936	ROR2
rs574924299	9	94,554,673	TC	T	0.509	0.924	ROR2
rs62563410	9	94,554,802	A	G	0.98	0.878	ROR2
rs56020365	9	94,554,834	C	T	0.93	0.93	ROR2
rs142852384	9	94,554,841	T	C	0.93	0.929	ROR2
rs60838127	9	94,554,870	A	G	0.88	0.945	ROR2
rs191784274	9	94,555,528	G	A	0.986	0.936	ROR2
rs16907784	9	94,555,614	T	C	0.989	0.99	ROR2
rs10992112	9	94,555,811	G	A	0.976	0.995	ROR2
9:94556781_GA_G	9	94,556,781	GA	G	0.965	0.993	ROR2
rs7855006	9	94,557,132	A	G	0.964	0.998	ROR2
rs10992114	9	94,557,413	A	G	0.965	0.998	ROR2
rs7855609	9	94,557,641	A	G	0.964	0.998	ROR2
rs4744101	9	94,558,058	A	G	0.964	0.998	ROR2
rs4743852	9	94,558,126	T	C	0.964	0.998	ROR2
rs10636189	9	94,558,260	C	CCT	0.964	0.994	ROR2
rs180974584	9	94,558,663	C	T	0.986	0.942	ROR2
rs144437686	9	94,559,053	A	C	0.964	0.993	ROR2
rs199581047	9	94,559,429	G	A	0.966	0.987	ROR2
rs35997310	9	94,560,755	A	G	0.075	0.869	ROR2
rs2103301	9	94,561,730	T	C	0.963	0.999	ROR2
rs150384436	9	94,562,443	G	A	0.989	0.997	ROR2
rs111878724	9	94,562,619	C	T	0.975	0.995	ROR2
rs7029814	9	94,562,681	T	C	0.699	0.965	ROR2
rs7872107	9	94,563,174	C	A	0.964	0.999	ROR2
rs7872492	9	94,563,477	C	T	0.964	0.999	ROR2

SNP	Chromosome	Position	Effect Allele	Other Allele	Effect Allele Frequency	Imputation quality	Gene
rs9299398	9	94,563,880	A	G	0.963	0.999	ROR2
rs73513266	9	94,564,208	T	G	0.964	0.998	ROR2
rs59394284	9	94,564,504	C	T	0.964	0.998	ROR2
9:94564523_CT_C	9	94,564,523	CT	C	0.964	0.996	ROR2
rs16907793	9	94,564,762	C	T	0.976	0.997	ROR2
rs77075046	9	94,565,115	G	C	0.989	0.996	ROR2
rs4743854	9	94,565,289	C	T	0.964	0.998	ROR2
rs34526003	9	94,566,461	G	T	0.96	0.958	ROR2
rs10992115	9	94,566,476	T	C	0.974	0.975	ROR2
rs4744103	9	94,566,643	C	T	0.964	0.998	ROR2
rs3802380	9	94,566,892	A	G	0.963	0.998	ROR2
rs138689182	9	94,567,192	C	G	0.988	0.915	ROR2
rs16907798	9	94,567,537	G	A	0.964	0.998	ROR2
rs117942540	9	94,567,806	C	A	0.989	0.994	ROR2
rs75738223	9	94,567,820	A	G	0.976	0.997	ROR2
rs12336034	9	94,567,835	A	G	0.963	0.997	ROR2
rs12683746	9	94,567,977	G	A	0.964	0.997	ROR2
rs10820904	9	94,568,424	C	A	0.976	0.996	ROR2
rs35357880	9	94,568,448	A	G	0.964	0.996	ROR2
rs140997188	9	94,569,073	G	A	0.973	0.967	ROR2
rs113782402	9	94,569,078	A	G	0.959	0.84	ROR2
rs2494788	9	94,569,127	A	T	0.973	0.961	ROR2
rs146223362	9	94,569,153	A	G	0.972	0.923	ROR2
rs7048143	9	94,569,160	C	T	0.777	0.954	ROR2
rs10761131	9	94,569,251	A	G	0.963	0.993	ROR2
rs80130165	9	94,569,558	G	A	0.988	0.99	ROR2
rs7861514	9	94,569,756	A	G	0.963	0.999	ROR2
rs7849482	9	94,569,803	C	T	0.963	0.998	ROR2
rs76386801	9	94,569,837	T	C	0.989	0.993	ROR2
9:94570391_GA_G	9	94,570,391	GA	G	0.956	0.965	ROR2
rs10992116	9	94,570,436	T	C	0.959	0.999	ROR2
rs10992117	9	94,570,583	A	G	0.959	0.999	ROR2
rs80355056	9	94,571,016	G	A	0.984	0.992	ROR2
rs28580750	9	94,571,084	C	T	0.974	0.997	ROR2
9:94571086_TA_T	9	94,571,086	TA	T	0.977	0.919	ROR2
rs57252496	9	94,571,201	C	A	0.975	1	ROR2
rs58953970	9	94,571,233	T	C	0.96	0.996	ROR2
9:94571284_CA_C	9	94,571,284	CA	C	0.911	0.834	ROR2
rs59903414	9	94,571,332	G	T	0.984	0.996	ROR2
rs10992118	9	94,571,449	A	T	0.958	0.998	ROR2
rs117869062	9	94,571,715	A	G	0.978	0.925	ROR2
rs7858240	9	94,572,240	C	T	0.959	0.998	ROR2
rs4744104	9	94,572,290	C	T	0.959	0.998	ROR2
rs10992119	9	94,572,312	G	A	0.975	1	ROR2
rs2841693	9	94,572,339	C	T	0.966	0.991	ROR2
rs77550788	9	94,572,671	G	A	0.984	0.994	ROR2
rs10992121	9	94,573,753	A	G	0.96	0.992	ROR2
rs73513287	9	94,573,919	T	C	0.961	0.993	ROR2
rs76427104	9	94,574,074	C	T	0.948	0.962	ROR2
rs527726654	9	94,574,343	C	CA	0.77	0.782	ROR2
rs73513289	9	94,574,413	T	C	0.96	0.991	ROR2
rs76306939	9	94,574,676	G	A	0.976	0.995	ROR2
rs76397354	9	94,574,872	C	G	0.961	0.992	ROR2
rs10992122	9	94,575,175	C	G	0.946	0.961	ROR2
rs73513293	9	94,575,400	A	C	0.976	0.998	ROR2
rs73513294	9	94,575,704	T	C	0.976	0.996	ROR2
rs80173010	9	94,576,210	G	A	0.985	0.984	ROR2
rs59845812	9	94,576,514	G	A	0.985	0.983	ROR2
rs17514885	9	94,576,636	G	A	0.831	0.939	ROR2
rs7853430	9	94,577,950	C	T	0.985	0.982	ROR2
rs528608875	9	94,578,854	G	A	0.989	0.988	ROR2
rs140292476	9	94,579,964	C	T	0.983	0.966	ROR2
rs116912684	9	94,580,561	T	G	0.986	0.946	ROR2
rs80331497	9	94,581,295	A	G	0.986	0.977	ROR2
rs372377330	9	94,581,935	T	TGTGTCTGTGTCTTTCA	0.249	0.721	ROR2
rs62563412	9	94,582,457	T	C	0.694	0.989	ROR2
rs117331532	9	94,582,505	C	T	0.989	0.831	ROR2
rs7032536	9	94,582,838	G	A	0.953	0.989	ROR2
rs10992123	9	94,582,873	G	A	0.762	0.987	ROR2
rs2312732	9	94,582,969	C	T	0.716	0.988	ROR2
rs10820905	9	94,584,121	A	C	0.67	0.989	ROR2
rs55663665	9	94,584,273	T	C	0.754	0.976	ROR2
rs769884817	9	94,584,275	TA	T	0.754	0.976	ROR2
rs1569141	9	94,584,365	C	T	0.953	0.99	ROR2
rs10992124	9	94,584,452	C	A	0.751	0.988	ROR2
rs79813193	9	94,584,837	C	T	0.971	0.986	ROR2
rs1997286	9	94,585,666	G	A	0.725	0.987	ROR2
rs2312733	9	94,586,200	A	G	0.677	0.988	ROR2
rs2312734	9	94,586,256	A	G	0.725	0.987	ROR2
rs1534535	9	94,586,556	C	T	0.784	0.986	ROR2
rs10820906	9	94,587,274	G	C	0.73	0.988	ROR2
rs11788348	9	94,588,331	C	T	0.953	0.99	ROR2
rs11790808	9	94,588,498	T	C	0.953	0.99	ROR2
rs73651562	9	94,588,625	C	T	0.966	0.987	ROR2
rs12349890	9	94,588,922	C	T	0.974	0.956	ROR2
9:94589070_AAAAAAAG_A	9	94,589,070	AAAAAAAG	A	0.952	0.985	ROR2
9:94589076_AG_A	9	94,589,076	AG	A	0.795	0.855	ROR2
rs59004196	9	94,589,109	T	G	0.966	0.987	ROR2
rs12551140	9	94,589,135	A	G	0.725	0.988	ROR2
rs16907830	9	94,589,163	A	C	0.966	0.987	ROR2
rs12552292	9	94,589,441	G	A	0.764	0.988	ROR2
rs140055303	9	94,589,872	A	C	0.98	0.986	ROR2
rs12376539	9	94,590,770	T	C	0.722	0.99	ROR2
rs7045333	9	94,591,789	G	A	0.722	0.99	ROR2
rs34687056	9	94,591,831	C	CAAAAAAAAAAA	0.722	0.985	ROR2
rs148920910	9	94,591,958	G	A	0.989	0.871	ROR2
rs11791450	9	94,592,511	G	C	0.67	0.99	ROR2
rs58247635	9	94,592,975	A	AG	0.671	0.985	ROR2
rs7022887	9	94,593,453	G	A	0.966	0.99	ROR2
rs531448361	9	94,593,552	C	CA	0.889	0.917	ROR2
rs4322068	9	94,593,679	C	A	0.73	0.99	ROR2
rs76705291	9	94,593,739	G	T	0.953	0.992	ROR2
rs12376231	9	94,593,925	C	T	0.73	0.99	ROR2
9:94593934_GTAATCTCAGCTACTTGGGAGGCTGAGGCAGGAGAATCCTA_G	9	94,593,934	GTAATCTCAGCTACTTGGGAGGCTGAGGCAGGAGAATCCTA	G	0.952	0.983	ROR2
rs143571569	9	94,593,970	T	A	0.983	0.93	ROR2
rs530547770	9	94,593,974	A	G	0.958	0.945	ROR2
rs7874148	9	94,594,375	T	C	0.73	0.99	ROR2
rs7856144	9	94,594,505	C	T	0.73	0.99	ROR2
rs7855417	9	94,594,626	G	C	0.764	0.99	ROR2
rs6479374	9	94,594,731	T	C	0.73	0.99	ROR2
rs4639579	9	94,595,355	A	C	0.764	0.99	ROR2
rs72746206	9	94,595,494	A	G	0.73	0.99	ROR2
rs73651565	9	94,595,561	T	C	0.966	0.991	ROR2
rs4743857	9	94,595,578	C	G	0.73	0.99	ROR2
rs73651566	9	94,595,968	A	T	0.966	0.99	ROR2
rs78254216	9	94,596,064	A	T	0.953	0.992	ROR2
rs77942610	9	94,596,065	C	A	0.953	0.992	ROR2
rs10992126	9	94,596,450	G	A	0.678	0.991	ROR2
rs1881385	9	94,596,781	T	C	0.678	0.991	ROR2
rs11794240	9	94,597,105	G	A	0.953	0.994	ROR2
rs7867707	9	94,597,646	G	A	0.764	0.991	ROR2
rs7870902	9	94,597,812	G	A	0.759	0.99	ROR2
rs74786201	9	94,598,686	G	A	0.953	0.995	ROR2
rs11792371	9	94,598,999	G	A	0.953	0.995	ROR2
rs34358957	9	94,599,009	A	ATCC	0.954	0.983	ROR2
rs11999739	9	94,599,193	C	G	0.966	0.993	ROR2
rs11788545	9	94,599,263	C	T	0.952	0.995	ROR2
rs10992127	9	94,599,432	T	G	0.989	0.759	ROR2
rs73651567	9	94,599,857	C	T	0.919	0.995	ROR2
rs10992128	9	94,599,967	T	C	0.764	0.992	ROR2
rs7847473	9	94,600,102	T	C	0.677	0.992	ROR2
rs17587223	9	94,600,207	G	A	0.78	0.964	ROR2
rs7847748	9	94,600,343	T	C	0.919	0.996	ROR2
rs62564594	9	94,600,419	C	T	0.978	0.96	ROR2
rs7874163	9	94,600,528	A	G	0.919	0.996	ROR2
rs75745017	9	94,600,630	C	T	0.787	0.964	ROR2
rs16907863	9	94,600,794	C	A	0.953	0.997	ROR2
rs12002827	9	94,600,910	A	G	0.919	0.997	ROR2
rs10992129	9	94,600,956	G	C	0.764	0.993	ROR2
9:94600994_GA_G	9	94,600,994	GA	G	0.764	0.989	ROR2
rs12002851	9	94,601,093	A	G	0.914	0.996	ROR2
rs12003273	9	94,601,233	T	C	0.966	0.997	ROR2
rs7863061	9	94,601,472	C	T	0.764	0.993	ROR2
rs200307863	9	94,601,531	GGGGTGTGTGTGT	G	0.569	0.956	ROR2
rs67304294	9	94,601,533	G	T	0.979	0.612	ROR2
rs75925420	9	94,601,609	T	C	0.953	0.998	ROR2
rs76456976	9	94,601,749	A	T	0.919	0.998	ROR2
rs73651569	9	94,602,080	G	A	0.919	0.998	ROR2
rs73651570	9	94,602,356	G	A	0.919	0.998	ROR2
rs17587376	9	94,602,903	G	A	0.972	0.935	ROR2
rs7032520	9	94,603,217	T	C	0.919	0.998	ROR2
rs12004009	9	94,603,708	A	T	0.919	0.998	ROR2
rs12002478	9	94,603,970	C	T	0.919	0.998	ROR2
rs75086638	9	94,604,290	C	T	0.953	0.998	ROR2
rs76950770	9	94,604,403	A	G	0.953	0.998	ROR2

SNP	Chromosome	Position	Effect Allele	Other Allele	Effect Allele Frequency	Imputation quality	Gene
rs73651572	9	94,604,474	C	T	0.919	0.997	ROR2
rs73651573	9	94,604,475	T	C	0.919	0.997	ROR2
rs78098713	9	94,604,766	G	A	0.953	0.998	ROR2
rs2841691	9	94,605,469	G	A	0.758	0.99	ROR2
rs7849644	9	94,605,545	G	A	0.919	0.999	ROR2
rs73651574	9	94,605,641	G	T	0.966	0.997	ROR2
rs2312735	9	94,605,908	A	G	0.677	0.994	ROR2
rs1534530	9	94,606,212	T	C	0.67	0.996	ROR2
rs16907877	9	94,606,663	C	T	0.966	0.998	ROR2
rs76350755	9	94,606,734	G	T	0.952	0.998	ROR2
rs78872325	9	94,606,735	G	T	0.952	0.998	ROR2
rs79188468	9	94,606,870	T	C	0.952	0.998	ROR2
rs1534531	9	94,607,154	G	A	0.763	0.995	ROR2
rs16907887	9	94,607,294	G	A	0.763	0.995	ROR2
rs7029950	9	94,607,333	G	A	0.73	0.994	ROR2
rs7019210	9	94,607,341	T	C	0.729	0.994	ROR2
rs73651579	9	94,607,743	T	C	0.966	0.998	ROR2
rs2141369	9	94,608,123	G	A	0.952	0.998	ROR2
rs5899143	9	94,608,159	C	CAAA	0.371	0.959	ROR2
rs35783050	9	94,608,398	C	T	0.952	0.998	ROR2
rs36034285	9	94,608,406	C	T	0.952	0.998	ROR2
rs117670467	9	94,608,501	C	G	0.988	0.955	ROR2
rs62564595	9	94,608,540	A	T	0.729	0.995	ROR2
rs6479375	9	94,608,587	A	C	0.729	0.995	ROR2
rs112183196	9	94,608,804	G	T	0.974	0.959	ROR2
rs73651580	9	94,608,814	G	A	0.966	0.997	ROR2
rs7859331	9	94,608,843	A	C	0.729	0.995	ROR2
rs6479376	9	94,609,030	G	A	0.763	0.996	ROR2
rs11793706	9	94,609,365	T	C	0.952	0.998	ROR2
rs2141368	9	94,609,451	A	C	0.783	0.995	ROR2
rs16907893	9	94,609,528	C	T	0.966	0.998	ROR2
rs73651581	9	94,609,884	G	A	0.966	0.998	ROR2
rs73651582	9	94,610,697	G	A	0.966	0.998	ROR2
rs62564596	9	94,610,981	G	A	0.762	0.997	ROR2
rs62564597	9	94,610,982	A	C	0.763	0.991	ROR2
rs1919105	9	94,611,199	A	G	0.925	0.951	ROR2
rs62564598	9	94,611,329	T	G	0.728	0.997	ROR2
rs10992130	9	94,611,390	C	A	0.762	0.997	ROR2
rs10992131	9	94,611,554	T	C	0.728	0.997	ROR2
rs118134274	9	94,611,731	C	A	0.988	0.971	ROR2
rs375019221	9	94,612,278	A	ATTTCT	0.986	0.97	ROR2
rs7048756	9	94,612,391	T	C	0.728	0.997	ROR2
rs752127192	9	94,612,533	CTTATG	C	0.762	0.996	ROR2
9:94612694_AC_A	9	94,612,694	AC	A	0.762	0.996	ROR2
9:94612963_CTAAG_C	9	94,612,963	CTAAG	C	0.974	0.962	ROR2
rs73651583	9	94,613,155	G	A	0.966	0.997	ROR2
rs7847061	9	94,613,202	A	G	0.67	0.997	ROR2
rs79635708	9	94,613,431	G	A	0.946	0.998	ROR2
rs11789408	9	94,613,497	G	A	0.946	0.997	ROR2
rs7850636	9	94,613,534	T	C	0.762	0.999	ROR2
rs9969713	9	94,613,768	G	A	0.966	0.997	ROR2
rs7864311	9	94,614,074	G	T	0.669	0.998	ROR2
rs116997940	9	94,614,115	G	A	0.98	0.944	ROR2
rs62564599	9	94,614,206	C	T	0.762	1	ROR2
rs575096382	9	94,614,255	T	TA	0.752	0.956	ROR2
rs7865855	9	94,614,602	C	T	0.669	0.998	ROR2
9:94614905_AAAC_A	9	94,614,905	AAAC	A	0.964	0.567	ROR2
9:94614928_CACA_C	9	94,614,928	CACA	C	0.948	0.567	ROR2
rs754228260	9	94,614,931	ACC	A	0.485	0.598	ROR2
rs10992133	9	94,614,933	C	A	0.591	0.898	ROR2
rs116756506	9	94,615,064	T	C	0.967	0.996	ROR2
rs10992134	9	94,615,230	C	T	0.674	0.999	ROR2
rs78044446	9	94,615,586	C	A	0.967	0.996	ROR2
rs7031514	9	94,615,903	A	G	0.966	0.997	ROR2
rs7031562	9	94,615,986	A	G	0.728	0.999	ROR2
rs1881392	9	94,616,030	G	A	0.762	0.999	ROR2
rs56088447	9	94,616,103	G	A	0.762	1	ROR2
rs5899144	9	94,616,290	T	TG	0.669	0.998	ROR2
rs76026028	9	94,616,998	G	C	0.946	0.998	ROR2
rs7039406	9	94,617,098	T	A	0.762	0.999	ROR2
rs10992135	9	94,617,450	A	G	0.728	0.999	ROR2
rs10992136	9	94,617,709	G	A	0.677	0.997	ROR2
9:94618095_CG_C	9	94,618,095	CG	C	0.947	0.996	ROR2
rs73651584	9	94,618,265	T	A	0.966	0.997	ROR2
rs7867837	9	94,618,329	A	G	0.946	0.998	ROR2
rs7871170	9	94,618,456	T	C	0.946	0.998	ROR2
rs117747660	9	94,618,581	G	C	0.986	0.962	ROR2
rs10992137	9	94,619,103	G	A	0.762	0.999	ROR2
rs55956910	9	94,619,276	T	C	0.67	0.998	ROR2
rs10992139	9	94,619,798	G	A	0.762	0.999	ROR2
rs11790201	9	94,619,809	G	A	0.946	0.997	ROR2
rs61160043	9	94,620,205	G	C	0.966	0.996	ROR2
rs12376130	9	94,620,277	A	T	0.7	0.996	ROR2
rs59646330	9	94,620,511	G	A	0.942	0.996	ROR2
rs58089833	9	94,620,607	A	G	0.942	0.996	ROR2
rs7041908	9	94,620,731	C	T	0.966	0.996	ROR2
rs57464764	9	94,620,752	A	G	0.942	0.996	ROR2
rs80340661	9	94,620,849	C	G	0.946	0.999	ROR2
rs74559234	9	94,620,990	T	C	0.946	0.999	ROR2
rs7045226	9	94,621,055	C	T	0.677	0.996	ROR2
rs77300151	9	94,621,186	G	C	0.946	0.999	ROR2
rs7030661	9	94,621,265	A	G	0.677	0.996	ROR2
rs7045708	9	94,621,395	C	A	0.966	0.996	ROR2
rs56308784	9	94,621,508	G	A	0.769	0.995	ROR2
rs78351804	9	94,621,645	C	T	0.966	0.996	ROR2
rs10992140	9	94,621,866	A	G	0.769	0.996	ROR2
rs11791415	9	94,622,451	G	A	0.946	1	ROR2
rs4744105	9	94,622,509	G	A	0.814	0.987	ROR2
rs73517062	9	94,622,536	A	G	0.942	0.997	ROR2
rs57944742	9	94,622,875	A	G	0.942	0.997	ROR2
9:94623525_GGTTTT_G	9	94,623,525	GGTTTT	G	0.967	0.989	ROR2
rs73517065	9	94,623,583	G	A	0.942	0.997	ROR2
rs7866996	9	94,623,611	A	C	0.966	0.995	ROR2
rs11792593	9	94,624,469	G	A	0.942	0.997	ROR2
rs539503617	9	94,624,950	C	CAA	0.947	0.995	ROR2
rs77699899	9	94,625,070	C	T	0.966	0.994	ROR2
rs28802998	9	94,625,560	G	A	0.966	0.994	ROR2
rs10992142	9	94,625,960	G	A	0.737	0.995	ROR2
rs79200307	9	94,625,984	G	A	0.981	0.985	ROR2
rs61199056	9	94,626,039	A	G	0.942	0.997	ROR2
rs56119264	9	94,626,067	A	G	0.881	0.976	ROR2
rs148793963	9	94,626,301	C	T	0.985	0.916	ROR2
rs7850118	9	94,626,543	A	G	0.671	0.996	ROR2
9:94627021_AT_A	9	94,627,021	AT	A	0.948	0.939	ROR2
9:94627026_AG_A	9	94,627,026	AG	A	0.985	0.657	ROR2
rs55864279	9	94,627,108	C	T	0.763	0.997	ROR2
rs11790345	9	94,627,230	C	A	0.942	0.998	ROR2
rs1881386	9	94,627,231	G	A	0.966	0.994	ROR2
rs73517077	9	94,628,300	C	T	0.942	0.996	ROR2
rs4744107	9	94,628,329	T	C	0.678	0.994	ROR2
rs80173338	9	94,628,331	A	G	0.942	0.996	ROR2
rs117798920	9	94,628,344	T	C	0.981	0.988	ROR2
rs4744108	9	94,628,401	G	A	0.77	0.994	ROR2
rs10820907	9	94,628,846	C	T	0.737	0.994	ROR2
rs10992143	9	94,628,882	T	C	0.772	0.993	ROR2
rs7853161	9	94,628,908	G	A	0.946	0.999	ROR2
rs73517083	9	94,629,431	T	C	0.942	0.996	ROR2
rs12552858	9	94,629,592	A	G	0.77	0.994	ROR2
rs72746232	9	94,629,794	G	A	0.882	0.978	ROR2
rs77204157	9	94,630,038	G	A	0.946	0.998	ROR2
rs140443976	9	94,630,064	A	ACAT	0.943	0.983	ROR2
rs146931199	9	94,630,660	C	T	0.983	0.922	ROR2
rs73517090	9	94,630,684	A	T	0.942	0.996	ROR2
rs73517091	9	94,630,948	C	G	0.942	0.996	ROR2
rs73517094	9	94,631,042	G	A	0.942	0.996	ROR2
rs74651754	9	94,631,331	G	A	0.966	0.994	ROR2
rs12002884	9	94,631,376	A	G	0.966	0.994	ROR2
rs10992144	9	94,631,630	C	T	0.683	0.994	ROR2
rs11789730	9	94,631,730	C	T	0.713	0.995	ROR2
rs67944481	9	94,631,759	C	T	0.771	0.995	ROR2
rs138750833	9	94,631,925	C	CT	0.969	0.982	ROR2
rs11790144	9	94,631,985	C	A	0.717	0.995	ROR2
rs117494812	9	94,632,019	T	C	0.946	0.998	ROR2
rs11792667	9	94,632,053	T	C	0.946	0.998	ROR2
rs16907923	9	94,632,749	A	G	0.946	0.998	ROR2
rs11792818	9	94,633,130	A	G	0.946	0.998	ROR2
rs1881387	9	94,633,406	G	A	0.771	0.994	ROR2
rs11793320	9	94,633,498	T	A	0.946	0.998	ROR2
rs12002460	9	94,633,912	C	T	0.966	0.993	ROR2
rs7874281	9	94,634,254	G	A	0.946	0.997	ROR2
rs12554669	9	94,634,668	A	G	0.969	0.962	ROR2
9:94635461_TA_T	9	94,635,461	TA	T	0.953	0.941	ROR2
9:94635486_TCTACCA_T	9	94,635,486	TCTACCA	T	0.717	0.99	ROR2
9:94635690_TA_T	9	94,635,690	TA	T	0.679	0.989	ROR2
rs112068899	9	94,635,734	A	G	0.946	0.994	ROR2

SNP	Chromosome	Position	Effect Allele	Other Allele	Effect Allele Frequency	Imputation quality	Gene
rs80217787	9	94,635,928	T	C	0.946	0.994	ROR2
rs79859663	9	94,636,057	G	C	0.946	0.994	ROR2
rs10992145	9	94,636,075	T	C	0.679	0.991	ROR2
rs11794532	9	94,636,360	A	G	0.946	0.993	ROR2
rs11788423	9	94,636,497	G	A	0.946	0.994	ROR2
rs7045662	9	94,636,514	T	C	0.946	0.993	ROR2
rs7026615	9	94,636,593	C	T	0.946	0.992	ROR2
rs146190680	9	94,636,703	T	TGAG	0.947	0.984	ROR2
rs79327832	9	94,636,726	T	C	0.946	0.994	ROR2
rs139677263	9	94,637,160	T	G	0.946	0.993	ROR2
rs62565674	9	94,637,165	T	C	0.763	0.993	ROR2
rs7033819	9	94,638,008	G	A	0.771	0.991	ROR2
rs11787708	9	94,638,095	A	C	0.946	0.989	ROR2
rs112844159	9	94,638,331	CA	C	0.906	0.775	ROR2
rs549559496	9	94,638,651	C	CA	0.703	0.951	ROR2
rs12684524	9	94,638,804	C	T	0.692	0.992	ROR2
rs12684533	9	94,638,962	C	T	0.695	0.991	ROR2
rs141389723	9	94,639,001	T	C	0.989	0.909	ROR2
rs73519115	9	94,639,418	T	A	0.96	0.991	ROR2
rs62565675	9	94,639,421	C	T	0.694	0.992	ROR2
rs76432051	9	94,639,738	C	T	0.965	0.991	ROR2
rs1528363	9	94,639,795	T	C	0.694	0.992	ROR2
rs1528364	9	94,639,829	T	C	0.694	0.992	ROR2
rs113564515	9	94,640,341	C	CCAGT	0.959	0.982	ROR2
rs1881389	9	94,640,621	G	A	0.489	0.992	ROR2
rs78870900	9	94,640,622	G	A	0.984	0.989	ROR2
rs17516141	9	94,640,800	C	T	0.945	0.937	ROR2
rs6479377	9	94,640,884	T	G	0.686	0.992	ROR2
rs10820908	9	94,640,935	T	C	0.761	0.992	ROR2
rs113052411	9	94,641,249	G	A	0.97	0.961	ROR2
rs10820909	9	94,641,723	G	T	0.761	0.986	ROR2
9:94641791_CT_C	9	94,641,791	CT	C	0.761	0.989	ROR2
rs10761132	9	94,642,129	C	T	0.687	0.993	ROR2
rs4744109	9	94,642,340	T	C	0.761	0.992	ROR2
rs11795340	9	94,642,377	T	C	0.965	0.993	ROR2
rs4744110	9	94,642,450	A	G	0.761	0.992	ROR2
rs77265149	9	94,642,566	C	G	0.956	0.985	ROR2
rs73519130	9	94,642,632	G	A	0.96	0.992	ROR2
rs4744111	9	94,642,762	C	T	0.687	0.993	ROR2
rs77575942	9	94,643,038	C	T	0.965	0.992	ROR2
rs58202197	9	94,643,546	C	G	0.726	0.993	ROR2
rs16907940	9	94,643,616	A	G	0.96	0.992	ROR2
rs56225147	9	94,644,426	G	A	0.96	0.992	ROR2
9:94644692_CA_C	9	94,644,692	CA	C	0.664	0.886	ROR2
rs10820910	9	94,644,814	A	T	0.687	0.993	ROR2
rs12555334	9	94,644,979	C	T	0.726	0.993	ROR2
rs73519140	9	94,645,039	G	A	0.96	0.992	ROR2
rs2030991	9	94,645,339	G	A	0.726	0.993	ROR2
rs7870049	9	94,645,758	C	T	0.687	0.993	ROR2
rs1534533	9	94,645,971	T	A	0.686	0.993	ROR2
rs10512218	9	94,646,318	C	A	0.96	0.993	ROR2
rs11789285	9	94,646,570	A	G	0.963	0.991	ROR2
rs78213816	9	94,646,628	G	A	0.963	0.988	ROR2
rs117878607	9	94,646,748	G	A	0.95	0.979	ROR2
rs572256110	9	94,646,757	T	TA	0.791	0.911	ROR2
rs10820911	9	94,646,797	C	T	0.694	0.996	ROR2
rs10761133	9	94,646,958	T	C	0.715	0.999	ROR2
rs10761134	9	94,646,983	T	C	0.714	0.999	ROR2
rs6479378	9	94,647,600	C	G	0.715	0.999	ROR2
rs112479398	9	94,647,899	G	C	0.966	0.974	ROR2
rs73651595	9	94,648,045	T	C	0.96	0.999	ROR2
rs7867567	9	94,648,219	A	G	0.716	0.999	ROR2
rs6479379	9	94,648,302	T	C	0.717	0.999	ROR2
rs10992149	9	94,648,679	G	A	0.756	0.998	ROR2
9:94648781_CGCACGGCGCG_C	9	94,648,781	CGCACGGCGCG	C	0.961	0.988	ROR2
rs4744112	9	94,648,911	T	G	0.678	0.999	ROR2
rs4744113	9	94,648,931	C	T	0.756	0.998	ROR2
rs12683797	9	94,649,104	G	A	0.756	0.998	ROR2
rs7021744	9	94,649,260	A	T	0.493	0.994	ROR2
rs7858435	9	94,651,415	A	G	0.569	0.995	ROR2
rs10116351	9	94,651,702	A	G	0.604	0.995	ROR2
rs17516460	9	94,651,792	T	C	0.966	0.992	ROR2
rs10116962	9	94,651,871	A	G	0.57	0.995	ROR2
rs11789973	9	94,651,984	T	C	0.756	0.997	ROR2
rs151257238	9	94,652,399	T	C	0.988	0.919	ROR2
rs4744114	9	94,652,581	G	A	0.604	0.995	ROR2
rs10992151	9	94,652,613	G	A	0.756	0.997	ROR2
rs755782206	9	94,653,534	CT	C	0.483	0.856	ROR2
rs59723153	9	94,654,689	C	T	0.756	0.998	ROR2
rs58431919	9	94,654,782	G	C	0.756	0.998	ROR2
rs774864311	9	94,654,872	CTT	C	0.612	0.96	ROR2
rs7047587	9	94,655,021	A	G	0.573	0.996	ROR2
rs7875037	9	94,655,453	A	G	0.848	0.994	ROR2
rs113787122	9	94,655,656	C	T	0.966	0.99	ROR2
rs148920471	9	94,655,770	C	A	0.979	0.984	ROR2
rs10512219	9	94,656,016	A	G	0.851	0.996	ROR2
rs76530119	9	94,656,037	C	G	0.981	0.978	ROR2
rs10992154	9	94,656,057	G	A	0.608	0.997	ROR2
rs4517189	9	94,657,130	C	T	0.756	0.995	ROR2
rs56119295	9	94,657,131	G	A	0.966	0.988	ROR2
rs115132021	9	94,657,553	G	A	0.965	0.995	ROR2
rs74548355	9	94,658,381	T	C	0.966	0.987	ROR2
rs59916937	9	94,658,598	T	C	0.814	0.994	ROR2
rs62565677	9	94,659,219	T	C	0.945	0.935	ROR2
rs75979691	9	94,659,546	T	C	0.925	0.982	ROR2
rs115262789	9	94,659,898	C	T	0.98	0.987	ROR2
rs79163830	9	94,661,036	C	A	0.967	0.985	ROR2
rs75611067	9	94,661,040	T	C	0.967	0.984	ROR2
rs12554679	9	94,661,115	G	A	0.778	0.987	ROR2
rs72746246	9	94,661,537	G	A	0.789	0.973	ROR2
rs118041535	9	94,661,589	G	A	0.965	0.949	ROR2
rs62565678	9	94,661,796	G	A	0.753	0.991	ROR2
rs62565679	9	94,662,092	G	A	0.711	0.99	ROR2
rs73519162	9	94,662,121	C	G	0.96	0.988	ROR2
rs55936817	9	94,662,137	A	C	0.949	0.977	ROR2
rs73519164	9	94,662,180	T	C	0.96	0.988	ROR2
rs4430151	9	94,662,474	A	G	0.711	0.99	ROR2
rs144807648	9	94,662,569	C	T	0.978	0.968	ROR2
rs4275276	9	94,662,604	A	G	0.711	0.991	ROR2
rs11792319	9	94,662,657	A	G	0.751	0.991	ROR2
rs4347043	9	94,662,787	C	T	0.751	0.991	ROR2
rs79679524	9	94,662,816	G	A	0.96	0.988	ROR2
rs4347044	9	94,662,817	C	T	0.751	0.991	ROR2
rs111444292	9	94,662,942	A	G	0.96	0.988	ROR2
rs73519170	9	94,663,063	T	C	0.96	0.988	ROR2
rs73519172	9	94,663,359	C	T	0.96	0.987	ROR2
rs73519174	9	94,663,647	T	C	0.96	0.988	ROR2
9:94663674_GTCCT_G	9	94,663,674	GTCCT	G	0.96	0.985	ROR2
rs11795130	9	94,664,004	G	A	0.569	0.989	ROR2
rs145203256	9	94,664,074	G	GGCGACAGGGCAA	0.731	0.949	ROR2
rs73519176	9	94,664,203	G	T	0.962	0.987	ROR2
rs57688942	9	94,664,869	C	G	0.751	0.991	ROR2
rs59917975	9	94,666,045	C	T	0.962	0.987	ROR2
rs73519181	9	94,666,197	G	T	0.959	0.988	ROR2
rs770738493	9	94,666,332	TTG	T	0.754	0.981	ROR2
rs2252879	9	94,666,693	C	T	0.976	0.949	ROR2
rs71494487	9	94,666,793	C	G	0.93	0.946	ROR2
rs10820915	9	94,666,958	T	C	0.761	0.985	ROR2
rs1881384	9	94,666,993	C	A	0.761	0.985	ROR2
9:94667336_CCACA_CCA	9	94,667,336	CCACA	CCA	0.76	0.981	ROR2
9:94667336_CCA_C	9	94,667,336	CCA	C	0.761	0.982	ROR2
rs192499004	9	94,667,426	A	G	0.985	0.97	ROR2
rs55897887	9	94,667,447	C	CCA	0.761	0.982	ROR2
rs117457033	9	94,667,508	C	T	0.976	0.953	ROR2
9:94667596_CACAT_C	9	94,667,596	CACAT	C	0.96	0.982	ROR2
rs2872123	9	94,668,300	A	C	0.609	0.982	ROR2
rs1919104	9	94,668,335	G	C	0.962	0.987	ROR2
rs12340061	9	94,668,588	G	A	0.962	0.987	ROR2
rs3905385	9	94,668,733	T	C	0.751	0.992	ROR2
rs12685595	9	94,669,125	A	C	0.567	0.988	ROR2
rs2030992	9	94,669,239	G	T	0.751	0.992	ROR2
rs76515420	9	94,669,409	T	C	0.96	0.988	ROR2
rs146296132	9	94,669,566	A	C	0.984	0.905	ROR2
rs2697194	9	94,670,971	C	T	0.023	0.868	ROR2
rs10659451	9	94,671,200	G	GAA	0.708	0.986	ROR2
rs539335798	9	94,671,795	AT	A	0.681	0.898	ROR2
rs2697195	9	94,671,863	G	A	0.977	0.95	ROR2
9:94672102_ATTCT_A	9	94,672,102	ATTCT	A	0.83	0.826	ROR2
rs7875069	9	94,672,175	T	C	0.959	0.989	ROR2
rs7872177	9	94,672,364	A	G	0.959	0.988	ROR2
rs7857164	9	94,672,421	C	T	0.959	0.988	ROR2
rs112000577	9	94,672,754	C	CA	0.897	0.979	ROR2
9:94672950_CT_C	9	94,672,950	CT	C	0.921	0.792	ROR2
rs143138789	9	94,673,238	G	A	0.96	0.989	ROR2
rs147488568	9	94,673,256	T	C	0.989	0.951	ROR2

SNP	Chromosome	Position	Effect Allele	Other Allele	Effect Allele Frequency	Imputation quality	Gene
rs76784905	9	94,673,381	G	A	0.96	0.989	ROR2
rs76981590	9	94,673,508	G	C	0.96	0.989	ROR2
rs147217097	9	94,673,781	A	AAAC	0.959	0.986	ROR2
rs76381086	9	94,673,912	C	T	0.959	0.988	ROR2
rs10114686	9	94,673,985	A	G	0.962	0.987	ROR2
rs150829980	9	94,674,282	A	ACTC	0.981	0.965	ROR2
9:94674424_AATTAG_A	9	94,674,424	AATTAG	A	0.96	0.986	ROR2
rs78408863	9	94,674,582	T	C	0.96	0.989	ROR2
rs117913604	9	94,674,626	C	T	0.979	0.989	ROR2
rs10992158	9	94,674,927	T	C	0.749	0.992	ROR2
rs78819566	9	94,675,087	G	A	0.959	0.989	ROR2
rs111513989	9	94,675,146	C	T	0.898	0.985	ROR2
rs148291124	9	94,675,262	G	T	0.959	0.989	ROR2
rs143014651	9	94,675,351	C	G	0.897	0.986	ROR2
rs146873314	9	94,675,658	A	AT	0.96	0.981	ROR2
rs7873326	9	94,676,039	C	A	0.959	0.989	ROR2
rs6479381	9	94,676,296	T	C	0.959	0.989	ROR2
9:94676358_GTTA_G	9	94,676,358	GTTA	G	0.96	0.987	ROR2
rs6479382	9	94,676,625	T	G	0.959	0.989	ROR2
rs75508548	9	94,676,643	C	T	0.897	0.986	ROR2
rs762423762	9	94,677,249	CCT	C	0.753	0.988	ROR2
rs78717190	9	94,677,662	A	G	0.959	0.99	ROR2
rs77157573	9	94,677,731	T	C	0.959	0.99	ROR2
rs10992159	9	94,677,777	T	C	0.709	0.992	ROR2
rs62565747	9	94,678,097	G	A	0.752	0.992	ROR2
rs11464558	9	94,678,627	C	CT	0.709	0.989	ROR2
rs6479383	9	94,679,355	C	T	0.712	0.993	ROR2
rs76753161	9	94,679,608	C	T	0.954	0.982	ROR2
rs74680418	9	94,680,030	G	C	0.979	0.989	ROR2
rs59195968	9	94,680,130	A	G	0.962	0.988	ROR2
rs60351369	9	94,680,289	C	T	0.962	0.987	ROR2
rs112388009	9	94,680,531	C	CAGA	0.637	0.946	ROR2
rs34777436	9	94,680,533	G	GAAC	0.629	0.955	ROR2
9:94680545_CA_C	9	94,680,545	CA	C	0.93	0.747	ROR2
9:94680647_TC_T	9	94,680,647	TC	T	0.979	0.978	ROR2
rs1406202	9	94,681,273	T	C	0.9	0.99	ROR2
9:94681305_ACCACT_A	9	94,681,305	ACCACT	A	0.962	0.983	ROR2
rs7866188	9	94,681,588	A	C	0.964	0.993	ROR2
rs80330767	9	94,681,615	A	G	0.979	0.99	ROR2
rs78705383	9	94,681,959	T	C	0.964	0.994	ROR2
rs7042585	9	94,681,965	A	G	0.942	0.992	ROR2
rs74764287	9	94,682,100	A	G	0.944	0.987	ROR2
rs61266489	9	94,682,448	T	G	0.962	0.988	ROR2
rs1892269	9	94,682,513	G	A	0.979	0.99	ROR2
rs12684333	9	94,682,714	A	G	0.942	0.992	ROR2
rs10116326	9	94,682,964	C	G	0.904	0.991	ROR2
rs12684752	9	94,682,990	T	C	0.942	0.993	ROR2
rs16907979	9	94,683,050	T	C	0.942	0.992	ROR2
rs79897735	9	94,683,075	G	A	0.978	0.989	ROR2
rs79152318	9	94,683,141	G	A	0.942	0.992	ROR2
rs117703609	9	94,683,355	T	C	0.964	0.995	ROR2
rs79581461	9	94,683,408	C	G	0.942	0.993	ROR2
rs77335334	9	94,683,518	A	G	0.964	0.995	ROR2
rs10820916	9	94,683,714	G	A	0.771	0.998	ROR2
rs147956035	9	94,683,793	C	T	0.986	0.941	ROR2
rs12340427	9	94,684,010	C	T	0.905	0.989	ROR2
rs12349258	9	94,684,011	G	A	0.963	0.984	ROR2
rs117288483	9	94,684,013	C	T	0.979	0.989	ROR2
rs145341048	9	94,684,070	G	A	0.964	0.995	ROR2
9:94684138_CA_C	9	94,684,138	CA	C	0.727	0.96	ROR2
rs7035117	9	94,684,351	G	A	0.963	0.989	ROR2
9:94684373_AAACAACAGCAAC_A	9	94,684,373	AAACAACAGCAAC	A	0.981	0.907	ROR2
rs201246049	9	94,684,391	C	A	0.986	0.723	ROR2
9:94684397_CAACAACAACA_C	9	94,684,397	CAACAACAACA	C	0.964	0.991	ROR2
9:94684400_CAACAACA_C	9	94,684,400	CAACAACA	C	0.771	0.997	ROR2
rs1892267	9	94,684,700	A	C	0.979	0.99	ROR2
rs5000046	9	94,684,747	A	T	0.978	0.978	ROR2
rs5000045	9	94,684,762	G	A	0.978	0.989	ROR2
rs5000044	9	94,684,763	T	C	0.978	0.988	ROR2
rs7866775	9	94,684,859	C	T	0.735	0.999	ROR2
rs10046835	9	94,685,030	C	T	0.962	0.989	ROR2
rs7853136	9	94,685,210	A	C	0.676	0.997	ROR2
rs10992160	9	94,685,585	T	A	0.962	0.989	ROR2
rs10820917	9	94,685,761	C	G	0.735	1	ROR2
rs117692403	9	94,685,768	A	C	0.979	0.989	ROR2
rs2001917	9	94,685,926	C	T	0.904	0.992	ROR2
rs28524099	9	94,686,452	C	T	0.962	0.99	ROR2
rs10820918	9	94,686,453	A	G	0.676	0.997	ROR2
rs7045073	9	94,686,736	C	T	0.962	0.99	ROR2
rs142274760	9	94,687,003	A	T	0.964	0.992	ROR2
rs535189760	9	94,687,063	T	TA	0.768	0.968	ROR2
rs7875147	9	94,687,508	G	A	0.964	0.996	ROR2
rs1892263	9	94,687,601	G	A	0.771	0.999	ROR2
rs57980458	9	94,687,748	A	G	0.953	0.945	ROR2
rs563298882	9	94,687,768	G	A	0.983	0.837	ROR2
rs7875439	9	94,687,772	G	A	0.934	0.619	ROR2
rs7862013	9	94,687,785	A	C	0.735	1	ROR2
rs10992161	9	94,687,927	T	C	0.771	0.999	ROR2
rs189923570	9	94,688,402	G	A	0.985	0.955	ROR2
rs10992162	9	94,688,429	C	T	0.735	0.999	ROR2
9:94688501_TA_T	9	94,688,501	TA	T	0.908	0.754	ROR2
rs34847971	9	94,688,664	CA	C	0.707	0.973	ROR2
rs6479384	9	94,688,705	A	G	0.697	0.998	ROR2
rs56013831	9	94,688,741	T	C	0.771	0.999	ROR2
rs56309039	9	94,688,806	C	T	0.774	0.988	ROR2
rs34550588	9	94,688,825	CA	C	0.693	0.904	ROR2
rs73520858	9	94,688,955	A	T	0.962	0.99	ROR2
9:94689343_TAAG_T	9	94,689,343	TAAG	T	0.772	0.998	ROR2
rs10992163	9	94,689,515	C	G	0.962	0.989	ROR2
rs10992165	9	94,689,777	A	G	0.738	0.994	ROR2
rs10992166	9	94,689,796	G	A	0.771	0.999	ROR2
rs147313353	9	94,689,967	T	C	0.979	0.99	ROR2
rs55884190	9	94,690,135	C	T	0.733	0.997	ROR2
rs10992167	9	94,690,190	T	C	0.733	0.997	ROR2
rs2312737	9	94,690,412	C	T	0.961	0.984	ROR2
rs148528853	9	94,690,477	C	T	0.979	0.992	ROR2
rs10992168	9	94,690,621	G	A	0.771	0.999	ROR2
rs78050298	9	94,690,760	A	G	0.944	0.988	ROR2
rs75412536	9	94,690,884	T	G	0.964	0.995	ROR2
rs10118781	9	94,691,315	C	G	0.962	0.99	ROR2
rs78556670	9	94,691,338	T	C	0.942	0.993	ROR2
rs10118792	9	94,691,380	C	T	0.675	0.997	ROR2
rs10118816	9	94,691,478	C	T	0.676	0.997	ROR2
rs10122619	9	94,691,479	T	C	0.963	0.985	ROR2
rs10124760	9	94,691,555	G	T	0.619	0.995	ROR2
rs76684062	9	94,691,627	G	A	0.979	0.989	ROR2
rs16908003	9	94,691,644	T	A	0.978	0.987	ROR2
rs10124796	9	94,691,668	G	A	0.962	0.989	ROR2
rs75117900	9	94,691,826	G	C	0.964	0.996	ROR2
rs76004798	9	94,692,165	C	T	0.944	0.989	ROR2
rs7036134	9	94,692,350	T	C	0.979	0.991	ROR2
rs7046846	9	94,692,417	G	A	0.964	0.995	ROR2
rs7032998	9	94,692,480	A	G	0.964	0.995	ROR2
rs7048052	9	94,692,634	C	A	0.964	0.995	ROR2
rs1881391	9	94,692,698	G	A	0.619	0.995	ROR2
rs7033397	9	94,692,772	A	G	0.942	0.993	ROR2
rs7036525	9	94,692,798	A	G	0.942	0.993	ROR2
rs148793849	9	94,693,069	C	T	0.988	0.957	ROR2
rs79005820	9	94,693,135	T	G	0.944	0.989	ROR2
rs10820921	9	94,693,244	C	T	0.771	0.998	ROR2
rs79153684	9	94,693,454	T	A	0.964	0.995	ROR2
rs77999883	9	94,693,929	A	G	0.964	0.995	ROR2
rs58964193	9	94,694,361	T	C	0.962	0.99	ROR2
rs74663054	9	94,694,630	G	A	0.989	0.915	ROR2
rs6479385	9	94,694,656	A	G	0.641	0.995	ROR2
rs6479386	9	94,694,690	C	T	0.641	0.995	ROR2
9:94694784_C_CA	9	94,694,784	C	CA	0.714	0.978	ROR2
rs144203325	9	94,694,914	C	G	0.986	0.924	ROR2
rs530773101	9	94,694,943	C	CA	0.976	0.944	ROR2
rs146575182	9	94,694,948	A	C	0.964	0.995	ROR2
rs118095543	9	94,694,970	A	G	0.964	0.995	ROR2
rs6479387	9	94,695,011	T	C	0.619	0.994	ROR2
rs7029733	9	94,695,150	C	A	0.677	0.995	ROR2
rs77094301	9	94,695,329	G	A	0.979	0.99	ROR2
rs117106774	9	94,695,369	C	A	0.979	0.991	ROR2
rs3932420	9	94,695,513	G	A	0.771	0.998	ROR2
rs7857924	9	94,695,554	C	T	0.964	0.994	ROR2
rs117503609	9	94,695,657	A	G	0.948	0.973	ROR2
rs76926693	9	94,695,670	A	T	0.978	0.985	ROR2
rs56747141	9	94,696,096	T	A	0.679	0.995	ROR2
rs3858082	9	94,696,219	A	C	0.966	0.994	ROR2
rs2049705	9	94,696,362	T	C	0.965	0.994	ROR2
rs143267816	9	94,696,390	C	CG	0.966	0.982	ROR2
rs73651600	9	94,696,500	G	T	0.953	0.987	ROR2
rs2049704	9	94,696,509	T	G	0.966	0.994	ROR2

SNP	Chromosome	Position	Effect Allele	Other Allele	Effect Allele Frequency	Imputation quality	Gene
rs139803808	9	94,696,623	T	TA	0.944	0.988	ROR2
rs118120292	9	94,696,777	C	T	0.955	0.978	ROR2
rs7034006	9	94,696,779	G	A	0.717	0.996	ROR2
rs7038017	9	94,696,852	C	T	0.623	0.995	ROR2
rs7037255	9	94,696,954	G	A	0.623	0.996	ROR2
rs57110729	9	94,696,962	T	TAG	0.979	0.987	ROR2
rs7038397	9	94,697,108	C	A	0.717	0.996	ROR2
rs10820922	9	94,697,364	T	G	0.781	0.995	ROR2
rs7038823	9	94,697,487	C	T	0.682	0.996	ROR2
rs73520881	9	94,697,549	A	T	0.962	0.991	ROR2
rs10992172	9	94,697,948	C	T	0.717	0.996	ROR2
rs10992173	9	94,697,987	G	A	0.978	0.992	ROR2
rs7043019	9	94,698,348	C	T	0.962	0.993	ROR2
rs7856148	9	94,698,511	A	G	0.962	0.994	ROR2
rs75616035	9	94,698,645	C	T	0.978	0.989	ROR2
rs7866702	9	94,698,842	A	G	0.85	0.994	ROR2
rs16908021	9	94,698,940	T	C	0.979	0.994	ROR2
rs10992175	9	94,699,109	G	A	0.962	0.988	ROR2
rs534010322	9	94,699,210	G	A	0.944	0.99	ROR2
rs60235289	9	94,700,062	C	T	0.94	0.994	ROR2
rs10115684	9	94,700,342	G	A	0.906	0.995	ROR2
rs10992176	9	94,700,355	T	A	0.978	0.992	ROR2
rs72746290	9	94,700,584	T	A	0.717	0.996	ROR2
rs75326261	9	94,701,033	T	G	0.979	0.994	ROR2
rs10992177	9	94,701,623	G	A	0.979	0.994	ROR2
rs1534534	9	94,701,825	C	T	0.682	0.997	ROR2
9:94701988_AAAG_A	9	94,701,988	AAAG	A	0.985	0.977	ROR2
rs75555389	9	94,702,244	C	G	0.979	0.994	ROR2
rs10115262	9	94,702,266	T	C	0.962	0.995	ROR2
rs79492899	9	94,702,433	G	A	0.981	0.99	ROR2
rs73520889	9	94,702,701	T	C	0.962	0.995	ROR2
9:94702838_CTG_C	9	94,702,838	CTG	C	0.979	0.97	ROR2
rs767062211	9	94,702,876	CT	C	0.944	0.806	ROR2
9:94702986_CCTGTCT_C	9	94,702,986	CCTGTCT	C	0.623	0.994	ROR2
rs112363424	9	94,703,099	G	C	0.962	0.996	ROR2
rs117369959	9	94,703,181	C	A	0.944	0.995	ROR2
rs12551564	9	94,703,491	A	G	0.79	0.997	ROR2
rs75076910	9	94,703,771	T	C	0.966	0.997	ROR2
rs7871522	9	94,703,861	G	A	0.717	0.998	ROR2
rs10116249	9	94,704,033	A	G	0.623	0.998	ROR2
rs138391904	9	94,704,048	G	A	0.953	0.988	ROR2
rs10125384	9	94,704,110	C	T	0.644	0.998	ROR2
rs56057700	9	94,704,214	A	AG	0.683	0.997	ROR2
rs10125466	9	94,704,474	C	G	0.645	0.999	ROR2
rs10992178	9	94,704,686	G	A	0.979	0.997	ROR2
rs10992179	9	94,705,123	C	T	0.979	0.997	ROR2
rs7862779	9	94,705,255	A	G	0.645	0.999	ROR2
rs79736533	9	94,705,310	A	G	0.944	0.998	ROR2
rs7863167	9	94,705,498	A	G	0.623	0.999	ROR2
rs74960244	9	94,705,697	G	T	0.966	0.998	ROR2
rs10820925	9	94,706,668	C	A	0.717	0.999	ROR2
rs117767190	9	94,706,924	G	A	0.944	0.999	ROR2
rs7027147	9	94,707,128	G	A	0.962	0.999	ROR2
rs72746299	9	94,707,171	C	T	0.773	0.999	ROR2
rs7047326	9	94,707,277	T	G	0.623	0.999	ROR2
9:94707361_CA_C	9	94,707,361	CA	C	0.862	0.744	ROR2
rs7048699	9	94,708,403	A	G	0.623	0.999	ROR2
rs78377272	9	94,708,626	G	T	0.944	0.999	ROR2
rs12344469	9	94,708,676	A	T	0.962	0.999	ROR2
rs138188217	9	94,709,044	A	AATG	0.622	0.995	ROR2
rs62565755	9	94,709,860	G	A	0.772	0.999	ROR2
rs7048508	9	94,709,888	C	A	0.963	0.989	ROR2
rs7036958	9	94,709,889	T	G	0.963	0.99	ROR2
rs55730193	9	94,710,850	C	A	0.962	0.999	ROR2
rs117620263	9	94,711,166	C	T	0.944	1	ROR2
rs111823595	9	94,711,300	A	C	0.944	1	ROR2
rs10120655	9	94,711,436	G	A	0.962	0.999	ROR2
rs7850309	9	94,711,649	G	C	0.661	1	ROR2
rs6479388	9	94,711,672	T	C	0.623	1	ROR2
rs7045603	9	94,711,931	T	C	0.962	0.999	ROR2
rs141262722	9	94,712,336	C	T	0.986	0.932	ROR2
rs357565	9	98,205,443	C	A	0.716	0.952	PTCH1
rs28380046	9	98,206,400	A	T	0.981	0.873	PTCH1
rs759856507	9	98,206,791	G	GCA	0.923	0.966	PTCH1
rs16909865	9	98,207,302	C	G	0.924	0.991	PTCH1
rs147342582	9	98,207,926	G	GAAGT	0.911	0.984	PTCH1
rs59925771	9	98,210,941	G	T	0.899	0.99	PTCH1
rs138968535	9	98,210,952	G	A	0.986	0.99	PTCH1
rs2282043	9	98,212,608	C	T	0.909	0.991	PTCH1
rs532039375	9	98,213,286	A	AG	0.769	0.981	PTCH1
rs9632916	9	98,213,728	C	G	0.767	0.986	PTCH1
rs148664376	9	98,213,760	G	A	0.985	0.959	PTCH1
rs9632897	9	98,214,367	A	G	0.762	0.988	PTCH1
rs147794976	9	98,214,610	GAC	G	0.83	0.832	PTCH1
rs9632898	9	98,214,758	C	T	0.762	0.988	PTCH1
rs75259420	9	98,215,185	T	C	0.909	0.992	PTCH1
rs2134721	9	98,215,306	C	T	0.762	0.988	PTCH1
rs28583690	9	98,215,631	T	C	0.909	0.992	PTCH1
rs28620532	9	98,216,876	A	G	0.65	0.99	PTCH1
rs28457693	9	98,217,348	A	G	0.893	0.993	PTCH1
rs28701981	9	98,217,581	T	C	0.65	0.99	PTCH1
rs531236471	9	98,218,474	G	GC	0.63	0.984	PTCH1
rs118084812	9	98,220,037	G	A	0.973	0.981	PTCH1
rs2236406	9	98,221,861	T	C	0.65	0.993	PTCH1
rs17368876	9	98,222,665	C	T	0.762	0.993	PTCH1
rs142978977	9	98,222,870	G	C	0.985	0.965	PTCH1
9:98222945_TG_T	9	98,222,945	TG	T	0.978	0.856	PTCH1
rs2274692	9	98,224,360	C	G	0.65	0.994	PTCH1
rs28377268	9	98,225,056	G	T	0.892	0.993	PTCH1
9:98225452_CA_C	9	98,225,452	CA	C	0.76	0.993	PTCH1
rs28571635	9	98,225,658	C	T	0.778	0.993	PTCH1
rs867452	9	98,226,066	G	A	0.892	0.997	PTCH1
rs28493225	9	98,226,307	C	T	0.908	0.997	PTCH1
rs28494679	9	98,226,395	A	C	0.892	0.998	PTCH1
rs28535536	9	98,226,702	T	C	0.892	0.998	PTCH1
rs59015093	9	98,227,415	C	T	0.892	0.998	PTCH1
rs16909892	9	98,227,810	G	T	0.892	0.998	PTCH1
rs28394554	9	98,228,225	C	T	0.763	0.999	PTCH1
rs2066829	9	98,229,389	C	G	0.643	1	PTCH1
rs2036963	9	98,230,545	C	T	0.909	0.999	PTCH1
rs28711421	9	98,230,907	T	C	0.908	1	PTCH1
rs2227970	9	98,231,084	T	C	0.983	0.999	PTCH1
rs554636683	9	98,231,346	C	G	0.921	0.71	PTCH1
rs56149656	9	98,232,754	A	G	0.892	1	PTCH1
rs142257681	9	98,233,198	C	A	0.988	1	PTCH1
rs28495472	9	98,234,097	A	G	0.643	0.999	PTCH1
rs28442014	9	98,234,563	C	T	0.762	0.999	PTCH1
9:98234650_GT_G	9	98,234,650	GT	G	0.762	0.999	PTCH1
rs62558340	9	98,234,903	C	T	0.762	0.998	PTCH1
rs28706464	9	98,235,310	T	C	0.892	1	PTCH1
rs539078574	9	98,235,824	AT	A	0.588	0.943	PTCH1
rs28733891	9	98,235,859	T	C	0.892	1	PTCH1
rs28535421	9	98,235,885	A	G	0.892	0.998	PTCH1
rs16909902	9	98,236,202	A	G	0.904	1	PTCH1
rs3824488	9	98,236,664	C	T	0.909	0.999	PTCH1
rs16909904	9	98,236,865	C	T	0.908	1	PTCH1
rs28472890	9	98,237,142	G	C	0.984	0.967	PTCH1
rs2236407	9	98,237,796	A	G	0.643	0.999	PTCH1
rs3780573	9	98,239,503	G	A	0.904	0.999	PTCH1
rs28448271	9	98,239,730	G	A	0.904	0.999	PTCH1
rs2297086	9	98,240,120	G	A	0.643	0.999	PTCH1
rs2274694	9	98,242,112	A	G	0.762	0.997	PTCH1
rs45619434	9	98,242,560	C	T	0.984	0.879	PTCH1
rs2297087	9	98,242,925	A	G	0.762	0.997	PTCH1
rs2297088	9	98,242,984	G	A	0.643	0.997	PTCH1
rs28520225	9	98,243,187	A	C	0.892	0.998	PTCH1
rs55727070	9	98,243,203	A	T	0.946	0.805	PTCH1
rs28446116	9	98,243,385	A	G	0.892	0.997	PTCH1
rs80155616	9	98,243,868	C	T	0.909	0.997	PTCH1
rs28372566	9	98,244,978	C	T	0.892	0.997	PTCH1
rs28510415	9	98,245,026	A	G	0.904	0.996	PTCH1
rs35938191	9	98,245,391	G	GA	0.892	0.993	PTCH1
9:98245546_CAA_C	9	98,245,546	CAA	C	0.909	0.993	PTCH1
rs551163039	9	98,245,547	A	T	0.986	0.716	PTCH1
rs568812460	9	98,245,548	A	C	0.986	0.716	PTCH1
rs28488553	9	98,245,855	T	C	0.762	0.995	PTCH1
rs17369383	9	98,245,974	A	G	0.762	0.995	PTCH1
rs146921950	9	98,246,654	C	T	0.987	0.946	PTCH1
rs28474857	9	98,247,204	C	T	0.909	0.995	PTCH1
rs28673156	9	98,247,790	C	T	0.904	0.995	PTCH1
rs2282040	9	98,248,328	A	G	0.904	0.995	PTCH1
rs2282041	9	98,248,387	A	G	0.892	0.995	PTCH1
rs3215874	9	98,248,431	G	GA	0.904	0.993	PTCH1
rs2282042	9	98,248,635	C	A	0.644	0.994	PTCH1
rs138972069	9	98,248,746	T	C	0.987	0.944	PTCH1

SNP	Chromosome	Position	Effect Allele	Other Allele	Effect Allele Frequency	Imputation quality	Gene
rs78400819	9	98,248,780	C	T	0.989	0.889	PTCH1
rs28504341	9	98,249,963	C	T	0.763	0.993	PTCH1
rs79057214	9	98,250,674	T	C	0.904	0.995	PTCH1
rs144971676	9	98,251,898	T	C	0.986	0.944	PTCH1
rs570275513	9	98,252,249	A	AT	0.983	0.998	PTCH1
rs28411363	9	98,252,313	T	C	0.762	0.992	PTCH1
rs141812150	9	98,252,508	C	A	0.977	0.962	PTCH1
rs372413212	9	98,252,802	G	C	0.99	0.995	PTCH1
rs28557305	9	98,252,847	G	A	0.644	0.994	PTCH1
rs117493439	9	98,253,211	C	T	0.983	1	PTCH1
rs28418339	9	98,253,476	G	A	0.909	0.994	PTCH1
rs79127746	9	98,254,185	T	C	0.982	0.935	PTCH1
rs28831479	9	98,254,526	C	A	0.74	0.993	PTCH1
rs61420024	9	98,255,103	A	AG	0.644	0.992	PTCH1
rs539286190	9	98,256,083	C	CAA	0.906	0.985	PTCH1
rs10512249	9	98,256,309	G	A	0.905	0.994	PTCH1
rs28633576	9	98,257,305	C	T	0.783	0.982	PTCH1
rs28504650	9	98,257,842	C	T	0.646	0.994	PTCH1
9:98259771_CT_C	9	98,259,771	CT	C	0.669	1	PTCH1
rs141110373	9	98,261,390	C	T	0.984	0.901	PTCH1
rs28489210	9	98,261,766	G	A	0.984	0.891	PTCH1
rs60417486	9	98,262,178	G	A	0.91	0.993	PTCH1
rs60150206	9	98,262,223	G	A	0.909	0.993	PTCH1
rs28468571	9	98,262,735	A	G	0.776	0.993	PTCH1
rs28629854	9	98,262,833	A	G	0.669	0.996	PTCH1
rs753518502	9	98,263,821	T	TA	0.701	0.945	PTCH1
rs28427480	9	98,264,942	A	C	0.905	0.992	PTCH1
rs148786069	9	98,265,151	G	A	0.983	0.926	PTCH1
rs201930449	9	98,265,699	A	ATC	0.968	0.967	PTCH1
rs16909919	9	98,265,780	C	T	0.669	0.992	PTCH1
rs16909922	9	98,265,901	A	G	0.905	0.992	PTCH1
rs4448343	9	98,266,370	A	G	0.669	0.991	PTCH1
rs28446321	9	98,266,855	T	A	0.909	0.992	PTCH1
rs71499905	9	98,267,643	A	T	0.973	0.999	PTCH1
rs28620668	9	98,267,746	T	G	0.671	0.991	PTCH1
rs370727606	9	98,268,396	G	A	0.989	0.998	PTCH1
rs191867316	9	98,268,477	G	T	0.989	0.999	PTCH1
rs145922731	9	98,269,098	G	A	0.989	0.999	PTCH1
9:98269418_CG_C	9	98,269,418	CG	C	0.766	0.984	PTCH1
rs144831338	9	98,269,490	G	C	0.987	0.896	PTCH1
rs1355620	9	98,270,168	A	G	0.677	0.973	PTCH1
rs1355619	9	98,270,171	A	G	0.677	0.972	PTCH1
9:98270316_TGTGA_T	9	98,270,316	TGTGA	T	0.859	0.898	PTCH1
rs28475503	9	98,270,324	A	T	0.983	0.974	PTCH1
rs191458964	9	98,270,362	T	G	0.988	0.938	PTCH1
rs569747736	9	98,272,284	T	TA	0.794	0.976	PTCH1
rs572627686	9	98,272,293	A	AC	0.985	0.938	PTCH1
rs3758301	9	98,272,464	T	C	0.778	0.988	PTCH1
rs3758302	9	98,272,630	G	C	0.671	0.99	PTCH1
rs181375055	9	98,273,188	G	T	0.989	0.999	PTCH1
rs145965565	9	98,273,305	T	G	0.905	0.993	PTCH1
9:98273568_TCTC_T	9	98,273,568	TCTC	T	0.988	0.843	PTCH1
rs28412122	9	98,273,791	G	A	0.776	0.988	PTCH1
rs1877456	9	98,275,097	C	G	0.776	0.988	PTCH1
rs75614054	9	98,275,789	C	T	0.91	0.993	PTCH1
rs28485705	9	98,276,105	C	G	0.78	0.988	PTCH1
rs28410513	9	98,276,371	G	T	0.777	0.986	PTCH1
rs28491365	9	98,276,374	T	A	0.777	0.986	PTCH1
rs28536201	9	98,276,753	C	T	0.671	0.99	PTCH1
rs79576312	9	98,276,931	A	T	0.875	0.822	PTCH1
9:98277367_AGTACCACC_A	9	98,277,367	AGTACCACC	A	0.778	0.983	PTCH1
rs28496034	9	98,278,332	C	G	0.671	0.99	PTCH1
rs113154802	9	98,278,413	C	T	0.908	0.993	PTCH1
rs28591501	9	98,278,644	C	A	0.671	0.983	PTCH1
rs142761971	9	98,279,188	T	A	0.973	0.995	PTCH1
rs118133906	9	98,279,234	C	T	0.985	0.997	PTCH1
rs41276090	10	34,400,093	G	A	0.976	0.955	PARD3
rs11009645	10	34,401,759	A	G	0.749	0.995	PARD3
10:34402424_AACAC_A	10	34,402,424	AACAC	A	0.99	0.921	PARD3
10:34402424_AAC_A	10	34,402,424	AAC	A	0.721	0.904	PARD3
rs11009647	10	34,403,117	G	A	0.728	0.987	PARD3
rs3862565	10	34,403,515	T	C	0.73	0.991	PARD3
rs3862566	10	34,404,005	G	A	0.74	0.99	PARD3
rs79900387	10	34,404,182	A	G	0.983	0.912	PARD3
rs117953632	10	34,404,514	G	A	0.983	0.937	PARD3
10:34404632_AT_A	10	34,404,632	AT	A	0.741	0.968	PARD3
rs11009650	10	34,404,663	C	T	0.74	0.989	PARD3
rs10763969	10	34,404,781	C	G	0.731	0.989	PARD3
rs10763970	10	34,404,997	T	C	0.729	0.988	PARD3
rs12245909	10	34,406,231	A	C	0.988	0.951	PARD3
rs191928089	10	34,406,699	C	T	0.987	0.975	PARD3
rs12264982	10	34,408,467	G	A	0.989	0.985	PARD3
rs11009651	10	34,408,636	T	C	0.731	0.987	PARD3
rs3004928	10	34,408,745	A	C	0.574	0.981	PARD3
rs10466050	10	34,409,630	A	C	0.733	0.987	PARD3
rs559898489	10	34,409,993	G	GA	0.739	0.959	PARD3
rs7909689	10	34,410,160	C	G	0.733	0.987	PARD3
rs11009653	10	34,410,212	T	G	0.733	0.987	PARD3
rs10159831	10	34,410,457	A	G	0.733	0.987	PARD3
rs7899786	10	34,411,419	T	C	0.743	0.985	PARD3
rs7899792	10	34,411,427	T	C	0.743	0.985	PARD3
rs7918316	10	34,411,961	C	T	0.733	0.987	PARD3
rs7903640	10	34,412,015	T	C	0.733	0.988	PARD3
rs2384076	10	34,412,080	A	C	0.037	0.938	PARD3
rs7918565	10	34,412,176	A	G	0.733	0.987	PARD3
rs529018698	10	34,412,222	A	AT	0.733	0.978	PARD3
rs11009654	10	34,412,516	G	T	0.98	0.949	PARD3
rs7918987	10	34,412,537	A	C	0.733	0.987	PARD3
rs7919435	10	34,412,628	G	T	0.733	0.988	PARD3
rs3004929	10	34,414,865	G	A	0.573	0.984	PARD3
rs4126706	10	34,415,490	C	T	0.733	0.988	PARD3
rs1854363	10	34,415,512	C	T	0.733	0.988	PARD3
rs76019755	10	34,416,182	G	C	0.979	0.961	PARD3
rs113374271	10	34,416,769	A	AATGT	0.733	0.982	PARD3
rs1415546	10	34,417,835	A	G	0.685	0.99	PARD3
rs1415547	10	34,417,876	T	C	0.695	0.989	PARD3
rs373617192	10	34,418,614	G	T	0.963	0.534	PARD3
rs1765173	10	34,419,273	C	G	0.396	0.99	PARD3
rs1765172	10	34,419,588	C	T	0.396	0.99	PARD3
rs1765171	10	34,419,786	T	C	0.396	0.99	PARD3
rs1765170	10	34,419,901	T	C	0.396	0.991	PARD3
rs970166	10	34,420,607	G	A	0.696	0.991	PARD3
10:34420742_CT_C	10	34,420,742	CT	C	0.682	0.973	PARD3
rs2480016	10	34,421,134	C	G	0.686	0.995	PARD3
rs2250368	10	34,421,164	C	T	0.696	0.995	PARD3
rs1692708	10	34,421,554	G	A	0.696	0.995	PARD3
rs1765161	10	34,422,120	C	T	0.396	0.993	PARD3
rs1692709	10	34,422,121	A	G	0.396	0.993	PARD3
rs1753586	10	34,422,197	T	C	0.398	0.996	PARD3
rs1692710	10	34,422,678	T	C	0.397	0.997	PARD3
rs1328755	10	34,422,694	C	T	0.695	0.996	PARD3
rs1692712	10	34,423,253	A	C	0.397	0.997	PARD3
rs1854364	10	34,424,201	A	G	0.399	0.998	PARD3
rs1692694	10	34,424,703	A	G	0.399	0.998	PARD3
rs1692695	10	34,424,793	T	C	0.399	0.999	PARD3
rs1328754	10	34,425,037	G	A	0.399	0.998	PARD3
rs913546	10	34,425,264	C	G	0.399	0.998	PARD3
rs76773640	10	34,425,282	A	T	0.987	0.986	PARD3
rs913545	10	34,425,864	A	C	0.399	0.999	PARD3
rs118191576	10	34,425,991	C	T	0.974	0.94	PARD3
rs730715	10	34,426,019	C	T	0.99	0.994	PARD3
rs928036	10	34,426,280	A	G	0.688	0.999	PARD3
rs928035	10	34,426,410	C	T	0.697	0.999	PARD3
rs1571429	10	34,426,506	A	G	0.711	1	PARD3
rs1123652	10	34,426,639	T	C	0.399	0.999	PARD3
rs1123653	10	34,426,685	C	T	0.399	0.999	PARD3
rs1571428	10	34,426,912	T	C	0.697	1	PARD3
rs1753584	10	34,427,769	C	T	0.399	1	PARD3
rs1692699	10	34,427,800	A	G	0.399	1	PARD3
rs67056903	10	34,427,890	C	CAA	0.624	0.918	PARD3
rs1536771	10	34,428,832	G	A	0.688	0.999	PARD3
rs1778875	10	34,429,097	A	G	0.399	0.999	PARD3
rs928034	10	34,429,958	A	G	0.399	0.999	PARD3
rs2254394	10	34,430,669	G	C	0.688	0.997	PARD3
rs1753585	10	34,431,288	C	T	0.689	0.996	PARD3
rs1778873	10	34,431,337	C	G	0.698	0.996	PARD3
rs2254510	10	34,431,524	G	C	0.698	0.996	PARD3
rs11594830	10	34,432,002	C	A	0.712	0.996	PARD3
10:34432228_ACT_A	10	34,432,228	ACT	A	0.698	0.993	PARD3
rs2645230	10	34,432,471	C	T	0.698	0.995	PARD3
rs2645229	10	34,432,759	G	A	0.698	0.994	PARD3
rs10827332	10	34,433,123	T	G	0.71	0.993	PARD3
rs12768194	10	34,434,117	G	A	0.71	0.979	PARD3
rs1692705	10	34,434,206	A	T	0.672	0.988	PARD3
rs1441016	10	34,435,177	G	A	0.662	0.986	PARD3

SNP	Chromosome	Position	Effect Allele	Other Allele	Effect Allele Frequency	Imputation quality	Gene
rs1441017	10	34,435,450	C	A	0.662	0.986	PARD3
rs11596306	10	34,435,859	T	A	0.714	0.988	PARD3
10:34436023_TA_T	10	34,436,023	TA	T	0.377	0.97	PARD3
rs1874506	10	34,436,158	T	C	0.713	0.986	PARD3
rs533460593	10	34,436,360	C	CT	0.712	0.982	PARD3
rs650212	10	34,436,875	G	A	0.678	0.984	PARD3
rs649760	10	34,436,944	C	T	0.711	0.987	PARD3
rs2244099	10	34,437,623	C	T	0.389	0.983	PARD3
10:34437696_TTC_T	10	34,437,696	TTC	T	0.712	0.98	PARD3
rs33997458	10	34,437,792	G	GT	0.663	0.97	PARD3
rs2799453	10	34,437,906	G	A	0.381	0.982	PARD3
rs6481891	10	34,438,740	G	A	0.712	0.986	PARD3
rs11595985	10	34,439,522	G	C	0.712	0.986	PARD3
rs624906	10	34,439,809	T	A	0.68	0.982	PARD3
rs920515	10	34,439,852	C	A	0.382	0.981	PARD3
rs1866856	10	34,439,894	T	A	0.712	0.985	PARD3
rs12359835	10	34,440,176	T	C	0.712	0.984	PARD3
rs1148241	10	34,440,780	T	C	0.679	0.978	PARD3
rs6481892	10	34,440,939	T	C	0.712	0.983	PARD3
rs1148242	10	34,441,217	C	T	0.68	0.978	PARD3
rs1148243	10	34,441,297	G	T	0.68	0.978	PARD3
rs7909110	10	34,441,377	T	C	0.712	0.983	PARD3
10:34441704_CA_C	10	34,441,704	CA	C	0.705	0.972	PARD3
rs11009657	10	34,441,712	C	T	0.712	0.983	PARD3
rs7896314	10	34,441,782	G	A	0.703	0.983	PARD3
rs7896420	10	34,442,151	A	G	0.71	0.981	PARD3
rs673009	10	34,442,336	T	C	0.962	0.96	PARD3
rs686993	10	34,443,115	T	C	0.678	0.988	PARD3
rs12768988	10	34,443,123	C	T	0.955	0.992	PARD3
rs674388	10	34,443,331	G	C	0.683	0.99	PARD3
rs72784165	10	34,443,334	C	T	0.969	0.966	PARD3
rs71495070	10	34,443,468	A	C	0.955	0.997	PARD3
rs34994615	10	34,444,108	G	T	0.925	0.993	PARD3
rs1822126	10	34,444,434	C	A	0.925	0.993	PARD3
rs1805202	10	34,444,526	T	C	0.683	0.991	PARD3
rs1274307	10	34,444,551	C	T	0.415	0.99	PARD3
rs1805194	10	34,444,565	A	G	0.683	0.99	PARD3
rs1977894	10	34,444,709	A	G	0.683	0.989	PARD3
rs2247538	10	34,444,740	C	T	0.683	0.989	PARD3
rs1573218	10	34,444,775	T	C	0.683	0.99	PARD3
rs138056044	10	34,444,841	A	T	0.989	0.903	PARD3
rs146868717	10	34,444,995	T	TAC	0.925	0.991	PARD3
rs1036727	10	34,445,001	T	A	0.757	0.993	PARD3
rs1946931	10	34,445,419	T	C	0.97	0.987	PARD3
rs995459	10	34,445,870	A	C	0.458	0.994	PARD3
rs1036728	10	34,445,890	C	A	0.756	0.993	PARD3
rs1036729	10	34,445,905	G	A	0.726	0.993	PARD3
rs141153647	10	34,446,258	T	C	0.987	0.98	PARD3
rs34624059	10	34,446,312	T	C	0.956	0.998	PARD3
rs1274479	10	34,446,740	C	T	0.703	0.996	PARD3
rs12784304	10	34,446,857	G	A	0.956	0.998	PARD3
rs140172362	10	34,446,903	C	T	0.99	0.909	PARD3
rs1274480	10	34,447,071	T	C	0.658	0.996	PARD3
rs1274481	10	34,447,145	A	C	0.733	0.995	PARD3
10:34447150_AAAAC_A	10	34,447,150	AAAAC	A	0.956	0.998	PARD3
rs35491345	10	34,447,219	G	T	0.956	0.999	PARD3
rs1441018	10	34,447,596	G	A	0.97	0.99	PARD3
rs2247982	10	34,448,525	G	C	0.757	0.996	PARD3
rs148921528	10	34,448,591	T	C	0.974	0.946	PARD3
rs72784171	10	34,448,804	G	A	0.97	0.991	PARD3
rs1274482	10	34,448,807	T	C	0.415	0.997	PARD3
rs1274483	10	34,448,878	A	C	0.733	0.995	PARD3
rs75638445	10	34,449,264	C	T	0.956	0.998	PARD3
rs138016380	10	34,449,466	C	A	0.976	0.982	PARD3
rs139502930	10	34,449,831	A	G	0.987	0.985	PARD3
rs373658384	10	34,450,173	G	GAAA	0.014	0.705	PARD3
rs60424974	10	34,451,045	C	CA	0.959	0.85	PARD3
rs1660619	10	34,451,109	C	T	0.66	0.996	PARD3
rs2248375	10	34,452,072	C	A	0.757	0.997	PARD3
rs1660620	10	34,452,163	C	T	0.417	0.998	PARD3
rs1036730	10	34,452,352	A	T	0.733	0.997	PARD3
rs1660621	10	34,452,552	T	C	0.416	0.998	PARD3
rs16935154	10	34,452,653	T	G	0.971	0.988	PARD3
rs2786555	10	34,452,698	A	G	0.757	0.998	PARD3
rs148253339	10	34,453,004	A	G	0.989	0.897	PARD3
rs17459796	10	34,453,044	C	T	0.956	0.998	PARD3
rs2582847	10	34,453,271	G	A	0.733	0.997	PARD3
rs16935155	10	34,453,509	G	A	0.97	0.991	PARD3
rs139932618	10	34,453,725	C	CACACACAT	0.946	0.544	PARD3
rs2582848	10	34,453,743	T	C	0.659	0.998	PARD3
rs34579071	10	34,454,423	G	A	0.956	0.999	PARD3
rs75583195	10	34,455,293	C	A	0.983	0.944	PARD3
rs1619530	10	34,455,462	A	G	0.415	0.999	PARD3
rs1778876	10	34,455,466	G	A	0.734	0.998	PARD3
rs1765167	10	34,456,034	C	T	0.757	0.999	PARD3
rs1778877	10	34,456,102	T	C	0.757	0.999	PARD3
rs117777984	10	34,456,106	C	T	0.976	0.925	PARD3
rs1778878	10	34,456,232	G	A	0.757	0.999	PARD3
rs34928212	10	34,456,300	C	G	0.956	0.998	PARD3
rs1765166	10	34,456,317	G	T	0.757	0.999	PARD3
rs1778879	10	34,456,450	C	A	0.757	0.999	PARD3
rs1660622	10	34,456,509	C	G	0.733	0.999	PARD3
rs763279451	10	34,456,512	AAGGTAATATTT	A	0.758	0.995	PARD3
rs10508797	10	34,456,531	T	C	0.97	0.991	PARD3
rs147810894	10	34,456,553	G	GA	0.97	0.99	PARD3
rs1660623	10	34,456,606	G	A	0.757	0.999	PARD3
rs72784181	10	34,456,723	A	G	0.97	0.992	PARD3
10:34456979_GT_G	10	34,456,979	GT	G	0.461	0.999	PARD3
rs34881345	10	34,457,150	A	G	0.956	0.998	PARD3
rs641003	10	34,457,318	A	G	0.757	0.999	PARD3
rs588279	10	34,457,428	T	C	0.757	0.999	PARD3
rs1415545	10	34,457,963	A	C	0.757	0.999	PARD3
rs654887	10	34,458,078	C	T	0.757	0.998	PARD3
rs1274485	10	34,458,254	G	C	0.491	0.999	PARD3
rs894082	10	34,458,661	A	G	0.49	0.999	PARD3
rs748105	10	34,459,320	A	G	0.758	0.998	PARD3
rs748104	10	34,459,326	C	T	0.491	0.998	PARD3
rs2008617	10	34,459,338	A	T	0.97	0.992	PARD3
rs1274487	10	34,459,350	A	C	0.733	0.998	PARD3
rs748103	10	34,459,407	C	T	0.757	0.999	PARD3
rs1274488	10	34,460,411	G	A	0.733	0.998	PARD3
rs2009026	10	34,460,463	T	A	0.491	0.999	PARD3
rs2799454	10	34,460,469	C	T	0.758	1	PARD3
rs142196907	10	34,461,023	T	C	0.987	0.982	PARD3
rs2582850	10	34,461,058	A	C	0.733	0.998	PARD3
rs646442	10	34,461,235	C	T	0.758	1	PARD3
rs1822127	10	34,461,345	A	G	0.491	0.999	PARD3
rs647223	10	34,461,364	C	G	0.733	0.998	PARD3
rs1822128	10	34,461,421	T	A	0.733	0.998	PARD3
rs36014115	10	34,461,437	G	A	0.956	0.997	PARD3
rs576862810	10	34,461,679	A	AAC	0.735	0.997	PARD3
rs4285824	10	34,461,686	A	C	0.975	0.974	PARD3
10:34461699_AC_A	10	34,461,699	AC	A	0.735	0.998	PARD3
rs683777	10	34,461,812	G	A	0.735	0.998	PARD3
rs682376	10	34,462,127	C	T	0.758	0.999	PARD3
rs681954	10	34,462,207	G	A	0.758	0.999	PARD3
rs726196	10	34,462,730	C	T	0.757	0.999	PARD3
rs726197	10	34,462,934	T	A	0.447	0.998	PARD3
rs12784812	10	34,463,038	A	G	0.956	0.998	PARD3
rs1550516	10	34,463,336	A	G	0.734	0.998	PARD3
rs1972897	10	34,463,378	A	C	0.733	0.998	PARD3
rs12761772	10	34,463,579	A	C	0.956	0.998	PARD3
rs2255399	10	34,463,657	G	A	0.757	0.999	PARD3
rs1992378	10	34,463,769	A	G	0.733	0.998	PARD3
rs75920603	10	34,463,964	C	T	0.956	0.998	PARD3
rs75912813	10	34,464,104	T	G	0.956	0.998	PARD3
rs2570328	10	34,464,203	T	C	0.491	0.998	PARD3
rs117282992	10	34,464,591	A	G	0.953	0.911	PARD3
rs138986179	10	34,464,723	A	G	0.984	0.867	PARD3
rs613367	10	34,464,969	A	G	0.69	0.999	PARD3
rs1441019	10	34,465,105	G	A	0.448	0.998	PARD3
rs614299	10	34,465,205	C	T	0.759	0.994	PARD3
rs614302	10	34,465,207	G	C	0.759	0.994	PARD3
rs139227591	10	34,465,234	C	T	0.974	0.986	PARD3
10:34465265_CATT_C	10	34,465,265	CATT	C	0.956	0.998	PARD3
rs1441020	10	34,465,378	T	C	0.977	0.985	PARD3
rs612926	10	34,465,468	G	C	0.758	0.998	PARD3
rs626394	10	34,465,556	C	A	0.735	0.999	PARD3
rs140550182	10	34,465,738	T	C	0.985	0.896	PARD3
rs1290002	10	34,466,656	G	A	0.7	0.998	PARD3
rs16935190	10	34,466,715	C	T	0.977	0.986	PARD3
rs199742210	10	34,467,221	G	GC	0.957	0.99	PARD3
rs2799455	10	34,467,327	C	T	0.759	0.998	PARD3
rs2582851	10	34,468,096	G	A	0.739	0.892	PARD3
rs2582852	10	34,468,098	A	G	0.763	0.909	PARD3

SNP	Chromosome	Position	Effect Allele	Other Allele	Effect Allele Frequency	Imputation quality	Gene
rs369395258	10	34,468,100	A	G	0.922	0.507	PARD3
rs536521412	10	34,468,108	A	G	0.826	0.877	PARD3
rs71495071	10	34,469,124	C	T	0.955	0.998	PARD3
rs71495072	10	34,469,255	G	C	0.955	0.999	PARD3
rs71495073	10	34,469,265	G	A	0.955	0.999	PARD3
rs1295771	10	34,469,600	A	G	0.423	0.996	PARD3
rs2799456	10	34,469,712	G	T	0.759	0.997	PARD3
rs1274466	10	34,470,204	C	T	0.433	0.995	PARD3
rs1660627	10	34,470,304	T	C	0.733	0.995	PARD3
rs765366183	10	34,470,426	TG	T	0.757	0.993	PARD3
rs35046543	10	34,470,507	A	G	0.955	0.998	PARD3
rs1660628	10	34,470,754	C	T	0.733	0.995	PARD3
rs1274467	10	34,470,775	C	T	0.433	0.995	PARD3
rs17539342	10	34,471,188	G	C	0.955	0.998	PARD3
rs67361109	10	34,471,298	A	C	0.974	0.985	PARD3
rs752639	10	34,471,458	A	C	0.733	0.994	PARD3
rs827476	10	34,471,654	T	C	0.735	0.996	PARD3
rs827477	10	34,471,662	A	G	0.737	0.982	PARD3
rs377517675	10	34,471,670	T	TAA	0.77	0.733	PARD3
rs1296521	10	34,471,937	G	T	0.429	0.991	PARD3
rs638386	10	34,472,572	A	G	0.733	0.996	PARD3
rs2799457	10	34,472,990	T	C	0.478	0.992	PARD3
rs640308	10	34,473,016	G	A	0.742	0.994	PARD3
rs1660630	10	34,473,335	T	G	0.467	0.993	PARD3
rs642099	10	34,473,443	A	G	0.76	0.994	PARD3
rs72784193	10	34,473,624	C	T	0.957	0.786	PARD3
rs566371041	10	34,473,626	G	GT	0.333	0.716	PARD3
rs1837692	10	34,474,093	A	G	0.976	0.982	PARD3
rs1778882	10	34,474,154	C	T	0.434	0.992	PARD3
rs2799458	10	34,474,386	T	C	0.76	0.993	PARD3
rs754068007	10	34,474,739	CTATAT	C	0.615	0.864	PARD3
rs7902338	10	34,474,745	T	C	0.977	0.977	PARD3
rs1778883	10	34,474,802	T	C	0.733	0.991	PARD3
rs1660631	10	34,474,822	A	G	0.732	0.991	PARD3
rs1778884	10	34,474,849	A	T	0.76	0.993	PARD3
rs35017014	10	34,474,937	C	T	0.955	0.996	PARD3
rs1660632	10	34,475,080	T	C	0.76	0.992	PARD3
rs1765157	10	34,475,237	A	G	0.76	0.993	PARD3
rs2786551	10	34,475,809	A	G	0.501	0.99	PARD3
rs2786552	10	34,475,881	C	G	0.478	0.99	PARD3
10:34476279_CA_C	10	34,476,279	CA	C	0.955	0.996	PARD3
rs589369	10	34,476,483	G	C	0.76	0.992	PARD3
rs591681	10	34,477,044	T	C	0.74	0.988	PARD3
rs796446069	10	34,477,677	T	TA	0.762	0.979	PARD3
rs2786553	10	34,477,688	C	T	0.946	0.538	PARD3
rs1274470	10	34,477,824	T	C	0.716	0.986	PARD3
rs2799459	10	34,478,675	C	T	0.76	0.99	PARD3
rs12772584	10	34,478,676	A	G	0.955	0.996	PARD3
rs1274471	10	34,478,759	A	G	0.457	0.989	PARD3
rs2570329	10	34,478,770	G	A	0.76	0.99	PARD3
rs777628748	10	34,478,777	AC	A	0.762	0.971	PARD3
rs1274472	10	34,478,845	C	G	0.457	0.989	PARD3
rs1274473	10	34,478,957	C	T	0.457	0.989	PARD3
rs34699529	10	34,478,974	A	G	0.955	0.996	PARD3
rs71487350	10	34,479,003	C	G	0.955	0.996	PARD3
rs59467120	10	34,480,096	T	G	0.955	0.997	PARD3
rs1274475	10	34,480,582	G	A	0.607	0.97	PARD3
rs2246184	10	34,480,923	A	G	0.771	0.972	PARD3
rs34995359	10	34,481,138	G	C	0.93	0.995	PARD3
10:34481140_CAT_C	10	34,481,140	CAT	C	0.964	0.948	PARD3
rs1125879	10	34,482,906	G	C	0.728	0.989	PARD3
rs117594676	10	34,483,083	T	A	0.983	0.953	PARD3
rs34808555	10	34,483,954	G	T	0.955	0.996	PARD3
rs77350547	10	34,484,058	G	A	0.987	0.971	PARD3
rs1274476	10	34,484,338	C	T	0.728	0.989	PARD3
rs58340357	10	34,484,351	T	TAC	0.931	0.977	PARD3
rs7907524	10	34,485,455	C	A	0.954	0.994	PARD3
rs376471496	10	34,485,500	T	C	0.987	0.814	PARD3
rs530481747	10	34,485,503	T	C	0.987	0.823	PARD3
rs1274477	10	34,485,570	T	C	0.547	0.982	PARD3
rs566762023	10	34,485,692	A	G	0.986	0.955	PARD3
rs35922452	10	34,486,444	T	C	0.978	0.986	PARD3
rs1274478	10	34,487,002	G	A	0.63	0.988	PARD3
10:34487381_GGTGT_G	10	34,487,381	GGTGT	G	0.698	0.972	PARD3
rs661515	10	34,487,720	G	C	0.73	0.997	PARD3
rs629039	10	34,488,364	G	A	0.73	0.997	PARD3
rs2065949	10	34,488,892	G	A	0.954	1	PARD3
rs641881	10	34,488,940	G	A	0.73	0.998	PARD3
rs2053450	10	34,489,203	T	G	0.954	0.999	PARD3
rs2053449	10	34,489,204	T	A	0.954	0.999	PARD3
rs648768	10	34,490,601	G	A	0.332	0.996	PARD3
rs10827336	10	34,490,737	C	T	0.78	0.998	PARD3
rs1441026	10	34,490,829	A	G	0.955	0.999	PARD3
rs647758	10	34,490,854	A	G	0.331	0.996	PARD3
rs71487351	10	34,491,039	T	A	0.954	0.999	PARD3
rs75042057	10	34,491,578	T	G	0.977	0.945	PARD3
rs117298009	10	34,491,581	T	C	0.987	0.975	PARD3
rs10827337	10	34,491,850	G	A	0.779	0.998	PARD3
rs116942183	10	34,491,884	G	A	0.953	0.999	PARD3
rs71487352	10	34,492,021	C	T	0.954	0.999	PARD3
rs71487353	10	34,492,121	G	C	0.954	0.999	PARD3
rs71487354	10	34,492,439	A	G	0.954	0.999	PARD3
rs17461532	10	34,492,775	C	T	0.954	0.999	PARD3
rs34621461	10	34,493,043	T	C	0.954	0.999	PARD3
rs1660643	10	34,493,123	C	T	0.733	0.999	PARD3
rs75577284	10	34,493,274	A	G	0.954	0.999	PARD3
rs11009677	10	34,493,337	G	T	0.974	0.99	PARD3
rs201586310	10	34,493,623	T	TTACTGCTATCAGA	0.955	0.988	PARD3
rs386742861	10	34,493,629	T	TC	0.954	0.997	PARD3
rs11009678	10	34,494,171	C	A	0.778	0.999	PARD3
rs566726518	10	34,494,223	A	AT	0.777	0.998	PARD3
rs626385	10	34,494,467	G	A	0.631	0.986	PARD3
rs1837693	10	34,494,481	T	C	0.954	0.999	PARD3
rs17552880	10	34,494,749	C	G	0.887	0.99	PARD3
rs34276985	10	34,494,907	T	G	0.955	0.991	PARD3
10:34495081_AGTGGGAGGTCCCAGTGCAGGT_A	10	34,495,081	AGTGGGAGGTCCCAGTGCAGGT	A	0.956	0.973	PARD3
rs678188	10	34,495,289	A	G	0.597	0.996	PARD3
rs11598416	10	34,495,994	A	G	0.778	0.999	PARD3
rs662090	10	34,496,551	T	A	0.434	0.994	PARD3
rs192468662	10	34,496,760	A	G	0.989	0.817	PARD3
rs35879916	10	34,496,833	A	T	0.779	0.999	PARD3
rs649546	10	34,497,030	A	T	0.704	0.993	PARD3
rs71487355	10	34,497,367	A	C	0.954	0.998	PARD3
rs75617778	10	34,498,398	C	A	0.887	0.991	PARD3
rs1765169	10	34,498,716	A	C	0.331	0.998	PARD3
rs10827338	10	34,499,647	A	G	0.731	0.998	PARD3
rs75572339	10	34,500,210	G	A	0.936	0.984	PARD3
rs67709793	10	34,500,384	A	AG	0.954	0.996	PARD3
rs68010167	10	34,500,483	A	G	0.954	0.997	PARD3
rs186656621	10	34,500,662	C	T	0.975	0.963	PARD3
rs138555223	10	34,500,808	T	TC	0.954	0.996	PARD3
rs11597106	10	34,500,848	G	A	0.778	0.998	PARD3
10:34500949_CT_C	10	34,500,949	CT	C	0.513	0.881	PARD3
rs7070564	10	34,501,809	A	G	0.776	0.998	PARD3
rs36114686	10	34,501,988	T	C	0.955	0.996	PARD3
rs117479196	10	34,502,101	A	G	0.963	0.99	PARD3
rs12763757	10	34,502,333	T	C	0.955	0.997	PARD3
rs11009680	10	34,502,942	G	C	0.777	0.998	PARD3
rs117890443	10	34,503,039	G	C	0.982	0.929	PARD3
rs71523320	10	34,503,055	C	CT	0.776	0.995	PARD3
rs12769982	10	34,503,122	T	C	0.955	0.997	PARD3
rs1765160	10	34,503,393	T	C	0.6	0.998	PARD3
rs199897990	10	34,503,588	T	TA	0.955	0.988	PARD3
rs17553026	10	34,503,797	G	A	0.99	0.87	PARD3
rs1660642	10	34,504,022	G	A	0.332	0.999	PARD3
rs624056	10	34,504,787	A	G	0.6	0.998	PARD3
rs17553102	10	34,505,078	T	C	0.955	0.996	PARD3
rs35423999	10	34,505,601	T	C	0.955	0.996	PARD3
rs11009681	10	34,505,828	T	A	0.777	0.997	PARD3
rs12778087	10	34,505,864	T	C	0.776	0.996	PARD3
rs17461956	10	34,506,001	G	C	0.955	0.997	PARD3
rs1274464	10	34,506,032	C	G	0.33	0.999	PARD3
rs17553227	10	34,506,579	C	G	0.955	0.997	PARD3
rs1274463	10	34,507,556	G	A	0.437	0.992	PARD3
rs34548589	10	34,507,709	C	G	0.957	0.995	PARD3
rs11009684	10	34,508,148	C	T	0.976	0.995	PARD3
rs671228	10	34,509,032	G	A	0.438	0.99	PARD3
rs12774016	10	34,509,213	G	A	0.977	0.998	PARD3
rs17462068	10	34,509,215	G	A	0.957	0.994	PARD3
rs657517	10	34,509,833	G	C	0.681	0.988	PARD3
rs657477	10	34,509,861	T	A	0.681	0.988	PARD3
rs7083562	10	34,509,874	A	G	0.779	0.99	PARD3
rs367617125	10	34,510,316	CT	C	0.639	0.93	PARD3
rs12268666	10	34,510,647	G	C	0.957	0.993	PARD3
rs71487356	10	34,511,195	A	G	0.957	0.992	PARD3

SNP	Chromosome	Position	Effect Allele	Other Allele	Effect Allele Frequency	Imputation quality	Gene
rs17553380	10	34,511,795	A	G	0.957	0.994	PARD3
rs117634802	10	34,511,881	C	T	0.989	0.902	PARD3
rs34846718	10	34,511,897	C	T	0.957	0.991	PARD3
rs11009685	10	34,511,990	C	T	0.755	0.991	PARD3
rs76080125	10	34,512,759	A	G	0.938	0.976	PARD3
rs11595477	10	34,514,279	T	C	0.764	0.995	PARD3
rs2786548	10	34,514,418	C	G	0.75	0.996	PARD3
rs71487357	10	34,514,455	G	A	0.955	0.997	PARD3
rs2799461	10	34,514,516	A	C	0.715	0.995	PARD3
rs35333776	10	34,515,785	G	A	0.955	0.997	PARD3
rs55924133	10	34,515,822	A	G	0.763	0.995	PARD3
rs118073899	10	34,516,161	T	C	0.982	0.937	PARD3
rs11009686	10	34,516,354	A	G	0.955	0.998	PARD3
rs143712177	10	34,516,415	A	C	0.971	0.995	PARD3
rs12761982	10	34,516,420	G	A	0.955	0.998	PARD3
rs12766906	10	34,516,754	A	T	0.955	0.996	PARD3
rs55681930	10	34,516,784	TTA	T	0.35	0.878	PARD3
rs34027302	10	34,518,303	A	G	0.955	0.998	PARD3
rs11592463	10	34,518,442	G	A	0.804	0.995	PARD3
rs775796456	10	34,518,603	TG	T	0.805	0.993	PARD3
rs190361328	10	34,519,126	C	A	0.984	0.936	PARD3
rs61840216	10	34,519,537	T	G	0.764	0.995	PARD3
rs71487358	10	34,520,009	G	A	0.956	0.995	PARD3
rs1545214	10	34,520,359	C	A	0.746	0.993	PARD3
rs113860483	10	34,520,598	C	T	0.979	0.95	PARD3
rs72786230	10	34,520,606	C	T	0.803	0.996	PARD3
rs72786231	10	34,520,715	T	C	0.803	0.996	PARD3
rs71487359	10	34,520,959	G	A	0.956	0.999	PARD3
rs35937933	10	34,521,925	C	T	0.955	0.996	PARD3
rs150685125	10	34,522,018	T	TC	0.956	0.998	PARD3
rs12252951	10	34,522,353	C	A	0.956	0.998	PARD3
rs183559600	10	34,523,081	C	T	0.97	0.871	PARD3
rs536745942	10	34,523,082	G	A	0.971	0.868	PARD3
rs35958549	10	34,523,122	C	T	0.959	0.963	PARD3
rs34549471	10	34,523,145	G	C	0.971	0.869	PARD3
rs35833258	10	34,523,322	C	T	0.957	0.995	PARD3
rs77311278	10	34,523,396	C	T	0.924	0.991	PARD3
rs11595978	10	34,523,806	G	C	0.803	0.996	PARD3
rs17553567	10	34,524,711	T	G	0.763	0.999	PARD3
rs34667570	10	34,524,712	G	T	0.957	0.994	PARD3
rs2804540	10	34,525,456	T	C	0.95	0.966	PARD3
rs17462441	10	34,525,611	C	A	0.956	0.996	PARD3
rs1111267	10	34,527,387	C	A	0.76	0.95	PARD3
rs76061053	10	34,527,778	T	G	0.893	0.952	PARD3
rs12773126	10	34,527,997	G	A	0.887	0.989	PARD3
rs1631551	10	34,528,347	A	G	0.645	0.955	PARD3
rs1778868	10	34,528,727	T	G	0.574	0.906	PARD3
rs12762239	10	34,530,037	A	G	0.904	0.974	PARD3
rs617809	10	34,530,306	A	G	0.393	0.798	PARD3
rs139873816	10	34,530,883	T	TA	0.905	0.974	PARD3
rs61840218	10	34,530,913	C	A	0.903	0.986	PARD3
rs61840219	10	34,530,954	C	T	0.902	0.991	PARD3
rs61840220	10	34,531,338	G	T	0.904	0.981	PARD3
rs61840221	10	34,531,345	C	T	0.904	0.98	PARD3
rs61840222	10	34,532,548	G	A	0.903	0.991	PARD3
rs1660639	10	34,532,628	T	G	0.884	0.983	PARD3
rs1660640	10	34,532,629	C	T	0.884	0.982	PARD3
rs61840223	10	34,533,344	A	G	0.902	0.996	PARD3
rs17462570	10	34,533,466	A	G	0.902	0.997	PARD3
rs1765162	10	34,533,488	G	A	0.367	0.981	PARD3
rs34977483	10	34,534,000	C	CTG	0.886	0.99	PARD3
rs5784401	10	34,534,393	A	AG	0.51	0.971	PARD3
rs1778871	10	34,535,021	C	G	0.882	0.997	PARD3
rs1765163	10	34,535,064	T	C	0.887	0.997	PARD3
rs593992	10	34,536,049	T	C	0.983	0.994	PARD3
rs202178430	10	34,537,517	GT	G	0.366	0.989	PARD3
rs77955776	10	34,537,627	C	T	0.956	0.983	PARD3
rs2804542	10	34,537,706	A	C	0.883	0.999	PARD3
rs148069451	10	34,537,978	C	CT	0.95	0.957	PARD3
rs369539207	10	34,537,979	A	AAGT	0.952	0.954	PARD3
rs2804543	10	34,538,130	A	G	0.369	0.994	PARD3
rs2248686	10	34,539,038	C	T	0.984	0.995	PARD3
rs112539593	10	34,539,082	T	C	0.903	0.993	PARD3
rs368442480	10	34,539,083	C	G	0.903	0.995	PARD3
rs113990249	10	34,539,085	A	C	0.909	0.945	PARD3
rs112564361	10	34,539,087	A	C	0.908	0.94	PARD3
10:34539107_CCTAGAACT_C	10	34,539,107	CCTAGAACT	C	0.98	0.94	PARD3
rs2248691	10	34,539,229	C	A	0.984	0.998	PARD3
rs61840224	10	34,539,621	G	A	0.897	0.999	PARD3
rs199826566	10	34,539,699	TATA	T	0.485	0.988	PARD3
rs61840225	10	34,539,771	C	G	0.897	0.999	PARD3
rs2570322	10	34,540,166	C	T	0.948	0.961	PARD3
rs61840226	10	34,541,502	G	A	0.902	0.998	PARD3
rs2582853	10	34,541,654	C	T	0.984	0.997	PARD3
rs10631689	10	34,541,686	C	CTA	0.866	0.947	PARD3
rs2570321	10	34,541,856	T	C	0.984	0.996	PARD3
rs189234407	10	34,542,512	G	A	0.986	0.954	PARD3
rs746346147	10	34,542,611	ATCAGACGACCAC	A	0.482	0.989	PARD3
rs660154	10	34,543,013	C	T	0.504	0.98	PARD3
rs201147328	10	34,543,324	CA	C	0.479	0.97	PARD3
rs536934338	10	34,543,465	C	CGT	0.954	0.746	PARD3
rs1441025	10	34,543,897	A	G	0.976	0.99	PARD3
rs1348105	10	34,544,801	T	C	0.469	0.999	PARD3
rs1348106	10	34,544,880	T	C	0.469	0.999	PARD3
rs1095686	10	34,546,007	C	A	0.976	0.99	PARD3
rs1616608	10	34,546,576	T	A	0.976	0.991	PARD3
rs1660636	10	34,546,577	C	A	0.976	0.991	PARD3
rs143356107	10	34,547,078	A	G	0.967	0.934	PARD3
rs61840228	10	34,547,772	C	G	0.901	0.997	PARD3
rs626371	10	34,548,025	G	A	0.366	0.998	PARD3
rs626380	10	34,548,030	G	A	0.469	0.999	PARD3
rs626859	10	34,548,095	C	G	0.467	0.997	PARD3
rs626843	10	34,548,102	G	C	0.47	0.997	PARD3
rs626810	10	34,548,127	A	G	0.47	0.997	PARD3
10:34548246_CA_C	10	34,548,246	CA	C	0.366	0.997	PARD3
rs77254643	10	34,548,305	G	T	0.901	0.996	PARD3
rs77950484	10	34,548,542	T	C	0.901	0.996	PARD3
rs2582854	10	34,550,525	A	G	0.465	0.999	PARD3
rs7083805	10	34,550,629	G	A	0.653	0.791	PARD3
rs60452107	10	34,550,693	G	A	0.459	0.977	PARD3
rs200141318	10	34,550,709	A	G	0.973	0.684	PARD3
rs7911053	10	34,550,775	C	A	0.364	0.989	PARD3
rs4934509	10	34,550,779	C	A	0.465	0.998	PARD3
rs766134184	10	34,551,049	GGGTTTGTAGTCAA	G	0.473	0.989	PARD3
rs61840229	10	34,551,059	T	A	0.885	0.947	PARD3
rs72786249	10	34,551,125	G	T	0.925	0.959	PARD3
rs61840231	10	34,551,242	T	C	0.901	0.996	PARD3
rs72786250	10	34,551,243	G	A	0.925	0.959	PARD3
rs6481895	10	34,552,021	T	G	0.493	0.997	PARD3
rs7901148	10	34,552,061	T	G	0.49	0.997	PARD3
rs2891436	10	34,552,179	A	C	0.49	0.997	PARD3
10:34553675_ATT_A	10	34,553,675	ATT	A	0.375	0.962	PARD3
rs622894	10	34,553,828	T	C	0.365	0.997	PARD3
rs2256217	10	34,555,225	A	G	0.487	0.996	PARD3
rs10827342	10	34,555,237	G	A	0.671	0.989	PARD3
rs17578916	10	34,556,211	G	A	0.976	0.989	PARD3
rs72786255	10	34,556,503	T	C	0.99	0.951	PARD3
rs17554398	10	34,556,616	C	T	0.901	0.994	PARD3
rs2582855	10	34,556,943	G	A	0.487	0.995	PARD3
rs2786554	10	34,556,954	A	G	0.491	0.995	PARD3
10:34557076_TA_T	10	34,557,076	TA	T	0.387	0.993	PARD3
rs11009705	10	34,557,348	T	C	0.488	0.993	PARD3
rs34489524	10	34,557,407	C	CG	0.397	0.969	PARD3
rs1660637	10	34,557,761	T	C	0.492	0.992	PARD3
rs1660638	10	34,557,920	A	G	0.387	0.995	PARD3
rs41276094	10	34,558,839	G	A	0.98	0.946	PARD3
10:34559068_CT_C	10	34,559,068	CT	C	0.468	0.907	PARD3
rs668752	10	34,560,038	A	T	0.484	0.996	PARD3
rs1441015	10	34,560,284	G	A	0.487	0.996	PARD3
rs147033841	10	34,560,764	A	G	0.98	0.972	PARD3
rs1660616	10	34,560,810	C	T	0.976	0.988	PARD3
rs2083562	10	34,561,213	T	A	0.515	0.995	PARD3
rs148024707	10	34,561,290	T	C	0.979	0.958	PARD3
rs150569806	10	34,561,441	T	C	0.984	0.918	PARD3
rs2099791	10	34,561,480	A	T	0.515	0.995	PARD3
rs1582122	10	34,561,923	G	A	0.976	0.988	PARD3
rs61840241	10	34,562,213	A	G	0.901	0.993	PARD3
rs2570338	10	34,563,206	G	A	0.877	0.992	PARD3
rs675070	10	34,563,210	T	C	0.976	0.988	PARD3
rs674723	10	34,563,236	A	C	0.976	0.988	PARD3
rs661661	10	34,563,883	A	G	0.484	0.995	PARD3
rs658885	10	34,563,925	A	G	0.385	0.994	PARD3
rs10763976	10	34,564,292	G	A	0.512	0.995	PARD3
rs2570339	10	34,564,546	C	T	0.976	0.987	PARD3
10:34564670_TA_T	10	34,564,670	TA	T	0.966	0.92	PARD3

SNP	Chromosome	Position	Effect Allele	Other Allele	Effect Allele Frequency	Imputation quality	Gene
10:34565175_TA_T	10	34,565,175	TA	T	0.077	0.937	PARD3
rs1441014	10	34,565,865	C	A	0.976	0.988	PARD3
rs1660614	10	34,566,190	C	T	0.487	0.995	PARD3
rs618808	10	34,566,573	T	C	0.484	0.994	PARD3
rs148258217	10	34,566,682	G	A	0.984	0.908	PARD3
rs641162	10	34,566,920	G	A	0.385	0.993	PARD3
rs1074324	10	34,567,085	C	G	0.511	0.993	PARD3
rs5784405	10	34,567,143	T	TA	0.483	0.99	PARD3
rs1660613	10	34,567,543	T	C	0.42	0.965	PARD3
rs1660612	10	34,567,642	C	T	0.485	0.99	PARD3
rs1765174	10	34,568,032	C	T	0.387	0.991	PARD3
rs77560532	10	34,568,156	C	A	0.895	0.992	PARD3
rs139980375	10	34,568,390	C	T	0.984	0.972	PARD3
rs1660611	10	34,568,762	T	C	0.491	0.994	PARD3
rs10827343	10	34,569,584	C	T	0.893	0.992	PARD3
rs10827344	10	34,569,737	T	G	0.893	0.992	PARD3
rs16935275	10	34,570,702	C	T	0.894	0.993	PARD3
rs117940706	10	34,570,826	T	A	0.989	0.853	PARD3
rs2804545	10	34,571,051	C	T	0.385	0.995	PARD3
rs17554786	10	34,571,540	C	G	0.895	0.993	PARD3
rs75365462	10	34,571,712	A	T	0.945	0.964	PARD3
rs61840242	10	34,573,827	A	G	0.894	0.993	PARD3
rs17554828	10	34,573,881	G	T	0.982	0.939	PARD3
rs1778869	10	34,574,512	A	G	0.894	0.993	PARD3
rs1778870	10	34,575,279	C	T	0.972	0.988	PARD3
10:34575300_TAAC_T	10	34,575,300	TAAC	T	0.895	0.991	PARD3
rs11009713	10	34,575,735	G	C	0.89	0.986	PARD3
rs61840243	10	34,575,746	G	C	0.894	0.993	PARD3
rs653496	10	34,576,066	T	C	0.493	0.995	PARD3
rs584581	10	34,576,089	A	G	0.386	0.996	PARD3
rs582744	10	34,576,493	A	T	0.389	0.996	PARD3
rs118149964	10	34,576,728	C	T	0.989	0.898	PARD3
rs665070	10	34,577,547	T	C	0.385	0.996	PARD3
rs664538	10	34,577,697	T	C	0.386	0.996	PARD3
rs661896	10	34,578,297	C	T	0.389	0.996	PARD3
rs610493	10	34,578,733	T	C	0.893	0.993	PARD3
rs610066	10	34,578,832	T	A	0.389	0.996	PARD3
rs609970	10	34,578,899	G	A	0.389	0.996	PARD3
rs1660644	10	34,579,747	G	C	0.893	0.993	PARD3
rs581682	10	34,580,532	T	C	0.389	0.996	PARD3
rs1985313	10	34,580,880	G	T	0.68	0.99	PARD3
rs3891844	10	34,580,891	G	A	0.894	0.993	PARD3
rs10508800	10	34,580,993	T	A	0.976	0.987	PARD3
rs117556294	10	34,581,123	A	T	0.941	0.962	PARD3
rs10508801	10	34,581,444	G	A	0.895	0.994	PARD3
rs2799446	10	34,581,650	A	T	0.394	0.977	PARD3
rs2799447	10	34,581,653	T	A	0.959	0.874	PARD3
rs11009716	10	34,581,674	C	G	0.893	0.993	PARD3
10:34581988_CA_C	10	34,581,988	CA	C	0.973	0.981	PARD3
rs75898538	10	34,582,289	C	T	0.894	0.994	PARD3
rs2804547	10	34,582,371	T	C	0.389	0.996	PARD3
rs2582860	10	34,582,534	C	T	0.893	0.993	PARD3
10:34582550_CT_C	10	34,582,550	CT	C	0.898	0.973	PARD3
rs10694343	10	34,583,011	T	TAGG	0.389	0.994	PARD3
rs2197012	10	34,583,635	A	C	0.386	0.997	PARD3
rs1441027	10	34,583,697	G	A	0.496	0.996	PARD3
rs2570336	10	34,584,254	T	A	0.389	0.997	PARD3
rs17555548	10	34,584,558	G	A	0.977	0.988	PARD3
rs61840244	10	34,585,438	T	A	0.894	0.994	PARD3
rs61840245	10	34,585,453	C	T	0.894	0.994	PARD3
rs2799448	10	34,585,775	T	C	0.493	0.996	PARD3
rs61840246	10	34,585,855	A	G	0.894	0.994	PARD3
rs61840247	10	34,585,868	C	A	0.893	0.994	PARD3
rs111712922	10	34,585,946	C	T	0.974	0.968	PARD3
rs2007655	10	34,586,689	T	G	0.493	0.996	PARD3
rs2570335	10	34,586,957	A	G	0.496	0.996	PARD3
rs2582857	10	34,587,271	C	A	0.377	0.998	PARD3
rs2799449	10	34,587,579	T	C	0.971	0.987	PARD3
rs148131119	10	34,588,096	G	A	0.98	0.979	PARD3
rs17470791	10	34,588,370	C	T	0.895	0.995	PARD3
rs16935300	10	34,588,467	A	G	0.893	0.995	PARD3
rs2804548	10	34,588,538	G	C	0.484	0.997	PARD3
rs72788235	10	34,588,942	G	A	0.989	0.946	PARD3
rs2799451	10	34,589,212	T	C	0.377	0.999	PARD3
10:34589559_TA_T	10	34,589,559	TA	T	0.382	0.909	PARD3
rs3039313	10	34,589,942	A	AGGATT	0.895	0.988	PARD3
rs2053448	10	34,589,951	T	C	0.377	0.999	PARD3
rs2799452	10	34,590,350	G	A	0.487	0.997	PARD3
rs111358425	10	34,590,746	C	CT	0.619	0.721	PARD3
rs2165413	10	34,591,096	A	G	0.487	0.997	PARD3
rs2570334	10	34,591,402	C	T	0.38	0.999	PARD3
rs140109000	10	34,591,584	C	T	0.989	0.927	PARD3
rs757415684	10	34,591,995	TTTA	T	0.413	0.952	PARD3
rs775997551	10	34,592,090	ATTT	A	0.405	0.973	PARD3
rs78003027	10	34,592,369	T	C	0.895	0.995	PARD3
rs10647298	10	34,592,399	C	CAA	0.387	0.985	PARD3
rs2255021	10	34,592,543	A	G	0.487	0.996	PARD3
rs372510801	10	34,592,550	G	GT	0.888	0.966	PARD3
rs2255030	10	34,592,884	G	C	0.487	0.996	PARD3
rs1110654	10	34,593,202	T	A	0.487	0.996	PARD3
rs561084976	10	34,593,266	T	TA	0.867	0.919	PARD3
rs2570333	10	34,594,662	A	T	0.377	0.997	PARD3
rs1121830	10	34,594,733	G	C	0.381	0.994	PARD3
rs1121829	10	34,594,759	A	T	0.487	0.993	PARD3
rs2570332	10	34,595,070	G	A	0.483	0.995	PARD3
rs146015794	10	34,595,146	T	TTGC	0.511	0.992	PARD3
rs61840257	10	34,595,491	C	T	0.939	0.983	PARD3
rs79308303	10	34,595,703	T	C	0.893	0.994	PARD3
rs189010754	10	34,595,964	G	A	0.895	0.993	PARD3
10:34595981_AT_A	10	34,595,981	AT	A	0.415	0.982	PARD3
rs2570331	10	34,596,147	A	G	0.486	0.995	PARD3
rs11009724	10	34,596,332	T	C	0.893	0.994	PARD3
rs116509283	10	34,597,349	C	T	0.977	0.986	PARD3
rs182178265	10	34,598,169	G	A	0.983	0.919	PARD3
rs61840258	10	34,598,282	T	G	0.895	0.995	PARD3
rs12241064	10	34,599,342	A	G	0.97	0.984	PARD3
rs79132728	10	34,599,551	G	A	0.962	0.992	PARD3
rs1936429	10	34,599,986	T	C	0.511	0.997	PARD3
rs2384136	10	34,600,193	T	A	0.514	0.996	PARD3
rs11596342	10	34,600,466	C	G	0.962	0.991	PARD3
rs61840259	10	34,600,492	G	A	0.895	0.995	PARD3
rs61842460	10	34,600,657	T	C	0.895	0.995	PARD3
rs11009727	10	34,600,678	T	C	0.513	0.997	PARD3
rs11591230	10	34,602,101	T	C	0.962	0.991	PARD3
rs10763978	10	34,602,653	T	C	0.521	0.997	PARD3
rs10763979	10	34,602,764	A	G	0.625	0.995	PARD3
10:34603121_GA_G	10	34,603,121	GA	G	0.656	0.985	PARD3
10:34603142_CG_C	10	34,603,142	CG	C	0.979	0.981	PARD3
rs76591066	10	34,604,054	A	G	0.895	0.996	PARD3
rs11009730	10	34,604,895	T	G	0.982	0.997	PARD3
rs74512133	10	34,605,202	A	G	0.895	0.996	PARD3
rs370694797	10	34,605,819	T	TTTTA	0.986	0.972	PARD3
rs61842462	10	34,606,922	T	A	0.895	0.996	PARD3
rs3781127	10	34,607,015	G	A	0.521	0.997	PARD3
rs61842463	10	34,607,189	C	T	0.895	0.996	PARD3
rs11009732	10	34,607,733	G	A	0.703	0.993	PARD3
rs184933555	10	34,607,847	C	T	0.987	0.953	PARD3
rs4391769	10	34,607,931	G	A	0.521	0.997	PARD3
rs11009735	10	34,608,162	A	G	0.982	0.995	PARD3
rs10827347	10	34,610,372	C	G	0.511	0.997	PARD3
rs183102770	10	34,610,893	C	T	0.988	0.846	PARD3
rs2384137	10	34,612,485	A	G	0.982	0.995	PARD3
rs78974204	10	34,612,882	G	A	0.98	0.971	PARD3
rs74787580	10	34,614,381	T	C	0.947	0.992	PARD3
rs17471456	10	34,614,695	T	C	0.895	0.996	PARD3
rs6481896	10	34,614,776	T	C	0.059	0.952	PARD3
rs61842464	10	34,614,798	C	A	0.895	0.995	PARD3
rs140094790	10	34,614,847	A	C	0.982	0.995	PARD3
rs61842465	10	34,615,404	T	C	0.895	0.996	PARD3
rs17471491	10	34,616,043	T	C	0.895	0.996	PARD3
rs4379776	10	34,617,020	C	T	0.637	0.996	PARD3
rs61842466	10	34,618,640	G	C	0.895	0.996	PARD3
rs10740885	10	34,619,946	A	C	0.515	0.997	PARD3
rs11009737	10	34,620,334	T	C	0.982	0.994	PARD3
rs3781129	10	34,620,507	A	G	0.532	0.999	PARD3
rs35638866	10	34,620,681	C	CT	0.677	0.948	PARD3
rs778047582	10	34,620,693	G	GG	0.989	0.516	PARD3
rs7900322	10	34,620,826	A	C	0.973	0.991	PARD3
rs3781130	10	34,620,851	C	T	0.532	0.999	PARD3
rs142663955	10	34,621,003	T	A	0.987	0.957	PARD3
rs7917590	10	34,621,231	T	C	0.532	0.999	PARD3
rs149366318	10	34,621,674	T	C	0.98	0.973	PARD3
rs117145710	10	34,621,877	G	C	0.984	0.915	PARD3
rs60771200	10	34,622,015	T	C	0.982	0.992	PARD3
rs1961976	10	34,622,259	G	A	0.518	0.998	PARD3

SNP	Chromosome	Position	Effect Allele	Other Allele	Effect Allele Frequency	Imputation quality	Gene
rs1961977	10	34,622,507	C	T	0.518	0.998	PARD3
rs1961978	10	34,622,639	A	G	0.516	0.998	PARD3
rs61842467	10	34,622,679	C	T	0.895	0.996	PARD3
rs10827348	10	34,624,696	C	T	0.518	0.997	PARD3
10:34625896_CAA_C	10	34,625,896	CAA	C	0.895	0.994	PARD3
rs10508802	10	34,626,113	A	G	0.982	0.991	PARD3
rs78983471	10	34,626,841	C	T	0.988	0.784	PARD3
rs10827349	10	34,627,039	T	C	0.973	0.991	PARD3
rs7913268	10	34,627,091	A	C	0.512	0.996	PARD3
rs2384208	10	34,627,212	C	T	0.517	0.997	PARD3
rs17472038	10	34,627,407	G	C	0.895	0.995	PARD3
rs2384209	10	34,628,248	A	G	0.515	0.996	PARD3
rs2002773	10	34,628,649	G	A	0.535	0.996	PARD3
rs10508804	10	34,628,788	G	A	0.895	0.995	PARD3
rs16935335	10	34,628,807	T	C	0.982	0.918	PARD3
rs7922046	10	34,629,060	C	A	0.535	0.996	PARD3
rs11009741	10	34,629,657	C	T	0.982	0.99	PARD3
rs2384210	10	34,629,851	A	G	0.621	0.995	PARD3
10:34630111_GA_G	10	34,630,111	GA	G	0.489	0.733	PARD3
rs3781132	10	34,630,187	G	T	0.535	0.996	PARD3
rs17472227	10	34,630,247	T	G	0.895	0.995	PARD3
rs2384211	10	34,631,239	A	G	0.509	0.995	PARD3
rs2384212	10	34,631,466	A	C	0.509	0.994	PARD3
rs4402244	10	34,631,489	A	G	0.509	0.994	PARD3
rs113431401	10	34,632,648	C	T	0.98	0.969	PARD3
rs17472443	10	34,633,003	A	G	0.895	0.995	PARD3
10:34633208_GTT_G	10	34,633,208	GTT	G	0.563	0.963	PARD3
rs1138170	10	34,633,491	T	A	0.982	0.991	PARD3
rs2384213	10	34,633,874	C	T	0.514	0.995	PARD3
rs11009743	10	34,634,266	C	T	0.982	0.989	PARD3
rs61842492	10	34,634,494	C	T	0.895	0.995	PARD3
rs17472557	10	34,635,134	G	A	0.895	0.995	PARD3
rs12247156	10	34,635,529	C	T	0.856	0.994	PARD3
rs79114543	10	34,635,558	G	C	0.946	0.977	PARD3
rs4934629	10	34,635,579	G	A	0.519	0.995	PARD3
rs11009744	10	34,635,595	G	C	0.66	0.994	PARD3
rs117099312	10	34,636,290	C	G	0.987	0.903	PARD3
10:34636376_CA_C	10	34,636,376	CA	C	0.895	0.993	PARD3
rs7077783	10	34,636,820	C	T	0.856	0.995	PARD3
rs2384214	10	34,637,638	A	G	0.681	0.994	PARD3
10:34637928_GA_G	10	34,637,928	GA	G	0.515	0.996	PARD3
rs2384215	10	34,638,005	A	G	0.011	0.908	PARD3
rs74775116	10	34,638,379	A	T	0.894	0.996	PARD3
rs528679417	10	34,638,508	T	TAA	0.605	0.946	PARD3
rs11009747	10	34,638,548	C	T	0.918	0.982	PARD3
rs200155147	10	34,639,562	G	GA	0.966	0.884	PARD3
rs7477058	10	34,639,563	G	A	0.704	0.938	PARD3
rs10763981	10	34,640,064	T	C	0.519	0.997	PARD3
rs3842233	10	34,640,179	G	GT	0.704	0.994	PARD3
rs3781134	10	34,641,093	T	C	0.621	0.998	PARD3
rs3781135	10	34,641,109	T	C	0.522	0.998	PARD3
rs146147502	10	34,641,207	G	C	0.988	0.976	PARD3
rs140200662	10	34,641,327	G	A	0.901	0.997	PARD3
rs10827352	10	34,641,483	G	C	0.522	0.999	PARD3
rs4326737	10	34,641,636	G	C	0.522	0.997	PARD3
rs4367893	10	34,641,814	A	C	0.716	0.995	PARD3
rs146421924	10	34,641,842	G	A	0.901	0.997	PARD3
rs61842493	10	34,642,079	C	T	0.978	0.969	PARD3
rs11009748	10	34,642,089	G	A	0.943	0.994	PARD3
rs111357740	10	34,642,370	G	A	0.962	0.998	PARD3
10:34642644_TAAA_T	10	34,642,644	TAAA	T	0.898	0.986	PARD3
rs10827353	10	34,642,656	A	T	0.511	0.983	PARD3
rs375279575	10	34,643,058	C	CA	0.882	0.94	PARD3
rs11009749	10	34,643,137	C	T	0.905	0.996	PARD3
rs74633985	10	34,643,535	G	C	0.901	0.998	PARD3
rs76485429	10	34,644,237	C	T	0.901	0.998	PARD3
rs7096264	10	34,644,382	T	C	0.962	0.998	PARD3
rs116994883	10	34,644,474	C	T	0.985	0.983	PARD3
rs12765938	10	34,644,886	C	A	0.547	0.971	PARD3
rs7914727	10	34,644,978	C	T	0.957	0.976	PARD3
rs73256797	10	34,645,214	T	C	0.962	0.998	PARD3
rs74496737	10	34,645,501	T	C	0.985	0.967	PARD3
rs144561659	10	34,645,898	T	C	0.98	0.962	PARD3
rs10827354	10	34,646,523	C	T	0.522	0.999	PARD3
rs118168960	10	34,646,906	A	G	0.943	0.995	PARD3
rs12268500	10	34,646,967	T	C	0.905	0.996	PARD3
rs28548689	10	34,647,480	G	T	0.963	0.989	PARD3
rs10740887	10	34,647,489	C	T	0.522	0.999	PARD3
rs56393945	10	34,647,530	A	C	0.962	0.998	PARD3
rs76423462	10	34,647,867	T	C	0.901	0.998	PARD3
rs61735565	10	34,649,146	G	C	0.976	0.973	PARD3
rs117203475	10	34,650,101	C	G	0.943	0.996	PARD3
rs7099164	10	34,650,120	T	C	0.57	0.996	PARD3
rs10827355	10	34,650,318	G	A	0.519	1	PARD3
rs77697091	10	34,650,413	A	G	0.966	0.993	PARD3
rs143237762	10	34,650,760	T	TA	0.902	0.991	PARD3
rs147478243	10	34,651,201	T	C	0.943	0.996	PARD3
rs7895534	10	34,651,221	T	C	0.519	1	PARD3
rs7910834	10	34,651,559	G	A	0.519	1	PARD3
rs12765275	10	34,651,844	C	A	0.981	0.92	PARD3
rs61842496	10	34,651,852	C	T	0.924	0.972	PARD3
rs11412974	10	34,652,255	T	TC	0.519	0.998	PARD3
rs10827356	10	34,652,986	G	C	0.522	1	PARD3
rs142605683	10	34,653,361	T	TC	0.901	0.996	PARD3
rs763018138	10	34,653,362	C	CCT	0.947	0.785	PARD3
rs75397161	10	34,655,126	A	C	0.901	0.998	PARD3
rs10763983	10	34,655,300	G	T	0.52	0.999	PARD3
10:34655674_AAAATTTTTCTT_A	10	34,655,674	AAAATTTTTCTT	A	0.902	0.992	PARD3
rs12268268	10	34,655,709	C	A	0.905	0.997	PARD3
rs78167495	10	34,656,275	T	C	0.942	0.979	PARD3
rs112562705	10	34,657,327	G	A	0.901	0.998	PARD3
10:34657945_CT_C	10	34,657,945	CT	C	0.988	0.712	PARD3
rs142317551	10	34,657,955	T	TC	0.963	0.989	PARD3
rs12251119	10	34,658,231	T	G	0.943	0.993	PARD3
rs763480399	10	34,658,232	ATTTATTTATTTG	A	0.568	0.962	PARD3
rs7477823	10	34,658,298	G	C	0.714	0.998	PARD3
rs7078563	10	34,658,302	T	C	0.905	0.997	PARD3
rs17557767	10	34,658,479	C	T	0.982	0.956	PARD3
rs187160675	10	34,658,580	C	T	0.978	0.969	PARD3
rs17472956	10	34,658,652	T	C	0.901	0.998	PARD3
rs78796168	10	34,658,773	A	G	0.901	0.998	PARD3
rs10763984	10	34,658,847	C	T	0.522	0.999	PARD3
rs10763985	10	34,659,351	C	T	0.714	0.998	PARD3
rs11814015	10	34,660,461	C	A	0.497	0.953	PARD3
rs78361744	10	34,660,955	T	C	0.901	0.998	PARD3
rs10827357	10	34,661,015	A	G	0.714	0.998	PARD3
rs78899888	10	34,661,016	T	A	0.901	0.998	PARD3
rs10763986	10	34,661,027	C	A	0.522	0.999	PARD3
rs10763987	10	34,661,555	G	C	0.714	0.998	PARD3
10:34661637_ATGTG_A	10	34,661,637	ATGTG	A	0.969	0.968	PARD3
rs78260646	10	34,662,231	A	C	0.901	0.998	PARD3
rs17473033	10	34,662,334	C	T	0.979	0.974	PARD3
rs10763988	10	34,662,927	C	A	0.524	0.978	PARD3
rs10763989	10	34,662,928	A	G	0.52	0.997	PARD3
rs2384218	10	34,663,267	T	A	0.52	0.997	PARD3
rs2384219	10	34,665,197	G	A	0.53	0.998	PARD3
rs17557935	10	34,665,476	C	T	0.901	0.999	PARD3
rs10827360	10	34,665,566	T	C	0.531	0.998	PARD3
rs756499765	10	34,665,709	ATATATGAAAC	A	0.962	0.998	PARD3
rs11009757	10	34,665,779	T	C	0.724	0.999	PARD3
rs10827361	10	34,666,715	T	A	0.724	0.996	PARD3
rs10740890	10	34,666,864	C	T	0.533	0.996	PARD3
rs151103297	10	34,667,765	T	TA	0.966	0.925	PARD3
rs7071337	10	34,668,816	T	C	0.978	0.944	PARD3
rs10763990	10	34,669,404	G	T	0.54	0.995	PARD3
10:34670840_CAAAAAAAAAAAAAA_C	10	34,670,840	CAAAAAAAAAAAAAA	C	0.231	0.941	PARD3
rs7905901	10	34,671,259	T	G	0.962	0.998	PARD3
rs16935346	10	34,672,050	C	G	0.944	0.997	PARD3
10:34672250_AAGAC_A	10	34,672,250	AAGAC	A	0.987	0.982	PARD3
rs76895032	10	34,672,363	T	C	0.901	1	PARD3
rs16935349	10	34,672,463	T	C	0.905	0.998	PARD3
rs58978489	10	34,672,465	C	T	0.943	0.997	PARD3
10:34672612_GTGTATCTGAATACAACCCAATAT_G	10	34,672,612	GTGTATCTGAATACAACCCAATAT	G	0.907	0.972	PARD3
rs376266245	10	34,672,635	T	TAAAG	0.937	0.834	PARD3
rs141728806	10	34,673,303	G	C	0.979	0.981	PARD3
rs1987114	10	34,673,746	C	T	0.53	0.999	PARD3
rs144313494	10	34,674,262	C	G	0.944	0.997	PARD3
rs4534519	10	34,675,489	G	C	0.901	1	PARD3
rs2891453	10	34,675,516	T	C	0.53	0.999	PARD3
rs9629909	10	34,675,789	G	A	0.944	0.998	PARD3
10:34676731_GT_G	10	34,676,731	GT	G	0.53	0.999	PARD3
rs16935352	10	34,677,345	G	A	0.962	0.998	PARD3
rs2891454	10	34,677,405	A	C	0.724	1	PARD3
rs17558405	10	34,677,699	A	G	0.901	1	PARD3
10:34678128_CAT_C	10	34,678,128	CAT	C	0.906	0.997	PARD3

SNP	Chromosome	Position	Effect Allele	Other Allele	Effect Allele Frequency	Imputation quality	Gene
rs2384221	10	34,678,823	A	G	0.53	0.999	PARD3
rs2384222	10	34,678,900	A	T	0.53	0.999	PARD3
rs148615928	10	34,680,349	C	T	0.901	0.999	PARD3
rs11009769	10	34,680,493	C	T	0.724	0.999	PARD3
rs184156993	10	34,681,176	C	G	0.978	0.97	PARD3
10:34681209_GAA_G	10	34,681,209	GAA	G	0.901	0.999	PARD3
10:34682078_TA_T	10	34,682,078	TA	T	0.72	0.988	PARD3
rs7090627	10	34,682,141	A	C	0.962	0.999	PARD3
rs79330217	10	34,682,250	G	A	0.901	0.999	PARD3
rs11009770	10	34,682,402	G	C	0.724	0.999	PARD3
rs4098725	10	34,682,541	A	G	0.53	0.999	PARD3
rs4098726	10	34,682,640	A	G	0.53	0.999	PARD3
10:34682764_AT_A	10	34,682,764	AT	A	0.519	0.932	PARD3
rs2384223	10	34,682,828	C	T	0.987	0.963	PARD3
rs562949330	10	34,684,484	G	A	0.586	0.939	PARD3
rs531812590	10	34,684,485	G	T	0.586	0.939	PARD3
rs552021280	10	34,684,486	C	G	0.586	0.939	PARD3
rs11818878	10	34,685,202	C	T	0.982	0.94	PARD3
rs7893539	10	34,685,741	T	C	0.53	0.998	PARD3
rs10508805	10	34,685,865	T	C	0.53	0.998	PARD3
rs3844127	10	34,686,067	A	C	0.723	0.998	PARD3
rs10827362	10	34,687,914	G	C	0.534	0.997	PARD3
rs7921237	10	34,688,676	A	G	0.944	0.997	PARD3
rs7922198	10	34,689,412	A	T	0.53	0.997	PARD3
rs5784413	10	34,689,753	C	CT	0.864	0.987	PARD3
rs17473994	10	34,689,959	G	A	0.967	0.994	PARD3
rs79617950	10	34,691,092	A	G	0.984	0.96	PARD3
rs10827363	10	34,691,319	G	A	0.534	0.996	PARD3
rs17474100	10	34,691,405	T	C	0.901	0.995	PARD3
rs151007617	10	34,692,194	T	G	0.986	0.936	PARD3
rs4113505	10	34,692,235	T	C	0.962	0.998	PARD3
rs4113504	10	34,692,280	C	T	0.534	0.996	PARD3
rs117537606	10	34,692,895	A	G	0.984	0.955	PARD3
rs4934632	10	34,692,950	G	A	0.534	0.996	PARD3
rs4934633	10	34,693,172	G	A	0.906	0.998	PARD3
rs118106309	10	34,693,319	G	A	0.969	0.946	PARD3
rs114525256	10	34,693,865	T	C	0.944	0.998	PARD3
rs748440225	10	34,694,001	AACACAAACACACAC	A	0.541	0.992	PARD3
rs7912827	10	34,694,203	A	T	0.534	0.995	PARD3
rs118011751	10	34,694,396	C	T	0.954	0.993	PARD3
rs77546240	10	34,694,682	C	T	0.902	0.993	PARD3
rs7067991	10	34,694,739	G	A	0.534	0.995	PARD3
rs3905150	10	34,694,931	C	T	0.534	0.995	PARD3
rs7894351	10	34,695,508	C	T	0.534	0.995	PARD3
rs7894258	10	34,695,562	A	C	0.534	0.994	PARD3
rs369654163	10	34,695,728	G	GA	0.856	0.958	PARD3
10:34696773_GA_G	10	34,696,773	GA	G	0.531	0.992	PARD3
rs11814813	10	34,696,835	G	A	0.982	0.936	PARD3
rs12251009	10	34,697,141	G	A	0.531	0.991	PARD3
rs140376978	10	34,697,145	A	AC	0.691	0.924	PARD3
rs11009775	10	34,697,689	A	G	0.724	0.994	PARD3
10:34697775_TATA_T	10	34,697,775	TATA	T	0.901	0.991	PARD3
rs3851066	10	34,697,974	A	G	0.531	0.994	PARD3
rs112524636	10	34,698,062	C	A	0.983	0.953	PARD3
rs17474366	10	34,698,118	A	G	0.902	0.992	PARD3
rs16935380	10	34,698,465	C	T	0.727	0.994	PARD3
rs77424513	10	34,698,802	T	C	0.901	0.993	PARD3
rs111340254	10	34,699,239	G	A	0.975	0.903	PARD3
rs77891043	10	34,699,334	G	T	0.903	0.989	PARD3
rs17474408	10	34,699,476	A	C	0.902	0.991	PARD3
rs117236092	10	34,700,257	T	C	0.987	0.975	PARD3
rs2384216	10	34,700,357	C	A	0.728	0.992	PARD3
rs750237467	10	34,700,409	AAGGCTG	A	0.962	0.986	PARD3
10:34700847_CGTGTGTGTGT_C	10	34,700,847	CGTGTGTGTGT	C	0.541	0.98	PARD3
rs149990062	10	34,701,160	G	C	0.982	0.916	PARD3
rs12267294	10	34,701,568	T	C	0.943	0.99	PARD3
rs534912330	10	34,702,068	T	C	0.859	0.942	PARD3
rs12269019	10	34,702,070	T	C	0.82	0.952	PARD3
rs12269020	10	34,702,072	T	C	0.816	0.943	PARD3
rs774419337	10	34,702,074	C	T	0.983	0.927	PARD3
rs78487421	10	34,702,110	T	C	0.969	0.605	PARD3
rs66474287	10	34,702,146	T	C	0.98	0.926	PARD3
rs34880760	10	34,702,375	CT	C	0.399	0.94	PARD3
rs17565687	10	34,702,731	C	T	0.986	0.839	PARD3
rs12241731	10	34,703,076	T	C	0.945	0.955	PARD3
rs3851067	10	34,703,078	T	A	0.592	0.943	PARD3
rs79720677	10	34,703,119	A	C	0.942	0.981	PARD3
rs10827365	10	34,703,539	T	C	0.711	0.996	PARD3
10:34703756_TA_T	10	34,703,756	TA	T	0.71	0.995	PARD3
rs148122009	10	34,704,469	G	C	0.987	0.781	PARD3
rs2265784	10	34,706,027	A	C	0.662	0.992	PARD3
rs1418403	10	34,707,034	T	G	0.686	0.997	PARD3
rs1418404	10	34,707,228	A	G	0.679	0.997	PARD3
rs1657233	10	34,707,794	G	T	0.957	0.977	PARD3
rs77008281	10	34,708,474	G	T	0.892	0.954	PARD3
rs77798334	10	34,708,475	C	T	0.892	0.955	PARD3
rs7900667	10	34,708,881	A	G	0.981	0.974	PARD3
rs2253179	10	34,709,875	C	G	0.168	0.978	PARD3
rs1774749	10	34,710,086	C	T	0.687	0.997	PARD3
rs1657219	10	34,710,150	T	C	0.682	0.997	PARD3
rs773936	10	34,711,588	G	A	0.314	0.997	PARD3
rs551988460	10	34,713,436	T	TTTTA	0.989	0.672	PARD3
rs1342788	10	34,714,029	C	T	0.313	0.997	PARD3
rs117439994	10	34,714,238	G	A	0.974	0.98	PARD3
rs7079002	10	34,714,686	C	T	0.365	0.997	PARD3
rs10827366	10	34,715,017	A	T	0.318	0.997	PARD3
rs1774747	10	34,715,346	C	T	0.313	0.997	PARD3
rs1657226	10	34,715,349	G	A	0.313	0.988	PARD3
rs773948	10	34,716,034	A	T	0.312	0.996	PARD3
rs773949	10	34,716,323	T	G	0.364	0.997	PARD3
rs773950	10	34,716,794	G	C	0.365	0.997	PARD3
rs10827367	10	34,716,855	T	C	0.365	0.997	PARD3
rs773951	10	34,716,888	G	A	0.365	0.997	PARD3
rs1030840	10	34,717,415	A	G	0.949	0.993	PARD3
rs773952	10	34,717,559	C	A	0.313	0.998	PARD3
rs10827368	10	34,717,596	G	C	0.365	0.998	PARD3
rs708400	10	34,717,740	G	A	0.365	0.998	PARD3
rs773954	10	34,718,748	T	C	0.318	0.997	PARD3
rs77027317	10	34,719,283	C	T	0.953	0.993	PARD3
rs773955	10	34,719,446	G	A	0.365	0.998	PARD3
rs773956	10	34,719,906	A	G	0.365	0.998	PARD3
rs10827369	10	34,720,371	C	T	0.365	0.998	PARD3
rs112556799	10	34,720,713	C	CATGCCTGTA	0.376	0.986	PARD3
rs147204360	10	34,720,748	C	T	0.903	0.974	PARD3
rs192254015	10	34,720,749	G	A	0.961	0.951	PARD3
rs138698368	10	34,720,920	A	C	0.949	0.993	PARD3
rs773957	10	34,721,457	C	T	0.365	0.998	PARD3
rs773960	10	34,723,713	T	C	0.313	1	PARD3
10:34725508_TGTAAACATACAATTTCATTTTTA_T	10	34,725,508	TGTAAACATACAATTTCATTTTTA	T	0.956	0.957	PARD3
rs773961	10	34,725,544	T	C	0.318	0.999	PARD3
rs146072524	10	34,726,009	T	TAAAA	0.318	0.998	PARD3
rs773962	10	34,727,005	C	A	0.318	0.999	PARD3
rs773963	10	34,727,179	C	T	0.312	0.995	PARD3
rs10763992	10	34,727,397	A	G	0.365	0.998	PARD3
rs10763993	10	34,728,086	G	C	0.365	0.998	PARD3
rs756589506	10	34,728,671	AAC	A	0.319	0.995	PARD3
rs773964	10	34,731,264	C	A	0.311	0.994	PARD3
rs773966	10	34,731,554	C	T	0.313	0.993	PARD3
rs773967	10	34,731,595	G	C	0.317	0.994	PARD3
rs568731568	10	34,731,904	A	AT	0.948	0.958	PARD3
rs10827370	10	34,732,007	G	A	0.37	0.985	PARD3
rs2384225	10	34,732,349	C	T	0.64	0.959	PARD3
rs893071	10	34,732,605	C	T	0.702	0.98	PARD3
rs10657322	10	34,732,950	T	TCA	0.648	0.994	PARD3
rs10827371	10	34,733,516	C	T	0.704	0.999	PARD3
rs10827372	10	34,733,597	C	G	0.704	0.999	PARD3
rs773968	10	34,734,875	T	C	0.654	1	PARD3
rs148153637	10	34,735,106	A	G	0.982	0.95	PARD3
rs78315116	10	34,735,284	C	T	0.983	0.983	PARD3
rs17475284	10	34,735,753	T	C	0.687	0.991	PARD3
10:34735927_CCTCT_C	10	34,735,927	CCTCT	C	0.966	0.959	PARD3
rs7897519	10	34,736,280	G	A	0.704	1	PARD3
10:34736574_CA_C	10	34,736,574	CA	C	0.955	0.967	PARD3
rs142220985	10	34,737,062	C	CGTGT	0.709	0.988	PARD3
rs12250387	10	34,737,229	C	T	0.699	1	PARD3
rs536652096	10	34,737,255	G	GA	0.724	0.944	PARD3
rs34590811	10	34,737,391	C	T	0.699	1	PARD3
rs34337481	10	34,737,726	T	C	0.699	1	PARD3
rs34623887	10	34,737,836	G	A	0.704	1	PARD3
rs184836546	10	34,737,949	C	T	0.963	0.932	PARD3
rs563705051	10	34,738,012	A	AT	0.654	0.999	PARD3
rs185400281	10	34,738,086	C	T	0.952	0.955	PARD3
rs1774742	10	34,738,378	T	C	0.654	0.999	PARD3
rs5784417	10	34,738,526	T	TA	0.659	0.986	PARD3
10:34738577_TC_T	10	34,738,577	TC	T	0.699	0.999	PARD3

SNP	Chromosome	Position	Effect Allele	Other Allele	Effect Allele Frequency	Imputation quality	Gene
rs982687	10	34,739,166	T	C	0.936	0.978	PARD3
rs11009790	10	34,739,915	C	T	0.699	1	PARD3
rs532564315	10	34,740,701	G	GT	0.704	0.969	PARD3
rs118075889	10	34,740,732	G	A	0.989	0.898	PARD3
10:34740908_TATACTA_T	10	34,740,908	TATACTA	T	0.964	0.99	PARD3
rs10827373	10	34,741,030	G	A	0.704	1	PARD3
rs10827374	10	34,741,427	A	C	0.845	0.974	PARD3
rs708399	10	34,743,403	G	A	0.955	0.999	PARD3
rs1030839	10	34,743,420	T	A	0.704	1	PARD3
rs116888303	10	34,743,698	G	C	0.985	0.842	PARD3
rs1469874	10	34,743,703	C	T	0.699	0.999	PARD3
rs773939	10	34,744,143	T	A	0.955	0.998	PARD3
rs773940	10	34,745,107	A	C	0.955	0.998	PARD3
rs773941	10	34,745,186	A	G	0.955	0.998	PARD3
rs1966689	10	34,745,523	G	A	0.704	0.999	PARD3
rs78853671	10	34,745,624	A	G	0.982	0.96	PARD3
rs7903841	10	34,746,642	T	C	0.699	0.999	PARD3
rs12784987	10	34,748,180	C	T	0.704	0.999	PARD3
rs34208719	10	34,749,483	C	T	0.704	0.999	PARD3
rs142593037	10	34,749,563	C	A	0.944	0.948	PARD3
rs78693215	10	34,750,128	C	A	0.962	0.977	PARD3
rs11009795	10	34,750,596	C	T	0.704	0.999	PARD3
rs773943	10	34,750,845	C	A	0.659	0.999	PARD3
rs773944	10	34,750,875	A	G	0.659	0.999	PARD3
rs1774741	10	34,752,788	T	C	0.654	0.999	PARD3
rs375290950	10	34,753,047	GA	G	0.647	0.974	PARD3
rs1616895	10	34,753,430	G	T	0.654	0.999	PARD3
rs1774740	10	34,754,754	G	A	0.955	0.998	PARD3
rs116872693	10	34,754,773	T	C	0.971	0.967	PARD3
rs810150	10	34,756,493	C	T	0.653	0.999	PARD3
rs12785126	10	34,756,716	C	T	0.965	0.993	PARD3
rs113382914	10	34,760,509	G	A	0.983	0.94	PARD3
rs773994	10	34,761,186	T	A	0.955	0.998	PARD3
rs72794312	10	34,762,465	A	T	0.705	0.993	PARD3
rs56390521	10	34,762,566	C	A	0.964	0.974	PARD3
rs139435359	10	34,762,686	C	T	0.984	0.932	PARD3
rs773995	10	34,762,908	T	C	0.955	0.998	PARD3
rs773996	10	34,764,768	T	C	0.652	0.999	PARD3
rs1657231	10	34,765,364	C	G	0.102	0.983	PARD3
rs140119729	10	34,767,080	T	C	0.981	0.947	PARD3
rs773997	10	34,767,507	C	A	0.654	0.999	PARD3
10:34768088_AT_A	10	34,768,088	AT	A	0.861	0.864	PARD3
rs773998	10	34,768,332	G	A	0.955	0.998	PARD3
rs773999	10	34,768,387	A	G	0.654	0.999	PARD3
rs774000	10	34,769,796	A	C	0.652	0.998	PARD3
10:34770124_TTTTA_T	10	34,770,124	TTTTA	T	0.652	0.997	PARD3
rs774001	10	34,770,578	T	C	0.652	0.999	PARD3
rs774002	10	34,770,855	C	G	0.652	0.999	PARD3
rs531739004	10	34,771,922	C	CA	0.987	0.799	PARD3
rs7100738	10	34,772,426	A	G	0.683	0.952	PARD3
rs78470473	10	34,772,484	T	C	0.979	0.968	PARD3
rs2804722	10	34,772,964	T	G	0.652	0.998	PARD3
10:34774265_CA_C	10	34,774,265	CA	C	0.653	0.988	PARD3
rs11009804	10	34,774,296	G	A	0.703	0.999	PARD3
rs138259586	10	34,774,878	G	T	0.983	0.994	PARD3
rs773980	10	34,774,914	C	T	0.658	0.998	PARD3
rs2804721	10	34,775,745	T	C	0.896	0.99	PARD3
rs144572925	10	34,776,221	T	C	0.98	0.965	PARD3
rs773981	10	34,776,350	C	T	0.657	0.998	PARD3
rs6481899	10	34,777,050	C	T	0.976	0.915	PARD3
rs773982	10	34,777,901	C	T	0.658	0.998	PARD3
rs148830765	10	34,778,330	G	A	0.987	0.959	PARD3
rs773983	10	34,779,131	C	T	0.955	0.997	PARD3
rs773984	10	34,782,519	C	G	0.651	0.998	PARD3
rs773985	10	34,782,752	C	T	0.652	0.998	PARD3
rs773986	10	34,782,921	A	G	0.657	0.998	PARD3
rs773987	10	34,784,023	G	A	0.657	0.998	PARD3
rs74428073	10	34,784,111	C	A	0.937	0.964	PARD3
rs146038123	10	34,785,061	C	G	0.981	0.967	PARD3
rs773988	10	34,785,547	C	T	0.656	0.998	PARD3
rs773989	10	34,785,685	A	C	0.656	0.998	PARD3
10:34785817_TAAA_T	10	34,785,817	TAAA	T	0.833	0.783	PARD3
rs60009061	10	34,785,818	A	C	0.651	0.996	PARD3
rs760566164	10	34,785,819	AAAATAGTATAC	A	0.715	0.875	PARD3
rs561109882	10	34,785,821	A	C	0.833	0.783	PARD3
10:34785823_TA_T	10	34,785,823	TA	T	0.833	0.783	PARD3
10:34785825_GTATACTA_G	10	34,785,825	GTATACTA	G	0.833	0.783	PARD3
rs61841109	10	34,786,384	G	A	0.963	0.944	PARD3
rs4934636	10	34,786,691	G	A	0.702	0.998	PARD3
rs773990	10	34,786,887	C	T	0.955	0.997	PARD3
rs773991	10	34,787,091	T	C	0.651	0.998	PARD3
rs16935461	10	34,787,367	T	C	0.702	0.998	PARD3
rs147101194	10	34,787,679	T	C	0.983	0.993	PARD3
rs138844885	10	34,791,474	T	C	0.98	0.966	PARD3
rs140849962	10	34,791,984	T	TA	0.955	0.991	PARD3
rs4934637	10	34,792,494	A	T	0.701	0.998	PARD3
rs72794328	10	34,793,006	T	C	0.98	0.954	PARD3
rs550527808	10	34,793,357	T	TA	0.716	0.955	PARD3
rs773973	10	34,794,018	A	G	0.651	0.998	PARD3
rs111457248	10	34,794,541	A	T	0.952	0.973	PARD3
rs773975	10	34,795,349	C	G	0.651	0.998	PARD3
rs773976	10	34,795,396	G	A	0.651	0.998	PARD3
rs73265393	10	34,796,476	C	A	0.754	0.794	PARD3
rs549652757	10	34,796,478	A	AAAAAAAAAAAC	0.699	0.941	PARD3
rs773977	10	34,797,205	A	G	0.651	0.998	PARD3
rs7477871	10	34,797,585	T	C	0.925	0.995	PARD3
rs201804245	10	34,798,729	C	CA	0.954	0.964	PARD3
rs773979	10	34,798,825	A	T	0.651	0.998	PARD3
rs753879105	10	34,800,584	AC	A	0.769	0.986	PARD3
rs76148659	10	34,801,326	G	A	0.953	0.989	PARD3
rs759406108	10	34,802,563	CAT	C	0.772	0.997	PARD3
rs7100998	10	34,802,635	C	T	0.925	0.995	PARD3
rs141694902	10	34,802,702	G	A	0.985	0.965	PARD3
rs1025637	10	34,803,585	C	T	0.771	0.999	PARD3
rs1025638	10	34,803,778	T	C	0.77	0.999	PARD3
rs560624649	10	34,804,900	C	CT	0.691	0.956	PARD3
rs10827377	10	34,805,261	T	A	0.687	0.986	PARD3
rs1780578	10	34,806,744	G	A	0.95	0.994	PARD3
rs7083117	10	34,807,134	T	A	0.645	0.983	PARD3
rs142908159	10	34,807,295	T	C	0.98	0.963	PARD3
rs76790356	10	34,808,513	G	A	0.911	0.989	PARD3
rs60321673	10	34,808,837	TA	T	0.693	0.992	PARD3
rs77318120	10	34,809,394	C	T	0.989	0.964	PARD3
rs1342339	10	34,811,236	T	C	0.643	0.996	PARD3
rs16935478	10	34,811,929	G	A	0.956	0.997	PARD3
rs145488845	10	34,812,385	T	C	0.98	0.976	PARD3
rs773969	10	34,813,600	C	T	0.955	0.999	PARD3
rs773970	10	34,813,742	G	A	0.643	0.996	PARD3
rs708401	10	34,813,908	A	C	0.955	0.999	PARD3
rs773971	10	34,814,167	T	C	0.643	0.997	PARD3
rs773972	10	34,814,624	C	T	0.643	0.997	PARD3
rs1436732	10	34,815,716	A	G	0.95	0.998	PARD3
rs1631489	10	34,815,915	C	T	0.643	0.997	PARD3
rs1657220	10	34,816,233	C	T	0.95	0.998	PARD3
rs7086954	10	34,816,758	A	C	0.643	0.997	PARD3
rs138359925	10	34,816,830	A	T	0.982	0.958	PARD3
rs10827378	10	34,817,000	A	T	0.693	0.997	PARD3
rs773945	10	34,817,343	C	T	0.95	0.999	PARD3
rs767034544	10	34,817,481	G	GTTTTGT	0.986	0.944	PARD3
rs34318724	10	34,818,669	C	CT	0.695	0.988	PARD3
rs118164313	10	34,820,104	A	T	0.971	0.99	PARD3
rs117216987	10	34,820,111	G	C	0.982	0.919	PARD3
rs773946	10	34,821,063	G	T	0.605	0.999	PARD3
rs773050204	10	34,821,446	CTGTGTGAAG	C	0.98	0.992	PARD3
rs10827380	10	34,822,258	C	T	0.686	0.998	PARD3
rs527855	10	34,822,980	G	A	0.61	0.999	PARD3
rs593034	10	34,823,300	C	T	0.605	0.999	PARD3
rs607870	10	34,824,273	A	C	0.61	0.999	PARD3
rs563556401	10	34,824,743	T	TA	0.62	0.968	PARD3
rs622285	10	34,825,187	T	C	0.605	0.999	PARD3
rs505537	10	34,825,669	C	T	0.61	0.999	PARD3
rs638053	10	34,826,420	A	C	0.605	0.999	PARD3
rs79553866	10	34,826,511	C	A	0.951	0.995	PARD3
rs665402	10	34,827,921	A	C	0.924	0.995	PARD3
rs667159	10	34,828,344	T	C	0.605	0.999	PARD3
rs484833	10	34,828,564	C	T	0.61	0.999	PARD3
rs80254764	10	34,828,765	C	G	0.964	0.965	PARD3
10:34829258_TA_T	10	34,829,258	TA	T	0.847	0.687	PARD3
rs187387206	10	34,829,413	T	C	0.981	0.975	PARD3
rs143582528	10	34,831,162	T	TGTGTGCAC	0.579	0.883	PARD3
rs1780575	10	34,831,429	T	G	0.605	0.999	PARD3
rs117965800	10	34,831,578	T	C	0.981	0.975	PARD3
rs493392	10	34,831,887	G	C	0.605	0.999	PARD3
rs35082383	10	34,832,028	C	CT	0.614	0.989	PARD3
rs1529940	10	34,832,599	A	T	0.61	0.998	PARD3

SNP	Chromosome	Position	Effect Allele	Other Allele	Effect Allele Frequency	Imputation quality	Gene
rs1624134	10	34,834,482	G	C	0.605	0.999	PARD3
rs77120493	10	34,834,982	T	C	0.979	0.961	PARD3
rs2804720	10	34,835,133	G	T	0.61	0.999	PARD3
10:34835444_CA_C	10	34,835,444	CA	C	0.598	0.99	PARD3
10:34835464_TTTG_T	10	34,835,464	TTTG	T	0.606	0.996	PARD3
rs1657222	10	34,837,139	G	A	0.604	0.994	PARD3
rs1657223	10	34,837,272	C	A	0.846	0.953	PARD3
rs115278153	10	34,837,274	A	C	0.942	0.974	PARD3
rs762325419	10	34,837,490	GA	G	0.605	0.998	PARD3
rs1657224	10	34,837,629	A	T	0.605	0.999	PARD3
rs36042302	10	34,838,937	C	T	0.964	0.97	PARD3
rs2762529	10	34,841,293	A	G	0.087	0.996	PARD3
10:34841642_CA_C	10	34,841,642	CA	C	0.601	0.988	PARD3
rs610601	10	34,841,918	A	G	0.61	0.999	PARD3
rs623335	10	34,842,473	T	C	0.61	0.999	PARD3
rs10508807	10	34,842,837	G	A	0.924	0.998	PARD3
rs1780576	10	34,843,326	C	T	0.985	0.982	PARD3
rs639291	10	34,843,790	C	T	0.605	0.998	PARD3
rs77334559	10	34,843,847	T	C	0.956	0.981	PARD3
10:34845313_CT_C	10	34,845,313	CT	C	0.678	0.979	PARD3
rs115242789	10	34,846,878	A	G	0.986	0.864	PARD3
rs4934511	10	34,847,064	T	C	0.616	0.999	PARD3
rs6481901	10	34,847,895	G	A	0.611	0.997	PARD3
10:34847965_AT_A	10	34,847,965	AT	A	0.981	0.925	PARD3
rs7068275	10	34,847,978	A	C	0.625	0.988	PARD3
rs372953621	10	34,848,379	GAA	G	0.616	0.998	PARD3
10:34848391_AAG_A	10	34,848,391	AAG	A	0.782	0.724	PARD3
rs397793572	10	34,848,392	AG	A	0.925	0.605	PARD3
rs11599915	10	34,848,612	T	C	0.97	0.992	PARD3
rs17490612	10	34,848,750	G	A	0.982	0.964	PARD3
rs10827381	10	34,850,203	A	G	0.611	0.998	PARD3
rs11009814	10	34,850,550	A	G	0.611	0.998	PARD3
rs111317024	10	34,850,612	G	A	0.956	0.938	PARD3
rs116612508	10	34,850,991	G	C	0.948	0.921	PARD3
rs2384227	10	34,851,111	T	C	0.612	0.998	PARD3
rs7071602	10	34,851,940	T	C	0.611	0.998	PARD3
rs2384228	10	34,852,419	C	A	0.616	0.998	PARD3
10:34852460_AT_A	10	34,852,460	AT	A	0.592	0.963	PARD3
rs149574452	10	34,852,496	T	C	0.93	0.982	PARD3
rs1324304	10	34,853,690	C	T	0.611	0.998	PARD3
rs1324305	10	34,853,746	T	C	0.611	0.998	PARD3
rs117164009	10	34,854,000	T	C	0.955	0.993	PARD3
rs1540953	10	34,855,095	A	G	0.616	0.999	PARD3
rs11009816	10	34,857,131	T	G	0.693	0.998	PARD3
rs148492721	10	34,857,289	G	A	0.98	0.994	PARD3
rs7899568	10	34,857,723	T	C	0.611	0.999	PARD3
rs56777924	10	34,858,591	C	T	0.616	0.998	PARD3
rs35199552	10	34,858,824	C	CA	0.494	0.904	PARD3
rs76043726	10	34,858,950	T	C	0.957	0.981	PARD3
rs11009819	10	34,858,994	T	C	0.617	0.999	PARD3
rs144160162	10	34,861,423	C	G	0.971	0.983	PARD3
rs11009820	10	34,862,304	C	A	0.612	0.999	PARD3
rs11009821	10	34,862,400	T	C	0.612	0.999	PARD3
10:34865300_TA_T	10	34,865,300	TA	T	0.979	0.975	PARD3
rs1408380	10	34,865,694	A	G	0.636	1	PARD3
rs1408379	10	34,865,799	G	A	0.631	1	PARD3
rs2025459	10	34,866,074	A	C	0.631	1	PARD3
rs546562935	10	34,866,978	C	CT	0.637	0.996	PARD3
rs10827382	10	34,867,925	G	A	0.635	0.999	PARD3
rs555542103	10	34,868,196	C	CA	0.642	0.973	PARD3
rs6481903	10	34,869,789	T	C	0.631	1	PARD3
rs4525173	10	34,870,394	C	T	0.636	1	PARD3
rs1324303	10	34,871,172	T	A	0.636	1	PARD3
rs1324302	10	34,871,258	T	C	0.636	1	PARD3
rs10827383	10	34,871,907	C	A	0.636	1	PARD3
rs78429736	10	34,872,427	C	T	0.924	0.997	PARD3
rs767612796	10	34,874,475	C	CT	0.639	0.991	PARD3
rs768579895	10	34,874,487	G	GG	0.979	0.562	PARD3
10:34876058_TTTTG_T	10	34,876,058	TTTTG	T	0.982	0.965	PARD3
rs1359281	10	34,876,830	C	T	0.631	1	PARD3
rs1359280	10	34,877,263	C	T	0.636	1	PARD3
rs80254271	10	34,878,351	A	G	0.887	0.995	PARD3
rs768699140	10	34,881,026	CG	C	0.924	1	PARD3
rs117586216	10	34,882,958	T	C	0.955	0.997	PARD3
rs140420439	10	34,883,430	T	A	0.986	0.956	PARD3
rs7916657	10	34,883,620	A	G	0.631	1	PARD3
rs546382471	10	34,885,164	C	CT	0.634	0.975	PARD3
rs142691148	10	34,886,202	C	T	0.983	0.915	PARD3
rs1540952	10	34,886,357	C	T	0.631	1	PARD3
rs10827384	10	34,887,003	C	T	0.713	1	PARD3
rs11009830	10	34,887,423	T	G	0.631	1	PARD3
rs112481056	10	34,889,108	C	CA	0.683	0.927	PARD3
10:34890196_GA_G	10	34,890,196	GA	G	0.627	0.986	PARD3
rs2384230	10	34,890,638	C	A	0.631	0.994	PARD3
rs2384231	10	34,890,639	G	C	0.631	0.994	PARD3
rs11595665	10	34,890,830	T	C	0.914	0.966	PARD3
rs9888021	10	34,892,174	C	T	0.631	1	PARD3
rs10827385	10	34,892,328	C	T	0.636	1	PARD3
rs2477012	10	34,892,550	T	C	0.631	1	PARD3
rs116962555	10	34,893,215	A	G	0.979	0.957	PARD3
rs2477013	10	34,893,226	A	G	0.631	1	PARD3
rs2496719	10	34,894,973	T	C	0.631	1	PARD3
rs2496720	10	34,895,329	T	C	0.887	0.959	PARD3
rs9418104	10	34,895,469	T	C	0.622	0.995	PARD3
rs531578595	10	34,895,893	T	TA	0.915	0.898	PARD3
rs117729216	10	34,896,206	C	A	0.982	0.996	PARD3
rs2496722	10	34,896,432	A	T	0.636	1	PARD3
rs2490804	10	34,896,745	T	C	0.636	1	PARD3
rs533339742	10	34,897,462	C	CT	0.781	0.912	PARD3
rs7098206	10	34,898,549	T	C	0.712	1	PARD3
rs2244475	10	34,898,795	A	C	0.985	0.981	PARD3
rs571031262	10	34,900,858	G	GC	0.636	1	PARD3
rs2496743	10	34,901,849	C	T	0.631	1	PARD3
rs2477015	10	34,902,333	T	C	0.631	1	PARD3
rs2489663	10	34,902,822	C	T	0.632	1	PARD3
rs2496714	10	34,903,536	G	C	0.632	1	PARD3
rs146268546	10	34,903,723	T	C	0.984	0.904	PARD3
rs533452319	10	34,904,329	T	TAACC	0.979	0.925	PARD3
rs2477016	10	34,904,494	G	A	0.632	1	PARD3
rs2490805	10	34,904,731	A	G	0.637	1	PARD3
rs2496715	10	34,906,191	T	A	0.632	1	PARD3
10:34906791_TA_T	10	34,906,791	TA	T	0.982	0.983	PARD3
rs28700890	10	34,907,984	T	C	0.99	0.748	PARD3
rs78889453	10	34,911,303	T	A	0.977	0.928	PARD3
rs2496716	10	34,911,436	C	T	0.636	1	PARD3
rs2489655	10	34,911,607	A	G	0.631	1	PARD3
rs2477017	10	34,911,879	G	A	0.632	0.999	PARD3
rs117460190	10	34,912,314	G	A	0.987	0.934	PARD3
rs2496718	10	34,913,376	A	G	0.632	1	PARD3
rs117992462	10	34,913,743	G	C	0.982	0.995	PARD3
rs2489654	10	34,914,875	C	T	0.637	1	PARD3
rs748769	10	34,916,731	T	A	0.632	1	PARD3
rs117076631	10	34,918,165	A	G	0.981	0.933	PARD3
rs2490806	10	34,918,550	C	T	0.631	1	PARD3
rs2490807	10	34,918,935	C	T	0.631	1	PARD3
rs2489662	10	34,922,118	C	T	0.637	1	PARD3
rs7076806	10	34,922,430	G	A	0.965	0.994	PARD3
rs2490808	10	34,922,751	C	A	0.924	0.999	PARD3
rs12782862	10	34,923,899	G	A	0.713	1	PARD3
rs117122986	10	34,923,916	C	T	0.965	0.954	PARD3
rs4934642	10	34,924,966	A	C	0.712	1	PARD3
rs2490809	10	34,925,075	T	C	0.985	0.976	PARD3
rs61841123	10	34,925,104	G	A	0.949	0.997	PARD3
rs2490810	10	34,925,145	C	T	0.637	1	PARD3
rs2490811	10	34,925,667	G	A	0.637	1	PARD3
rs11009844	10	34,926,653	A	C	0.709	0.996	PARD3
rs2490812	10	34,927,301	T	C	0.631	1	PARD3
rs75013255	10	34,928,835	G	A	0.955	0.997	PARD3
rs141980352	10	34,929,592	G	A	0.982	0.916	PARD3
rs556419861	10	34,929,636	C	CAAAA	0.013	0.829	PARD3
rs2490814	10	34,930,175	C	T	0.631	0.999	PARD3
rs117267981	10	34,930,353	C	T	0.984	0.949	PARD3
rs2496721	10	34,930,751	A	G	0.636	0.999	PARD3
rs35966771	10	34,931,360	G	GCAC	0.636	0.997	PARD3
rs2490815	10	34,931,689	T	C	0.636	0.999	PARD3
rs2031335	10	34,932,228	T	C	0.712	0.999	PARD3
rs146538467	10	34,932,716	G	A	0.925	0.998	PARD3
rs2249715	10	34,933,064	T	A	0.638	0.999	PARD3
rs2001536	10	34,934,129	G	A	0.646	0.99	PARD3
rs883065	10	34,934,918	C	T	0.638	0.999	PARD3
rs117618774	10	34,934,919	G	A	0.976	0.956	PARD3
rs883066	10	34,935,154	G	A	0.643	0.999	PARD3
rs17491878	10	34,935,418	T	A	0.982	0.987	PARD3
rs12049675	10	34,936,953	C	T	0.719	0.998	PARD3
rs80346441	10	34,937,423	C	T	0.96	0.969	PARD3

SNP	Chromosome	Position	Effect Allele	Other Allele	Effect Allele Frequency	Imputation quality	Gene
rs2253088	10	34,937,639	T	C	0.636	0.999	PARD3
rs75194137	10	34,937,655	A	G	0.973	0.957	PARD3
rs2263157	10	34,939,124	C	T	0.638	0.999	PARD3
10:34939939_CAAAAAAAAAAAAAAAAAAAAAAA_C	10	34,939,939	CAAAAAAAAAAAAAAAAAAAAAAA	C	0.256	0.92	PARD3
rs557458623	10	34,939,940	A	C	0.963	0.964	PARD3
rs2490817	10	34,941,134	G	A	0.689	0.999	PARD3
10:34942722_CA_C	10	34,942,722	CA	C	0.5	0.918	PARD3
rs2489657	10	34,943,040	T	C	0.687	0.999	PARD3
rs10508809	10	34,943,898	C	T	0.763	0.999	PARD3
rs2489656	10	34,944,141	A	G	0.682	0.999	PARD3
rs3002030	10	34,944,610	C	T	0.687	0.999	PARD3
rs9418107	10	34,945,341	T	A	0.687	0.999	PARD3
rs7911185	10	34,945,656	A	C	0.924	0.998	PARD3
rs7915232	10	34,946,063	G	A	0.924	0.998	PARD3
rs141341650	10	34,946,516	G	A	0.945	0.955	PARD3
rs55637815	10	34,947,359	C	T	0.931	0.983	PARD3
rs76643003	10	34,947,745	A	G	0.982	0.986	PARD3
rs116928365	10	34,948,810	T	C	0.984	0.887	PARD3
rs2496724	10	34,950,172	C	T	0.683	0.999	PARD3
rs2489661	10	34,950,521	G	A	0.683	0.999	PARD3
rs2496727	10	34,951,044	C	T	0.683	0.999	PARD3
rs117927812	10	34,951,096	C	T	0.981	0.996	PARD3
10:34951799_GAA_G	10	34,951,799	GAA	G	0.675	0.974	PARD3
rs2496728	10	34,951,834	C	T	0.683	0.997	PARD3
rs2489659	10	34,951,837	C	G	0.677	0.997	PARD3
rs2496729	10	34,952,764	A	G	0.681	0.997	PARD3
rs2476994	10	34,954,172	C	T	0.677	0.997	PARD3
rs200707284	10	34,954,289	AC	A	0.695	0.967	PARD3
rs61408800	10	34,954,290	C	A	0.97	0.637	PARD3
rs12773194	10	34,954,301	A	T	0.683	0.997	PARD3
rs191430826	10	34,954,542	C	A	0.982	0.982	PARD3
rs529558435	10	34,954,768	A	C	0.977	0.683	PARD3
rs559910750	10	34,955,145	C	CAA	0.683	0.992	PARD3
rs2476995	10	34,955,317	T	C	0.682	0.995	PARD3
rs2496731	10	34,955,387	C	T	0.681	0.995	PARD3
rs10827389	10	34,957,566	A	G	0.595	0.994	PARD3
rs2025460	10	34,957,681	G	A	0.718	0.996	PARD3
rs80355788	10	34,957,749	A	T	0.977	0.935	PARD3
rs2476997	10	34,958,429	A	C	0.716	0.997	PARD3
rs184781252	10	34,958,707	A	T	0.989	0.966	PARD3
rs11596284	10	34,958,841	A	C	0.946	0.986	PARD3
rs2489653	10	34,959,199	C	T	0.969	0.992	PARD3
rs17492254	10	34,959,285	T	C	0.601	0.999	PARD3
rs4934643	10	34,960,077	C	T	0.312	0.999	PARD3
rs11009858	10	34,960,288	C	T	0.601	0.999	PARD3
rs72505226	10	34,960,797	T	TA	0.312	0.999	PARD3
rs2476998	10	34,961,169	C	T	0.717	0.999	PARD3
rs2477000	10	34,962,461	A	G	0.717	0.999	PARD3
rs2496733	10	34,962,886	A	G	0.313	0.999	PARD3
rs2477001	10	34,962,926	C	A	0.309	0.995	PARD3
rs77549757	10	34,962,931	A	G	0.623	0.968	PARD3
rs75879646	10	34,963,398	C	G	0.879	0.987	PARD3
rs189267134	10	34,963,768	C	T	0.984	0.888	PARD3
rs2496734	10	34,963,894	G	A	0.317	1	PARD3
rs7893932	10	34,964,046	T	C	0.747	0.998	PARD3
rs76632395	10	34,964,310	A	T	0.98	0.956	PARD3
rs2489664	10	34,964,725	A	G	0.318	1	PARD3
rs2477002	10	34,965,409	C	T	0.313	0.999	PARD3
rs3002031	10	34,965,703	C	T	0.317	0.999	PARD3
rs566964098	10	34,966,406	T	A	0.987	0.797	PARD3
rs547268989	10	34,966,772	T	TA	0.989	0.727	PARD3
rs148878124	10	34,966,930	T	TAC	0.247	0.59	PARD3
rs117726181	10	34,966,976	T	C	0.879	0.991	PARD3
rs77429782	10	34,967,256	C	A	0.974	0.976	PARD3
rs118158327	10	34,968,998	T	A	0.983	0.946	PARD3
10:34969485_TA_T	10	34,969,485	TA	T	0.299	0.954	PARD3
rs535068797	10	34,969,768	G	C	0.975	0.941	PARD3
rs2496736	10	34,970,233	A	G	0.317	1	PARD3
rs1060486	10	34,970,982	T	A	0.317	1	PARD3
rs11598578	10	34,971,389	T	C	0.599	1	PARD3
rs148877507	10	34,971,523	C	T	0.97	0.994	PARD3
rs942431	10	34,972,787	T	C	0.718	0.999	PARD3
rs2496737	10	34,972,844	T	C	0.312	0.999	PARD3
rs942430	10	34,972,946	T	C	0.317	1	PARD3
rs576346690	10	34,973,748	C	CAT	0.639	0.943	PARD3
rs2985300	10	34,973,764	T	C	0.77	0.93	PARD3
rs117359120	10	34,973,776	T	C	0.6	0.998	PARD3
rs2985301	10	34,973,944	T	C	0.317	1	PARD3
rs2985302	10	34,974,285	G	T	0.317	1	PARD3
rs766247837	10	34,974,524	C	CT	0.297	0.954	PARD3
rs2477004	10	34,974,668	C	T	0.317	1	PARD3
rs2985303	10	34,974,831	T	A	0.718	1	PARD3
rs2477005	10	34,975,125	A	G	0.317	1	PARD3
10:34975364_TA_T	10	34,975,364	TA	T	0.36	0.902	PARD3
rs12219302	10	34,975,564	G	A	0.317	1	PARD3
rs11009873	10	34,975,657	A	G	0.317	1	PARD3
rs10827390	10	34,975,724	C	A	0.317	1	PARD3
rs184610125	10	34,975,895	T	G	0.986	0.905	PARD3
rs11009874	10	34,975,951	C	T	0.317	1	PARD3
rs74359722	10	34,976,840	C	T	0.976	0.95	PARD3
rs3002032	10	34,977,585	T	C	0.317	1	PARD3
rs117775373	10	34,978,225	G	C	0.982	0.993	PARD3
10:34978861_TGTTA_T	10	34,978,861	TGTTA	T	0.661	0.932	PARD3
rs76200555	10	34,979,430	A	C	0.979	0.975	PARD3
rs2477006	10	34,979,687	A	G	0.97	0.993	PARD3
rs79103227	10	34,979,863	G	A	0.982	0.953	PARD3
rs2477007	10	34,979,906	C	G	0.312	1	PARD3
rs116923783	10	34,980,021	A	G	0.98	0.977	PARD3
rs55907048	10	34,980,221	C	A	0.956	0.971	PARD3
10:34980728_CATCT_C	10	34,980,728	CATCT	C	0.682	1	PARD3
rs10400190	10	34,980,881	C	T	0.683	1	PARD3
rs10400191	10	34,980,926	G	A	0.683	1	PARD3
rs10400192	10	34,980,933	G	A	0.683	1	PARD3
rs10400006	10	34,981,030	T	C	0.683	1	PARD3
rs775935249	10	34,981,160	CA	C	0.395	0.73	PARD3
rs2477011	10	34,981,529	G	A	0.683	1	PARD3
rs11009875	10	34,981,559	T	C	0.599	1	PARD3
rs12254240	10	34,981,797	A	G	0.599	1	PARD3
rs2477010	10	34,981,978	T	A	0.683	1	PARD3
rs2496738	10	34,981,987	C	T	0.317	1	PARD3
rs2496739	10	34,983,060	A	G	0.312	1	PARD3
10:34983082_GAAAA_G	10	34,983,082	GAAAA	G	0.605	0.972	PARD3
rs778967660	10	34,983,090	AAAAG	A	0.605	0.972	PARD3
rs199740347	10	34,983,094	G	C	0.944	0.609	PARD3
rs2496740	10	34,983,280	T	C	0.317	1	PARD3
rs144451670	10	34,983,287	T	G	0.988	0.962	PARD3
rs10763996	10	34,983,885	T	C	0.317	1	PARD3
rs111542057	10	34,983,995	G	GTGCTAC	0.318	0.998	PARD3
rs190372329	10	34,984,357	G	C	0.983	0.974	PARD3
rs4934644	10	34,984,466	G	A	0.317	1	PARD3
rs72781626	10	34,984,498	C	T	0.988	0.887	PARD3
rs2496741	10	34,984,849	A	T	0.317	1	PARD3
rs2496742	10	34,984,884	C	T	0.317	1	PARD3
rs17571153	10	34,985,068	G	A	0.963	0.966	PARD3
rs5784425	10	34,985,197	C	CA	0.316	0.997	PARD3
rs7904305	10	34,985,984	C	A	0.321	0.986	PARD3
rs11440199	10	34,986,032	G	GA	0.334	0.97	PARD3
rs3002033	10	34,986,474	T	A	0.317	1	PARD3
rs2985304	10	34,986,699	T	A	0.317	1	PARD3
rs3002034	10	34,986,853	T	A	0.317	1	PARD3
rs11009878	10	34,987,749	T	C	0.317	1	PARD3
rs5784426	10	34,988,256	T	TA	0.317	0.999	PARD3
rs2496744	10	34,988,379	C	T	0.317	1	PARD3
rs2245124	10	34,988,455	G	C	0.317	1	PARD3
rs2496745	10	34,989,041	G	A	0.721	0.998	PARD3
rs534224961	10	34,990,259	G	GGAT	0.634	0.947	PARD3
rs11594939	10	34,990,264	T	A	0.747	0.747	PARD3
rs3002035	10	34,990,762	G	A	0.317	0.996	PARD3
rs142847016	10	34,992,281	C	T	0.98	0.948	PARD3
10:34992388_GAA_G	10	34,992,388	GAA	G	0.308	0.975	PARD3
rs1324301	10	34,992,669	A	G	0.317	0.994	PARD3
rs10827393	10	34,992,826	T	C	0.683	0.994	PARD3
rs6481907	10	34,994,191	A	G	0.683	0.993	PARD3
rs7908758	10	34,994,352	A	G	0.683	0.993	PARD3
rs7908983	10	34,994,403	C	G	0.282	0.993	PARD3
rs557564980	10	34,994,418	G	GA	0.702	0.928	PARD3
rs10827394	10	34,995,034	C	T	0.688	0.992	PARD3
rs7081764	10	34,995,120	T	A	0.599	0.993	PARD3
rs35081978	10	34,995,675	T	A	0.748	0.992	PARD3
rs36035625	10	34,997,195	G	GA	0.699	0.899	PARD3
rs10827395	10	34,997,538	T	C	0.688	0.99	PARD3
rs6481908	10	34,997,892	G	A	0.036	0.984	PARD3
rs76253757	10	34,998,238	G	A	0.988	0.954	PARD3
rs113362484	10	34,998,606	G	T	0.988	0.928	PARD3
rs72783645	10	34,998,945	C	G	0.862	0.865	PARD3

SNP	Chromosome	Position	Effect Allele	Other Allele	Effect Allele Frequency	Imputation quality	Gene
rs151111066	10	34,999,179	A	G	0.982	0.955	PARD3
rs6481909	10	34,999,300	C	T	0.323	0.989	PARD3
rs12220105	10	34,999,497	T	C	0.594	0.995	PARD3
rs4485051	10	34,999,785	T	G	0.722	0.992	PARD3
rs10827396	10	34,999,903	A	G	0.594	0.995	PARD3
rs35418769	10	34,999,913	G	C	0.726	0.991	PARD3
rs11009882	10	35,000,285	T	C	0.722	0.993	PARD3
rs137952587	10	35,000,678	G	A	0.963	0.98	PARD3
rs12264807	10	35,000,846	T	C	0.722	0.993	PARD3
rs11009884	10	35,001,472	T	G	0.726	0.991	PARD3
rs10827398	10	35,001,480	G	A	0.722	0.993	PARD3
rs7920227	10	35,001,932	C	T	0.316	0.994	PARD3
rs10827399	10	35,003,979	C	T	0.722	0.996	PARD3
10:35004004_CT_C	10	35,004,004	CT	C	0.562	0.96	PARD3
rs10827400	10	35,004,107	G	A	0.594	0.999	PARD3
rs11009886	10	35,004,171	C	T	0.722	0.996	PARD3
rs11598077	10	35,004,448	C	G	0.96	0.984	PARD3
rs4622206	10	35,005,969	G	T	0.594	1	PARD3
rs4622207	10	35,005,973	G	A	0.594	1	PARD3
rs10740893	10	35,006,638	T	C	0.316	0.997	PARD3
rs10827404	10	35,007,012	G	A	0.722	0.997	PARD3
rs189303056	10	35,007,030	C	A	0.979	0.953	PARD3
rs10740894	10	35,007,309	T	C	0.316	0.997	PARD3
rs11009888	10	35,008,098	T	C	0.721	0.996	PARD3
rs12781515	10	35,008,159	T	A	0.594	1	PARD3
rs4934645	10	35,008,523	C	A	0.04	0.984	PARD3
rs10827405	10	35,008,862	G	A	0.594	1	PARD3
rs10827406	10	35,009,620	G	T	0.722	0.996	PARD3
rs7075990	10	35,009,682	G	A	0.594	1	PARD3
rs377520129	10	35,009,726	C	CA	0.613	0.966	PARD3
rs12356453	10	35,009,728	A	G	0.74	0.962	PARD3
rs10827407	10	35,010,609	T	G	0.594	1	PARD3
rs11009889	10	35,010,711	T	C	0.594	1	PARD3
rs7905697	10	35,010,993	G	A	0.316	0.997	PARD3
rs10763997	10	35,012,181	A	G	0.594	0.999	PARD3
10:35012505_CTGTT_C	10	35,012,505	CTGTT	C	0.983	0.935	PARD3
rs10218857	10	35,013,218	C	T	0.316	0.997	PARD3
rs10827408	10	35,013,379	T	A	0.594	1	PARD3
rs7091786	10	35,013,632	G	A	0.316	0.997	PARD3
rs61843471	10	35,014,081	C	G	0.306	0.976	PARD3
rs139040840	10	35,014,082	A	T	0.306	0.976	PARD3
rs146738041	10	35,014,085	G	C	0.306	0.976	PARD3
rs12772997	10	35,014,390	A	T	0.594	1	PARD3
rs10508811	10	35,014,434	T	G	0.722	0.996	PARD3
rs7080111	10	35,014,456	T	C	0.594	1	PARD3
10:35015219_AAC_A	10	35,015,219	AAC	A	0.987	0.986	PARD3
rs10763998	10	35,015,739	T	C	0.728	0.985	PARD3
rs199738657	10	35,016,498	AT	A	0.039	0.956	PARD3
rs11009892	10	35,016,575	T	C	0.729	0.993	PARD3
rs4339985	10	35,016,625	C	T	0.316	0.997	PARD3
rs11462023	10	35,017,087	A	AT	0.316	0.994	PARD3
rs79596836	10	35,018,007	A	C	0.949	0.961	PARD3
rs11009893	10	35,018,285	C	T	0.594	1	PARD3
rs11009894	10	35,018,450	C	T	0.722	0.994	PARD3
rs374891935	10	35,018,458	C	CA	0.62	0.948	PARD3
rs11009896	10	35,019,671	C	T	0.594	0.999	PARD3
rs72783649	10	35,019,813	G	A	0.974	0.978	PARD3
rs11009897	10	35,019,863	T	C	0.594	0.999	PARD3
rs142000229	10	35,019,903	T	A	0.987	0.917	PARD3
rs11009898	10	35,020,006	T	G	0.594	0.999	PARD3
rs142554724	10	35,020,047	A	AT	0.313	0.989	PARD3
rs11426700	10	35,020,055	A	T	0.978	0.991	PARD3
rs78338602	10	35,020,083	T	C	0.981	0.999	PARD3
rs11009899	10	35,020,507	T	C	0.594	0.999	PARD3
rs11009900	10	35,020,638	T	C	0.594	0.999	PARD3
rs11009901	10	35,020,982	G	A	0.594	0.999	PARD3
rs150792167	10	35,021,291	G	A	0.986	0.914	PARD3
rs35190695	10	35,021,483	G	A	0.596	0.998	PARD3
rs10128209	10	35,021,701	C	T	0.716	0.99	PARD3
rs149691167	10	35,022,218	T	C	0.981	0.96	PARD3
rs61843474	10	35,022,490	C	G	0.594	0.999	PARD3
rs11448579	10	35,022,876	G	GT	0.604	0.963	PARD3
rs11009903	10	35,023,778	C	G	0.723	0.996	PARD3
rs10128259	10	35,023,789	G	A	0.316	0.996	PARD3
rs143164280	10	35,023,912	T	TG	0.601	0.99	PARD3
rs34568689	10	35,023,915	GA	G	0.787	0.821	PARD3
rs11009904	10	35,024,344	T	C	0.869	0.986	PARD3
rs10732429	10	35,025,573	A	T	0.046	0.979	PARD3
rs10734017	10	35,025,577	A	T	0.443	0.922	PARD3
rs4934648	10	35,025,585	T	A	0.046	0.979	PARD3
rs61240130	10	35,025,589	T	A	0.78	0.961	PARD3
rs75148957	10	35,025,593	T	A	0.93	0.878	PARD3
rs79603736	10	35,026,002	A	T	0.959	0.991	PARD3
rs10450314	10	35,026,141	G	A	0.723	0.996	PARD3
rs4259777	10	35,026,575	T	C	0.317	0.993	PARD3
rs10636688	10	35,027,445	C	CTT	0.315	0.994	PARD3
rs11009905	10	35,027,614	G	A	0.98	0.999	PARD3
rs61843476	10	35,028,164	G	A	0.593	0.997	PARD3
rs7075263	10	35,028,713	T	C	0.593	0.998	PARD3
rs10827411	10	35,028,784	T	C	0.723	0.996	PARD3
rs7476052	10	35,029,689	T	C	0.723	0.996	PARD3
rs11009908	10	35,029,862	G	T	0.594	0.998	PARD3
rs11009909	10	35,030,216	G	A	0.593	0.998	PARD3
rs11599163	10	35,030,501	T	C	0.593	0.997	PARD3
rs10827412	10	35,031,382	T	G	0.732	0.983	PARD3
rs17493513	10	35,031,758	G	A	0.526	0.996	PARD3
rs117368234	10	35,031,910	G	A	0.984	0.957	PARD3
rs11009911	10	35,031,934	A	T	0.266	0.997	PARD3
rs11009912	10	35,033,116	G	A	0.741	0.997	PARD3
rs10827413	10	35,033,607	C	G	0.525	0.998	PARD3
rs16935679	10	35,033,772	G	A	0.741	0.997	PARD3
rs140960424	10	35,034,359	G	GGAAAGAGAGAGA	0.266	0.995	PARD3
rs12572123	10	35,034,757	G	A	0.741	0.998	PARD3
rs35355141	10	35,034,962	G	A	0.525	0.998	PARD3
rs11009914	10	35,035,327	T	C	0.742	0.997	PARD3
rs11009915	10	35,035,420	C	T	0.741	0.998	PARD3
rs11009916	10	35,036,847	G	A	0.741	0.998	PARD3
rs12761460	10	35,037,042	C	T	0.525	0.998	PARD3
rs12761677	10	35,037,123	C	T	0.525	0.998	PARD3
rs61843488	10	35,037,163	T	C	0.741	0.998	PARD3
rs12761879	10	35,037,231	C	G	0.525	0.998	PARD3
rs10732430	10	35,038,089	G	A	0.266	0.998	PARD3
rs11009917	10	35,038,881	C	T	0.525	0.998	PARD3
10:35039286_AAG_A	10	35,039,286	AAG	A	0.525	0.996	PARD3
10:35039390_AACACAC_A	10	35,039,390	AACACAC	A	0.307	0.981	PARD3
rs12416213	10	35,039,469	C	T	0.741	0.999	PARD3
rs10643701	10	35,039,619	A	ATTT	0.725	0.952	PARD3
rs7908823	10	35,039,774	A	G	0.038	0.993	PARD3
rs7909296	10	35,039,864	G	A	0.741	0.999	PARD3
10:35040144_TA_T	10	35,040,144	TA	T	0.252	0.975	PARD3
rs7909790	10	35,040,201	G	T	0.741	0.999	PARD3
rs7086123	10	35,040,504	G	A	0.741	0.999	PARD3
rs56386890	10	35,040,525	C	T	0.967	0.978	PARD3
rs7086042	10	35,040,640	C	T	0.267	0.998	PARD3
rs11813991	10	35,040,655	T	C	0.526	0.998	PARD3
rs546186706	10	35,040,699	C	CT	0.79	0.859	PARD3
rs78661820	10	35,041,595	A	G	0.975	0.977	PARD3
rs113014456	10	35,041,880	C	CTCTA	0.741	0.998	PARD3
rs17578633	10	35,041,889	T	C	0.968	0.96	PARD3
rs7079424	10	35,042,460	T	C	0.04	0.995	PARD3
rs10827416	10	35,043,058	T	C	0.742	1	PARD3
rs7923244	10	35,043,405	G	A	0.742	1	PARD3
rs200851860	10	35,044,697	CT	C	0.265	0.996	PARD3
rs10827417	10	35,044,728	A	G	0.742	1	PARD3
rs10740895	10	35,045,009	A	T	0.266	0.999	PARD3
10:35045388_CT_C	10	35,045,388	CT	C	0.505	0.996	PARD3
rs10827418	10	35,045,835	C	A	0.742	1	PARD3
rs10763999	10	35,046,789	C	G	0.742	1	PARD3
rs10764000	10	35,046,802	C	A	0.742	1	PARD3
rs10508812	10	35,047,413	G	A	0.742	1	PARD3
rs7906411	10	35,047,919	T	C	0.741	1	PARD3
rs117883695	10	35,048,058	A	G	0.981	0.933	PARD3
rs10508813	10	35,048,855	T	C	0.742	1	PARD3
10:35049119_ACAGCG_A	10	35,049,119	ACAGCG	A	0.267	0.99	PARD3
rs10827419	10	35,049,209	A	G	0.741	1	PARD3
rs7895074	10	35,049,515	C	T	0.742	1	PARD3
rs7915211	10	35,049,858	T	G	0.741	0.999	PARD3
rs7074053	10	35,050,214	C	A	0.967	0.98	PARD3
rs11009923	10	35,050,573	T	C	0.741	0.999	PARD3
rs117428613	10	35,051,060	A	G	0.976	0.966	PARD3
rs139337549	10	35,051,187	G	GGAGAGAGA	0.74	0.998	PARD3
rs796709989	10	35,051,218	CAG	C	0.71	0.947	PARD3
rs11009924	10	35,051,336	C	T	0.741	0.999	PARD3
rs10764001	10	35,052,062	G	A	0.741	0.999	PARD3
rs4076544	10	35,052,212	T	G	0.742	0.999	PARD3

SNP	Chromosome	Position	Effect Allele	Other Allele	Effect Allele Frequency	Imputation quality	Gene
rs77303985	10	35,052,598	T	C	0.97	0.999	PARD3
rs12413778	10	35,052,646	G	C	0.742	0.999	PARD3
rs11009925	10	35,052,961	C	T	0.741	0.999	PARD3
rs10827420	10	35,053,048	A	C	0.742	0.999	PARD3
rs12358036	10	35,054,112	G	A	0.98	0.993	PARD3
rs10764003	10	35,054,190	T	A	0.266	1	PARD3
rs58534530	10	35,054,376	C	T	0.935	0.967	PARD3
rs112970917	10	35,054,599	C	CA	0.747	0.969	PARD3
rs11009926	10	35,054,766	A	C	0.742	1	PARD3
rs80304970	10	35,054,944	G	T	0.984	0.985	PARD3
rs10764004	10	35,055,185	G	T	0.742	1	PARD3
rs10764005	10	35,055,464	C	T	0.742	1	PARD3
rs10740896	10	35,055,496	A	G	0.266	1	PARD3
rs146779048	10	35,055,545	CA	C	0.499	0.892	PARD3
rs57862696	10	35,055,633	G	GA	0.741	0.998	PARD3
rs61840961	10	35,056,125	T	A	0.742	1	PARD3
rs16935716	10	35,056,297	T	C	0.742	1	PARD3
rs16935717	10	35,056,323	T	C	0.742	1	PARD3
10:35056528_CTGTT_C	10	35,056,528	CTGTT	C	0.743	0.993	PARD3
rs117119992	10	35,056,569	C	T	0.975	0.983	PARD3
rs7099563	10	35,056,776	T	C	0.742	1	PARD3
rs12572476	10	35,056,975	T	C	0.742	1	PARD3
rs28483914	10	35,057,266	A	G	0.742	0.999	PARD3
rs61840962	10	35,057,540	C	T	0.742	0.999	PARD3
rs12783964	10	35,057,788	C	T	0.78	0.997	PARD3
rs16935722	10	35,057,828	A	C	0.742	0.999	PARD3
rs61281038	10	35,057,854	C	A	0.742	0.999	PARD3
rs9418115	10	35,058,057	C	T	0.742	0.999	PARD3
rs77196258	10	35,058,317	C	T	0.973	0.973	PARD3
rs9417140	10	35,058,509	A	C	0.742	0.999	PARD3
rs9417141	10	35,058,576	C	T	0.742	0.999	PARD3
10:35058695_AAAAC_A	10	35,058,695	AAAAC	A	0.747	0.984	PARD3
rs11009928	10	35,058,712	A	G	0.747	0.985	PARD3
rs59494394	10	35,058,716	G	A	0.985	0.806	PARD3
rs772597252	10	35,058,732	C	CA	0.044	0.685	PARD3
rs9418116	10	35,058,877	C	G	0.742	0.999	PARD3
rs9417142	10	35,058,930	C	T	0.742	0.999	PARD3
rs9418117	10	35,059,013	C	T	0.742	0.999	PARD3
rs9417143	10	35,059,237	T	C	0.742	0.999	PARD3
rs10827421	10	35,059,666	C	T	0.524	0.999	PARD3
rs79478455	10	35,059,834	C	T	0.955	0.968	PARD3
rs7080176	10	35,059,869	T	C	0.741	0.998	PARD3
rs79923002	10	35,060,048	G	C	0.979	0.99	PARD3
rs9418118	10	35,060,180	T	G	0.742	0.996	PARD3
rs7080745	10	35,060,247	T	C	0.875	0.99	PARD3
rs9418119	10	35,060,341	C	T	0.74	0.998	PARD3
rs75787859	10	35,060,457	A	G	0.963	0.992	PARD3
rs9418120	10	35,060,501	G	A	0.74	0.998	PARD3
rs11599721	10	35,061,310	T	G	0.526	0.998	PARD3
rs4600153	10	35,062,404	A	T	0.266	0.999	PARD3
rs35585382	10	35,063,647	A	AT	0.74	0.996	PARD3
rs117022900	10	35,064,706	A	G	0.979	0.963	PARD3
rs9418121	10	35,065,245	C	T	0.74	0.997	PARD3
rs77632106	10	35,065,495	T	C	0.988	0.864	PARD3
rs144499144	10	35,067,205	G	C	0.972	0.971	PARD3
rs180796451	10	35,068,150	C	T	0.987	0.952	PARD3
rs191808829	10	35,068,276	G	A	0.972	0.972	PARD3
rs7904348	10	35,068,814	A	G	0.298	0.992	PARD3
rs138178469	10	35,069,356	T	C	0.981	0.965	PARD3
10:35072532_CAA_C	10	35,072,532	CAA	C	0.375	0.851	PARD3
10:35073383_TTG_T	10	35,073,383	TTG	T	0.927	0.66	PARD3
10:35073384_TG_T	10	35,073,384	TG	T	0.771	0.994	PARD3
rs754259755	10	35,073,385	G	T	0.985	0.524	PARD3
rs7912166	10	35,074,019	T	C	0.769	1	PARD3
rs7907833	10	35,074,846	G	C	0.769	1	PARD3
rs7907947	10	35,074,901	G	A	0.769	1	PARD3
rs759862669	10	35,074,980	CAT	C	0.815	0.995	PARD3
rs6481911	10	35,075,379	T	C	0.769	1	PARD3
rs12763418	10	35,075,793	T	C	0.494	0.998	PARD3
rs7897283	10	35,075,817	T	G	0.769	1	PARD3
rs7912526	10	35,076,034	G	C	0.769	1	PARD3
rs7912348	10	35,076,127	A	G	0.769	1	PARD3
rs7092771	10	35,076,794	A	G	0.262	0.999	PARD3
rs74494347	10	35,076,987	C	T	0.989	0.994	PARD3
rs12769125	10	35,077,003	C	T	0.493	0.999	PARD3
rs67427607	10	35,077,232	G	A	0.769	1	PARD3
rs4271337	10	35,077,393	C	T	0.124	0.989	PARD3
rs117404090	10	35,077,904	T	C	0.985	0.998	PARD3
rs4934654	10	35,078,092	C	T	0.769	1	PARD3
rs4934655	10	35,078,287	C	G	0.262	0.998	PARD3
rs4934656	10	35,078,305	G	A	0.769	1	PARD3
rs4934657	10	35,078,593	C	T	0.769	1	PARD3
rs200691336	10	35,078,637	G	GC	0.985	0.776	PARD3
10:35078674_CACAA_C	10	35,078,674	CACAA	C	0.769	0.998	PARD3
rs11009935	10	35,078,967	T	C	0.769	1	PARD3
rs151229916	10	35,078,985	T	C	0.966	0.985	PARD3
rs7068716	10	35,079,064	A	G	0.262	0.998	PARD3
rs7069244	10	35,079,165	G	T	0.769	1	PARD3
rs10764006	10	35,079,874	A	T	0.262	0.998	PARD3
rs11593745	10	35,079,883	G	A	0.963	0.996	PARD3
rs10827425	10	35,079,904	A	G	0.493	0.998	PARD3
rs7074701	10	35,080,661	G	C	0.493	0.999	PARD3
rs11598664	10	35,080,743	A	G	0.769	1	PARD3
rs117915141	10	35,081,110	G	A	0.938	0.965	PARD3
rs4934658	10	35,081,336	C	T	0.262	0.998	PARD3
rs7907359	10	35,081,678	G	C	0.769	1	PARD3
rs7907384	10	35,081,755	G	T	0.769	1	PARD3
rs4934659	10	35,081,865	A	C	0.769	1	PARD3
rs11009936	10	35,082,453	A	G	0.769	1	PARD3
rs11009937	10	35,082,526	C	T	0.82	0.996	PARD3
rs7087523	10	35,082,740	A	G	0.77	0.999	PARD3
rs7088268	10	35,083,158	C	T	0.769	1	PARD3
rs7088938	10	35,083,381	G	T	0.769	1	PARD3
rs10827426	10	35,083,392	T	C	0.493	0.999	PARD3
10:35083786_AT_A	10	35,083,786	AT	A	0.942	0.963	PARD3
rs11009938	10	35,084,080	A	G	0.769	1	PARD3
rs11009939	10	35,084,200	C	T	0.769	1	PARD3
rs11009940	10	35,084,277	A	G	0.769	1	PARD3
rs11009941	10	35,084,445	T	G	0.769	0.999	PARD3
rs4315011	10	35,084,809	T	C	0.126	0.997	PARD3
rs186650571	10	35,084,868	T	C	0.985	0.944	PARD3
rs4391770	10	35,085,417	G	T	0.769	1	PARD3
rs4331033	10	35,086,203	C	G	0.769	1	PARD3
rs4440973	10	35,086,204	G	A	0.769	0.999	PARD3
rs4539260	10	35,086,350	A	G	0.262	0.998	PARD3
rs4430454	10	35,086,365	G	C	0.262	0.998	PARD3
rs4934515	10	35,086,655	T	C	0.769	1	PARD3
rs4934660	10	35,086,976	G	A	0.769	1	PARD3
10:35087090_GA_G	10	35,087,090	GA	G	0.768	0.996	PARD3
rs7906382	10	35,087,192	A	G	0.769	1	PARD3
rs11009942	10	35,087,431	A	G	0.769	1	PARD3
rs11594839	10	35,087,515	C	T	0.493	0.999	PARD3
rs11009943	10	35,087,526	A	G	0.77	0.999	PARD3
rs56259405	10	35,087,565	A	G	0.769	1	PARD3
rs4472878	10	35,087,581	C	G	0.769	1	PARD3
10:35087600_TC_T	10	35,087,600	TC	T	0.774	0.987	PARD3
rs76302767	10	35,087,633	CA	C	0.36	0.916	PARD3
rs143339106	10	35,088,060	C	T	0.985	0.926	PARD3
rs772986078	10	35,088,755	C	CA	0.765	0.983	PARD3
rs547315296	10	35,088,783	T	G	0.986	0.568	PARD3
rs6481913	10	35,088,789	C	T	0.769	1	PARD3
rs12766926	10	35,088,959	G	A	0.493	0.999	PARD3
10:35088984_GGAGGTT_G	10	35,088,984	GGAGGTT	G	0.979	0.936	PARD3
10:35089069_CA_C	10	35,089,069	CA	C	0.765	0.976	PARD3
rs139322518	10	35,089,356	G	A	0.989	0.991	PARD3
rs4934661	10	35,089,478	G	A	0.815	0.999	PARD3
rs4277058	10	35,090,964	G	C	0.493	0.999	PARD3
rs11009944	10	35,091,600	A	G	0.769	1	PARD3
rs10827427	10	35,091,766	G	A	0.262	0.998	PARD3
rs11597833	10	35,091,864	G	A	0.769	1	PARD3
rs780681187	10	35,091,998	G	GAA	0.824	0.869	PARD3
rs11597893	10	35,092,162	G	A	0.769	1	PARD3
rs112366679	10	35,092,178	T	TAAATA	0.263	0.993	PARD3
rs12765143	10	35,092,356	T	A	0.742	0.997	PARD3
rs12243180	10	35,092,763	T	A	0.769	1	PARD3
rs7095518	10	35,093,584	T	C	0.769	1	PARD3
rs7098992	10	35,093,759	T	C	0.769	1	PARD3
rs7081365	10	35,093,995	A	G	0.769	1	PARD3
rs7081495	10	35,094,013	C	T	0.963	0.995	PARD3
rs7081501	10	35,094,023	C	T	0.769	1	PARD3
rs138847588	10	35,094,040	C	T	0.978	0.974	PARD3
rs56276736	10	35,094,494	T	C	0.77	0.999	PARD3
rs55847352	10	35,094,496	C	T	0.77	0.999	PARD3
rs11009945	10	35,094,633	G	A	0.764	0.99	PARD3
rs11009946	10	35,094,643	G	A	0.769	1	PARD3

SNP	Chromosome	Position	Effect Allele	Other Allele	Effect Allele Frequency	Imputation quality	Gene
rs72783690	10	35,094,991	C	T	0.769	1	PARD3
rs570042072	10	35,095,125	A	AT	0.529	0.862	PARD3
rs117140457	10	35,095,289	A	G	0.981	0.949	PARD3
rs11009947	10	35,095,396	C	T	0.493	0.999	PARD3
rs10827428	10	35,095,524	T	A	0.769	1	PARD3
rs7095898	10	35,095,587	G	C	0.77	1	PARD3
rs116500759	10	35,096,052	C	A	0.963	0.995	PARD3
rs7099649	10	35,096,100	C	G	0.769	1	PARD3
10:35096318_TA_T	10	35,096,318	TA	T	0.985	0.999	PARD3
rs12220026	10	35,096,416	G	A	0.769	1	PARD3
rs11009948	10	35,096,465	T	C	0.769	1	PARD3
rs11009949	10	35,096,470	G	A	0.769	1	PARD3
rs10827431	10	35,096,572	G	T	0.769	1	PARD3
rs766725579	10	35,096,616	GTGTA	G	0.769	0.996	PARD3
rs10764007	10	35,096,641	A	G	0.769	1	PARD3
rs117343503	10	35,096,663	C	T	0.979	0.975	PARD3
rs10827432	10	35,096,683	T	C	0.769	1	PARD3
rs10827433	10	35,096,860	A	G	0.769	1	PARD3
rs10827434	10	35,096,911	T	A	0.769	1	PARD3
rs113803089	10	35,097,110	C	CACACA	0.769	0.999	PARD3
rs4934662	10	35,097,267	T	G	0.769	1	PARD3
10:35097288_CA_C	10	35,097,288	CA	C	0.772	0.993	PARD3
rs4934663	10	35,097,326	A	G	0.769	1	PARD3
rs4934664	10	35,097,388	A	C	0.769	1	PARD3
rs4934665	10	35,097,398	T	C	0.769	1	PARD3
rs4934666	10	35,097,449	T	C	0.262	0.998	PARD3
rs4934667	10	35,097,574	T	A	0.262	0.999	PARD3
rs7342019	10	35,097,933	G	T	0.769	1	PARD3
rs7342018	10	35,098,015	C	T	0.262	0.999	PARD3
rs12264595	10	35,098,158	G	C	0.769	1	PARD3
rs4934668	10	35,098,333	A	T	0.769	1	PARD3
rs11009952	10	35,098,836	T	G	0.769	1	PARD3
rs75896554	10	35,098,947	T	TTA	0.77	0.999	PARD3
rs6481914	10	35,099,586	C	G	0.77	0.997	PARD3
rs7342064	10	35,099,799	G	A	0.77	0.999	PARD3
rs11009954	10	35,100,560	A	G	0.483	0.998	PARD3
rs4934516	10	35,100,604	A	G	0.782	0.998	PARD3
rs12569527	10	35,100,812	G	A	0.866	0.998	PARD3
rs4934669	10	35,101,261	C	T	0.125	0.998	PARD3
rs4934670	10	35,101,551	A	G	0.791	0.999	PARD3
rs16935760	10	35,101,935	C	G	0.791	0.999	PARD3
rs4468299	10	35,101,955	T	C	0.125	0.998	PARD3
rs768479018	10	35,102,217	AGAGTGCATATTACT	A	0.865	0.858	PARD3
rs11009955	10	35,102,387	A	G	0.473	0.999	PARD3
rs4934671	10	35,102,818	G	A	0.833	0.998	PARD3
rs148300427	10	35,102,996	G	A	0.983	0.922	PARD3
rs73264844	10	35,103,340	A	G	0.792	0.998	PARD3
rs185778956	10	35,103,650	G	A	0.971	0.862	PARD3
rs113044591	10	35,103,684	G	A	0.792	0.999	PARD3
rs12355985	10	35,104,026	G	A	0.87	0.985	PARD3
rs193076331	10	35,104,146	T	G	0.792	0.998	PARD3
rs12356003	10	35,104,190	G	T	0.122	0.977	PARD3
rs1051246	10	81,697,818	A	G	0.878	0.999	SFTPD
rs12241521	10	81,698,095	C	A	0.916	0.995	SFTPD
rs2819098	10	81,698,097	G	A	0.407	1	SFTPD
rs17879726	10	81,698,723	G	A	0.932	1	SFTPD
rs17879583	10	81,698,768	G	A	0.956	0.996	SFTPD
rs2245121	10	81,699,238	G	A	0.573	0.999	SFTPD
rs17886630	10	81,699,309	A	T	0.878	0.999	SFTPD
rs17878456	10	81,699,563	T	C	0.968	0.992	SFTPD
rs3842640	10	81,699,723	G	GTC	0.406	0.997	SFTPD
rs17880779	10	81,699,950	A	G	0.984	0.996	SFTPD
rs2819097	10	81,699,981	C	G	0.406	1	SFTPD
rs2758545	10	81,700,090	A	G	0.406	1	SFTPD
rs12784549	10	81,700,574	C	G	0.956	0.995	SFTPD
rs12776624	10	81,700,893	T	C	0.956	0.995	SFTPD
rs17882375	10	81,701,129	C	T	0.956	0.996	SFTPD
rs911887	10	81,701,523	T	C	0.576	0.998	SFTPD
rs911886	10	81,701,580	C	T	0.968	0.995	SFTPD
rs17878558	10	81,702,353	G	C	0.988	0.919	SFTPD
rs1998374	10	81,702,722	T	C	0.901	0.999	SFTPD
rs10887199	10	81,702,834	T	C	0.901	0.999	SFTPD
rs3793925	10	81,703,373	G	C	0.969	1	SFTPD
rs17880086	10	81,703,490	G	A	0.932	0.999	SFTPD
rs17882021	10	81,703,832	C	T	0.955	0.997	SFTPD
rs17886286	10	81,703,852	C	G	0.932	0.999	SFTPD
rs2181205	10	81,704,156	G	A	0.969	0.999	SFTPD
rs2255601	10	81,704,559	A	G	0.412	0.996	SFTPD
rs7078012	10	81,705,433	C	T	0.85	0.994	SFTPD
rs12768733	10	81,705,480	T	C	0.955	0.997	SFTPD
10:81705815_AT_A	10	81,705,815	AT	A	0.978	0.883	SFTPD
10:81705843_GTTTAATTAAATA_G	10	81,705,843	GTTTAATTAAATA	G	0.969	0.999	SFTPD
rs6413523	10	81,706,135	C	A	0.969	1	SFTPD
rs6413520	10	81,706,281	A	G	0.926	0.839	SFTPD
rs721917	10	81,706,324	A	G	0.579	1	SFTPD
rs726287	10	81,706,793	A	T	0.85	0.994	SFTPD
rs726289	10	81,706,951	G	A	0.932	1	SFTPD
rs726288	10	81,706,973	C	T	0.97	0.997	SFTPD
rs2255326	10	81,707,174	G	A	0.722	0.999	SFTPD
rs7074156	10	81,707,371	G	A	0.918	0.995	SFTPD
rs2819096	10	81,707,613	A	C	0.428	0.997	SFTPD
rs723191	10	81,707,920	A	G	0.932	1	SFTPD
rs723192	10	81,708,013	C	T	0.899	0.995	SFTPD
rs958865	10	81,708,029	C	A	0.901	1	SFTPD
rs723193	10	81,708,155	G	T	0.932	1	SFTPD
rs726014	10	81,708,744	T	C	0.901	0.999	SFTPD
rs28362945	11	57,365,895	G	A	0.824	0.962	SERPING1
rs3054018	11	57,366,406	G	GTG	0.698	0.937	SERPING1
rs796798875	11	57,366,407	T	TGT	0.697	0.937	SERPING1
rs1005511	11	57,366,656	C	T	0.701	0.999	SERPING1
11:57368423_CA_C	11	57,368,423	CA	C	0.482	0.868	SERPING1
rs1812005	11	57,368,644	C	A	0.7	1	SERPING1
rs28362947	11	57,369,008	C	G	0.734	0.996	SERPING1
rs28362948	11	57,369,013	A	C	0.734	0.996	SERPING1
rs188723430	11	57,369,318	G	A	0.989	0.698	SERPING1
rs11229063	11	57,369,730	G	A	0.734	0.998	SERPING1
rs11820040	11	57,369,957	T	C	0.734	0.998	SERPING1
rs190753015	11	57,369,979	C	T	0.987	0.873	SERPING1
rs574854879	11	57,370,256	C	CA	0.662	0.983	SERPING1
rs2432810	11	57,370,301	C	T	0.7	0.999	SERPING1
rs34858800	11	57,370,400	T	TA	0.74	0.979	SERPING1
rs78364821	11	57,370,742	C	T	0.734	0.998	SERPING1
rs570525960	11	57,370,920	T	C	0.987	0.983	SERPING1
rs11229065	11	57,371,033	C	T	0.967	0.935	SERPING1
rs76525968	11	57,371,192	G	T	0.734	0.998	SERPING1
rs144256346	11	57,371,412	C	T	0.734	0.998	SERPING1
rs28362949	11	57,371,911	G	T	0.734	0.997	SERPING1
rs28362950	11	57,371,918	G	A	0.734	0.997	SERPING1
rs28362951	11	57,372,526	A	G	0.734	0.998	SERPING1
rs12806113	11	57,372,974	G	A	0.986	0.967	SERPING1
11:57373055_CT_C	11	57,373,055	CT	C	0.286	0.95	SERPING1
rs76190365	11	57,373,072	T	C	0.988	0.901	SERPING1
rs11603020	11	57,374,332	T	C	0.731	0.998	SERPING1
rs11229066	11	57,374,871	C	G	0.731	0.998	SERPING1
rs11229067	11	57,374,946	G	T	0.731	0.998	SERPING1
rs139095413	11	57,375,399	T	C	0.987	0.936	SERPING1
rs2508441	11	57,375,424	G	A	0.564	0.999	SERPING1
rs78624400	11	57,375,463	G	C	0.731	0.999	SERPING1
rs2454659	11	57,376,130	T	C	0.295	0.999	SERPING1
rs138770460	11	57,376,131	G	A	0.731	0.999	SERPING1
rs2508442	11	57,376,947	T	G	0.295	1	SERPING1
rs151035150	11	57,377,215	G	C	0.981	0.97	SERPING1
rs3824988	11	57,377,968	A	G	0.731	0.999	SERPING1
rs2508443	11	57,377,992	G	T	0.566	0.999	SERPING1
rs2454660	11	57,378,640	G	A	0.564	1	SERPING1
rs10896631	11	57,381,263	T	C	0.731	0.999	SERPING1
rs1557522	11	57,381,519	T	C	0.565	0.998	SERPING1
rs11570176	11	57,529,429	A	G	0.565	1	CTNND1
rs7120868	11	57,530,528	T	G	0.565	1	CTNND1
rs530094	11	57,531,037	G	A	0.766	1	CTNND1
rs77736623	11	57,531,159	A	G	0.985	0.974	CTNND1
rs117331991	11	57,532,160	G	A	0.959	0.989	CTNND1
rs80286487	11	57,533,140	A	G	0.989	0.964	CTNND1
rs72929820	11	57,533,764	A	G	0.987	0.922	CTNND1
rs12146692	11	57,534,190	A	G	0.884	0.979	CTNND1
rs11229116	11	57,534,542	C	G	0.672	1	CTNND1
rs17455422	11	57,534,799	C	T	0.564	1	CTNND1
rs113418884	11	57,535,617	T	TA	0.766	1	CTNND1
rs11229119	11	57,535,966	C	T	0.563	1	CTNND1
rs150371695	11	57,536,304	G	A	0.985	0.945	CTNND1
rs10792110	11	57,536,346	C	T	0.563	1	CTNND1
rs113152085	11	57,537,658	C	G	0.985	0.969	CTNND1
rs60449955	11	57,537,670	C	T	0.989	0.917	CTNND1
rs12270209	11	57,537,787	G	A	0.563	1	CTNND1
rs491416	11	57,538,140	T	G	0.987	0.983	CTNND1

SNP	Chromosome	Position	Effect Allele	Other Allele	Effect Allele Frequency	Imputation quality	Gene
rs140412383	11	57,538,894	T	TTATATAGAG	0.768	0.993	CTNND1
rs536394341	11	57,539,568	A	ATT	0.778	0.955	CTNND1
rs185235400	11	57,539,712	T	G	0.981	0.981	CTNND1
rs776166263	11	57,540,594	ATTAC	A	0.563	0.999	CTNND1
rs580223	11	57,541,120	T	A	0.766	1	CTNND1
rs374576333	11	57,542,804	G	GA	0.84	0.783	CTNND1
rs533962028	11	57,542,816	A	AC	0.988	0.749	CTNND1
rs147874424	11	57,542,820	A	AAC	0.67	0.997	CTNND1
rs2156638	11	57,542,998	G	C	0.563	1	CTNND1
rs17455626	11	57,544,484	T	C	0.564	1	CTNND1
rs541245	11	57,545,948	A	G	0.765	1	CTNND1
rs35808061	11	57,547,000	T	C	0.672	1	CTNND1
rs58673483	11	57,547,543	T	TTTTTG	0.172	0.92	CTNND1
rs186573301	11	57,548,633	A	G	0.985	0.997	CTNND1
rs141568292	11	57,549,608	T	C	0.985	0.911	CTNND1
rs185694221	11	57,549,941	C	T	0.989	0.969	CTNND1
rs12362406	11	57,550,785	A	G	0.761	0.991	CTNND1
rs112893917	11	57,550,919	C	A	0.986	0.956	CTNND1
rs753461944	11	57,551,881	C	CAA	0.711	0.803	CTNND1
rs4611222	11	57,552,265	G	A	0.563	1	CTNND1
rs576786	11	57,553,022	G	T	0.02	0.856	CTNND1
rs184449174	11	57,553,821	C	T	0.987	0.964	CTNND1
rs7127789	11	57,554,073	G	A	0.973	0.993	CTNND1
rs11570181	11	57,555,770	T	C	0.564	1	CTNND1
rs7126553	11	57,556,673	T	A	0.803	0.992	CTNND1
rs11570184	11	57,557,211	A	G	0.959	0.989	CTNND1
rs12274865	11	57,557,668	C	T	0.563	1	CTNND1
rs146423529	11	57,557,940	C	CCGCCTCCCTGATTTG	0.677	0.988	CTNND1
rs5792064	11	57,558,204	A	AT	0.765	1	CTNND1
11:57560304_AT_A	11	57,560,304	AT	A	0.058	0.814	CTNND1
rs11570190	11	57,560,452	A	C	0.672	1	CTNND1
11:57560772_CAG_C	11	57,560,772	CAG	C	0.67	0.996	CTNND1
rs501054	11	57,561,106	G	T	0.766	1	CTNND1
rs11229130	11	57,562,169	A	G	0.672	1	CTNND1
rs11229131	11	57,562,525	G	A	0.672	1	CTNND1
rs10896644	11	57,563,991	C	T	0.564	1	CTNND1
rs189950945	11	57,564,622	T	C	0.982	0.975	CTNND1
rs58132137	11	57,564,797	G	A	0.763	0.991	CTNND1
rs112976685	11	57,564,836	G	A	0.563	1	CTNND1
rs10792112	11	57,565,800	C	T	0.672	1	CTNND1
11:57565816_CA_C	11	57,565,816	CA	C	0.165	0.819	CTNND1
rs552528411	11	57,565,833	A	G	0.988	0.87	CTNND1
rs567017	11	57,566,327	A	T	0.765	1	CTNND1
rs79344679	11	57,570,228	G	A	0.969	0.993	CTNND1
rs653889	11	57,570,430	A	G	0.564	0.999	CTNND1
rs3781885	11	57,570,680	C	T	0.688	0.998	CTNND1
rs36105845	11	57,570,850	A	G	0.942	0.995	CTNND1
rs10896647	11	57,571,883	C	T	0.672	1	CTNND1
rs1268667	11	57,575,099	A	G	0.566	0.997	CTNND1
rs11570210	11	57,576,626	A	G	0.95	0.977	CTNND1
rs612688	11	57,577,096	A	G	0.765	1	CTNND1
rs11570214	11	57,578,377	C	T	0.977	0.981	CTNND1
rs581217	11	57,578,412	T	C	0.765	1	CTNND1
rs11570216	11	57,578,501	C	T	0.986	0.94	CTNND1
rs665058	11	57,579,166	T	C	0.563	1	CTNND1
rs11570219	11	57,579,588	G	C	0.943	0.976	CTNND1
rs117394301	11	57,580,009	C	G	0.978	0.943	CTNND1
rs658936	11	57,580,560	T	G	0.437	1	CTNND1
rs623541	11	57,581,613	C	A	0.437	1	CTNND1
rs708228	11	57,585,662	C	T	0.672	1	CTNND1
rs750371148	11	57,586,583	CAA	C	0.93	0.989	CTNND1
rs544021	11	66,315,661	C	G	0.574	0.997	ACTN3
rs597626	11	66,316,557	T	C	0.426	0.997	ACTN3
rs140613388	11	66,316,839	C	T	0.985	0.892	ACTN3
rs140376150	11	66,317,493	G	A	0.988	0.939	ACTN3
rs117533632	11	66,317,789	C	T	0.952	0.996	ACTN3
rs1791690	11	66,317,823	G	A	0.574	0.997	ACTN3
rs57127845	11	66,318,325	C	T	0.824	0.999	ACTN3
rs490998	11	66,318,972	G	A	0.574	0.997	ACTN3
rs531439359	11	66,319,184	C	T	0.989	0.961	ACTN3
rs484358	11	66,319,262	G	A	0.337	0.988	ACTN3
rs487444	11	66,319,313	A	G	0.397	0.997	ACTN3
rs192632292	11	66,319,707	G	A	0.986	0.968	ACTN3
11:66319840_CA_C	11	66,319,840	CA	C	0.573	0.986	ACTN3
rs574399913	11	66,320,260	G	A	0.982	0.533	ACTN3
rs2096583	11	66,320,353	A	T	0.907	0.679	ACTN3
rs4990284	11	66,320,355	A	T	0.629	0.749	ACTN3
rs647029	11	66,320,357	A	T	0.712	0.813	ACTN3
rs545522154	11	66,320,359	A	T	0.895	0.529	ACTN3
rs200389883	11	66,320,361	A	T	0.979	0.528	ACTN3
rs2511216	11	66,321,303	T	C	0.574	0.993	ACTN3
rs2511217	11	66,321,304	G	A	0.574	0.993	ACTN3
rs35121692	11	66,321,324	CA	C	0.818	0.734	ACTN3
rs574346914	11	66,321,927	C	CA	0.676	0.834	ACTN3
rs149608017	11	66,322,412	G	A	0.989	0.968	ACTN3
rs74802042	11	66,323,882	C	T	0.977	0.986	ACTN3
rs150633715	11	66,324,094	G	C	0.979	0.948	ACTN3
rs531490	11	66,324,360	T	G	0.458	1	ACTN3
rs679228	11	66,324,422	C	T	0.634	1	ACTN3
rs10896128	11	66,324,529	G	A	0.824	1	ACTN3
rs677488	11	66,324,762	C	T	0.458	1	ACTN3
rs2000939	11	66,325,830	A	G	0.458	1	ACTN3
rs145104915	11	66,326,248	G	A	0.986	0.944	ACTN3
rs2229455	11	66,328,055	A	G	0.814	1	ACTN3
rs3837428	11	66,328,494	G	GTTA	0.447	0.999	ACTN3
rs618838	11	66,328,719	T	C	0.447	1	ACTN3
rs2290463	11	66,328,852	G	C	0.93	0.999	ACTN3
rs607736	11	66,328,877	G	A	0.704	1	ACTN3
rs5742714	12	102,789,852	C	G	0.91	0.995	IGF1
rs6222	12	102,789,901	G	A	0.977	0.991	IGF1
rs6219	12	102,790,192	C	T	0.91	0.995	IGF1
rs530631102	12	102,790,566	C	CT	0.978	0.861	IGF1
rs1140655	12	102,790,780	A	T	0.301	0.991	IGF1
12:102791800_TA_T	12	102,791,800	TA	T	0.978	0.975	IGF1
rs117058953	12	102,792,733	G	A	0.984	0.951	IGF1
rs5742705	12	102,792,897	G	A	0.977	0.991	IGF1
rs55650342	12	102,793,059	G	C	0.974	0.968	IGF1
rs6214	12	102,793,569	C	T	0.578	0.989	IGF1
rs5742704	12	102,793,921	T	C	0.977	0.991	IGF1
rs6220	12	102,794,515	G	A	0.281	0.996	IGF1
rs3730204	12	102,795,514	A	G	0.985	0.961	IGF1
rs70961704	12	102,796,022	A	T	0.97	0.896	IGF1
rs3032449	12	102,796,024	T	A	0.862	0.858	IGF1
rs1520220	12	102,796,522	G	C	0.17	0.996	IGF1
rs1549593	12	102,796,791	G	T	0.852	0.978	IGF1
rs1520221	12	102,796,843	G	A	0.99	0.895	IGF1
rs5742696	12	102,797,092	C	G	0.973	0.983	IGF1
rs10745940	12	102,797,196	A	G	0.281	0.996	IGF1
rs80280982	12	102,797,230	G	A	0.931	0.981	IGF1
rs1520222	12	102,797,293	G	A	0.259	0.997	IGF1
rs11111262	12	102,798,177	G	A	0.91	0.995	IGF1
rs5800510	12	102,798,541	G	GT	0.082	0.841	IGF1
rs34097393	12	102,798,854	G	GCTTT	0.559	0.974	IGF1
rs5742694	12	102,799,236	C	A	0.244	0.997	IGF1
rs12316064	12	102,800,803	C	T	0.704	0.987	IGF1
rs6539035	12	102,800,935	C	T	0.244	0.997	IGF1
rs978458	12	102,802,239	T	C	0.259	0.998	IGF1
rs17884734	12	102,802,385	C	T	0.977	0.99	IGF1
rs17885209	12	102,803,398	A	G	0.976	0.99	IGF1
rs9308315	12	102,803,893	A	T	0.259	0.997	IGF1
rs17885564	12	102,804,319	C	A	0.986	0.997	IGF1
rs78668884	12	102,806,183	C	G	0.976	0.993	IGF1
rs4764883	12	102,806,305	C	T	0.283	0.997	IGF1
rs17882373	12	102,807,670	G	A	0.985	0.996	IGF1
rs17878318	12	102,808,355	A	C	0.985	0.997	IGF1
rs2195242	12	102,808,464	A	G	0.258	0.998	IGF1
12:102809034_TCATC_T	12	102,809,034	TCATC	T	0.099	0.942	IGF1
rs775575484	12	102,809,038	C	T	0.971	0.531	IGF1
rs138390877	12	102,809,154	T	TA	0.985	0.997	IGF1
rs17727841	12	102,809,630	G	C	0.821	0.997	IGF1
rs5009837	12	102,810,269	T	C	0.281	0.997	IGF1
rs4764696	12	102,810,398	T	C	0.243	0.997	IGF1
rs10860863	12	102,810,596	C	T	0.062	0.995	IGF1
rs113221407	12	102,810,997	T	TGAA	0.244	0.995	IGF1
rs11111267	12	102,811,239	A	G	0.821	0.997	IGF1
rs5742688	12	102,812,526	G	A	0.979	0.963	IGF1
rs2072592	12	102,813,632	C	T	0.985	0.998	IGF1
rs5742678	12	102,814,332	C	G	0.259	0.998	IGF1
rs5742671	12	102,815,758	G	A	0.82	0.997	IGF1
rs5742669	12	102,815,800	C	CA	0.985	0.998	IGF1
rs771782568	12	102,816,479	GAT	G	0.258	0.996	IGF1
rs557562964	12	102,817,071	G	A	0.986	0.996	IGF1
12:102818017_CT_C	12	102,818,017	CT	C	0.767	0.816	IGF1
rs140619447	12	102,818,081	A	G	0.981	0.902	IGF1
rs17878716	12	102,819,256	C	A	0.983	0.94	IGF1

SNP	Chromosome	Position	Effect Allele	Other Allele	Effect Allele Frequency	Imputation quality	Gene
rs79858768	12	102,819,325	A	T	0.977	0.991	IGF1
rs4764884	12	102,819,599	T	C	0.244	0.998	IGF1
rs6539036	12	102,821,155	C	A	0.244	0.998	IGF1
rs4764885	12	102,822,528	A	G	0.282	0.997	IGF1
rs10860864	12	102,822,862	C	T	0.82	0.998	IGF1
rs5742667	12	102,823,478	A	G	0.259	0.998	IGF1
rs5742666	12	102,823,519	C	T	0.244	0.998	IGF1
rs5742665	12	102,823,550	C	G	0.859	0.978	IGF1
rs4764886	12	102,823,712	T	C	0.985	0.999	IGF1
rs972936	12	102,824,921	T	C	0.259	0.998	IGF1
rs75930213	12	102,826,523	G	A	0.976	0.99	IGF1
rs143463116	12	102,826,528	C	G	0.986	0.998	IGF1
rs542994694	12	102,826,625	A	AATAC	0.972	0.988	IGF1
12:102826653_CATAA_C	12	102,826,653	CATAA	C	0.956	0.856	IGF1
rs2373721	12	102,827,045	G	C	0.244	0.998	IGF1
rs11111272	12	102,827,441	G	C	0.283	0.998	IGF1
rs549090422	12	102,827,924	C	CTCT	0.965	0.624	IGF1
rs12833855	12	102,827,926	T	C	0.423	0.973	IGF1
rs181969742	12	102,828,076	C	A	0.959	0.95	IGF1
rs186537413	12	102,828,079	C	T	0.959	0.951	IGF1
rs141251113	12	102,828,131	T	C	0.985	0.998	IGF1
rs2288378	12	102,830,008	T	C	0.243	0.999	IGF1
rs17881922	12	102,830,532	G	A	0.977	0.991	IGF1
rs10860865	12	102,831,722	T	G	0.259	0.999	IGF1
rs73180982	12	102,832,231	A	G	0.972	0.994	IGF1
rs35403470	12	102,833,309	G	A	0.831	0.98	IGF1
rs140686082	12	102,833,372	C	T	0.977	0.991	IGF1
rs375877793	12	102,833,496	G	GA	0.39	0.846	IGF1
rs7296464	12	102,834,010	G	A	0.244	0.999	IGF1
rs5742658	12	102,834,624	T	G	0.976	0.99	IGF1
rs5742657	12	102,834,868	A	G	0.985	0.998	IGF1
rs5742656	12	102,834,902	G	A	0.987	0.987	IGF1
rs529196905	12	102,835,681	A	G	0.985	0.728	IGF1
rs10860866	12	102,835,687	T	G	0.557	0.726	IGF1
rs10745941	12	102,835,693	C	T	0.238	0.983	IGF1
rs12810139	12	102,835,736	T	C	0.543	0.808	IGF1
rs5742653	12	102,835,859	C	T	0.259	0.999	IGF1
rs5742652	12	102,836,066	C	T	0.977	0.991	IGF1
rs5742649	12	102,837,180	G	A	0.978	0.971	IGF1
rs5742643	12	102,837,863	T	C	0.245	0.999	IGF1
rs28682289	12	102,837,969	G	A	0.977	0.991	IGF1
rs149329383	12	102,838,224	C	A	0.985	0.966	IGF1
rs7136446	12	102,838,515	C	T	0.415	0.987	IGF1
rs17882108	12	102,838,899	G	A	0.977	0.991	IGF1
rs10778174	12	102,838,996	A	G	0.244	0.999	IGF1
rs10778175	12	102,839,114	A	C	0.243	0.999	IGF1
rs12425939	12	102,840,175	C	G	0.985	0.998	IGF1
rs2373722	12	102,840,457	A	G	0.064	0.996	IGF1
rs12426645	12	102,841,811	G	A	0.985	0.997	IGF1
rs10860867	12	102,841,826	C	T	0.985	0.997	IGF1
rs56704014	12	102,841,883	G	GT	0.064	0.994	IGF1
rs763757711	12	102,841,885	C	CC	0.854	0.558	IGF1
rs183979209	12	102,842,218	C	T	0.985	0.995	IGF1
rs193083548	12	102,842,384	G	A	0.975	0.987	IGF1
rs79910493	12	102,843,754	C	T	0.977	0.993	IGF1
rs10735380	12	102,844,236	G	A	0.27	0.998	IGF1
rs9651925	12	102,845,544	G	T	0.27	0.997	IGF1
rs17884646	12	102,845,815	C	T	0.905	0.965	IGF1
rs11111278	12	102,846,020	A	G	0.793	0.995	IGF1
rs538007224	12	102,846,039	C	CAAAA	0.49	0.839	IGF1
rs12313279	12	102,846,504	A	G	0.288	0.997	IGF1
rs2033178	12	102,847,076	A	G	0.064	0.995	IGF1
rs5742639	12	102,848,246	G	A	0.985	0.996	IGF1
rs17728444	12	102,849,913	T	A	0.981	0.974	IGF1
rs9989002	12	102,850,223	A	G	0.273	0.995	IGF1
rs79519967	12	102,850,462	T	G	0.989	0.912	IGF1
rs187077464	12	102,850,718	G	C	0.989	0.852	IGF1
rs117520647	12	102,850,975	A	T	0.978	0.963	IGF1
rs143254896	12	102,852,390	C	G	0.975	0.952	IGF1
rs577236052	12	102,852,508	C	CAAAAAAAAAA	0.296	0.973	IGF1
rs78974397	12	102,852,617	T	C	0.967	0.995	IGF1
rs4764697	12	102,853,172	T	C	0.282	0.995	IGF1
rs11831436	12	102,853,243	C	T	0.967	0.995	IGF1
rs149948658	12	102,854,224	T	TC	0.968	0.994	IGF1
rs79711124	12	102,854,243	C	T	0.987	0.99	IGF1
rs7971494	12	102,854,267	C	A	0.969	0.994	IGF1
rs77110635	12	102,854,349	T	C	0.967	0.995	IGF1
rs79675546	12	102,855,511	A	G	0.967	0.996	IGF1
rs61935423	12	102,855,852	A	C	0.877	0.959	IGF1
rs118057318	12	102,856,414	T	A	0.976	0.979	IGF1
rs5742632	12	102,856,474	A	G	0.766	1	IGF1
rs2195239	12	102,856,702	C	G	0.766	1	IGF1
rs141454352	12	102,857,016	G	A	0.984	0.901	IGF1
rs5742629	12	102,857,263	T	C	0.733	0.999	IGF1
12:102858088_CT_C	12	102,858,088	CT	C	0.78	0.998	IGF1
rs7956547	12	102,858,816	T	C	0.747	0.998	IGF1
rs12423791	12	102,858,828	G	C	0.986	0.995	IGF1
rs747735019	12	102,859,649	AGGTTGCAGTGAGCCG	A	0.82	0.965	IGF1
12:102859714_CAAAT_C	12	102,859,714	CAAAT	C	0.116	0.916	IGF1
rs4764698	12	102,860,060	C	G	0.986	0.996	IGF1
rs4764699	12	102,860,088	G	C	0.986	0.996	IGF1
rs73181000	12	102,861,183	G	A	0.972	0.994	IGF1
rs74658525	12	102,861,764	C	T	0.986	0.996	IGF1
rs17032623	12	102,861,810	A	C	0.986	0.996	IGF1
rs79838442	12	102,862,351	A	G	0.967	0.998	IGF1
rs10778176	12	102,862,979	C	T	0.735	0.998	IGF1
rs17884900	12	102,863,596	T	C	0.985	0.969	IGF1
rs17882461	12	102,863,979	A	T	0.779	0.998	IGF1
rs10860869	12	102,865,052	A	T	0.732	0.997	IGF1
rs17796225	12	102,866,638	T	C	0.745	0.996	IGF1
rs78611929	12	102,867,198	A	G	0.967	0.997	IGF1
rs10745942	12	102,867,636	A	C	0.067	0.988	IGF1
rs12821878	12	102,867,667	G	A	0.77	0.971	IGF1
rs5742620	12	102,869,600	G	T	0.969	0.993	IGF1
rs5742615	12	102,870,801	G	A	0.976	0.961	IGF1
rs2162679	12	102,871,259	C	T	0.15	0.991	IGF1
rs371700453	12	102,871,313	A	AT	0.392	0.86	IGF1
rs17882264	12	102,871,356	T	C	0.917	0.965	IGF1
rs11831702	12	102,871,693	A	G	0.978	0.989	IGF1
12:102871774_TC_T	12	102,871,774	TC	T	0.859	0.536	IGF1
rs761290155	12	102,871,775	CT	C	0.076	0.808	IGF1
rs67214112	12	102,872,155	C	CT	0.152	0.896	IGF1
rs8546	12	124,809,027	G	A	0.791	0.992	NCOR2
rs1055878	12	124,809,032	C	T	0.966	0.984	NCOR2
rs1055941	12	124,809,459	G	A	0.91	0.997	NCOR2
rs113021933	12	124,809,541	C	T	0.984	0.972	NCOR2
rs77571124	12	124,809,879	T	C	0.883	0.982	NCOR2
rs368678225	12	124,809,925	C	CCG	0.101	0.888	NCOR2
rs10846657	12	124,810,174	G	A	0.368	0.993	NCOR2
rs11057583	12	124,810,329	G	A	0.893	0.997	NCOR2
rs1006100	12	124,810,968	C	T	0.209	0.991	NCOR2
rs77428605	12	124,811,041	A	G	0.981	0.986	NCOR2
12:124811393_ACT_A	12	124,811,393	ACT	A	0.893	0.995	NCOR2
rs2272368	12	124,811,855	A	G	0.874	0.995	NCOR2
rs59475273	12	124,812,574	T	A	0.969	0.985	NCOR2
rs3741508	12	124,812,678	T	G	0.874	0.995	NCOR2
rs140431269	12	124,812,706	G	A	0.964	0.99	NCOR2
rs78310648	12	124,812,842	G	T	0.984	0.987	NCOR2
rs58543684	12	124,813,107	T	TG	0.968	0.986	NCOR2
rs150344768	12	124,813,126	C	T	0.984	0.985	NCOR2
rs75977598	12	124,813,198	T	C	0.98	0.986	NCOR2
rs74643464	12	124,813,316	C	T	0.981	0.987	NCOR2
rs117604817	12	124,813,583	C	T	0.985	0.793	NCOR2
rs36018719	12	124,813,893	G	A	0.969	0.951	NCOR2
rs61161987	12	124,814,142	C	G	0.981	0.986	NCOR2
rs117368827	12	124,814,655	C	G	0.988	0.796	NCOR2
rs12367963	12	124,815,504	C	T	0.91	0.993	NCOR2
rs112395349	12	124,815,717	T	A	0.968	0.992	NCOR2
rs73223563	12	124,816,013	T	C	0.91	0.993	NCOR2
rs1244099	12	124,816,131	T	G	0.216	0.991	NCOR2
rs5801546	12	124,816,165	G	GT	0.217	0.985	NCOR2
rs117877142	12	124,816,266	T	C	0.968	0.992	NCOR2
rs11057586	12	124,816,575	G	A	0.983	0.984	NCOR2
rs5801547	12	124,818,205	A	AC	0.91	0.862	NCOR2
rs28639958	12	124,818,473	T	C	0.983	0.948	NCOR2
rs4765545	12	124,819,460	C	A	0.957	0.998	NCOR2
rs4765547	12	124,819,855	G	T	0.957	0.998	NCOR2
rs1106317	12	124,820,013	A	G	0.789	0.995	NCOR2
rs1271309	12	124,820,705	A	G	0.164	0.994	NCOR2
rs73223564	12	124,820,896	G	A	0.985	0.74	NCOR2
rs2230944	12	124,821,413	G	A	0.958	0.998	NCOR2
rs2227277	12	124,821,416	C	T	0.958	0.998	NCOR2
rs61751354	12	124,821,666	G	A	0.984	0.988	NCOR2
rs2229842	12	124,821,696	C	T	0.852	0.997	NCOR2
rs146138051	12	124,822,006	G	A	0.985	0.986	NCOR2

SNP	Chromosome	Position	Effect Allele	Other Allele	Effect Allele Frequency	Imputation quality	Gene
12:124822318_AGCTGGGGCACAGCTGCCCCTGCACAGG_A	12	124,822,318	AGCTGGGGCACAGCTGCCCCTGCACAGG	A	0.983	0.94	NCOR2
rs7138007	12	124,822,463	C	T	0.844	0.999	NCOR2
rs150940851	12	124,822,464	G	A	0.987	0.959	NCOR2
rs111766704	12	124,822,881	G	A	0.841	0.998	NCOR2
rs73419839	12	124,822,935	C	T	0.783	0.998	NCOR2
rs61051553	12	124,823,437	A	AACCC	0.861	0.927	NCOR2
rs114910742	12	124,823,562	A	C	0.985	0.98	NCOR2
12:124823672_ACCAC_A	12	124,823,672	ACCAC	A	0.046	0.952	NCOR2
12:124823684_CCCAT_C	12	124,823,684	CCCAT	C	0.13	0.65	NCOR2
rs56117563	12	124,823,765	A	T	0.682	0.948	NCOR2
rs56374933	12	124,823,766	C	A	0.682	0.948	NCOR2
rs534541609	12	124,823,911	C	CACCT	0.741	0.973	NCOR2
rs79887820	12	124,824,131	T	C	0.988	0.927	NCOR2
rs67723817	12	124,824,159	T	C	0.407	0.879	NCOR2
rs61934021	12	124,824,265	G	A	0.63	0.759	NCOR2
rs61934022	12	124,824,275	T	C	0.588	0.768	NCOR2
12:124824409_CAT_C	12	124,824,409	CAT	C	0.952	0.934	NCOR2
rs2660386	12	124,824,430	C	G	0.38	0.987	NCOR2
rs746383152	12	124,824,721	C	CGCCGCTGCT	0.026	0.614	NCOR2
rs1794973	12	124,825,592	C	T	0.407	0.989	NCOR2
rs116242084	12	124,825,823	T	C	0.985	0.983	NCOR2
rs1702333	12	124,825,924	T	C	0.287	0.992	NCOR2
rs11057587	12	124,826,019	G	C	0.933	0.989	NCOR2
rs11057588	12	124,826,021	T	A	0.933	0.989	NCOR2
rs10846658	12	124,826,036	C	A	0.714	0.992	NCOR2
rs2230942	12	124,826,598	T	C	0.933	0.989	NCOR2
rs58918660	12	124,826,947	G	C	0.933	0.989	NCOR2
rs59494544	12	124,827,149	T	C	0.933	0.989	NCOR2
rs117216700	12	124,827,209	C	G	0.953	0.841	NCOR2
rs73419849	12	124,827,316	G	A	0.933	0.988	NCOR2
rs1263993	12	124,828,059	C	T	0.396	0.989	NCOR2
rs150894060	12	124,828,918	C	T	0.97	0.953	NCOR2
rs1263992	12	124,829,234	C	T	0.286	0.989	NCOR2
rs1244085	12	124,829,255	A	G	0.287	0.987	NCOR2
rs75788176	12	124,830,446	T	C	0.948	0.963	NCOR2
rs141297356	12	124,830,507	G	A	0.987	0.817	NCOR2
12:124830529_GCTGGGGCCGCCTGGGGGA_G	12	124,830,529	GCTGGGGCCGCCTGGGGGA	G	0.949	0.953	NCOR2
rs1881074	12	124,830,864	A	C	0.157	0.994	NCOR2
rs2272371	12	124,831,018	G	T	0.715	0.993	NCOR2
rs11370026	12	124,831,062	C	CG	0.849	0.983	NCOR2
rs2272370	12	124,831,067	C	A	0.715	0.993	NCOR2
rs1244066	12	124,831,596	G	A	0.442	0.991	NCOR2
rs1244065	12	124,832,474	G	A	0.157	0.99	NCOR2
rs1244063	12	124,832,606	A	G	0.146	0.984	NCOR2
rs2272369	12	124,833,073	G	C	0.716	0.995	NCOR2
rs1244061	12	124,833,303	A	G	0.429	0.988	NCOR2
rs3782244	12	124,833,385	G	A	0.713	0.994	NCOR2
rs150523238	12	124,833,418	G	A	0.982	0.706	NCOR2
rs3782245	12	124,833,568	T	A	0.715	0.996	NCOR2
rs3782246	12	124,833,816	C	T	0.802	0.988	NCOR2
rs113974966	12	124,834,009	C	T	0.956	0.956	NCOR2
rs3782247	12	124,834,040	T	C	0.681	0.983	NCOR2
rs3782248	12	124,834,042	A	G	0.564	0.986	NCOR2
rs181055707	12	124,834,794	C	T	0.987	0.925	NCOR2
rs1244060	12	124,834,795	A	G	0.426	0.987	NCOR2
rs4765551	12	124,835,352	A	C	0.716	0.997	NCOR2
rs12825956	12	124,835,391	C	T	0.984	0.909	NCOR2
rs61934025	12	124,835,827	A	C	0.716	0.998	NCOR2
12:124835930_TG_T	12	124,835,930	TG	T	0.715	0.994	NCOR2
rs61934026	12	124,835,950	T	C	0.71	0.997	NCOR2
rs58700298	12	124,836,110	T	C	0.716	0.999	NCOR2
rs58463871	12	124,836,115	T	A	0.733	0.995	NCOR2
rs4765552	12	124,836,549	C	T	0.711	0.998	NCOR2
rs4765553	12	124,836,554	G	A	0.716	0.999	NCOR2
rs4765143	12	124,836,778	C	T	0.711	0.998	NCOR2
rs56065860	12	124,836,974	C	T	0.71	0.995	NCOR2
12:124836981_CTG_C	12	124,836,981	CTG	C	0.713	0.99	NCOR2
rs3782249	12	124,837,943	A	C	0.716	1	NCOR2
rs3782251	12	124,838,092	T	C	0.716	1	NCOR2
rs3782252	12	124,838,099	T	C	0.716	1	NCOR2
rs3782253	12	124,838,175	C	T	0.716	1	NCOR2
rs4765554	12	124,838,417	C	G	0.716	0.999	NCOR2
rs4765555	12	124,838,591	C	G	0.716	0.999	NCOR2
rs117746471	12	124,838,619	C	A	0.966	0.836	NCOR2
rs3817108	12	124,838,774	T	A	0.716	0.999	NCOR2
rs2271142	12	124,839,150	G	A	0.716	0.998	NCOR2
rs2271141	12	124,839,165	G	A	0.716	0.998	NCOR2
rs2271140	12	124,839,317	C	T	0.987	0.886	NCOR2
rs2271139	12	124,839,540	C	A	0.716	0.998	NCOR2
rs150082761	12	124,839,553	C	T	0.988	0.886	NCOR2
rs870050	12	124,839,867	A	G	0.291	0.996	NCOR2
rs870051	12	124,839,899	T	C	0.714	0.997	NCOR2
rs870052	12	124,839,945	T	C	0.715	0.997	NCOR2
rs4765144	12	124,841,421	C	T	0.716	0.997	NCOR2
rs75548337	12	124,841,777	C	T	0.963	0.95	NCOR2
rs73223579	12	124,842,324	G	C	0.803	0.983	NCOR2
rs57170960	12	124,842,449	A	ATG	0.556	0.931	NCOR2
rs144126565	12	124,842,631	A	ATG	0.383	0.79	NCOR2
rs9971647	12	124,842,642	C	T	0.595	0.767	NCOR2
rs77581721	12	124,842,643	G	A	0.426	0.797	NCOR2
rs559913758	12	124,842,647	G	A	0.964	0.752	NCOR2
12:124842660_CGT_C	12	124,842,660	CGT	C	0.987	0.763	NCOR2
rs1244076	12	124,842,841	G	A	0.914	0.842	NCOR2
rs764703523	12	124,842,900	TGAA	T	0.962	0.926	NCOR2
rs1244074	12	124,842,947	T	C	0.279	0.985	NCOR2
rs191011028	12	124,842,949	C	T	0.989	0.806	NCOR2
12:124843326_CATT_C	12	124,843,326	CATT	C	0.776	0.948	NCOR2
rs147508641	12	124,843,851	C	T	0.972	0.847	NCOR2
rs1244071	12	124,843,895	A	C	0.74	0.649	NCOR2
rs11057590	12	124,843,999	A	G	0.934	0.717	NCOR2
rs111404346	12	124,844,053	G	A	0.977	0.781	NCOR2
rs7975419	12	124,844,581	G	A	0.295	0.863	NCOR2
rs7978718	12	124,844,954	G	A	0.948	0.872	NCOR2
rs34457329	12	124,845,148	G	A	0.989	0.947	NCOR2
rs1244069	12	124,845,280	C	G	0.153	0.987	NCOR2
rs35361917	12	124,845,369	G	GC	0.154	0.981	NCOR2
rs1268892	12	124,845,413	T	G	0.956	0.99	NCOR2
rs1263990	12	124,845,436	C	T	0.19	0.965	NCOR2
rs7980316	12	124,845,590	C	A	0.157	0.987	NCOR2
rs75787667	12	124,845,632	C	T	0.857	0.981	NCOR2
12:124845668_CAA_C	12	124,845,668	CAA	C	0.176	0.817	NCOR2
rs11057591	12	124,845,698	G	C	0.201	0.981	NCOR2
rs35480942	12	124,846,379	T	TAAG	0.156	0.986	NCOR2
rs1244067	12	124,846,561	C	T	0.938	0.919	NCOR2
rs2660373	12	124,847,088	C	G	0.155	0.994	NCOR2
rs116969278	12	124,847,415	C	T	0.982	0.886	NCOR2
rs77406635	12	124,847,957	T	C	0.852	0.998	NCOR2
rs75164514	12	124,848,482	T	G	0.856	0.989	NCOR2
rs1702316	12	124,848,559	G	A	0.811	0.99	NCOR2
rs1794957	12	124,848,572	A	G	0.155	0.998	NCOR2
rs11057595	12	124,849,097	C	T	0.957	0.985	NCOR2
rs116940446	12	124,849,233	C	T	0.987	0.943	NCOR2
rs11837649	12	124,850,168	T	C	0.852	0.999	NCOR2
rs112086432	12	124,850,404	C	T	0.974	0.741	NCOR2
rs906302	12	124,850,457	C	G	0.961	0.989	NCOR2
rs2048108	12	124,850,666	T	C	0.506	0.972	NCOR2
rs2141247	12	124,850,753	C	A	0.387	0.995	NCOR2
rs2048107	12	124,850,796	A	G	0.196	0.993	NCOR2
rs2178082	12	124,851,129	T	G	0.344	0.995	NCOR2
rs11057600	12	124,851,142	C	T	0.391	0.994	NCOR2
rs73223592	12	124,851,259	T	C	0.852	0.999	NCOR2
rs1881071	12	124,851,420	G	T	0.198	0.997	NCOR2
rs778155222	12	124,851,821	GGCACACACAGAGGCCCA	G	0.853	0.994	NCOR2
rs368108497	12	124,851,839	A	T	0.852	0.997	NCOR2
rs1794959	12	124,852,314	G	A	0.96	0.991	NCOR2
rs7976285	12	124,852,461	T	C	0.809	0.997	NCOR2
rs11057601	12	124,852,564	A	G	0.409	0.994	NCOR2
rs76330997	12	124,852,819	G	T	0.853	0.996	NCOR2
rs11835010	12	124,852,825	A	G	0.81	0.997	NCOR2
rs11835564	12	124,852,843	T	C	0.809	0.996	NCOR2
rs10773085	12	124,853,051	T	C	0.198	0.998	NCOR2
rs77078314	12	124,853,158	T	C	0.856	0.998	NCOR2
rs12369200	12	124,853,416	C	T	0.961	0.996	NCOR2
rs73223595	12	124,853,700	A	T	0.852	0.999	NCOR2
rs3782257	12	124,854,903	G	A	0.389	0.999	NCOR2
rs142513345	12	124,855,089	T	C	0.979	0.931	NCOR2
rs1881072	12	124,855,223	C	T	0.856	0.999	NCOR2
rs80015478	12	124,855,274	G	A	0.856	0.999	NCOR2
rs112965534	12	124,855,881	C	G	0.987	0.811	NCOR2
rs79957340	12	124,855,975	G	A	0.856	0.999	NCOR2
rs77017033	12	124,856,025	T	C	0.855	0.999	NCOR2
rs1108109	12	124,856,332	T	C	0.659	0.998	NCOR2
rs7961196	12	124,856,618	A	G	0.665	0.998	NCOR2
rs3741513	12	124,856,660	T	A	0.39	0.998	NCOR2
rs142292731	12	124,856,830	C	T	0.97	0.959	NCOR2

SNP	Chromosome	Position	Effect Allele	Other Allele	Effect Allele Frequency	Imputation quality	Gene
rs7978237	12	124,857,033	C	T	0.855	0.998	NCOR2
rs1109271	12	124,857,199	G	T	0.85	0.994	NCOR2
rs4765147	12	124,857,274	G	A	0.467	0.984	NCOR2
rs1109270	12	124,857,293	G	A	0.952	0.996	NCOR2
rs11057604	12	124,857,763	G	A	0.39	0.998	NCOR2
rs12832637	12	124,857,795	C	A	0.956	0.994	NCOR2
rs12830830	12	124,857,797	G	A	0.956	0.994	NCOR2
rs28432783	12	124,857,823	T	C	0.393	0.99	NCOR2
rs61931987	12	124,857,838	T	C	0.392	0.99	NCOR2
rs112279878	12	124,857,935	C	A	0.473	0.899	NCOR2
rs2950603	12	124,858,163	G	A	0.956	0.629	NCOR2
rs1702326	12	124,858,183	G	A	0.961	0.926	NCOR2
12:124858345_CAT_C	12	124,858,345	CAT	C	0.914	0.839	NCOR2
rs2950604	12	124,858,362	T	C	0.352	0.802	NCOR2
rs2950605	12	124,858,373	T	C	0.086	0.95	NCOR2
rs2939984	12	124,858,415	C	A	0.575	0.74	NCOR2
rs2660377	12	124,858,457	C	A	0.882	0.734	NCOR2
rs11057606	12	124,859,135	G	A	0.437	0.997	NCOR2
rs10773086	12	124,859,244	G	T	0.906	0.998	NCOR2
rs75375965	12	124,859,904	A	C	0.855	0.997	NCOR2
rs144356706	12	124,859,998	C	A	0.979	0.93	NCOR2
rs10846663	12	124,860,830	C	T	0.246	0.997	NCOR2
12:124861071_TCCTTCCATCAGA_T	12	124,861,071	TCCTTCCATCAGA	T	0.982	0.921	NCOR2
rs11837407	12	124,861,223	A	G	0.436	0.998	NCOR2
rs7963151	12	124,861,726	T	C	0.855	0.996	NCOR2
rs12367444	12	124,861,864	G	A	0.437	0.997	NCOR2
rs4765558	12	124,862,112	T	C	0.246	0.996	NCOR2
rs12830424	12	124,862,531	A	G	0.957	0.996	NCOR2
rs1244050	12	124,862,697	G	A	0.959	0.985	NCOR2
rs3741515	12	124,862,769	G	T	0.957	0.993	NCOR2
rs118148371	12	124,863,070	C	T	0.975	0.932	NCOR2
rs11057607	12	124,863,195	G	C	0.436	0.997	NCOR2
rs112634030	12	124,863,548	C	T	0.98	0.975	NCOR2
rs7970925	12	124,863,550	T	C	0.874	0.561	NCOR2
rs7967845	12	124,863,560	A	G	0.425	0.974	NCOR2
rs55925172	12	124,863,563	A	ATGGGACAAGGAGCACAAGGGCAGCATCAGAGGGTCGCGCGGGTGAAACGGGG	0.812	0.99	NCOR2
rs7953314	12	124,863,578	C	A	0.853	0.858	NCOR2
rs7953329	12	124,863,599	C	T	0.811	0.995	NCOR2
rs7980860	12	124,863,657	G	A	0.81	0.996	NCOR2
rs61931988	12	124,863,847	T	C	0.441	0.997	NCOR2
rs117136387	12	124,863,998	G	A	0.987	0.921	NCOR2
rs144214817	12	124,865,361	C	T	0.989	0.925	NCOR2
rs2135758	12	124,865,792	T	C	0.81	0.994	NCOR2
rs1269481	12	124,866,324	C	A	0.749	0.994	NCOR2
rs12369386	12	124,866,338	G	A	0.81	0.994	NCOR2
rs139546800	12	124,867,383	A	C	0.983	0.99	NCOR2
rs873206	12	124,867,454	C	G	0.441	0.999	NCOR2
rs542754148	12	124,867,583	G	GATGA	0.932	0.953	NCOR2
rs2880348	12	124,867,774	G	A	0.529	0.938	NCOR2
rs1268891	12	124,867,996	G	T	0.749	0.993	NCOR2
12:124868079_CA_C	12	124,868,079	CA	C	0.789	0.758	NCOR2
rs12370377	12	124,868,410	G	A	0.81	0.986	NCOR2
rs12370388	12	124,868,485	G	A	0.853	0.986	NCOR2
rs867429	12	124,868,778	C	T	0.416	0.924	NCOR2
rs906299	12	124,869,817	C	T	0.788	0.911	NCOR2
rs906300	12	124,869,933	C	G	0.78	0.897	NCOR2
rs55861018	12	124,870,239	G	A	0.958	0.846	NCOR2
rs75988694	12	124,870,715	G	A	0.963	0.92	NCOR2
rs1542975	12	124,870,737	C	T	0.838	0.957	NCOR2
rs11838138	12	124,871,329	C	A	0.916	0.965	NCOR2
rs12366929	12	124,872,883	C	G	0.598	0.951	NCOR2
rs114890969	12	124,872,886	C	T	0.916	0.971	NCOR2
rs118132240	12	124,873,074	G	A	0.971	0.985	NCOR2
rs7960906	12	124,873,079	T	G	0.643	0.896	NCOR2
12:124873411_TGAGG_T	12	124,873,411	TGAGG	T	0.145	0.741	NCOR2
rs191752208	12	124,873,427	G	C	0.965	0.987	NCOR2
rs12819167	12	124,873,633	G	C	0.759	0.979	NCOR2
rs78624214	12	124,874,453	T	G	0.986	0.95	NCOR2
rs4622357	12	124,874,823	C	T	0.913	0.961	NCOR2
rs11057609	12	124,875,279	G	A	0.851	0.951	NCOR2
rs1876382	12	124,875,298	A	G	0.726	0.981	NCOR2
rs12708369	12	124,875,577	C	T	0.603	0.968	NCOR2
rs11057610	12	124,875,605	G	A	0.939	0.976	NCOR2
rs1532944	12	124,875,657	T	C	0.492	0.961	NCOR2
12:124876048_CG_C	12	124,876,048	CG	C	0.161	0.781	NCOR2
rs7955415	12	124,876,198	C	G	0.977	0.975	NCOR2
rs12423923	12	124,876,509	C	T	0.755	0.994	NCOR2
rs67417817	12	124,877,186	G	A	0.577	0.963	NCOR2
rs139320351	12	124,877,284	C	CA	0.823	0.921	NCOR2
rs951976	12	124,877,610	A	G	0.823	0.949	NCOR2
rs951977	12	124,877,620	C	G	0.974	0.93	NCOR2
rs60861600	12	124,878,105	C	T	0.755	0.981	NCOR2
rs73225415	12	124,878,632	C	T	0.851	0.955	NCOR2
rs141154403	12	124,878,993	T	TC	0.909	0.95	NCOR2
rs138631560	12	124,879,232	G	A	0.974	0.922	NCOR2
rs1702335	12	124,879,836	G	A	0.967	0.962	NCOR2
rs201543862	12	124,880,313	T	G	0.975	0.788	NCOR2
rs1244084	12	124,881,294	G	A	0.308	0.78	NCOR2
rs139046109	12	124,881,297	G	A	0.985	0.911	NCOR2
rs56951720	12	124,881,576	C	A	0.945	0.935	NCOR2
rs118041883	12	124,881,600	G	A	0.977	0.938	NCOR2
rs61932026	12	124,881,752	G	A	0.838	0.959	NCOR2
rs12317633	12	124,881,782	G	C	0.522	0.959	NCOR2
rs143129326	12	124,881,977	G	A	0.941	0.974	NCOR2
rs75056841	12	124,882,604	G	A	0.97	0.835	NCOR2
rs117510453	12	124,882,834	G	A	0.915	0.965	NCOR2
rs12321007	12	124,883,101	G	A	0.667	0.879	NCOR2
rs11057612	12	124,883,105	T	A	0.856	0.997	NCOR2
rs3782260	12	124,883,680	G	A	0.856	0.995	NCOR2
rs10846665	12	124,884,297	G	A	0.573	0.982	NCOR2
rs10846666	12	124,884,353	A	G	0.815	0.98	NCOR2
rs10846667	12	124,884,424	C	T	0.516	0.981	NCOR2
rs10846668	12	124,884,443	G	A	0.515	0.983	NCOR2
rs10744175	12	124,884,522	A	G	0.329	0.995	NCOR2
rs2293514	12	124,885,249	C	T	0.336	0.998	NCOR2
rs2293513	12	124,885,290	T	C	0.336	0.998	NCOR2
rs34583413	12	124,885,756	C	CA	0.54	0.935	NCOR2
rs71458841	12	124,886,239	G	T	0.989	0.908	NCOR2
rs2342921	12	124,886,589	T	C	0.157	0.99	NCOR2
rs2342922	12	124,886,690	C	T	0.522	0.983	NCOR2
rs35831183	12	124,887,058	G	GGCT	0.565	0.77	NCOR2
rs906301	12	124,887,189	C	A	0.852	0.991	NCOR2
rs1244058	12	124,887,710	C	T	0.852	0.988	NCOR2
rs10846669	12	124,887,946	A	G	0.832	0.976	NCOR2
rs34583564	12	124,888,809	G	A	0.828	0.981	NCOR2
rs56045702	12	124,889,685	C	T	0.945	0.967	NCOR2
rs76434065	12	124,889,754	A	T	0.967	0.963	NCOR2
rs1244053	12	124,890,212	C	T	0.855	0.974	NCOR2
rs10846670	12	124,890,231	C	T	0.458	0.967	NCOR2
rs56977560	12	124,890,319	G	A	0.883	0.973	NCOR2
rs34135175	12	124,890,536	G	C	0.944	0.966	NCOR2
rs61247712	12	124,891,494	G	A	0.942	0.999	NCOR2
rs57824943	12	124,892,992	T	C	0.944	0.962	NCOR2
rs34777431	12	124,893,328	A	G	0.944	0.961	NCOR2
rs12582168	12	124,894,184	T	C	0.793	0.918	NCOR2
rs2141246	12	124,895,821	G	C	0.174	0.796	NCOR2
rs560167699	12	124,895,823	G	C	0.965	0.613	NCOR2
rs3782264	12	124,896,153	A	G	0.983	0.975	NCOR2
rs3782265	12	124,896,347	G	A	0.458	0.994	NCOR2
rs3782266	12	124,896,416	G	C	0.846	0.982	NCOR2
rs7309449	12	124,896,832	C	G	0.457	0.997	NCOR2
rs3782267	12	124,897,153	C	T	0.458	0.998	NCOR2
12:124897466_CAG_C	12	124,897,466	CAG	C	0.923	0.95	NCOR2
rs149499713	12	124,897,715	A	AC	0.964	0.924	NCOR2
rs73225421	12	124,898,899	C	T	0.935	0.993	NCOR2
rs1794945	12	124,899,881	A	C	0.028	0.993	NCOR2
rs761594467	12	124,900,781	AC	A	0.932	0.952	NCOR2
rs74923662	12	124,900,920	C	T	0.911	0.995	NCOR2
rs112577830	12	124,901,243	C	T	0.972	0.995	NCOR2
12:124901374_ACAGGGAGACCCCGTGGCC_A	12	124,901,374	ACAGGGAGACCCCGTGGCC	A	0.936	0.843	NCOR2
rs79299117	12	124,901,732	G	A	0.98	0.641	NCOR2
rs1702340	12	124,901,852	C	T	0.163	0.997	NCOR2
rs34407234	12	124,902,166	C	CTCT	0.971	0.993	NCOR2
rs3782269	12	124,902,491	C	T	0.988	0.992	NCOR2
rs11608348	12	124,902,593	T	C	0.982	0.588	NCOR2
12:124903024_CG_C	12	124,903,024	CG	C	0.032	0.989	NCOR2
12:124903238_CA_C	12	124,903,238	CA	C	0.971	0.992	NCOR2
rs1702338	12	124,903,514	A	C	0.032	0.993	NCOR2
rs74404514	12	124,903,534	C	G	0.969	0.96	NCOR2
rs1794952	12	124,904,015	C	T	0.836	0.984	NCOR2
rs11837447	12	124,905,116	C	T	0.969	0.982	NCOR2
rs9988975	12	124,905,137	G	C	0.148	0.998	NCOR2
rs2463428	12	124,905,208	C	T	0.028	0.997	NCOR2
rs80271182	12	124,906,776	C	G	0.976	0.989	NCOR2
12:124907237_CA_C	12	124,907,237	CA	C	0.936	0.992	NCOR2

SNP	Chromosome	Position	Effect Allele	Other Allele	Effect Allele Frequency	Imputation quality	Gene
12:124907415_TC_T	12	124,907,415	TC	T	0.975	0.981	NCOR2
rs543181594	12	124,907,424	T	C	0.985	0.875	NCOR2
rs150305452	12	124,907,741	T	G	0.966	0.904	NCOR2
rs3782270	12	124,907,914	G	A	0.417	0.989	NCOR2
rs1702318	12	124,908,024	T	C	0.028	0.993	NCOR2
rs77514136	12	124,908,066	C	T	0.988	0.991	NCOR2
rs10846672	12	124,908,118	G	A	0.417	0.989	NCOR2
rs1794951	12	124,908,315	T	C	0.028	0.992	NCOR2
rs73225424	12	124,908,564	A	C	0.935	0.997	NCOR2
rs2293515	12	124,908,813	A	G	0.714	0.987	NCOR2
rs1244090	12	124,909,777	A	G	0.118	0.992	NCOR2
rs1244091	12	124,909,837	G	A	0.835	0.983	NCOR2
rs10846673	12	124,910,457	G	C	0.417	0.988	NCOR2
rs75494638	12	124,910,598	G	T	0.988	0.993	NCOR2
rs73225425	12	124,911,129	G	C	0.935	0.994	NCOR2
rs1244092	12	124,911,470	A	G	0.061	0.992	NCOR2
rs550290583	12	124,912,635	A	G	0.982	0.513	NCOR2
rs1244093	12	124,912,636	C	T	0.164	0.993	NCOR2
rs7301068	12	124,912,677	G	C	0.968	0.985	NCOR2
rs149549557	12	124,913,134	C	T	0.98	0.985	NCOR2
12:124914585_CCT_C	12	124,914,585	CCT	C	0.935	0.988	NCOR2
rs146690869	12	124,915,067	T	C	0.975	0.989	NCOR2
rs1244096	12	124,915,091	T	C	0.093	0.995	NCOR2
rs59053140	12	124,915,431	T	C	0.86	0.958	NCOR2
rs55654184	12	124,915,901	A	G	0.883	0.983	NCOR2
rs373904013	12	124,915,947	T	C	0.976	0.986	NCOR2
rs1244097	12	124,916,023	C	G	0.809	0.983	NCOR2
12:124916051_CACAT_C	12	124,916,051	CACAT	C	0.784	0.971	NCOR2
12:124916351_GCAGA_G	12	124,916,351	GCAGA	G	0.869	0.969	NCOR2
rs1244098	12	124,916,372	C	A	0.785	0.983	NCOR2
rs115296697	12	124,916,521	G	C	0.975	0.987	NCOR2
rs55671091	12	124,917,410	G	C	0.877	0.972	NCOR2
rs77144223	12	124,918,345	A	G	0.988	0.996	NCOR2
rs73421995	12	124,918,514	C	A	0.97	0.988	NCOR2
rs2660376	12	124,919,791	A	G	0.118	0.993	NCOR2
rs1702324	12	124,919,824	C	T	0.118	0.992	NCOR2
rs117207436	12	124,920,273	T	C	0.961	0.89	NCOR2
rs1702325	12	124,920,813	T	C	0.118	0.994	NCOR2
rs1702323	12	124,921,656	C	T	0.029	0.985	NCOR2
rs146373233	12	124,921,661	A	G	0.985	0.994	NCOR2
rs188644821	12	124,921,677	T	A	0.988	0.935	NCOR2
rs143106171	12	124,921,682	A	T	0.988	0.994	NCOR2
rs1702322	12	124,922,325	C	T	0.214	0.975	NCOR2
rs111575734	12	124,922,735	A	G	0.973	0.989	NCOR2
rs1702321	12	124,922,980	A	G	0.147	0.992	NCOR2
12:124923096_AC_A	12	124,923,096	AC	A	0.81	0.974	NCOR2
rs10161137	12	124,923,165	C	T	0.436	0.989	NCOR2
rs4394929	12	124,923,177	C	T	0.701	0.986	NCOR2
rs147834583	12	124,923,196	G	A	0.972	0.991	NCOR2
rs55766036	12	124,923,216	C	CAG	0.448	0.969	NCOR2
rs145798655	12	124,923,964	G	A	0.875	0.982	NCOR2
rs11616045	12	124,924,024	G	A	0.368	0.979	NCOR2
rs372039271	12	124,924,041	G	A	0.979	0.926	NCOR2
rs556182017	12	124,924,169	C	T	0.938	0.92	NCOR2
rs571762708	12	124,924,407	C	T	0.987	0.656	NCOR2
rs61932029	12	124,924,607	C	G	0.849	0.988	NCOR2
rs2695991	12	124,924,775	A	G	0.026	0.99	NCOR2
rs74968945	12	124,924,975	C	T	0.963	0.96	NCOR2
rs74825512	12	124,925,874	G	C	0.982	0.99	NCOR2
rs117300401	12	124,926,093	C	T	0.972	0.994	NCOR2
rs183323706	12	124,926,191	T	C	0.99	0.955	NCOR2
rs11057625	12	124,926,954	G	C	0.879	0.997	NCOR2
rs7980125	12	124,927,970	G	A	0.32	0.998	NCOR2
rs141568559	12	124,928,031	C	CTTTA	0.879	0.997	NCOR2
rs113016174	12	124,928,197	T	C	0.987	0.887	NCOR2
rs148171074	12	124,928,220	C	T	0.944	0.996	NCOR2
rs1794978	12	124,928,446	C	T	0.856	0.998	NCOR2
rs113844483	12	124,928,608	G	A	0.972	0.998	NCOR2
12:124928917_AGAG_A	12	124,928,917	AGAG	A	0.964	0.696	NCOR2
rs113055626	12	124,929,079	A	G	0.972	0.998	NCOR2
rs1702320	12	124,929,291	T	G	0.11	0.997	NCOR2
rs11057627	12	124,929,906	G	A	0.879	0.997	NCOR2
rs11611457	12	124,929,964	G	A	0.419	0.998	NCOR2
rs12231617	12	124,930,127	C	G	0.879	0.998	NCOR2
rs1243888	12	124,930,214	T	C	0.813	0.997	NCOR2
rs112725166	12	124,930,333	G	C	0.972	0.996	NCOR2
rs1243889	12	124,930,451	T	C	0.607	0.997	NCOR2
rs10846674	12	124,930,732	G	C	0.419	0.996	NCOR2
rs1243890	12	124,931,533	A	G	0.026	0.993	NCOR2
rs112369883	12	124,931,650	G	A	0.966	0.988	NCOR2
rs1243891	12	124,931,665	C	T	0.037	0.995	NCOR2
rs34346265	12	124,931,707	C	T	0.985	0.86	NCOR2
rs10744176	12	124,931,712	C	T	0.42	0.996	NCOR2
rs4765565	12	124,931,727	C	T	0.879	0.998	NCOR2
rs4765566	12	124,932,244	C	T	0.887	0.96	NCOR2
rs55814503	12	124,932,259	C	A	0.879	0.998	NCOR2
rs111590000	12	124,932,357	C	G	0.972	0.999	NCOR2
rs55823529	12	124,932,361	G	A	0.879	0.998	NCOR2
rs111930690	12	124,932,363	C	T	0.972	0.999	NCOR2
rs55856606	12	124,932,638	A	G	0.879	0.998	NCOR2
rs4765567	12	124,932,893	A	G	0.879	0.999	NCOR2
rs111794705	12	124,933,267	G	A	0.972	1	NCOR2
rs883822	12	124,933,744	C	T	0.419	0.996	NCOR2
rs3825138	12	124,933,910	T	G	0.982	0.998	NCOR2
rs73225433	12	124,934,109	C	G	0.963	0.994	NCOR2
rs114071181	12	124,934,449	G	C	0.978	0.97	NCOR2
rs3782276	12	124,935,835	C	T	0.419	0.996	NCOR2
rs112351984	12	124,936,040	G	A	0.968	0.989	NCOR2
rs112074468	12	124,936,117	T	TTCTG	0.608	0.991	NCOR2
rs1812730	12	124,936,219	G	A	0.419	0.996	NCOR2
rs967500	12	124,936,366	G	A	0.419	0.996	NCOR2
rs117868195	12	124,936,379	A	G	0.963	0.977	NCOR2
rs114286918	12	124,937,316	A	G	0.982	0.998	NCOR2
rs114954846	12	124,937,347	T	C	0.982	0.997	NCOR2
rs75467614	12	124,937,410	G	A	0.962	0.888	NCOR2
rs140991946	12	124,937,561	G	A	0.987	0.815	NCOR2
rs114482325	12	124,937,790	C	T	0.982	0.998	NCOR2
rs115775504	12	124,938,373	C	T	0.964	0.989	NCOR2
rs3782277	12	124,938,609	C	T	0.377	0.996	NCOR2
rs758392166	12	124,939,255	ACGGCCACTTTT	A	0.024	0.691	NCOR2
rs12425127	12	124,939,978	T	C	0.972	0.997	NCOR2
rs73225435	12	124,940,762	G	A	0.963	0.993	NCOR2
rs906306	12	124,941,543	C	T	0.882	0.997	NCOR2
rs2277341	12	124,941,816	T	C	0.982	0.996	NCOR2
rs55847449	12	124,941,955	G	A	0.879	0.997	NCOR2
rs113525400	12	124,942,581	G	A	0.972	0.996	NCOR2
rs114705144	12	124,942,600	T	C	0.98	0.977	NCOR2
rs10846676	12	124,942,851	C	T	0.427	0.992	NCOR2
rs1243885	12	124,942,883	G	C	0.822	0.988	NCOR2
rs3782280	12	124,943,288	G	A	0.879	0.997	NCOR2
rs2342923	12	124,944,434	C	T	0.849	0.864	NCOR2
rs117216130	12	124,944,653	A	G	0.966	0.986	NCOR2
rs12425977	12	124,945,244	G	T	0.975	0.997	NCOR2
rs10846677	12	124,945,991	C	G	0.875	0.996	NCOR2
rs142635853	12	124,946,911	C	T	0.984	0.974	NCOR2
rs151004874	12	124,946,961	C	A	0.964	0.999	NCOR2
rs73225437	12	124,948,054	G	A	0.964	0.999	NCOR2
rs1147286	12	124,948,235	G	A	0.833	0.995	NCOR2
rs116153551	12	124,948,307	G	A	0.982	0.997	NCOR2
rs117058202	12	124,948,448	T	C	0.935	0.992	NCOR2
rs74757820	12	124,948,568	C	G	0.964	0.998	NCOR2
rs3782281	12	124,950,060	G	A	0.964	0.997	NCOR2
rs1794944	12	124,950,420	C	G	0.791	0.996	NCOR2
rs4765569	12	124,950,652	T	C	0.839	0.997	NCOR2
rs111602731	12	124,950,923	C	G	0.975	0.996	NCOR2
rs113159998	12	124,950,937	G	A	0.975	0.995	NCOR2
rs1626237	12	124,951,216	G	C	0.791	0.997	NCOR2
rs62650986	12	124,951,900	T	C	0.238	0.986	NCOR2
rs577484459	12	124,951,982	C	CT	0.288	0.657	NCOR2
rs138759859	12	124,952,557	T	C	0.878	0.995	NCOR2
rs147189850	12	124,952,583	A	ATCC	0.384	0.78	NCOR2
rs576474290	12	124,952,633	C	T	0.979	0.628	NCOR2
rs745307817	12	124,952,876	ATCC	A	0.445	0.954	NCOR2
12:124952930_ATCC_A	12	124,952,930	ATCC	A	0.877	0.969	NCOR2
12:124953128_AC_A	12	124,953,128	AC	A	0.431	0.984	NCOR2
12:124953340_ATCATCC_A	12	124,953,340	ATCATCC	A	0.966	0.981	NCOR2
rs12372323	12	124,953,724	G	A	0.868	0.998	NCOR2
rs12830666	12	124,953,976	G	A	0.427	0.997	NCOR2
rs74891675	12	124,954,057	G	C	0.982	0.999	NCOR2
rs1243734	12	124,954,904	C	T	0.038	0.993	NCOR2
rs12371681	12	124,955,192	A	G	0.86	0.997	NCOR2
12:124955272_CAG_C	12	124,955,272	CAG	C	0.962	0.948	NCOR2
rs4765149	12	124,955,517	A	G	0.167	0.996	NCOR2
rs10846678	12	124,956,290	A	G	0.86	0.997	NCOR2
rs1243733	12	124,956,552	C	T	0.897	0.999	NCOR2

SNP	Chromosome	Position	Effect Allele	Other Allele	Effect Allele Frequency	Imputation quality	Gene
rs112375997	12	124,956,663	G	C	0.975	0.998	NCOR2
rs11057630	12	124,956,803	T	A	0.86	0.997	NCOR2
rs112659030	12	124,957,238	T	C	0.975	0.999	NCOR2
rs10846679	12	124,957,627	C	T	0.867	0.996	NCOR2
rs117719191	12	124,957,905	C	T	0.987	0.932	NCOR2
rs117086975	12	124,957,909	T	C	0.952	0.999	NCOR2
rs2088228	12	124,958,142	A	T	0.155	0.984	NCOR2
rs113464544	12	124,958,206	G	T	0.975	0.998	NCOR2
rs2880492	12	124,958,217	T	C	0.952	0.998	NCOR2
rs735010	12	124,958,653	C	T	0.436	0.995	NCOR2
rs1794943	12	124,958,663	G	C	0.035	0.988	NCOR2
rs116891475	12	124,958,830	C	T	0.988	0.966	NCOR2
rs1567162	12	124,959,845	C	T	0.844	0.996	NCOR2
rs12427067	12	124,960,645	C	T	0.975	0.996	NCOR2
rs77438070	12	124,961,328	A	C	0.982	0.808	NCOR2
12:124961448_TAAAAAA_T	12	124,961,448	TAAAAAA	T	0.295	0.953	NCOR2
12:124962368_CA_C	12	124,962,368	CA	C	0.886	0.992	NCOR2
12:124962411_TA_T	12	124,962,411	TA	T	0.898	0.982	NCOR2
rs11057632	12	124,963,620	C	T	0.886	0.995	NCOR2
rs12422635	12	124,963,981	A	C	0.975	0.995	NCOR2
rs1263883	12	124,963,982	C	G	0.648	0.992	NCOR2
rs144568176	12	124,964,000	T	TACAC	0.281	0.895	NCOR2
rs1243732	12	124,964,077	A	G	0.824	0.988	NCOR2
12:124964652_TCAATC_T	12	124,964,652	TCAATC	T	0.972	0.975	NCOR2
rs75444392	12	124,965,288	T	C	0.923	0.99	NCOR2
rs112940925	12	124,965,375	C	T	0.975	0.986	NCOR2
rs7138486	12	124,965,596	G	A	0.89	0.889	NCOR2
rs1243731	12	124,965,623	A	T	0.819	0.991	NCOR2
rs112370898	12	124,965,811	C	T	0.975	0.996	NCOR2
rs113945973	12	124,966,183	T	C	0.975	0.997	NCOR2
rs73225443	12	124,966,355	C	T	0.918	0.809	NCOR2
rs113274152	12	124,967,317	G	A	0.975	0.995	NCOR2
rs11057633	12	124,967,622	G	A	0.886	0.994	NCOR2
rs747443	12	124,968,349	T	C	0.307	0.99	NCOR2
rs906303	12	124,968,359	C	T	0.889	0.987	NCOR2
rs10846680	12	124,969,136	C	T	0.43	0.989	NCOR2
rs142508393	12	124,969,156	C	T	0.987	0.812	NCOR2
rs138479742	12	124,969,219	T	A	0.973	0.904	NCOR2
rs146700870	12	124,969,479	C	T	0.973	0.966	NCOR2
rs1147289	12	124,970,066	G	A	0.857	0.985	NCOR2
rs4765150	12	124,970,087	G	A	0.886	0.994	NCOR2
rs61932053	12	124,970,353	T	C	0.878	0.993	NCOR2
rs73225444	12	124,970,423	C	T	0.971	0.988	NCOR2
rs766468526	12	124,970,497	ACCCCACCTCCCTCCCACT	A	0.819	0.971	NCOR2
rs2280534	12	124,970,875	T	C	0.885	0.999	NCOR2
rs2280532	12	124,970,914	C	T	0.885	0.998	NCOR2
rs11057634	12	124,971,802	G	A	0.46	0.998	NCOR2
12:124972419_TAA_T	12	124,972,419	TAA	T	0.416	0.932	NCOR2
rs882882	12	124,972,475	G	C	0.406	0.961	NCOR2
rs1567163	12	124,973,153	G	A	0.411	0.877	NCOR2
rs12311820	12	124,973,702	G	A	0.94	0.852	NCOR2
rs55637736	12	124,973,707	C	T	0.933	0.843	NCOR2
rs2342924	12	124,973,786	C	T	0.279	0.824	NCOR2
rs201622941	12	124,974,168	G	GC	0.978	0.754	NCOR2
rs60794598	12	124,974,205	C	CCT	0.841	0.819	NCOR2
rs11057636	12	124,974,954	A	G	0.865	0.666	NCOR2
rs572259915	12	124,975,172	A	AC	0.899	0.826	NCOR2
rs12580511	12	124,975,274	A	G	0.753	0.849	NCOR2
rs7979020	12	124,975,674	C	T	0.867	0.888	NCOR2
rs939894	12	124,975,676	G	A	0.738	0.853	NCOR2
rs201838130	12	124,975,715	GT	G	0.414	0.832	NCOR2
rs80132760	12	124,975,748	A	G	0.926	0.831	NCOR2
rs139433080	12	124,976,404	G	A	0.941	0.82	NCOR2
rs12814949	12	124,976,612	C	T	0.921	0.902	NCOR2
rs77475618	12	124,976,982	G	A	0.928	0.966	NCOR2
rs58525539	12	124,978,361	G	C	0.79	0.88	NCOR2
rs527941628	12	124,978,766	G	GAC	0.884	0.895	NCOR2
rs2342926	12	124,978,773	A	G	0.764	0.91	NCOR2
rs872225	12	124,979,789	T	G	0.826	0.899	NCOR2
rs148141514	12	124,979,811	G	A	0.972	0.893	NCOR2
rs872224	12	124,979,833	C	G	0.497	0.911	NCOR2
rs112419507	12	124,980,157	C	T	0.95	0.868	NCOR2
rs77709037	12	124,980,311	G	A	0.773	0.885	NCOR2
rs3759105	12	124,980,431	A	G	0.895	0.958	NCOR2
rs3759106	12	124,980,586	C	T	0.886	0.947	NCOR2
rs117480353	12	124,980,618	G	A	0.984	0.869	NCOR2
rs147646635	12	124,980,663	G	A	0.935	0.993	NCOR2
rs7306073	12	124,980,809	C	T	0.26	0.93	NCOR2
rs66494628	12	124,982,078	A	G	0.931	0.899	NCOR2
rs3759107	12	124,982,111	C	T	0.895	0.956	NCOR2
rs3759108	12	124,982,657	G	A	0.904	0.963	NCOR2
rs4765151	12	124,982,862	G	T	0.218	0.953	NCOR2
rs870844	12	124,983,925	T	C	0.875	0.929	NCOR2
rs1630069	12	124,984,112	T	C	0.851	0.906	NCOR2
rs12322603	12	124,984,486	G	A	0.954	0.827	NCOR2
rs1242999	12	124,984,488	A	G	0.703	0.88	NCOR2
rs80249592	12	124,984,814	G	A	0.986	0.902	NCOR2
rs1242997	12	124,984,828	G	A	0.174	0.95	NCOR2
rs4765572	12	124,984,976	T	A	0.989	0.969	NCOR2
rs4765152	12	124,985,150	C	T	0.789	0.981	NCOR2
rs869331	12	124,985,251	C	T	0.707	0.982	NCOR2
12:124985591_AGCGC_A	12	124,985,591	AGCGC	A	0.106	0.953	NCOR2
rs375542139	12	124,985,622	A	G	0.63	0.753	NCOR2
rs79581243	12	124,985,624	A	G	0.63	0.753	NCOR2
12:124986028_CCTCTGGGGG_C	12	124,986,028	CCTCTGGGGG	C	0.989	0.963	NCOR2
rs870163	12	124,986,454	G	T	0.684	0.99	NCOR2
rs1242993	12	124,986,518	C	T	0.317	0.991	NCOR2
rs1032525	12	124,986,539	C	G	0.317	0.99	NCOR2
rs117221603	12	124,986,604	C	T	0.959	0.973	NCOR2
rs77417099	12	124,986,842	G	A	0.959	0.973	NCOR2
rs941181	12	124,987,078	T	C	0.315	0.992	NCOR2
rs870710	12	124,987,154	G	A	0.316	0.992	NCOR2
rs112478330	12	124,987,398	C	G	0.973	0.923	NCOR2
rs1242991	12	124,987,620	G	A	0.317	0.994	NCOR2
rs57591636	12	124,987,885	T	C	0.869	0.984	NCOR2
rs7979866	12	124,987,982	G	C	0.919	0.985	NCOR2
rs532070914	12	124,988,163	T	C	0.975	0.737	NCOR2
12:124988285_GATGGATGC_G	12	124,988,285	GATGGATGC	G	0.879	0.932	NCOR2
rs1798891	12	124,988,358	G	A	0.064	0.972	NCOR2
rs61933578	12	124,988,785	G	T	0.919	0.986	NCOR2
rs61933579	12	124,989,178	C	T	0.785	0.994	NCOR2
rs11057642	12	124,989,560	C	T	0.785	0.994	NCOR2
rs56325379	12	124,989,655	T	C	0.785	0.994	NCOR2
rs111705351	12	124,989,666	T	C	0.987	0.896	NCOR2
rs864503	12	124,989,900	C	T	0.317	0.999	NCOR2
rs200020619	12	124,991,122	AC	A	0.815	0.977	NCOR2
rs837936	12	124,991,322	G	C	0.225	0.989	NCOR2
rs191490493	12	124,992,730	C	G	0.989	0.891	NCOR2
rs74721149	12	124,993,275	G	A	0.989	0.988	NCOR2
rs71458849	12	124,993,416	G	A	0.989	0.89	NCOR2
rs11837423	12	124,993,538	G	A	0.913	0.991	NCOR2
rs72458904	12	124,993,786	G	GGC	0.076	0.804	NCOR2
rs77991162	12	124,994,425	C	A	0.989	0.997	NCOR2
rs11608538	12	124,995,327	G	A	0.937	0.995	NCOR2
rs60419381	12	124,995,329	G	A	0.916	0.992	NCOR2
rs11608551	12	124,995,394	G	A	0.937	0.994	NCOR2
rs701043	12	124,996,220	A	G	0.225	0.998	NCOR2
rs56314649	12	124,996,295	C	T	0.943	0.993	NCOR2
rs837950	12	124,996,514	G	C	0.225	0.996	NCOR2
rs61933581	12	124,996,667	A	G	0.869	0.98	NCOR2
rs75649450	12	124,997,693	C	G	0.945	0.977	NCOR2
rs701042	12	124,999,152	A	G	0.14	0.99	NCOR2
rs701041	12	124,999,344	G	C	0.166	0.986	NCOR2
12:124999363_CAA_C	12	124,999,363	CAA	C	0.12	0.893	NCOR2
rs138756220	12	124,999,811	G	A	0.987	0.862	NCOR2
rs4765575	12	124,999,913	G	A	0.989	0.963	NCOR2
12:124999926_GA_G	12	124,999,926	GA	G	0.053	0.772	NCOR2
12:125000192_CAT_C	12	125,000,192	CAT	C	0.916	0.965	NCOR2
rs748852184	12	125,000,202	TGA	T	0.301	0.95	NCOR2
12:125000287_CGT_C	12	125,000,287	CGT	C	0.86	0.954	NCOR2
rs58179607	12	125,001,003	A	G	0.917	0.973	NCOR2
rs147497561	12	125,001,164	C	A	0.966	0.906	NCOR2
rs59858345	12	125,001,339	G	GC	0.917	0.958	NCOR2
rs7971663	12	125,001,865	C	T	0.989	0.94	NCOR2
rs6488932	12	125,002,119	T	C	0.988	0.927	NCOR2
rs150954431	12	125,002,323	C	T	0.982	0.808	NCOR2
rs837948	12	125,002,525	G	A	0.685	0.916	NCOR2
rs1692067	12	125,003,647	C	T	0.65	0.623	NCOR2
rs10846681	12	125,003,705	G	A	0.835	0.928	NCOR2
rs11610540	12	125,003,871	G	A	0.768	0.995	NCOR2
rs111229375	12	125,004,114	G	A	0.986	0.869	NCOR2
rs701039	12	125,004,362	G	A	0.186	0.937	NCOR2
rs4765153	12	125,004,392	C	T	0.957	0.947	NCOR2
rs7955154	12	125,004,639	C	T	0.861	0.904	NCOR2
rs7972734	12	125,004,649	T	C	0.896	0.854	NCOR2

SNP	Chromosome	Position	Effect Allele	Other Allele	Effect Allele Frequency	Imputation quality	Gene
rs701038	12	125,005,128	T	C	0.77	0.974	NCOR2
rs701037	12	125,005,142	C	T	0.721	0.967	NCOR2
rs11057652	12	125,005,167	G	C	0.805	0.946	NCOR2
rs701036	12	125,005,483	A	G	0.133	0.982	NCOR2
rs12820652	12	125,005,510	G	A	0.797	0.996	NCOR2
rs12822442	12	125,005,712	C	G	0.797	0.994	NCOR2
rs114131659	12	125,006,145	C	T	0.989	0.975	NCOR2
rs701034	12	125,006,162	A	G	0.362	0.999	NCOR2
rs60007858	12	125,006,344	G	A	0.871	0.987	NCOR2
rs117337655	12	125,006,737	A	T	0.985	0.959	NCOR2
rs77861094	12	125,007,011	C	A	0.871	0.986	NCOR2
rs711158	12	125,007,021	G	A	0.684	0.989	NCOR2
rs77471279	12	125,007,040	T	C	0.95	0.984	NCOR2
rs77823416	12	125,007,083	C	T	0.984	0.984	NCOR2
rs10773091	12	125,007,222	T	C	0.731	0.988	NCOR2
rs865092	12	125,007,401	A	G	0.362	0.998	NCOR2
rs837492	12	125,008,031	C	T	0.681	0.988	NCOR2
rs12370545	12	125,009,074	G	A	0.787	0.989	NCOR2
rs78900942	12	125,009,307	G	C	0.984	0.985	NCOR2
rs2018254	12	125,009,352	G	A	0.917	0.984	NCOR2
rs864492	12	125,009,384	C	T	0.684	0.988	NCOR2
rs185192029	12	125,009,681	G	C	0.984	0.935	NCOR2
rs79564982	12	125,010,239	G	A	0.871	0.985	NCOR2
rs837466	12	125,010,447	T	C	0.217	0.987	NCOR2
12:125010952_GC_G	12	125,010,952	GC	G	0.95	0.979	NCOR2
rs77946381	12	125,011,280	G	A	0.95	0.986	NCOR2
rs76670309	12	125,012,628	C	T	0.95	0.987	NCOR2
rs79617619	12	125,012,759	A	T	0.95	0.987	NCOR2
rs117645397	12	125,012,769	G	C	0.928	0.983	NCOR2
rs117008182	12	125,013,704	G	A	0.95	0.987	NCOR2
rs112706806	12	125,013,720	C	T	0.946	0.958	NCOR2
rs11057656	12	125,014,122	T	C	0.96	0.969	NCOR2
rs11611906	12	125,014,351	T	C	0.95	0.988	NCOR2
rs148385273	12	125,015,729	T	G	0.985	0.957	NCOR2
rs871548	12	125,015,739	C	G	0.206	0.986	NCOR2
rs939895	12	125,016,294	C	T	0.335	0.99	NCOR2
rs11057658	12	125,016,331	G	A	0.72	0.985	NCOR2
rs939896	12	125,016,363	A	T	0.674	0.988	NCOR2
rs12228332	12	125,016,422	C	A	0.726	0.983	NCOR2
rs78407684	12	125,017,089	C	T	0.927	0.987	NCOR2
rs1798881	12	125,017,278	A	C	0.324	0.989	NCOR2
rs776921296	12	125,017,479	CCT	C	0.575	0.797	NCOR2
rs837945	12	125,017,558	T	C	0.132	0.975	NCOR2
rs11057661	12	125,017,600	A	C	0.893	0.961	NCOR2
rs701033	12	125,018,301	T	C	0.133	0.983	NCOR2
rs701032	12	125,018,431	A	G	0.743	0.991	NCOR2
rs532337920	12	125,018,477	A	AT	0.7	0.799	NCOR2
rs701031	12	125,018,502	A	C	0.743	0.991	NCOR2
rs837465	12	125,018,951	A	G	0.314	0.989	NCOR2
rs11405381	12	125,019,002	G	GA	0.617	0.957	NCOR2
rs837464	12	125,019,242	G	A	0.844	0.975	NCOR2
rs701030	12	125,019,660	T	C	0.216	0.991	NCOR2
rs117804988	12	125,019,807	G	A	0.925	0.988	NCOR2
rs58217600	12	125,020,250	C	T	0.924	0.989	NCOR2
rs142286865	12	125,020,440	C	CCA	0.583	0.991	NCOR2
rs117880793	12	125,020,545	G	A	0.973	0.986	NCOR2
rs11057663	12	125,021,165	C	T	0.925	0.99	NCOR2
rs80152279	12	125,021,406	A	G	0.932	0.998	NCOR2
rs837462	12	125,021,715	A	G	0.556	0.991	NCOR2
rs11057664	12	125,021,771	C	T	0.925	0.99	NCOR2
rs837461	12	125,021,807	C	T	0.632	0.991	NCOR2
rs11614747	12	125,022,126	G	A	0.932	0.998	NCOR2
rs151289025	12	125,022,247	C	T	0.982	0.969	NCOR2
rs56700160	12	125,022,407	G	T	0.932	0.999	NCOR2
rs58908973	12	125,022,578	G	A	0.932	0.999	NCOR2
rs117102981	12	125,023,174	G	A	0.925	0.99	NCOR2
rs78682792	12	125,023,175	C	T	0.894	0.987	NCOR2
rs11611492	12	125,023,309	C	T	0.932	0.999	NCOR2
rs4765577	12	125,023,324	C	G	0.51	0.992	NCOR2
rs150061360	12	125,023,561	G	GCCAGGGCA	0.932	0.997	NCOR2
rs12307174	12	125,023,691	C	T	0.664	0.99	NCOR2
rs10846683	12	125,023,711	G	C	0.554	0.988	NCOR2
rs11615887	12	125,024,098	G	A	0.932	0.998	NCOR2
rs139862918	12	125,024,652	T	TCA	0.934	0.975	NCOR2
rs76497469	12	125,024,786	G	T	0.957	0.97	NCOR2
rs837460	12	125,024,792	C	T	0.741	0.995	NCOR2
rs111219308	12	125,025,733	G	A	0.932	0.996	NCOR2
rs116928246	12	125,025,742	G	A	0.55	0.981	NCOR2
rs191888629	12	125,025,779	G	C	0.983	0.825	NCOR2
12:125026361_GA_G	12	125,026,361	GA	G	0.976	0.885	NCOR2
rs4765578	12	125,026,884	A	G	0.545	0.991	NCOR2
rs190061215	12	125,027,086	T	G	0.989	0.927	NCOR2
rs148554134	12	125,027,313	C	A	0.983	0.886	NCOR2
rs73425652	12	125,027,424	G	A	0.932	0.996	NCOR2
rs117996979	12	125,028,037	C	A	0.894	0.988	NCOR2
rs117013704	12	125,028,148	C	T	0.925	0.992	NCOR2
rs837478	12	125,029,190	G	A	0.075	0.991	NCOR2
rs11057665	12	125,029,570	G	A	0.548	0.991	NCOR2
rs837479	12	125,029,775	C	T	0.671	0.991	NCOR2
rs837480	12	125,029,792	A	G	0.672	0.991	NCOR2
rs11057666	12	125,030,170	A	G	0.294	0.994	NCOR2
rs837481	12	125,030,197	C	T	0.781	0.996	NCOR2
rs11611313	12	125,030,210	G	A	0.932	0.994	NCOR2
rs837482	12	125,030,939	T	C	0.075	0.992	NCOR2
rs147288572	12	125,031,050	T	C	0.891	0.989	NCOR2
rs7136910	12	125,031,761	A	C	0.57	0.993	NCOR2
rs151169012	12	125,032,136	C	T	0.984	0.872	NCOR2
rs885026	12	125,032,789	C	A	0.57	0.992	NCOR2
rs2001346	12	125,032,798	C	T	0.845	0.99	NCOR2
rs11609965	12	125,032,946	G	A	0.933	0.985	NCOR2
rs11616150	12	125,032,947	A	T	0.933	0.985	NCOR2
rs885025	12	125,033,035	A	G	0.565	0.993	NCOR2
rs837484	12	125,033,328	A	C	0.804	0.997	NCOR2
rs10773092	12	125,033,338	T	G	0.706	0.992	NCOR2
rs701021	12	125,033,497	G	A	0.121	0.992	NCOR2
rs117905932	12	125,033,542	T	A	0.977	0.944	NCOR2
rs11057667	12	125,033,839	G	A	0.925	0.995	NCOR2
rs139784632	12	125,033,851	T	TA	0.929	0.993	NCOR2
rs79075254	12	125,034,044	G	A	0.932	0.992	NCOR2
rs148926209	12	125,034,208	G	C	0.973	0.893	NCOR2
rs10846684	12	125,034,350	G	A	0.774	0.993	NCOR2
rs837485	12	125,034,683	A	G	0.803	0.996	NCOR2
rs796235669	12	125,034,710	A	AG	0.075	0.993	NCOR2
rs837486	12	125,034,711	A	C	0.075	0.992	NCOR2
rs141874977	12	125,035,362	G	A	0.973	0.955	NCOR2
rs701023	12	125,035,524	A	C	0.804	0.995	NCOR2
rs939893	12	125,035,703	C	T	0.805	0.995	NCOR2
rs701024	12	125,036,339	G	A	0.805	0.994	NCOR2
rs113162522	12	125,036,619	A	C	0.932	0.991	NCOR2
rs837487	12	125,036,718	C	T	0.075	0.998	NCOR2
rs117338341	12	125,037,491	A	G	0.925	0.996	NCOR2
rs192399245	12	125,038,144	G	T	0.985	0.948	NCOR2
rs837489	12	125,038,397	T	C	0.075	0.997	NCOR2
rs837490	12	125,038,926	C	T	0.821	0.98	NCOR2
rs78424865	12	125,039,083	C	T	0.87	0.99	NCOR2
rs151220919	12	125,039,147	C	T	0.952	0.965	NCOR2
rs79636296	12	125,039,148	G	A	0.872	0.993	NCOR2
rs763900892	12	125,039,547	CACAGTCCAGGGG	C	0.652	0.975	NCOR2
rs76322511	12	125,040,007	G	A	0.872	0.993	NCOR2
rs149791498	12	125,040,807	A	AG	0.872	0.988	NCOR2
rs837491	12	125,041,081	G	A	0.809	0.985	NCOR2
rs144925609	12	125,041,396	CA	C	0.641	0.874	NCOR2
rs113603401	12	125,042,039	C	T	0.989	0.825	NCOR2
rs701025	12	125,042,336	T	C	0.808	0.984	NCOR2
rs531027713	12	125,042,812	A	AC	0.654	0.969	NCOR2
rs701026	12	125,042,872	A	G	0.255	0.984	NCOR2
rs12369870	12	125,042,873	G	C	0.924	0.972	NCOR2
rs701027	12	125,043,419	G	C	0.605	0.984	NCOR2
rs11057671	12	125,043,791	G	A	0.65	0.983	NCOR2
rs10846685	12	125,044,746	A	T	0.925	0.979	NCOR2
rs10846686	12	125,045,278	C	T	0.925	0.978	NCOR2
rs11057673	12	125,045,404	T	C	0.971	0.947	NCOR2
rs4765579	12	125,045,520	T	A	0.65	0.983	NCOR2
rs531271752	12	125,045,834	A	C	0.954	0.629	NCOR2
rs10773093	12	125,046,036	T	C	0.573	0.982	NCOR2
rs117231757	12	125,046,095	G	A	0.931	0.994	NCOR2
rs139139370	12	125,046,608	T	C	0.988	0.959	NCOR2
rs13377760	12	125,047,095	C	T	0.925	0.976	NCOR2
rs13377762	12	125,047,128	C	T	0.925	0.976	NCOR2
rs837511	12	125,047,289	A	G	0.178	0.992	NCOR2
rs7972214	12	125,047,758	A	G	0.605	0.977	NCOR2
rs117700021	12	125,048,086	C	T	0.971	0.945	NCOR2
rs117089640	12	125,048,557	A	G	0.983	0.948	NCOR2
rs864493	12	125,049,808	G	A	0.84	0.97	NCOR2
rs113634180	12	125,050,011	T	A	0.988	0.835	NCOR2

SNP	Chromosome	Position	Effect Allele	Other Allele	Effect Allele Frequency	Imputation quality	Gene
rs12316085	12	125,051,053	G	A	0.942	0.973	NCOR2
rs79830634	12	125,051,843	C	T	0.771	0.95	NCOR2
12:125052155_TGGCGGCGGCGGCGGC_T	12	125,052,155	TGGCGGCGGCGGCGGC	T	0.382	0.908	NCOR2
rs74054236	14	54,416,662	G	A	0.985	0.992	BMP4
rs2071047	14	54,418,411	G	A	0.593	0.994	BMP4
rs542905354	14	54,418,419	C	G	0.978	0.595	BMP4
rs562412111	14	54,418,520	C	T	0.901	0.657	BMP4
rs72680532	14	54,418,543	C	T	0.987	0.944	BMP4
rs35107139	14	54,419,106	A	C	0.598	0.934	BMP4
rs10130587	14	54,419,110	G	C	0.597	0.918	BMP4
rs59035991	14	54,419,552	T	A	0.95	0.984	BMP4
rs2855532	14	54,419,965	G	A	0.54	0.991	BMP4
rs2761882	14	54,420,309	C	T	0.501	0.984	BMP4
rs71446481	14	54,420,647	A	ACC	0.552	0.985	BMP4
rs141900713	14	54,420,892	T	G	0.954	0.947	BMP4
rs2761884	14	54,421,052	G	T	0.549	0.995	BMP4
rs201885725	14	54,421,177	G	C	0.983	0.907	BMP4
rs2855530	14	54,421,917	G	C	0.502	0.996	BMP4
14:54421998_GCCCGGGTT_G	14	54,421,998	GCCCGGGTT	G	0.957	0.977	BMP4
rs114176271	14	54,422,524	C	T	0.97	0.989	BMP4
rs762643	14	54,422,767	G	T	0.55	0.999	BMP4
rs9925964	16	31,129,895	A	G	0.643	0.998	KAT8
rs1978487	16	31,129,942	C	T	0.362	0.998	KAT8
rs1978485	16	31,131,121	C	T	0.362	0.998	KAT8
rs4889620	16	31,131,174	G	A	0.643	0.998	KAT8
rs4527034	16	31,131,614	A	G	0.358	0.998	KAT8
rs11865499	16	31,132,250	A	G	0.714	0.998	KAT8
rs113125305	16	31,132,406	T	TTG	0.547	0.945	KAT8
rs571230406	16	31,132,455	T	G	0.983	0.547	KAT8
rs56388508	16	31,132,510	C	T	0.644	0.999	KAT8
rs149604995	16	31,132,633	G	A	0.965	0.969	KAT8
rs28725459	16	31,132,662	G	A	0.714	0.998	KAT8
rs59735493	16	31,133,100	G	A	0.714	0.998	KAT8
rs11649030	16	31,133,110	A	G	0.644	0.999	KAT8
rs1060506	16	31,133,449	C	T	0.714	0.998	KAT8
rs61320757	16	31,134,059	G	A	0.719	0.998	KAT8
rs60996860	16	31,134,213	T	A	0.72	0.984	KAT8
rs138259061	16	31,136,066	A	AAAAG	0.638	0.995	KAT8
rs4404083	16	31,136,085	T	C	0.356	0.999	KAT8
rs889548	16	31,137,712	C	T	0.643	0.998	KAT8
rs72785553	16	31,138,445	G	A	0.644	0.999	KAT8
16:31140187_AG_A	16	31,140,187	AG	A	0.921	0.989	KAT8
rs9936329	16	31,140,799	G	T	0.643	0.998	KAT8
rs1549293	16	31,141,993	C	T	0.645	0.997	KAT8
rs41522747	16	58,060,611	T	G	0.241	0.993	MMP15
rs41418849	16	58,062,081	T	G	0.941	0.958	MMP15
rs182900625	16	58,062,414	G	T	0.989	0.948	MMP15
rs187532996	16	58,062,415	A	T	0.989	0.948	MMP15
rs11648508	16	58,063,513	G	T	0.317	0.991	MMP15
rs41352550	16	58,064,274	G	C	0.98	0.991	MMP15
rs76045219	16	58,065,098	G	A	0.987	0.912	MMP15
rs4784886	16	58,065,459	G	A	0.317	0.99	MMP15
rs4784887	16	58,065,624	G	A	0.299	0.99	MMP15
rs190910517	16	58,066,073	C	A	0.985	0.904	MMP15
16:58066923_TG_T	16	58,066,923	TG	T	0.299	0.984	MMP15
rs111361894	16	58,068,637	C	T	0.977	0.945	MMP15
rs572408280	16	58,068,823	G	GCCTGATCCTCCCCCAC	0.989	0.723	MMP15
rs41393749	16	58,069,541	G	C	0.987	0.915	MMP15
rs41493346	16	58,069,822	C	T	0.977	0.943	MMP15
rs12928392	16	58,070,964	T	A	0.762	0.999	MMP15
rs1436426	16	58,072,570	A	G	0.756	0.996	MMP15
rs144162485	16	58,073,018	C	T	0.988	0.954	MMP15
rs144705678	16	58,073,477	C	T	0.982	0.908	MMP15
rs2304488	16	58,074,210	A	G	0.762	0.997	MMP15
rs147884368	16	58,074,219	C	A	0.982	0.947	MMP15
rs12447579	16	58,075,085	G	A	0.778	0.998	MMP15
rs41390948	16	58,075,235	C	T	0.986	0.942	MMP15
rs12447804	16	58,075,282	C	T	0.778	0.998	MMP15
rs2118018	16	58,076,422	A	G	0.775	0.996	MMP15
rs147487572	16	58,076,581	C	T	0.988	0.994	MMP15
rs41504346	16	58,079,127	A	G	0.979	0.865	MMP15
rs3743563	16	58,079,165	G	A	0.778	0.995	MMP15
rs150382270	16	58,079,376	C	T	0.988	0.995	MMP15
rs41411446	16	58,079,868	T	C	0.957	0.945	MMP15
rs11076213	16	58,080,009	C	T	0.395	0.982	MMP15
rs1050779	16	58,080,215	G	C	0.777	0.994	MMP15
rs3743735	16	68,119,555	G	A	0.873	1	NFATC3
rs78537727	16	68,120,606	A	G	0.873	1	NFATC3
rs36046686	16	68,122,269	T	C	0.88	0.95	NFATC3
rs2418738	16	68,122,604	G	C	0.872	1	NFATC3
rs2418737	16	68,122,726	T	C	0.872	1	NFATC3
rs75739761	16	68,122,914	A	G	0.955	0.999	NFATC3
rs59708959	16	68,123,916	A	T	0.873	1	NFATC3
16:68124198_AC_A	16	68,124,198	AC	A	0.356	0.845	NFATC3
rs237834	16	68,125,316	A	G	0.025	0.952	NFATC3
16:68125378_TGTA_T	16	68,125,378	TGTA	T	0.979	0.993	NFATC3
rs12446198	16	68,125,972	G	A	0.873	1	NFATC3
rs117225319	16	68,126,366	A	T	0.985	0.864	NFATC3
rs28508024	16	68,126,606	T	C	0.873	0.997	NFATC3
rs75735284	16	68,126,635	C	A	0.874	1	NFATC3
16:68126984_TCCA_T	16	68,126,984	TCCA	T	0.874	0.993	NFATC3
16:68127222_TAG_T	16	68,127,222	TAG	T	0.874	0.998	NFATC3
rs1074143	16	68,127,278	A	G	0.874	0.999	NFATC3
rs765127530	16	68,127,846	AT	A	0.955	0.999	NFATC3
rs12599178	16	68,128,104	A	G	0.824	1	NFATC3
rs114329216	16	68,129,101	A	G	0.955	1	NFATC3
rs74604428	16	68,129,638	A	G	0.913	0.992	NFATC3
rs76595854	16	68,130,233	T	C	0.874	1	NFATC3
rs78961715	16	68,131,407	A	G	0.955	0.998	NFATC3
rs9932251	16	68,131,732	G	A	0.872	1	NFATC3
rs7192187	16	68,132,109	T	C	0.872	1	NFATC3
rs237833	16	68,132,203	A	T	0.429	0.774	NFATC3
rs11864819	16	68,133,263	A	G	0.824	0.999	NFATC3
rs140020897	16	68,134,202	G	A	0.984	0.959	NFATC3
rs111606836	16	68,134,278	C	T	0.874	1	NFATC3
rs9928783	16	68,134,296	T	C	0.827	0.999	NFATC3
rs143820262	16	68,134,790	C	CTGTG	0.877	0.997	NFATC3
rs147100136	16	68,136,001	G	T	0.955	0.998	NFATC3
rs8058690	16	68,136,622	T	G	0.872	1	NFATC3
rs72790386	16	68,136,932	G	T	0.967	0.997	NFATC3
rs77996485	16	68,137,420	C	G	0.898	0.994	NFATC3
rs78479045	16	68,137,486	G	T	0.97	0.999	NFATC3
rs117784871	16	68,138,091	G	T	0.942	0.929	NFATC3
rs74817508	16	68,138,349	A	G	0.955	0.998	NFATC3
rs778016298	16	68,138,556	G	GTTTTT	0.941	0.69	NFATC3
rs147561310	16	68,138,672	C	T	0.955	0.998	NFATC3
rs141859350	16	68,138,715	G	A	0.961	0.956	NFATC3
rs76984161	16	68,139,423	C	A	0.875	0.999	NFATC3
rs139472624	16	68,140,237	C	G	0.985	0.976	NFATC3
rs117815871	16	68,140,319	C	T	0.97	0.96	NFATC3
rs4627335	16	68,140,639	T	G	0.675	0.978	NFATC3
rs193192671	16	68,140,648	T	G	0.875	1	NFATC3
rs151193115	16	68,140,780	T	C	0.955	0.998	NFATC3
rs112529313	16	68,143,483	T	G	0.874	1	NFATC3
rs8047978	16	68,143,598	T	G	0.827	1	NFATC3
rs8047343	16	68,143,745	G	A	0.825	0.999	NFATC3
rs111337363	16	68,144,519	AT	A	0.901	0.979	NFATC3
rs140374938	16	68,144,905	C	T	0.985	0.974	NFATC3
rs7194894	16	68,145,062	C	T	0.873	1	NFATC3
rs75286242	16	68,145,798	C	T	0.875	0.999	NFATC3
rs55642862	16	68,146,535	A	ATTT	0.826	0.995	NFATC3
rs8044328	16	68,146,859	G	C	0.872	1	NFATC3
rs116988148	16	68,146,952	C	T	0.955	0.998	NFATC3
rs150788247	16	68,147,929	A	G	0.984	0.965	NFATC3
rs7188350	16	68,149,316	T	C	0.828	1	NFATC3
rs12447640	16	68,150,527	A	G	0.875	0.999	NFATC3
rs12599880	16	68,152,035	A	G	0.874	1	NFATC3
rs7205935	16	68,152,157	A	G	0.827	1	NFATC3
rs73612690	16	68,153,200	G	A	0.873	1	NFATC3
rs76419201	16	68,153,553	G	A	0.875	1	NFATC3
rs545780178	16	68,154,017	CA	C	0.888	0.742	NFATC3
rs139784051	16	68,154,151	C	T	0.875	1	NFATC3
rs112099181	16	68,154,230	T	C	0.873	1	NFATC3
rs28700885	16	68,154,544	A	G	0.828	1	NFATC3
rs2418736	16	68,154,862	G	A	0.825	0.999	NFATC3
16:68154994_CA_C	16	68,154,994	CA	C	0.967	0.589	NFATC3
rs12445396	16	68,155,121	G	A	0.875	1	NFATC3
rs778309194	16	68,155,860	GTT	G	0.875	1	NFATC3
rs547087364	16	68,157,400	CA	C	0.752	0.815	NFATC3
rs118005841	16	68,157,562	A	T	0.874	0.999	NFATC3
rs148018782	16	68,157,676	C	T	0.955	0.999	NFATC3
16:68157711_AACCGTGTCTCTACAAAAAATAC_A	16	68,157,711	AACCGTGTCTCTACAAAAAATAC	A	0.907	0.95	NFATC3
rs137997039	16	68,157,770	C	T	0.979	0.939	NFATC3
rs3815173	16	68,160,289	T	G	0.886	1	NFATC3
rs111339124	16	68,160,778	C	T	0.975	0.979	NFATC3

SNP	Chromosome	Position	Effect Allele	Other Allele	Effect Allele Frequency	Imputation quality	Gene
rs6499160	16	68,160,968	G	T	0.84	1	NFATC3
rs73612697	16	68,162,016	G	A	0.886	1	NFATC3
rs568419724	16	68,163,572	G	C	0.398	0.671	NFATC3
rs4640173	16	68,163,602	C	A	0.84	1	NFATC3
rs4567701	16	68,163,662	T	C	0.84	1	NFATC3
rs75229939	16	68,164,949	G	A	0.887	1	NFATC3
rs78229587	16	68,165,243	T	C	0.887	1	NFATC3
rs140912340	16	68,165,431	T	A	0.955	1	NFATC3
rs56230350	16	68,166,971	A	C	0.887	1	NFATC3
rs150881084	16	68,167,014	C	G	0.955	1	NFATC3
rs12597573	16	68,167,521	C	A	0.887	1	NFATC3
rs61202983	16	68,167,786	T	A	0.955	0.998	NFATC3
rs9939821	16	68,167,787	T	A	0.853	0.946	NFATC3
rs28431294	16	68,167,788	A	T	0.956	0.88	NFATC3
rs7206792	16	68,168,200	C	G	0.84	1	NFATC3
16:68168886_CT_C	16	68,168,886	CT	C	0.909	0.97	NFATC3
rs779371097	16	68,169,279	TGA	T	0.955	1	NFATC3
rs373528972	16	68,169,321	G	A	0.988	0.942	NFATC3
rs111518243	16	68,169,332	A	G	0.988	0.941	NFATC3
rs140979755	16	68,169,694	G	A	0.887	1	NFATC3
rs117300105	16	68,170,298	C	T	0.955	1	NFATC3
rs117751972	16	68,171,968	C	T	0.887	1	NFATC3
rs12596883	16	68,172,416	A	T	0.886	1	NFATC3
rs75788262	16	68,172,490	A	G	0.955	0.999	NFATC3
rs74851284	16	68,172,568	C	G	0.955	1	NFATC3
rs144029258	16	68,172,966	A	G	0.987	0.967	NFATC3
rs116971206	16	68,173,156	T	C	0.955	1	NFATC3
rs62057369	16	68,173,293	A	G	0.972	0.95	NFATC3
rs7195398	16	68,175,289	G	A	0.955	1	NFATC3
rs74629553	16	68,176,415	C	A	0.887	1	NFATC3
rs76495028	16	68,177,036	G	C	0.891	0.993	NFATC3
rs543522911	16	68,178,573	A	T	0.976	0.97	NFATC3
rs4517790	16	68,178,646	C	G	0.875	1	NFATC3
rs139277503	16	68,178,851	G	C	0.958	0.999	NFATC3
rs779205090	16	68,179,036	CT	C	0.932	0.791	NFATC3
rs55723050	16	68,179,151	G	A	0.912	0.99	NFATC3
rs12447931	16	68,179,195	C	T	0.875	1	NFATC3
rs201845231	16	68,179,220	G	GT	0.955	1	NFATC3
rs55790290	16	68,180,242	A	G	0.875	1	NFATC3
rs116867512	16	68,180,834	T	G	0.988	0.979	NFATC3
rs74327209	16	68,181,129	T	G	0.981	0.991	NFATC3
rs9922165	16	68,181,177	C	A	0.873	1	NFATC3
rs146894512	16	68,181,230	A	G	0.981	0.991	NFATC3
rs141536427	16	68,181,412	C	T	0.988	0.967	NFATC3
rs9938553	16	68,183,064	T	A	0.873	1	NFATC3
rs141876578	16	68,183,250	A	G	0.981	0.991	NFATC3
rs118143176	16	68,184,250	C	T	0.955	1	NFATC3
rs147965979	16	68,185,154	T	C	0.986	0.967	NFATC3
rs7199487	16	68,186,082	A	G	0.886	1	NFATC3
rs7199699	16	68,186,186	A	G	0.885	1	NFATC3
rs7199878	16	68,186,301	A	G	0.887	1	NFATC3
rs111701446	16	68,187,718	G	A	0.887	1	NFATC3
16:68187782_CT_C	16	68,187,782	CT	C	0.558	0.995	NFATC3
rs114739995	16	68,188,464	G	A	0.955	1	NFATC3
rs12446007	16	68,189,054	C	T	0.875	1	NFATC3
rs8044995	16	68,189,340	G	A	0.84	1	NFATC3
rs11275011	16	68,189,591	T	TCAGTTAAAGTC	0.842	0.994	NFATC3
rs55725810	16	68,189,636	C	T	0.875	1	NFATC3
rs117806247	16	68,189,730	C	A	0.982	0.895	NFATC3
rs561946216	16	68,190,237	A	AAC	0.876	0.988	NFATC3
rs562498359	16	68,190,239	A	AC	0.876	0.988	NFATC3
rs77884900	16	68,190,239	A	C	0.969	0.528	NFATC3
rs8060893	16	68,191,608	A	T	0.875	1	NFATC3
rs560272945	16	68,192,024	G	GA	0.985	0.962	NFATC3
rs56111587	16	68,192,097	T	G	0.958	0.999	NFATC3
rs34395120	16	68,192,672	G	GA	0.873	0.999	NFATC3
16:68193307_CT_C	16	68,193,307	CT	C	0.987	0.801	NFATC3
rs550241593	16	68,193,323	T	A	0.983	0.889	NFATC3
rs7194694	16	68,193,585	T	C	0.827	0.995	NFATC3
rs117817130	16	68,193,849	C	T	0.988	0.977	NFATC3
rs184938575	16	68,194,611	A	G	0.985	0.868	NFATC3
rs1968065	16	68,194,647	T	G	0.546	0.993	NFATC3
rs748496395	16	68,194,863	CATCT	C	0.825	0.998	NFATC3
rs117620519	16	68,194,889	C	T	0.958	0.998	NFATC3
16:68196851_TAAC_T	16	68,196,851	TAAC	T	0.836	0.966	NFATC3
rs186642615	16	68,197,170	G	A	0.875	1	NFATC3
rs56097211	16	68,198,088	T	C	0.873	1	NFATC3
rs56138523	16	68,198,595	G	A	0.828	0.999	NFATC3
rs75126935	16	68,198,999	A	C	0.958	0.998	NFATC3
rs149437058	16	68,199,295	C	T	0.701	0.589	NFATC3
rs7188900	16	68,201,878	G	A	0.887	1	NFATC3
rs185383158	16	68,202,375	T	G	0.955	0.999	NFATC3
rs4359427	16	68,202,747	C	T	0.875	0.996	NFATC3
rs79323321	16	68,203,360	T	A	0.949	0.952	NFATC3
rs191333125	16	68,203,714	C	T	0.988	0.966	NFATC3
rs34579856	16	68,204,789	C	CTT	0.872	0.998	NFATC3
rs12600144	16	68,205,054	G	A	0.886	1	NFATC3
rs8046355	16	68,205,214	T	A	0.827	1	NFATC3
rs111967459	16	68,205,656	A	G	0.874	1	NFATC3
rs142270245	16	68,206,435	C	T	0.981	0.992	NFATC3
rs118030546	16	68,206,660	G	A	0.969	0.995	NFATC3
rs116990236	16	68,207,913	G	A	0.982	0.922	NFATC3
rs145337756	16	68,208,549	A	G	0.986	0.969	NFATC3
rs75796305	16	68,208,791	A	G	0.874	1	NFATC3
rs12448677	16	68,209,741	G	A	0.875	1	NFATC3
rs8044014	16	68,209,872	T	C	0.873	1	NFATC3
rs8044558	16	68,210,165	T	C	0.873	1	NFATC3
rs8048034	16	68,210,604	A	G	0.827	1	NFATC3
rs75450962	16	68,210,868	C	T	0.875	1	NFATC3
rs548092276	16	68,210,935	C	CT	0.836	0.952	NFATC3
rs113579235	16	68,211,157	T	C	0.875	1	NFATC3
rs62057372	16	68,211,249	C	T	0.978	0.936	NFATC3
rs28548264	16	68,211,321	A	G	0.873	1	NFATC3
16:68211482_ACTT_A	16	68,211,482	ACTT	A	0.874	0.994	NFATC3
rs117240558	16	68,212,641	C	G	0.97	0.984	NFATC3
rs75297764	16	68,212,820	C	T	0.875	1	NFATC3
rs116883401	16	68,213,125	C	T	0.985	0.973	NFATC3
rs74775711	16	68,213,680	C	T	0.958	0.997	NFATC3
rs73612222	16	68,216,072	A	G	0.874	1	NFATC3
rs78587942	16	68,216,739	G	T	0.955	1	NFATC3
rs191996617	16	68,218,686	A	C	0.875	1	NFATC3
16:68218735_AAC_A	16	68,218,735	AAC	A	0.899	0.84	NFATC3
rs12445876	16	68,218,742	A	C	0.875	1	NFATC3
rs113726439	16	68,218,748	C	CAG	0.884	0.939	NFATC3
rs12448991	16	68,218,894	T	A	0.874	0.995	NFATC3
rs56159097	16	68,219,728	C	T	0.989	0.864	NFATC3
rs9932414	16	68,219,969	A	G	0.825	0.999	NFATC3
rs749757405	16	68,220,681	CTGTTATGTTAG	C	0.833	0.981	NFATC3
rs117078787	16	68,221,389	G	A	0.989	0.973	NFATC3
rs148479799	16	68,222,290	G	A	0.984	0.967	NFATC3
rs4275847	16	68,222,830	C	T	0.99	0.957	NFATC3
rs9923710	16	68,224,018	C	T	0.873	1	NFATC3
rs7199588	16	68,224,582	C	T	0.873	1	NFATC3
rs543537776	16	68,226,164	G	A	0.708	0.91	NFATC3
rs755672344	16	68,226,164	G	GTA	0.654	0.899	NFATC3
rs577002611	16	68,226,182	G	GTA	0.955	0.999	NFATC3
rs12933243	16	68,226,237	G	T	0.693	0.803	NFATC3
rs12933247	16	68,226,239	G	T	0.946	0.646	NFATC3
rs1125333	16	68,227,566	C	A	0.874	1	NFATC3
rs1125332	16	68,227,780	A	G	0.874	1	NFATC3
rs1125331	16	68,228,119	T	C	0.872	1	NFATC3
rs55757502	16	68,228,427	C	T	0.874	1	NFATC3
rs113804408	16	68,229,113	G	A	0.874	0.999	NFATC3
rs117999269	16	68,229,123	A	G	0.897	0.958	NFATC3
16:68229282_AG_A	16	68,229,282	AG	A	0.989	0.981	NFATC3
rs200794675	16	68,229,579	T	TGGTCGC	0.875	0.999	NFATC3
rs71393983	16	68,232,344	A	G	0.975	0.952	NFATC3
rs531423438	16	68,232,794	C	CA	0.987	0.904	NFATC3
rs111632263	16	68,233,122	T	A	0.875	0.995	NFATC3
rs141991634	16	68,233,150	A	G	0.828	0.998	NFATC3
rs9746872	16	68,235,052	A	C	0.827	0.999	NFATC3
16:68235731_CATGCCTGTAGTCCCAGCTACTTGGGAGGCTGGCATAGGAGAA_C	16	68,235,731	CATGCCTGTAGTCCCAGCTACTTGGGAGGCTGGCATAGGAGAA	C	0.875	0.999	NFATC3
rs55857142	16	68,236,741	A	T	0.874	0.996	NFATC3
rs6499161	16	68,238,197	A	G	0.828	0.999	NFATC3
rs114645460	16	68,238,511	C	T	0.955	0.999	NFATC3
rs113502835	16	68,238,965	A	T	0.874	1	NFATC3
rs6499162	16	68,239,460	T	C	0.827	0.999	NFATC3
rs8064114	16	68,240,185	G	A	0.872	0.999	NFATC3
rs79719939	16	68,240,216	A	G	0.874	1	NFATC3
16:68240908_TA_T	16	68,240,908	TA	T	0.897	0.707	NFATC3
rs7195172	16	68,241,608	A	G	0.872	1	NFATC3
rs60249778	16	68,241,829	T	TC	0.828	0.999	NFATC3
rs118035472	16	68,242,311	A	G	0.874	1	NFATC3
rs57408712	16	68,242,573	G	C	0.874	1	NFATC3
rs112759570	16	68,242,631	C	CA	0.976	0.575	NFATC3

SNP	Chromosome	Position	Effect Allele	Other Allele	Effect Allele Frequency	Imputation quality	Gene
rs76053508	16	68,243,054	A	T	0.874	1	NFATC3
rs113610474	16	68,243,884	C	CATG	0.825	0.998	NFATC3
rs147356036	16	68,243,966	C	A	0.955	1	NFATC3
rs142497510	16	68,244,070	G	A	0.955	1	NFATC3
16:68244200_CA_C	16	68,244,200	CA	C	0.829	0.971	NFATC3
rs113790673	16	68,244,347	C	T	0.874	1	NFATC3
rs12447080	16	68,245,331	C	T	0.874	1	NFATC3
rs143652376	16	68,245,335	C	T	0.955	0.994	NFATC3
rs148073592	16	68,245,363	A	G	0.9	0.993	NFATC3
rs2418735	16	68,245,385	G	A	0.872	1	NFATC3
rs12444968	16	68,245,598	T	C	0.874	0.999	NFATC3
rs77055954	16	68,246,428	C	T	0.955	0.999	NFATC3
rs1073632	16	68,248,607	G	A	0.955	0.999	NFATC3
rs146129762	16	68,248,742	G	A	0.978	0.962	NFATC3
rs145157356	16	68,249,757	A	G	0.955	0.999	NFATC3
rs146091695	16	68,249,821	T	C	0.97	0.985	NFATC3
rs146980965	16	68,249,923	G	A	0.955	0.999	NFATC3
rs35830195	16	68,250,567	T	TG	0.891	0.995	NFATC3
rs117132504	16	68,251,299	A	G	0.913	0.977	NFATC3
rs9938020	16	68,251,784	C	G	0.84	0.999	NFATC3
rs9928653	16	68,252,079	T	C	0.837	1	NFATC3
rs746112373	16	68,252,478	CT	C	0.979	0.515	NFATC3
rs111402974	16	68,252,516	A	G	0.837	0.999	NFATC3
rs148537303	16	68,252,523	C	G	0.887	1	NFATC3
rs544615798	16	68,252,563	C	T	0.984	0.965	NFATC3
rs142854649	16	68,252,627	C	T	0.971	0.98	NFATC3
rs59057303	16	68,252,744	A	G	0.012	0.83	NFATC3
rs13339471	16	68,253,008	A	G	0.84	0.999	NFATC3
rs8048364	16	68,253,324	T	G	0.837	0.999	NFATC3
rs8048365	16	68,253,326	T	A	0.837	0.999	NFATC3
rs185856818	16	68,253,389	G	A	0.984	0.967	NFATC3
rs117992789	16	68,253,882	C	T	0.985	0.977	NFATC3
rs117761434	16	68,254,196	G	A	0.887	1	NFATC3
rs12444313	16	68,254,404	A	C	0.887	0.999	NFATC3
rs766042696	16	68,254,660	C	CA	0.851	0.945	NFATC3
rs111948678	16	68,255,959	G	A	0.887	0.999	NFATC3
rs200585603	16	68,256,005	C	CAAT	0.956	0.963	NFATC3
16:68256239_GA_G	16	68,256,239	GA	G	0.98	0.832	NFATC3
rs9931987	16	68,256,639	G	A	0.885	1	NFATC3
16:68258026_GT_G	16	68,258,026	GT	G	0.892	0.952	NFATC3
rs11075669	16	68,258,507	G	A	0.887	1	NFATC3
16:68258662_CAGAA_C	16	68,258,662	CAGAA	C	0.99	0.903	NFATC3
rs12598630	16	68,258,852	T	G	0.887	0.999	NFATC3
rs9922934	16	68,258,981	G	A	0.84	1	NFATC3
rs8056649	16	68,260,083	C	T	0.885	1	NFATC3
rs74625559	16	68,260,122	G	A	0.924	0.975	NFATC3
rs11043	16	68,262,958	G	A	0.885	0.999	NFATC3
rs9890012	17	15,932,627	T	C	0.466	0.999	NCOR1
rs9916868	17	15,932,820	G	C	0.439	0.999	NCOR1
rs11650682	17	15,933,118	C	A	0.938	0.987	NCOR1
rs147988351	17	15,933,916	C	CTTTCCAAG	0.973	0.997	NCOR1
rs73981417	17	15,935,456	C	T	0.973	0.998	NCOR1
rs8076864	17	15,936,270	A	G	0.467	0.999	NCOR1
rs144845313	17	15,936,552	G	A	0.973	0.998	NCOR1
17:15936650_CA_C	17	15,936,650	CA	C	0.98	0.789	NCOR1
rs10646922	17	15,936,966	G	GTTTTA	0.439	0.998	NCOR1
rs6502488	17	15,937,487	C	A	0.44	0.999	NCOR1
rs9908031	17	15,938,690	G	A	0.467	0.999	NCOR1
rs7224403	17	15,940,525	T	C	0.973	0.998	NCOR1
rs73981421	17	15,940,835	G	A	0.973	0.998	NCOR1
17:15941749_CT_C	17	15,941,749	CT	C	0.438	0.995	NCOR1
rs8080221	17	15,942,085	C	G	0.432	0.999	NCOR1
rs8081406	17	15,942,145	T	C	0.439	0.999	NCOR1
rs191115242	17	15,942,304	C	T	0.989	0.914	NCOR1
17:15942409_TA_T	17	15,942,409	TA	T	0.973	0.998	NCOR1
rs4792716	17	15,943,144	A	G	0.439	0.999	NCOR1
rs11657160	17	15,943,331	G	C	0.439	0.999	NCOR1
rs1075901	17	15,943,910	T	C	0.439	0.999	NCOR1
rs1065822	17	15,944,208	A	T	0.467	0.998	NCOR1
rs12602453	17	15,945,119	T	C	0.866	0.924	NCOR1
rs142141461	17	15,945,156	C	T	0.974	0.981	NCOR1
rs145923295	17	15,945,182	C	G	0.931	0.943	NCOR1
rs62073589	17	15,945,286	G	A	0.97	0.987	NCOR1
rs10684399	17	15,945,474	C	CAAAG	0.443	0.993	NCOR1
rs3785632	17	15,945,608	C	T	0.378	0.996	NCOR1
rs3837816	17	15,945,613	G	GTCAT	0.439	0.997	NCOR1
rs3785631	17	15,945,946	A	G	0.466	0.999	NCOR1
rs4435297	17	15,947,217	T	C	0.439	0.999	NCOR1
rs34691717	17	15,947,482	CA	C	0.966	0.833	NCOR1
rs7218152	17	15,948,322	A	G	0.973	0.998	NCOR1
rs4792717	17	15,948,430	A	G	0.439	1	NCOR1
rs9889822	17	15,948,963	C	T	0.466	1	NCOR1
rs144455669	17	15,948,971	C	T	0.982	0.986	NCOR1
rs9909615	17	15,949,099	C	T	0.466	1	NCOR1
rs1079534	17	15,949,133	T	A	0.44	0.999	NCOR1
rs1079533	17	15,950,198	G	A	0.467	1	NCOR1
rs8067494	17	15,950,751	A	G	0.973	0.999	NCOR1
17:15951008_TA_T	17	15,951,008	TA	T	0.44	0.999	NCOR1
rs73981425	17	15,951,364	G	C	0.973	0.999	NCOR1
rs11651290	17	15,951,471	C	T	0.467	1	NCOR1
rs79040621	17	15,952,093	G	C	0.957	0.975	NCOR1
rs3815039	17	15,952,588	C	A	0.467	1	NCOR1
rs11078327	17	15,952,941	C	G	0.44	1	NCOR1
rs78233018	17	15,953,414	A	C	0.974	0.983	NCOR1
rs186452832	17	15,953,554	T	A	0.955	0.975	NCOR1
rs11459724	17	15,954,048	T	TA	0.439	1	NCOR1
rs8079698	17	15,954,812	G	A	0.973	0.999	NCOR1
rs12945546	17	15,954,906	T	C	0.467	1	NCOR1
rs8065190	17	15,955,009	G	T	0.44	1	NCOR1
rs186014490	17	15,955,186	T	C	0.985	0.909	NCOR1
rs11078328	17	15,955,491	T	A	0.467	1	NCOR1
rs147141256	17	15,955,529	G	A	0.977	0.99	NCOR1
rs11657912	17	15,956,140	G	T	0.467	1	NCOR1
rs11656046	17	15,958,014	T	C	0.439	1	NCOR1
rs12945463	17	15,958,185	A	G	0.439	1	NCOR1
rs4792719	17	15,958,402	A	G	0.466	1	NCOR1
rs9652827	17	15,958,774	C	T	0.44	1	NCOR1
rs3760296	17	15,959,279	A	G	0.971	0.986	NCOR1
17:15959412_GCTT_G	17	15,959,412	GCTT	G	0.468	0.998	NCOR1
rs9652828	17	15,959,714	C	T	0.467	1	NCOR1
rs4791660	17	15,960,190	A	G	0.467	1	NCOR1
rs2157990	17	15,961,993	T	A	0.44	1	NCOR1
rs3785629	17	15,962,121	A	G	0.44	1	NCOR1
rs9709250	17	15,962,151	A	G	0.44	1	NCOR1
rs77616854	17	15,962,966	C	T	0.984	0.975	NCOR1
rs1073093	17	15,963,063	C	T	0.973	0.999	NCOR1
rs1073094	17	15,963,449	T	C	0.973	0.999	NCOR1
rs1073095	17	15,963,465	T	G	0.973	0.999	NCOR1
rs73981431	17	15,965,268	G	A	0.973	0.999	NCOR1
rs754982762	17	15,965,696	AAAAC	A	0.467	0.999	NCOR1
rs11652025	17	15,965,863	T	G	0.439	1	NCOR1
rs137982058	17	15,966,994	T	C	0.989	0.892	NCOR1
rs73981433	17	15,967,338	C	A	0.973	0.999	NCOR1
rs530573051	17	15,967,928	C	CA	0.937	0.955	NCOR1
rs2285583	17	15,968,143	T	C	0.43	1	NCOR1
rs3815038	17	15,968,549	A	C	0.467	1	NCOR1
rs2285582	17	15,968,673	G	A	0.44	1	NCOR1
rs59172522	17	15,969,681	A	C	0.973	0.999	NCOR1
17:15969756_GTCTC_G	17	15,969,756	GTCTC	G	0.973	0.994	NCOR1
rs3785628	17	15,970,682	C	T	0.439	1	NCOR1
rs34727549	17	15,972,029	A	AT	0.494	0.946	NCOR1
rs12948361	17	15,972,081	G	A	0.466	1	NCOR1
rs56375441	17	15,972,308	T	C	0.973	0.998	NCOR1
rs12942295	17	15,973,844	A	T	0.466	1	NCOR1
rs9891379	17	15,976,558	T	G	0.466	1	NCOR1
rs117087658	17	15,976,696	T	C	0.982	0.991	NCOR1
rs11078330	17	15,977,005	A	T	0.466	1	NCOR1
rs9896441	17	15,977,043	C	T	0.439	1	NCOR1
rs9898313	17	15,977,132	T	C	0.466	1	NCOR1
rs73981436	17	15,977,238	G	A	0.973	0.999	NCOR1
17:15977769_CAG_C	17	15,977,769	CAG	C	0.986	0.99	NCOR1
rs11078331	17	15,977,869	T	G	0.439	1	NCOR1
rs150427494	17	15,977,942	G	A	0.975	0.915	NCOR1
rs9905429	17	15,978,404	G	C	0.466	1	NCOR1
rs3760295	17	15,978,663	T	C	0.973	0.999	NCOR1
rs73981438	17	15,980,223	A	G	0.973	0.999	NCOR1
17:15980573_CGGCTGCGCACACCTGTAATTCCAGCTACTT_C	17	15,980,573	CGGCTGCGCACACCTGTAATTCCAGCTACTT	C	0.935	0.902	NCOR1
rs545193792	17	15,980,902	G	A	0.954	0.677	NCOR1
rs113652180	17	15,980,904	G	A	0.954	0.677	NCOR1
rs7223346	17	15,981,874	T	C	0.439	0.999	NCOR1
17:15981894_CT_C	17	15,981,894	CT	C	0.424	0.971	NCOR1
rs112242506	17	15,984,248	A	C	0.976	0.978	NCOR1
rs758387809	17	15,984,528	G	GT	0.466	0.998	NCOR1
rs7214695	17	15,984,544	C	T	0.439	0.999	NCOR1
17:15984696_TA_T	17	15,984,696	TA	T	0.373	0.714	NCOR1

SNP	Chromosome	Position	Effect Allele	Other Allele	Effect Allele Frequency	Imputation quality	Gene
rs73981440	17	15,984,890	T	G	0.973	0.999	NCOR1
rs73981442	17	15,985,249	G	C	0.973	0.999	NCOR1
rs73981443	17	15,985,355	A	G	0.973	0.999	NCOR1
17:15986670_CA_C	17	15,986,670	CA	C	0.437	0.981	NCOR1
rs142477515	17	15,986,887	G	T	0.987	0.92	NCOR1
rs745637108	17	15,987,332	GC	G	0.973	0.994	NCOR1
rs77037672	17	15,987,434	T	C	0.905	0.961	NCOR1
rs118114849	17	15,988,317	A	G	0.99	0.926	NCOR1
rs73981444	17	15,988,617	T	C	0.973	0.999	NCOR1
rs77890400	17	15,988,743	G	C	0.973	0.998	NCOR1
rs2051736	17	15,990,013	T	C	0.469	0.996	NCOR1
rs73981445	17	15,990,445	G	A	0.973	0.999	NCOR1
rs60011951	17	15,990,754	G	A	0.973	0.999	NCOR1
rs56733317	17	15,991,031	T	A	0.973	0.999	NCOR1
17:15991419_TTTTA_T	17	15,991,419	TTTTA	T	0.973	0.998	NCOR1
rs8080706	17	15,991,560	G	C	0.467	0.999	NCOR1
rs8081667	17	15,991,568	T	C	0.431	0.999	NCOR1
rs57825293	17	15,992,430	T	C	0.973	0.999	NCOR1
rs12947205	17	15,993,327	G	A	0.468	0.999	NCOR1
rs1125156	17	15,994,554	G	A	0.467	1	NCOR1
rs34527265	17	15,994,864	T	TCTCA	0.44	0.997	NCOR1
rs2078050	17	15,994,888	C	T	0.44	1	NCOR1
rs8064702	17	15,995,762	C	T	0.44	1	NCOR1
rs72833498	17	15,995,802	T	C	0.471	1	NCOR1
rs7217493	17	15,997,154	C	T	0.968	0.999	NCOR1
rs11871961	17	15,997,426	A	G	0.472	1	NCOR1
rs11872032	17	15,997,773	A	C	0.472	1	NCOR1
rs36001065	17	15,998,172	A	C	0.472	1	NCOR1
rs186444075	17	15,998,563	C	T	0.973	0.999	NCOR1
rs111631027	17	15,998,823	C	T	0.498	0.987	NCOR1
rs8080378	17	15,998,837	A	G	0.468	0.957	NCOR1
rs143493691	17	15,999,045	G	A	0.473	0.997	NCOR1
rs12937434	17	15,999,081	T	C	0.434	0.997	NCOR1
rs191488290	17	15,999,127	G	A	0.982	0.988	NCOR1
rs74580998	17	16,000,192	A	C	0.975	0.983	NCOR1
rs75706055	17	16,001,549	C	T	0.973	0.999	NCOR1
rs73981447	17	16,002,530	G	A	0.973	0.999	NCOR1
rs9913754	17	16,002,708	A	T	0.981	0.987	NCOR1
rs73981450	17	16,003,112	T	C	0.973	1	NCOR1
rs146312731	17	16,003,683	G	A	0.981	0.885	NCOR1
rs7207463	17	16,004,259	A	G	0.44	1	NCOR1
rs7207889	17	16,004,368	C	T	0.472	1	NCOR1
rs62073621	17	16,004,537	G	A	0.855	0.991	NCOR1
rs2285580	17	16,005,187	T	C	0.439	1	NCOR1
rs7219263	17	16,006,703	A	C	0.973	0.997	NCOR1
rs4792721	17	16,006,827	A	G	0.416	0.981	NCOR1
rs7211957	17	16,008,586	T	A	0.969	1	NCOR1
rs181761312	17	16,008,604	G	A	0.987	0.977	NCOR1
rs4791661	17	16,008,708	G	A	0.472	1	NCOR1
rs4791662	17	16,008,872	T	C	0.472	1	NCOR1
rs35550102	17	16,009,751	C	CA	0.492	0.996	NCOR1
rs73981453	17	16,009,842	A	G	0.969	1	NCOR1
rs76275417	17	16,009,915	C	T	0.945	0.999	NCOR1
rs9907273	17	16,010,759	A	G	0.44	1	NCOR1
rs4566208	17	16,010,920	A	G	0.441	1	NCOR1
rs4500784	17	16,011,100	C	G	0.969	1	NCOR1
rs746427605	17	16,011,493	CA	C	0.392	0.89	NCOR1
rs7222886	17	16,012,494	A	T	0.44	1	NCOR1
rs191696194	17	16,012,829	T	G	0.99	0.853	NCOR1
rs111902958	17	16,013,054	A	ATTTTTCTT	0.44	0.999	NCOR1
rs61352879	17	16,013,843	A	G	0.973	1	NCOR1
rs140150945	17	16,013,910	G	A	0.989	0.936	NCOR1
rs76693872	17	16,014,239	T	G	0.984	0.981	NCOR1
rs6502490	17	16,014,865	A	G	0.44	1	NCOR1
17:16015180_ATTTATT_A	17	16,015,180	ATTTATT	A	0.969	0.998	NCOR1
rs1126148	17	16,015,579	G	A	0.472	1	NCOR1
rs143128771	17	16,015,874	A	G	0.955	0.969	NCOR1
rs35160872	17	16,016,039	G	A	0.472	1	NCOR1
rs9905225	17	16,017,072	C	T	0.44	1	NCOR1
rs184004065	17	16,017,682	C	T	0.987	0.98	NCOR1
rs7216043	17	16,017,922	C	T	0.025	0.979	NCOR1
rs7207901	17	16,019,958	G	A	0.973	1	NCOR1
rs9909697	17	16,019,978	G	A	0.472	1	NCOR1
rs71354643	17	16,020,032	C	CAGA	0.439	0.992	NCOR1
rs776548571	17	16,020,828	GA	G	0.484	0.988	NCOR1
rs62073625	17	16,020,856	G	T	0.988	0.72	NCOR1
rs7223409	17	16,021,010	G	A	0.973	1	NCOR1
rs12449895	17	16,021,611	A	G	0.491	1	NCOR1
rs12936657	17	16,022,441	A	T	0.986	0.964	NCOR1
rs12150017	17	16,024,094	T	C	0.472	1	NCOR1
rs11410605	17	16,024,701	C	CA	0.506	0.915	NCOR1
rs11870608	17	16,025,670	A	G	0.44	1	NCOR1
rs11078332	17	16,026,068	A	G	0.472	1	NCOR1
rs75769803	17	16,026,557	T	C	0.982	0.996	NCOR1
rs10852833	17	16,027,361	A	G	0.472	1	NCOR1
rs73981460	17	16,027,419	C	A	0.969	1	NCOR1
rs73981461	17	16,027,504	T	C	0.969	1	NCOR1
rs151245282	17	16,027,917	C	A	0.984	0.98	NCOR1
rs11650678	17	16,028,027	G	A	0.472	1	NCOR1
rs35285459	17	16,029,218	G	A	0.986	0.958	NCOR1
rs73981462	17	16,029,776	C	T	0.969	1	NCOR1
rs113394588	17	16,030,460	T	C	0.976	0.746	NCOR1
rs34243080	17	16,030,490	T	C	0.474	0.967	NCOR1
rs34351630	17	16,030,520	T	C	0.467	1	NCOR1
rs766733998	17	16,030,594	TAC	T	0.48	0.971	NCOR1
rs556030483	17	16,030,596	C	T	0.665	0.805	NCOR1
17:16030622_TAC_T	17	16,030,622	TAC	T	0.973	0.99	NCOR1
rs560845992	17	16,030,625	A	G	0.984	0.737	NCOR1
rs62641986	17	16,030,627	G	A	0.511	0.964	NCOR1
rs563966014	17	16,030,635	A	G	0.987	0.651	NCOR1
rs62650875	17	16,030,683	G	A	0.443	0.993	NCOR1
rs62650876	17	16,030,693	A	G	0.427	0.957	NCOR1
rs147550673	17	16,030,734	C	CAT	0.441	0.995	NCOR1
rs55844265	17	16,030,777	G	A	0.44	1	NCOR1
rs114670500	17	16,030,843	A	G	0.969	0.999	NCOR1
rs116725065	17	16,030,852	C	T	0.973	0.998	NCOR1
rs35375077	17	16,030,866	C	T	0.444	0.99	NCOR1
17:16030883_GTA_G	17	16,030,883	GTA	G	0.975	0.982	NCOR1
rs35799415	17	16,030,900	T	C	0.472	1	NCOR1
rs142902364	17	16,030,983	A	G	0.984	0.88	NCOR1
rs12937088	17	16,031,029	A	G	0.492	0.973	NCOR1
rs12939418	17	16,031,034	T	C	0.472	0.997	NCOR1
rs529318372	17	16,031,073	A	C	0.974	0.991	NCOR1
rs74800269	17	16,031,075	C	A	0.369	0.907	NCOR1
rs4792723	17	16,032,006	T	C	0.969	1	NCOR1
rs73981464	17	16,032,673	T	A	0.973	1	NCOR1
rs7216834	17	16,032,765	C	A	0.472	1	NCOR1
rs1160109	17	16,033,132	T	A	0.472	1	NCOR1
rs2324141	17	16,033,443	C	A	0.441	1	NCOR1
rs9902141	17	16,034,773	G	T	0.472	1	NCOR1
rs10451230	17	16,035,225	A	T	0.441	1	NCOR1
rs58475330	17	16,035,478	G	A	0.969	1	NCOR1
rs57843678	17	16,035,500	T	C	0.969	1	NCOR1
rs7219365	17	16,036,644	G	C	0.973	1	NCOR1
rs9891646	17	16,036,861	T	C	0.472	1	NCOR1
rs7224575	17	16,036,922	T	C	0.973	1	NCOR1
rs9897388	17	16,037,626	C	A	0.441	1	NCOR1
rs145586930	17	16,038,460	G	A	0.973	1	NCOR1
rs762957500	17	16,038,472	CA	C	0.986	0.596	NCOR1
rs1972503	17	16,039,912	G	A	0.476	0.997	NCOR1
rs60918926	17	16,040,099	C	T	0.973	1	NCOR1
rs201314008	17	16,040,135	A	AG	0.043	0.791	NCOR1
rs1986040	17	16,040,139	A	G	0.475	0.992	NCOR1
rs117982001	17	16,041,149	G	A	0.977	0.982	NCOR1
rs2324142	17	16,041,306	C	A	0.44	1	NCOR1
rs2875265	17	16,041,636	T	C	0.44	1	NCOR1
rs6502491	17	16,041,777	C	T	0.44	1	NCOR1
rs145557673	17	16,041,778	A	G	0.981	0.909	NCOR1
rs3760293	17	16,041,792	A	G	0.44	1	NCOR1
rs73981466	17	16,042,681	T	C	0.973	1	NCOR1
rs1989856	17	16,043,651	T	C	0.472	1	NCOR1
rs556258431	17	16,044,126	T	TCAAA	0.982	0.725	NCOR1
rs145936946	17	16,044,927	A	C	0.982	0.996	NCOR1
rs73981467	17	16,045,860	T	C	0.973	1	NCOR1
rs62072469	17	16,046,002	A	T	0.968	0.985	NCOR1
rs188457668	17	16,046,524	C	T	0.988	0.96	NCOR1
rs2285579	17	16,046,845	A	T	0.44	1	NCOR1
rs3214129	17	16,047,179	TTAA	T	0.472	0.998	NCOR1
rs75780247	17	16,048,042	T	C	0.969	0.999	NCOR1
rs532777381	17	16,048,732	G	C	0.901	0.843	NCOR1
rs551481476	17	16,048,733	T	C	0.901	0.843	NCOR1
rs568699741	17	16,048,742	G	GGCA	0.923	0.78	NCOR1
rs115005413	17	16,048,858	C	T	0.973	0.998	NCOR1
rs549916217	17	16,048,912	A	G	0.906	0.828	NCOR1
rs569885135	17	16,048,915	C	A	0.905	0.827	NCOR1

SNP	Chromosome	Position	Effect Allele	Other Allele	Effect Allele Frequency	Imputation quality	Gene
rs9908591	17	16,049,042	T	C	0.44	1	NCOR1
rs9907313	17	16,049,090	G	A	0.472	1	NCOR1
rs76502056	17	16,049,360	T	C	0.973	1	NCOR1
rs183071464	17	16,049,563	C	A	0.971	0.867	NCOR1
rs11078333	17	16,049,626	A	T	0.472	1	NCOR1
rs184310130	17	16,050,488	G	C	0.988	0.928	NCOR1
rs5819557	17	16,050,724	C	CA	0.472	1	NCOR1
17:16050777_GAAGT_G	17	16,050,777	GAAGT	G	0.983	0.861	NCOR1
rs138946195	17	16,051,316	A	G	0.987	0.943	NCOR1
rs8079969	17	16,051,444	C	T	0.44	1	NCOR1
rs9903464	17	16,052,381	C	T	0.476	0.998	NCOR1
rs536204578	17	16,054,019	A	G	0.98	0.717	NCOR1
rs201731396	17	16,055,582	T	TA	0.986	0.984	NCOR1
rs178662	17	16,055,659	G	A	0.472	1	NCOR1
rs178659	17	16,056,442	C	T	0.472	1	NCOR1
rs62072471	17	16,057,037	G	A	0.969	0.977	NCOR1
rs178658	17	16,057,490	G	C	0.472	1	NCOR1
rs178657	17	16,057,933	A	G	0.472	1	NCOR1
rs143462460	17	16,058,485	T	C	0.969	1	NCOR1
17:16058664_CA_C	17	16,058,664	CA	C	0.515	0.941	NCOR1
rs9895875	17	16,058,695	A	C	0.984	0.87	NCOR1
rs16959547	17	16,059,208	C	G	0.973	1	NCOR1
rs73981471	17	16,060,953	C	T	0.973	1	NCOR1
rs79403135	17	16,061,197	T	G	0.989	0.95	NCOR1
rs178656	17	16,061,435	G	T	0.926	0.945	NCOR1
17:16062400_TA_T	17	16,062,400	TA	T	0.443	0.975	NCOR1
rs143637824	17	16,063,304	C	A	0.978	0.929	NCOR1
17:16064595_CTGTTTT_C	17	16,064,595	CTGTTTT	C	0.963	0.869	NCOR1
rs178654	17	16,064,716	A	G	0.44	1	NCOR1
rs150970554	17	16,064,723	T	C	0.969	1	NCOR1
rs178653	17	16,065,477	G	A	0.44	1	NCOR1
rs141137935	17	16,065,811	T	C	0.973	0.876	NCOR1
rs34172004	17	16,067,557	G	GA	0.534	0.852	NCOR1
rs150876737	17	16,068,096	C	T	0.973	1	NCOR1
rs73981477	17	16,070,240	A	G	0.973	1	NCOR1
rs77031368	17	16,070,258	C	T	0.968	0.998	NCOR1
rs558112910	17	16,072,325	A	G	0.977	0.969	NCOR1
rs144170116	17	16,072,582	A	AAAC	0.973	1	NCOR1
rs747472165	17	16,072,585	C	CAAA	0.978	0.918	NCOR1
rs754503049	17	16,072,588	C	CAAG	0.978	0.917	NCOR1
rs747775576	17	16,072,590	A	ACAC	0.978	0.918	NCOR1
rs769476069	17	16,072,594	C	CAAA	0.977	0.915	NCOR1
rs73981482	17	16,072,735	T	C	0.973	1	NCOR1
17:16072953_TA_T	17	16,072,953	TA	T	0.436	0.992	NCOR1
rs178839	17	16,073,871	C	G	0.44	1	NCOR1
rs9898135	17	16,074,041	C	T	0.981	0.998	NCOR1
rs556473992	17	16,074,154	C	CA	0.99	0.607	NCOR1
rs7225813	17	16,074,287	G	C	0.973	1	NCOR1
rs7207630	17	16,074,465	T	C	0.973	1	NCOR1
rs178838	17	16,075,901	A	T	0.44	1	NCOR1
rs178837	17	16,076,898	C	A	0.491	1	NCOR1
rs178836	17	16,076,998	G	C	0.44	1	NCOR1
rs178835	17	16,077,116	T	A	0.441	0.999	NCOR1
rs178834	17	16,077,124	A	T	0.471	0.997	NCOR1
rs7208681	17	16,078,137	G	C	0.973	1	NCOR1
17:16078346_GTA_G	17	16,078,346	GTA	G	0.974	0.983	NCOR1
rs79606155	17	16,078,510	G	A	0.973	1	NCOR1
rs77471309	17	16,078,832	C	T	0.957	0.96	NCOR1
rs178833	17	16,078,836	C	T	0.44	1	NCOR1
rs117595452	17	16,078,981	C	T	0.977	0.977	NCOR1
rs556538040	17	16,079,143	T	A	0.989	0.924	NCOR1
17:16079148_TA_T	17	16,079,148	TA	T	0.616	0.79	NCOR1
rs111780805	17	16,079,465	G	A	0.983	0.914	NCOR1
rs139400668	17	16,079,531	C	T	0.982	0.994	NCOR1
rs7220699	17	16,080,210	T	C	0.973	1	NCOR1
rs8077949	17	16,080,613	T	G	0.973	1	NCOR1
rs178831	17	16,080,849	A	G	0.431	0.999	NCOR1
rs146764392	17	16,081,294	C	T	0.977	0.903	NCOR1
rs199645449	17	16,081,504	C	CA	0.973	1	NCOR1
rs178830	17	16,081,958	C	T	0.491	1	NCOR1
rs6502495	17	16,082,668	A	G	0.973	1	NCOR1
rs112629652	17	16,082,912	C	T	0.974	0.913	NCOR1
rs178829	17	16,083,189	G	A	0.472	1	NCOR1
rs178828	17	16,083,586	C	T	0.44	1	NCOR1
rs178827	17	16,083,666	A	G	0.44	1	NCOR1
rs178826	17	16,083,684	C	T	0.44	1	NCOR1
rs112789985	17	16,084,519	A	G	0.975	0.981	NCOR1
rs568415428	17	16,084,545	G	A	0.977	0.991	NCOR1
rs201366691	17	16,084,569	A	G	0.897	0.687	NCOR1
17:16084704_TA_T	17	16,084,704	TA	T	0.017	0.842	NCOR1
rs118177172	17	16,084,725	A	G	0.973	1	NCOR1
rs3030884	17	16,084,985	A	AACAC	0.471	1	NCOR1
rs145786774	17	16,085,799	C	T	0.973	1	NCOR1
rs178825	17	16,085,830	A	G	0.44	1	NCOR1
rs178824	17	16,086,407	T	A	0.44	1	NCOR1
rs542089140	17	16,086,544	C	CA	0.979	0.961	NCOR1
rs21180	17	16,088,008	C	T	0.491	0.999	NCOR1
rs178821	17	16,088,135	T	C	0.44	1	NCOR1
rs178820	17	16,090,785	A	G	0.472	1	NCOR1
17:16090854_TA_T	17	16,090,854	TA	T	0.455	0.954	NCOR1
rs545755	17	16,091,170	T	C	0.44	1	NCOR1
rs545738	17	16,091,172	C	T	0.472	1	NCOR1
rs549672110	17	16,091,179	A	C	0.989	0.698	NCOR1
rs178818	17	16,091,694	A	G	0.456	0.994	NCOR1
rs178817	17	16,091,794	G	A	0.472	1	NCOR1
rs36022788	17	16,092,477	G	GT	0.472	1	NCOR1
rs178815	17	16,092,929	C	T	0.44	1	NCOR1
17:16093247_ATAAC_A	17	16,093,247	ATAAC	A	0.989	0.946	NCOR1
rs141480389	17	16,093,470	G	A	0.989	0.932	NCOR1
rs178814	17	16,094,155	G	C	0.44	1	NCOR1
rs74718949	17	16,094,233	A	G	0.947	0.989	NCOR1
rs6502496	17	16,094,710	C	A	0.973	1	NCOR1
17:16096024_AT_A	17	16,096,024	AT	A	0.981	0.988	NCOR1
rs140343984	17	16,096,738	G	C	0.973	1	NCOR1
rs178813	17	16,097,070	C	T	0.491	0.999	NCOR1
rs60770606	17	16,097,115	G	A	0.968	1	NCOR1
rs178812	17	16,097,136	G	T	0.445	1	NCOR1
rs178811	17	16,097,144	T	C	0.467	1	NCOR1
rs178810	17	16,097,430	C	T	0.44	1	NCOR1
rs189636761	17	16,097,601	G	T	0.963	0.974	NCOR1
rs35644251	17	16,097,602	T	TAAG	0.439	0.998	NCOR1
17:16098784_CAAAA_C	17	16,098,784	CAAAA	C	0.497	0.952	NCOR1
17:16098792_AAAAG_A	17	16,098,792	AAAAG	A	0.48	0.98	NCOR1
rs178809	17	16,099,773	C	A	0.472	1	NCOR1
rs178808	17	16,099,954	G	A	0.492	0.998	NCOR1
rs146873336	17	16,099,955	C	G	0.977	0.807	NCOR1
17:16100138_CA_C	17	16,100,138	CA	C	0.459	0.956	NCOR1
rs141228006	17	16,100,342	G	GA	0.44	1	NCOR1
rs178807	17	16,100,561	A	G	0.44	1	NCOR1
rs178806	17	16,100,611	G	C	0.472	0.999	NCOR1
rs178805	17	16,101,128	T	C	0.472	1	NCOR1
17:16101671_CAA_C	17	16,101,671	CAA	C	0.465	0.993	NCOR1
rs533909966	17	16,102,528	A	C	0.97	0.764	NCOR1
17:16102530_AAC_A	17	16,102,530	AAC	A	0.536	0.953	NCOR1
rs6502497	17	16,102,530	A	C	0.907	0.789	NCOR1
rs178804	17	16,103,238	G	T	0.472	0.999	NCOR1
rs140472168	17	16,103,339	T	C	0.98	0.949	NCOR1
rs178803	17	16,103,426	C	T	0.472	0.999	NCOR1
rs1971676	17	16,103,765	C	T	0.982	0.993	NCOR1
rs201968953	17	16,105,249	C	CA	0.977	0.961	NCOR1
rs7212326	17	16,105,769	C	T	0.973	1	NCOR1
rs764345007	17	16,106,354	TACACACACAC	T	0.472	0.999	NCOR1
rs140622231	17	16,106,801	T	G	0.975	0.942	NCOR1
rs178802	17	16,107,045	T	A	0.472	0.999	NCOR1
rs138222025	17	16,107,116	G	A	0.98	0.886	NCOR1
17:16107799_ATTC_A	17	16,107,799	ATTC	A	0.973	0.999	NCOR1
rs178801	17	16,108,464	G	A	0.472	1	NCOR1
rs7210264	17	16,108,567	T	C	0.973	0.998	NCOR1
rs178800	17	16,108,926	G	A	0.472	1	NCOR1
rs35190516	17	16,108,957	G	GA	0.445	0.983	NCOR1
rs9897073	17	16,109,675	C	T	0.981	0.99	NCOR1
rs75340982	17	16,111,395	G	A	0.973	0.999	NCOR1
rs191338697	17	16,111,951	G	A	0.982	0.995	NCOR1
rs76346906	17	16,112,929	A	G	0.973	0.999	NCOR1
rs569325583	17	16,112,950	G	GTA	0.583	0.782	NCOR1
rs535819725	17	16,112,967	C	A	0.989	0.887	NCOR1
rs178799	17	16,114,185	A	G	0.472	0.999	NCOR1
rs7212874	17	16,114,457	G	C	0.973	0.999	NCOR1
17:16114576_TA_T	17	16,114,576	TA	T	0.987	0.611	NCOR1
rs78314206	17	16,114,896	A	G	0.973	0.999	NCOR1
rs77644223	17	16,115,268	A	T	0.973	0.999	NCOR1
rs537795184	17	16,116,803	C	CAAAAAA	0.447	0.986	NCOR1
rs116935512	17	16,117,355	T	C	0.977	0.981	NCOR1
rs178798	17	16,117,359	C	A	0.472	0.999	NCOR1
rs116639184	17	16,118,544	C	T	0.973	0.998	NCOR1

SNP	Chromosome	Position	Effect Allele	Other Allele	Effect Allele Frequency	Imputation quality	Gene
rs76607790	17	16,118,651	G	A	0.973	0.998	NCOR1
rs76584199	17	38,466,052	G	A	0.909	0.968	RARA
rs75309102	17	38,466,139	C	T	0.975	0.998	RARA
rs117011100	17	38,468,692	C	T	0.952	0.996	RARA
rs7217852	17	38,470,021	A	G	0.872	0.998	RARA
rs74453681	17	38,470,142	G	C	0.952	0.995	RARA
rs563136944	17	38,470,429	T	G	0.644	0.758	RARA
rs373717398	17	38,470,432	A	G	0.644	0.758	RARA
rs137891066	17	38,470,768	A	C	0.975	0.998	RARA
rs199818699	17	38,471,421	T	C	0.925	0.897	RARA
rs147747703	17	38,472,361	G	C	0.975	0.998	RARA
rs140987324	17	38,472,368	T	G	0.975	0.998	RARA
rs143494424	17	38,474,528	C	T	0.975	0.998	RARA
rs76921025	17	38,474,665	C	G	0.987	0.954	RARA
17:38475259_GTGTTCC_G	17	38,475,259	GTGTTCC	G	0.99	0.884	RARA
rs145488738	17	38,477,358	G	A	0.981	0.958	RARA
rs77015611	17	38,478,118	G	A	0.975	0.997	RARA
rs182924104	17	38,478,450	T	C	0.967	0.969	RARA
rs9904270	17	38,479,831	C	T	0.898	0.997	RARA
rs55656553	17	38,480,006	G	C	0.898	0.992	RARA
rs192512602	17	38,480,095	G	T	0.975	0.995	RARA
rs12946680	17	38,480,657	C	G	0.872	0.997	RARA
rs143850116	17	38,481,274	G	A	0.976	0.983	RARA
rs180735555	17	38,481,288	G	A	0.984	0.941	RARA
rs36102671	17	38,481,361	G	A	0.904	0.976	RARA
rs56124263	17	38,482,207	C	T	0.933	0.991	RARA
rs190676187	17	38,482,766	T	C	0.975	0.997	RARA
rs77209292	17	38,484,060	C	T	0.975	0.997	RARA
rs78275281	17	38,484,328	G	A	0.986	0.961	RARA
rs140872469	17	38,485,025	G	A	0.975	0.997	RARA
rs118082529	17	38,485,154	C	T	0.989	0.991	RARA
17:38485389_CT_C	17	38,485,389	CT	C	0.876	0.932	RARA
rs9896312	17	38,486,001	C	T	0.915	0.996	RARA
rs138410713	17	38,486,635	G	C	0.983	0.955	RARA
rs117494298	17	38,487,817	A	G	0.975	0.997	RARA
rs35286757	17	38,488,539	A	G	0.872	0.996	RARA
rs2715554	17	38,489,170	A	G	0.85	1	RARA
rs140235616	17	38,489,659	A	G	0.975	0.997	RARA
rs145108176	17	38,490,139	T	G	0.975	0.997	RARA
rs535798423	17	38,492,807	A	AT	0.973	0.986	RARA
rs12051734	17	38,494,150	C	T	0.897	0.995	RARA
rs139444391	17	38,494,581	G	A	0.989	0.989	RARA
rs77783454	17	38,494,861	G	C	0.898	0.995	RARA
rs141223821	17	38,494,886	G	C	0.975	0.996	RARA
rs2957323	17	38,495,150	A	G	0.946	0.938	RARA
rs2715553	17	38,496,320	G	A	0.459	0.993	RARA
rs59406133	17	38,496,752	T	A	0.898	0.995	RARA
rs74814495	17	38,499,761	C	T	0.984	0.988	RARA
rs9303285	17	38,500,914	T	C	0.872	0.994	RARA
rs9303286	17	38,500,928	G	C	0.898	0.994	RARA
rs112509970	17	38,500,946	C	T	0.984	0.963	RARA
rs145105278	17	38,501,982	G	A	0.975	0.995	RARA
rs78200580	17	38,504,260	G	A	0.979	0.896	RARA
rs33998939	17	38,506,301	C	T	0.975	0.998	RARA
rs35898909	17	38,506,374	C	T	0.975	0.998	RARA
rs9894845	17	38,506,457	T	C	0.946	0.995	RARA
rs186956796	17	38,506,458	G	A	0.975	0.998	RARA
rs34627238	17	38,507,072	A	AGTG	0.946	0.989	RARA
rs143915804	17	38,507,250	G	A	0.983	0.952	RARA
17:38507378_ACTCTCC_A	17	38,507,378	ACTCTCC	A	0.977	0.957	RARA
rs182610022	17	38,507,681	T	C	0.975	0.998	RARA
rs4890109	17	38,508,104	G	T	0.956	0.966	RARA
rs41283421	17	38,508,756	C	T	0.981	0.991	RARA
rs35783632	17	38,508,968	G	T	0.975	0.999	RARA
rs41283423	17	38,511,963	C	T	0.975	0.998	RARA
rs8069541	17	38,512,197	T	C	0.946	0.995	RARA
rs2229773	17	38,512,480	C	T	0.975	0.998	RARA
rs72829861	17	46,652,885	C	A	0.939	0.995	HOXB4
rs200016818	17	46,653,037	AT	A	0.92	0.978	HOXB4
rs201603635	17	46,653,038	T	A	0.937	0.97	HOXB4
17:46653080_CATA_C	17	46,653,080	CATA	C	0.918	0.989	HOXB4
17:46653783_CTCTT_C	17	46,653,783	CTCTT	C	0.939	0.992	HOXB4
rs3833172	17	46,653,871	T	TG	0.423	0.993	HOXB4
rs2740755	17	46,653,999	G	A	0.917	0.994	HOXB4
rs3744776	17	46,654,430	G	A	0.983	0.99	HOXB4
rs756761454	17	46,654,512	GC	G	0.09	0.993	HOXB4
rs7406324	17	46,654,947	T	C	0.09	0.996	HOXB4
rs114727888	17	46,655,081	G	A	0.983	0.99	HOXB4
rs151227265	17	46,655,142	A	AC	0.983	0.978	HOXB4
rs201040786	17	46,655,364	G	A	0.967	0.894	HOXB4
rs118023735	17	47,866,526	G	T	0.988	0.95	KAT7
rs9903642	17	47,868,921	G	A	0.966	0.982	KAT7
17:47870935_AT_A	17	47,870,935	AT	A	0.8	0.839	KAT7
rs17713809	17	47,872,001	G	A	0.961	0.986	KAT7
rs8081972	17	47,872,517	C	G	0.829	0.997	KAT7
rs16948378	17	47,872,822	G	A	0.879	0.995	KAT7
17:47873340_TA_T	17	47,873,340	TA	T	0.83	0.992	KAT7
17:47873413_AT_A	17	47,873,413	AT	A	0.116	0.997	KAT7
rs55971006	17	47,874,955	C	T	0.988	0.948	KAT7
rs201181776	17	47,876,151	TC	T	0.779	0.991	KAT7
rs530756603	17	47,877,372	C	CT	0.165	0.584	KAT7
rs7502494	17	47,878,914	C	T	0.338	0.996	KAT7
rs16948381	17	47,880,427	G	C	0.931	0.997	KAT7
rs185678050	17	47,880,948	G	A	0.988	0.966	KAT7
rs528777338	17	47,881,740	C	A	0.928	0.67	KAT7
rs112480726	17	47,881,803	G	A	0.952	0.995	KAT7
rs11079877	17	47,881,828	A	C	0.117	0.998	KAT7
rs113968716	17	47,884,002	A	G	0.988	0.918	KAT7
rs111754564	17	47,884,078	G	A	0.988	0.923	KAT7
rs117024504	17	47,885,588	A	G	0.971	0.981	KAT7
rs112316627	17	47,886,600	C	T	0.985	0.962	KAT7
rs16948388	17	47,887,176	C	T	0.931	0.996	KAT7
rs76301358	17	47,887,443	G	A	0.847	0.991	KAT7
rs9910685	17	47,887,705	A	G	0.117	0.998	KAT7
rs117806575	17	47,888,315	C	A	0.974	0.934	KAT7
rs35069050	17	47,889,596	C	CT	0.093	0.576	KAT7
rs59814289	17	47,890,322	C	T	0.829	0.997	KAT7
rs35886652	17	47,890,326	G	A	0.912	0.918	KAT7
rs7213050	17	47,891,201	C	T	0.94	0.987	KAT7
rs755736	17	47,891,904	A	G	0.338	0.996	KAT7
rs137978710	17	47,892,055	C	A	0.973	0.988	KAT7
rs906423	17	47,892,230	G	A	0.952	0.994	KAT7
17:47893780_ATATT_A	17	47,893,780	ATATT	A	0.491	0.987	KAT7
rs139572894	17	47,895,065	G	A	0.985	0.868	KAT7
17:47896104_GT_G	17	47,896,104	GT	G	0.097	0.883	KAT7
rs186677299	17	47,897,325	G	A	0.988	0.975	KAT7
rs539596857	17	47,897,605	C	CA	0.963	0.927	KAT7
rs118013804	17	47,897,930	C	T	0.988	0.954	KAT7
rs12449890	17	47,898,147	G	T	0.338	0.995	KAT7
rs12941596	17	47,900,880	A	G	0.962	0.979	KAT7
rs4794066	17	47,901,294	C	G	0.821	0.996	KAT7
rs3785928	17	47,903,146	G	A	0.964	0.989	KAT7
rs61749929	17	47,903,381	C	T	0.879	0.995	KAT7
rs544682665	17	47,907,247	G	GT	0.823	0.941	KAT7
rs9898183	17	47,907,383	G	C	0.964	0.989	KAT7
rs7221118	17	47,907,641	T	C	0.786	0.995	KAT7
rs7225180	17	47,909,054	G	T	0.964	0.989	KAT7
rs139220653	17	47,909,453	G	A	0.986	0.952	KAT7
rs79510689	17	47,910,882	A	T	0.98	0.992	KAT7
rs35126550	17	70,118,699	G	A	0.539	0.993	SOX9
rs12950966	17	70,119,245	C	G	0.537	0.993	SOX9
rs929651	17	70,120,111	G	A	0.976	0.972	SOX9
rs1042667	17	70,120,551	A	C	0.531	0.993	SOX9
rs1042673	17	70,121,339	A	G	0.509	0.992	SOX9
rs74999341	17	70,121,454	T	C	0.99	0.968	SOX9
rs73354570	17	70,121,457	A	C	0.987	0.984	SOX9
rs1042678	17	70,122,108	G	A	0.531	0.994	SOX9
rs796209434	17	70,122,505	CT	C	0.523	0.973	SOX9
rs28610978	18	19,750,068	G	C	0.977	0.982	GATA6
rs143674545	18	19,750,094	T	A	0.974	0.925	GATA6
rs140398105	18	19,753,533	G	A	0.981	0.909	GATA6
rs62092221	18	19,754,505	T	C	0.989	0.723	GATA6
rs75527379	18	19,755,872	C	T	0.965	0.981	GATA6
rs3764504	18	19,756,856	C	T	0.958	0.945	GATA6
rs55958737	18	19,757,925	T	G	0.969	0.733	GATA6
rs12960498	18	19,760,649	T	A	0.961	0.907	GATA6
rs9949157	18	19,760,650	T	A	0.484	0.864	GATA6
rs117350956	18	19,762,607	C	T	0.977	0.899	GATA6
rs138042365	18	19,768,194	A	G	0.974	0.915	GATA6
rs146405465	18	19,771,325	C	T	0.98	0.901	GATA6
rs17730399	18	19,773,747	G	A	0.981	0.908	GATA6
rs752762827	18	19,777,041	C	CT	0.724	0.782	GATA6
rs919049	18	19,777,281	A	T	0.042	0.903	GATA6
rs11662020	18	19,777,391	G	C	0.888	0.88	GATA6
rs10454095	18	19,777,440	T	C	0.322	0.821	GATA6

SNP	Chromosome	Position	Effect Allele	Other Allele	Effect Allele Frequency	Imputation quality	Gene
rs763012878	18	19,777,756	CTCCTTCTCA	C	0.983	0.866	GATA6
rs142907786	18	19,777,839	G	A	0.973	0.841	GATA6
18:19778026_CTCTT_C	18	19,778,026	CTCTT	C	0.867	0.9	GATA6
18:19778033_TTTC_T	18	19,778,033	TTTC	T	0.867	0.9	GATA6
rs202022105	18	19,778,036	CT	C	0.632	0.939	GATA6
rs8090509	18	19,778,179	T	G	0.668	0.989	GATA6
rs35737016	18	19,778,223	G	A	0.966	0.947	GATA6
rs35886787	18	19,778,313	A	C	0.872	0.99	GATA6
rs2385472	18	19,779,637	A	G	0.599	0.997	GATA6
rs2385473	18	19,779,662	T	C	0.468	0.992	GATA6
rs4800368	18	19,779,771	A	T	0.929	0.985	GATA6
rs548304091	18	19,780,162	T	C	0.989	0.737	GATA6
rs8094980	18	19,780,169	G	A	0.929	0.984	GATA6
rs1941084	18	19,780,858	G	A	0.545	0.959	GATA6
rs1941083	18	19,780,863	A	G	0.863	0.985	GATA6
rs2303040	19	39,138,608	T	C	0.508	0.994	ACTN4
rs10424568	19	39,138,948	A	G	0.416	0.995	ACTN4
rs62121765	19	39,139,417	G	A	0.981	0.989	ACTN4
rs143072521	19	39,139,761	C	T	0.978	0.994	ACTN4
rs796469796	19	39,139,912	TG	T	0.932	0.988	ACTN4
rs12977599	19	39,139,987	G	A	0.933	0.997	ACTN4
rs732135	19	39,141,974	C	T	0.933	0.995	ACTN4
rs78871891	19	39,142,209	G	A	0.985	0.965	ACTN4
rs752067	19	39,142,329	C	T	0.29	0.995	ACTN4
rs113293049	19	39,142,391	T	C	0.961	0.956	ACTN4
rs73032668	19	39,142,688	G	A	0.871	0.993	ACTN4
rs117477533	19	39,142,928	G	A	0.978	0.996	ACTN4
rs13344413	19	39,143,289	G	A	0.528	0.995	ACTN4
rs3033329	19	39,143,725	C	CA	0.553	0.904	ACTN4
rs12460849	19	39,143,884	G	A	0.509	0.996	ACTN4
rs979972	19	39,144,167	A	G	0.508	0.997	ACTN4
rs16972761	19	39,144,189	A	T	0.978	0.995	ACTN4
rs979971	19	39,144,244	C	T	0.51	0.996	ACTN4
rs888997	19	39,144,714	C	A	0.25	0.998	ACTN4
19:39144736_TC_T	19	39,144,736	TC	T	0.933	0.996	ACTN4
rs138116138	19	39,145,158	C	A	0.978	0.997	ACTN4
rs750236909	19	39,145,201	C	CA	0.958	0.729	ACTN4
rs35488280	19	39,145,577	G	A	0.933	0.998	ACTN4
rs12984295	19	39,146,447	C	T	0.982	0.981	ACTN4
rs55876653	19	39,146,780	G	C	0.508	0.998	ACTN4
rs189809900	19	39,147,164	A	G	0.982	0.913	ACTN4
19:39147786_AT_A	19	39,147,786	AT	A	0.89	0.834	ACTN4
rs148097547	19	39,148,048	G	T	0.978	0.997	ACTN4
rs56365761	19	39,148,103	A	G	0.507	0.998	ACTN4
rs186397237	19	39,148,282	C	A	0.978	0.997	ACTN4
rs117415439	19	39,148,461	A	G	0.978	0.997	ACTN4
rs12980438	19	39,149,705	C	T	0.585	0.72	ACTN4
rs7351086	19	39,150,199	A	G	0.823	0.998	ACTN4
rs10415219	19	39,150,235	A	G	0.515	0.998	ACTN4
rs796496657	19	39,150,561	C	CT	0.701	0.855	ACTN4
rs888996	19	39,150,622	G	T	0.293	0.996	ACTN4
rs1808382	19	39,151,034	G	T	0.516	0.973	ACTN4
rs62121811	19	39,151,182	C	T	0.93	0.997	ACTN4
rs12462503	19	39,152,004	G	C	0.293	0.997	ACTN4
rs112979658	19	39,152,394	G	A	0.945	0.991	ACTN4
rs12977238	19	39,152,530	T	G	0.93	0.998	ACTN4
rs41482151	19	39,152,743	C	G	0.825	0.996	ACTN4
rs16972767	19	39,153,044	G	A	0.515	0.999	ACTN4
rs75544435	19	39,153,265	A	G	0.978	0.996	ACTN4
rs17665451	19	39,153,307	T	A	0.826	0.998	ACTN4
rs79232931	19	39,153,311	G	A	0.981	0.969	ACTN4
rs8112875	19	39,153,479	G	C	0.989	0.946	ACTN4
rs377341870	19	39,153,549	G	GT	0.807	0.906	ACTN4
rs3786835	19	39,155,880	G	A	0.516	0.998	ACTN4
rs888995	19	39,156,138	C	T	0.871	0.995	ACTN4
rs73032691	19	39,156,382	A	G	0.871	0.995	ACTN4
rs3810341	19	39,156,481	C	T	0.387	0.996	ACTN4
rs117087613	19	39,156,513	C	T	0.978	0.995	ACTN4
rs7246368	19	39,157,599	C	T	0.93	0.999	ACTN4
rs574289358	19	39,158,346	C	CA	0.558	0.86	ACTN4
rs1985636	19	39,159,278	G	A	0.294	0.996	ACTN4
rs572687238	19	39,159,383	C	CAG	0.516	0.97	ACTN4
rs117784702	19	39,159,686	G	A	0.987	0.927	ACTN4
rs62121814	19	39,159,729	C	T	0.516	0.998	ACTN4
rs111779128	19	39,159,794	T	C	0.953	0.979	ACTN4
rs28377909	19	39,161,438	T	C	0.438	0.998	ACTN4
19:39161561_TA_T	19	39,161,561	TA	T	0.76	0.739	ACTN4
rs6508813	19	39,162,230	A	C	0.24	0.999	ACTN4
rs7260070	19	39,162,435	C	T	0.93	1	ACTN4
rs8182484	19	39,162,800	C	A	0.826	0.999	ACTN4
rs138414242	19	39,162,919	C	T	0.972	0.997	ACTN4
rs2112651	19	39,163,005	T	C	0.24	0.999	ACTN4
rs73032697	19	39,163,197	A	G	0.826	0.999	ACTN4
rs11670636	19	39,163,443	A	C	0.415	0.999	ACTN4
rs34083318	19	39,163,969	T	C	0.93	1	ACTN4
rs80037173	19	39,164,081	T	A	0.978	0.987	ACTN4
rs8182571	19	39,164,167	T	G	0.933	0.959	ACTN4
rs8182552	19	39,164,168	C	A	0.933	0.959	ACTN4
rs549326584	19	39,164,314	T	TTA	0.409	0.975	ACTN4
rs573802176	19	39,164,381	A	G	0.986	0.505	ACTN4
19:39164393_ATT_A	19	39,164,393	ATT	A	0.515	0.994	ACTN4
rs62121816	19	39,164,464	C	T	0.516	0.998	ACTN4
rs34525537	19	39,164,715	C	T	0.93	0.999	ACTN4
rs35691365	19	39,165,062	G	T	0.93	1	ACTN4
rs34558140	19	39,165,407	C	T	0.93	1	ACTN4
rs5828014	19	39,166,779	C	CG	0.93	0.996	ACTN4
rs2368476	19	39,167,141	G	T	0.871	0.994	ACTN4
rs62121818	19	39,167,760	T	C	0.51	0.999	ACTN4
19:39168010_AACAG_A	19	39,168,010	AACAG	A	0.516	0.995	ACTN4
rs145484023	19	39,168,251	A	AGCTGACCCTACACTAAGCT	0.295	0.987	ACTN4
rs67953948	19	39,170,029	C	T	0.871	0.994	ACTN4
rs10406778	19	39,170,473	A	G	0.509	0.999	ACTN4
rs11403419	19	39,170,499	G	GA	0.827	0.997	ACTN4
rs60687267	19	39,170,856	T	G	0.509	0.998	ACTN4
rs7252589	19	39,170,908	G	A	0.509	0.999	ACTN4
rs62121819	19	39,171,104	A	G	0.929	0.999	ACTN4
rs73933041	19	39,171,999	C	T	0.978	0.998	ACTN4
rs3786837	19	39,172,042	G	C	0.528	0.995	ACTN4
rs8103530	19	39,172,220	A	G	0.929	0.999	ACTN4
rs115060312	19	39,172,234	C	T	0.975	0.997	ACTN4
rs117202080	19	39,172,509	T	C	0.978	0.997	ACTN4
rs7254100	19	39,173,486	A	C	0.516	0.999	ACTN4
rs117044606	19	39,173,555	C	T	0.972	0.994	ACTN4
rs34695688	19	39,173,645	T	C	0.93	0.999	ACTN4
rs12981194	19	39,174,097	C	T	0.957	0.992	ACTN4
rs973010	19	39,174,401	A	G	0.011	0.955	ACTN4
rs77129448	19	39,174,916	G	A	0.978	0.995	ACTN4
rs62121821	19	39,175,617	C	T	0.935	0.999	ACTN4
rs150193161	19	39,176,864	C	T	0.95	0.939	ACTN4
19:39177865_GCC_GC	19	39,177,865	GCC	GC	0.935	0.999	ACTN4
rs62121823	19	39,178,481	G	A	0.934	0.999	ACTN4
rs78400579	19	39,178,653	G	A	0.941	0.939	ACTN4
rs139032698	19	39,178,959	C	T	0.978	0.997	ACTN4
rs747499172	19	39,179,030	CAAAA	C	0.883	0.974	ACTN4
rs35074956	19	39,179,092	T	G	0.934	0.999	ACTN4
rs34592527	19	39,179,142	G	C	0.934	0.999	ACTN4
rs13343794	19	39,179,271	G	A	0.512	0.999	ACTN4
rs35941349	19	39,179,279	A	G	0.934	0.999	ACTN4
rs138810803	19	39,179,372	C	CA	0.825	0.996	ACTN4
rs35685980	19	39,179,839	T	G	0.934	0.999	ACTN4
rs11083473	19	39,179,934	A	G	0.446	0.999	ACTN4
rs10415646	19	39,180,458	C	T	0.512	0.999	ACTN4
rs34126343	19	39,181,245	T	C	0.934	0.999	ACTN4
rs62121827	19	39,181,314	C	T	0.934	0.999	ACTN4
rs62121828	19	39,181,369	T	G	0.88	0.985	ACTN4
rs74878936	19	39,181,691	G	A	0.934	0.999	ACTN4
rs7258742	19	39,181,778	G	A	0.804	0.999	ACTN4
rs4347742	19	39,181,912	A	G	0.242	0.999	ACTN4
rs62121829	19	39,181,972	C	T	0.934	0.999	ACTN4
rs117985824	19	39,181,977	G	T	0.978	0.997	ACTN4
rs34653447	19	39,182,300	T	C	0.981	0.987	ACTN4
rs3786839	19	39,182,565	C	T	0.507	0.999	ACTN4
rs3786840	19	39,182,692	T	A	0.934	0.999	ACTN4
rs60454376	19	39,182,899	T	C	0.934	0.999	ACTN4
rs117851231	19	39,183,514	C	T	0.978	0.997	ACTN4
rs35716073	19	39,183,584	A	G	0.934	0.999	ACTN4
rs73034621	19	39,183,779	G	A	0.826	0.999	ACTN4
rs56113315	19	39,184,166	C	T	0.513	0.999	ACTN4
rs34368672	19	39,184,170	T	G	0.934	0.998	ACTN4
rs34843929	19	39,184,360	C	T	0.934	0.999	ACTN4
rs34105297	19	39,184,669	C	G	0.931	0.991	ACTN4
rs7251903	19	39,184,811	G	A	0.515	0.999	ACTN4
rs7252630	19	39,184,822	T	G	0.249	0.999	ACTN4
rs563718301	19	39,184,904	C	CT	0.82	0.983	ACTN4
rs34150721	19	39,185,519	C	CG	0.934	0.999	ACTN4

SNP	Chromosome	Position	Effect Allele	Other Allele	Effect Allele Frequency	Imputation quality	Gene
rs2080988	19	39,185,557	G	C	0.242	0.999	ACTN4
rs3786841	19	39,186,229	T	C	0.242	0.999	ACTN4
rs10421560	19	39,186,613	G	A	0.507	0.998	ACTN4
rs10426809	19	39,186,950	A	G	0.439	1	ACTN4
rs12971309	19	39,187,004	C	A	0.934	0.999	ACTN4
rs34862518	19	39,187,375	A	T	0.934	0.999	ACTN4
rs3786843	19	39,187,385	A	G	0.242	0.999	ACTN4
rs34899634	19	39,187,479	A	G	0.934	0.999	ACTN4
rs779187281	19	39,187,746	AAGCCAG	A	0.934	0.999	ACTN4
rs4802743	19	39,188,064	C	G	0.242	0.999	ACTN4
rs4802744	19	39,188,149	A	G	0.803	0.999	ACTN4
rs62119101	19	39,188,236	G	T	0.934	0.999	ACTN4
rs62119102	19	39,188,301	G	A	0.934	0.997	ACTN4
rs62119103	19	39,188,316	C	T	0.934	0.999	ACTN4
rs57230275	19	39,188,731	C	T	0.934	0.999	ACTN4
rs138542012	19	39,189,224	A	G	0.983	0.971	ACTN4
rs141515562	19	39,189,367	A	G	0.981	0.986	ACTN4
rs749701	19	39,189,746	T	C	0.416	1	ACTN4
rs749702	19	39,189,895	C	T	0.901	0.992	ACTN4
rs12981984	19	39,189,961	G	A	0.934	1	ACTN4
rs62119105	19	39,190,267	C	T	0.934	1	ACTN4
rs35576862	19	39,190,431	A	G	0.934	1	ACTN4
rs56946356	19	39,190,463	A	G	0.934	0.999	ACTN4
rs4802747	19	39,190,588	C	T	0.826	0.999	ACTN4
rs112084615	19	39,191,051	C	T	0.982	0.942	ACTN4
rs77307137	19	39,191,323	C	T	0.978	0.997	ACTN4
rs2112649	19	39,191,383	G	C	0.242	0.999	ACTN4
rs73038909	19	39,191,544	G	A	0.826	0.999	ACTN4
rs140381330	19	39,191,733	C	T	0.983	0.967	ACTN4
rs74832972	19	39,193,690	C	T	0.978	0.996	ACTN4
rs11452623	19	39,194,045	A	AT	0.935	0.989	ACTN4
rs118144110	19	39,194,170	C	A	0.978	0.997	ACTN4
rs1123300	19	39,194,182	G	A	0.935	0.993	ACTN4
rs191623045	19	39,194,599	C	T	0.98	0.908	ACTN4
rs61296025	19	39,195,015	C	T	0.978	0.997	ACTN4
rs3786845	19	39,195,083	C	G	0.514	0.999	ACTN4
rs35436155	19	39,195,167	G	A	0.934	1	ACTN4
rs757940594	19	39,195,302	AGGAG	A	0.514	0.996	ACTN4
rs2287729	19	39,195,314	T	G	0.514	0.996	ACTN4
rs2287728	19	39,195,558	C	T	0.934	1	ACTN4
rs2368475	19	39,195,735	C	T	0.978	0.996	ACTN4
rs141476190	19	39,195,795	G	A	0.977	0.992	ACTN4
rs117124256	19	39,196,025	C	G	0.969	0.971	ACTN4
rs17589359	19	39,196,085	A	G	0.934	0.999	ACTN4
rs34332511	19	39,196,207	C	T	0.934	1	ACTN4
rs62119109	19	39,196,224	G	A	0.934	0.999	ACTN4
rs11665964	19	39,196,328	C	T	0.425	0.999	ACTN4
rs17589415	19	39,196,531	C	T	0.825	0.999	ACTN4
rs11553600	19	39,196,736	G	A	0.824	0.999	ACTN4
rs3745859	19	39,196,745	C	T	0.514	0.999	ACTN4
rs35729716	19	39,197,102	A	G	0.934	1	ACTN4
rs8104815	19	39,197,310	C	T	0.934	0.999	ACTN4
rs8105055	19	39,197,502	C	T	0.934	1	ACTN4
rs8105069	19	39,197,527	C	G	0.934	1	ACTN4
rs8105080	19	39,197,552	C	G	0.934	1	ACTN4
rs6508814	19	39,197,585	A	G	0.242	0.999	ACTN4
rs8105216	19	39,197,729	A	G	0.934	1	ACTN4
rs8105550	19	39,197,773	G	A	0.934	1	ACTN4
rs35856174	19	39,197,954	T	C	0.934	1	ACTN4
rs61432695	19	39,197,984	C	T	0.978	0.997	ACTN4
rs12975256	19	39,198,320	A	G	0.934	1	ACTN4
rs34082501	19	39,199,387	T	G	0.934	1	ACTN4
rs8099948	19	39,199,621	T	C	0.249	0.998	ACTN4
19:39199746_AG_A	19	39,199,746	AG	A	0.978	0.992	ACTN4
rs56737532	19	39,199,980	G	T	0.978	0.991	ACTN4
rs45561939	19	39,199,998	G	A	0.978	0.991	ACTN4
rs58197765	19	39,200,001	G	T	0.978	0.991	ACTN4
rs12984794	19	39,200,843	C	T	0.934	0.999	ACTN4
rs77100563	19	39,201,274	C	G	0.824	0.999	ACTN4
rs12462270	19	39,201,488	C	T	0.514	0.999	ACTN4
rs2279144	19	39,201,781	C	T	0.514	0.999	ACTN4
rs2279145	19	39,201,816	T	C	0.934	1	ACTN4
rs73038918	19	39,202,056	C	T	0.825	0.999	ACTN4
rs899199	19	39,202,071	G	A	0.934	1	ACTN4
rs899200	19	39,202,184	G	A	0.934	0.999	ACTN4
rs8103824	19	39,202,341	G	A	0.825	0.999	ACTN4
rs2279146	19	39,202,617	C	G	0.934	1	ACTN4
rs565261907	19	39,202,886	A	AG	0.935	0.989	ACTN4
rs28488032	19	39,202,888	G	C	0.498	0.981	ACTN4
rs28624276	19	39,202,897	G	A	0.507	0.997	ACTN4
rs779788411	19	39,203,589	CTGGCCCCTCTCTGAGGGGGTAG	C	0.938	0.959	ACTN4
rs79001257	19	39,203,750	C	G	0.964	0.963	ACTN4
rs560826891	19	39,204,237	G	A	0.987	0.932	ACTN4
rs117849076	19	39,204,694	G	C	0.978	0.996	ACTN4
rs35666464	19	39,204,845	C	T	0.934	0.999	ACTN4
rs10403540	19	39,204,846	G	A	0.514	0.998	ACTN4
rs73038924	19	39,204,961	G	T	0.825	0.999	ACTN4
rs117968873	19	39,204,981	T	A	0.952	0.992	ACTN4
rs2306196	19	39,205,015	C	T	0.945	0.975	ACTN4
rs2086148	19	39,205,607	G	A	0.934	0.999	ACTN4
rs883394	19	39,205,613	C	T	0.516	0.998	ACTN4
rs883433	19	39,206,288	C	T	0.514	0.999	ACTN4
rs144543907	19	39,206,686	C	T	0.978	0.996	ACTN4
rs67438539	19	39,207,234	G	A	0.825	1	ACTN4
rs3786848	19	39,207,329	C	T	0.514	0.999	ACTN4
rs746461041	19	39,207,498	CAA	C	0.934	0.996	ACTN4
rs185752145	19	39,207,582	G	C	0.984	0.966	ACTN4
rs118095019	19	39,208,213	G	C	0.978	0.996	ACTN4
rs7248577	19	39,208,795	C	T	0.934	1	ACTN4
rs73038930	19	39,209,286	G	A	0.825	1	ACTN4
rs12978715	19	39,209,287	A	C	0.934	1	ACTN4
rs35640008	19	39,209,560	C	A	0.934	1	ACTN4
rs1031849	19	39,209,888	C	G	0.249	0.998	ACTN4
rs73038932	19	39,210,013	G	A	0.825	1	ACTN4
rs112235278	19	39,210,223	G	A	0.961	0.949	ACTN4
rs62119116	19	39,210,673	C	T	0.934	1	ACTN4
rs73038936	19	39,210,733	C	T	0.825	1	ACTN4
rs1564203	19	39,210,961	A	G	0.241	0.998	ACTN4
rs34026184	19	39,212,052	T	C	0.934	1	ACTN4
rs77822825	19	39,212,392	C	T	0.824	1	ACTN4
rs8111746	19	39,212,859	C	T	0.514	0.999	ACTN4
19:39213117_AG_A	19	39,213,117	AG	A	0.517	0.994	ACTN4
rs2126970	19	39,213,187	A	G	0.934	1	ACTN4
rs34717167	19	39,213,252	A	G	0.934	1	ACTN4
rs73933052	19	39,213,256	C	T	0.978	0.996	ACTN4
rs62120068	19	39,214,411	C	T	0.934	1	ACTN4
rs12986337	19	39,215,172	T	C	0.934	1	ACTN4
rs145474119	19	39,215,193	G	A	0.989	0.91	ACTN4
rs3786851	19	39,215,333	C	T	0.514	0.999	ACTN4
rs12985558	19	39,215,539	C	T	0.934	1	ACTN4
rs34563817	19	39,215,608	A	G	0.934	1	ACTN4
rs781269177	19	39,215,724	ATTAC	A	0.934	1	ACTN4
rs34134906	19	39,216,191	G	A	0.934	1	ACTN4
rs12979947	19	39,216,203	T	C	0.934	1	ACTN4
rs139207522	19	39,216,849	G	A	0.985	0.869	ACTN4
rs1105759	19	39,217,507	G	A	0.934	1	ACTN4
rs3214908	19	39,218,277	CG	C	0.849	0.984	ACTN4
rs12980398	19	39,218,394	A	G	0.934	1	ACTN4
rs12980399	19	39,218,395	A	C	0.934	1	ACTN4
rs12981131	19	39,218,694	A	G	0.934	1	ACTN4
rs12975079	19	39,219,124	T	G	0.936	0.97	ACTN4
rs79947842	19	39,219,274	C	T	0.918	0.946	ACTN4
rs12974733	19	39,219,560	G	T	0.934	1	ACTN4
rs1136956	19	39,219,780	T	C	0.758	1	ACTN4
rs10034	19	39,220,708	G	A	0.979	0.994	ACTN4
19:39220801_AC_A	19	39,220,801	AC	A	0.986	0.779	ACTN4
rs117152229	19	39,221,189	T	A	0.981	0.988	ACTN4
rs1060186	19	39,221,295	A	G	0.239	0.996	ACTN4
rs748134757	19	39,221,715	CCCATGCCCCA	C	0.822	0.995	ACTN4
rs200530826	19	39,221,828	T	G	0.947	0.501	ACTN4
rs4801859	19	39,221,947	A	T	0.801	0.999	ACTN4
rs140123622	20	31,408,058	T	TGGG	0.424	0.999	MAPRE1
rs11274745	20	31,408,597	A	AGGGCAGAGGTC	0.948	0.983	MAPRE1
rs242550	20	31,408,801	C	T	0.699	0.999	MAPRE1
rs774851201	20	31,408,830	CT	C	0.884	0.782	MAPRE1
rs191130144	20	31,408,887	C	T	0.989	0.939	MAPRE1
rs3827027	20	31,409,320	C	T	0.946	1	MAPRE1
rs242551	20	31,409,792	T	C	0.424	0.999	MAPRE1
rs148959080	20	31,409,838	G	GT	0.946	1	MAPRE1
rs34128265	20	31,410,265	G	T	0.963	0.997	MAPRE1
rs187178	20	31,410,279	A	G	0.424	0.999	MAPRE1
rs6057651	20	31,410,337	G	A	0.979	0.998	MAPRE1
rs56120821	20	31,411,644	G	A	0.946	1	MAPRE1
rs242552	20	31,411,708	A	G	0.018	0.99	MAPRE1
rs242553	20	31,411,825	T	C	0.424	0.999	MAPRE1

SNP	Chromosome	Position	Effect Allele	Other Allele	Effect Allele Frequency	Imputation quality	Gene
rs544966335	20	31,412,487	G	GT	0.796	0.859	MAPRE1
rs35229267	20	31,412,546	T	G	0.982	0.994	MAPRE1
rs762087573	20	31,412,955	CA	C	0.975	0.716	MAPRE1
rs3831804	20	31,413,015	T	TA	0.784	0.998	MAPRE1
20:31414020_CT_C	20	31,414,020	CT	C	0.544	0.81	MAPRE1
rs147297128	20	31,414,185	G	A	0.965	0.916	MAPRE1
20:31414318_GC_G	20	31,414,318	GC	G	0.963	0.995	MAPRE1
rs853863	20	31,414,457	A	C	0.424	0.999	MAPRE1
rs143731582	20	31,415,546	C	T	0.985	0.951	MAPRE1
rs556859007	20	31,415,700	C	CA	0.679	0.911	MAPRE1
rs2268320	20	31,415,903	G	C	0.838	0.998	MAPRE1
rs242546	20	31,415,922	G	A	0.698	1	MAPRE1
rs2300419	20	31,416,589	T	A	0.839	0.999	MAPRE1
rs201053901	20	31,416,745	G	GT	0.956	0.989	MAPRE1
rs142101819	20	31,417,770	C	CT	0.946	0.998	MAPRE1
rs2268321	20	31,418,109	C	A	0.838	0.998	MAPRE1
rs20654	20	31,418,296	A	G	0.698	1	MAPRE1
rs374922973	20	31,418,545	C	CT	0.684	0.946	MAPRE1
rs242547	20	31,418,581	T	C	0.424	0.999	MAPRE1
rs149266207	20	31,418,598	T	TC	0.946	0.995	MAPRE1
rs242548	20	31,418,617	A	G	0.424	0.999	MAPRE1
rs242549	20	31,418,641	T	C	0.031	0.99	MAPRE1
rs661623	20	31,419,530	G	A	0.699	1	MAPRE1
rs12481627	20	31,419,605	T	C	0.957	0.978	MAPRE1
rs6057652	20	31,419,809	A	C	0.979	0.999	MAPRE1
20:31420199_GA_G	20	31,420,199	GA	G	0.425	0.998	MAPRE1
rs853854	20	31,420,757	T	A	0.478	0.999	MAPRE1
rs6057654	20	31,420,986	C	T	0.942	0.998	MAPRE1
rs73114109	20	31,421,069	A	C	0.963	0.998	MAPRE1
rs73114112	20	31,421,145	T	A	0.963	0.998	MAPRE1
rs73114114	20	31,421,291	C	T	0.963	0.998	MAPRE1
rs41289868	20	31,421,509	C	T	0.963	0.998	MAPRE1
rs73904049	20	31,421,748	T	C	0.979	0.997	MAPRE1
rs35802810	20	31,422,053	G	C	0.806	0.986	MAPRE1
rs73114118	20	31,422,185	G	A	0.946	1	MAPRE1
20:31422280_TAGA_T	20	31,422,280	TAGA	T	0.946	0.999	MAPRE1
rs6141825	20	31,422,619	A	G	0.839	0.999	MAPRE1
rs28489252	20	31,422,811	G	T	0.979	0.998	MAPRE1
rs58178200	20	31,423,292	T	C	0.979	0.999	MAPRE1
rs117137123	20	31,423,777	C	A	0.984	0.942	MAPRE1
rs183832401	20	31,423,880	T	G	0.989	0.94	MAPRE1
rs410488	20	31,425,069	A	G	0.445	0.999	MAPRE1
rs388492	20	31,425,221	G	A	0.699	1	MAPRE1
rs6141827	20	31,425,633	A	G	0.838	0.999	MAPRE1
rs113176577	20	31,426,131	A	C	0.961	0.982	MAPRE1
rs416815	20	31,426,626	C	A	0.424	1	MAPRE1
rs79396740	20	31,426,949	A	G	0.959	0.973	MAPRE1
rs59413480	20	31,427,089	A	G	0.979	0.997	MAPRE1
rs2235760	20	31,427,342	C	T	0.838	0.999	MAPRE1
20:31427484_TTTTG_T	20	31,427,484	TTTTG	T	0.839	0.995	MAPRE1
rs2070090	20	31,427,635	C	T	0.838	0.999	MAPRE1
rs55860691	20	31,427,694	C	T	0.946	1	MAPRE1
rs13045669	20	31,427,961	A	G	0.963	0.998	MAPRE1
rs62207058	20	31,429,319	C	T	0.946	1	MAPRE1
rs402951	20	31,429,489	G	T	0.699	1	MAPRE1
rs8121782	20	31,429,838	T	C	0.946	1	MAPRE1
rs6058906	20	31,430,055	C	A	0.979	0.998	MAPRE1
rs709047	20	31,430,191	A	G	0.424	1	MAPRE1
rs7271735	20	31,430,636	T	C	0.946	1	MAPRE1
rs6579038	20	31,430,770	A	G	0.946	0.999	MAPRE1
rs35993481	20	31,430,884	C	T	0.963	0.998	MAPRE1
20:31431118_GC_G	20	31,431,118	GC	G	0.979	0.996	MAPRE1
rs8117474	20	31,431,883	C	T	0.983	0.998	MAPRE1
rs140709766	20	31,432,478	A	G	0.979	0.998	MAPRE1
rs242534	20	31,432,913	G	T	0.018	0.997	MAPRE1
rs2268322	20	31,433,017	T	G	0.839	0.999	MAPRE1
rs242535	20	31,433,190	C	T	0.424	1	MAPRE1
rs55703537	20	31,433,969	A	G	0.946	0.999	MAPRE1
rs242536	20	31,435,260	T	G	0.424	0.999	MAPRE1
rs190649096	20	31,436,385	C	G	0.986	0.912	MAPRE1
rs1061992	20	31,436,681	C	T	0.963	0.997	MAPRE1
rs242538	20	31,436,747	C	T	0.424	0.999	MAPRE1
rs7270085	20	31,437,607	G	A	0.979	0.996	MAPRE1
20:33814576_GGCCGCCGCC_G	20	33,814,576	GGCCGCCGCC	G	0.25	0.923	MMP24
rs34613590	20	33,817,219	C	A	0.822	0.994	MMP24
rs2425017	20	33,817,638	T	C	0.851	0.994	MMP24
rs201399952	20	33,818,177	A	AT	0.988	0.76	MMP24
rs2425018	20	33,818,766	G	A	0.86	0.995	MMP24
rs2425019	20	33,819,415	A	G	0.57	0.994	MMP24
rs117586544	20	33,820,111	A	T	0.984	0.911	MMP24
rs6058209	20	33,821,577	G	A	0.68	0.996	MMP24
rs7267837	20	33,823,510	A	T	0.987	0.984	MMP24
rs2425020	20	33,823,832	T	C	0.257	0.95	MMP24
rs56280929	20	33,825,157	C	T	0.905	0.99	MMP24
rs12479765	20	33,825,378	G	A	0.824	0.996	MMP24
rs6120880	20	33,829,406	C	G	0.581	0.988	MMP24
rs2425021	20	33,829,778	G	C	0.848	0.997	MMP24
rs6088774	20	33,830,164	T	A	0.816	0.998	MMP24
rs370583522	20	33,830,748	A	C	0.982	0.929	MMP24
rs2425022	20	33,831,187	T	C	0.847	0.998	MMP24
rs2425023	20	33,832,494	T	C	0.665	0.992	MMP24
rs12479973	20	33,832,495	G	A	0.826	0.988	MMP24
rs6060321	20	33,833,781	C	A	0.856	0.999	MMP24
rs116934196	20	33,833,838	T	C	0.982	0.886	MMP24
20:33834093_TA_T	20	33,834,093	TA	T	0.755	0.817	MMP24
rs6088776	20	33,835,773	T	C	0.862	0.996	MMP24
rs6088778	20	33,836,110	T	C	0.889	0.718	MMP24
rs6060324	20	33,836,180	T	C	0.987	0.998	MMP24
rs671988	20	33,837,414	C	T	0.862	0.997	MMP24
20:33838919_CT_C	20	33,838,919	CT	C	0.792	0.689	MMP24
rs561724986	20	33,839,088	A	AT	0.986	0.978	MMP24
rs2247828	20	33,839,939	A	G	0.862	0.995	MMP24
rs6058210	20	33,839,973	T	C	0.987	0.999	MMP24
rs6058211	20	33,840,164	C	T	0.987	0.999	MMP24
rs13037818	20	33,840,199	C	T	0.87	0.994	MMP24
rs6060327	20	33,840,703	G	A	0.987	0.999	MMP24
rs114919877	20	33,840,930	G	A	0.987	1	MMP24
rs59270155	20	33,841,959	G	A	0.987	0.999	MMP24
rs77745605	20	33,844,024	C	T	0.987	0.999	MMP24
rs80347293	20	33,847,046	G	A	0.987	0.998	MMP24
rs2425025	20	33,847,154	G	A	0.092	0.971	MMP24
rs2425026	20	33,847,253	C	T	0.572	0.991	MMP24
rs11907928	20	33,848,456	T	G	0.841	0.991	MMP24
rs113515921	20	33,848,606	G	C	0.987	0.999	MMP24
rs2425027	20	33,849,390	C	G	0.865	0.999	MMP24
rs144888216	20	33,849,644	C	T	0.988	0.999	MMP24
rs147962064	20	33,849,897	G	A	0.975	0.908	MMP24
rs539820256	20	33,850,164	A	T	0.988	1	MMP24
rs200605260	20	33,850,211	G	C	0.987	0.999	MMP24
rs144979141	20	33,850,853	A	G	0.987	1	MMP24
rs114658163	20	33,850,948	T	G	0.988	1	MMP24
20:33851234_CCTGA_C	20	33,851,234	CCTGA	C	0.987	0.978	MMP24
rs74410031	20	33,852,011	T	C	0.987	0.999	MMP24
rs2425030	20	33,853,200	A	C	0.865	0.994	MMP24
rs11482676	20	33,853,244	CA	C	0.95	0.71	MMP24
rs6060331	20	33,853,273	C	T	0.987	0.998	MMP24
rs6060332	20	33,853,342	C	G	0.987	0.998	MMP24
rs2378346	20	33,854,104	T	G	0.864	0.995	MMP24
rs144199548	20	33,854,111	C	T	0.988	0.998	MMP24
rs2425031	20	33,855,284	T	C	0.864	0.994	MMP24
rs2425032	20	33,855,454	T	C	0.859	0.985	MMP24
rs2425033	20	33,855,626	T	C	0.852	0.995	MMP24
rs2425034	20	33,855,814	G	C	0.864	0.994	MMP24
rs2425035	20	33,855,819	T	C	0.864	0.994	MMP24
rs11906746	20	33,855,871	G	A	0.987	0.995	MMP24
rs2425036	20	33,855,981	G	C	0.864	0.994	MMP24
rs2425037	20	33,856,429	A	T	0.864	0.993	MMP24
rs2425038	20	33,856,627	A	G	0.852	0.939	MMP24
20:33856673_AT_A	20	33,856,673	AT	A	0.981	0.756	MMP24
rs2425039	20	33,856,674	T	C	0.114	0.983	MMP24
rs570591360	20	33,856,675	A	C	0.985	0.758	MMP24
20:33856828_TA_T	20	33,856,828	TA	T	0.837	0.914	MMP24
rs1205411	20	33,857,242	C	T	0.864	0.993	MMP24
rs2275274	20	33,857,544	C	T	0.917	0.983	MMP24
rs2254207	20	33,857,711	A	C	0.768	0.989	MMP24
rs116723830	20	33,858,289	T	A	0.987	0.994	MMP24
rs11907955	20	33,858,331	G	C	0.987	0.993	MMP24
rs6058218	20	33,858,772	A	G	0.768	0.988	MMP24
20:33858869_AGAG_A	20	33,858,869	AGAG	A	0.988	0.991	MMP24
rs2425040	20	33,858,878	G	A	0.865	0.991	MMP24
rs2425041	20	33,859,013	C	A	0.865	0.991	MMP24
rs6058219	20	33,859,244	G	C	0.864	0.991	MMP24
rs6058220	20	33,859,264	T	G	0.917	0.984	MMP24
rs11696548	20	33,859,675	C	T	0.848	0.981	MMP24
rs11907632	20	33,860,039	T	A	0.987	0.987	MMP24

SNP	Chromosome	Position	Effect Allele	Other Allele	Effect Allele Frequency	Imputation quality	Gene
rs11907661	20	33,860,211	T	C	0.988	0.992	MMP24
rs73903035	20	33,860,481	G	A	0.942	0.983	MMP24
rs533403393	20	33,860,547	C	T	0.988	0.719	MMP24
20:33861597_TGTGAA_T	20	33,861,597	TGTGAA	T	0.956	0.975	MMP24
rs639763	20	33,861,904	C	T	0.798	0.989	MMP24
rs73107212	20	33,863,334	C	T	0.944	0.972	MMP24
rs6060341	20	33,863,633	A	G	0.794	0.992	MMP24
rs7280	20	33,864,484	A	G	0.585	0.99	MMP24
rs75192893	21	36,161,662	G	C	0.958	0.94	RUNX1
rs201214658	21	36,162,230	G	GA	0.976	0.859	RUNX1
rs55744508	21	36,163,294	G	T	0.942	0.97	RUNX1
rs549844608	21	36,164,311	C	G	0.986	0.812	RUNX1
rs13051066	21	36,164,405	G	T	0.565	0.988	RUNX1
rs11700756	21	36,165,041	T	C	0.474	0.966	RUNX1
rs138850381	21	36,165,578	C	T	0.986	0.854	RUNX1
21:36166003_CT_C	21	36,166,003	CT	C	0.632	0.961	RUNX1
rs117101908	21	36,166,084	A	T	0.949	0.938	RUNX1
rs118036297	21	36,170,273	G	T	0.976	0.853	RUNX1
rs75708523	21	36,170,906	A	T	0.963	0.871	RUNX1
rs2073354	21	36,172,283	G	T	0.912	0.931	RUNX1
rs117700570	21	36,173,668	G	A	0.987	0.867	RUNX1
rs79656007	21	36,173,931	C	T	0.962	0.843	RUNX1
rs928742	21	36,174,790	G	A	0.014	0.933	RUNX1
rs117226154	21	36,175,351	A	C	0.984	0.967	RUNX1
rs2249650	21	36,180,986	A	G	0.473	0.976	RUNX1
rs2268276	21	36,181,014	G	A	0.55	0.978	RUNX1
rs2268277	21	36,182,049	G	C	0.646	0.999	RUNX1
rs76292615	21	36,182,136	G	A	0.979	0.928	RUNX1
rs2834642	21	36,186,319	G	A	0.579	0.995	RUNX1
rs2834643	21	36,186,396	A	G	0.578	0.995	RUNX1
rs2834644	21	36,186,747	T	C	0.576	0.992	RUNX1
rs2834645	21	36,187,686	T	C	0.766	0.959	RUNX1
rs2253319	21	36,188,046	C	T	0.677	0.955	RUNX1
rs149721392	21	36,188,409	C	T	0.984	0.94	RUNX1
rs17762984	21	36,189,083	G	A	0.986	0.857	RUNX1
rs2834646	21	36,190,040	A	C	0.098	0.987	RUNX1
rs2834647	21	36,191,308	T	C	0.049	0.991	RUNX1
rs1003314	21	36,191,646	G	C	0.09	0.998	RUNX1
rs928743	21	36,192,484	C	G	0.093	0.992	RUNX1
rs117924639	21	36,192,828	T	C	0.985	0.861	RUNX1
rs144139759	21	36,192,858	C	T	0.972	0.95	RUNX1
rs2018329	21	36,193,871	T	C	0.091	0.994	RUNX1
rs4817694	21	36,194,538	C	G	0.09	0.993	RUNX1
rs58016583	21	36,195,727	GTTTTTTTTTTTT	G	0.029	0.908	RUNX1
rs76290191	21	36,197,249	T	C	0.976	0.941	RUNX1
rs73201053	21	36,198,190	A	G	0.835	0.965	RUNX1
rs56062024	21	36,198,520	C	T	0.983	0.985	RUNX1
rs192282961	21	36,198,922	G	A	0.989	0.867	RUNX1
rs141825017	21	36,201,011	T	C	0.981	0.848	RUNX1
rs2226303	21	36,202,775	C	T	0.193	0.972	RUNX1
rs2246738	21	36,203,846	G	A	0.662	0.98	RUNX1
rs139200660	21	36,205,452	G	C	0.957	0.937	RUNX1
rs147810414	21	36,205,678	C	G	0.987	0.782	RUNX1
rs2051394	21	36,206,142	C	G	0.521	0.98	RUNX1
rs55789220	21	36,206,521	G	A	0.973	0.99	RUNX1
rs79609088	21	36,207,450	C	G	0.973	0.982	RUNX1
rs71329085	21	36,208,240	C	T	0.97	0.979	RUNX1
rs7280071	21	36,209,023	T	A	0.772	0.995	RUNX1
rs2834649	21	36,209,650	G	A	0.925	0.986	RUNX1
rs139563574	21	36,209,990	G	A	0.973	0.996	RUNX1
21:36210279_GA_G	21	36,210,279	GA	G	0.973	0.991	RUNX1
rs144776957	21	36,210,307	C	A	0.974	0.996	RUNX1
21:36210511_ATCT_A	21	36,210,511	ATCT	A	0.973	0.993	RUNX1
21:36210515_GTGGC_G	21	36,210,515	GTGGC	G	0.973	0.993	RUNX1
rs34835411	21	36,211,188	C	G	0.396	0.989	RUNX1
rs55756507	21	36,211,593	C	T	0.974	0.998	RUNX1
rs74670244	21	36,212,155	G	C	0.974	1	RUNX1
rs76469164	21	36,212,351	C	T	0.973	0.999	RUNX1
rs116189288	21	36,212,396	T	C	0.973	0.999	RUNX1
rs116683479	21	36,212,502	G	A	0.974	0.999	RUNX1
rs114382302	21	36,212,513	C	A	0.974	0.999	RUNX1
rs114694996	21	36,212,523	G	A	0.973	0.998	RUNX1
rs117273686	21	36,212,815	C	G	0.974	0.998	RUNX1
rs201195657	21	36,212,923	G	C	0.973	0.998	RUNX1
rs114210939	21	36,212,940	G	A	0.974	0.998	RUNX1
rs116877183	21	36,213,160	C	T	0.974	0.998	RUNX1
rs149097953	21	36,213,330	G	C	0.974	0.998	RUNX1
rs116109464	21	36,213,405	G	T	0.974	0.998	RUNX1
rs116273341	21	36,213,484	C	T	0.973	0.994	RUNX1
rs61209186	21	36,213,523	A	G	0.973	0.995	RUNX1
rs59524736	21	36,213,610	C	G	0.973	0.994	RUNX1
rs114848905	21	36,213,769	C	T	0.974	0.997	RUNX1
rs76692587	21	36,213,832	A	G	0.973	0.997	RUNX1
rs2284612	21	36,213,879	T	C	0.394	0.994	RUNX1
rs9978897	21	36,213,897	A	G	0.221	0.992	RUNX1
rs114736217	21	36,214,127	G	C	0.974	0.997	RUNX1
21:36214317_GAGTACTC_G	21	36,214,317	GAGTACTC	G	0.973	0.994	RUNX1
rs139353312	21	36,214,528	A	G	0.973	0.997	RUNX1
rs181586420	21	36,214,660	C	T	0.989	0.836	RUNX1
rs56335473	21	36,215,094	G	A	0.974	0.994	RUNX1
rs56161069	21	36,215,102	A	C	0.974	0.995	RUNX1
rs56381260	21	36,215,117	G	C	0.973	0.995	RUNX1
rs143062622	21	36,215,236	G	A	0.976	0.842	RUNX1
rs114362780	21	36,215,280	T	C	0.973	0.996	RUNX1
rs117419053	21	36,215,323	T	C	0.973	0.996	RUNX1
rs2268279	21	36,215,354	G	A	0.554	0.992	RUNX1
rs113996067	21	36,215,490	T	C	0.973	0.996	RUNX1
rs114758675	21	36,215,657	G	A	0.973	0.996	RUNX1
rs8129714	21	36,215,909	C	T	0.973	0.996	RUNX1
rs2268280	21	36,215,976	G	C	0.831	0.994	RUNX1
rs77340933	21	36,216,214	A	G	0.973	0.996	RUNX1
rs147016829	21	36,216,360	G	A	0.988	0.802	RUNX1
rs79906723	21	36,216,363	A	G	0.973	0.996	RUNX1
rs79008866	21	36,216,488	A	T	0.973	0.995	RUNX1
rs114302972	21	36,216,565	G	A	0.973	0.995	RUNX1
rs79370398	21	36,216,662	C	T	0.973	0.995	RUNX1
rs74328086	21	36,216,666	A	T	0.973	0.995	RUNX1
21:36216883_ATGAG_A	21	36,216,883	ATGAG	A	0.875	0.977	RUNX1
rs2268282	21	36,217,802	C	T	0.85	0.988	RUNX1
rs2284613	21	36,218,264	A	G	0.675	0.995	RUNX1
rs374933824	21	36,218,491	CT	C	0.383	0.861	RUNX1
rs762164	21	36,218,774	C	A	0.676	0.996	RUNX1
rs56399902	21	36,218,927	G	C	0.974	0.992	RUNX1
rs4817695	21	36,219,317	A	G	0.676	0.996	RUNX1
rs2154450	21	36,219,566	A	G	0.677	0.996	RUNX1
rs2186290	21	36,219,716	T	G	0.674	0.996	RUNX1
21:36219771_CAAT_C	21	36,219,771	CAAT	C	0.676	0.989	RUNX1
rs62211770	21	36,220,206	C	T	0.677	0.996	RUNX1
rs2268283	21	36,220,493	G	A	0.677	0.997	RUNX1
rs2268284	21	36,220,679	T	C	0.864	0.976	RUNX1
rs2268285	21	36,220,720	A	C	0.677	0.997	RUNX1
rs62211771	21	36,220,734	G	A	0.968	0.903	RUNX1
rs2268286	21	36,220,771	A	G	0.677	0.997	RUNX1
rs8128445	21	36,221,088	C	T	0.677	0.997	RUNX1
rs571296550	21	36,221,681	G	GTA	0.987	0.76	RUNX1
rs188695559	21	36,221,730	T	G	0.709	0.971	RUNX1
21:36221760_GTA_G	21	36,221,760	GTA	G	0.68	0.977	RUNX1
rs8134380	21	36,222,139	T	A	0.447	0.996	RUNX1
rs11088295	21	36,222,258	G	A	0.673	0.996	RUNX1
21:36222324_AAAC_A	21	36,222,324	AAAC	A	0.871	0.909	RUNX1
rs2409534	21	36,222,447	T	C	0.664	0.994	RUNX1
rs2409535	21	36,222,560	T	C	0.672	0.996	RUNX1
rs2409536	21	36,222,713	G	C	0.667	0.998	RUNX1
rs56963816	21	36,222,862	T	C	0.663	0.984	RUNX1
rs60855390	21	36,223,234	T	C	0.662	0.949	RUNX1
rs968625	21	36,223,627	G	A	0.785	0.911	RUNX1
rs2284614	21	36,223,744	C	T	0.701	0.91	RUNX1
rs2284615	21	36,223,886	G	A	0.672	0.904	RUNX1
rs2284616	21	36,223,906	T	C	0.663	0.902	RUNX1
rs12483501	21	36,224,276	T	C	0.63	0.876	RUNX1
rs111449846	21	36,224,315	A	G	0.8	0.862	RUNX1
rs7279383	21	36,224,963	C	G	0.822	0.742	RUNX1
rs2032097	21	36,224,983	T	C	0.724	0.729	RUNX1
rs137941718	21	36,225,292	C	T	0.978	0.831	RUNX1
rs140639726	21	36,225,387	T	C	0.978	0.726	RUNX1
rs138310248	21	36,225,893	T	C	0.989	0.854	RUNX1
rs34643463	21	36,226,112	A	AT	0.442	0.734	RUNX1
rs77439206	21	36,226,645	A	T	0.977	0.962	RUNX1
rs117528518	21	36,226,796	C	T	0.978	0.971	RUNX1
21:36227051_ACGGGCTG_A	21	36,227,051	ACGGGCTG	A	0.978	0.967	RUNX1
rs77700124	21	36,227,414	C	T	0.978	0.976	RUNX1
rs77367874	21	36,227,547	T	A	0.978	0.976	RUNX1
rs8127581	21	36,227,745	C	T	0.299	0.988	RUNX1
rs115631819	21	36,227,924	C	T	0.978	0.981	RUNX1
rs2248383	21	36,229,941	C	T	0.195	0.993	RUNX1

SNP	Chromosome	Position	Effect Allele	Other Allele	Effect Allele Frequency	Imputation quality	Gene
rs2409560	21	36,229,995	C	A	0.192	0.997	RUNX1
rs7282751	21	36,230,819	C	T	0.99	0.963	RUNX1
rs2834653	21	36,231,159	A	C	0.467	0.988	RUNX1
rs7283251	21	36,231,175	C	T	0.778	0.996	RUNX1
rs56138997	21	36,231,613	G	A	0.978	0.986	RUNX1
rs2248734	21	36,232,482	T	C	0.798	0.995	RUNX1
rs2248735	21	36,232,535	T	C	0.798	0.995	RUNX1
rs2268288	21	36,232,671	T	C	0.797	0.995	RUNX1
rs11702579	21	36,232,856	T	C	0.697	0.981	RUNX1
rs79760373	21	36,233,756	T	G	0.978	0.992	RUNX1
rs7280216	21	36,233,905	C	G	0.697	0.976	RUNX1
rs13047845	21	36,234,200	C	A	0.816	0.987	RUNX1
rs112283955	21	36,234,325	T	TAA	0.978	0.988	RUNX1
rs76826924	21	36,234,423	G	C	0.978	0.993	RUNX1
rs7275871	21	36,234,763	A	G	0.989	0.979	RUNX1
rs1003574	21	36,235,047	G	T	0.529	0.969	RUNX1
rs2834654	21	36,235,311	T	C	0.221	0.981	RUNX1
rs138807819	21	36,235,341	A	G	0.978	0.986	RUNX1
rs9979015	21	36,235,568	C	A	0.797	0.965	RUNX1
rs2251826	21	36,235,739	A	G	0.456	0.939	RUNX1
rs118125122	21	36,236,084	A	G	0.977	0.797	RUNX1
rs7282616	21	36,236,101	T	C	0.824	0.877	RUNX1
rs2268289	21	36,236,443	T	C	0.751	0.95	RUNX1
rs2268291	21	36,236,819	G	A	0.977	0.722	RUNX1
rs11700925	21	36,237,656	C	T	0.736	0.999	RUNX1
21:36237805_GTCCATCCAAATGCC_G	21	36,237,805	GTCCATCCAAATGCC	G	0.735	0.992	RUNX1
rs35068491	21	36,238,307	T	C	0.788	0.994	RUNX1
rs11701104	21	36,238,517	C	T	0.774	0.951	RUNX1
rs138024348	21	36,239,997	T	C	0.977	0.819	RUNX1
rs148409889	21	36,241,624	C	T	0.985	0.679	RUNX1
rs2252585	21	36,241,929	T	C	0.731	0.796	RUNX1
rs2268292	21	36,243,108	A	G	0.935	0.847	RUNX1
rs2268293	21	36,243,213	A	C	0.727	0.786	RUNX1
rs2268294	21	36,243,258	A	G	0.74	0.784	RUNX1
rs117657409	21	36,243,755	C	T	0.98	0.744	RUNX1
21:36244143_CTT_C	21	36,244,143	CTT	C	0.783	0.65	RUNX1
rs143138559	21	36,244,193	C	T	0.982	0.883	RUNX1
rs2015876	21	36,244,416	C	T	0.858	0.844	RUNX1
rs8126925	21	36,245,236	C	G	0.762	0.987	RUNX1
rs11910742	21	36,245,671	G	C	0.874	0.964	RUNX1
rs2284617	21	36,245,902	A	C	0.761	0.99	RUNX1
rs2284618	21	36,246,078	T	C	0.761	0.99	RUNX1
rs2284619	21	36,246,092	A	G	0.762	0.991	RUNX1
rs9981173	21	36,246,157	C	T	0.762	0.992	RUNX1
rs9981325	21	36,246,158	G	A	0.776	0.991	RUNX1
rs2834656	21	36,246,252	G	A	0.766	0.993	RUNX1
rs2834657	21	36,246,352	A	C	0.765	0.993	RUNX1
rs28623037	21	36,246,610	T	A	0.765	0.995	RUNX1
rs777235941	21	36,246,696	CAGG	C	0.765	0.992	RUNX1
21:36246974_TA_T	21	36,246,974	TA	T	0.643	0.98	RUNX1
rs113529220	21	36,247,203	G	A	0.988	0.663	RUNX1
rs8128348	21	36,247,444	C	T	0.76	0.996	RUNX1
rs2300396	21	36,248,121	G	C	0.741	0.994	RUNX1
rs9976377	21	36,248,159	G	C	0.905	0.979	RUNX1
21:36249151_CTTTTTCT_C	21	36,249,151	CTTTTTCT	C	0.692	0.883	RUNX1
rs2300397	21	36,249,389	C	T	0.79	0.998	RUNX1
rs2300398	21	36,249,431	C	T	0.79	0.998	RUNX1
21:36250466_CA_C	21	36,250,466	CA	C	0.864	0.776	RUNX1
rs2834659	21	36,250,764	G	A	0.905	0.986	RUNX1
rs2298351	21	36,252,595	G	A	0.755	0.994	RUNX1
21:36253146_ATAACT_A	21	36,253,146	ATAACT	A	0.901	0.976	RUNX1
rs2298354	21	36,253,194	T	A	0.74	0.994	RUNX1
rs12482508	21	36,254,043	C	T	0.759	0.993	RUNX1
rs2300399	21	36,254,732	G	A	0.774	0.992	RUNX1
rs2300400	21	36,254,807	T	C	0.789	0.994	RUNX1
21:36254987_GTT_G	21	36,254,987	GTT	G	0.773	0.985	RUNX1
rs2300401	21	36,258,025	T	C	0.642	0.987	RUNX1
rs8126699	21	36,258,839	T	C	0.882	0.988	RUNX1
rs192533631	21	36,260,953	G	A	0.99	0.892	RUNX1
21:36261695_CT_C	21	36,261,695	CT	C	0.977	0.777	RUNX1
rs113111621	21	36,261,705	G	A	0.969	0.747	RUNX1
rs529894328	21	36,261,949	G	C	0.99	0.907	RUNX1
rs544550393	21	36,262,014	T	A	0.981	0.746	RUNX1
rs13048584	21	36,262,291	G	A	0.899	0.987	RUNX1
rs7281361	21	36,262,427	A	G	0.646	0.982	RUNX1
rs75009210	21	36,262,764	C	G	0.975	0.896	RUNX1
rs76056678	21	36,262,898	C	T	0.974	0.895	RUNX1
rs761249134	21	36,264,004	TA	T	0.849	0.9	RUNX1
rs2298322	21	36,264,005	A	T	0.857	0.668	RUNX1
rs560233151	21	36,266,078	T	A	0.963	0.838	RUNX1
rs371244047	21	36,266,079	T	A	0.775	0.831	RUNX1
rs2018330	21	36,266,080	T	A	0.652	0.917	RUNX1
rs2186289	21	36,266,081	A	T	0.948	0.653	RUNX1
rs78861611	21	36,266,090	C	A	0.923	0.626	RUNX1
rs62211817	21	36,266,986	T	C	0.772	0.983	RUNX1
rs8130963	21	36,267,418	A	G	0.645	0.985	RUNX1
21:36267720_CA_C	21	36,267,720	CA	C	0.989	0.665	RUNX1
rs62211818	21	36,268,139	G	T	0.743	0.983	RUNX1
rs113026296	21	36,268,269	T	TAAGGTAG	0.746	0.964	RUNX1
rs6517260	21	36,268,873	A	G	0.736	0.983	RUNX1
rs6517261	21	36,268,936	T	C	0.736	0.983	RUNX1
rs10639291	21	36,268,982	A	AAAAAG	0.735	0.974	RUNX1
rs28690822	21	36,269,249	G	C	0.746	0.982	RUNX1
rs9976623	21	36,269,508	T	C	0.746	0.983	RUNX1
rs2409561	21	36,270,411	C	T	0.66	0.985	RUNX1
rs2834660	21	36,271,158	C	T	0.772	0.982	RUNX1
rs7283301	21	36,271,348	T	C	0.636	0.988	RUNX1
rs73201080	21	36,272,062	C	G	0.918	0.774	RUNX1
rs76621869	21	36,272,365	T	C	0.943	0.915	RUNX1
rs118143904	21	36,272,562	G	A	0.983	0.631	RUNX1
rs10854374	21	36,273,518	A	C	0.759	0.984	RUNX1
rs2834662	21	36,274,176	T	C	0.74	0.987	RUNX1
rs35791004	21	36,274,187	T	C	0.735	0.988	RUNX1
rs7279817	21	36,274,373	G	A	0.759	0.986	RUNX1
rs2834663	21	36,274,440	A	G	0.874	0.98	RUNX1
rs7279557	21	36,274,443	A	G	0.739	0.988	RUNX1
rs7283379	21	36,274,648	A	G	0.74	0.988	RUNX1
rs6417685	21	36,274,936	T	C	0.085	0.705	RUNX1
rs62211821	21	36,275,298	T	G	0.754	0.986	RUNX1
rs2834664	21	36,275,439	T	C	0.873	0.977	RUNX1
rs2834665	21	36,275,646	T	G	0.74	0.986	RUNX1
rs16992490	21	36,276,512	C	T	0.745	0.982	RUNX1
rs2834666	21	36,276,675	G	A	0.913	0.984	RUNX1
rs2834667	21	36,276,686	T	G	0.735	0.983	RUNX1
rs7364037	21	36,276,892	A	G	0.739	0.979	RUNX1
21:36277275_AAAG_A	21	36,277,275	AAAG	A	0.913	0.964	RUNX1
rs146286453	21	36,279,593	G	T	0.989	0.809	RUNX1
rs112637192	21	36,279,918	T	TCCTGA	0.909	0.586	RUNX1
rs9976688	21	36,279,933	C	T	0.833	0.679	RUNX1
rs2834670	21	36,280,376	A	G	0.833	0.737	RUNX1
rs201708857	21	36,280,873	A	AG	0.946	0.845	RUNX1
rs2834671	21	36,281,630	T	C	0.935	0.847	RUNX1
rs557185015	21	36,281,885	C	CAAA	0.881	0.844	RUNX1
rs34902592	21	36,282,225	C	T	0.577	0.92	RUNX1
rs73373765	21	36,282,500	A	G	0.971	0.816	RUNX1
rs73373771	21	36,282,821	T	C	0.969	0.82	RUNX1
rs28567906	21	36,283,811	A	G	0.912	0.959	RUNX1
rs150601921	21	36,284,795	C	T	0.983	0.765	RUNX1
rs73373773	21	36,284,849	T	C	0.964	0.863	RUNX1
rs71329087	21	36,285,832	C	T	0.975	0.769	RUNX1
rs28421324	21	36,286,938	A	T	0.9	0.995	RUNX1
rs77884453	21	36,287,785	C	A	0.951	0.99	RUNX1
rs2834672	21	36,289,022	G	A	0.525	0.983	RUNX1
rs2834673	21	36,289,048	C	T	0.574	0.983	RUNX1
rs35202532	21	36,290,299	G	A	0.596	0.971	RUNX1
rs143421769	21	36,290,969	G	T	0.985	0.696	RUNX1
rs13051176	21	36,291,133	A	G	0.1	0.997	RUNX1
rs13051383	21	36,291,216	C	A	0.101	0.998	RUNX1
rs11702617	21	36,291,237	G	A	0.098	0.996	RUNX1
rs140146597	21	36,291,646	C	A	0.981	0.985	RUNX1
rs2834674	21	36,292,082	C	T	0.1	0.999	RUNX1
rs2834675	21	36,292,500	G	A	0.942	0.864	RUNX1
rs12482105	21	36,292,545	C	T	0.101	0.999	RUNX1
rs11384665	21	36,293,085	G	GT	0.1	0.998	RUNX1
rs35655125	21	36,293,261	T	C	0.569	0.994	RUNX1
rs34372043	21	36,293,319	C	T	0.569	0.995	RUNX1
rs4816499	21	36,293,381	A	C	0.099	0.997	RUNX1
rs4816500	21	36,293,565	A	G	0.011	0.974	RUNX1
rs35497104	21	36,293,609	C	T	0.565	0.997	RUNX1
21:36293932_AT_A	21	36,293,932	AT	A	0.097	0.983	RUNX1
rs34872427	21	36,294,044	T	C	0.583	0.998	RUNX1
rs9982324	21	36,294,385	C	T	0.92	0.995	RUNX1
rs4816501	21	36,294,539	T	C	0.099	0.996	RUNX1
rs56185286	21	36,294,987	G	A	0.568	0.997	RUNX1
rs558467033	21	36,296,195	A	T	0.913	0.991	RUNX1

SNP	Chromosome	Position	Effect Allele	Other Allele	Effect Allele Frequency	Imputation quality	Gene
21:36296387_AC_A	21	36,296,387	AC	A	0.117	0.944	RUNX1
rs116213488	21	36,296,389	C	G	0.117	0.945	RUNX1
rs1006639	21	36,296,864	G	T	0.118	0.958	RUNX1
rs4817698	21	36,297,092	T	C	0.097	0.993	RUNX1
rs9305558	21	36,297,132	C	T	0.913	0.992	RUNX1
rs4817699	21	36,297,178	T	C	0.099	0.993	RUNX1
rs2017589	21	36,298,493	T	A	0.099	0.987	RUNX1
rs62216422	21	36,298,843	T	C	0.603	0.963	RUNX1
rs7280125	21	36,298,856	C	A	0.927	0.525	RUNX1
rs2051392	21	36,298,914	T	C	0.603	0.974	RUNX1
rs773376152	21	36,299,142	ACAT	A	0.603	0.959	RUNX1
rs764967	21	36,299,665	G	T	0.137	0.954	RUNX1
rs17812779	21	36,300,619	T	C	0.981	0.973	RUNX1
rs7282509	21	36,300,963	T	C	0.958	0.989	RUNX1
rs111275792	21	36,300,966	G	A	0.96	0.99	RUNX1
21:36300976_TA_T	21	36,300,976	TA	T	0.907	0.73	RUNX1
rs10483023	21	36,301,020	G	T	0.958	0.989	RUNX1
rs4502515	21	36,301,095	A	G	0.958	0.99	RUNX1
rs565653105	21	36,301,250	A	AT	0.221	0.505	RUNX1
rs142522858	21	36,301,407	A	G	0.987	0.871	RUNX1
rs9979181	21	36,301,712	G	A	0.959	0.994	RUNX1
rs9978940	21	36,301,737	A	T	0.96	0.994	RUNX1
rs9979335	21	36,302,066	C	T	0.96	0.994	RUNX1
21:36302534_TA_T	21	36,302,534	TA	T	0.957	0.973	RUNX1
rs531974953	21	36,302,536	T	G	0.957	0.973	RUNX1
rs141115974	21	36,302,565	A	ATGTG	0.545	0.982	RUNX1
rs34843396	21	36,302,590	A	T	0.949	0.959	RUNX1
rs117091111	21	36,302,594	A	T	0.958	0.986	RUNX1
rs9982365	21	36,302,857	C	T	0.958	0.992	RUNX1
rs9982454	21	36,302,917	C	T	0.958	0.992	RUNX1
rs112793728	21	36,303,012	T	A	0.806	0.697	RUNX1
rs532845689	21	36,303,020	A	T	0.971	0.914	RUNX1
rs9974398	21	36,303,998	C	T	0.958	0.993	RUNX1
rs9974558	21	36,304,178	C	T	0.958	0.993	RUNX1
rs28610975	21	36,305,028	G	A	0.958	0.994	RUNX1
rs2834677	21	36,305,109	C	T	0.958	0.994	RUNX1
rs28625365	21	36,305,134	C	T	0.958	0.993	RUNX1
rs2032098	21	36,305,766	T	C	0.494	0.997	RUNX1
rs915573	21	36,306,410	T	C	0.01	0.959	RUNX1
rs11701937	21	36,306,566	A	T	0.957	0.994	RUNX1
rs2834678	21	36,306,719	T	G	0.957	0.993	RUNX1
rs28520273	21	36,307,252	T	C	0.959	0.994	RUNX1
rs9976231	21	36,307,668	T	C	0.959	0.994	RUNX1
rs7282762	21	36,308,348	T	G	0.494	0.995	RUNX1
rs755645167	21	36,308,455	AGTGCTGTTT	A	0.96	0.991	RUNX1
rs777304638	21	36,308,567	CT	C	0.958	0.988	RUNX1
rs7283100	21	36,308,575	T	A	0.957	0.991	RUNX1
rs928774	21	36,308,576	A	T	0.557	0.995	RUNX1
rs559369519	21	36,308,577	A	T	0.944	0.9	RUNX1
rs35504628	21	36,309,617	C	CA	0.689	0.725	RUNX1
rs2834681	21	36,310,410	C	T	0.957	0.996	RUNX1
rs17812791	21	36,310,516	T	C	0.981	0.976	RUNX1
rs2834683	21	36,311,296	T	C	0.558	0.989	RUNX1
rs11702474	21	36,311,716	T	C	0.988	0.99	RUNX1
rs1892687	21	36,312,022	A	G	0.494	0.994	RUNX1
rs1892688	21	36,312,115	T	G	0.958	0.993	RUNX1
rs2834684	21	36,312,347	C	T	0.65	0.946	RUNX1
rs16992540	21	36,312,884	C	G	0.958	0.993	RUNX1
rs8126567	21	36,313,618	C	T	0.559	0.996	RUNX1
21:36314127_CT_C	21	36,314,127	CT	C	0.944	0.682	RUNX1
rs144753134	21	36,314,441	C	G	0.989	0.915	RUNX1
21:36315606_ACTTCTTT_A	21	36,315,606	ACTTCTTT	A	0.989	0.661	RUNX1
rs60928534	21	36,315,615	C	A	0.961	0.97	RUNX1
rs58549964	21	36,315,616	T	A	0.961	0.97	RUNX1
21:36315617_GTCT_G	21	36,315,617	GTCT	G	0.962	0.965	RUNX1
21:36315623_ACC_A	21	36,315,623	ACC	A	0.961	0.97	RUNX1
rs59214225	21	36,315,629	T	A	0.961	0.971	RUNX1
rs6517265	21	36,316,585	A	G	0.957	0.996	RUNX1
rs3804014	21	36,316,985	T	G	0.957	0.997	RUNX1
rs9980275	21	36,317,936	T	A	0.964	0.963	RUNX1
rs75296070	21	36,318,128	G	A	0.887	0.841	RUNX1
rs2834687	21	36,318,738	T	C	0.957	0.998	RUNX1
rs112710603	21	36,318,773	T	C	0.988	0.771	RUNX1
rs531166468	21	36,320,398	G	A	0.981	0.622	RUNX1
rs9981562	21	36,320,468	T	A	0.959	0.996	RUNX1
rs113112312	21	36,321,167	T	C	0.981	0.968	RUNX1
rs8131102	21	36,321,657	C	T	0.957	0.997	RUNX1
rs7281340	21	36,321,807	T	C	0.957	0.996	RUNX1
rs117846350	21	36,322,900	G	A	0.987	0.996	RUNX1
rs150355206	21	36,322,926	C	T	0.989	0.917	RUNX1
rs9979153	21	36,323,507	C	T	0.959	0.994	RUNX1
rs6517266	21	36,324,506	T	C	0.957	0.996	RUNX1
21:36325424_CT_C	21	36,325,424	CT	C	0.861	0.742	RUNX1
rs8133478	21	36,325,863	A	G	0.957	0.996	RUNX1
rs8133613	21	36,325,913	A	C	0.957	0.997	RUNX1
rs2051179	21	36,326,553	A	T	0.497	0.995	RUNX1
rs762246	21	36,327,365	A	G	0.958	0.998	RUNX1
rs117264958	21	36,327,507	C	T	0.982	0.985	RUNX1
rs8127214	21	36,328,403	G	T	0.958	0.999	RUNX1
rs17227139	21	36,330,465	T	C	0.981	0.977	RUNX1
rs149834074	21	36,332,016	G	GA	0.944	0.893	RUNX1
rs2006089	21	36,333,368	C	T	0.557	0.995	RUNX1
rs76793647	21	36,334,356	A	C	0.981	0.987	RUNX1
rs9975428	21	36,334,456	T	C	0.512	0.997	RUNX1
rs35002125	21	36,334,878	G	GT	0.512	0.996	RUNX1
rs9978027	21	36,335,405	G	A	0.959	0.989	RUNX1
rs2834693	21	36,335,579	G	A	0.494	0.998	RUNX1
rs145098259	21	36,335,659	C	T	0.977	0.835	RUNX1
rs2834694	21	36,335,981	C	A	0.499	0.993	RUNX1
rs2834695	21	36,336,023	C	A	0.494	0.998	RUNX1
rs76354179	21	36,336,063	C	T	0.981	0.984	RUNX1
rs2256731	21	36,336,246	T	C	0.493	0.999	RUNX1
rs9981278	21	36,336,551	C	T	0.959	0.988	RUNX1
rs4239795	21	36,336,751	C	A	0.494	0.999	RUNX1
rs2834696	21	36,336,853	A	T	0.493	1	RUNX1
rs78062086	21	36,337,036	C	T	0.981	0.984	RUNX1
rs2834698	21	36,337,085	C	T	0.494	0.999	RUNX1
rs2834699	21	36,337,097	A	G	0.493	0.999	RUNX1
rs780423715	21	36,337,294	CACAT	C	0.958	0.978	RUNX1
rs558972647	21	36,337,300	C	T	0.965	0.726	RUNX1
rs8127732	21	36,337,320	C	T	0.503	0.993	RUNX1
rs7276908	21	36,337,493	G	A	0.451	0.996	RUNX1
rs11270485	21	36,337,499	G	GCACACACACAGCCT	0.494	0.995	RUNX1
rs2834700	21	36,337,591	T	C	0.494	0.997	RUNX1
rs2834701	21	36,337,654	A	C	0.494	0.997	RUNX1
21:36337807_GA_G	21	36,337,807	GA	G	0.497	0.979	RUNX1
rs781605294	21	36,337,986	AT	A	0.958	0.957	RUNX1
rs11700785	21	36,338,087	C	T	0.941	0.96	RUNX1
rs9976513	21	36,338,091	T	A	0.958	0.963	RUNX1
rs9976122	21	36,338,306	C	T	0.958	0.959	RUNX1
21:36338345_TG_T	21	36,338,345	TG	T	0.958	0.939	RUNX1
rs9976241	21	36,338,475	C	T	0.958	0.953	RUNX1
rs11701453	21	36,338,916	C	G	0.203	0.946	RUNX1
rs11701555	21	36,339,264	G	A	0.826	0.766	RUNX1
rs58144960	21	36,339,522	G	C	0.75	0.83	RUNX1
rs7278199	21	36,339,532	G	A	0.752	0.829	RUNX1
rs2834702	21	36,340,396	G	A	0.342	0.948	RUNX1
rs78578760	21	36,340,733	G	A	0.987	0.921	RUNX1
rs9974986	21	36,340,816	T	C	0.339	0.971	RUNX1
rs2834703	21	36,340,856	C	T	0.345	0.974	RUNX1
rs9975183	21	36,341,040	T	C	0.655	0.975	RUNX1
rs9975188	21	36,341,045	T	C	0.655	0.975	RUNX1
rs2834705	21	36,341,685	G	C	0.663	0.975	RUNX1
rs62216442	21	36,341,719	G	A	0.658	0.979	RUNX1
rs66816131	21	36,342,073	C	A	0.671	0.941	RUNX1
rs2834706	21	36,342,691	T	C	0.653	0.98	RUNX1
rs2834707	21	36,343,552	C	T	0.635	0.997	RUNX1
rs78265346	21	36,345,622	G	A	0.97	0.808	RUNX1
rs139843464	21	36,346,261	T	C	0.989	0.938	RUNX1
rs55857134	21	36,347,627	T	C	0.669	0.979	RUNX1
21:36348425_CA_C	21	36,348,425	CA	C	0.676	0.933	RUNX1
rs112651059	21	36,348,711	G	A	0.973	0.868	RUNX1
21:36349158_TA_T	21	36,349,158	TA	T	0.936	0.594	RUNX1
rs2834709	21	36,349,625	C	T	0.634	0.994	RUNX1
rs2834710	21	36,350,221	C	T	0.634	0.994	RUNX1
rs2834711	21	36,350,830	A	G	0.631	0.992	RUNX1
rs144353115	21	36,351,498	G	A	0.965	0.832	RUNX1
rs2834712	21	36,351,891	T	C	0.627	0.991	RUNX1
rs56068023	21	36,352,251	A	G	0.64	0.985	RUNX1
rs73203018	21	36,352,272	C	T	0.971	0.875	RUNX1
rs62216445	21	36,352,353	G	A	0.641	0.984	RUNX1
rs140568284	21	36,354,694	G	GGTGTGTGT	0.43	0.832	RUNX1
21:36356733_TAC_T	21	36,356,733	TAC	T	0.478	0.983	RUNX1
rs2834714	21	36,357,352	T	C	0.653	0.998	RUNX1
rs990116	21	36,357,612	G	A	0.654	0.998	RUNX1

SNP	Chromosome	Position	Effect Allele	Other Allele	Effect Allele Frequency	Imputation quality	Gene
rs1008882	21	36,358,077	A	G	0.655	0.997	RUNX1
rs1008881	21	36,358,229	G	A	0.655	0.997	RUNX1
rs28391463	21	36,358,763	T	C	0.653	0.996	RUNX1
rs2834716	21	36,359,392	G	A	0.662	0.993	RUNX1
rs933131	21	36,359,688	G	A	0.477	0.992	RUNX1
rs762248	21	36,360,139	C	T	0.123	0.989	RUNX1
rs73203029	21	36,360,149	C	T	0.975	0.955	RUNX1
rs762249	21	36,360,202	A	G	0.123	0.989	RUNX1
rs743342	21	36,360,433	A	G	0.653	0.995	RUNX1
rs118131631	21	36,360,816	C	T	0.954	0.957	RUNX1
rs2834718	21	36,360,884	A	T	0.876	0.988	RUNX1
rs144294671	21	36,361,297	C	CTCTA	0.876	0.965	RUNX1
rs71329090	21	36,361,313	T	C	0.669	0.981	RUNX1
rs8129389	21	36,362,141	C	G	0.876	0.987	RUNX1
rs2834719	21	36,362,205	C	T	0.876	0.987	RUNX1
rs8129743	21	36,362,404	C	T	0.876	0.987	RUNX1
rs8130071	21	36,362,414	G	A	0.876	0.987	RUNX1
rs8129846	21	36,362,433	C	T	0.876	0.987	RUNX1
rs2242877	21	36,362,568	A	C	0.876	0.987	RUNX1
rs2242879	21	36,362,664	G	A	0.876	0.987	RUNX1
rs2242880	21	36,362,733	A	T	0.876	0.987	RUNX1
rs2242881	21	36,362,786	A	T	0.876	0.988	RUNX1
rs572191266	21	36,362,944	A	AT	0.877	0.937	RUNX1
rs2409563	21	36,362,961	C	T	0.876	0.987	RUNX1
rs2409564	21	36,363,054	A	G	0.876	0.987	RUNX1
rs737234	21	36,363,322	A	T	0.876	0.987	RUNX1
rs4816502	21	36,363,528	C	A	0.699	0.986	RUNX1
rs9977678	21	36,363,560	G	A	0.877	0.988	RUNX1
21:36363606_AAG_A	21	36,363,606	AAG	A	0.876	0.977	RUNX1
rs11088301	21	36,364,083	A	C	0.876	0.986	RUNX1
rs9636887	21	36,364,102	C	T	0.2	0.996	RUNX1
rs11088302	21	36,364,162	A	G	0.532	0.989	RUNX1
rs61006454	21	36,364,303	G	A	0.877	0.986	RUNX1
rs8131269	21	36,364,528	G	A	0.877	0.986	RUNX1
rs8130985	21	36,364,598	A	G	0.877	0.985	RUNX1
rs8127202	21	36,364,720	T	C	0.877	0.985	RUNX1
rs8134978	21	36,364,751	G	A	0.877	0.984	RUNX1
rs8127225	21	36,364,765	T	C	0.876	0.982	RUNX1
rs8134862	21	36,364,816	C	G	0.877	0.982	RUNX1
rs8127333	21	36,364,825	T	C	0.877	0.982	RUNX1
rs59680634	21	36,364,836	T	C	0.635	0.984	RUNX1
rs34125228	21	36,364,904	G	A	0.877	0.982	RUNX1
rs767333755	21	36,364,971	TA	T	0.618	0.945	RUNX1
rs13051092	21	36,365,228	C	T	0.689	0.988	RUNX1
rs73203034	21	36,365,246	G	A	0.986	0.893	RUNX1
rs148086448	21	36,365,843	G	GAC	0.877	0.964	RUNX1
21:36365888_ACACACACAGG_A	21	36,365,888	ACACACACAGG	A	0.967	0.782	RUNX1
rs551849935	21	36,365,932	A	G	0.944	0.712	RUNX1
rs11459165	21	36,366,493	A	AT	0.875	0.955	RUNX1
rs59390681	21	36,367,089	T	C	0.653	0.999	RUNX1
rs9636888	21	36,367,434	C	T	0.37	0.945	RUNX1
rs61207288	21	36,368,017	C	T	0.896	0.991	RUNX1
rs55773542	21	36,368,272	C	T	0.896	0.993	RUNX1
rs34841406	21	36,368,365	T	A	0.889	0.998	RUNX1
rs753301685	21	36,368,574	CTCTGCCT	C	0.891	0.986	RUNX1
rs60369602	21	36,368,583	A	G	0.889	1	RUNX1
rs13052096	21	36,368,626	A	C	0.889	0.999	RUNX1
rs73900763	21	36,368,709	C	T	0.889	0.999	RUNX1
rs148399889	21	36,368,930	A	ACTCTTC	0.641	0.997	RUNX1
rs117361029	21	36,369,081	A	C	0.962	0.992	RUNX1
rs73900764	21	36,369,265	A	T	0.889	0.998	RUNX1
rs147275608	21	36,369,273	T	C	0.957	0.92	RUNX1
rs73900765	21	36,369,342	C	T	0.889	0.997	RUNX1
rs73900767	21	36,369,446	C	T	0.68	0.996	RUNX1
rs36087189	21	36,370,073	C	A	0.889	0.995	RUNX1
rs28360527	21	36,370,185	T	C	0.763	0.99	RUNX1
rs184418280	21	36,370,995	T	C	0.983	0.901	RUNX1
rs375669842	21	36,371,028	T	C	0.987	0.86	RUNX1
rs189358252	21	36,371,089	T	C	0.976	0.954	RUNX1
rs180853897	21	36,371,091	C	G	0.976	0.956	RUNX1
rs78618702	21	36,371,129	T	C	0.775	0.967	RUNX1
rs13046934	21	36,371,207	G	A	0.765	0.991	RUNX1
rs13051552	21	36,372,118	T	C	0.723	0.994	RUNX1
rs199907894	21	36,373,584	TA	T	0.775	0.991	RUNX1
21:36373605_TTATATATATA_T	21	36,373,605	TTATATATATA	T	0.8	0.952	RUNX1
rs11702508	21	36,373,790	A	T	0.892	0.991	RUNX1
rs59121812	21	36,374,383	T	C	0.736	0.997	RUNX1
21:36374679_TTTTA_T	21	36,374,679	TTTTA	T	0.357	0.847	RUNX1
rs181280001	21	36,374,756	G	A	0.989	0.985	RUNX1
rs4305348	21	36,374,969	T	C	0.316	0.971	RUNX1
rs34468007	21	36,375,080	G	T	0.778	0.993	RUNX1
rs11700942	21	36,375,594	G	A	0.777	0.993	RUNX1
rs59293947	21	36,375,750	T	C	0.733	0.992	RUNX1
21:36375816_CTT_C	21	36,375,816	CTT	C	0.891	0.981	RUNX1
21:36375825_TTTA_T	21	36,375,825	TTTA	T	0.958	0.64	RUNX1
rs58218340	21	36,375,826	TTA	T	0.856	0.965	RUNX1
rs56878448	21	36,375,827	T	A	0.982	0.639	RUNX1
rs55901652	21	36,376,128	C	G	0.737	0.993	RUNX1
rs75865116	21	36,376,621	A	G	0.961	0.984	RUNX1
rs2834720	21	36,376,839	G	A	0.726	0.99	RUNX1
rs2834721	21	36,376,860	T	C	0.725	0.99	RUNX1
rs73900770	21	36,377,080	A	T	0.892	0.986	RUNX1
rs117420669	21	36,377,094	T	G	0.988	0.914	RUNX1
rs2834722	21	36,377,139	A	C	0.725	0.99	RUNX1
rs7282535	21	36,377,221	A	T	0.873	0.989	RUNX1
rs78571420	21	36,377,390	T	A	0.961	0.983	RUNX1
rs2834723	21	36,377,560	A	C	0.765	0.988	RUNX1
rs6517269	21	36,378,476	T	C	0.719	0.988	RUNX1
rs734382	21	36,378,571	A	G	0.72	0.987	RUNX1
rs734383	21	36,378,615	G	T	0.721	0.987	RUNX1
rs2834724	21	36,378,839	T	G	0.72	0.987	RUNX1
rs2834725	21	36,378,874	A	G	0.724	0.988	RUNX1
rs146113005	21	36,378,938	C	T	0.983	0.948	RUNX1
rs79213184	21	36,379,375	C	G	0.96	0.982	RUNX1
rs11701223	21	36,379,500	C	T	0.891	0.983	RUNX1
rs11700551	21	36,379,871	C	G	0.851	0.983	RUNX1
rs2834726	21	36,380,048	A	G	0.911	0.914	RUNX1
rs75034227	21	36,380,122	G	A	0.96	0.981	RUNX1
rs2834727	21	36,380,344	T	C	0.847	0.982	RUNX1
rs2834728	21	36,380,372	A	G	0.847	0.982	RUNX1
rs11700843	21	36,380,543	T	C	0.846	0.981	RUNX1
rs2834729	21	36,380,852	G	A	0.848	0.982	RUNX1
rs34937724	21	36,381,250	A	G	0.847	0.981	RUNX1
rs2242882	21	36,381,815	C	T	0.888	0.979	RUNX1
rs932285	21	36,382,081	A	G	0.848	0.98	RUNX1
rs932284	21	36,382,334	G	A	0.888	0.978	RUNX1
rs7281510	21	36,382,460	C	T	0.849	0.977	RUNX1
21:36382492_GT_G	21	36,382,492	GT	G	0.85	0.956	RUNX1
rs2834730	21	36,382,552	A	G	0.839	0.965	RUNX1
rs2834731	21	36,383,142	T	C	0.794	0.936	RUNX1
rs9984871	21	36,383,602	A	T	0.595	0.975	RUNX1
rs9976900	21	36,384,363	G	T	0.547	0.99	RUNX1
rs2834732	21	36,386,727	C	T	0.63	0.97	RUNX1
rs2242885	21	36,386,766	C	T	0.702	0.967	RUNX1
rs76691544	21	36,386,924	T	C	0.907	0.999	RUNX1
rs11700637	21	36,387,466	C	T	0.955	0.979	RUNX1
rs881387	21	36,387,561	G	T	0.591	0.989	RUNX1
rs2242888	21	36,387,828	T	C	0.54	0.997	RUNX1
rs11088303	21	36,388,176	G	A	0.545	0.998	RUNX1
rs882776	21	36,388,287	C	T	0.546	0.998	RUNX1
rs77728098	21	36,389,539	C	T	0.922	0.989	RUNX1
rs2834733	21	36,389,603	C	T	0.664	0.987	RUNX1
rs2834734	21	36,389,629	A	G	0.928	0.997	RUNX1
rs2834735	21	36,389,938	T	C	0.612	0.997	RUNX1
rs111252602	21	36,390,428	T	C	0.928	0.997	RUNX1
rs2834736	21	36,390,941	G	T	0.663	0.986	RUNX1
rs56285302	21	36,392,356	T	C	0.928	0.996	RUNX1
rs73203043	21	36,393,747	G	C	0.928	0.996	RUNX1
rs12483476	21	36,394,680	T	C	0.907	0.991	RUNX1
rs11088304	21	36,395,061	G	A	0.615	0.995	RUNX1
rs114860532	21	36,395,358	G	A	0.988	0.982	RUNX1
rs117469119	21	36,395,468	T	G	0.988	0.987	RUNX1
rs2242891	21	36,395,829	C	G	0.472	0.997	RUNX1
rs2242892	21	36,395,974	G	A	0.928	0.994	RUNX1
rs2242893	21	36,396,015	T	C	0.472	0.997	RUNX1
rs2834737	21	36,396,167	A	G	0.472	0.997	RUNX1
rs1888451	21	36,396,218	T	C	0.4	0.998	RUNX1
rs56117721	21	36,398,586	T	A	0.928	0.993	RUNX1
rs2834738	21	36,399,762	T	C	0.607	0.991	RUNX1
rs7282591	21	36,400,030	C	T	0.607	0.991	RUNX1
rs1883067	21	36,400,176	T	C	0.927	0.989	RUNX1
rs75967349	21	36,400,441	C	G	0.95	0.987	RUNX1
rs73203049	21	36,400,742	G	A	0.927	0.989	RUNX1
rs2242894	21	36,401,856	C	T	0.611	0.99	RUNX1
rs76356722	21	36,402,891	A	G	0.88	0.992	RUNX1

SNP	Chromosome	Position	Effect Allele	Other Allele	Effect Allele Frequency	Imputation quality	Gene
rs4257458	21	36,404,290	A	G	0.609	0.989	RUNX1
rs113530905	21	36,405,419	C	CTCAT	0.607	0.978	RUNX1
rs12482247	21	36,405,465	T	C	0.476	0.99	RUNX1
rs75102343	21	36,405,472	C	G	0.965	0.913	RUNX1
rs1474479	21	36,405,666	C	T	0.608	0.988	RUNX1
rs143934231	21	36,405,694	T	A	0.977	0.889	RUNX1
rs9979477	21	36,405,895	T	C	0.604	0.988	RUNX1
rs56358585	21	36,406,114	A	T	0.927	0.985	RUNX1
rs9980210	21	36,406,550	T	C	0.604	0.986	RUNX1
rs528900294	21	36,406,750	A	C	0.899	0.595	RUNX1
rs2242895	21	36,406,917	G	C	0.603	0.985	RUNX1
rs73203053	21	36,407,177	C	T	0.927	0.982	RUNX1
rs73203055	21	36,407,530	C	G	0.927	0.98	RUNX1
rs2294162	21	36,408,627	T	G	0.604	0.977	RUNX1
rs58527026	21	36,408,785	G	A	0.926	0.968	RUNX1
rs2834739	21	36,408,913	A	G	0.4	0.977	RUNX1
rs756596194	21	36,408,919	AT	A	0.871	0.983	RUNX1
rs78663639	21	36,409,172	C	T	0.871	0.992	RUNX1
rs1882732	21	36,409,284	A	G	0.868	0.993	RUNX1
rs11700684	21	36,409,832	T	G	0.926	0.968	RUNX1
rs3746865	21	36,411,119	G	T	0.812	0.971	RUNX1
rs61750966	21	36,411,127	C	T	0.927	0.963	RUNX1
rs11701383	21	36,411,387	G	A	0.867	0.995	RUNX1
rs1883066	21	36,412,156	C	G	0.871	0.99	RUNX1
rs2236431	21	36,413,739	A	C	0.537	0.953	RUNX1
21:36413957_CTT_C	21	36,413,957	CTT	C	0.423	0.753	RUNX1
rs28665366	21	36,414,027	T	C	0.861	0.968	RUNX1
rs147331040	21	36,414,320	C	A	0.989	0.82	RUNX1
rs11701532	21	36,414,780	C	G	0.927	0.939	RUNX1
rs7279123	21	36,415,087	T	C	0.274	0.86	RUNX1
21:36417495_CT_C	21	36,417,495	CT	C	0.853	0.93	RUNX1
rs143365361	21	36,418,245	T	A	0.985	0.952	RUNX1
rs9978978	21	36,418,601	T	C	0.909	0.994	RUNX1
rs7277157	21	36,419,783	T	C	0.821	0.985	RUNX1
rs533547690	21	36,419,862	T	A	0.848	0.802	RUNX1
rs75759495	21	36,419,920	C	A	0.914	0.988	RUNX1
rs73900786	21	36,420,534	A	C	0.959	0.964	RUNX1
rs12627198	21	36,420,786	C	T	0.854	0.996	RUNX1
rs73900787	21	36,420,874	C	T	0.959	0.965	RUNX1
rs9981811	21	36,420,875	G	A	0.909	0.988	RUNX1
rs8133634	21	36,421,036	T	C	0.499	0.98	RUNX1
rs56045941	21	36,421,331	T	C	0.828	0.982	RUNX1
rs56031201	22	39,619,814	G	C	0.981	0.954	PDGFB
rs9611110	22	39,619,897	G	A	0.979	0.998	PDGFB
rs56133813	22	39,620,641	T	C	0.974	0.956	PDGFB
rs2285099	22	39,622,058	C	T	0.58	0.999	PDGFB
rs2285097	22	39,622,334	T	C	0.58	0.999	PDGFB
rs2285096	22	39,622,390	T	C	0.58	0.999	PDGFB
rs2285095	22	39,622,541	A	G	0.58	0.999	PDGFB
rs2285094	22	39,622,560	T	C	0.58	0.999	PDGFB
rs4990914	22	39,622,711	C	T	0.58	0.999	PDGFB
rs4990915	22	39,622,712	A	G	0.58	0.999	PDGFB
rs4990916	22	39,622,836	A	G	0.58	0.998	PDGFB
rs4990917	22	39,622,837	T	C	0.58	0.998	PDGFB
rs4990918	22	39,622,939	C	A	0.58	0.999	PDGFB
rs4990919	22	39,622,940	T	C	0.339	0.996	PDGFB
rs4990920	22	39,622,986	T	C	0.339	0.996	PDGFB
rs4990921	22	39,622,991	G	T	0.58	0.999	PDGFB
rs4990922	22	39,623,113	A	G	0.58	0.999	PDGFB
rs2003809	22	39,623,389	A	G	0.573	0.999	PDGFB
rs4416326	22	39,623,725	C	T	0.766	0.996	PDGFB
rs9611115	22	39,623,779	A	G	0.58	1	PDGFB
rs9607624	22	39,623,812	A	G	0.58	1	PDGFB
rs9611117	22	39,624,105	T	G	0.58	1	PDGFB
rs745842239	22	39,624,171	TACTC	T	0.705	0.995	PDGFB
rs9611118	22	39,624,212	A	G	0.58	1	PDGFB
rs5757570	22	39,624,238	C	T	0.766	0.996	PDGFB
rs9611119	22	39,624,248	G	T	0.58	1	PDGFB
rs9611120	22	39,624,498	G	C	0.58	0.999	PDGFB
rs9611121	22	39,624,722	C	T	0.58	0.998	PDGFB
rs9607625	22	39,624,768	T	C	0.58	0.999	PDGFB
rs7288082	22	39,624,866	A	C	0.58	0.998	PDGFB
rs6001509	22	39,624,900	T	A	0.507	0.884	PDGFB
rs58510506	22	39,624,910	T	G	0.64	0.915	PDGFB
rs568270600	22	39,624,914	G	T	0.646	0.918	PDGFB
rs9622978	22	39,625,068	G	T	0.58	0.998	PDGFB
rs9611122	22	39,625,221	T	G	0.58	0.998	PDGFB
rs9611123	22	39,625,320	T	G	0.58	0.998	PDGFB
rs2285100	22	39,625,676	T	C	0.582	0.997	PDGFB
rs2239769	22	39,626,024	C	T	0.58	0.998	PDGFB
rs2239768	22	39,626,419	C	T	0.58	0.997	PDGFB
rs4821875	22	39,626,823	G	A	0.58	0.995	PDGFB
rs4821876	22	39,626,926	C	G	0.58	0.994	PDGFB
rs55634318	22	39,627,630	G	C	0.987	0.964	PDGFB
rs118089209	22	39,628,698	T	C	0.963	0.97	PDGFB
rs36091905	22	39,629,373	G	A	0.986	0.939	PDGFB
rs55823804	22	39,629,854	G	A	0.972	0.972	PDGFB
rs5845416	22	39,630,017	C	CGGAG	0.307	0.967	PDGFB
rs2247128	22	39,630,287	A	G	0.325	0.985	PDGFB
rs879180	22	39,631,547	T	C	0.248	0.983	PDGFB
rs56180415	22	39,631,963	G	T	0.919	0.983	PDGFB
rs6001512	22	39,632,523	G	A	0.919	0.984	PDGFB
rs5757572	22	39,632,920	C	G	0.37	0.988	PDGFB
rs9607626	22	39,633,479	G	A	0.98	0.99	PDGFB
rs5757573	22	39,633,622	C	T	0.368	0.989	PDGFB
rs2267406	22	39,633,749	T	C	0.256	0.986	PDGFB
rs5750781	22	39,634,434	A	C	0.288	0.99	PDGFB
rs71319025	22	39,634,444	C	A	0.922	0.987	PDGFB
rs5750782	22	39,634,923	A	G	0.281	0.99	PDGFB
rs77247985	22	39,635,432	C	G	0.969	0.953	PDGFB
rs3985946	22	39,635,858	G	GCA	0.367	0.975	PDGFB
rs34350734	22	39,636,510	T	TG	0.44	0.974	PDGFB
rs2857402	22	39,636,586	C	G	0.278	0.989	PDGFB
rs5757575	22	39,636,928	A	G	0.36	0.988	PDGFB
rs5757576	22	39,636,930	A	G	0.366	0.979	PDGFB
22:39636942_ACACACT_A	22	39,636,942	ACACACT	A	0.822	0.702	PDGFB
22:39639257_GTCTCTC_G	22	39,639,257	GTCTCTC	G	0.313	0.972	PDGFB
rs147322884	22	39,639,550	G	C	0.988	0.928	PDGFB
rs1800817	22	39,639,853	G	T	0.29	0.993	PDGFB
rs1800818	22	39,640,703	C	T	0.371	0.993	PDGFB
