# Supplementary material for: Lung development genes, adult lung function and cardiovascular comorbidities
Source: Thorax. 2025 May 30;80(10):e222474. doi: 10.1136/thorax-2024-222474 (PMC12505039; doi:10.1136/thorax-2024-222474)
Supplement: visual abstract [file thorax-80-10-s003.pptx]

## Slide 1
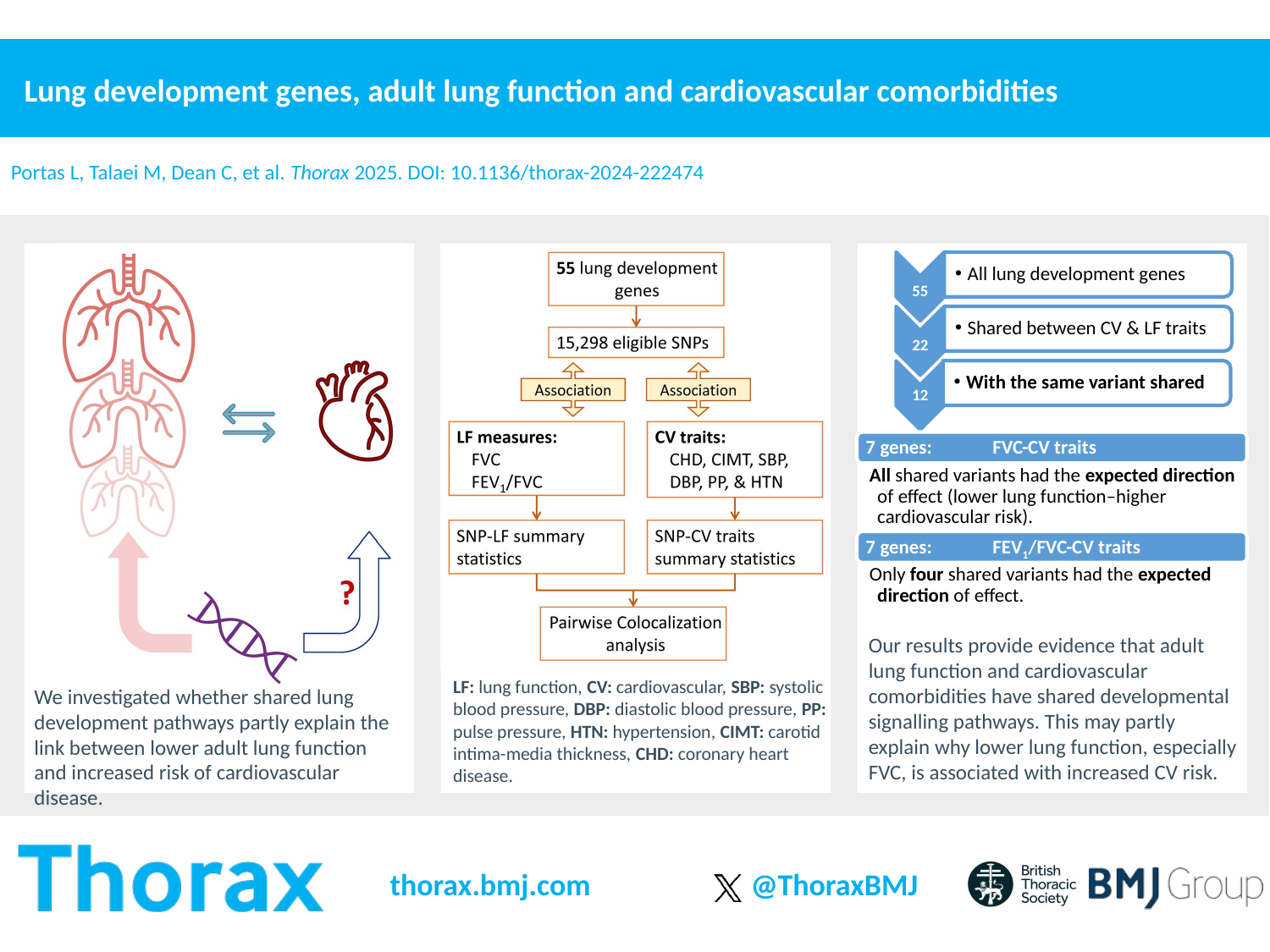

Lung development genes, adult lung function and cardiovascular comorbidities
Portas L, Talaei M, Dean C, et al. Thorax 2025. DOI: 10.1136/thorax-2024-222474
Manuscript Title
Our results provide evidence that adult lung function and cardiovascular comorbidities have shared developmental signalling pathways. This may partly explain why lower lung function, especially FVC, is associated with increased CV risk.
LF: lung function, CV: cardiovascular, SBP: systolic blood pressure, DBP: diastolic blood pressure, PP: pulse pressure, HTN: hypertension, CIMT: carotid intima-media thickness, CHD: coronary heart disease.
We investigated whether shared lung development pathways partly explain the link between lower adult lung function and increased risk of cardiovascular disease.
© Author(s) (or their employer(s) 2019. Re-use permitted under CC BY. Published by BMJ.
thorax.bmj.com @ThoraxBMJ
